# Characterisation of the Complete Mitochondrial Genome of Critically Endangered *Mustela lutreola* (Carnivora: Mustelidae) and Its Phylogenetic and Conservation Implications

**DOI:** 10.3390/genes13010125

**Published:** 2022-01-10

**Authors:** Jakub Skorupski

**Affiliations:** 1 Institute of Marine and Environmental Sciences, University of Szczecin, Adama Mickiewicza 16 St., 70-383 Szczecin, Poland; jakub.skorupski@usz.edu.pl; Tel.: +48-91-444-16-85; 2 Polish Society for Conservation Genetics LUTREOLA, Maciejkowa 21 St., 71-784 Szczecin, Poland; 3 The European Mink Centre, 71-415 Szczecin, Poland

**Keywords:** conservation genetics, European mink, mitochondrial DNA, mitogenome, mitogenomics, mtDNA, next-generation sequencing, phylomitogenomics

## Abstract

In this paper, a complete mitochondrial genome of the critically endangered European mink *Mustela lutreola* L., 1761 is reported. The mitogenome was 16,504 bp in length and encoded the typical 13 protein-coding genes, two ribosomal RNA genes and 22 transfer RNA genes, and harboured a putative control region. The A+T content of the entire genome was 60.06% (A > T > C > G), and the AT-skew and GC-skew were 0.093 and −0.308, respectively. The encoding-strand identity of genes and their order were consistent with a collinear gene order characteristic for vertebrate mitogenomes. The start codons of all protein-coding genes were the typical ATN. In eight cases, they were ended by complete stop codons, while five had incomplete termination codons (TA or T). All tRNAs had a typical cloverleaf secondary structure, except tRNA^Ser(AGC)^ and tRNA^Lys^, which lacked the DHU stem and had reduced DHU loop, respectively. Both rRNAs were capable of folding into complex secondary structures, containing unmatched base pairs. Eighty-one single nucleotide variants (substitutions and indels) were identified. Comparative interspecies analyses confirmed the close phylogenetic relationship of the European mink to the so-called ferret group, clustering the European polecat, the steppe polecat and the black-footed ferret. The obtained results are expected to provide useful molecular data, informing and supporting effective conservation measures to save *M. lutreola*.

## 1. Introduction

European mink *Mustela lutreola* L., 1761 (Carnivora: Mustelidae) is a semiaquatic, mainly nocturnal and solitary mammalian species [1,2]. This medium-sized carnivoran has an elongated, slender body with short limbs and tail [2]. Pelage colour is dark brown to black, with characteristic white spots on the upper and lower lips and chin, sometimes continuing down the neck, chest and stomach area [1,2]. European mink is a food generalist preying primarily on amphibians, small mammals, fish, birds, insects and crustaceans [3]. It inhabits a densely vegetated banks of streams, small rivers and lakes [4].

European mink populations were distributed in continental Europe by the 19th century, but have undergone a severe decline over the past 150 years [5]. Due to habitat loss, extensive commercial over-hunting for fur and competition with invasive non-native American mink *Neogale vison*, its range has been dramatically reduced by 97% and has shrunk to a few isolated populations, restricted to several locations in south-western France, northern Spain, the Danube Delta and the European part of Russia [5,6,7]. Reintroduced populations were established in Estonia (Hiiumaa Island) and Germany (Saarland and Lower Saxony) [5].

Ongoing population decline and a reduction in geographical range led to the classification of *M. lutreola* as a Critically Endangered Species on the IUCN Red List in 2011 [5]. To halt extinction and restore viable wild populations of this species, the European Association of Zoos and Aquaria (EAZA) Ex-situ Programme (EEP) for European mink was established in 1992 [8]. About 270 animals are being kept in captivity under this programme [9]. Several captive breeding and local reintroduction initiatives were launched in Estonia, France, Germany, Russia and Spain, and another is planned in Romania [9,10].

Rapid and ongoing population decline leads to reduction in gene flow, random genetic drift, inbreeding and, consequently, a decrease in genetic diversity [11]. Thus, small populations are threatened by genetic and demographic stochasticity, which, interacting with environmental factors, seriously elevate extinction risk [12,13]. For proper risk assessment, and hence, planning and implementation of effective countermeasures and mi-tigation measures, it is essential to use tools provided by conservation genetics [14]. Conservation genetics links knowledge on a species genetics with practice of its protection and conservation [15].

Research in the field of genetics of European mink is very limited [14] and needs to be urgently completed, especially in the context of the high extinction risk of the species. The rapidly disappearing genetic resources most likely will largely never be studied and described, which is an irreversible loss from cognitive and practical points of view—the meagre data on intra- and inter-population genetic diversity significantly impair the efficacy of the implemented activities for species restitution [14,16,17]. Such studies directly contribute to obtaining very valuable knowledge with high practical potential in terms of planning and implementing effective protective measures, both ex situ (conservation breeding) and in situ (translocations and reintroduction programmes) [8,16,18,19]. An extremely important applicatory aspect of genomic (including mitogenomic) research on species threatened with extinction should be emphasised [14,20,21,22].

Mitochondrial DNA sequences deposited at GenBank (http://www.ncbi.nlm.nih.gov/genbank/, accessed on 25 February 2021) include 43 records for partial and complete sequences of the *cytb* gene, *tRNA^Thr^* and *tRNA^Pro^* genes, gene for 12S rRNA, gene for NADH dehydrogenase subunit 2 and the D-loop (displacement loop) [23].

Fragments of the European mink’s mitochondrial genome are used in phylogenetic analyses. This applies to the following mitochondrial genes—*cytb* [24,25,26,27], *rrnS* [27,28] and *nad2* [27], as well as to the D-loop sequence [24,29]. However, the systematic position of this species at the genus and subgenus taxonomic level remains a debatable issue [14]. The full sequence of the mitogenome of *M. lutreola* has not been previously known. To date, complete mitogenomes have been sequenced for 10 out of 17 species of the genus *Mustela,* namely, *Mustela altaica*, *M. erminea*, *M. eversmannii*, *M. frenata*, *M. itatsi*, *M. kathiah*, *M. nigripes*, *M. nivalis*, *M. putorius* and *M. sibirica* [23,30].

Keeping in view this background, to fill the gap of knowledge on the European mink mitogenome, resolve its phylogeny, and provide genetic information supporting protection and conservation measures dedicated to this critically endangered species, I report, annotate and characterise for the first time the complete mitogenome sequence of *M. lutreola*. This includes a molecular analysis of the protein-coding genes (PCGs), RNA genes and non-coding regions. Furthermore, a comparative mitogenomic analysis of complete mitogenomes was performed for European mink and the selected caniforms, including abovementioned *Mustela* species. In addition, phylogenetic analysis, exploration of sequence variations and preliminary assessment of mitochondrial genome as an intraspecific variation genomic marker in *M. lutreola* was conducted.

## 2. Results

### 2.1. Mitochondrial Genome Structure, Organization and Composition

**Figure 1:**
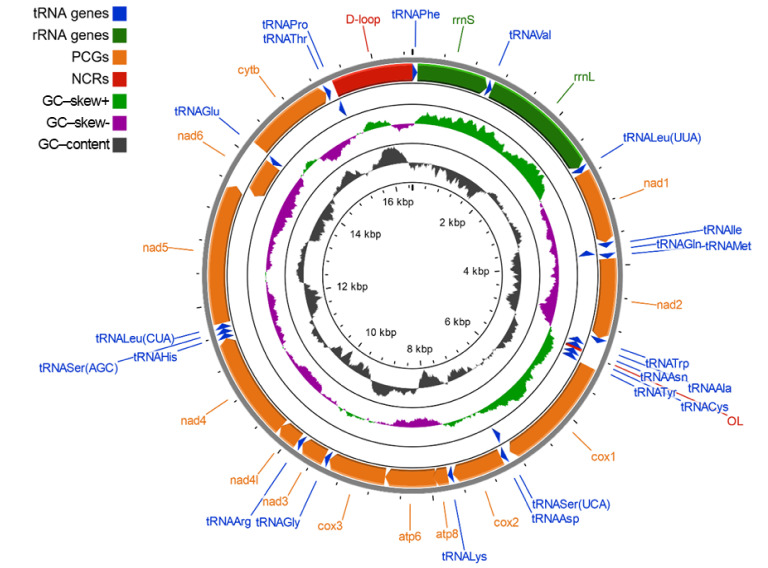
Graphical map of the complete mitochondrial genome of *Mustela lutreola*, drawn to scale as indicated by the innermost circle. Genes encoded by the heavy strand were shown outside, and encoded by the light strand inside the outermost circle respectively (arrows indicate the direction of gene transcription). Intermediate ring showed the GC–skewness (GC–skew is plotted using a green and purple sliding window, indicating its positive and negative values respectively). The GC–content is plotted using a dark grey sliding window, as the deviation from the average GC–content of the entire sequence; PCGs—protein-coding genes, NCRs—non–coding regions, O_L_—light strand replication origin.

The mitochondrial genome of European mink is a double-stranded, circular molecule 16,504 bp in length and of molecular weight equal to 10,196.19 kDa. It consists of 13 protein-coding genes (*nad1*, *nad2*, *cox1*, *cox2*, *atp8*, *atp6*, *co*x3, *nad3*, *nad4l*, *nad4*, *nad5*, *nad6*, *cytb*), 22 tRNA genes (one for each amino acid, two for leucine and serine), two rRNA genes (gene for 12S rRNA, *rrnS*; and 16S rRNA, *rrnL*) and a major non-coding region, known as the control region (CR; DLP, D-loop and associated promoters). Protein-coding genes account for 68.7% of the mitogenome, tRNA genes 9.1% and rRNA genes 15.3%, while non-coding regions cover 6.8% of the *M. lutreola* mtDNA. Most PCGs, tRNA genes and rRNA genes are encoded on the heavy strand (H-strand), except the *nad6* and eight tRNA genes (*tRNA^Gln^*, *tRNA^Ala^*, *tRNA^Asn^*, *tRNA^Cys^*, *tRNA^Tyr^*, *tRNA^Ser(UCA)^*, *tRNA^Glu^*, *tRNA^Pro^*), encoded on the light strand (L-strand). Features of the *M. lutreola* mitochondrial genome are summarised in Figure 1 and Table 1.

Mitochondrial genes are compactly arranged—some genes overlap each other, and only few, very short intergenic separators were found (Table 1). Overlapping genes include the following pairs: *tRNA^Val^*–*rrnL*, *tRNA^Ile^*–*tRNA^Gln^*, *cox1*–*tRNA^Ser(UCA)^*, *atp8*–*atp6*, *atp6*–*cox3*, *nad4l*–*nad4*, *nad5*–*nad6*, *tRNA^Thr^*–*tRNA^Pro^*. Only four (*tRNA^Val^*–*rrnL*, *atp8*–*atp6*, *atp6*–*cox3*, *nad4l*–*nad4*) overlap on the same strand. The longest, 43 bp sequence overlap, is shared by the gene for ATPase8 and ATPase6 (Table 1).

**Figure 2:**
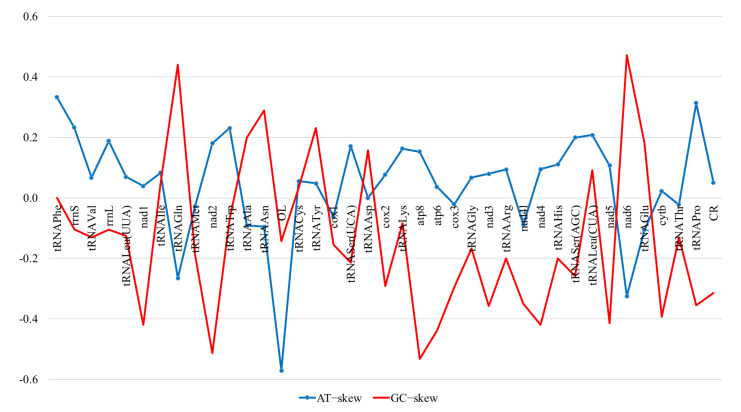
AT- and GC-skewness of different regions of the *Mustela lutreola* mitogenome (O_L_—light strand replication origin, CR—control region).

The overall base composition of the European mink mitogenome (H-strand), in descending order, is 5417 A (32.82%), 4496 T (27.24%), 4309 C (26.11%) and 2282 G (13.83%), which demonstrates a bias towards A and T nucleotides (60.06%). However, different regions have different G+C contents, ranging from 60% (light strand replication origin) to 21.74% (*tRNA^His^*). The overall highest content of Gs and Cs was recorded in the non-coding regions (44.34%) and the lowest in the tRNA genes (36.24%), whereas the protein-coding genes and the rRNA genes contained 40.17% and 38.85% of G + C nucleotides, respectively. The AT-skewness for the heavy strand was slightly positive (0.093), indicating the occurrence of more As than Ts, whereas the GC-skewness value was negative (−0.308), indicating the presence of more Cs than Gs (bias towards A and C). The highest values of the AT-skew and GC-skew had the rRNA genes (0.205) and the tRNA genes (−0.022), respectively, while the lowest values had the non-coding regions (0.033) and the protein-coding genes (−0.331), respectively (Figure 2). The nucleotide composition and skews for individual regions of the *M. lutreola* mitogenome are summarised in Supplementary Table S1. A putative CpG reach region (window size = 100; length of an island > 200; observed/expected CpG dinucleotides ratio > 0.6; C+G percentage > 50.0), length of 283 bp, was identified at position 15,975–16,257 bp (Supplementary Figure S1).

### 2.2. Repetitive and Palindromic Sequences

A total of 67 tandem repeats of more than 6 bp were identified in the *M. lutreola* mitogenome (Supplementary Table S2). The length of the repeat units in these regions varied between 6 and 160 bp (213 bp including mismatches), repeated in 2 to 22.1 copies. The longest and most complex repetitive DNA region (minisatellite) was found at position 16,020–16,253 bp, with the following consensus pattern: 5′-GCACACGTAC-3′ (period size: 10, copy number: 22.1, matches: 96%).

In addition to the direct repeats, 97 short inverted repetitive sequences (SIRs) were detected (Supplementary Table S3). The lengths of repeat motifs were 6, 7, 8 and 9 in 76, 11, 6 and 3 cases, respectively, and 11 bp in one case. The inverted repetitive sequences were evenly distri-buted throughout the mitogenome; however, the longest and most complex inverted repeat region of 146 bp was located at position 16,035–16,180 bp and overlapped with the previously mentioned minisatellite sequence. It included the 5′-CGTACG-3′ motif interspersed with the 5′-CACA-3′ sequence.

The detected palindrome sequences were also characterised by an evenly spaced pattern (Supplementary Table S4). A total of 301 palindromes were found, 6 (234 cases), 8 (56 cases), 10 (9 cases) and 12 (2 cases) bp long. Distribution of repetitive and palindromic sequences in the mitochondrial genome of the European mink is shown in Supplementary Figure S2.

### 2.3. Protein-Coding Genes and Codon Usage

The total length of the PCGs was 11,410 bp (69.14% of the complete sequence of the European mink mitochondrial genome), with 3793 coded amino acids and 31 bp stop codons. Average A+T content was 59.83%, varying from 55.48% (*cox3*) to 70.59% (*atp8*). The AT-skew and the GC-skew were equal to 0.036 and −0.331, respectively, indicating bias towards A and C (Supplementary Table S1). Of 13 protein-coding genes, 12 were encoded on the heavy strand, while the *nad6* was encoded on the light strand. Three reading-frame overlaps were observed on the same strand: *atp8* and *atp6* shared 43 nucleotides, *atp6* and *cox3* shared one nucleotide, *nad4l* and *nad4* shared seven nucleotides (Table 1). All the PCGs started with the typical ATN codons. The start codon ATG was used for *nad1*, *cox1*, *cox2*, *atp8*, *atp6*, *cox3*, *nad4l*, *nad4*, *nad6* and *cytb*, while alternative start codons of the vertebrate mitochondrial code were used for *nad2* (ATT), *nad5* (ATT) and *nad3* (ATA). The *nad2*, *cox3* and *nad4* genes were terminated by a single T, *nad1* and *nad3* used truncated TA stop codon, while the rest of protein-coding genes ended with the complete termination codon TAA, with the exception of the *cytb* gene, which had an alternate termination codon AGA. The start and stop codons of the 13 PCGs in the mtDNA of *M. lutreola* are shown in Table 1.

The analysis of the nucleotide composition at each codon position of the concatenated 13 PCGs of European mink (including presumed polyadenylated incomplete termination codons [31]) demonstrated that the third codon positions had especially high A+T content (63.24%). The A+T content of three codon sites was significantly different, with the third codon site showing much higher A+T content than that of the first and the second sites. The most frequent nucleotide at the first and the third codon position was A, occurring for 31.66% and 41.93% of the codons, respectively, while at the second position, the most frequent nucleotide was T, occurring for 41.96% of the codons. A strong bias against G (only 6.52%) at the third codon position was observed. Nucleotide bias at different codon positions was also demonstrated by the AT- and GC-skewness, indicating the presence of more As than Ts at the first and the third codon positions and more Ts than As at the second position, as well as more Cs than Gs at all three positions (Supplementary Table S1).

**Figure 3:**
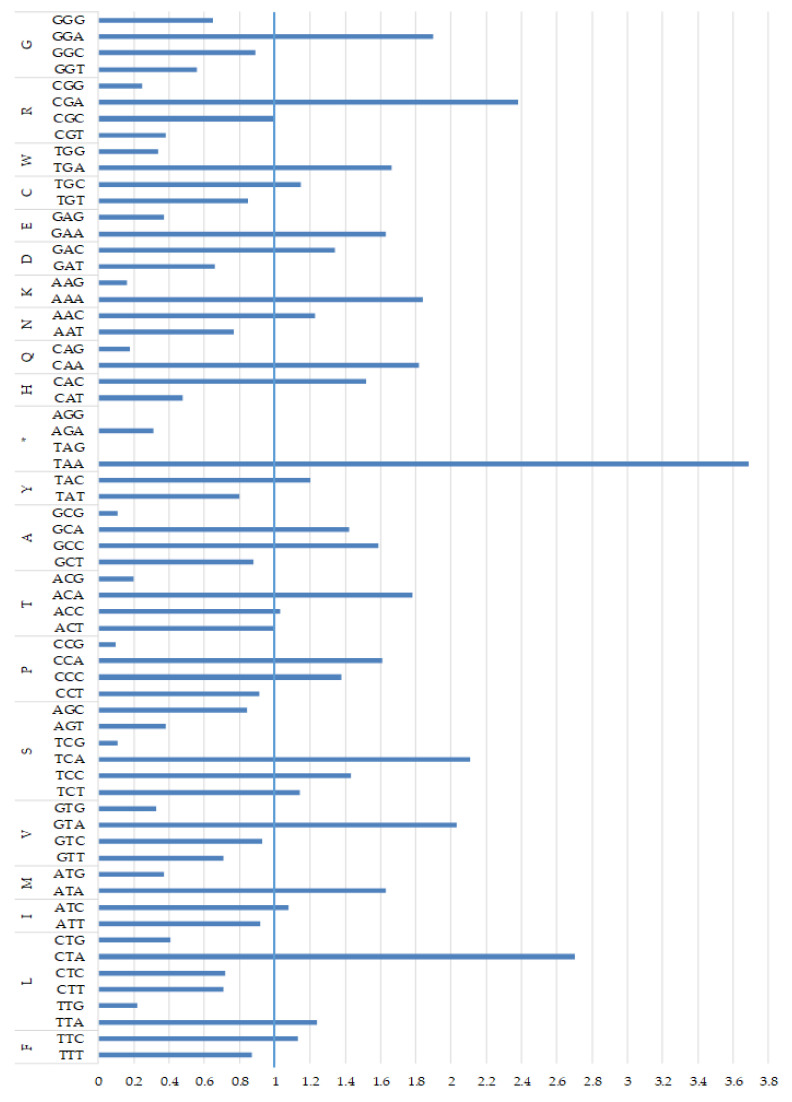
Relative synonymous codon usage (RSCU) in the mitochondrial genome of *Mustela lutreola*, including presumed polyadenylated incomplete termination codons [31] (RSCU = the actual number of synonymous codons used to translate specific amino acids/the expected number; when the observed values of synonymous codons are the same as the expected values, RSCU = 1, and the codons are not biased, when RSCU > 1, the codons are positively biased, and when RSCU < 1, the codons are negatively biased [32]; * termination codon).

The high A+T content (over 58.9%) and nucleotide bias in the PCGs were also reflected in codon usage, as the most frequently used were the following AT-rich codons: CTA (Leu), ATA (Met), ATC (Ile), ATT (Ile) and ACA (Thr), accounting for 7.20%, 5.28%, 4.65%, 3.99% and 3.73% of all codons, respectively (Table 2). Only two termination codons, TAG and AGG, were absent. The results of an analysis of the relative synonymous codon usage (RSCU), presented in Figure 3, further demonstrate a nucleotide composition bias in the *M. lutreola* mitogenome. The RSCU values of NNC and NNG codons are usually < 1 (in 18 out of 30 cases), while for NNA and NNT, codon values below and above 1 were found in the same number of cases. The appearance frequency of codons that ended with A or T (most frequent in case of 60% of synonymous codons for a given amino acid), as well as AT-rich codons (65% of aforementioned), was much higher than that of the other synonymous codons.

**Figure 4:**
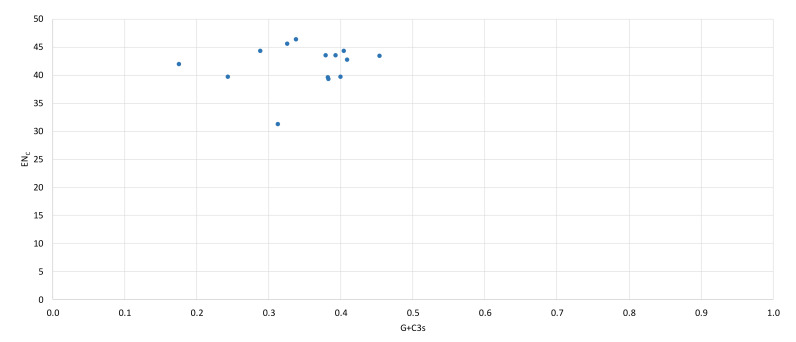
ENc-GC3s values plot for PCGs of the European mink mitochondrial genome (EN_C_—effective codon number, GC3—frequency of G−C nucleotides at the third codon position).

The value of an effective codon number (EN_C_), measuring the extent of codon pre-ference in a gene [33], for all the PCGs was equal to 44.34, indicating a strong codon bias, ranging from 31.27 to 46.36. The codon bias index (CBI), measuring the extent to which a gene uses a subset of optimal codons [34], for all protein-coding genes was 0.367, which confirms considerable codon bias. Additionally, to explain the relationship between nucleotide composition and codon bias EN_C_ and GC3s (frequency of G−C nucleotides at the third codon position), values for all protein-coding genes were plotted (Figure 4). The obtained result indicated that the codon bias in European mink mitochondrial genome might be shaped by other factors such as natural (translational) selection rather than by mutation bias only. Calculated values of EN_C_, GC3s and CBI indices are shown in Table 3.

**Figure 5:**
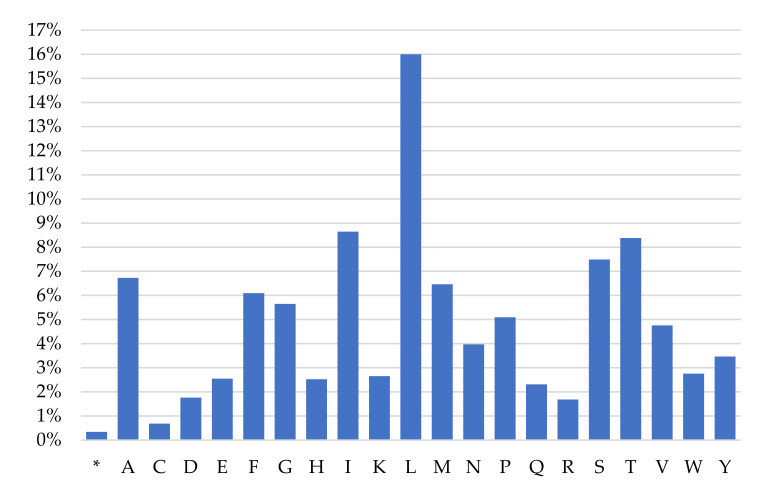
Codon frequency for individual amino acids in the mitochondrial protein-coding genes of European mink (presumed polyadenylated incomplete termination codons included [31]; * termination codon).

Leucine (16%), isoleucine (8.64%), tryptophan (8.38%), serine (7.49%) and alanine (6.73%) are the most frequent amino acids encoded by the mitochondrial PCGs of *M. lutreola*, while cysteine (0.68%), arginine (1.68%), aspartic acid (1.76%), glutamine (2.31%) and histidine (2.52%) are relatively scarce (Figure 5).

In the mtDNA sequence of *M. lutreola,* a total of 31 open reading frames longer than 75 codons were identified on the H-strand and 25 on the L-strand. They ranged in size between 78 and 1923 bp, and thus, their putative protein products had between 25 and 640 amino acids (Supplementary Table S5). The total number of identified ORFs far exceeded the total number of confirmed mitochondrial genes. ORFs that are not among the 13 canonical protein-coding genes recognised for the European mink mitogenome have no identifiable orthologs if translated and were thus considered unidentified reading frames (URFs) [35].

### 2.4. Transfer RNA Genes

The 22 tRNA genes (one specific for each amino acid and two for leucine and serine) were interspersed throughout the mitogenome. Fourteen of them were encoded on the H-strand and eight on the L-strand (Table 1). They ranged from 62 (*tRNA^Ser(AGC^*^)^) to 75 bp (*tRNA^Leu(UUA)^*) in size and showed a clear A+T bias (63.76%). The average A+T content in all tRNA genes is higher than that of protein-coding and rRNA genes. They also exhibit a slight skew of A versus T (AT-skew = 0.068) and C versus G (GC-skew = −0.022). The full length of tRNA genes was 1506 bp (Table 1 and Supplementary Table S1). Three conserved tRNA clusters were identified in the *M. lutreola* mitogenome: IQM (isoleucine, glutamine and methionine), WANCY (tryptophan, alanine, asparagine, cysteine and tyrosine), HSL (histidine, serine and leucine) [36].

All tRNAs were capable of folding into a canonical cloverleaf secondary structure, except tRNA^Ser(AGC)^, which lacked the dihydrouridine (DHU) stem. In addition, a DHU loop of the tRNA^Lys^ was reduced. Numerous mismatches (A-A, A-G, A-C and U-U) and non-complementary U-G weak bonds were found in the steam regions, as indicated in Table 4. The inferred tRNA cloverleaf structure contains 7 nt in the aminoacyl-acceptor stem, 3–5 nt in the TΨC stem, 4–5 nt in the anticodon stem and 3–5 nt in the DHU stem (Table 4). The inferred secondary structures for tRNAs are provided in Supplementary Figure S3.

### 2.5. Ribosomal RNA Genes

The mitochondrial genes encoding the large (16S) and small (12S) rRNA subunits (*rrnL* and *rrnS*) of *M. lutreola* were 1571 and 959 bp in size, with 61.30% and 60.90% A+T content, respectively, indicating A−T richness. The full length of rRNA genes was 2530 bp (Table 1 and Supplementary Table S1). Both rRNA genes had positive AT-skew and negative GC-skew. Both *rrnS* and *rrnL* genes were found on the H-strand of the mitochondrial genome. They were located between *tRNA^Phe^* and *tRNA^Leu(UUA)^* and were separated by *tRNA^Val^*, which shared two nucleotides with *rrnL*.

The predicted secondary structures of the two ribosomal RNA genes’ products are displayed in Figures S4 and S5. Both RNAs contain mismatches and non-canonical G-U base pairs, and have a complex secondary structure, including helices, hairpin loops, internal loops, multibranch loops and bulges.

### 2.6. Non-Coding Regions

Three types of non-coding sequences were identified in the *M. lutreola* mitochondrial genome: several ultra-short intergenic separators, the light strand replication origin, the control region.

A total of 11 short intergenic separators (excluding O_L_ sequence), ranging from 1 to 10 bp, were interspersed within the mitogenome, adding up to a total of 32 bp (Table 1). The longest intergenic spacer was found between *tRNA^Trp^* and *tRNA^Ala^*. The A+T content of this type of a non-coding sequence was higher (74.19%) than that of other regions in mitochondrial genome. Intergenic spacers also exhibited negative AT- and GC-skew (Supplementary Table S1).

**Figure 6:**
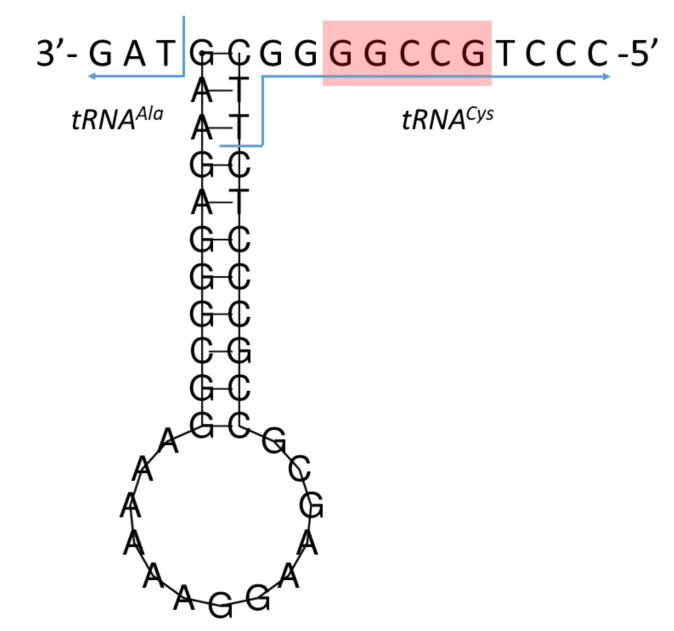
Predicted secondary structure of the L-strand replication origin (the 5′-GCCGG-3′ conserved motif indicated by a red box).

The putative L-strand replication origin was located within a conserved cluster of five tRNA genes, located between *tRNA^Asn^* and *tRNA^Cys^* (WANCY cluster), and starting at 5171 bp and ending at 5205 bp (Table 1). It comprised 35 nt in length, including three nucleotides overlapping with *tRNA^Cys^*, and was located on the L-strand. The O_L_ sequence was folded into a hairpin (stem-loop) secondary structure (Figure 6). The stem contained 11 bp and the loop contained 13 nt. A conserved motif 5′-GCCGG-3′ [36,37] was found in the 3′-end flanking region of the stem.

**Figure 7:**
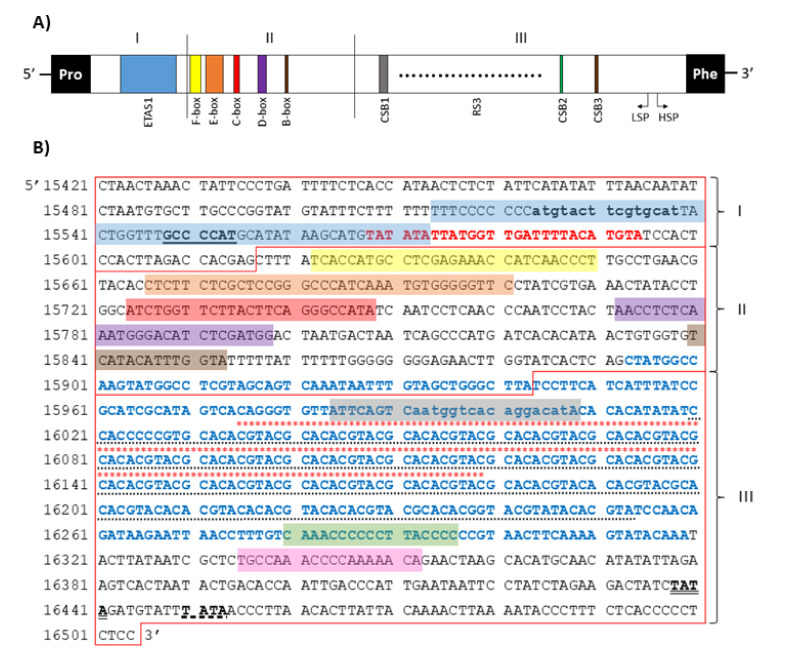
Schematic diagram of the organization (**A**) and nucleotide sequence (**B**) of the mtDNA control region of European mink (I—ETAS domain, II—CD domain, III—CSB domain; ETAS1 sequence indicated by a blue box; ATGN_9_CAT motif in bold lowercase; D-loop termination underlined; TAS-A sequence in bold red; F, E, D, C and B box sequence indicated by a yellow, orange, red, purple and brown box respectively; CSB1, CSB2 and CSB3 block sequence indicated by a grey, green and pink box respectively; RS3 region underlined by dots; CpG island region in bold blue; inverted repetitive sequences rich region indicated by red upper asterisks; LSP sequence double underlined; HSP sequence underlined with dashes; Pro—*tRNA^Pro^*; Phe—*tRNA^Phe^*).

The control region was the longest non-coding region and measured 1065 bp in length, with A+T content of 55.6%, showing a slight bias towards A and a strong bias towards C (Supplementary Table S1). DLP sequence was located between *tRNA^Pro^* and *tRNA^Phe^* genes and could be subdivided into a central conserved domain (CD; 15,617–15,943 bp) and two flanking variable domains, namely, ETAS (extended termination-associated sequence) domain (15,440–15,616 bp) and conserved sequence block (CSB) domain (15,944–16,505 bp) [38,39,40]. Within the ETAS domain, located at the 5′-end of the CR, an extended termination-associated sequence 1 (ETAS1—15,514–15,575 bp) [38,39], the D-loop termination motif (5′-GCCCCAT-3′—15,548–15,554 bp) [41] and a sequence with homology to the termination-associated sequence A (TAS-A—15,568–15,594 bp) [40,42] were identified. In the CD domain, five conserved boxes (F-box—15,624–15,651 bp, E-box—15,666–15,701 bp, D-box—15,724–15,748 bp, C-box—15,772–15,798 bp, B-box—15,838–15,854 bp) [39,43] were mapped. The CSB domain, positioned at the 5′-end of the CR, comprised three conserved sequence blocks (CSB1—15,984–16,008 bp, CSB2—16,279–16,296 bp, CSB3—16,334–16,352 bp) [39,44], as well as two putative transcriptional initiation promoters, i.e., heavy-strand promoter (HSP—16,438–16,441) and light-strand promoter (LSP—16,450–16,453) [38,42]. The organisation and structure of the control region are presented in Figure 7.

**Figure 8:**
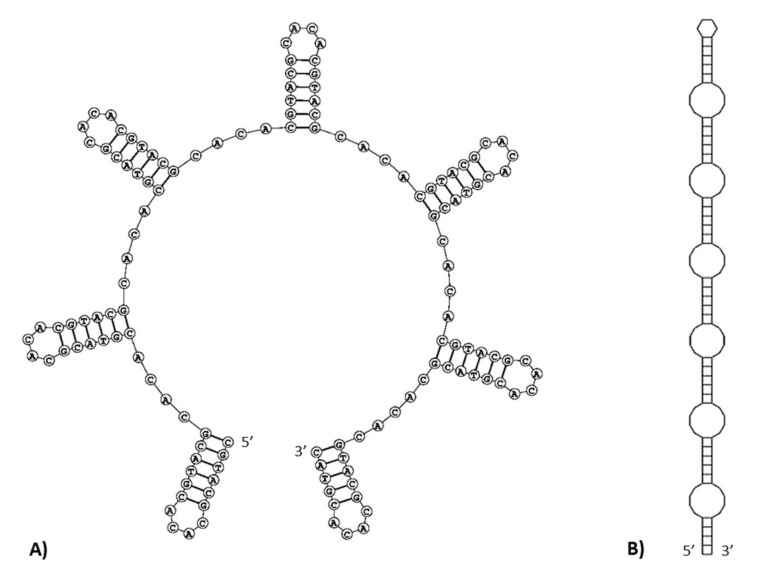
Putative single-strand hairpin structures predicted for an inverted repetitive sequences rich region within the control region of the European mink mitogenome ((**A**)—structure of a linear stem-loop set, (**B**)—simplified scheme of a contiguous stem structure).

In the *M. lutreola* mitogenome, microsatellite sequences were detected, such as (CT)_3_, (AT)_3_ (occurred in three locations), (TA)_3_, (CA)_3_ and (AC)_3_ (Supplementary Table S2). Four hexanucleotide (T_6_, G_6_ and two cases of C_6_), one heptanucleotide (C_7_), one octanucleotide (G_8_) and one nonanucleotide (T_9_) SSRs (simple sequence repeats) were identified in the control region. As previously mentioned, a 234 bp minisatellite region (consensus pattern 5′-GCACACGTAC-3′, imperfectly repeated 22.1 times), called RS3 [45], was identified between CSB1 and CSB2 blocks (16,020–16,253 bp). Within this region, an area rich in inverted repetitive sequences of 146 bp (motif (CGTACG)_15_(CACA)_16_) was found at position 16,035–16,180 bp (Figure 7). It was found to have the ability to form multiple single-strand hairpin structures. Two possible folding patterns were identified, a series of seven stem-loop structures separated by the 5′-CACA-3′ motif (Figure 8A) or a contiguous stem structure, consisting of a set of stems (5′-CGTACG-3′) linked together by six short internal loops and ended with a small end loop (Figure 8B). The free energy values for these secondary structure patterns were −44.0 kcal/mol and −56.6 kcal/mol, respectively. The A+C content was particularly high for an area rich in inverted repetitive sequences (70.1%), indicating an A-C richness in this region.

Additionally, two closely related 15 bp and evolutionarily conserved palindromic sequence motifs (ATGN_9_CAT) [46] were found in the ETAS domain (within the ETAS1 region) and the CSB domain (within the conserved sequence box 1) (Figure 7B). This motif can form stable secondary hairpin structures, with the stem containing 12 bp and the loop containing 3 nt. A CpG island was found at the 3’-end of the central conserved domain and in the CSB domain (Figure 7B).

### 2.7. Mitochondrial DNA Sequence Heterogeneity

Within the nucleotide mitogenomic sequence of six examined European mink, 54 single nucleotide variants (SNVs) were detected, comprising three transversions, 40 transitions and 11 insertion–deletion (indel) cases, as indicated in Supplementary Table S6. Out of 28 SNVs occurring in the PCGs, two were a nonsynonymous (missense) substitutions, while the remainder had a synonymous character (Supplementary Table S6).

**Figure 9:**
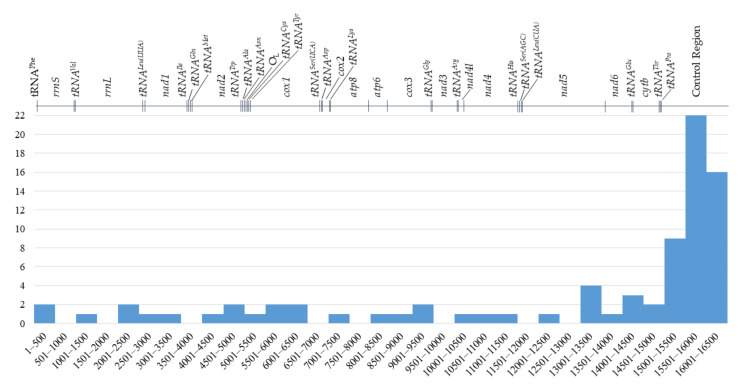
Distribution of single nucleotide variants (substitutions and indels) in the European mink mitogenome (X axis—position in bp, Y axis—number of variants).

The incidence of variable nucleotides within the identified mtDNA sequence was 1/305.6 bp (0.33% of nucleotides), while the coding regions for this parameter assumed a value of 1/480.5 bp (0.21% of nucleotides) and for non-coding regions 1/51.3 bp (1.95% of nucleotides). The control region was the most variable in the whole mitogenome and included 22 variable sites of single nucleotide variants and variable number tandem repeats (VNTRs). The incidence of variable sites in this region was 1/49.8 bp, which translates to 2% of the region’s sequence. The number of SNVs in the protein-coding genes varied from 1 (*cox2*, *nad6*) to 5 (*cox1*, *nad5*). Variable sites were not detected in any tRNA gene as well as in *atp8* and *nad4l* sequences (Figure 9). Both the L-strand replication origin and short intergenic spacers were also found to be monomorphic in the studied individuals. Identified VNTRs resulted in differences in the total length of the mitogenome sequence in stu-died individuals, ranging from 16,501 bp to 16,523 bp (an average of 16,508 bp).

**Figure 10:**
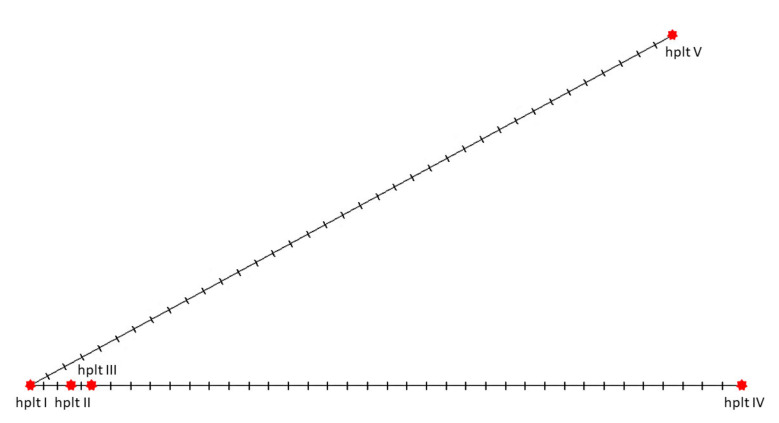
The median-joining network of five mitochondrial haplotypes identified in the studied individuals (hplt—haplotype; vertical bars indicate distinguishing variable positions; haplotype designation with Roman numerals explained in the text).

Nucleotide diversity (π) was 0.0326 and haplotype diversity (H_d_) was 0.9333. Sequence conservation index (C), reflecting the proportion of conserved (invariable, monomorphic) sites in the alignment sites, was equal to 0.9470, and that of the overall mean genetic distance was 0.03 (indicating the number of base differences per site from averaging over all sequence pairs). Twenty-five variable sites were considered parsimony-informative sites and allowed to identify five different haplotypes: I—MW197425, II—MW197426, III—MW148603, IV—MT304869 and MW197423, V—MW197424 (Figure 10).

Alignment of the complete mitochondrial genome with the 43 mtDNA sequences of *M. lutreola* deposited in the GenBank (complete and partial sequences of *cytb*, *nad2*, *rrnS*, *tRNA^Thr^*, *tRNA^Pro^* and control region, ranging in length from 337 to 1140 bp) revealed 27 additional SNVs, including 22 transitions, four transversions and one indel site (Supplementary Table S6, Figure 9). Multiple sequence alignment is presented on Supplementary Figure S6. One SNV was harboured by the *rrnS* and *tRNA^Pro^* gene each, four (including three missense substitutions) were found within the *cytb* sequence and 21 within the control region. When considering these variable sites as well, the overall incidence of SNVs within the *M. lutreola* mitogenome sequence was 1/203.8 bp (0.49% of nucleotides), within the coding regions 1/404.6 bp (0.25% of nucleotides) and within non-coding regions 1/26.2 bp (3.81% of nucleotides).

Two highly variable areas (putative hypervariable segments, HVSs [47]), located at the 3′-end of the control region, between 15,463 and 15,973 bp and between 16,170 and 16,232 bp, were identified. They were separated by an area rich in inverted repetitive sequences and were characterised by SNVs incidence equal to 1/18.9 bp and 1/3.9 bp, respectively (Supplementary Table S6).

### 2.8. Interspecies Comparative and Phylogenetic Analyses of the European Mink Mitogenome

The results of a comparison of the structural features of the European mink mitochondrial genome with the known mitogenomes of other representatives of the genus *Mustela*, and other selected Caniformia species are summarised in Table 5 and further discussed in the Discussion section.

Overall mean genetic distance for compared *Mustela* species was 0.64, while pairwise genetic distances for analysed species of this group are presented in Table 6. In total, 3653 variable sites were identified, comprising 1700 singleton variable sites and 1953 parsimony informative sites. The number of revealed indel sites was equal to 704. The average G+C content among species of the genus was equal to 0.396. Nucleotide diversity (π) was estimated at 0.0751, the sequence conservation (C) at 0.76, and the average number of nucleotide differences (k) at 1199.04. Four conserved regions in the control region were identified (Table 7).

**Figure 11:**
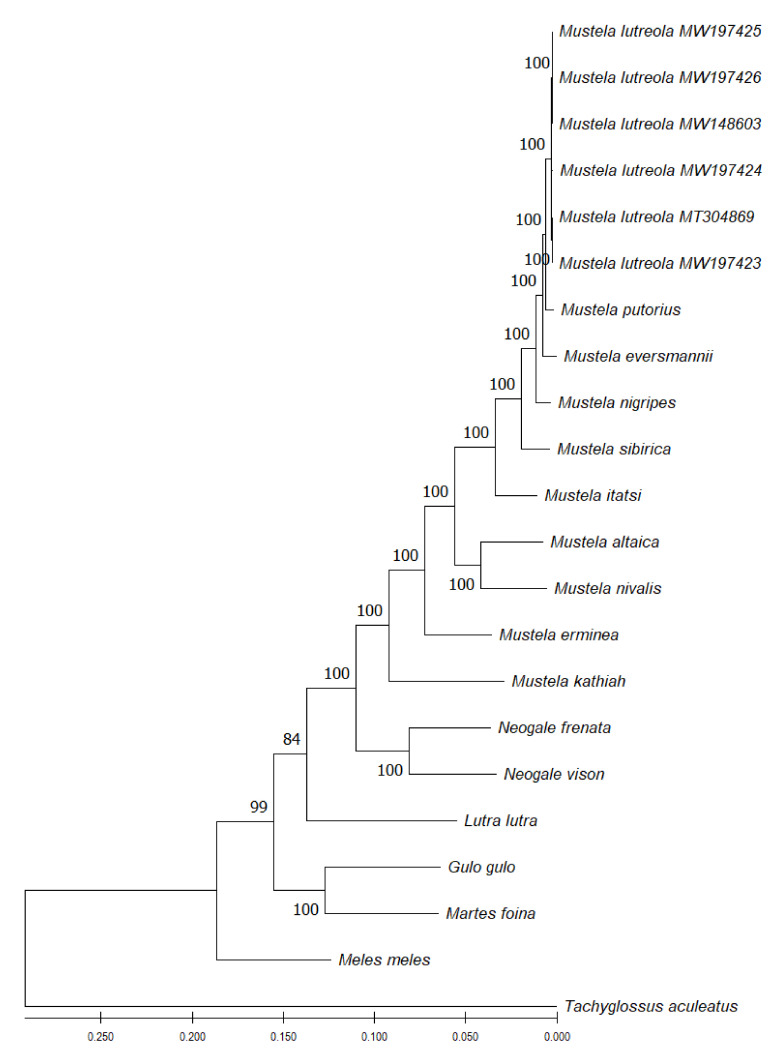
Phylogenetic tree generated by the Maximum Likelihood method and Tamura-Nei model in MEGA 11 based on complete mitochondrial genome sequence alignments of 11 reference sequences in the genus *Mustela* (six individuals of *M. lutreola*—the present study, *M. putorius* HM106318, *M. eversmanii* NC_028013, *M. nigripes* NC_024942, *M. sibirica* MN206976, *M. itatsi* NC_034330, *M. altaica* NC_021751, *M. nivalis* MF459691, *M. erminea* MW257230, *M. kathiah* HM106320, *M. frenata* HM106321), five other species of the mustelid family (*Neogale vison* NC_020641, *Lutra lutra* NC_011358, *Gulo gulo* NC_009685, *Martes foina* NC_020643, *Meles meles* NC_011125) and an outgroup species (*Tachyglossus aculeatus* AJ303116). The percentage of replicate trees in which the associated taxa clustered together in the bootstrap test (1000 replicates) is shown next to the branches. The tree is drawn to scale, with branch lengths measured in the number of substitutions per site.

Mitochondrial genome phylogenetic analyses based on the Maximum Likelihood, the Neighbour-Joining, the Minimum Evolution and the Maximum Parsimony methods yielded identical phylogenetic trees. *M.* *lutreola* cladded well in the so-called ferret group (clustering the European polecat, the steppe polecat and the black-footed ferret), with 100% bootstrap values (Figure 11). The group was closely related to *M. sibirica* and *M. itatsi*. More distant was the mountain weasel and the least weasel, clustered into a separate group, the stoat and the yellow-bellied weasel. All listed species formed a monophyletic lineage within the genus *Mustela* (supported by bootstrap values of 100%).

## 3. Discussion

### 3.1. General Features of the European Mink Mitochondrial Genome

The size of the European mink mitogenome ranks between the average for the known mitochondrial genomes of *Mustela* species (16,477 ± 145 bp) and of its closest evolutionary relatives [24,27,60], the polecat (ferret) group (*M. putorius*, *M. eversmanni*, *M. nigripes*) and Siberian weasel (16,521 ± 35 bp), as indicated in Table 5 [48]. It is worth noting that the variation in size of the known genomes of the *Mustela* species, expressed by the value of the standard deviation, is relatively small. The overall sequence length of known mustelid mitogenomes, excluding the control region, is nearly identical, as most variations in their size are due to differences in the D-loop and associated promoters sequence length [40,53,61]. Analysis of intergenic spacers and overlapping regions in the *M. lutreola* mitogenome indicated its compactness greater than in *Microtus* sp., *Vulpes vulpes* and *N. vison*, and less than in *Lepus yarkandensis* and *Lutra lutra* [36,57,62,63,64]. In general, the European mink mitogenome fits in well with the pattern observed in animals, according to which mtDNA is characterised as a highly genetically economised genome with intronless genes and only short sections of non-coding DNA and intergenic spacers [65,66].

In terms of the H-strand nucleotide composition, the order identified in this study, A > T > C > G, corresponds with the order characteristic for mitogenomes of Mustelidae species and, in a broader context, of other mammals [36,40,48,49,53,57,62,64,67,68,69]. The revealed base compositions were found to be skewed similarly to those of mitogenomes of other vertebrate sequences, with more A+T than G+C base pairs and higher deoxyadenosine monophosphate and deoxycytidine monophosphate contents in the L-strand [36,40,70]. A particularly high similarity in this respect is observed between *M. lutreola* and *M. putorius*, *M. eversmanni*, *M. nigripes*, *M. sibirica* and *M. itatsi* (Table 5) [48,49,50]. With regard to individual genes (regions) of the mitogenome, a particularly high similarity is observed for European mink and, for example, black-footed ferret [48].

The number of PCGs, as well as tRNA and rRNA genes in mitochondrial genome of *M. lutreola* is typical for mustelids, as well as most other animals [36,48,49,51,52,57,62,64,68,69,71,72]. The encoding-strand identity of the mitochondrial genes of European mink and their order in the mitochondrial chromosome is consistent with a collinear gene order characteristic for other vertebrate mitogenomes [36,48,57,62,64,73].

Although short tandem repeats (STRs) are usually reported in the control region [74,75,76,77,78], their presence was revealed also in other, including genic, regions of mitogenomes of many species [61,79,80,81,82,83]. Their distribution throughout the European mink’s mitochondrial genome is therefore consistent with the general regularity. The same applies to pa-lindromic sequences and inverted repeats, which are accurately described for many vertebrate species in the DLP region, but their distribution and role, mainly within coding sequences, are poorly understood [61,82,84,85,86]. However, also in other mammalian species, there are reports of their presence outside the control region [82,87,88]. The characteristics of repetitive and palindromic sequences in the *M. lutreola* mitogenome, claimed to be of regulatory, evolutionary and stabilizing importance, are discussed later in this section.

As is the case with European mink, variable start codons and incomplete stop codons of PCGs have been reported in mitogenomes of many species, including mustelids [31,36,48,49,52,62,69,89]. The most frequent start codon among the latter group is ATG, with some exceptions that vary from species to species, e.g., unlike *M. lutreola*, ATC is used as a start codon in *nad2* of *M. nivalis*, *N. vison* and *L. lutra;* ATA in *nad5* of *M. putorius*, *M. nigripes*, *M. sibirica*, *L. lutra* and *N. vison*—as well as in *atp6* of the latter species, and ATT in *nad3* of *M. nivalis* (GenBank Accession No. NC_020638, [36,48,49,52,62,69]). The most significant difference in this respect is the lack of the ATC start codon in the European mink mitogenome. The same situation occurs with the steppe polecat, which shows an identical pattern of mitochondrial PCGs’ start codons as European mink [GenBank Accession No. NC_028013]. Importantly, the initiation codon was ATN in all indicated cases, following the vertebrate mitochondrial code (translation Supplementary Table S1), and with regard to alternative initiation codons (in particular, ATT and ATA) [90,91].

As for the stop codons, *M. lutreola* follows the regularity common in mustelids, according to which the AGA codon terminates translation of mitochondrially encoded cytochrome b [36,48,49,52,62,69]. However, it does not have the TAG stop codon, described in American mink and Eurasian otter, or ATT, found in least weasel [40,52,62]. As the transcripts of several vertebrate mitochondrial genes end in incomplete stop codons (T-- and TA-), they become termination codons (TAA) upon subsequent, post-transcriptional polyadenylation at the 3′-end of mRNA [31,40]. Such incomplete stop codons are also commonly detected in mitochondrial genomes of other mustelids, although with some marked differences. In some previous mitogenome studies of other Mustelidae species, different rates of complete termination TAA codon usage were revealed, used in case of six PCGs in *M. putorius*, *M. eversmannii* and *M. nivalis*; eight in *M. nigripes* and *N. vison*; nine in *L. lutra*; while *M. sibirica* uses TAA as a stop codon as often as European mink (GenBank Accession No. NC_020638 and NC_028013, [40,48,49,52,54,62,69]).

A bias toward a higher content of nucleotides A and T and against G in PCGs is a common feature of metazoan mitogenomes observed also in European mink and other mustelids, and leads to a subsequent bias in the corresponding encoded amino acids (expressed by an EN_C_ and a CBI values) [36,40,92,93,94]. This is reflected in the fact that the third codon position is especially A+T-rich and NNT and NNA codons are usually the most frequent. A nucleotide composition bias in the *M. lutreola* mitogenome is further demonstrated by values of their relative synonymous codon usage, indicating that the lost codons are usually G+C-rich [95,96]. The relative synonymous codon usage reflects the phenomenon of mitogenome’s codon usage bias [97]. The obtained result supports the hypothesis according to which the codon usage bias in mitochondrial genomes may be positively correlated with the AT bias of the third codon position [98,99]. To explain what factors affect the observed synonymous codon usage bias (SCUB) among genes in differ-rent organisms, a plot of ENc versus GC3 content is widely used [33,100]. If codon usage variation among the genes is determined mainly not by translational selection, but compositional constraints, then values of the effective codon number would fall on the continuous, bell-shaped curve between EN_C_ value and GC3 content [33]. As for European mink most points in the ENc–GC3s values plot lay outside this curve (Figure 4), apart from mutation bias, other factors might also shape the codon usage bias of mitochondrial PCGs of this species [101].

The frequency of mitochondrial amino acids encoded in European mink (Figure 5) is consistent with findings for other mustelids, and more generally for metazoans, with leucine occurring with highest frequency, and cysteine with the lowest [36,57,61,62].

Additional open reading frames with no identifiable orthologs (URFs), identified in *M. lutreola* (Supplementary Table S5), were also reported in other organisms [102,103,104,105]. It was suggested that such previously undetected protein-coding genes may also occur in human mitochondrial genome [35,104,106]. Different mechanisms that could enable the mitochondrial genome to code for additional proteins without an increase in size were proposed, e.g., small open reading frames (sORF) in intergenic regions, encoding biologically active peptides, transcription of protein genes within rRNA genes, PCGs from different strands or from the same strand but in different reading frames [103,106].

The set of 22 tRNA genes detected in the mitogenome of *M. lutreola* showed a typical and conserved arrangement as found in most vertebrates [36,48,69]. Features of predicted secondary structure of the European mink tRNAs (typical cloverleaf pattern, stems and loops sizes, presence of some unmatched base pairs in stem regions, lack of the DHU loop in the tRNA^Ser(AGC)^; Supplementary Figure S3) are mostly identical to those found in other mammal mitogenomes [40,48,57,59,69,93,107]. As for mismatched base pairs, most of them are U-G, which were proven to form a weak bond in tRNAs [108]. Mismatched base pairs can also be corrected by RNA editing [109]. A special feature of *M. lutreola* in this regard is reduced DHU arm (forming a recognition site for aminoacyl-tRNA synthetase) of tRNA^Lys^ [110,111]. This was reported also for many bird mitogenomes, but not for mammals [112,113,114].

The secondary structure of both the 12S rRNA and the 16S rRNA predicted for European mink (Supplementary Figures S4 and S5) does not differ substantially in complexity and the fact of the occurrence of non-canonical nucleotides pairs from that of other mammalian species [89,115,116,117]. The lack of data on the secondary structure of rRNA in other mustelids, and their scarcity for members of the Carnivora order, indicates the need to complete the knowledge in this field and to conduct an in-depth structural analysis of mitochondrial ribosomal RNAs in these groups. The importance of this knowledge is primarily due to the fact that biological activity of rRNA is dependent on its structural conformation; it also allows identification of a functional sites within rRNA molecules, and is useful in phylogenetic analyses, as secondary structure models can be utilised to adjust the primary sequence alignment to increase positional homology [115,116,118].

Typically for mammalian mitogenomes [119], non-coding regions of European mink include intergenic spacers, the light strand replication origin and the control region. As in most vertebrates, the origin of L-strand replication in *M. lutreola* was within the conserved WANCY cluster [36]. Its length was within the range typical for mustelids, i.e., from 35 to 36 bp [36,40,52]. Like in other representatives of the Mustelidae family, the O_L_ sequence comprised a conserved 5′-GCCGG-3′ motif, known to be involved in the transition from RNA synthesis to DNA synthesis in human mitogenome [36,37,40], and had common potential to be folded into a stable hairpin structure [36]. It was evidenced that caniforms’ mitochondrial genomes are characterised by conservativeness of a stem regions and complementary structures of the origin of L-strand replication sequence, while minor variations in the loop sequence may occur [40]. For example, the Eurasian otter loop includes, unlike that of the European mink loop described in this paper, 14 nucleotides [40].

As in other mammals (except primates), the control region of the *M. lutreola* mitogenome was in accordance with the A+T > G+C pattern [38,120]. It shows all the main fun-ctional sites typical for vertebrates [45,121]. The nucleotide composition of this region was consistent with CR sequences described for other mustelids [39]. The degree of sequence homology of the functional motifs was high enough to identify them in the *M. lutreola* control region based on the known control regions of other representatives of the Mustelidae family [39,42,43]. The conserved sequences harboured by the putative control region of the *M. lutreola* mitochondrial genome are assumed to play a regulatory role in mtDNA replication and transcription. This highly structured region includes, as is typical for mammalian mtDNA, a conserved central domain, playing a role in the cleaving of the L-strand transcript, and two peripheral domains, exhibiting a high rate of both nucleotide substitutions and variation in copy number of tandem repeats (Figure 7) [42,45,122]. This three-domain structure is suggested to be conserved throughout more than 65 million years of placental mammals evolutionary history [123].

The ETAS1 sequence, mapped in the ETAS domain, showed a homology to the termination-associated sequences found in other mammals [42]. Within the ETAS1 sequence, a functional, conserved motif 5′-GCCCCAT-3′, being the D-loop stop point, was identified [41]. It has already been mapped in CRs of a variety of mammalian groups [41,45]. Termination-associated sequences (TASs) found in European mink flanked the TAS-A region and slightly differed from those found in *L. lutra*, i.e., the 5′-TACAT-3′ motif is replaced by 5′-TATAT-3′ in *M. lutreola* [40,43]. The TAS-A sequence, overlapping with the ETAS1 sequence, is involved in the termination of the H-strand replication and in the displacement of the original H-strand to create a three-stranded D-loop [42].

Both the boundaries of the central conserved domain as well as the conserved boxes in the European mink control region have been mapped based on well-recognised corresponding sectors of other mustelids mitogenomes, namely, *M. putorius*, *Martes zibellina*, *Martes flavigula*, *Vormela peregusna*, *L. lutra*, *Enhydra lutris*, *M. meles*, *Gulo gulo*, *Conepatus chinga*, *Conepatus leuconotus* and *Spilogale putorius* [39,42,43]. Although the conserved boxes of central domain are characterised by a high similarity level among all the carnivore taxa, they show different patterns of nucleotide substitutions and thus were proven to be helpful in resolving the phylogeny of carnivorans [42,121]. The usefulness of the mapped European mink control region, as well as the complete mitogenome, as a phylogenetic marker requires further research.

It was suggested that conserved sequence blocks (CSB1, CSB2, CSB3) of the CSB domain are functionally important for replication and transcription of mtDNA in the D-loop-containing region [124,125]. They are involved in positioning RNA polymerase for both transcription and priming replication [126,127]. The putative origin of the heavy strand replication (O_H_), arising from the proximity of the beginning of the CSB domain and nearby CSB1 [128], was not identified in the present study. For Eurasian otter, the motif 5′-CCCCGCCGC-3′ was proposed as a possible origin of the replication [43]. Within the CSB domain, two promoters, HSP and LSP, were identified in the European mink mitogenome, likewise in other mammals [38,42].

A specific feature of the European mink DLP region, not previously reported in other mustelids, is the presence of a CpG sector, with a high GC content and enriched for the CG dinucleotide (CpG islands), extending from the 3′-end of the central conserved domain through most of the CSB domain. A similar overall pattern of CpG islands distribution has been described in the *Canis lupus familiaris* mitogenome, where the CpG-rich region of 271 bp is located in the D-loop (between 16,179 and 16,449 bp) and covers the VNTR region. In the mitochondrial genomes of primates (*Pan troglodytes ellioti* and *Homo sapiens sapiens*), as well as *Danio rerio*, *Latimeria chalumnae*, *Crocodylus porosus* and *Gallus gallus,* CpG islands were identified most often in coding sequences (e.g., *rrnS*, *nad 5*, *cyt b*) [129]. CpG islands in the control region have been also described in humans [130,131]. It was shown that DNA methylation (epigenetic modification) occurs in CpG islands of mammalian mitogenomes and is involved in the regulation of gene expression, contributing to transcription, processing and decay of mitochondrial RNA [130,131,132]. The higher GC content in the non-coding control regions (42.2%, on average) compared to the coding sequences (37.5%, on average) is consistent with the proven regularity, according to which CpG islands are linked to biologically functional genomic elements [122,133].

The regulatory functions of the CSB domain are also proven by the presence of thermodynamically stable stem-loop structures [38,46,124]. An evolutionary conserved palindromic sequence motif of 15 nt (5′-ATGN_9_CAT-3′), identified in this region (as well as within the ETAS1 sequence) of the *M. lutreola* mtDNA, was suggested to be a sequence recognised by sequence-specific DNA-binding proteins [46]. Just upstream of this conserved motif, a region rich in inverted repetitive sequences, overlapping with tandem repeat arrays at the RS3 section was found in European mink. The latter region was located between CSB1 and CSB2, as in other mammals [38,40,42,43,74,77,134,135]. A single region with tandem repeats was found also in the control region of *L. lutra*, *Lontra Canadensis*, *C. leuconotus* and *C. chinga*, as well as many other carnivorans [40,42]. *M. lutreola* has a typical core repetitive motif 5′-ACGT-3′, found also in other mammals, including Eurasian otter, yellow-throated marten and West-South hog-nosed skunk [42,77,135]. Array of repeated sequences based on or derived from this motif were found to form a complex minisatellite in mammals, with enough palindromic sequences to fold into specific, stem-and-loop se-condary structures with minimised free energy (Figure 8), potentially playing an important role in mtDNA sequence duplications, transcription and replication [74,76,136,137]. A similar complex repetitive region, with 22 bp motif repeated 10 times, has been identified in *L. lutra* [42]. Additionally, in this species, putative secondary structures within this region have been proposed [42]. Inverted repeat sequences are present in mtDNA of variety of animals taxa, and enriched in sequences from the replication origin and D-loop [76,86].

It was proven that the mitochondrial genome size varies among animal taxa due to polymorphism in variation in the copy number of tandemly repeated sequences (VNTRs) of the control region, rather than as a result of large amplifications or deletions in the protein-coding sequences [76,138,139]. The VNTRs variation was found both among species, populations, and even within an individual (heteroplasmy) [140,141,142,143,144,145]. Intraspecific differentiation of the length of this mitogenome segment was proved in this study for European mink. Substitutions and indels found in the European mink RS3 region are in line with the trend observed in variety of mammals, including mustelids [42,134,146]. It is, however, claimed that sequence variations within this region are not informative for phylogenetic reconstruction above the species level [42].

The European mink’s control region is longer than that of *M. putorius* (881 bp), *E. lutris* (984 bp), *M. meles* (1000 bp) and *M. nivalis* (1016 bp), but shorter than that of *M. zibellina* (1089 bp), *G. gulo* (1098 bp), *M. flavigula* (1107 bp), *V. peregusna* (1108 bp), *L. lutra* (1112 bp), *Conepatus* sp. (1113 bp), *M. nigripes* (1117 bp), *M. sibirica* (1121 bp), *N. vison* (1130 bp) and *S. putorius* (1138 bp), and thus does not reflect the phylogenetic relationships between these taxa [39,48,52,62,69]. In agreement with other vertebrates, the most uniform, in terms of length, segment of the control region of the mustelids mentioned above and *M. lutreola* is the central conserved domain (average length of 326 ± 1 bp), while more diverse in size is the ETAS domain (182 ± 19 bp), and the biggest differences are found in length of the CSB domain (558 ± 71 bp) [38,39]. This fact supports the conclusion that the latter is a preferential site for insertion of short and long repeated sequences [38].

### 3.2. mtDNA Sequence Heterogeneity

The single nucleotide variants identified in this study enable the delineation of highly variable regions of the European mink mitogenome, generally corresponding to the hypervariable sites recognised in the human mtDNA control region [47]. Such sites evolve at a rate much faster than average and represent mutational hotspots [147]. Determination of the exact location of these sites in *M. lutreola* mitogenome requires further research at an interpopulation level (population mitogenomics), but the pattern of their distribution within the control region identified in the present study was similar to that found for *C. l. familiaris* [148].

The level of intraspecies sequence heterogeneity, revealed for complete mtDNA sequence of six individuals (representing one population) examined in the present study (π = 0.0326, five identified haplotypes, H_d_ = 0.933), was higher than in the case using only fragments of the mitogenome as markers of interpopulation genetic diversity of *M. lutreola*. The results of analysis of 43 individuals from three distinct populations (Northeastern European, Western European and Southeastern European [14]), based on the D-loop and 450 bp fragment of 5′-region of *cytb* show values of nucleotide diversity and haplotype diversity varying between 0 and 0.0197, and 0 and 1, respectively [16]. The number of haplotypes identified in these studies ranged from 1 to 11 in different populations. Ana-lysis based on the complete D-loop sequence in 176 individuals, from the same populations, revealed the presence of 1 to 15 haplotypes, π values ranging from 0 to 0.012, and H_d_ from 0 to 0.939 [17]. Korablev et al. [29] genotyped 11 individuals representing one population in terms of the 526 bp fragment of the control region and revealed the presence of eight haplotypes, with nucleotide diversity equal to 0.0092 and haplotype diversity to 0.95. In the same studies, additional analysis was performed, including sequences used by Michaux et al. [16], indicating π = 0.0134 and H_d_ = 0.98. Overall values of the same indicators, calculated by Cabria et al. [149] for 157 specimens from three distinct populations and based on the 614 bp mtDNA fragment including the 3′-end of the *cytb* gene and the control region, were 0.005 and 0.857, respectively. The number of haplotypes identified for these populations was 1 to 13. Thus, the potential of the complete mitogenome to resolve patterns of population genetics, and possibly also the phylogeny and phylogeographic structure of the European mink species, seems to be much greater than that of its fragments. This statement is supported by the fact that the level of genetic diversity (mea-sured by nucleotide diversity) calculated in this study is from 0.9 to 9.8 times greater than in the studies by other authors quoted above, despite examination of a smaller number of animals from a single population. However, further research is required to confirm this claim.

### 3.3. Phylogenetic Considerations

An interesting result of multi-alignment of mitochondrial PCGs’ sequences of 11 *Mustela* species is an equal degree of conservation of the cytochrome b gene and the NADH dehydrogenase genes, and a higher degree of conservation of the latter than the cytochrome c oxidase subunit (*cox1*, *cox2* and *cox3*) genes, whereas in most metazoans, *cox* and *cytb* genes are characterised by lowest interspecies variability [150]. The number of parsimony informative sites identified for carnivorans analysed in the present study is smaller than number of such sites (1739) resulting from the comparison of mitogenomes of five mustelids (*E. lutris*, *G. gulo*, *L. lutra*, *Martes melampus*, *M. meles*), performed by Ki et al. [40].

The degree of similarity between the complete sequence of the European mink mitogenome and the known mitochondrial genomes of other animals supports previous fin-dings regarding phylogenetic relationships within the class *Mammalia* L., 1758 and the taxonomic position of *M. lutreola* [24,25,26,27,28,55,60,151,152,153]. This similarity reflects the European mink belonging to taxa from successive systematic levels. The greatest similarity of mtDNA sequences was found for this species and other members of the *Mustela* L., 1758 genus. Slightly less similarity was noted with other members of the Mustelinae Fisher, 1817 subfamily; then the Mustelidae Fisher, 1817 family; the Musteloidea Fischer, 1817 superfamily; and finally, other Caniformia Kretzoi, 1943 species within the order Carnivora Bowdich, 1821 (Table 5).

The most interesting conclusion of the reconstruction and study conducted in this paper of the evolutionary relationships among members of the *Mustela* genus is confirmation of the validity of the results obtained by Davison et al. [24,60], Hosoda et al. [25], Marmi et al. [151], Flynn et al. [27], Kurose et al. [28] and Abramov et al. [153], among others, indicating significantly close evolutionary relatedness between European mink and representatives of the previously defined polecat (ferret) group. Thus, the obtained results confirm the previous findings regarding recent speciation of polecats and the European mink or horizontal gene flow between these taxa [28,60].

The results of the phylomitogenomic analysis are consistent with the results of phylogenetic reconstructions based on nuclear gene sequences as well as evolutionary infe-rence based on multi-sequence phylogenetic analyses, including both mtDNA fragments and nuclear DNA sequences [27,153]. Thus, the complete mitochondrial genome can be considered a suitable and useful marker for the reconstruction of *M. lutreola* phylogeny. It is worth noting that its usefulness in phylogenetic analyses has been proven for many other species, including mustelids [49,51,52,53,68,69,72,154,155,156,157,158,159,160,161,162,163]. Further studies should focus on an in-depth mitophylogenomic analysis of the European mink and other represen-tatives of the Mustelidae family, based on the complete mitogenome sequence announced in this paper.

## 4. Materials and Methods

### 4.1. Sample Collection and Mitochondrial DNA Extraction

The muscle material was obtained from six adult individuals of *M. lutreola* (three males and three females; offspring of different parents) kept by the European mink conservation breeding facility of the Zoological Garden in Osnabrück and the association EuroNerz e.V. (Osnabrück, Germany; 52°15′00″ N 08°04′13″ E). This institution participates in the EAZA EEP for European mink. The animals used in this study died of natural causes. Their carcasses were collected, frozen and conveyed to the Polish Society for Conservation Genetics LUTREOLA under the permit of the Regional Director for Environmental Protection in Szczecin of 31 October 2016, no. WOPN-OG.6401.272.2016.MKP. The specimens are deposited in the zoological collection of the Polish Society for Conservation Genetics LUTREOLA (Szczecin, Poland) under the accession number *M*.*l*.-M810-2016 (voucher specimen, characterized by typical phenotypic features; first sequenced and reported to the GenBank), *M*.*l*.-M598-2016, *M*.*l*.-M540-2016, *M*.*l*.-F490-2016, *M*.*l*.-F516-2016 and *M*.*l*.-F835-2016, and stored at −80 °C (Table 8).

The total genomic DNA was isolated with a method based on the modified Plasmid Mini AX kit (A&A Biotechnology, Gdańsk, Poland) protocol, with tissue lysis in LSU buffer (A&A Biotechnology, Gdańsk, Poland) and Proteinase K. First, 600 μL of LSU lysis buffer and 20 μL of Proteinase K were added to 50–100 mg of previously ground tissue. The whole mixture was mixed and incubated at 50 °C for 60 min. The samples were vortexed several times during the incubation, and after that, 600 µL of L2 alkaline lysis solution was added and carefully mixed and then left for 3 min at room temperature. Then, 600 µL of L3T neutralizing solution was added and mixed carefully. Lysates were centrifuged for 5 min at 10,000–15,000 rpm. The DNA extraction was then continued according to the Plasmid Mini AX protocol, starting at point 5 of the manufacturer’s instructions. Isolated DNA was stored frozen at −20 °C for further analysis.

### 4.2. Mitochondrial Genome Sequencing and Assembly

The total mitogenome of *M. lutreola* was obtained by the next-generation sequencing using the Illumina (NEB; Ipswich, MA, USA) sequencing by synthesis (SBS) technology [164]. A sequencing library with fragments of an insert size ranging from 200 bp to 500 bp was generated using the NEBNext^®^ DNA Library Prep Master Mix Set for Illumina (NEB; Ipswich, MA, USA), following the manufacturer’s protocol. The library preparations were sequenced on an Illumina (NEB; Ipswich, MA, USA) MiSeq PE-250 platform (MiSeq Reporter v2.6.). Two-step analysis was applied, including automatic demultiplexing of samples and generating fastq files containing raw reads. The raw reads were trimmed and filtered with Cutadapt v. 1.12 software [165] (low-quality reads (<Q30) were excluded from further analyses) and their quality was controlled by the FastQC v. 0.11.9 software (Babraham Bioinformatics, Cambridge, UK). Clean data were then assembled and mapped to the mitochondrial genome of ferret *Mustela putorius furo* (GenBank Accession No. KT693383), using the assembly algorithm of the CLC Genomics Workbench v.7.5 (Qiagen, Hilden, Germany). The same programme was used to generate, on the basis of the obtained mapping, a consensus sequence for each analysed individual (minimum coverage 3×).

### 4.3. Gene Annotation and Sequence Analysis

Protein-coding genes and RNA (tRNA and rRNA) genes were annotated by the web-based tool MITOS [166] and the GeSeq platform [167], utilising BLAT (Standalone BLAT v.35 × 1) [168] to annotate mitochondrial genes and, additionally, tRNAscan-SE v.2.0.5 [169] and ARWEN v.1.2.3 [170] for tRNA genes annotation. The exact gene boundaries were further confirmed in Geneious software v.10.0.2 (Biomatters, Auckland, New Zealand) by aligning each gene to its orthologs from available annotated Mustelidae mitochondrial genomes at the NCBI GenBank [23]. Conserved motifs within the newly recognised mtDNA were identified by comparison with carnivoran mitogenomes with known locations for these sequences, deposited in the GenBank [23]. The physical circular map of the mitochondrial genome was drawn using the online mitochondrial visualisation tool CGView [171].

DNA molecular weight was calculated in the Sequence Manipulation Suite v. 2 (http://www.bioinformatics.org/sms2/, accessed on 25 February 2021) [172]. MEGA 11 v. 11.0.10 [173] was used to analyse nucleotide composition. CpG islands, defined as 200 bp DNA regions with a G+C content greater than 50% and the ratio of observed CpG to expected CpG greater or equal to 0.6 [174], were identified using the Sequence Manipulation Suite: CpG Islands software [172] and the EMBOSS Cpgplot online tool [175]. The Genomics %G~C Content Calculator (http://www.sciencebuddies.org/science-fair-projects/references/genomics-g-c-content-calculator, accessed on 25 February 2021) was utilised to calculate the nucleotide composition. The nucleotide composition skewness, which indicates the compositional differences between the two strands (strands asymmetry), was calculated using the formula by [176]: GC-skew = (G−C)/(G+C) and AT-skew = (A−T)/(A+T), where C, G, A and T are the frequencies of the four nucleobases.

Tandem repeats were identified using the Tandem Repeat Finder v. 4.09 (matching weight, 2; mismatching penalty, 7; indel penalty, 7; match probability, 80; indel probabi-lity, 10; minimum alignment score, 50; maximum period size, 500) [177] and the Microsatellite repeats finder (http://insilico.ehu.es/mini_tools/microsatellites/, accessed on 25 February 2021; minimum length of repeated sequence, 2 bp; maximum length of repeated sequence, 10 bp; minimum number of repeats, 3; minimum length of tandem repeat, 6 bp; allowed percentage of mismatches, 0). Short inverted repeats (SIRs; a short single stranded DNA sequence repeated downstream in the reverse-complement orientation, with or without an intervening sequence [178]) were detected using the DNA Analyser v. 2.6.6, a web-based server for nucleotide sequence analysis (minimum length of palindrome, 6 bp; maximum length of palindrome, 50 bp; maximum gap between repeated regions, 20 bp; number of mismatches allowed, 0) [179]. The Palindromic Sequences Finder was used to identify the palindromes in the mtDNA of European mink (http://www.novoprolabs.com/tools/dna-palindrome, accessed on 25 February 2021; minimum and maximum length of palindromic sequence, 6 bp and 30 bp, respectively). The conserved sequences in the control region were determined by eye, based on interspecies homology search.

The frequencies of both codons and amino acids, and relative synonymous codon usage (RSCU) were calculated using MEGA 11 v. 11.0.10 [173]. RSCU was calculated using nucleotide sequence of PCGs, in which incomplete stop codons, ending in T or TA, are extended with adenine nucleotides to become complete termination codons (TAA) [31]. The ORF-Finder (http://www.ncbi.nlm.nih.gov/gorf/gorf.html, accessed on 25 February 2021) was used to predict and annotate open reading frames (ORFs) with a minimum size of 75 codons (vertebrate mitochondrial genetic code, ATG or alternative initiation codons as a start codon, ignoring nested ORFs).

The secondary structures of tRNAs and rRNAs were examined with MITOS WebServer [112]. The RNAstructure software [180], with default settings for DNA and vertebrate mitochondrial predictors, and the RNAfold web tool [181], with default settings, were used to predict potential secondary structures of the control region and the light strand replication origin (O_L_). When more than one secondary structure was possible, the one with the lowest free energy score was used.

The newly determined, complete mitochondrial DNA sequences of *M. lutreola* were deposited in the GenBank database under the following accession numbers: MW197423, MW197424, MW197425, MW197426, MW148603, MT304869 (Table 8). For analyses of mtDNA sequence composition and structure (gene order, non-coding regions, nucleotide composition, secondary structures, amino acid composition of protein-coding genes, codon usage bias) and interspecies comparisons, the mitogenome of a voucher specimen (MW148603) was used as a reference, while mitogenomic sequences of specimens MW197423, MT304869, MW197424, MW197425 and MW197426 were used for variable sites identification.

### 4.4. Analysis of Sequence Heterogeneity, Interspecies Comparison and Phylogenetic Inference

In order to detect intraspecies variable sites, obtained sequences were subjected to multiple alignment by Clustal Omega Multiple Sequence Alignment software [175], and further manually analysed using the Jalview v. 2.11.1.3 applet [182]. Separate analysis was performed for the complete mitogenomes of the six individuals included in the study, and for the 43 mtDNA sequences of *M. lutreola* deposited in the GenBank (Accession Nos. AB026105, EF689084, EF689085, EF987742, EU548039, EU548040, EU548041, EU548045, EU548046, EU548035, EU548036, EU548037, EU548038, EU548047, EU548048, EU548049, EU548050, EU548042, EU548043, EU548044, EU548051, AB051263, AF068544, AF207712, AF207713, AF207714, AY750628, AB119070, AB601576, JX982499, JX982495, JX982496, JX982497, JX982498, JX982500, JX982501, JX982502, AF207721, AF207724, AF207725, AF207720, AF207722, AF207723 [23]), which were aligned to the MW148603 reference sequence. In the case of the JX982501 sequence, the nucleotide in the first position was excluded from the analyses due to the suspicion of a possible sequencing artifact.

Genetic diversity within the mitogenome sequence of *M. lutreola* was assessed on the basis of nucleotide diversity (π), haplotype diversity (H_d_) [183] and sequence conservation index (C), using DnaSP v. 6 software [184]. Additionally, overall mean genetic distance was calculated for six aligned mitogenome sequencies using MEGA 11 v. 11.0.10 software [173]. The same programme was used to calculate the effective codon number (EN_C_) [33] and the codon bias index (CBI) [34] for protein-coding genes. The median-joining haplotype network was drawn by the NETWORK v. 10.2.0.0 software (Fluxus Technology Ltd., Colchester, UK) [185], available at http://www.fluxus-engineering.com/ (accessed on 25 February 2021).

Features of the obtained sequence of the *M. lutreola* mitogenome were further compared to the sequences of other mustelids, available in the GenBank (http://www.ncbi.nlm.nih.gov/genbank/, accessed on 25 February 2021) and in the available scientific literature. To compare mitochondrial genomes between various Caniformia species, the whole mitogenome sequences of *M. putorius* (HM106318), *M. eversmanii* (NC_028013), *M. nigripes* (NC_024942), *M. sibirica* (MN206976), *M. itatsi* (NC_034330), *M. altaica* (NC_021751), *M. nivalis* (MF459691), *M. erminea* (MW257230), *M. kathiah* (HM106320), *M. frenata* (HM106321), *N. vison* (NC_020641), *L. lutra* (NC_011358), *G. gulo* (NC_009685), *Martes foina* (NC_020643), *Meles meles* (NC_011125), *Procyon lotor* (NC_009126), *Ailurus flugens* (NC_009691), *Canis lupus* (NC_002008), *V. vulpes* (KP342452), *Ursus arctos* (NC_003427) and *Ailuropoda melanoleuca* (NC_009492) were aligned using Clustal Omega v. 1.2.4 [186]. MEGA 11 v. 11.0.10 [173] was used to estimate an overall mean genetic distance for analysed species of the *Mustela* genus. To determine the interspecies sequence data characteristics (i.e., nucleotide diversity (π), sequence conservation index (C), homozygosity (the difference between one and the value of the observed hete-rozygosity), average number of nucleotide differences (k), number of singleton and parsimony informative sites, number of indel sites), the DnaSP v. 6 programme [184] was used.

The evolutionary history of the genus *Mustela* was inferred by using the Maximum Likelihood method and the Tamura–Nei model [187]. The tree with the highest log likelihood (−99,072.16) was shown. Initial trees for the heuristic search were obtained automa-tically by applying Neighbour-Joining and BioNJ algorithms to a matrix of pairwise distances estimated using the Tamura–Nei model, and then selecting the topology with superior log likelihood value. The bootstrap method (1000 replicates) was used to test phylogeny. This analysis involved six mtDNA sequences reported in the present study, as well as whole mitogenome sequences of 10 representatives of the genus *Mustela* and five other species of the mustelid family listed in Table 5. *Tachyglossus aculeatus* was used as an outgroup species. In total, there were 16,853 positions in the final dataset. Evolutionary analyses were conducted in MEGA 11 v. 11.0.10 [173]. The same software was used to calculate pairwise genetic evolutionary distances between sequences estimated by computing the proportion of nucleotide differences between each pair of sequences.

## 5. Conclusions

Assembly of the *M. lutreola* mitochondrial genome resulted in a DNA molecule with genomic features typical for mustelid mitogenomes, i.e., conserved gene order, gene content, gene size, base composition, codon usage of PCGs and tRNA secondary structures. However, unique features were also found (mitogenome sequence heterogeneity and length variation), not only species-specific, but also with potential as markers of inter- or even intra-population genetic diversity with a high discriminant power. The complete mitogenome of European mink determined in this study enriched the number of known mitogenomes of the genus *Mustela* and helps resolve its phylogeny.

The mitochondrial genome is characterised by a faster evolution rate compared to nuclear DNA, maternal inheritance and behaving as a single, nonrecombining locus [188]. These features make mtDNA a marker useful in resolving many research problems in the field of conservation genetics, focusing on the effects of contemporary genetic structuring on long-term survival of endangered species [188]. In the case of wild populations of species threatened with extinction, the greatest application importance of complete mitogenomic sequence is related to the possibility of using it for planning and implementing effective conservation measures [14,21,189]. It is also claimed that lack of knowledge of mitochondrial genomes is a major limitation for population genetic and more reliable phylogenetic reconstructions in the Mustelidae family [36]. Complete mitogenomes can faci-litate accurate species identification and thus can be used to characterise the distribution and abundance of species, especially in case of rare or difficult-to-observe species for which faces, hair samples and environmental samples (eDNA) can be useful DNA sources [190,191,192,193,194]. Mitogenomic markers can also elucidate the population genetic structure [42]. In addition, complete mitogenomes allow comparative mitogenomic studies [40,195]. The possibility to use the complete mitogenome sequence to define the taxonomic rank of *M. lutreola* subspecies (molecular systematics) is promising and must be further investigated, as it has been proven useful for other endangered species [21,196]. mtDNA can be also used to identify genetically defined conservation units at an intraspecies level, e.g., evolutionary significant units (ESUs), management units and distinct population segments (DPSs) [16,17,197,198,199,200].

Conservation genetics issues of key importance for European mink are the determination of an optimal scenario for restoration programs, captive breeding genetics and identification and assessment of hybridisation and introgression events [8,14,16,17,201]. Mitogenome sequence data might address these issues and provide genetic information supporting protection and conservation measures, important in the preservation of genetic resources of *M. lutreola*. However, serious drawbacks of mtDNA in population genetics should be noted, including its gene-specific, species-specific and lineage-specific evolution; restriction of its use to exploring phylogenetic events in maternal lineage; and its limited use in investigating recent loss of genetic variation [188]. Consequently, it is claimed that mtDNA is a useful auxiliary genetic marker to nuclear DNA, and it is thus recommended to apply combined analyses of nuclear and mitochondrial markers in conservation genetics [188].

The next step in mitogenomic research on European mink should focus on sequen-cing and analysing complete mitochondrial genomes of a representative number of individuals from the preserved wild populations and of captive stock of conservation breeding programmes. The usefulness of the complete mitogenome sequence in tracking natural hybridisation events between *M. lutreola* and polecats also requires further investigation.

## Figures and Tables

**Table 1:** Features of the mitochondrial genome of *Mustela lutreola*.

Gene	Position		Size (bp)	Amino Acids Count ^1^	Codon		Anticodon	Intergenic Nucleotide (bp) ^2^	Strand ^3^
	From	To			Start	Stop			
*tRNA^Phe^*	1	69	69				GAA	0	H
*rrnS*	70	1028	959					0	H
*tRNA^Val^*	1029	1096	68				TAC	−2	H
*rrnL*	1095	2665	1571					0	H
*tRNA^Leu(UUA)^*	2666	2740	75				TAA	2	H
*nad1*	2743	3698	956	318	ATG	TA ^5^		0	H
*tRNA^Ile^*	3699	3767	69				GAT	−3	H
*tRNA^Gln^*	3765	3838	74				TTG	1	L
*tRNA^Met^*	3840	3908	69				CAT	0	H
*nad2*	3909	4950	1042	347	ATT ^4^	T ^5^		0	H
*tRNA^Trp^*	4951	5017	67				TCA	10	H
*tRNA^Ala^*	5028	5096	69				TGC	1	L
*tRNA^Asn^*	5098	5170	73				GTT	0	L
O_L_	5171	5205	35					−3	L
*tRNA^Cys^*	5203	5269	67				GCA	1	L
*tRNA^Tyr^*	5271	5338	68				GTA	1	L
*cox1*	5340	6884	1545	514	ATG	TAA		−3	H
*tRNA^Ser(UCA)^*	6882	6950	69				TGA	5	L
*tRNA^Asp^*	6956	7022	67				GTC	0	H
*cox2*	7023	7706	684	227	ATG	TAA		3	H
*tRNA^Lys^*	7710	7776	67				TTT	1	H
*atp8*	7778	7981	204	67	ATG	TAA		−43	H
*atp6*	7939	8619	681	226	ATG	TAA		−1	H
*cox3*	8619	9402	784	261	ATG	T ^5^		0	H
*tRNA^Gly^*	9403	9471	69				TCC	0	H
*nad3*	9472	9818	347	115	ATA ^4^	TA ^5^		0	H
*tRNA^Arg^*	9819	9886	68				TCG	0	H
*nad4l*	9887	10,183	297	98	ATG	TAA		−7	H
*nad4*	10,177	11,554	1378	459	ATG	T ^5^		0	H
*tRNA^His^*	11,555	11,623	69				GTG	0	H
*tRNA^Ser(AGC)^*	11,624	11,685	62				GCT	0	H
*tRNA^Leu(CUA)^*	11,686	11,755	70				TAG	0	H
*nad5*	11,756	13,576	1821	606	ATT ^4^	TAA		−17	H
*nad6*	13,560	14,090	531	176	ATG	TAA		3	L
*tRNA^Glu^*	14,094	14,162	69				TTC	4	L
*Cytb*	14,167	15,306	1140	379	ATG	AGA		0	H
*tRNA^Thr^*	15,307	15,374	68				TGT	−1	H
*tRNA^Pro^*	15,374	15,439	66				TGG	0	L
CR	15,440	16,504	1065					0	H

^1^ Termination codons excluded, ^2^ numbers correspond to the nucleotides separating adjacent genes; negative numbers indicate overlapping nucleotides, ^3^ letter indicates the gene encoding chain (H—heavy, L—light), ^4^ alternative start codons of the vertebrate mitochondrial code, ^5^ incomplete stop codons likely extended upon subsequent polyadenylation [31]; O_L_—light strand replication origin, CR—control region.

**Table 2:** Codon usage table of 13 mitochondrial protein-coding genes of European mink (including presumed polyadenylated incomplete termination codons [31]).

Amino Acid	Codon	%	Amino Acid	Codon	%	Amino Acid	Codon	%	Amino Acid	Codon	%
F	TTT	2.65	S	TCT	1.42	A	GCA	2.39	D	GAT	0.58
	TTC	3.44		TCC	1.79		GCG	0.18		GAC	1.18
L	TTA	3.31		TCA	2.63	Y	TAT	1.39	E	GAA	2.08
	TTG	0.58		TCG	0.13		TAC	2.08		GAG	0.47
	CTT	1.89		AGT	0.47	*	TAA	0.32	C	TGT	0.29
	CTC	1.92		AGC	1.05		TAG	0.00		TGC	0.39
	CTA	7.20	P	CCT	1.16		AGA	0.03	W	TGA	2.29
	CTG	1.10		CCC	1.76		AGG	0.00		TGG	0.47
I	ATT	3.99		CCA	2.05	H	CAT	0.60	R	CGT	0.16
	ATC	4.65		CCG	0.13		CAC	1.92		CGC	0.42
M	ATA	5.28	T	ACT	2.08	Q	CAA	2.10		CGA	1.00
	ATG	1.18		ACC	2.15		CAG	0.21		CGG	0.11
V	GTT	0.84		ACA	3.73	N	AAT	1.52	G	GGT	0.79
	GTC	1.10		ACG	0.42		AAC	2.44		GGC	1.26
	GTA	2.42	A	GCT	1.47	K	AAA	2.44		GGA	2.68
	GTG	0.39		GCC	2.68		AAG	0.21		GGG	0.92

* Termination codon.

**Table 3:** Codon bias indicators revealed for protein-coding genes found in the mtDNA of European mink.

Gene	EN_C_ ^1^	CBI ^2^	G+C3s ^3^
*nad1*	43.567	0.464	0.379
*nad2*	31.270	0.597	0.313
*cox1*	46.359	0.321	0.338
*cox2*	45.559	0.417	0.326
*atp8*	41.945	0.647	0.175
*atp6*	39.575	0.454	0.382
*cox3*	39.337	0.482	0.383
*nad3*	44.332	0.561	0.288
*nad4l*	39.698	0.585	0.400
*nad4*	43.570	0.454	0.393
*nad5*	42.770	0.429	0.409
*nad6*	39.644	0.565	0.243
*Cytb*	43.446	0.477	0.454
overall of PCGs	44.341	0.367	0.404

^1^ EN_C_—effective codon number, ^2^ CBI—codon bias index, ^3^ G+C3s—frequency of G−C nucleotides at the third codon position, PCG—protein-coding gene.

**Table 4:** Characteristics of the secondary structure of the mitochondrial tRNAs in European mink.

Feature		tRNA^Phe^	tRNA^Val^	tRNA^Leu(UUA)^	tRNA^Ile^	tRNA^Gln^	tRNA^Met^	tRNA^Trp^	tRNA^Ala^	tRNA^Asn^	tRNA^Cys^	tRNA^Tyr^	tRNA^Ser(UCA)^	tRNA^Asp^	tRNA^Lys^	tRNA^Gly^	tRNA^Arg^	tRNA^His^	tRNA^Ser(AGC)^	tRNA^Leu(CUA)^	tRNA^Glu^	tRNA^Thr^	tRNA^Pro^
**Unmatched Base Pairs**	U-G pairing ^1^	0	0	1	0	1	0	1	4	1	2	2	4	1	0	2	0	0	0	0	3	0	5
	Mismatching	A-A					A-G, U-U, U-U	A-C						U-U	A-A, A-C							U-U	
**Stem (Arm) Size (bp)**	Acceptor	7	7	7	7	7	7	7	7	7	7	7	7	7	7	7	7	7	7	7	7	7	7
	TΨC	5	4	5	5	3	5	5	5	5	4	5	5	5	5	5	5	5	5	4	5	5	5
	Anticodon	5	5	4	5	4	5	5	5	5	5	5	5	5	5	5	5	5	4	5	5	5	5
	DHU	4	4	4	3	4	4	4	4	3	4	3	3	4	5	4	4	4	0	4	4	3	4

^1^ Number of cases indicated.

**Table 5:** Comparison of the basic features of the mitochondrial genomes of *Mustela lutreola* and other members of the Caniformia suborder.

Species	Family	Size (bp)	GC%	AT-Skew	GC-Skew	GenBank Accession No.	Percent Identity	Reference ^1^
*Mustela lutreola*	Mustelidae	16,504	39.9	0.093	−0.308	MW148603	100.00%	this study
*Mustela putorius*		16,523	39.8	0.091	−0.308	HM106318	99.04%	d.s.
*Mustela eversmanni*		16,463	40.0	0.091	−0.305	NC_028013	98.72%	d.s.
*Mustela nigripes*		16,556	39.9	0.095	−0.310	NC_024942	98.31%	[48]
*Mustela sibirica*		16,558	39.8	0.092	−0.303	MN206976	96.99%	[49]
*Mustela itatsi*		16,027	39.5	0.091	−0.304	NC_034330	95.24%	[50]
*Mustela altaica*		16,521	39.7	0.086	−0.301	NC_021751	91.96%	[51]
*Mustela nivalis*		16,502	40.0	0.087	−0.296	MF459691	91.50%	[52]
*Mustela ermine*		16,500	40.1	0.112	−0.324	MW257230	91.32%	d.s.
*Mustela kathiah*		16,552	38.9	0.088	−0.301	HM106320	89.29%	d.s.
*Mustela frenata*		16,543	39.2	0.096	−0.314	HM106321	88.59%	d.s.
*Neogale vison*		16,552	38.6	0.093	−0.312	NC_020641	88.50%	[53]
*Lutra lutra*		16,536	42.0	0.111	−0.310	NC_011358	86.07%	d.s.
*Gulo gulo*		16,541	41.1	0.093	−0.304	NC_009685	86.02%	[54]
*Martes foina*		16,530	41.9	0.105	−0.310	NC_020643	86.00%	d.s.
*Meles meles*		16.442	38.9	0.091	−0.316	NC_011125	85.46%	[54]
*Procyon lotor*	Procyonidae	16,623	39.6	0.095	−0.288	NC_009126	82.33%	d.s.
*Ailurus flugens*	Ailuridae	16,374	37.5	0.054	−0.292	NC_009691	82.30%	[55]
*Canis lupus*	Canidae	16,727	39.7	0.048	−0.287	NC_002008	81.13%	[56]
*Vulpes vulpes*		16,723	40.7	0.058	−0.276	KP342452	79.47%	[57]
*Ursus arctos*	Ursidae	17,020	41.3	0.053	−0.239	NC_003427	80.81%	[58]
*Ailuropoda melanoleuca*		16,805	38.8	0.038	−0.227	NC_009492	80.73%	[59]

^1^ d.s.—direct submission.

**Table 6:** Pairwise genetic distance ^1^ matrix of mitogenome sequences among specimens of the *Mustela* genus ^2^ (the number of base differences per site from between sequences are shown; all ambiguous positions were removed for each sequence pair).

Species	*M.er.*	*M.s.*	*M.ng.*	*M.k.*	*M.f.*	*M.p.*	*M.a.*	*M.nv.*	*M.ev.*	*M.i.*	*M.l.*
*M.er.*	-										
*M.s.*	0.5967	-									
*M.ng.*	0.5989	0.6459	-								
*M.k.*	0.7195	0.6988	0.7102	-							
*M.f.*	0.6944	0.6602	0.7204	0.6936	-						
*M.p.*	0.6266	0.6346	0.5102	0.7240	0.3992	-					
*M.a.*	0.3230	0.7213	0.6900	0.7188	0.6077	0.7205	-				
*M.nv.*	0.7311	0.7194	0.7433	0.7200	0.7295	0.7406	0.7319	-			
*M.ev.*	0.6372	0.6250	0.4969	0.7311	0.6843	0.4787	0.7364	0.7363	-		
*M.i.*	0.6622	0.6429	0.6100	0.7184	0.6930	0.5848	0.7205	0.7341	0.5674	-	
*M.l.*	0.6346	0.6238	**0.4975**	0.7321	0.6845	**0.4790**	0.7361	0.7349	**0.0241**	0.5660	-

^1^ Evolutionary distances between sequences estimated by computing the proportion of nucleotide differences between each pair of sequences, ^2^ *M.er.* = *M. ermine*, *M.s.* = *M. sibirica*, *M.ng.* = *M. nigripes*, *M.k.* = *M. kathiah*, *M.f.* = *M. frenata*, *M.p.* = *M. putorius*, *M.a.* = *M. altaica*, *M.nv.* = *M. nivalis*, *M.ev.* = *M. eversmannii*, *M.i.* = *M. itatsi*, *M.l.* = *M. lutreola*; the smallest values of genetic distance for European mink are marked in bold.

**Table 7:** Conserved regions identified in the control region of the European mink, European polecat, steppe polecat, black-footed ferret, Siberian weasel, Japanese weasel, mountain weasel, least weasel, stoat, yellow-bellied weasels and long-tailed weasel mitogenomes.

Position (bp) ^1^	C ^2^	Hom. ^3^	*p*-Value	Region
15,348–15,514	0.861	0.957	0.0009	ETAS domain (5′-end)
15,677–15,825	0.865	0.951	0.0011	CD domain (E-box, C-box, D-box)
15,830–16,038	0.899	0.967	0	CD domain (B-box, 3′-end) + CSB domain (5′-end, CSB1)
16,421–16,476	0.862	0.962	0.0423	CSB domain (LSP, HSP, 3′-end)

^1^ Positions given relative to the European mink reference sequence (GenBank Accession No. MW148603), ^2^ C—sequence conservation index, ^3^ Hom.—homozygosity.

**Table 8:** Characteristics of samples used in the study.

Sample Name	Sex	Age ^1^	Date of Death	GenBank Accession No.
*M*.*l*.-M540-2016	♂	62	June 2013	MT304869
*M*.*l*.-M598-2016	♂	61	May 2014	MW197423
***M*** **.*l*.-M810-2016**	♂	**6**	**November 2012**	**MW148603**
*M*.*l*.-F490-2016	♀	67	November 2012	MW197424
*M*.*l*.-F516-2016	♀	73	May 2014	MW197425
*M*.*l*.-F835-2016	♀	20	February 2014	MW197426

^1^ In months; reference genome in bold.

## Data Availability

The data presented in this study are available in Supplementary Materials.

## Supplementary Materials

The following supporting information can be downloaded at: https://www.mdpi.com/article/10.3390/genes13010125/s1, Table S1: Percent base composition and nucleotide skews for coding and non-coding regions found in the mtDNA of European mink, Table S2: Tandem repeats detected in the mitochondrial genome of *Mustela lutreola*, Table S3: Inverted repeats in the mitochondrial genome of European mink, Table S4: Palindromic sequences found in the European mink mitogenome, Table S5: Characteristics of the open reading frames (ORFs) identified in the *Mustela lutreola* mitogenome, Table S6. Characteristics of variable sites identified in the European mink mitogenome, Figure S1: Graphical analysis of the European mink mitogenome sequence for the presence and distribution of CpG dinucleotides (upper graph—distribution of observed/expected ratios of CpG dinucleotides, middle graph—distribution of C+G nucleotides, lower graph—identification of putative CpG island), Figure S2: Distribution of repetitive and palindromic sequences in the European mink mitochondrial genome, Figure S3: Predicted secondary structures of 22 mitochondrial tRNAs in European mink, Figure S4: Predicted secondary structure of the mitochondrial ribosomal RNA of the large ribosomal subunit in *Mustela lutreola* (dashes indicate Watson-Crick base pairing; red asterisks indicate non-canonical G-U pairs; the 5′-end is marked with a green circle and the 3′-end with a red circle), Figure S5: Predicted secondary structure of the mitochondrial ribosomal RNA of the small ribosomal subunit in *Mustela lutreola* (dashes indicate Watson-Crick base pairing; red asterisks indicate non-canonical G-U pairs; the 5′-end is marked with a green circle and the 3′-end with a red circle), Figure S6: Multiple alignment for the 43 mtDNA sequences of *Mustela lutreola* deposited in the GenBank [23] aligned to the MW148603 reference sequence, reported in this paper.
