# Supplementary material for: Characterisation of the Complete Mitochondrial Genome of Critically Endangered Mustela lutreola (Carnivora: Mustelidae) and Its Phylogenetic and Conservation Implications
Source: Genes (Basel). 2022 Jan 10;13(1):125. doi: 10.3390/genes13010125 (PMC8774856; doi:10.3390/genes13010125)
Supplement: Supplementary file 1 [file genes-13-00125-s001.zip › genes-1516746-supplementary.pdf]

Skorupski J. 2021. **Characterisation of the complete mitochondrial genome of critically endangered Mustela lutreola (Carnivora: Mustelidae) and its phylogenetic and conservation implications** – Supplementary Materials

Table S1. Percent base composition and nucleotide skews for coding and non-coding regions found in the mtDNA of European mink.

Mitogenome section	A (%)	T (%)	G (%)	C (%)	A+T (%)	AT-skew	GC-skew
nad1	30.44	28.14	12.03	29.39	58.58	0.039	-0.419
nad2	37.62	26.10	8.83	27.45	63.72	0.181	-0.513
cox1	27.44	31.07	17.54	23.95	58.51	-0.062	-0.154
cox2	32.75	28.07	13.89	25.29	60.82	0.077	-0.291
atp8	40.69	29.90	6.86	22.55	70.59	0.153	-0.533
atp6	30.69	28.49	11.45	29.37	59.18	0.037	-0.439
cox3	27.17	28.32	15.69	28.83	55.48	-0.021	-0.295
nad3	33.14	28.24	12.39	26.22	61.38	0.080	-0.358
nad4l	27.61	33.00	12.79	26.60	60.61	-0.089	-0.350
nad4	32.73	27.07	11.68	28.52	59.80	0.095	-0.419
nad5	33.33	26.91	11.64	28.12	60.24	0.107	-0.414
nad6 [1]	21.47	42.18	26.74	9.60	63.65	-0.325	0.472
Cytb	29.12	27.81	13.07	30.00	56.93	0.023	-0.393
**overall of protein-coding genes** [2]	**31.00**	**28.83**	**13.44**	**26.73**	**59.83**	**0.036**	**-0.331**
1st in codon [3]	31.66	23.17	21.44	23.73	54.83	0.155	-0.051
2nd in codon [3]	19.55	41.96	12.32	26.17	61.51	-0.364	-0.360
3rd in codon [3]	41.93	21.31	6.52	30.24	63.24	0.326	-0.645
rrnS	37.54	23.36	17.52	21.58	60.90	0.233	-0.104
rrnL	36.41	24.89	17.31	21.39	61.30	0.188	-0.105
**overall of rRNA genes**	**36.84**	**24.31**	**17.39**	**21.46**	**61.15**	**0.205**	**-0.105**
$tRNA^{Phe}$	43.48	21.74	17.39	17.39	65.22	0.333	0.000
$tRNA^{Val}$	35.29	30.88	14.71	19.12	66.18	0.067	-0.130
$tRNA^{Leu(UUA)}$	30.67	26.67	18.67	24.00	57.33	0.070	-0.125
$tRNA^{Ile}$	37.68	31.88	15.94	14.49	69.57	0.083	0.048
$tRNA^{Gln}$ [1]	24.32	41.89	24.32	9.46	66.22	-0.265	0.440
$tRNA^{Met}$	26.09	27.54	18.84	27.54	53.62	-0.027	-0.188
$tRNA^{Trp}$	35.82	22.39	19.40	22.39	58.21	0.231	-0.071
$tRNA^{Ala}$ [1]	28.99	34.78	21.74	14.49	63.77	-0.091	0.200
$tRNA^{Asn}$ [1]	26.03	31.51	27.40	15.07	57.53	-0.095	0.290
$tRNA^{Cys}$ [1]	28.36	25.37	23.88	22.39	53.73	0.056	0.032
$tRNA^{Tyr}$ [1]	32.35	29.41	23.53	14.71	61.76	0.048	0.231
$tRNA^{Ser(UCA)}$ [1]	34.48	24.64	15.94	24.64	59.42	0.171	-0.214
$tRNA^{Asp}$	35.82	35.82	16.42	11.94	71.64	0.000	0.158
$tRNA^{Lys}$	37.31	26.87	16.42	19.40	64.18	0.163	-0.083
$tRNA^{Gly}$	34.78	30.43	14.49	20.29	65.22	0.067	-0.167
$tRNA^{Arg}$	42.65	35.29	8.82	13.24	77.94	0.094	-0.200
$tRNA^{His}$	43.48	34.78	8.70	13.04	78.26	0.111	-0.200

tRNA[Ser(AGC)]	33.87	22.58	16.13	27.42	56.45	0.200	-0.259
tRNA[Leu(CUA)]	41.43	27.14	17.14	14.29	68.57	0.208	0.091
tRNA[Glu] [1]	30.43	37.68	18.84	13.04	68.12	-0.106	0.182
tRNA[Thr]	32.35	33.82	14.71	19.12	66.18	-0.022	-0.130
tRNA[Pro] [1]	34.85	18.18	15.15	31.82	53.03	0.314	-0.355
**overall of tRNA genes**	**34.06**	**29.70**	**17.72**	**18.52**	**63.76**	**0.068**	**-0.022**
control region	29.23	26.41	15.23	29.14	55.64	0.051	-0.314
O_L [1]	8.57	31.43	25.71	34.29	40.00	-0.571	-0.143
intergenic spacers	35.48	38.71	3.23	22.58	74.19	-0.043	-0.750
**overall of non-coding regions**	**28.76**	**26.90**	**15.22**	**29.12**	**55.66**	**0.033**	**-0.313**
**overall of the mitogenome**	**32.82**	**27.24**	**13.83**	**26.11**	**60.06**	**0.093**	**-0.308**

[1] L-strand, [2] excluding presumed polyadenylated incomplete termination codons [31], [3] including presumed polyadenylated incomplete termination codons [31]; genes and non-coding/regulatory regions are color-coded: light blue – tRNA genes, light green – rRNA genes, light yellow – protein-coding genes, light red – non-coding/regulatory regions (O_L – light strand replication origin).

Table S2. Tandem repeats detected in the mitochondrial genome of *Mustela lutreola*.

Position (bp)	Motif length (bp)	Repeats	Sequence (5′-3′)
681	2	3	TATATA
743	2	3	AAAAAA
808	2	3	ACACAC
873	2	3	AGAGAG
906	2	3	CACACA
935	2	3	ACACAC
1,076	2	3	ATATAT
1,571	2	3	AGAGAG
1,684	2	3	ACACAC
2,639	2	3	CCCCCC
3,243	2	3	CACACA
3,432	3	3	CATCATCAT
3,612	2	3	AAAAAA
3,684	2	3	CCCCCC
4,043	2	3	AAAAAA
4,309	2	3	AAAAAA
4,318	2	3	CCCCCC
4,357	2	4	ACACACAC
4,462	2	3	CACACA
4,721	2	3	AAAAAA
4,868	2	3	AAAAAA
5,189	2	3	AAAAAA
5,726	2	3	CCCCCC
5,919	3	3	CTTCTTCTT
6,012	2	3	GGGGGG
6,128	2	3	AAAAAA
6,211	2	3	ATATAT
6,238	2	3	ACACAC
6,389	2	3	GGGGGG
6,846	2	3	CACACA

7,763	2	3	TCTCTC
7,838	2	3	TTTTTT
8,223	2	3	CACACA
8,631	2	3	ACACAC
8,778	2	3	ATATAT
9,207	2	3	TTTTTT
9,461	2	3	AGAGAG
9,681	3	3	ACTACTACT
9,897	2	3	TATATA
9,970	2	3	CTCTCT
10,450	2	3	AAAAAA
11,101	2	3	TATATA
11,726	2	3	AAAAAA
11,830	2	3	TATATA
12,222	3	3	GATGATGAT
12,273	2	3	TATATA
12,650	2	3	AAAAAA
13,431	2	3	AAAAAA
13,906	2	3	CCCCCC
13,939	2	3	AAAAAA
14,485	2	3	TATATA
14,628	2	3	ATATAT
14,954	2	3	ACACAC
15,455	2	3	CTCTCT
15,465	2	3	ATATAT
15,508	2	4	TTTTTTTTT
15,518	2	3	CCCCCC
15,568	2	3	TATATA
15,823	2	3	CACACA
15,860	2	3	TTTTTT
15,867	2	3	GGGGGG
16,008	2	3	ACACAC
16,030	10	16	GCACACGTACGCACACGTACGCACACGTACGCACACGT ACGCACACGTACGCACACGTACGCACACGTACGCACAC GTACGCACACGTACGCACACGTACGCACACGTACGCAC ACGTACGCACACGTACGCACACGTACGCACACGTACGC ACACGTAC
16,199	8	4	CACACGTACACACGTACACACGTACACACGTA
16,030	10	22.1	GCACACGTACGCACACGTACGCACACGTACGCACACGT ACGCACACGTACGCACACGTACGCACACGTACGCACAC GTACGCACACGTACGCACACGTACGCACACGTACGCAC ACGTACGCACACGTACGCACACGTACGCACACGTACGC ACACGTACACACGTACGCACACGTACACACGTACACAC GTACACACGTACGCACACGGTAC
16,284	2	3	CCCCCC
16,293	2	3	CCCCCC
16,371	2	3	ATATAT

Table S3. Inverted repeats in the mitochondrial genome of European mink.

Length of repeat (bp)	Length of spacer (bp)	Position (bp)	Sequence (5′-3′)[1]
6	1	394	GTTAAA—A—TTTAAC
6	17	402	TTAACA—AGGCTGTAAAAAGCCAC—TGTTAA
6	5	448	AAGTAA—CTTTA—TTACTT
6	10	532	ATAATT—ATCACAACAA—AATTAT
6	4	821	AGTTTT—TATG—AAAACT
8	7	821	AGTTTTTA—TGAAAAC—TAAAAACT
6	13	834	ACTAAA—AACTAAAGGTGGA—TTTAGT
6	6	841	ACTAAA—GGTGGA—TTTAGT
7	9	863	AATTAAG—AATAGAGAG—CTTAATT
9	6	1,527	TCTAAAAAG—GTACAG—CTTTTTAGA
6	4	1,701	TCTATT—TTAT—AATAGA
6	15	1,774	GTTAAC—GAATACCCACTGATA—GTTAAC
6	13	1,940	CCTCCA—GCATATCTAGTAT—TGGAGG
6	8	2,112	TTCCCG—TGAAGAGG—CGGGAA
7	11	2,254	CTCGGAG—AATAAAATAAC—CTCCGAG
6	9	2,322	TTGATC—CAATAATTT—GATCAA
6	17	2,376	CCTATT—TGAGAGTCCATATCAAC—AATAGG
8	17	2,541	TACTTCTC—CCAGTACGAAAGGACAA—GAGAAGTA
7	4	2,647	AACCCTA—GAAA—TAGGGTT
8	10	2,858	TTGTAGGA—CCCTACGGCC—TCCTACAA
6	1	3,139	CTACGG—G—CCGTAG
6	1	3,645	TATATG—A—CATATA
6	18	3,887	GGTTTA—TCCCCTTCCCGTACTAAT—TAAACC
6	16	3,941	TACTAT—TATCTCAGGGACTATC—ATAGTA
6	11	4,882	TATTAC—CCCCATTAATT—GTAATA
7	3	4,994	TAAGTGA—CAC—TCACTTA
11	13	5,171	CTTCTCCCGCC—GCGAAGGAAAAAA—GGCGGGAGAAG
6	18	5,201	AGAAGC—CCCGGCAGGGTTGAAGCT—GCTTCT
7	3	5,231	TTGAATT—TGC—AATTCAA
6	8	5,355	TGATTA—TTCTCCAC—TAATCA
6	7	6,072	ATCCTG—ATTCTCC—CAGGAT
6	12	6,204	GCCCAC—CATATATTTACT—GTGGGC
6	15	6,554	AAATGA—TGTATGAGCAAAAAT—TCATTT
6	13	6,720	TCATTA—ACGGCAGTCATAC—TAATGA
6	7	6,791	AGTTGA—ATTAACC—TCAACT
8	1	6,977	TGACTTTG—T—CAAAGTCA
6	13	7,103	ACTAAT—AATTGTATTTCTA—ATTAGT
6	0	7,424	ACTTCT—AGAAGT
6	16	7,460	AGAAAT—AACAATTCGTATACTA—ATTTCT
6	2	7,945	GAAAAT—CT—ATTTTC
6	0	8,273	CAGTAA—TTACTG
6	15	8,569	GCTTAC—GTCTTTACCCTACTA—GTAAGC
6	4	9,594	TGATCC—CTTA—GGATCA
7	6	9,861	ATTGTAA—CTCATA—TTACAAT
6	15	9,885	AAATGT—CCGTAGTATATATCA—ACATTT

6	14	9,996	TGTTAT—CCCTCTTCGTCATA—ATAACA
6	10	10,135	CACATA—CGGGACAGAT—TATGTG
6	7	10,188	TATTAT—CCCAACC—ATAATA
8	15	10,248	AATTAATA—CAACAGCTCACAGCA—TATTAATT
6	20	10,387	TGGCTT—CTTCCCCTGATGCTCATAGC—AAGCCA
7	19	10,758	ACTATGA—CTAGCATGCATAATAGCAT—TCATAGT
6	7	10,972	TTATGA—GGGATAA—TCATAA
6	12	11,108	GAGCAA—CAGCCCTAATAA—TTGCTC
9	2	11,201	TAATCCTCG—CA—CGAGGATTA
7	7	11,525	ACCCTAA—AATTATC—TTAGGGT
6	1	11,548	ATTTAC—T—GTAAAT
7	3	11,578	TTAGATT—GTG—AATCTAA
7	10	11,596	AATAAAA—GCTCAAACCT—TTTTATT
6	8	11,611	CTTTTT—ATTTACCG—AAAAAG
6	9	11,637	GCATGA—ACTGCTAAC—TCATGC
6	6	11,660	CCGTGT—ATAAAA—ACACGG
9	6	12,193	AGTAGGAAT—TATATC—ATTCCTACT
6	16	12,204	TATCAT—TCCTACTTATCGGATG—ATGATA
6	18	12,899	GGAATA—CCCTTCTTAACGGGATTT—TATTCC
6	12	13,073	ATTATT—ACAATCAACGAG—AATAAT
6	10	13,191	AGATAA—CTATGCCTTA—TTATCT
6	11	13,410	TTCTAG—ATATAATTTGA—CTAGAA
8	4	14,021	AGGGTTTT—GAAG—AAAACCCT
6	12	14,024	GTTTTG—AAGAAAACCCTA—CAAAAC
6	18	14,069	GAATAA—ATACAATGTATGTCATCA—TTATTC
7	10	14,279	GAATCTG—CCTAATTATT—CAGATTC
6	7	14,875	CTAATA—CTAACAC—TATTAG
6	5	14,879	TACTAA—CACTA—TTAGTA
6	12	15,027	ATTAGG—AGGGGTACTAGC—CCTAAT
6	7	15,329	TTTGGT—CTTGTAA—ACCAAA
6	7	15,399	ATCAGC—ACCCAAA—GCTGAT
6	17	15,564	CATGTA—TATATTATGGTTGATTT—TACATG
6	16	15,877	ACTTGG—TATCACTCAGCTATGG—CCAAGT
6	4	16,035	CGTACG—CACA—CGTACG
6	4	16,045	CGTACG—CACA—CGTACG
6	4	16,055	CGTACG—CACA—CGTACG
6	4	16,065	CGTACG—CACA—CGTACG
6	4	16,075	CGTACG—CACA—CGTACG
6	4	16,085	CGTACG—CACA—CGTACG
6	4	16,095	CGTACG—CACA—CGTACG
6	4	16,105	CGTACG—CACA—CGTACG
6	4	16,115	CGTACG—CACA—CGTACG
6	4	16,125	CGTACG—CACA—CGTACG
6	4	16,135	CGTACG—CACA—CGTACG
6	4	16,145	CGTACG—CACA—CGTACG
6	4	16,155	CGTACG—CACA—CGTACG
6	4	16,165	CGTACG—CACA—CGTACG
6	12	16,175	CGTACG—CACACGTACACA—CGTACG
6	15	16,218	ACGTAC—ACACGTACGCACACG—GTACGT
6	7	16,226	ACGTAC—GCACACG—GTACGT

6	4	16,309	AAGTAT─ACAA─ATACTT
6	6	16,374	TATTAG─AAGTCA─CTAATA

[1] base motif – intervening sequence – repeated inverted sequence, complementary to a base motif

Table S4. Palindromic sequences found in the European mink mitogenome.

Position	Sequence (5'-3')	Length (bp)
287	TGTTAACA	8
288	GTTAAC	6
305	AAATTT	6
321	ACCGCGGT	8
322	CCGCGG	6
398	AAATTT	6
532	ATAATTAT	8
533	TAATTA	6
613	CCTCTAGAGG	10
614	CTCTAGAG	8
615	TCTAGA	6
636	ATCGAT	6
681	TATATA	6
732	TAATATTA	8
733	AATATT	6
944	AATATT	6
1,040	TTAATTAA	8
1,041	TAATTA	6
1,076	ATATAT	6
1,080	ATTAAT	6
1,201	CTATAG	6
1,289	AGCTAGCT	8
1,290	GCTAGC	6
1,291	CTAGCTAG	8
1,292	TAGCTA	6
1,314	AGATCT	6
1,318	CTTAAG	6
1,477	TTTAAA	6
1,479	TAAATTTA	8
1,480	AAATTT	6
1,511	TAAATTTA	8
1,512	AAATTT	6
1,515	TTTAAA	6
1,598	TAGGCCTA	8
1,599	AGGCCT	6
1,707	TTATAA	6

1,760	ATAAGCTTAT	10
1,761	TAAGCTTA	8
1,762	AAGCTT	6
1,766	TTATAA	6
1,774	GTTAAC	6
1,795	GTTAAC	6
1,827	AATATT	6
1,843	CAATTG	6
1,863	GCATGC	6
1,983	ACTAGT	6
1,993	CGGCCG	6
1,996	CCGCGG	6
2,061	TGGCCA	6
2,205	AGGCCT	6
2,317	ATTAAT	6
2,335	TTGATCAA	8
2,336	TGATCA	6
2,355	CCCTAGGG	8
2,356	CCTAGG	6
2,453	ATTAAT	6
2,533	ATTATAAT	8
2,534	TTATAA	6
2,537	TAATTA	6
2,747	TTATAA	6
2,750	TAATTA	6
2,885	CAATTG	6
3,044	GGATCC	6
3,096	ATCCGGAT	8
3,097	TCCGGA	6
3,284	TAGCTA	6
3,359	AACTAGTT	8
3,360	ACTAGT	6
3,511	ATTAAT	6
3,557	GAATTC	6
3,614	AAAATTTT	8
3,615	AAATTT	6
3,668	TTATAA	6
3,763	TTCTAGAA	8
3,764	TCTAGA	6
3,820	AAATTT	6
3,852	TAATTA	6
3,865	GGGCCC	6

3,907	TAATTA	6
3,964	TAGTACTA	8
3,965	AGTACT	6
4,063	CAATTG	6
4,080	AAATATTT	8
4,081	AATATT	6
4,276	CATTAATG	8
4,277	ATTAAT	6
4,365	TTATTAATAA	10
4,366	TATTAATA	8
4,367	ATTAAT	6
4,375	CTATAG	6
4,412	AGGCCT	6
4,533	ATTATAAT	8
4,534	TTATAA	6
4,732	TAATTA	6
4,738	CTGCAG	6
4,745	AATATT	6
4,892	ATTAAT	6
5,078	TTAATTAA	8
5,079	TAATTA	6
5,132	GTTAAC	6
5,237	TTGCAA	6
5,346	ATTAAT	6
5,350	ATCGAT	6
5,498	TTATAA	6
5,639	AAGCTT	6
5,721	GTATAC	6
5,895	TGATCA	6
5,905	TAATTA	6
5,949	GCCGGC	6
6,175	CAATTG	6
6,184	TCCTAGGA	8
6,185	CCTAGG	6
6,211	ATATAT	6
6,231	GACGTC	6
6,234	GTCGAC	6
6,301	TCAGCTGA	8
6,302	CAGCTG	6
6,340	GAGCTC	6
6,445	ATACGTAT	8
6,446	TACGTA	6

6,499	TTGCAA	6
6,703	CCATGG	6
6,748	TCATGA	6
6,760	AAGCTT	6
6,852	TTCGAA	6
6,867	TACGTA	6
7,043	AGGCCT	6
7,119	CTAATTAG	8
7,120	TAATTA	6
7,167	AAGCTT	6
7,182	AGTACT	6
7,186	CTATAG	6
7,240	TGATCA	6
7,267	GAATTC	6
7,424	ACTTCTAGAAGT	12
7,425	CTTCTAGAAG	10
7,426	TTCTAGAA	8
7,427	TCTAGA	6
7,474	GTATAC	6
7,505	CTCATGAG	8
7,506	TCATGA	6
7,523	CCTAGG	6
7,677	TTCGAA	6
7,802	TGATTAATCA	10
7,803	GATTAATC	8
7,804	ATTAAT	6
8,145	ATTAAT	6
8,241	CCTAGG	6
8,248	ATAGCTAT	8
8,249	TAGCTA	6
8,273	CAGTAATTACTG	12
8,274	AGTAATTACT	10
8,275	GTAATTAC	8
8,276	TAATTA	6
8,396	CCATGG	6
8,445	ATTAAT	6
8,491	AGTACT	6
8,507	TAATTA	6
8,567	AAGCTT	6
8,585	TACTAGTA	8
8,586	ACTAGT	6
8,677	GAGCTC	6

8,750	CCTAGG	6
8,778	ATATAT	6
9,035	CTCCGGAG	8
9,036	TCCGGA	6
9,054	TGGGCCCA	8
9,055	GGGCCC	6
9,122	CCTAGG	6
9,125	AGGCCT	6
9,321	TTCGAA	6
9,327	GCAGCTGC	8
9,328	CAGCTG	6
9,441	AACTAGTT	8
9,442	ACTAGT	6
9,479	TAATATTA	8
9,480	AATATT	6
9,578	CATATG	6
9,630	AAAATTTT	8
9,631	AAATTT	6
9,644	TAGCTA	6
9,822	TAATTA	6
9,897	TATATA	6
9,898	ATATAT	6
10,202	TAATATTA	8
10,203	AATATT	6
10,206	ATTAAT	6
10,249	ATTAAT	6
10,272	ATTAAT	6
10,274	TAATTA	6
10,371	ACTAGT	6
10,474	GTAATATTAC	10
10,475	TAATATTA	8
10,476	AATATT	6
10,493	TAATTA	6
10,496	TTATAA	6
10,565	TAATTA	6
10,690	GGTACC	6
10,704	ATTAAT	6
10,768	GCATGC	6
10,770	ATGCAT	6
10,882	CTTAAG	6
10,958	TTATAA	6
11,100	CTATATAG	8

11,101	TATATA	6
11,335	TCATGA	6
11,689	TTTAAA	6
11,754	TAATTA	6
11,830	TATATA	6
11,858	TACGTA	6
11,886	TCATGA	6
11,889	TGATCA	6
11,993	TTTAAA	6
12,047	TGATCA	6
12,058	AGAATTCT	8
12,059	GAATTC	6
12,098	AATCGATT	8
12,099	ATCGAT	6
12,110	AAGTACTT	8
12,111	AGTACT	6
12,260	CTGCAG	6
12,273	TATATA	6
12,592	CCTAGG	6
12,688	ATTAAT	6
12,775	CATATG	6
12,782	TCCGGA	6
12,842	TTTAAA	6
13,022	AGTACT	6
13,325	AACATGTT	8
13,326	ACATGT	6
13,411	TCTAGA	6
13,498	AGGCCT	6
13,664	CTATAG	6
13,767	TTTTAAAA	8
13,768	TTTAAA	6
13,868	TAGCTA	6
13,869	AGCTATAGCT	10
13,870	GCTATAGC	8
13,871	CTATAG	6
13,991	TAATTA	6
14,119	ACTAGT	6
14,162	CATTAATG	8
14,163	ATTAAT	6
14,288	TAATTA	6
14,380	GACGTC	6
14,485	TATATA	6

14,486	ATATAT	6
14,566	CCATGG	6
14,599	GTAATTAC	8
14,600	TAATTA	6
14,628	ATATAT	6
14,791	TCCGGA	6
14,794	GGAATTCC	8
14,795	GAATTC	6
14,853	CCTAGG	6
14,857	GGCGCC	6
14,891	TAGTACTA	8
14,892	AGTACT	6
15,111	AATATT	6
15,242	TCATGA	6
15,322	TAATTA	6
15,465	ATATAT	6
15,553	ATGCAT	6
15,568	TATATA	6
15,569	ATATAT	6
15,587	TACATGTA	8
15,588	ACATGT	6
15,631	CTCGAG	6
15,679	GGGCCC	6
15,819	TGATCA	6
15,896	TGGCCA	6
16,014	ATATAT	6
16,028	GTGCAC	6
16,035	CGTACG	6
16,045	CGTACG	6
16,055	CGTACG	6
16,065	CGTACG	6
16,075	CGTACG	6
16,085	CGTACG	6
16,095	CGTACG	6
16,105	CGTACG	6
16,115	CGTACG	6
16,125	CGTACG	6
16,135	CGTACG	6
16,145	CGTACG	6
16,155	CGTACG	6
16,165	CGTACG	6
16,175	CGTACG	6

16,193	CGTACG	6
16,227	CGTACG	6
16,240	TACGTA	6
16,243	GTATAC	6
16,311	GTATAC	6
16,323	TTATAA	6
16,371	ATATAT	6
16,399	CAATTG	6
16,424	TCTAGA	6
16,435	ATCTATAGAT	10
16,436	TCTATAGA	8
16,437	CTATAG	6
16,449	TTATAA	6

Table S5. Characteristics of the open reading frames (ORFs) identified in the *Mustela lutreola* mitogenome.

ORF	Strand	Frame	Start	Stop	Length (bp)	Length (aa) [1]
ORF1	+	2	143	259	117	38
ORF2	-	2	234	127	108	35
ORF3	-	1	382	197	186	61
ORF4	-	2	513	427	87	28
ORF5	-	1	637	536	102	33
ORF6	-	2	813	679	135	44
ORF7	+	3	888	977	90	29
ORF8	-	3	938	798	141	46
ORF9	-	2	1,077	967	111	36
ORF10	-	1	1,189	1,058	132	43
ORF11	+	2	1,418	1,513	96	31
ORF12	-	1	1,645	1,487	159	52
ORF13	+	1	1,732	1,824	93	30
ORF14	-	2	1,821	1,702	120	39
ORF15	-	1	2,218	2,087	132	43
ORF16	+	3	2,379	2,480	102	33
ORF17	-	1	2,434	2,357	78	25
ORF18	+	2	2,453	2,539	87	28
ORF19	+	1	2,476	2,574	99	32
ORF20	+	2	2,672	2,752	81	26
ORF21	-	2	2,727	2,650	78	25
ORF22	+	1	2,740	3,699	960	319
ORF23	+	3	3,537	3,701	165	54
ORF24	-	2	3,834	3,757	78	25
ORF25	+	3	3,882	4,952	1,071	356
ORF26	+	2	4,850	4,960	111	36
ORF27	+	3	5,166	5,303	138	45
ORF28	-	3	5,255	5,148	108	35
ORF29	+	3	5,304	6,884	1,581	526
ORF30	+	3	7,002	7,706	705	234

ORF31	+	2	7,778	7,981	204	67
ORF32	+	1	7,939	8,619	681	226
ORF33	+	3	8,547	9,446	900	299
ORF34	+	1	9,355	9,465	111	36
ORF35	+	1	9,469	9,828	360	119
ORF36	+	2	9,818	10,183	366	121
ORF37	+	1	10,171	11,700	1,530	509
ORF38	-	3	10,277	10,131	147	48
ORF39	+	2	11,654	13,576	1,923	640
ORF40	-	3	11,711	11,619	93	30
ORF41	-	2	11,913	11,647	267	88
ORF42	+	1	14,071	14,160	90	29
ORF43	-	3	14,096	13,560	537	178
ORF44	+	1	14,167	15,306	1,140	379
ORF45	-	3	14,222	14,109	114	37
ORF46	-	1	14,257	14,153	105	34
ORF47	-	3	15,362	15,258	105	34
ORF48	+	3	15,432	15,608	177	58
ORF49	+	1	15,484	15,609	126	41
ORF50	+	3	15,621	15,773	153	50
ORF51	+	1	15,685	15,810	126	41
ORF52	+	2	15,746	15,916	171	56
ORF53	+	3	15,810	15,971	162	53
ORF54	-	2	15,954	15,790	165	54
ORF55	-	1	16,372	15,941	432	143
ORF56	-	2	16,410	16,333	78	25

[1] length of putative protein-product; open reading frames coincide with identified protein-coding genes marked in gray.

Table S6. Characteristics of variable sites identified in the European mink mitogenome.

Position (bp) [1]	Mitogenome section	MW197425	MW197426	MW148603	MT304869	MW197423	MW197424	AB119070	AF207714	AF207713	AF207712	AF068544	EU548044	EU548051	EU548039	EU548036	EU548043	EU548045	EU548041	JX982495	JX982502	JX982498	JX982501	JX982499	JX982500	Type of variable	Codon	Amino acid
		**Specimen / SNVs [2] / haplotype**																										
162	*rrnS*	G	G	G	G	G	C	G	?	?	?	?	?	?	?	?	?	?	?	?	?	?	?	?	?	trs		
422		C	C	C	C	C	C	T	?	?	?	?	?	?	?	?	?	?	?	?	?	?	?	?	?	trn		
1,299	*rrnL*	A	A	A	T	T	A	?	?	?	?	?	?	?	?	?	?	?	?	?	?	?	?	?	?	trs		
2,061		T	T	T	T	T	C	?	?	?	?	?	?	?	?	?	?	?	?	?	?	?	?	?	?	trn		
2,195		T	T	T	C	C	T	?	?	?	?	?	?	?	?	?	?	?	?	?	?	?	?	?	?	trn		
2,958	*nad1*	C	C	C	C	C	T	?	?	?	?	?	?	?	?	?	?	?	?	?	?	?	?	?	?	trn	GTC→GTT [4]	Val→Val
3,024		C	C	C	C	C	A	?	?	?	?	?	?	?	?	?	?	?	?	?	?	?	?	?	?	trs	CCC→CCA [4]	Pro→Pro
4,238	*nad2*	T	T	T	T	T	C	?	?	?	?	?	?	?	?	?	?	?	?	?	?	?	?	?	?	trn	CCT→CCC [4]	Pro→Pro
4,569		T	T	T	C	C	T	?	?	?	?	?	?	?	?	?	?	?	?	?	?	?	?	?	?	trn	TTT→TTC	Phe→Phe
4,901		A	A	A	G	G	G	?	?	?	?	?	?	?	?	?	?	?	?	?	?	?	?	?	?	trn	GTA→GTG [4]	Val→Val
5,459	*cox1*	A	A	A	G	G	A	?	?	?	?	?	?	?	?	?	?	?	?	?	?	?	?	?	?	trn	GAA→GAG [4]	Glu→Glu
5,708		G	G	G	A	A	G	?	?	?	?	?	?	?	?	?	?	?	?	?	?	?	?	?	?	trn	GGG→GGA [4]	Gly→Gly

5,774		G	G	G	G	G	A	?	?	?	?	?	?	?	?	?	?	?	?	?	?	?	?	?	?	trn	CTG→CTA [4]	Leu→Leu
6,110		T	T	T	C	C	C	?	?	?	?	?	?	?	?	?	?	?	?	?	?	?	?	?	?	trn	GTT→GTC [4]	Val→Val
6,309		T	T	T	C	C	C	?	?	?	?	?	?	?	?	?	?	?	?	?	?	?	?	?	?	trn	TTA→CTA	Leu→Leu
7,406	*cox2*	A	A	A	G	G	G	?	?	?	?	?	?	?	?	?	?	?	?	?	?	?	?	?	?	trn	CTA→CTG [4]	Leu→Leu
8,253	*atp6*	T	T	T	T	T	C	?	?	?	?	?	?	?	?	?	?	?	?	?	?	?	?	?	?	trn	GCT→GCC [4]	Ala→Ala
8,571		T	T	T	C	C	T	?	?	?	?	?	?	?	?	?	?	?	?	?	?	?	?	?	?	trn	GCT→GCC [4]	Ala→Ala
9,142	*cox3*	T	T	T	T	T	C	?	?	?	?	?	?	?	?	?	?	?	?	?	?	?	?	?	?	trn	GTC→GCC	Val→Ala [5]
9,289		G	G	G	G	G	A	?	?	?	?	?	?	?	?	?	?	?	?	?	?	?	?	?	?	trn	AGC→AAC	Ser→Asn [5]
10,284	*nad4*	A	A	A	G	G	G	?	?	?	?	?	?	?	?	?	?	?	?	?	?	?	?	?	?	trn	CTA→CTG [4]	Leu→Leu
10,857		G	G	G	A	A	A	?	?	?	?	?	?	?	?	?	?	?	?	?	?	?	?	?	?	trn	GGG→GGA [4]	Gly→Gly
11,166		C	C	C	T	T	T	?	?	?	?	?	?	?	?	?	?	?	?	?	?	?	?	?	?	trn	GCC→GCT [4]	Ala→Ala
12,331	*nad5*	T	T	T	T	T	C	?	?	?	?	?	?	?	?	?	?	?	?	?	?	?	?	?	?	trn	AAT→AAC [4]	Asn→Asn
13,114		C	C	C	T	T	T	?	?	?	?	?	?	?	?	?	?	?	?	?	?	?	?	?	?	trn	TCC→TCT [4]	Ser→Ser

13,141		C	C	C	T	T	T	?	?	?	?	?	?	?	?	?	?	?	?	?	?	?	?	?	?	trn	ATC→ATT [4]	Ile→Ile
13,205		T	T	T	C	C	C	?	?	?	?	?	?	?	?	?	?	?	?	?	?	?	?	?	?	trn	TAT→TAC [4]	Tyr→Tyr
13,462		C	C	C	C	C	T	?	?	?	?	?	?	?	?	?	?	?	?	?	?	?	?	?	?	trn	TTC→TTT [4]	Phe→Phe
13,887	*nad6*	T	T	T	T	T	C	?	?	?	?	?	?	?	?	?	?	?	?	?	?	?	?	?	?	trn	GGA→GGG [4]	Gly→Gly
14,218		C	C	C	C	C	C	?	T	C	C	C	?	?	?	?	?	?	?	?	?	?	?	?	?	trn	CTC→TTC	Leu→Phe [5]
14,233		G	G	G	G	G	G	?	G	A	G	G	?	?	?	?	?	?	?	?	?	?	?	?	?	trn	GCC→ACC	Ala→Thr [5]
14,406		A	A	A	A	A	A	?	A	A	G	A	?	?	?	?	?	?	?	?	?	?	?	?	?	trn	CGA→CGG [4]	Arg→Arg
14,571	*Cytb*	G	G	G	G	G	A	?	G	G	G	G	?	?	?	?	?	?	?	?	?	?	?	?	?	trn	TGG→TGA [4]	Trp→Trp
14,784		T	T	T	C	C	T	?	T	T	T	T	?	?	?	?	?	?	?	?	?	?	?	?	?	trn	AAT→AAC [4]	Asn→Asn
15,147		G	G	G	G	G	A	?	G	G	G	G	?	?	?	?	?	?	?	?	?	?	?	?	?	trn	CTG→CTA [4]	Leu→Leu
15,204		G	G	G	A	A	A	?	?	?	?	?	?	?	?	?	?	?	?	?	?	?	?	?	?	trn	CCG→CCA [4]	Pro→Pro
15,272		T	T	T	T	T	T	?	?	?	?	?	C	T	T	T	C	T	T	?	?	?	?	?	?	trn	ATC→ACC	Ile→Thr [5]
15,396	*tRNA^Pro^*	G	G	G	G	G	G	?	?	?	?	?	G	T	G	G	G	G	G	?	?	?	?	?	?	trn		
15,463	control	T	T	T	T	T	T	?	?	?	?	?	T	C	T	T	T	T	T	?	?	?	?	?	?	trn		
15,470	region	T	T	T	T	T	T	?	?	?	?	?	T	C	T	T	T	T	T	?	?	?	?	?	?	trn		

15,474		A	A	A	A	A	A	?	?	?	?	?	A	A	G	A	A	A	A	A	A	A	A	A	A	trn
15,488		G	G	G	A	A	G	?	?	?	?	?	G	G	A	G	G	G	G	G	G	G	A	A	G	trn
15,489		C	C	C	C	C	C	?	?	?	?	?	C	C	C	C	C	C	C	G	C	C	C	C	C	trs
15,513		-	-	T	T	T	T	?	?	?	?	?	-	-	T	T	-	T	T	-	-	-	-	T	-	indel
15,514		T	T	T	T	T	T	?	?	?	?	?	-	-	-	-	-	T	T	-	-	-	-	-	-	indel
15,515		T	T	T	T	T	T	?	?	?	?	?	T	T	C	T	T	T	T	T	T	T	C	T	T	trn
15,518		C	C	C	C	C	C	?	?	?	?	?	C	C	C	C	C	C	C	C	T	C	C	C	C	trn
15,523		C	C	C	C	C	C	?	?	?	?	?	T	T	C	C	T	C	C	C	C	C	C	C	C	trn
15,579		G	G	G	G	G	A	?	?	?	?	?	G	G	G	G	G	G	G	G	G	G	G	G	G	trn
15,580		G	G	G	G	G	G	?	?	?	?	?	G	G	G	G	G	G	G	G	G	A	G	G	G	trn
15,585		T	T	T	T	T	T	?	?	?	?	?	T	T	T	C	T	T	T	T	T	T	T	T	T	trn
15,594		T	T	T	T	T	T	?	?	?	?	?	T	T	T	T	T	T	T	T	T	T	A	T	T	trs
15,599		C	C	C	C	C	T	?	?	?	?	?	C	C	C	C	C	C	C	C	C	C	T	C	C	trn
15,601		C	C	C	T	T	C	?	?	?	?	?	C	C	T	T	C	C	C	C	T	C	T	T	T	trn
15,604		C	C	C	C	C	C	?	?	?	?	?	C	C	C	C	G	C	C	C	C	C	C	C	C	trs
15,610		C	C	C	C	C	C	?	?	?	?	?	C	T	C	C	C	C	C	C	T	C	C	C	C	trn
15,616		G	G	G	G	G	G	?	?	?	?	?	G	G	G	G	G	G	G	G	G	G	A	G	G	trn
15,688		A	A	A	A	A	A	?	?	?	?	?	A	A	A	A	A	G	A	A	A	A	A	A	A	trn
15,701		C	C	C	C	C	T	?	?	?	?	?	T	C	C	C	T	T	T	C	C	T	C	C	C	trn
15,720		T	T	T	T	T	T	?	?	?	?	?	T	T	T	T	T	T	T	G	T	T	T	T	T	trn
15,749		T	T	T	T	T	T	?	?	?	?	?	T	T	T	T	T	T	T	T	C	T	T	T	T	trn
15,753		T	T	T	T	T	T	?	?	?	?	?	T	T	T	T	T	T	C	T	T	T	T	T	T	trn
15,901		A	A	A	A	A	A	?	?	?	?	?	A	A	A	A	A	A	A	T	A	A	A	A	A	trs
15,928		T	T	T	T	T	T	?	?	?	?	?	T	T	T	T	T	T	T	T	T	T	T	C	T	trn

15,973	C	C	C	C	C	C	?	?	?	?	?	C	C	C	C	C	C	C	C	C	C	C	C	T	trn
16,170	G	G	G	G	G	A	?	?	?	?	?	?	?	?	?	?	?	?	?	?	?	?	?	?	trn
16,174	-	A	A	A	A	-	?	?	?	?	?	?	?	?	?	?	?	?	?	?	?	?	?	?	indel
16,175	-	C	C	C	C	-	?	?	?	?	?	?	?	?	?	?	?	?	?	?	?	?	?	?	indel
16,189 +1	-	-	-	G	G	G	?	?	?	?	?	?	?	?	?	?	?	?	?	?	?	?	?	?	indel
16,189 +2	-	-	-	C	C	C	?	?	?	?	?	?	?	?	?	?	?	?	?	?	?	?	?	?	indel
16,198	A	A	G	G	G	G	?	?	?	?	?	?	?	?	?	?	?	?	?	?	?	?	?	?	trn
16,208	A	A	A	G	G	A	?	?	?	?	?	?	?	?	?	?	?	?	?	?	?	?	?	?	trn
16,212	G	G	G	A	A	G	?	?	?	?	?	?	?	?	?	?	?	?	?	?	?	?	?	?	trn
16,213	T	T	T	C	C	T	?	?	?	?	?	?	?	?	?	?	?	?	?	?	?	?	?	?	trn
16,214 +1	-	-	-	T	T	-	?	?	?	?	?	?	?	?	?	?	?	?	?	?	?	?	?	?	indel
16,214 +2	-	-	-	A	A	-	?	?	?	?	?	?	?	?	?	?	?	?	?	?	?	?	?	?	indel
16,214 +3	-	-	-	C	C	C	?	?	?	?	?	?	?	?	?	?	?	?	?	?	?	?	?	?	indel
16,214 +4	-	-	-	G	G	G	?	?	?	?	?	?	?	?	?	?	?	?	?	?	?	?	?	?	indel
16,223 +1	-	-	-	G	G	-	?	?	?	?	?	?	?	?	?	?	?	?	?	?	?	?	?	?	indel
16,223 +2	-	-	-	C	C	-	?	?	?	?	?	?	?	?	?	?	?	?	?	?	?	?	?	?	indel
16,232	G	G	G	G	G	A	?	?	?	?	?	?	?	?	?	?	?	?	?	?	?	?	?	?	trn

[1] the numbering given is relative to the reference sequence, [2] single nucleotide variant, [3] trs – transversion, trn – transition, indel – insertion-deletion variation, [4] single nucleotide variability in the codon's wobble position, [5] nonsynonymous (missense) substitution; a dash indicates a deletion; ? – no data; single nucleotide variants identified by alignment with the mtDNA sequences of *M. lutreola* deposited in the GenBank (Accession No.: AB026105, EF689084, EF689085, EF987742, EU548039, EU548040, EU548041, EU548045, EU548046, EU548035, EU548036, EU548037, EU548038, EU548047, EU548048, EU548049, EU548050, EU548042, EU548043, EU548044, EU548051, AB051263, AF068544, AF207712, AF207713, AF207714, AY750628, AB119070, AB601576, JX982499, JX982495, JX982496, JX982497, JX982498, JX982500, JX982501, JX982502, AF207721, AF207724, AF207725, AF207720, AF207722, AF207723) marked in gray.

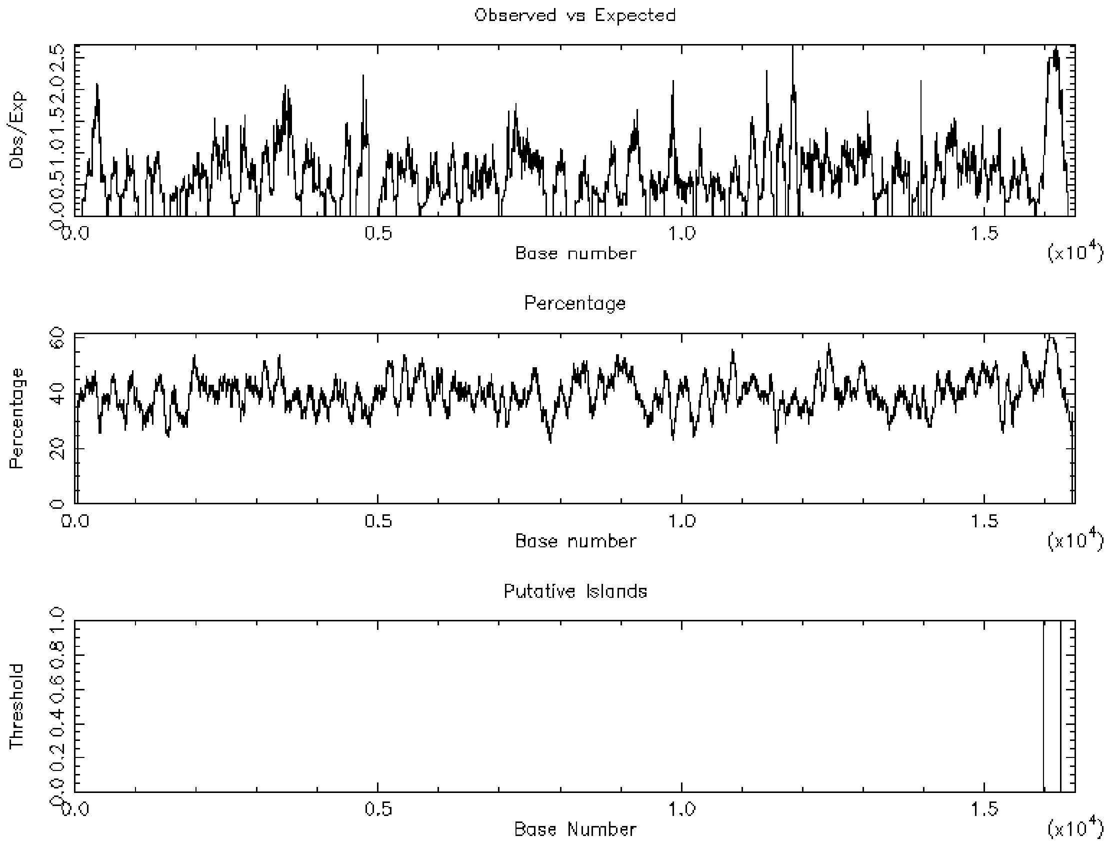

Figure S1. Graphical analysis of the European mink mitogenome sequence for the presence and distribution of CpG dinucleotides (upper graph – distribution of observed/expected ratios of CpG dinucleotides, middle graph – distribution of C+G nucleotides, lower graph – identification of putative CpG island).

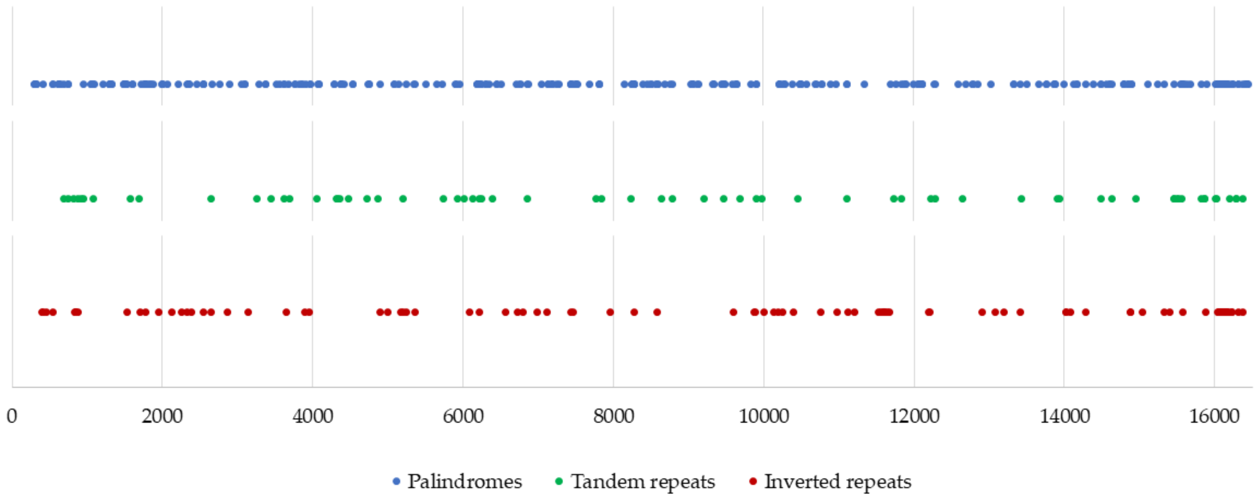

Figure S2. Distribution of repetitive and palindromic sequences in the European mink mitochondrial genome.

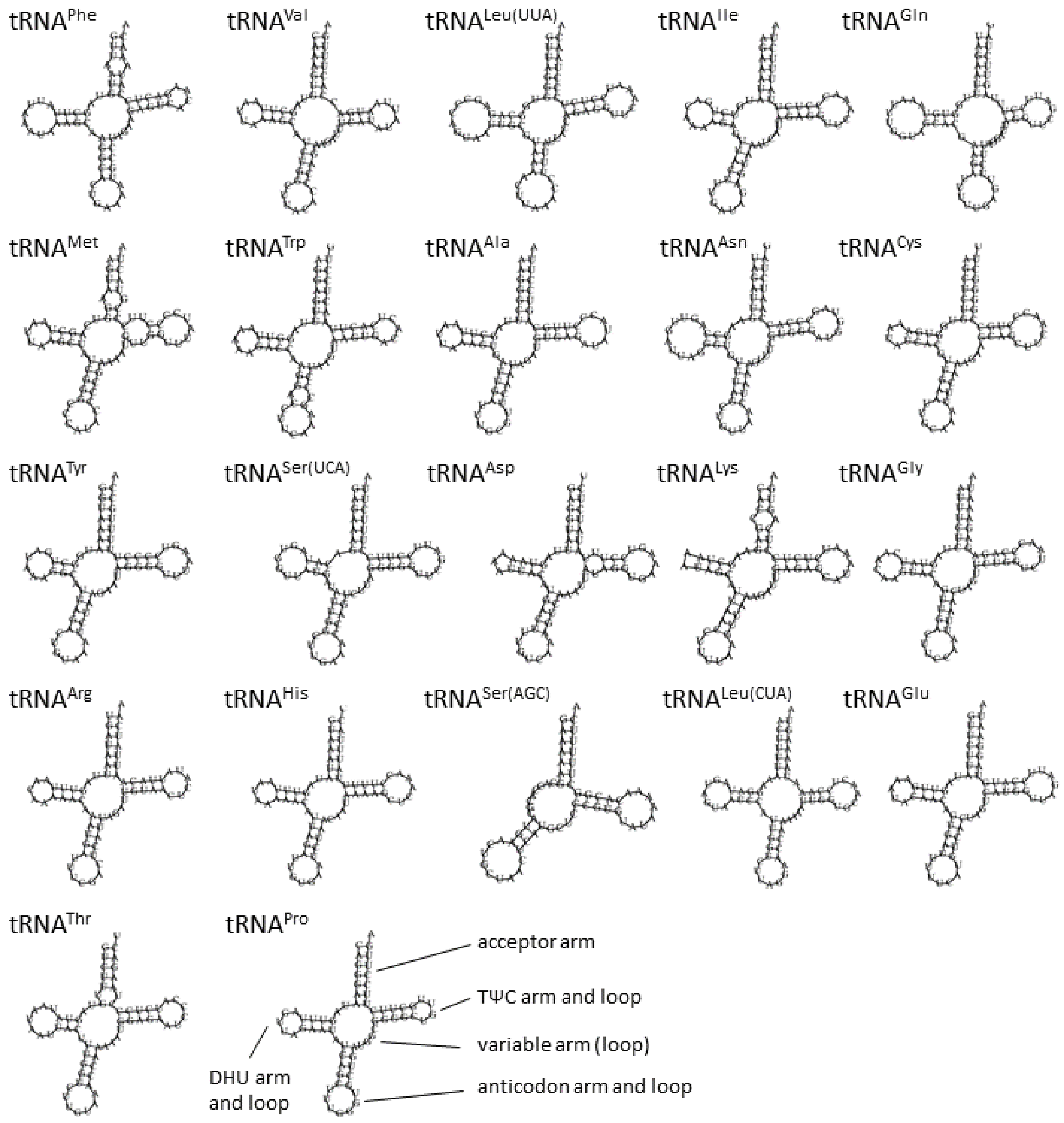

Figure S3. Predicted secondary structures of 22 mitochondrial tRNAs in European mink.

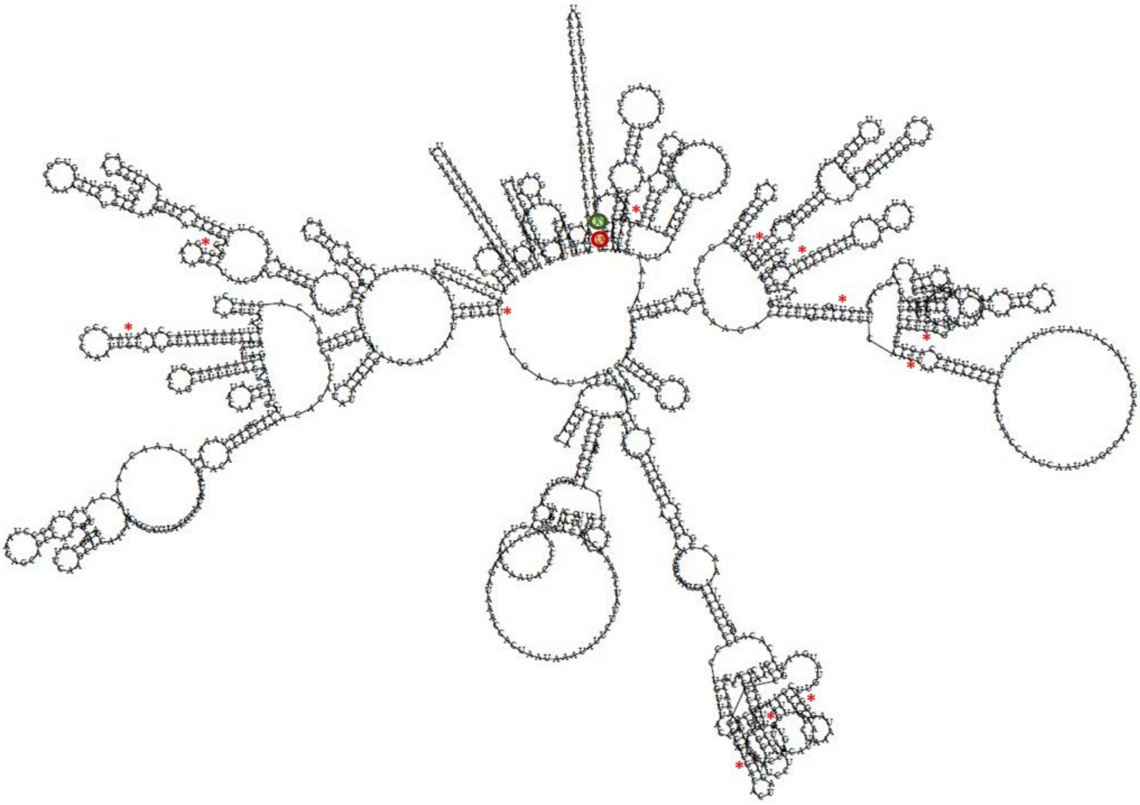

Figure S4. Predicted secondary structure of the mitochondrial ribosomal RNA of the large ribosomal subunit in *Mustela lutreola* (dashes indicate Watson-Crick base pairing; red asterisks indicate non-canonical G-U pairs; the 5′-end is marked with a green circle and the 3′-end with a red circle).

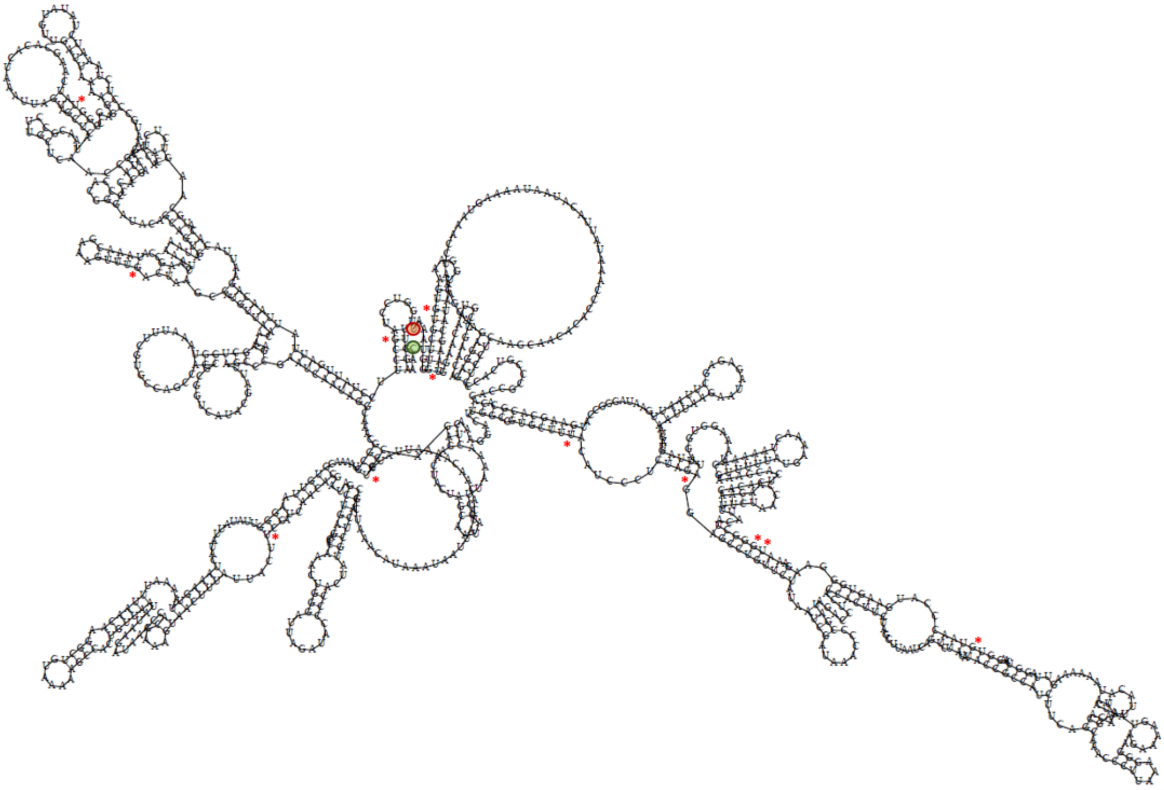

Figure S5. Predicted secondary structure of the mitochondrial ribosomal RNA of the small ribosomal subunit in *Mustela lutreola* (dashes indicate Watson-Crick base pairing; red asterisks indicate non-canonical G-U pairs; the 5′-end is marked with a green circle and the 3′-end with a red circle).

```
    JX982502.1          ------------------------------------------------------
------ 0
    JX982501.1          ------------------------------------------------------
------ 0
    JX982498.1          ------------------------------------------------------
------ 0
    JX982497.1          ------------------------------------------------------
------ 0
    JX982495.1          ------------------------------------------------------
------ 0
    JX982496.1          ------------------------------------------------------
------ 0
    JX982500.1          ------------------------------------------------------
------ 0
    EU548051.1          ------------------------------------------------------
------ 0
    EU548044.1          ------------------------------------------------------
------ 0
    EU548042.1          ------------------------------------------------------
------ 0
    EU548043.1          ------------------------------------------------------
------ 0
    EU548047.1          ------------------------------------------------------
------ 0
    EU548050.1          ------------------------------------------------------
------ 0
    EU548048.1          ------------------------------------------------------
------ 0
    EU548049.1          ------------------------------------------------------
------ 0
    AF207722.1          ------------------------------------------------------
------ 0
    EU548037.1          ------------------------------------------------------
------ 0
    AF207723.1          ------------------------------------------------------
------ 0
    EU548038.1          ------------------------------------------------------
------ 0
    EU548036.1          ------------------------------------------------------
------ 0
    EU548035.1          ------------------------------------------------------
------ 0
    AF207720.1          ------------------------------------------------------
------ 0
    AB601576.1          ------------------------------------------------------
------ 0
    EU548040.1          ------------------------------------------------------
------ 0
    EU548039.1          ------------------------------------------------------
------ 0
    AF207721.1          ------------------------------------------------------
------ 0
    AF207724.1          ------------------------------------------------------
------ 0
    JX982499.1          ------------------------------------------------------
------ 0
    EU548041.1          ------------------------------------------------------
------ 0
    EU548045.1          ------------------------------------------------------
------ 0
    AF207725.1          ------------------------------------------------------
------ 0
    EU548046.1          ------------------------------------------------------
------ 0
```

```
   AF207714.1      ------------------------------------------------------
------ 0
   AF207713.1      ------------------------------------------------------
------ 0
   AF207712.1      ------------------------------------------------------
------ 0
   AY750628.1      ------------------------------------------------------
------ 0
   EF689084.1      ------------------------------------------------------
------ 0
   EF689085.1      ------------------------------------------------------
------ 0
   AB119070.1      ------------------------------------------------------
------ 0
   EF987742.1      ------------------------------------------------------
------ 0
   AB026105.1      ------------------------------------------------------
------ 0
   MW148603.1
GTTAATGTAGCTTATTAAATAAAGCAAGGCACTGAAAATGCCTAGAAGAGTCACAAGACT   60
   AB051263.1      ------------------------------------------------------
------ 0
   AF068544.1      ------------------------------------------------------
------ 0


   JX982502.1      ------------------------------------------------------
------ 0
   JX982501.1      ------------------------------------------------------
------ 0
   JX982498.1      ------------------------------------------------------
------ 0
   JX982497.1      ------------------------------------------------------
------ 0
   JX982495.1      ------------------------------------------------------
------ 0
   JX982496.1      ------------------------------------------------------
------ 0
   JX982500.1      ------------------------------------------------------
------ 0
   EU548051.1      ------------------------------------------------------
------ 0
   EU548044.1      ------------------------------------------------------
------ 0
   EU548042.1      ------------------------------------------------------
------ 0
   EU548043.1      ------------------------------------------------------
------ 0
   EU548047.1      ------------------------------------------------------
------ 0
   EU548050.1      ------------------------------------------------------
------ 0
   EU548048.1      ------------------------------------------------------
------ 0
   EU548049.1      ------------------------------------------------------
------ 0
   AF207722.1      ------------------------------------------------------
------ 0
   EU548037.1      ------------------------------------------------------
------ 0
   AF207723.1      ------------------------------------------------------
------ 0
   EU548038.1      ------------------------------------------------------
------ 0
   EU548036.1      ------------------------------------------------------
------ 0
```

```
   EU548035.1      ------------------------------------------------------
------ 0
   AF207720.1      ------------------------------------------------------
------ 0
   AB601576.1      ------------------------------------------------------
------ 0
   EU548040.1      ------------------------------------------------------
------ 0
   EU548039.1      ------------------------------------------------------
------ 0
   AF207721.1      ------------------------------------------------------
------ 0
   AF207724.1      ------------------------------------------------------
------ 0
   JX982499.1      ------------------------------------------------------
------ 0
   EU548041.1      ------------------------------------------------------
------ 0
   EU548045.1      ------------------------------------------------------
------ 0
   AF207725.1      ------------------------------------------------------
------ 0
   EU548046.1      ------------------------------------------------------
------ 0
   AF207714.1      ------------------------------------------------------
------ 0
   AF207713.1      ------------------------------------------------------
------ 0
   AF207712.1      ------------------------------------------------------
------ 0
   AY750628.1      ------------------------------------------------------
------ 0
   EF689084.1      ------------------------------------------------------
------ 0
   EF689085.1      ------------------------------------------------------
------ 0
   AB119070.1                                                  ------------
AGGTTTGGTCCTAGCCTTCCTATTGATTATTAACAGAATTACACATGC 48
   EF987742.1      ------------------------------------------------------
------ 0
   AB026105.1      ------------------------------------------------------
------ 0
   MW148603.1
CCATAAACACAAAGGTTTGGTCCTAGCCTTCCTATTGATTATTAACAGAATTACACATGC   120
   AB051263.1      ------------------------------------------------------
------ 0
   AF068544.1      ------------------------------------------------------
------ 0


   JX982502.1      ------------------------------------------------------
------ 0
   JX982501.1      ------------------------------------------------------
------ 0
   JX982498.1      ------------------------------------------------------
------ 0
   JX982497.1      ------------------------------------------------------
------ 0
   JX982495.1      ------------------------------------------------------
------ 0
   JX982496.1      ------------------------------------------------------
------ 0
   JX982500.1      ------------------------------------------------------
------ 0
   EU548051.1      ------------------------------------------------------
------ 0
```

```
    EU548044.1      ------------------------------------------------------
------ 0
    EU548042.1      ------------------------------------------------------
------ 0
    EU548043.1      ------------------------------------------------------
------ 0
    EU548047.1      ------------------------------------------------------
------ 0
    EU548050.1      ------------------------------------------------------
------ 0
    EU548048.1      ------------------------------------------------------
------ 0
    EU548049.1      ------------------------------------------------------
------ 0
    AF207722.1      ------------------------------------------------------
------ 0
    EU548037.1      ------------------------------------------------------
------ 0
    AF207723.1      ------------------------------------------------------
------ 0
    EU548038.1      ------------------------------------------------------
------ 0
    EU548036.1      ------------------------------------------------------
------ 0
    EU548035.1      ------------------------------------------------------
------ 0
    AF207720.1      ------------------------------------------------------
------ 0
    AB601576.1      ------------------------------------------------------
------ 0
    EU548040.1      ------------------------------------------------------
------ 0
    EU548039.1      ------------------------------------------------------
------ 0
    AF207721.1      ------------------------------------------------------
------ 0
    AF207724.1      ------------------------------------------------------
------ 0
    JX982499.1      ------------------------------------------------------
------ 0
    EU548041.1      ------------------------------------------------------
------ 0
    EU548045.1      ------------------------------------------------------
------ 0
    AF207725.1      ------------------------------------------------------
------ 0
    EU548046.1      ------------------------------------------------------
------ 0
    AF207714.1      ------------------------------------------------------
------ 0
    AF207713.1      ------------------------------------------------------
------ 0
    AF207712.1      ------------------------------------------------------
------ 0
    AY750628.1      ------------------------------------------------------
------ 0
    EF689084.1      ------------------------------------------------------
------ 0
    EF689085.1      ------------------------------------------------------
------ 0
    AB119070.1
AAGTCTCTACACCCCAGTGAGAATGCCCTCTAAATCTATATGTTGATTAAAAGGAGCGGG   108
    EF987742.1      ------------------------------------------------------
------ 0
    AB026105.1      ------------------------------------------------------
------ 0
```

```
   MW148603.1
AAGTCTCTACACCCCAGTGAGAATGCCCTCTAAATCTATATGTTGATTAAAAGGAGCGGG   180
   AB051263.1      ------------------------------------------------------
------ 0
   AF068544.1      ------------------------------------------------------
------ 0


   JX982502.1      ------------------------------------------------------
------ 0
   JX982501.1      ------------------------------------------------------
------ 0
   JX982498.1      ------------------------------------------------------
------ 0
   JX982497.1      ------------------------------------------------------
------ 0
   JX982495.1      ------------------------------------------------------
------ 0
   JX982496.1      ------------------------------------------------------
------ 0
   JX982500.1      ------------------------------------------------------
------ 0
   EU548051.1      ------------------------------------------------------
------ 0
   EU548044.1      ------------------------------------------------------
------ 0
   EU548042.1      ------------------------------------------------------
------ 0
   EU548043.1      ------------------------------------------------------
------ 0
   EU548047.1      ------------------------------------------------------
------ 0
   EU548050.1      ------------------------------------------------------
------ 0
   EU548048.1      ------------------------------------------------------
------ 0
   EU548049.1      ------------------------------------------------------
------ 0
   AF207722.1      ------------------------------------------------------
------ 0
   EU548037.1      ------------------------------------------------------
------ 0
   AF207723.1      ------------------------------------------------------
------ 0
   EU548038.1      ------------------------------------------------------
------ 0
   EU548036.1      ------------------------------------------------------
------ 0
   EU548035.1      ------------------------------------------------------
------ 0
   AF207720.1      ------------------------------------------------------
------ 0
   AB601576.1      ------------------------------------------------------
------ 0
   EU548040.1      ------------------------------------------------------
------ 0
   EU548039.1      ------------------------------------------------------
------ 0
   AF207721.1      ------------------------------------------------------
------ 0
   AF207724.1      ------------------------------------------------------
------ 0
   JX982499.1      ------------------------------------------------------
------ 0
   EU548041.1      ------------------------------------------------------
------ 0
```

```
   EU548045.1      ------------------------------------------------------
------ 0
   AF207725.1      ------------------------------------------------------
------ 0
   EU548046.1      ------------------------------------------------------
------ 0
   AF207714.1      ------------------------------------------------------
------ 0
   AF207713.1      ------------------------------------------------------
------ 0
   AF207712.1      ------------------------------------------------------
------ 0
   AY750628.1      ------------------------------------------------------
------ 0
   EF689084.1      ------------------------------------------------------
------ 0
   EF689085.1      ------------------------------------------------------
------ 0
   AB119070.1
TATCAAGCACACTAAATTAGTAGCTCATAACGCCTTGCTCAACCACACCCCCACGGGATA   168
   EF987742.1      ------------------------------------------------------
------ 0
   AB026105.1      ------------------------------------------------------
------ 0
   MW148603.1
TATCAAGCACACTAAATTAGTAGCTCATAACGCCTTGCTCAACCACACCCCCACGGGATA   240
   AB051263.1      ------------------------------------------------------
------ 0
   AF068544.1      ------------------------------------------------------
------ 0


   JX982502.1      ------------------------------------------------------
------ 0
   JX982501.1      ------------------------------------------------------
------ 0
   JX982498.1      ------------------------------------------------------
------ 0
   JX982497.1      ------------------------------------------------------
------ 0
   JX982495.1      ------------------------------------------------------
------ 0
   JX982496.1      ------------------------------------------------------
------ 0
   JX982500.1      ------------------------------------------------------
------ 0
   EU548051.1      ------------------------------------------------------
------ 0
   EU548044.1      ------------------------------------------------------
------ 0
   EU548042.1      ------------------------------------------------------
------ 0
   EU548043.1      ------------------------------------------------------
------ 0
   EU548047.1      ------------------------------------------------------
------ 0
   EU548050.1      ------------------------------------------------------
------ 0
   EU548048.1      ------------------------------------------------------
------ 0
   EU548049.1      ------------------------------------------------------
------ 0
   AF207722.1      ------------------------------------------------------
------ 0
   EU548037.1      ------------------------------------------------------
------ 0
```

```
   AF207723.1      ------------------------------------------------------
------ 0
   EU548038.1      ------------------------------------------------------
------ 0
   EU548036.1      ------------------------------------------------------
------ 0
   EU548035.1      ------------------------------------------------------
------ 0
   AF207720.1      ------------------------------------------------------
------ 0
   AB601576.1      ------------------------------------------------------
------ 0
   EU548040.1      ------------------------------------------------------
------ 0
   EU548039.1      ------------------------------------------------------
------ 0
   AF207721.1      ------------------------------------------------------
------ 0
   AF207724.1      ------------------------------------------------------
------ 0
   JX982499.1      ------------------------------------------------------
------ 0
   EU548041.1      ------------------------------------------------------
------ 0
   EU548045.1      ------------------------------------------------------
------ 0
   AF207725.1      ------------------------------------------------------
------ 0
   EU548046.1      ------------------------------------------------------
------ 0
   AF207714.1      ------------------------------------------------------
------ 0
   AF207713.1      ------------------------------------------------------
------ 0
   AF207712.1      ------------------------------------------------------
------ 0
   AY750628.1      ------------------------------------------------------
------ 0
   EF689084.1      ------------------------------------------------------
------ 0
   EF689085.1      ------------------------------------------------------
------ 0
   AB119070.1
CAGCAGTGATAAAAATTAAGCCATAAACGAAAGTTTGACTAAGCCATGTTAACAAAGAGC   228
   EF987742.1      ------------------------------------------------------
------ 0
   AB026105.1      ------------------------------------------------------
------ 0
   MW148603.1
CAGCAGTGATAAAAATTAAGCCATAAACGAAAGTTTGACTAAGCCATGTTAACAAAGAGC   300
   AB051263.1      ------------------------------------------------------
------ 0
   AF068544.1      ------------------------------------------------------
------ 0


   JX982502.1      ------------------------------------------------------
------ 0
   JX982501.1      ------------------------------------------------------
------ 0
   JX982498.1      ------------------------------------------------------
------ 0
   JX982497.1      ------------------------------------------------------
------ 0
   JX982495.1      ------------------------------------------------------
------ 0
```

```
    JX982496.1        ------------------------------------------------------
------ 0
    JX982500.1        ------------------------------------------------------
------ 0
    EU548051.1        ------------------------------------------------------
------ 0
    EU548044.1        ------------------------------------------------------
------ 0
    EU548042.1        ------------------------------------------------------
------ 0
    EU548043.1        ------------------------------------------------------
------ 0
    EU548047.1        ------------------------------------------------------
------ 0
    EU548050.1        ------------------------------------------------------
------ 0
    EU548048.1        ------------------------------------------------------
------ 0
    EU548049.1        ------------------------------------------------------
------ 0
    AF207722.1        ------------------------------------------------------
------ 0
    EU548037.1        ------------------------------------------------------
------ 0
    AF207723.1        ------------------------------------------------------
------ 0
    EU548038.1        ------------------------------------------------------
------ 0
    EU548036.1        ------------------------------------------------------
------ 0
    EU548035.1        ------------------------------------------------------
------ 0
    AF207720.1        ------------------------------------------------------
------ 0
    AB601576.1        ------------------------------------------------------
------ 0
    EU548040.1        ------------------------------------------------------
------ 0
    EU548039.1        ------------------------------------------------------
------ 0
    AF207721.1        ------------------------------------------------------
------ 0
    AF207724.1        ------------------------------------------------------
------ 0
    JX982499.1        ------------------------------------------------------
------ 0
    EU548041.1        ------------------------------------------------------
------ 0
    EU548045.1        ------------------------------------------------------
------ 0
    AF207725.1        ------------------------------------------------------
------ 0
    EU548046.1        ------------------------------------------------------
------ 0
    AF207714.1        ------------------------------------------------------
------ 0
    AF207713.1        ------------------------------------------------------
------ 0
    AF207712.1        ------------------------------------------------------
------ 0
    AY750628.1        ------------------------------------------------------
------ 0
    EF689084.1        ------------------------------------------------------
------ 0
    EF689085.1        ------------------------------------------------------
------ 0
```

```
    AB119070.1
TGGTAAATTTCGTGCCAGCCACCGCGGTCATACGATTAGCCCGAATCAATAGGCAAACGG    288
    EF987742.1        ------------------------------------------------------
------ 0
    AB026105.1        ------------------------------------------------------
------ 0
    MW148603.1
TGGTAAATTTCGTGCCAGCCACCGCGGTCATACGATTAGCCCGAATCAATAGGCAAACGG    360
    AB051263.1        ------------------------------------------------------
------ 0
    AF068544.1        ------------------------------------------------------
------ 0


    JX982502.1        ------------------------------------------------------
------ 0
    JX982501.1        ------------------------------------------------------
------ 0
    JX982498.1        ------------------------------------------------------
------ 0
    JX982497.1        ------------------------------------------------------
------ 0
    JX982495.1        ------------------------------------------------------
------ 0
    JX982496.1        ------------------------------------------------------
------ 0
    JX982500.1        ------------------------------------------------------
------ 0
    EU548051.1        ------------------------------------------------------
------ 0
    EU548044.1        ------------------------------------------------------
------ 0
    EU548042.1        ------------------------------------------------------
------ 0
    EU548043.1        ------------------------------------------------------
------ 0
    EU548047.1        ------------------------------------------------------
------ 0
    EU548050.1        ------------------------------------------------------
------ 0
    EU548048.1        ------------------------------------------------------
------ 0
    EU548049.1        ------------------------------------------------------
------ 0
    AF207722.1        ------------------------------------------------------
------ 0
    EU548037.1        ------------------------------------------------------
------ 0
    AF207723.1        ------------------------------------------------------
------ 0
    EU548038.1        ------------------------------------------------------
------ 0
    EU548036.1        ------------------------------------------------------
------ 0
    EU548035.1        ------------------------------------------------------
------ 0
    AF207720.1        ------------------------------------------------------
------ 0
    AB601576.1        ------------------------------------------------------
------ 0
    EU548040.1        ------------------------------------------------------
------ 0
    EU548039.1        ------------------------------------------------------
------ 0
    AF207721.1        ------------------------------------------------------
------ 0
```

```
   AF207724.1      ------------------------------------------------------
------ 0
   JX982499.1      ------------------------------------------------------
------ 0
   EU548041.1      ------------------------------------------------------
------ 0
   EU548045.1      ------------------------------------------------------
------ 0
   AF207725.1      ------------------------------------------------------
------ 0
   EU548046.1      ------------------------------------------------------
------ 0
   AF207714.1      ------------------------------------------------------
------ 0
   AF207713.1      ------------------------------------------------------
------ 0
   AF207712.1      ------------------------------------------------------
------ 0
   AY750628.1      ------------------------------------------------------
------ 0
   EF689084.1      ------------------------------------------------------
------ 0
   EF689085.1      ------------------------------------------------------
------ 0
   AB119070.1
CGTAAAACGTGTTAAGGGTTATATTATACTAAAGTTAAAATTTAACAAGGCTGTAAAAAG   348
   EF987742.1      ------------------------------------------------------
------ 0
   AB026105.1      ------------------------------------------------------
------ 0
   MW148603.1
CGTAAAACGTGTTAAGGGTTATATTATACTAAAGTTAAAATTTAACAAGGCTGTAAAAAG   420
   AB051263.1      ------------------------------------------------------
------ 0
   AF068544.1      ------------------------------------------------------
------ 0


   JX982502.1      ------------------------------------------------------
------ 0
   JX982501.1      ------------------------------------------------------
------ 0
   JX982498.1      ------------------------------------------------------
------ 0
   JX982497.1      ------------------------------------------------------
------ 0
   JX982495.1      ------------------------------------------------------
------ 0
   JX982496.1      ------------------------------------------------------
------ 0
   JX982500.1      ------------------------------------------------------
------ 0
   EU548051.1      ------------------------------------------------------
------ 0
   EU548044.1      ------------------------------------------------------
------ 0
   EU548042.1      ------------------------------------------------------
------ 0
   EU548043.1      ------------------------------------------------------
------ 0
   EU548047.1      ------------------------------------------------------
------ 0
   EU548050.1      ------------------------------------------------------
------ 0
   EU548048.1      ------------------------------------------------------
------ 0
```

EU548049.1 --
------ 0
AF207722.1 --
------ 0
EU548037.1 --
------ 0
AF207723.1 --
------ 0
EU548038.1 --
------ 0
EU548036.1 --
------ 0
EU548035.1 --
------ 0
AF207720.1 --
------ 0
AB601576.1 --
------ 0
EU548040.1 --
------ 0
EU548039.1 --
------ 0
AF207721.1 --
------ 0
AF207724.1 --
------ 0
JX982499.1 --
------ 0
EU548041.1 --
------ 0
EU548045.1 --
------ 0
AF207725.1 --
------ 0
EU548046.1 --
------ 0
AF207714.1 --
------ 0
AF207713.1 --
------ 0
AF207712.1 --
------ 0
AY750628.1 --
------ 0
EF689084.1 --
------ 0
EF689085.1 --
------ 0
AB119070.1
CTACTGTTAATATAAGATAAACCACGAAAGTAACTTTATTACTTCCATCAACACGATAGC 408
EF987742.1 --
------ 0
AB026105.1 --
------ 0
MW148603.1
CCACTGTTAATATAAGATAAACCACGAAAGTAACTTTATTACTTCCATCAACACGATAGC 480
AB051263.1 --
------ 0
AF068544.1 --
------ 0

JX982502.1 --
------ 0
JX982501.1 --
------ 0

```
JX982498.1          ------------------------------------------------------
------ 0
JX982497.1          ------------------------------------------------------
------ 0
JX982495.1          ------------------------------------------------------
------ 0
JX982496.1          ------------------------------------------------------
------ 0
JX982500.1          ------------------------------------------------------
------ 0
EU548051.1          ------------------------------------------------------
------ 0
EU548044.1          ------------------------------------------------------
------ 0
EU548042.1          ------------------------------------------------------
------ 0
EU548043.1          ------------------------------------------------------
------ 0
EU548047.1          ------------------------------------------------------
------ 0
EU548050.1          ------------------------------------------------------
------ 0
EU548048.1          ------------------------------------------------------
------ 0
EU548049.1          ------------------------------------------------------
------ 0
AF207722.1          ------------------------------------------------------
------ 0
EU548037.1          ------------------------------------------------------
------ 0
AF207723.1          ------------------------------------------------------
------ 0
EU548038.1          ------------------------------------------------------
------ 0
EU548036.1          ------------------------------------------------------
------ 0
EU548035.1          ------------------------------------------------------
------ 0
AF207720.1          ------------------------------------------------------
------ 0
AB601576.1          ------------------------------------------------------
------ 0
EU548040.1          ------------------------------------------------------
------ 0
EU548039.1          ------------------------------------------------------
------ 0
AF207721.1          ------------------------------------------------------
------ 0
AF207724.1          ------------------------------------------------------
------ 0
JX982499.1          ------------------------------------------------------
------ 0
EU548041.1          ------------------------------------------------------
------ 0
EU548045.1          ------------------------------------------------------
------ 0
AF207725.1          ------------------------------------------------------
------ 0
EU548046.1          ------------------------------------------------------
------ 0
AF207714.1          ------------------------------------------------------
------ 0
AF207713.1          ------------------------------------------------------
------ 0
AF207712.1          ------------------------------------------------------
------ 0
```

```
   AY750628.1      -----------------------------------------------------------
------ 0
   EF689084.1      -----------------------------------------------------------
------ 0
   EF689085.1      -----------------------------------------------------------
------ 0
   AB119070.1
TGAGACCCAAACTGGGATTAGATACCCCACTATGCTCAGCCCTAAACATAAATAATTATC   468
   EF987742.1      -----------------------------------------------------------
------ 0
   AB026105.1      -----------------------------------------------------------
------ 0
   MW148603.1
TGAGACCCAAACTGGGATTAGATACCCCACTATGCTCAGCCCTAAACATAAATAATTATC   540
   AB051263.1      -----------------------------------------------------------
------ 0
   AF068544.1      -----------------------------------------------------------
------ 0


   JX982502.1      -----------------------------------------------------------
------ 0
   JX982501.1      -----------------------------------------------------------
------ 0
   JX982498.1      -----------------------------------------------------------
------ 0
   JX982497.1      -----------------------------------------------------------
------ 0
   JX982495.1      -----------------------------------------------------------
------ 0
   JX982496.1      -----------------------------------------------------------
------ 0
   JX982500.1      -----------------------------------------------------------
------ 0
   EU548051.1      -----------------------------------------------------------
------ 0
   EU548044.1      -----------------------------------------------------------
------ 0
   EU548042.1      -----------------------------------------------------------
------ 0
   EU548043.1      -----------------------------------------------------------
------ 0
   EU548047.1      -----------------------------------------------------------
------ 0
   EU548050.1      -----------------------------------------------------------
------ 0
   EU548048.1      -----------------------------------------------------------
------ 0
   EU548049.1      -----------------------------------------------------------
------ 0
   AF207722.1      -----------------------------------------------------------
------ 0
   EU548037.1      -----------------------------------------------------------
------ 0
   AF207723.1      -----------------------------------------------------------
------ 0
   EU548038.1      -----------------------------------------------------------
------ 0
   EU548036.1      -----------------------------------------------------------
------ 0
   EU548035.1      -----------------------------------------------------------
------ 0
   AF207720.1      -----------------------------------------------------------
------ 0
   AB601576.1      -----------------------------------------------------------
------ 0
```

```
    EU548040.1       ------------------------------------------------------
------ 0
    EU548039.1       ------------------------------------------------------
------ 0
    AF207721.1       ------------------------------------------------------
------ 0
    AF207724.1       ------------------------------------------------------
------ 0
    JX982499.1       ------------------------------------------------------
------ 0
    EU548041.1       ------------------------------------------------------
------ 0
    EU548045.1       ------------------------------------------------------
------ 0
    AF207725.1       ------------------------------------------------------
------ 0
    EU548046.1       ------------------------------------------------------
------ 0
    AF207714.1       ------------------------------------------------------
------ 0
    AF207713.1       ------------------------------------------------------
------ 0
    AF207712.1       ------------------------------------------------------
------ 0
    AY750628.1       ------------------------------------------------------
------ 0
    EF689084.1       ------------------------------------------------------
------ 0
    EF689085.1       ------------------------------------------------------
------ 0
    AB119070.1
ACAACAAAATTATCTGCCAGAGAACTACTAGCAATAGCTTAAAACTCAAAGGACTTGGCG   528
    EF987742.1       ------------------------------------------------------
------ 0
    AB026105.1       ------------------------------------------------------
------ 0
    MW148603.1
ACAACAAAATTATCTGCCAGAGAACTACTAGCAATAGCTTAAAACTCAAAGGACTTGGCG   600
    AB051263.1       ------------------------------------------------------
------ 0
    AF068544.1       ------------------------------------------------------
------ 0


    JX982502.1       ------------------------------------------------------
------ 0
    JX982501.1       ------------------------------------------------------
------ 0
    JX982498.1       ------------------------------------------------------
------ 0
    JX982497.1       ------------------------------------------------------
------ 0
    JX982495.1       ------------------------------------------------------
------ 0
    JX982496.1       ------------------------------------------------------
------ 0
    JX982500.1       ------------------------------------------------------
------ 0
    EU548051.1       ------------------------------------------------------
------ 0
    EU548044.1       ------------------------------------------------------
------ 0
    EU548042.1       ------------------------------------------------------
------ 0
    EU548043.1       ------------------------------------------------------
------ 0
```

EU548047.1 --
------ 0
EU548050.1 --
------ 0
EU548048.1 --
------ 0
EU548049.1 --
------ 0
AF207722.1 --
------ 0
EU548037.1 --
------ 0
AF207723.1 --
------ 0
EU548038.1 --
------ 0
EU548036.1 --
------ 0
EU548035.1 --
------ 0
AF207720.1 --
------ 0
AB601576.1 --
------ 0
EU548040.1 --
------ 0
EU548039.1 --
------ 0
AF207721.1 --
------ 0
AF207724.1 --
------ 0
JX982499.1 --
------ 0
EU548041.1 --
------ 0
EU548045.1 --
------ 0
AF207725.1 --
------ 0
EU548046.1 --
------ 0
AF207714.1 --
------ 0
AF207713.1 --
------ 0
AF207712.1 --
------ 0
AY750628.1 --
------ 0
EF689084.1 --
------ 0
EF689085.1 --
------ 0
AB119070.1
GTGCTTTACATCCCTCTAGAGGAGCCTGTTCTATAATCGATAAACCCCGATAGACCTCAC 588
EF987742.1 --
------ 0
AB026105.1 --
------ 0
MW148603.1
GTGCTTTACATCCCTCTAGAGGAGCCTGTTCTATAATCGATAAACCCCGATAGACCTCAC 660
AB051263.1 --
------ 0
AF068544.1 --
------ 0

```
    JX982502.1        ------------------------------------------------------
------ 0
    JX982501.1        ------------------------------------------------------
------ 0
    JX982498.1        ------------------------------------------------------
------ 0
    JX982497.1        ------------------------------------------------------
------ 0
    JX982495.1        ------------------------------------------------------
------ 0
    JX982496.1        ------------------------------------------------------
------ 0
    JX982500.1        ------------------------------------------------------
------ 0
    EU548051.1        ------------------------------------------------------
------ 0
    EU548044.1        ------------------------------------------------------
------ 0
    EU548042.1        ------------------------------------------------------
------ 0
    EU548043.1        ------------------------------------------------------
------ 0
    EU548047.1        ------------------------------------------------------
------ 0
    EU548050.1        ------------------------------------------------------
------ 0
    EU548048.1        ------------------------------------------------------
------ 0
    EU548049.1        ------------------------------------------------------
------ 0
    AF207722.1        ------------------------------------------------------
------ 0
    EU548037.1        ------------------------------------------------------
------ 0
    AF207723.1        ------------------------------------------------------
------ 0
    EU548038.1        ------------------------------------------------------
------ 0
    EU548036.1        ------------------------------------------------------
------ 0
    EU548035.1        ------------------------------------------------------
------ 0
    AF207720.1        ------------------------------------------------------
------ 0
    AB601576.1        ------------------------------------------------------
------ 0
    EU548040.1        ------------------------------------------------------
------ 0
    EU548039.1        ------------------------------------------------------
------ 0
    AF207721.1        ------------------------------------------------------
------ 0
    AF207724.1        ------------------------------------------------------
------ 0
    JX982499.1        ------------------------------------------------------
------ 0
    EU548041.1        ------------------------------------------------------
------ 0
    EU548045.1        ------------------------------------------------------
------ 0
    AF207725.1        ------------------------------------------------------
------ 0
    EU548046.1        ------------------------------------------------------
------ 0
```

```
    AF207714.1      ------------------------------------------------------
------ 0
    AF207713.1      ------------------------------------------------------
------ 0
    AF207712.1      ------------------------------------------------------
------ 0
    AY750628.1      ------------------------------------------------------
------ 0
    EF689084.1      ------------------------------------------------------
------ 0
    EF689085.1      ------------------------------------------------------
------ 0
    AB119070.1
CACTTCTAGCTTAATCAGTCTATATACCGCCATCTTCAGCAAACCCTTAAAGGGAAGAAA    648
    EF987742.1      ------------------------------------------------------
------ 0
    AB026105.1      ------------------------------------------------------
------ 0
    MW148603.1
CACTTCTAGCTTAATCAGTCTATATACCGCCATCTTCAGCAAACCCTTAAAGGGAAGAAA    720
    AB051263.1      ------------------------------------------------------
------ 0
    AF068544.1      ------------------------------------------------------
------ 0


    JX982502.1      ------------------------------------------------------
------ 0
    JX982501.1      ------------------------------------------------------
------ 0
    JX982498.1      ------------------------------------------------------
------ 0
    JX982497.1      ------------------------------------------------------
------ 0
    JX982495.1      ------------------------------------------------------
------ 0
    JX982496.1      ------------------------------------------------------
------ 0
    JX982500.1      ------------------------------------------------------
------ 0
    EU548051.1      ------------------------------------------------------
------ 0
    EU548044.1      ------------------------------------------------------
------ 0
    EU548042.1      ------------------------------------------------------
------ 0
    EU548043.1      ------------------------------------------------------
------ 0
    EU548047.1      ------------------------------------------------------
------ 0
    EU548050.1      ------------------------------------------------------
------ 0
    EU548048.1      ------------------------------------------------------
------ 0
    EU548049.1      ------------------------------------------------------
------ 0
    AF207722.1      ------------------------------------------------------
------ 0
    EU548037.1      ------------------------------------------------------
------ 0
    AF207723.1      ------------------------------------------------------
------ 0
    EU548038.1      ------------------------------------------------------
------ 0
    EU548036.1      ------------------------------------------------------
------ 0
```

```
   EU548035.1      ------------------------------------------------------
------ 0
   AF207720.1      ------------------------------------------------------
------ 0
   AB601576.1      ------------------------------------------------------
------ 0
   EU548040.1      ------------------------------------------------------
------ 0
   EU548039.1      ------------------------------------------------------
------ 0
   AF207721.1      ------------------------------------------------------
------ 0
   AF207724.1      ------------------------------------------------------
------ 0
   JX982499.1      ------------------------------------------------------
------ 0
   EU548041.1      ------------------------------------------------------
------ 0
   EU548045.1      ------------------------------------------------------
------ 0
   AF207725.1      ------------------------------------------------------
------ 0
   EU548046.1      ------------------------------------------------------
------ 0
   AF207714.1      ------------------------------------------------------
------ 0
   AF207713.1      ------------------------------------------------------
------ 0
   AF207712.1      ------------------------------------------------------
------ 0
   AY750628.1      ------------------------------------------------------
------ 0
   EF689084.1      ------------------------------------------------------
------ 0
   EF689085.1      ------------------------------------------------------
------ 0
   AB119070.1
AGTAAGCACAATAATATTACATAAAAAAGTTAGGTCAAGGTGTAACCCATGAAGTGGGAA   708
   EF987742.1      ------------------------------------------------------
------ 0
   AB026105.1      ------------------------------------------------------
------ 0
   MW148603.1
AGTAAGCACAATAATATTACATAAAAAAGTTAGGTCAAGGTGTAACCCATGAAGTGGGAA   780
   AB051263.1      ------------------------------------------------------
------ 0
   AF068544.1      ------------------------------------------------------
------ 0


   JX982502.1      ------------------------------------------------------
------ 0
   JX982501.1      ------------------------------------------------------
------ 0
   JX982498.1      ------------------------------------------------------
------ 0
   JX982497.1      ------------------------------------------------------
------ 0
   JX982495.1      ------------------------------------------------------
------ 0
   JX982496.1      ------------------------------------------------------
------ 0
   JX982500.1      ------------------------------------------------------
------ 0
   EU548051.1      ------------------------------------------------------
------ 0
```

```
   EU548044.1      ------------------------------------------------------
------ 0
   EU548042.1      ------------------------------------------------------
------ 0
   EU548043.1      ------------------------------------------------------
------ 0
   EU548047.1      ------------------------------------------------------
------ 0
   EU548050.1      ------------------------------------------------------
------ 0
   EU548048.1      ------------------------------------------------------
------ 0
   EU548049.1      ------------------------------------------------------
------ 0
   AF207722.1      ------------------------------------------------------
------ 0
   EU548037.1      ------------------------------------------------------
------ 0
   AF207723.1      ------------------------------------------------------
------ 0
   EU548038.1      ------------------------------------------------------
------ 0
   EU548036.1      ------------------------------------------------------
------ 0
   EU548035.1      ------------------------------------------------------
------ 0
   AF207720.1      ------------------------------------------------------
------ 0
   AB601576.1      ------------------------------------------------------
------ 0
   EU548040.1      ------------------------------------------------------
------ 0
   EU548039.1      ------------------------------------------------------
------ 0
   AF207721.1      ------------------------------------------------------
------ 0
   AF207724.1      ------------------------------------------------------
------ 0
   JX982499.1      ------------------------------------------------------
------ 0
   EU548041.1      ------------------------------------------------------
------ 0
   EU548045.1      ------------------------------------------------------
------ 0
   AF207725.1      ------------------------------------------------------
------ 0
   EU548046.1      ------------------------------------------------------
------ 0
   AF207714.1      ------------------------------------------------------
------ 0
   AF207713.1      ------------------------------------------------------
------ 0
   AF207712.1      ------------------------------------------------------
------ 0
   AY750628.1      ------------------------------------------------------
------ 0
   EF689084.1      ------------------------------------------------------
------ 0
   EF689085.1      ------------------------------------------------------
------ 0
   AB119070.1
GAAATGGGCTACATTTTCTAACCAAGAACACACTCACGAAAGTTTTTATGAAAACTAAAA   768
   EF987742.1      ------------------------------------------------------
------ 0
   AB026105.1      ------------------------------------------------------
------ 0
```

```
    MW148603.1
GAAATGGGCTACATTTTCTAACCAAGAACACACTCACGAAAGTTTTTATGAAAACTAAAA    840
    AB051263.1      ------------------------------------------------------
------ 0
    AF068544.1      ------------------------------------------------------
------ 0


    JX982502.1      ------------------------------------------------------
------ 0
    JX982501.1      ------------------------------------------------------
------ 0
    JX982498.1      ------------------------------------------------------
------ 0
    JX982497.1      ------------------------------------------------------
------ 0
    JX982495.1      ------------------------------------------------------
------ 0
    JX982496.1      ------------------------------------------------------
------ 0
    JX982500.1      ------------------------------------------------------
------ 0
    EU548051.1      ------------------------------------------------------
------ 0
    EU548044.1      ------------------------------------------------------
------ 0
    EU548042.1      ------------------------------------------------------
------ 0
    EU548043.1      ------------------------------------------------------
------ 0
    EU548047.1      ------------------------------------------------------
------ 0
    EU548050.1      ------------------------------------------------------
------ 0
    EU548048.1      ------------------------------------------------------
------ 0
    EU548049.1      ------------------------------------------------------
------ 0
    AF207722.1      ------------------------------------------------------
------ 0
    EU548037.1      ------------------------------------------------------
------ 0
    AF207723.1      ------------------------------------------------------
------ 0
    EU548038.1      ------------------------------------------------------
------ 0
    EU548036.1      ------------------------------------------------------
------ 0
    EU548035.1      ------------------------------------------------------
------ 0
    AF207720.1      ------------------------------------------------------
------ 0
    AB601576.1      ------------------------------------------------------
------ 0
    EU548040.1      ------------------------------------------------------
------ 0
    EU548039.1      ------------------------------------------------------
------ 0
    AF207721.1      ------------------------------------------------------
------ 0
    AF207724.1      ------------------------------------------------------
------ 0
    JX982499.1      ------------------------------------------------------
------ 0
    EU548041.1      ------------------------------------------------------
------ 0
```

```
   EU548045.1      ------------------------------------------------------
------ 0
   AF207725.1      ------------------------------------------------------
------ 0
   EU548046.1      ------------------------------------------------------
------ 0
   AF207714.1      ------------------------------------------------------
------ 0
   AF207713.1      ------------------------------------------------------
------ 0
   AF207712.1      ------------------------------------------------------
------ 0
   AY750628.1      ------------------------------------------------------
------ 0
   EF689084.1      ------------------------------------------------------
------ 0
   EF689085.1      ------------------------------------------------------
------ 0
   AB119070.1
ACTAAAGGTGGATTTAGTAGTAAATTAAGAATAGAGAGCTTAATTGAATAGGGCCATGAA   828
   EF987742.1      ------------------------------------------------------
------ 0
   AB026105.1      ------------------------------------------------------
------ 0
   MW148603.1
ACTAAAGGTGGATTTAGTAGTAAATTAAGAATAGAGAGCTTAATTGAATAGGGCCATGAA   900
   AB051263.1      ------------------------------------------------------
------ 0
   AF068544.1      ------------------------------------------------------
------ 0


   JX982502.1      ------------------------------------------------------
------ 0
   JX982501.1      ------------------------------------------------------
------ 0
   JX982498.1      ------------------------------------------------------
------ 0
   JX982497.1      ------------------------------------------------------
------ 0
   JX982495.1      ------------------------------------------------------
------ 0
   JX982496.1      ------------------------------------------------------
------ 0
   JX982500.1      ------------------------------------------------------
------ 0
   EU548051.1      ------------------------------------------------------
------ 0
   EU548044.1      ------------------------------------------------------
------ 0
   EU548042.1      ------------------------------------------------------
------ 0
   EU548043.1      ------------------------------------------------------
------ 0
   EU548047.1      ------------------------------------------------------
------ 0
   EU548050.1      ------------------------------------------------------
------ 0
   EU548048.1      ------------------------------------------------------
------ 0
   EU548049.1      ------------------------------------------------------
------ 0
   AF207722.1      ------------------------------------------------------
------ 0
   EU548037.1      ------------------------------------------------------
------ 0
```

```
    AF207723.1      ------------------------------------------------------
------ 0
    EU548038.1      ------------------------------------------------------
------ 0
    EU548036.1      ------------------------------------------------------
------ 0
    EU548035.1      ------------------------------------------------------
------ 0
    AF207720.1      ------------------------------------------------------
------ 0
    AB601576.1      ------------------------------------------------------
------ 0
    EU548040.1      ------------------------------------------------------
------ 0
    EU548039.1      ------------------------------------------------------
------ 0
    AF207721.1      ------------------------------------------------------
------ 0
    AF207724.1      ------------------------------------------------------
------ 0
    JX982499.1      ------------------------------------------------------
------ 0
    EU548041.1      ------------------------------------------------------
------ 0
    EU548045.1      ------------------------------------------------------
------ 0
    AF207725.1      ------------------------------------------------------
------ 0
    EU548046.1      ------------------------------------------------------
------ 0
    AF207714.1      ------------------------------------------------------
------ 0
    AF207713.1      ------------------------------------------------------
------ 0
    AF207712.1      ------------------------------------------------------
------ 0
    AY750628.1      ------------------------------------------------------
------ 0
    EF689084.1      ------------------------------------------------------
------ 0
    EF689085.1      ------------------------------------------------------
------ 0
    AB119070.1
GCACGCACACACCGCCCGTCACCCTCCTCAAGCAACACACCCAAATATTACATAATAAAA   888
    EF987742.1      ------------------------------------------------------
------ 0
    AB026105.1      ------------------------------------------------------
------ 0
    MW148603.1
GCACGCACACACCGCCCGTCACCCTCCTCAAGCAACACACCCAAATATTACATAATAAAA   960
    AB051263.1      ------------------------------------------------------
------ 0
    AF068544.1      ------------------------------------------------------
------ 0


    JX982502.1      ------------------------------------------------------
------ 0
    JX982501.1      ------------------------------------------------------
------ 0
    JX982498.1      ------------------------------------------------------
------ 0
    JX982497.1      ------------------------------------------------------
------ 0
    JX982495.1      ------------------------------------------------------
------ 0
```

```
    JX982496.1       ------------------------------------------------------
------ 0
    JX982500.1       ------------------------------------------------------
------ 0
    EU548051.1       ------------------------------------------------------
------ 0
    EU548044.1       ------------------------------------------------------
------ 0
    EU548042.1       ------------------------------------------------------
------ 0
    EU548043.1       ------------------------------------------------------
------ 0
    EU548047.1       ------------------------------------------------------
------ 0
    EU548050.1       ------------------------------------------------------
------ 0
    EU548048.1       ------------------------------------------------------
------ 0
    EU548049.1       ------------------------------------------------------
------ 0
    AF207722.1       ------------------------------------------------------
------ 0
    EU548037.1       ------------------------------------------------------
------ 0
    AF207723.1       ------------------------------------------------------
------ 0
    EU548038.1       ------------------------------------------------------
------ 0
    EU548036.1       ------------------------------------------------------
------ 0
    EU548035.1       ------------------------------------------------------
------ 0
    AF207720.1       ------------------------------------------------------
------ 0
    AB601576.1       ------------------------------------------------------
------ 0
    EU548040.1       ------------------------------------------------------
------ 0
    EU548039.1       ------------------------------------------------------
------ 0
    AF207721.1       ------------------------------------------------------
------ 0
    AF207724.1       ------------------------------------------------------
------ 0
    JX982499.1       ------------------------------------------------------
------ 0
    EU548041.1       ------------------------------------------------------
------ 0
    EU548045.1       ------------------------------------------------------
------ 0
    AF207725.1       ------------------------------------------------------
------ 0
    EU548046.1       ------------------------------------------------------
------ 0
    AF207714.1       ------------------------------------------------------
------ 0
    AF207713.1       ------------------------------------------------------
------ 0
    AF207712.1       ------------------------------------------------------
------ 0
    AY750628.1       ------------------------------------------------------
------ 0
    EF689084.1       ------------------------------------------------------
------ 0
    EF689085.1       ------------------------------------------------------
------ 0
```

```
    AB119070.1
GTAAACCTAAAGCAAGAGGAGACAAGTCGTAACAAGGTAAGCATACTGGAAAGTGTGCTT   948
    EF987742.1      ------------------------------------------------------
------ 0
    AB026105.1      ------------------------------------------------------
------ 0
    MW148603.1
GTAAACCTAAAGCAAGAGGAGACAAGTCGTAACAAGGTAAGCATACTGGAAAGTGTGCTT   1020
    AB051263.1      ------------------------------------------------------
------ 0
    AF068544.1      ------------------------------------------------------
------ 0


    JX982502.1      ------------------------------------------------------
------ 0
    JX982501.1      ------------------------------------------------------
------ 0
    JX982498.1      ------------------------------------------------------
------ 0
    JX982497.1      ------------------------------------------------------
------ 0
    JX982495.1      ------------------------------------------------------
------ 0
    JX982496.1      ------------------------------------------------------
------ 0
    JX982500.1      ------------------------------------------------------
------ 0
    EU548051.1      ------------------------------------------------------
------ 0
    EU548044.1      ------------------------------------------------------
------ 0
    EU548042.1      ------------------------------------------------------
------ 0
    EU548043.1      ------------------------------------------------------
------ 0
    EU548047.1      ------------------------------------------------------
------ 0
    EU548050.1      ------------------------------------------------------
------ 0
    EU548048.1      ------------------------------------------------------
------ 0
    EU548049.1      ------------------------------------------------------
------ 0
    AF207722.1      ------------------------------------------------------
------ 0
    EU548037.1      ------------------------------------------------------
------ 0
    AF207723.1      ------------------------------------------------------
------ 0
    EU548038.1      ------------------------------------------------------
------ 0
    EU548036.1      ------------------------------------------------------
------ 0
    EU548035.1      ------------------------------------------------------
------ 0
    AF207720.1      ------------------------------------------------------
------ 0
    AB601576.1      ------------------------------------------------------
------ 0
    EU548040.1      ------------------------------------------------------
------ 0
    EU548039.1      ------------------------------------------------------
------ 0
    AF207721.1      ------------------------------------------------------
------ 0
```

```
    AF207724.1      ------------------------------------------------------
------ 0
    JX982499.1      ------------------------------------------------------
------ 0
    EU548041.1      ------------------------------------------------------
------ 0
    EU548045.1      ------------------------------------------------------
------ 0
    AF207725.1      ------------------------------------------------------
------ 0
    EU548046.1      ------------------------------------------------------
------ 0
    AF207714.1      ------------------------------------------------------
------ 0
    AF207713.1      ------------------------------------------------------
------ 0
    AF207712.1      ------------------------------------------------------
------ 0
    AY750628.1      ------------------------------------------------------
------ 0
    EF689084.1      ------------------------------------------------------
------ 0
    EF689085.1      ------------------------------------------------------
------ 0
    AB119070.1      GGGTAAAT----------------------------------------------
------ 956
    EF987742.1      ------------------------------------------------------
------ 0
    AB026105.1      ------------------------------------------------------
------ 0
    MW148603.1
GGGTAAATCAAAGTGTAGCTTAATTAAAGCATCTGGCTTACACCCAGAAGATTTCATATA   1080
    AB051263.1      ------------------------------------------------------
------ 0
    AF068544.1      ------------------------------------------------------
------ 0


    JX982502.1      ------------------------------------------------------
------ 0
    JX982501.1      ------------------------------------------------------
------ 0
    JX982498.1      ------------------------------------------------------
------ 0
    JX982497.1      ------------------------------------------------------
------ 0
    JX982495.1      ------------------------------------------------------
------ 0
    JX982496.1      ------------------------------------------------------
------ 0
    JX982500.1      ------------------------------------------------------
------ 0
    EU548051.1      ------------------------------------------------------
------ 0
    EU548044.1      ------------------------------------------------------
------ 0
    EU548042.1      ------------------------------------------------------
------ 0
    EU548043.1      ------------------------------------------------------
------ 0
    EU548047.1      ------------------------------------------------------
------ 0
    EU548050.1      ------------------------------------------------------
------ 0
    EU548048.1      ------------------------------------------------------
------ 0
```

```
   EU548049.1      ------------------------------------------------------
------ 0
   AF207722.1      ------------------------------------------------------
------ 0
   EU548037.1      ------------------------------------------------------
------ 0
   AF207723.1      ------------------------------------------------------
------ 0
   EU548038.1      ------------------------------------------------------
------ 0
   EU548036.1      ------------------------------------------------------
------ 0
   EU548035.1      ------------------------------------------------------
------ 0
   AF207720.1      ------------------------------------------------------
------ 0
   AB601576.1      ------------------------------------------------------
------ 0
   EU548040.1      ------------------------------------------------------
------ 0
   EU548039.1      ------------------------------------------------------
------ 0
   AF207721.1      ------------------------------------------------------
------ 0
   AF207724.1      ------------------------------------------------------
------ 0
   JX982499.1      ------------------------------------------------------
------ 0
   EU548041.1      ------------------------------------------------------
------ 0
   EU548045.1      ------------------------------------------------------
------ 0
   AF207725.1      ------------------------------------------------------
------ 0
   EU548046.1      ------------------------------------------------------
------ 0
   AF207714.1      ------------------------------------------------------
------ 0
   AF207713.1      ------------------------------------------------------
------ 0
   AF207712.1      ------------------------------------------------------
------ 0
   AY750628.1      ------------------------------------------------------
------ 0
   EF689084.1      ------------------------------------------------------
------ 0
   EF689085.1      ------------------------------------------------------
------ 0
   AB119070.1      ------------------------------------------------------
------ 956
   EF987742.1      ------------------------------------------------------
------ 0
   AB026105.1      ------------------------------------------------------
------ 0
   MW148603.1
TTAATGACCACTTTGAACCAATACTAGCCCAACTTATCACTAACTCAATTATCACAGTCA   1140
   AB051263.1      ------------------------------------------------------
------ 0
   AF068544.1      ------------------------------------------------------
------ 0


   JX982502.1      ------------------------------------------------------
------ 0
   JX982501.1      ------------------------------------------------------
------ 0
```

```
    JX982498.1        ------------------------------------------------------
------ 0
    JX982497.1        ------------------------------------------------------
------ 0
    JX982495.1        ------------------------------------------------------
------ 0
    JX982496.1        ------------------------------------------------------
------ 0
    JX982500.1        ------------------------------------------------------
------ 0
    EU548051.1        ------------------------------------------------------
------ 0
    EU548044.1        ------------------------------------------------------
------ 0
    EU548042.1        ------------------------------------------------------
------ 0
    EU548043.1        ------------------------------------------------------
------ 0
    EU548047.1        ------------------------------------------------------
------ 0
    EU548050.1        ------------------------------------------------------
------ 0
    EU548048.1        ------------------------------------------------------
------ 0
    EU548049.1        ------------------------------------------------------
------ 0
    AF207722.1        ------------------------------------------------------
------ 0
    EU548037.1        ------------------------------------------------------
------ 0
    AF207723.1        ------------------------------------------------------
------ 0
    EU548038.1        ------------------------------------------------------
------ 0
    EU548036.1        ------------------------------------------------------
------ 0
    EU548035.1        ------------------------------------------------------
------ 0
    AF207720.1        ------------------------------------------------------
------ 0
    AB601576.1        ------------------------------------------------------
------ 0
    EU548040.1        ------------------------------------------------------
------ 0
    EU548039.1        ------------------------------------------------------
------ 0
    AF207721.1        ------------------------------------------------------
------ 0
    AF207724.1        ------------------------------------------------------
------ 0
    JX982499.1        ------------------------------------------------------
------ 0
    EU548041.1        ------------------------------------------------------
------ 0
    EU548045.1        ------------------------------------------------------
------ 0
    AF207725.1        ------------------------------------------------------
------ 0
    EU548046.1        ------------------------------------------------------
------ 0
    AF207714.1        ------------------------------------------------------
------ 0
    AF207713.1        ------------------------------------------------------
------ 0
    AF207712.1        ------------------------------------------------------
------ 0
```

```
   AY750628.1      ------------------------------------------------------
------ 0
   EF689084.1      ------------------------------------------------------
------ 0
   EF689085.1      ------------------------------------------------------
------ 0
   AB119070.1      ------------------------------------------------------
------ 956
   EF987742.1      ------------------------------------------------------
------ 0
   AB026105.1      ------------------------------------------------------
------ 0
   MW148603.1
CATAAATCAAAACATTTAATCACATTATTACAGTATAGGAGATAGAAATTCTACTTGGAG   1200
   AB051263.1      ------------------------------------------------------
------ 0
   AF068544.1      ------------------------------------------------------
------ 0


   JX982502.1      ------------------------------------------------------
------ 0
   JX982501.1      ------------------------------------------------------
------ 0
   JX982498.1      ------------------------------------------------------
------ 0
   JX982497.1      ------------------------------------------------------
------ 0
   JX982495.1      ------------------------------------------------------
------ 0
   JX982496.1      ------------------------------------------------------
------ 0
   JX982500.1      ------------------------------------------------------
------ 0
   EU548051.1      ------------------------------------------------------
------ 0
   EU548044.1      ------------------------------------------------------
------ 0
   EU548042.1      ------------------------------------------------------
------ 0
   EU548043.1      ------------------------------------------------------
------ 0
   EU548047.1      ------------------------------------------------------
------ 0
   EU548050.1      ------------------------------------------------------
------ 0
   EU548048.1      ------------------------------------------------------
------ 0
   EU548049.1      ------------------------------------------------------
------ 0
   AF207722.1      ------------------------------------------------------
------ 0
   EU548037.1      ------------------------------------------------------
------ 0
   AF207723.1      ------------------------------------------------------
------ 0
   EU548038.1      ------------------------------------------------------
------ 0
   EU548036.1      ------------------------------------------------------
------ 0
   EU548035.1      ------------------------------------------------------
------ 0
   AF207720.1      ------------------------------------------------------
------ 0
   AB601576.1      ------------------------------------------------------
------ 0
```

```
   EU548040.1      ------------------------------------------------------
------ 0
   EU548039.1      ------------------------------------------------------
------ 0
   AF207721.1      ------------------------------------------------------
------ 0
   AF207724.1      ------------------------------------------------------
------ 0
   JX982499.1      ------------------------------------------------------
------ 0
   EU548041.1      ------------------------------------------------------
------ 0
   EU548045.1      ------------------------------------------------------
------ 0
   AF207725.1      ------------------------------------------------------
------ 0
   EU548046.1      ------------------------------------------------------
------ 0
   AF207714.1      ------------------------------------------------------
------ 0
   AF207713.1      ------------------------------------------------------
------ 0
   AF207712.1      ------------------------------------------------------
------ 0
   AY750628.1      ------------------------------------------------------
------ 0
   EF689084.1      ------------------------------------------------------
------ 0
   EF689085.1      ------------------------------------------------------
------ 0
   AB119070.1      ------------------------------------------------------
------ 956
   EF987742.1      ------------------------------------------------------
------ 0
   AB026105.1      ------------------------------------------------------
------ 0
   MW148603.1
CTATAGAGAAAGTACCGCAAGGGAACGATGAAAGAAAAATTCAAAGTAATAAACAGCAAA   1260
   AB051263.1      ------------------------------------------------------
------ 0
   AF068544.1      ------------------------------------------------------
------ 0


   JX982502.1      ------------------------------------------------------
------ 0
   JX982501.1      ------------------------------------------------------
------ 0
   JX982498.1      ------------------------------------------------------
------ 0
   JX982497.1      ------------------------------------------------------
------ 0
   JX982495.1      ------------------------------------------------------
------ 0
   JX982496.1      ------------------------------------------------------
------ 0
   JX982500.1      ------------------------------------------------------
------ 0
   EU548051.1      ------------------------------------------------------
------ 0
   EU548044.1      ------------------------------------------------------
------ 0
   EU548042.1      ------------------------------------------------------
------ 0
   EU548043.1      ------------------------------------------------------
------ 0
```

```
   EU548047.1      ------------------------------------------------------
------ 0
   EU548050.1      ------------------------------------------------------
------ 0
   EU548048.1      ------------------------------------------------------
------ 0
   EU548049.1      ------------------------------------------------------
------ 0
   AF207722.1      ------------------------------------------------------
------ 0
   EU548037.1      ------------------------------------------------------
------ 0
   AF207723.1      ------------------------------------------------------
------ 0
   EU548038.1      ------------------------------------------------------
------ 0
   EU548036.1      ------------------------------------------------------
------ 0
   EU548035.1      ------------------------------------------------------
------ 0
   AF207720.1      ------------------------------------------------------
------ 0
   AB601576.1      ------------------------------------------------------
------ 0
   EU548040.1      ------------------------------------------------------
------ 0
   EU548039.1      ------------------------------------------------------
------ 0
   AF207721.1      ------------------------------------------------------
------ 0
   AF207724.1      ------------------------------------------------------
------ 0
   JX982499.1      ------------------------------------------------------
------ 0
   EU548041.1      ------------------------------------------------------
------ 0
   EU548045.1      ------------------------------------------------------
------ 0
   AF207725.1      ------------------------------------------------------
------ 0
   EU548046.1      ------------------------------------------------------
------ 0
   AF207714.1      ------------------------------------------------------
------ 0
   AF207713.1      ------------------------------------------------------
------ 0
   AF207712.1      ------------------------------------------------------
------ 0
   AY750628.1      ------------------------------------------------------
------ 0
   EF689084.1      ------------------------------------------------------
------ 0
   EF689085.1      ------------------------------------------------------
------ 0
   AB119070.1      ------------------------------------------------------
------ 956
   EF987742.1      ------------------------------------------------------
------ 0
   AB026105.1      ------------------------------------------------------
------ 0
   MW148603.1
GATTACACCTTATACCTTTTGCATAATGAGCTAGCTAGAATAATTCAGCAAAGAGATCTT   1320
   AB051263.1      ------------------------------------------------------
------ 0
   AF068544.1      ------------------------------------------------------
------ 0
```

```
    JX982502.1      ------------------------------------------------------
------ 0
    JX982501.1      ------------------------------------------------------
------ 0
    JX982498.1      ------------------------------------------------------
------ 0
    JX982497.1      ------------------------------------------------------
------ 0
    JX982495.1      ------------------------------------------------------
------ 0
    JX982496.1      ------------------------------------------------------
------ 0
    JX982500.1      ------------------------------------------------------
------ 0
    EU548051.1      ------------------------------------------------------
------ 0
    EU548044.1      ------------------------------------------------------
------ 0
    EU548042.1      ------------------------------------------------------
------ 0
    EU548043.1      ------------------------------------------------------
------ 0
    EU548047.1      ------------------------------------------------------
------ 0
    EU548050.1      ------------------------------------------------------
------ 0
    EU548048.1      ------------------------------------------------------
------ 0
    EU548049.1      ------------------------------------------------------
------ 0
    AF207722.1      ------------------------------------------------------
------ 0
    EU548037.1      ------------------------------------------------------
------ 0
    AF207723.1      ------------------------------------------------------
------ 0
    EU548038.1      ------------------------------------------------------
------ 0
    EU548036.1      ------------------------------------------------------
------ 0
    EU548035.1      ------------------------------------------------------
------ 0
    AF207720.1      ------------------------------------------------------
------ 0
    AB601576.1      ------------------------------------------------------
------ 0
    EU548040.1      ------------------------------------------------------
------ 0
    EU548039.1      ------------------------------------------------------
------ 0
    AF207721.1      ------------------------------------------------------
------ 0
    AF207724.1      ------------------------------------------------------
------ 0
    JX982499.1      ------------------------------------------------------
------ 0
    EU548041.1      ------------------------------------------------------
------ 0
    EU548045.1      ------------------------------------------------------
------ 0
    AF207725.1      ------------------------------------------------------
------ 0
    EU548046.1      ------------------------------------------------------
------ 0
```

```
   AF207714.1      ------------------------------------------------------
------ 0
   AF207713.1      ------------------------------------------------------
------ 0
   AF207712.1      ------------------------------------------------------
------ 0
   AY750628.1      ------------------------------------------------------
------ 0
   EF689084.1      ------------------------------------------------------
------ 0
   EF689085.1      ------------------------------------------------------
------ 0
   AB119070.1      ------------------------------------------------------
------ 956
   EF987742.1      ------------------------------------------------------
------ 0
   AB026105.1      ------------------------------------------------------
------ 0
   MW148603.1
AAGCTAAATCCCCCGAAACCAGACGAGCTACCTACGAACAATCCACAGGGATACACTCAT   1380
   AB051263.1      ------------------------------------------------------
------ 0
   AF068544.1      ------------------------------------------------------
------ 0


   JX982502.1      ------------------------------------------------------
------ 0
   JX982501.1      ------------------------------------------------------
------ 0
   JX982498.1      ------------------------------------------------------
------ 0
   JX982497.1      ------------------------------------------------------
------ 0
   JX982495.1      ------------------------------------------------------
------ 0
   JX982496.1      ------------------------------------------------------
------ 0
   JX982500.1      ------------------------------------------------------
------ 0
   EU548051.1      ------------------------------------------------------
------ 0
   EU548044.1      ------------------------------------------------------
------ 0
   EU548042.1      ------------------------------------------------------
------ 0
   EU548043.1      ------------------------------------------------------
------ 0
   EU548047.1      ------------------------------------------------------
------ 0
   EU548050.1      ------------------------------------------------------
------ 0
   EU548048.1      ------------------------------------------------------
------ 0
   EU548049.1      ------------------------------------------------------
------ 0
   AF207722.1      ------------------------------------------------------
------ 0
   EU548037.1      ------------------------------------------------------
------ 0
   AF207723.1      ------------------------------------------------------
------ 0
   EU548038.1      ------------------------------------------------------
------ 0
   EU548036.1      ------------------------------------------------------
------ 0
```

```
   EU548035.1      ------------------------------------------------------
------ 0
   AF207720.1      ------------------------------------------------------
------ 0
   AB601576.1      ------------------------------------------------------
------ 0
   EU548040.1      ------------------------------------------------------
------ 0
   EU548039.1      ------------------------------------------------------
------ 0
   AF207721.1      ------------------------------------------------------
------ 0
   AF207724.1      ------------------------------------------------------
------ 0
   JX982499.1      ------------------------------------------------------
------ 0
   EU548041.1      ------------------------------------------------------
------ 0
   EU548045.1      ------------------------------------------------------
------ 0
   AF207725.1      ------------------------------------------------------
------ 0
   EU548046.1      ------------------------------------------------------
------ 0
   AF207714.1      ------------------------------------------------------
------ 0
   AF207713.1      ------------------------------------------------------
------ 0
   AF207712.1      ------------------------------------------------------
------ 0
   AY750628.1      ------------------------------------------------------
------ 0
   EF689084.1      ------------------------------------------------------
------ 0
   EF689085.1      ------------------------------------------------------
------ 0
   AB119070.1      ------------------------------------------------------
------ 956
   EF987742.1      ------------------------------------------------------
------ 0
   AB026105.1      ------------------------------------------------------
------ 0
   MW148603.1
CTATGTCGCAAAATAGTGAGAAGATTCATAGGTAGAGGTGAAAAGCCTAACGAGCCTGGT   1440
   AB051263.1      ------------------------------------------------------
------ 0
   AF068544.1      ------------------------------------------------------
------ 0


   JX982502.1      ------------------------------------------------------
------ 0
   JX982501.1      ------------------------------------------------------
------ 0
   JX982498.1      ------------------------------------------------------
------ 0
   JX982497.1      ------------------------------------------------------
------ 0
   JX982495.1      ------------------------------------------------------
------ 0
   JX982496.1      ------------------------------------------------------
------ 0
   JX982500.1      ------------------------------------------------------
------ 0
   EU548051.1      ------------------------------------------------------
------ 0
```

```
   EU548044.1      ------------------------------------------------------
------ 0
   EU548042.1      ------------------------------------------------------
------ 0
   EU548043.1      ------------------------------------------------------
------ 0
   EU548047.1      ------------------------------------------------------
------ 0
   EU548050.1      ------------------------------------------------------
------ 0
   EU548048.1      ------------------------------------------------------
------ 0
   EU548049.1      ------------------------------------------------------
------ 0
   AF207722.1      ------------------------------------------------------
------ 0
   EU548037.1      ------------------------------------------------------
------ 0
   AF207723.1      ------------------------------------------------------
------ 0
   EU548038.1      ------------------------------------------------------
------ 0
   EU548036.1      ------------------------------------------------------
------ 0
   EU548035.1      ------------------------------------------------------
------ 0
   AF207720.1      ------------------------------------------------------
------ 0
   AB601576.1      ------------------------------------------------------
------ 0
   EU548040.1      ------------------------------------------------------
------ 0
   EU548039.1      ------------------------------------------------------
------ 0
   AF207721.1      ------------------------------------------------------
------ 0
   AF207724.1      ------------------------------------------------------
------ 0
   JX982499.1      ------------------------------------------------------
------ 0
   EU548041.1      ------------------------------------------------------
------ 0
   EU548045.1      ------------------------------------------------------
------ 0
   AF207725.1      ------------------------------------------------------
------ 0
   EU548046.1      ------------------------------------------------------
------ 0
   AF207714.1      ------------------------------------------------------
------ 0
   AF207713.1      ------------------------------------------------------
------ 0
   AF207712.1      ------------------------------------------------------
------ 0
   AY750628.1      ------------------------------------------------------
------ 0
   EF689084.1      ------------------------------------------------------
------ 0
   EF689085.1      ------------------------------------------------------
------ 0
   AB119070.1      ------------------------------------------------------
------ 956
   EF987742.1      ------------------------------------------------------
------ 0
   AB026105.1      ------------------------------------------------------
------ 0
```

```
    MW148603.1
GATAGCTGGTTGCCCAGAACAGAATCTCAGTTCAACTTTAAATTTACCTAATAACCCCCA   1500
    AB051263.1       ------------------------------------------------------
------ 0
    AF068544.1       ------------------------------------------------------
------ 0


    JX982502.1       ------------------------------------------------------
------ 0
    JX982501.1       ------------------------------------------------------
------ 0
    JX982498.1       ------------------------------------------------------
------ 0
    JX982497.1       ------------------------------------------------------
------ 0
    JX982495.1       ------------------------------------------------------
------ 0
    JX982496.1       ------------------------------------------------------
------ 0
    JX982500.1       ------------------------------------------------------
------ 0
    EU548051.1       ------------------------------------------------------
------ 0
    EU548044.1       ------------------------------------------------------
------ 0
    EU548042.1       ------------------------------------------------------
------ 0
    EU548043.1       ------------------------------------------------------
------ 0
    EU548047.1       ------------------------------------------------------
------ 0
    EU548050.1       ------------------------------------------------------
------ 0
    EU548048.1       ------------------------------------------------------
------ 0
    EU548049.1       ------------------------------------------------------
------ 0
    AF207722.1       ------------------------------------------------------
------ 0
    EU548037.1       ------------------------------------------------------
------ 0
    AF207723.1       ------------------------------------------------------
------ 0
    EU548038.1       ------------------------------------------------------
------ 0
    EU548036.1       ------------------------------------------------------
------ 0
    EU548035.1       ------------------------------------------------------
------ 0
    AF207720.1       ------------------------------------------------------
------ 0
    AB601576.1       ------------------------------------------------------
------ 0
    EU548040.1       ------------------------------------------------------
------ 0
    EU548039.1       ------------------------------------------------------
------ 0
    AF207721.1       ------------------------------------------------------
------ 0
    AF207724.1       ------------------------------------------------------
------ 0
    JX982499.1       ------------------------------------------------------
------ 0
    EU548041.1       ------------------------------------------------------
------ 0
```

EU548045.1 --
------ 0
AF207725.1 --
------ 0
EU548046.1 --
------ 0
AF207714.1 --
------ 0
AF207713.1 --
------ 0
AF207712.1 --
------ 0
AY750628.1 --
------ 0
EF689084.1 --
------ 0
EF689085.1 --
------ 0
AB119070.1 --
------ 956
EF987742.1 --
------ 0
AB026105.1 --
------ 0
MW148603.1
AATTGTAATGTAAATTTAAAATATAGTCTAAAAAGGTACAGCTTTTTAGAATAAGGATAC 1560
AB051263.1 --
------ 0
AF068544.1 --
------ 0

JX982502.1 --
------ 0
JX982501.1 --
------ 0
JX982498.1 --
------ 0
JX982497.1 --
------ 0
JX982495.1 --
------ 0
JX982496.1 --
------ 0
JX982500.1 --
------ 0
EU548051.1 --
------ 0
EU548044.1 --
------ 0
EU548042.1 --
------ 0
EU548043.1 --
------ 0
EU548047.1 --
------ 0
EU548050.1 --
------ 0
EU548048.1 --
------ 0
EU548049.1 --
------ 0
AF207722.1 --
------ 0
EU548037.1 --
------ 0

```
    AF207723.1      ------------------------------------------------------
------ 0
    EU548038.1      ------------------------------------------------------
------ 0
    EU548036.1      ------------------------------------------------------
------ 0
    EU548035.1      ------------------------------------------------------
------ 0
    AF207720.1      ------------------------------------------------------
------ 0
    AB601576.1      ------------------------------------------------------
------ 0
    EU548040.1      ------------------------------------------------------
------ 0
    EU548039.1      ------------------------------------------------------
------ 0
    AF207721.1      ------------------------------------------------------
------ 0
    AF207724.1      ------------------------------------------------------
------ 0
    JX982499.1      ------------------------------------------------------
------ 0
    EU548041.1      ------------------------------------------------------
------ 0
    EU548045.1      ------------------------------------------------------
------ 0
    AF207725.1      ------------------------------------------------------
------ 0
    EU548046.1      ------------------------------------------------------
------ 0
    AF207714.1      ------------------------------------------------------
------ 0
    AF207713.1      ------------------------------------------------------
------ 0
    AF207712.1      ------------------------------------------------------
------ 0
    AY750628.1      ------------------------------------------------------
------ 0
    EF689084.1      ------------------------------------------------------
------ 0
    EF689085.1      ------------------------------------------------------
------ 0
    AB119070.1      ------------------------------------------------------
------ 956
    EF987742.1      ------------------------------------------------------
------ 0
    AB026105.1      ------------------------------------------------------
------ 0
    MW148603.1
AACCTTGCTTAGAGAGTAAAATTAAACAAAACCATAGTAGGCCTAAGAGCAGCCACCAAT   1620
    AB051263.1      ------------------------------------------------------
------ 0
    AF068544.1      ------------------------------------------------------
------ 0


    JX982502.1      ------------------------------------------------------
------ 0
    JX982501.1      ------------------------------------------------------
------ 0
    JX982498.1      ------------------------------------------------------
------ 0
    JX982497.1      ------------------------------------------------------
------ 0
    JX982495.1      ------------------------------------------------------
------ 0
```

```
    JX982496.1          ------------------------------------------------------
------ 0
    JX982500.1          ------------------------------------------------------
------ 0
    EU548051.1          ------------------------------------------------------
------ 0
    EU548044.1          ------------------------------------------------------
------ 0
    EU548042.1          ------------------------------------------------------
------ 0
    EU548043.1          ------------------------------------------------------
------ 0
    EU548047.1          ------------------------------------------------------
------ 0
    EU548050.1          ------------------------------------------------------
------ 0
    EU548048.1          ------------------------------------------------------
------ 0
    EU548049.1          ------------------------------------------------------
------ 0
    AF207722.1          ------------------------------------------------------
------ 0
    EU548037.1          ------------------------------------------------------
------ 0
    AF207723.1          ------------------------------------------------------
------ 0
    EU548038.1          ------------------------------------------------------
------ 0
    EU548036.1          ------------------------------------------------------
------ 0
    EU548035.1          ------------------------------------------------------
------ 0
    AF207720.1          ------------------------------------------------------
------ 0
    AB601576.1          ------------------------------------------------------
------ 0
    EU548040.1          ------------------------------------------------------
------ 0
    EU548039.1          ------------------------------------------------------
------ 0
    AF207721.1          ------------------------------------------------------
------ 0
    AF207724.1          ------------------------------------------------------
------ 0
    JX982499.1          ------------------------------------------------------
------ 0
    EU548041.1          ------------------------------------------------------
------ 0
    EU548045.1          ------------------------------------------------------
------ 0
    AF207725.1          ------------------------------------------------------
------ 0
    EU548046.1          ------------------------------------------------------
------ 0
    AF207714.1          ------------------------------------------------------
------ 0
    AF207713.1          ------------------------------------------------------
------ 0
    AF207712.1          ------------------------------------------------------
------ 0
    AY750628.1          ------------------------------------------------------
------ 0
    EF689084.1          ------------------------------------------------------
------ 0
    EF689085.1          ------------------------------------------------------
------ 0
```

AB119070.1 --
------ 956
EF987742.1 --
------ 0
AB026105.1 --
------ 0
MW148603.1
TAAGAAAGCGTTCAAGCTCAACAATACAGCCACCTTAATCCCTATAATCATATACAACTC 1680
AB051263.1 --
------ 0
AF068544.1 --
------ 0

JX982502.1 --
------ 0
JX982501.1 --
------ 0
JX982498.1 --
------ 0
JX982497.1 --
------ 0
JX982495.1 --
------ 0
JX982496.1 --
------ 0
JX982500.1 --
------ 0
EU548051.1 --
------ 0
EU548044.1 --
------ 0
EU548042.1 --
------ 0
EU548043.1 --
------ 0
EU548047.1 --
------ 0
EU548050.1 --
------ 0
EU548048.1 --
------ 0
EU548049.1 --
------ 0
AF207722.1 --
------ 0
EU548037.1 --
------ 0
AF207723.1 --
------ 0
EU548038.1 --
------ 0
EU548036.1 --
------ 0
EU548035.1 --
------ 0
AF207720.1 --
------ 0
AB601576.1 --
------ 0
EU548040.1 --
------ 0
EU548039.1 --
------ 0
AF207721.1 --
------ 0

AF207724.1 --
------ 0
JX982499.1 --
------ 0
EU548041.1 --
------ 0
EU548045.1 --
------ 0
AF207725.1 --
------ 0
EU548046.1 --
------ 0
AF207714.1 --
------ 0
AF207713.1 --
------ 0
AF207712.1 --
------ 0
AY750628.1 --
------ 0
EF689084.1 --
------ 0
EF689085.1 --
------ 0
AB119070.1 --
------ 956
EF987742.1 --
------ 0
AB026105.1 --
------ 0
MW148603.1
CTAACACACTACTGGGCTAATCTATTTTATAATAGAAGCAATAATGCTAGTATGAGTAAC 1740
AB051263.1 --
------ 0
AF068544.1 --
------ 0

JX982502.1 --
------ 0
JX982501.1 --
------ 0
JX982498.1 --
------ 0
JX982497.1 --
------ 0
JX982495.1 --
------ 0
JX982496.1 --
------ 0
JX982500.1 --
------ 0
EU548051.1 --
------ 0
EU548044.1 --
------ 0
EU548042.1 --
------ 0
EU548043.1 --
------ 0
EU548047.1 --
------ 0
EU548050.1 --
------ 0
EU548048.1 --
------ 0

EU548049.1 --
------ 0
AF207722.1 --
------ 0
EU548037.1 --
------ 0
AF207723.1 --
------ 0
EU548038.1 --
------ 0
EU548036.1 --
------ 0
EU548035.1 --
------ 0
AF207720.1 --
------ 0
AB601576.1 --
------ 0
EU548040.1 --
------ 0
EU548039.1 --
------ 0
AF207721.1 --
------ 0
AF207724.1 --
------ 0
JX982499.1 --
------ 0
EU548041.1 --
------ 0
EU548045.1 --
------ 0
AF207725.1 --
------ 0
EU548046.1 --
------ 0
AF207714.1 --
------ 0
AF207713.1 --
------ 0
AF207712.1 --
------ 0
AY750628.1 --
------ 0
EF689084.1 --
------ 0
EF689085.1 --
------ 0
AB119070.1 --
------ 956
EF987742.1 --
------ 0
AB026105.1 --
------ 0
MW148603.1
AAGAAACATTTCTCCTTGCATAAGCTTATAACAGTTAACGAATACCCACTGATAGTTAAC 1800
AB051263.1 --
------ 0
AF068544.1 --
------ 0

JX982502.1 --
------ 0
JX982501.1 --
------ 0

```
    JX982498.1      ------------------------------------------------------
------ 0
    JX982497.1      ------------------------------------------------------
------ 0
    JX982495.1      ------------------------------------------------------
------ 0
    JX982496.1      ------------------------------------------------------
------ 0
    JX982500.1      ------------------------------------------------------
------ 0
    EU548051.1      ------------------------------------------------------
------ 0
    EU548044.1      ------------------------------------------------------
------ 0
    EU548042.1      ------------------------------------------------------
------ 0
    EU548043.1      ------------------------------------------------------
------ 0
    EU548047.1      ------------------------------------------------------
------ 0
    EU548050.1      ------------------------------------------------------
------ 0
    EU548048.1      ------------------------------------------------------
------ 0
    EU548049.1      ------------------------------------------------------
------ 0
    AF207722.1      ------------------------------------------------------
------ 0
    EU548037.1      ------------------------------------------------------
------ 0
    AF207723.1      ------------------------------------------------------
------ 0
    EU548038.1      ------------------------------------------------------
------ 0
    EU548036.1      ------------------------------------------------------
------ 0
    EU548035.1      ------------------------------------------------------
------ 0
    AF207720.1      ------------------------------------------------------
------ 0
    AB601576.1      ------------------------------------------------------
------ 0
    EU548040.1      ------------------------------------------------------
------ 0
    EU548039.1      ------------------------------------------------------
------ 0
    AF207721.1      ------------------------------------------------------
------ 0
    AF207724.1      ------------------------------------------------------
------ 0
    JX982499.1      ------------------------------------------------------
------ 0
    EU548041.1      ------------------------------------------------------
------ 0
    EU548045.1      ------------------------------------------------------
------ 0
    AF207725.1      ------------------------------------------------------
------ 0
    EU548046.1      ------------------------------------------------------
------ 0
    AF207714.1      ------------------------------------------------------
------ 0
    AF207713.1      ------------------------------------------------------
------ 0
    AF207712.1      ------------------------------------------------------
------ 0
```

```
   AY750628.1      ------------------------------------------------------
------ 0
   EF689084.1      ------------------------------------------------------
------ 0
   EF689085.1      ------------------------------------------------------
------ 0
   AB119070.1      ------------------------------------------------------
------ 956
   EF987742.1      ------------------------------------------------------
------ 0
   AB026105.1      ------------------------------------------------------
------ 0
   MW148603.1
AACAAGATAAAGATAAACCACTAATAAATATTCTTATCAAACCAATTGTTAGTCCAACAC   1860
   AB051263.1      ------------------------------------------------------
------ 0
   AF068544.1      ------------------------------------------------------
------ 0


   JX982502.1      ------------------------------------------------------
------ 0
   JX982501.1      ------------------------------------------------------
------ 0
   JX982498.1      ------------------------------------------------------
------ 0
   JX982497.1      ------------------------------------------------------
------ 0
   JX982495.1      ------------------------------------------------------
------ 0
   JX982496.1      ------------------------------------------------------
------ 0
   JX982500.1      ------------------------------------------------------
------ 0
   EU548051.1      ------------------------------------------------------
------ 0
   EU548044.1      ------------------------------------------------------
------ 0
   EU548042.1      ------------------------------------------------------
------ 0
   EU548043.1      ------------------------------------------------------
------ 0
   EU548047.1      ------------------------------------------------------
------ 0
   EU548050.1      ------------------------------------------------------
------ 0
   EU548048.1      ------------------------------------------------------
------ 0
   EU548049.1      ------------------------------------------------------
------ 0
   AF207722.1      ------------------------------------------------------
------ 0
   EU548037.1      ------------------------------------------------------
------ 0
   AF207723.1      ------------------------------------------------------
------ 0
   EU548038.1      ------------------------------------------------------
------ 0
   EU548036.1      ------------------------------------------------------
------ 0
   EU548035.1      ------------------------------------------------------
------ 0
   AF207720.1      ------------------------------------------------------
------ 0
   AB601576.1      ------------------------------------------------------
------ 0
```

```
    EU548040.1      ------------------------------------------------------
------ 0
    EU548039.1      ------------------------------------------------------
------ 0
    AF207721.1      ------------------------------------------------------
------ 0
    AF207724.1      ------------------------------------------------------
------ 0
    JX982499.1      ------------------------------------------------------
------ 0
    EU548041.1      ------------------------------------------------------
------ 0
    EU548045.1      ------------------------------------------------------
------ 0
    AF207725.1      ------------------------------------------------------
------ 0
    EU548046.1      ------------------------------------------------------
------ 0
    AF207714.1      ------------------------------------------------------
------ 0
    AF207713.1      ------------------------------------------------------
------ 0
    AF207712.1      ------------------------------------------------------
------ 0
    AY750628.1      ------------------------------------------------------
------ 0
    EF689084.1      ------------------------------------------------------
------ 0
    EF689085.1      ------------------------------------------------------
------ 0
    AB119070.1      ------------------------------------------------------
------ 956
    EF987742.1      ------------------------------------------------------
------ 0
    AB026105.1      ------------------------------------------------------
------ 0
    MW148603.1
AGGCATGCAACAAGGAAAGATTAAAAGAAGTAAAAGGAACTCGGCAAACTCAAACCCCGC   1920
    AB051263.1      ------------------------------------------------------
------ 0
    AF068544.1      ------------------------------------------------------
------ 0


    JX982502.1      ------------------------------------------------------
------ 0
    JX982501.1      ------------------------------------------------------
------ 0
    JX982498.1      ------------------------------------------------------
------ 0
    JX982497.1      ------------------------------------------------------
------ 0
    JX982495.1      ------------------------------------------------------
------ 0
    JX982496.1      ------------------------------------------------------
------ 0
    JX982500.1      ------------------------------------------------------
------ 0
    EU548051.1      ------------------------------------------------------
------ 0
    EU548044.1      ------------------------------------------------------
------ 0
    EU548042.1      ------------------------------------------------------
------ 0
    EU548043.1      ------------------------------------------------------
------ 0
```

```
   EU548047.1        ------------------------------------------------------
------ 0
   EU548050.1        ------------------------------------------------------
------ 0
   EU548048.1        ------------------------------------------------------
------ 0
   EU548049.1        ------------------------------------------------------
------ 0
   AF207722.1        ------------------------------------------------------
------ 0
   EU548037.1        ------------------------------------------------------
------ 0
   AF207723.1        ------------------------------------------------------
------ 0
   EU548038.1        ------------------------------------------------------
------ 0
   EU548036.1        ------------------------------------------------------
------ 0
   EU548035.1        ------------------------------------------------------
------ 0
   AF207720.1        ------------------------------------------------------
------ 0
   AB601576.1        ------------------------------------------------------
------ 0
   EU548040.1        ------------------------------------------------------
------ 0
   EU548039.1        ------------------------------------------------------
------ 0
   AF207721.1        ------------------------------------------------------
------ 0
   AF207724.1        ------------------------------------------------------
------ 0
   JX982499.1        ------------------------------------------------------
------ 0
   EU548041.1        ------------------------------------------------------
------ 0
   EU548045.1        ------------------------------------------------------
------ 0
   AF207725.1        ------------------------------------------------------
------ 0
   EU548046.1        ------------------------------------------------------
------ 0
   AF207714.1        ------------------------------------------------------
------ 0
   AF207713.1        ------------------------------------------------------
------ 0
   AF207712.1        ------------------------------------------------------
------ 0
   AY750628.1        ------------------------------------------------------
------ 0
   EF689084.1        ------------------------------------------------------
------ 0
   EF689085.1        ------------------------------------------------------
------ 0
   AB119070.1        ------------------------------------------------------
------ 956
   EF987742.1        ------------------------------------------------------
------ 0
   AB026105.1        ------------------------------------------------------
------ 0
   MW148603.1
CTGTTTACCAAAAACATCACCTCCAGCATATCTAGTATTGGAGGCACTGCCTGCCCAGTG   1980
   AB051263.1        ------------------------------------------------------
------ 0
   AF068544.1        ------------------------------------------------------
------ 0
```

JX982502.1 --
------ 0
JX982501.1 --
------ 0
JX982498.1 --
------ 0
JX982497.1 --
------ 0
JX982495.1 --
------ 0
JX982496.1 --
------ 0
JX982500.1 --
------ 0
EU548051.1 --
------ 0
EU548044.1 --
------ 0
EU548042.1 --
------ 0
EU548043.1 --
------ 0
EU548047.1 --
------ 0
EU548050.1 --
------ 0
EU548048.1 --
------ 0
EU548049.1 --
------ 0
AF207722.1 --
------ 0
EU548037.1 --
------ 0
AF207723.1 --
------ 0
EU548038.1 --
------ 0
EU548036.1 --
------ 0
EU548035.1 --
------ 0
AF207720.1 --
------ 0
AB601576.1 --
------ 0
EU548040.1 --
------ 0
EU548039.1 --
------ 0
AF207721.1 --
------ 0
AF207724.1 --
------ 0
JX982499.1 --
------ 0
EU548041.1 --
------ 0
EU548045.1 --
------ 0
AF207725.1 --
------ 0
EU548046.1 --
------ 0

```
    AF207714.1      ------------------------------------------------------
------ 0
    AF207713.1      ------------------------------------------------------
------ 0
    AF207712.1      ------------------------------------------------------
------ 0
    AY750628.1      ------------------------------------------------------
------ 0
    EF689084.1      ------------------------------------------------------
------ 0
    EF689085.1      ------------------------------------------------------
------ 0
    AB119070.1      ------------------------------------------------------
------ 956
    EF987742.1      ------------------------------------------------------
------ 0
    AB026105.1      ------------------------------------------------------
------ 0
    MW148603.1
ACACTAGTTTAACGGCCGCGGTATCCTGACCGTGCAAAGGTAGCATAATCATTTGTTCTC   2040
    AB051263.1      ------------------------------------------------------
------ 0
    AF068544.1      ------------------------------------------------------
------ 0

    JX982502.1      ------------------------------------------------------
------ 0
    JX982501.1      ------------------------------------------------------
------ 0
    JX982498.1      ------------------------------------------------------
------ 0
    JX982497.1      ------------------------------------------------------
------ 0
    JX982495.1      ------------------------------------------------------
------ 0
    JX982496.1      ------------------------------------------------------
------ 0
    JX982500.1      ------------------------------------------------------
------ 0
    EU548051.1      ------------------------------------------------------
------ 0
    EU548044.1      ------------------------------------------------------
------ 0
    EU548042.1      ------------------------------------------------------
------ 0
    EU548043.1      ------------------------------------------------------
------ 0
    EU548047.1      ------------------------------------------------------
------ 0
    EU548050.1      ------------------------------------------------------
------ 0
    EU548048.1      ------------------------------------------------------
------ 0
    EU548049.1      ------------------------------------------------------
------ 0
    AF207722.1      ------------------------------------------------------
------ 0
    EU548037.1      ------------------------------------------------------
------ 0
    AF207723.1      ------------------------------------------------------
------ 0
    EU548038.1      ------------------------------------------------------
------ 0
    EU548036.1      ------------------------------------------------------
------ 0
```

```
   EU548035.1      ------------------------------------------------------
------ 0
   AF207720.1      ------------------------------------------------------
------ 0
   AB601576.1      ------------------------------------------------------
------ 0
   EU548040.1      ------------------------------------------------------
------ 0
   EU548039.1      ------------------------------------------------------
------ 0
   AF207721.1      ------------------------------------------------------
------ 0
   AF207724.1      ------------------------------------------------------
------ 0
   JX982499.1      ------------------------------------------------------
------ 0
   EU548041.1      ------------------------------------------------------
------ 0
   EU548045.1      ------------------------------------------------------
------ 0
   AF207725.1      ------------------------------------------------------
------ 0
   EU548046.1      ------------------------------------------------------
------ 0
   AF207714.1      ------------------------------------------------------
------ 0
   AF207713.1      ------------------------------------------------------
------ 0
   AF207712.1      ------------------------------------------------------
------ 0
   AY750628.1      ------------------------------------------------------
------ 0
   EF689084.1      ------------------------------------------------------
------ 0
   EF689085.1      ------------------------------------------------------
------ 0
   AB119070.1      ------------------------------------------------------
------ 956
   EF987742.1      ------------------------------------------------------
------ 0
   AB026105.1      ------------------------------------------------------
------ 0
   MW148603.1
TAAATAGGGACTTGTATGAATGGCCACACGAGGGTTTAACTGTCTCTTACTTCCAATCAG   2100
   AB051263.1      ------------------------------------------------------
------ 0
   AF068544.1      ------------------------------------------------------
------ 0


   JX982502.1      ------------------------------------------------------
------ 0
   JX982501.1      ------------------------------------------------------
------ 0
   JX982498.1      ------------------------------------------------------
------ 0
   JX982497.1      ------------------------------------------------------
------ 0
   JX982495.1      ------------------------------------------------------
------ 0
   JX982496.1      ------------------------------------------------------
------ 0
   JX982500.1      ------------------------------------------------------
------ 0
   EU548051.1      ------------------------------------------------------
------ 0
```

```
   EU548044.1      ------------------------------------------------------
------ 0
   EU548042.1      ------------------------------------------------------
------ 0
   EU548043.1      ------------------------------------------------------
------ 0
   EU548047.1      ------------------------------------------------------
------ 0
   EU548050.1      ------------------------------------------------------
------ 0
   EU548048.1      ------------------------------------------------------
------ 0
   EU548049.1      ------------------------------------------------------
------ 0
   AF207722.1      ------------------------------------------------------
------ 0
   EU548037.1      ------------------------------------------------------
------ 0
   AF207723.1      ------------------------------------------------------
------ 0
   EU548038.1      ------------------------------------------------------
------ 0
   EU548036.1      ------------------------------------------------------
------ 0
   EU548035.1      ------------------------------------------------------
------ 0
   AF207720.1      ------------------------------------------------------
------ 0
   AB601576.1      ------------------------------------------------------
------ 0
   EU548040.1      ------------------------------------------------------
------ 0
   EU548039.1      ------------------------------------------------------
------ 0
   AF207721.1      ------------------------------------------------------
------ 0
   AF207724.1      ------------------------------------------------------
------ 0
   JX982499.1      ------------------------------------------------------
------ 0
   EU548041.1      ------------------------------------------------------
------ 0
   EU548045.1      ------------------------------------------------------
------ 0
   AF207725.1      ------------------------------------------------------
------ 0
   EU548046.1      ------------------------------------------------------
------ 0
   AF207714.1      ------------------------------------------------------
------ 0
   AF207713.1      ------------------------------------------------------
------ 0
   AF207712.1      ------------------------------------------------------
------ 0
   AY750628.1      ------------------------------------------------------
------ 0
   EF689084.1      ------------------------------------------------------
------ 0
   EF689085.1      ------------------------------------------------------
------ 0
   AB119070.1      ------------------------------------------------------
------ 956
   EF987742.1      ------------------------------------------------------
------ 0
   AB026105.1      ------------------------------------------------------
------ 0
```

```
    MW148603.1
TGAAATTGACCTTCCCGTGAAGAGGCGGGAATATACCAATAAGACGAGAAGACCCTATGG   2160
    AB051263.1      ------------------------------------------------------
------ 0
    AF068544.1      ------------------------------------------------------
------ 0


    JX982502.1      ------------------------------------------------------
------ 0
    JX982501.1      ------------------------------------------------------
------ 0
    JX982498.1      ------------------------------------------------------
------ 0
    JX982497.1      ------------------------------------------------------
------ 0
    JX982495.1      ------------------------------------------------------
------ 0
    JX982496.1      ------------------------------------------------------
------ 0
    JX982500.1      ------------------------------------------------------
------ 0
    EU548051.1      ------------------------------------------------------
------ 0
    EU548044.1      ------------------------------------------------------
------ 0
    EU548042.1      ------------------------------------------------------
------ 0
    EU548043.1      ------------------------------------------------------
------ 0
    EU548047.1      ------------------------------------------------------
------ 0
    EU548050.1      ------------------------------------------------------
------ 0
    EU548048.1      ------------------------------------------------------
------ 0
    EU548049.1      ------------------------------------------------------
------ 0
    AF207722.1      ------------------------------------------------------
------ 0
    EU548037.1      ------------------------------------------------------
------ 0
    AF207723.1      ------------------------------------------------------
------ 0
    EU548038.1      ------------------------------------------------------
------ 0
    EU548036.1      ------------------------------------------------------
------ 0
    EU548035.1      ------------------------------------------------------
------ 0
    AF207720.1      ------------------------------------------------------
------ 0
    AB601576.1      ------------------------------------------------------
------ 0
    EU548040.1      ------------------------------------------------------
------ 0
    EU548039.1      ------------------------------------------------------
------ 0
    AF207721.1      ------------------------------------------------------
------ 0
    AF207724.1      ------------------------------------------------------
------ 0
    JX982499.1      ------------------------------------------------------
------ 0
    EU548041.1      ------------------------------------------------------
------ 0
```

EU548045.1 --
------ 0
AF207725.1 --
------ 0
EU548046.1 --
------ 0
AF207714.1 --
------ 0
AF207713.1 --
------ 0
AF207712.1 --
------ 0
AY750628.1 --
------ 0
EF689084.1 --
------ 0
EF689085.1 --
------ 0
AB119070.1 --
------ 956
EF987742.1 --
------ 0
AB026105.1 --
------ 0
MW148603.1
AGCTTCAATTAACTAACCCACAATAACCAATCAATATGCCAACCAGGCCTAACATAATCT 2220
AB051263.1 --
------ 0
AF068544.1 --
------ 0

JX982502.1 --
------ 0
JX982501.1 --
------ 0
JX982498.1 --
------ 0
JX982497.1 --
------ 0
JX982495.1 --
------ 0
JX982496.1 --
------ 0
JX982500.1 --
------ 0
EU548051.1 --
------ 0
EU548044.1 --
------ 0
EU548042.1 --
------ 0
EU548043.1 --
------ 0
EU548047.1 --
------ 0
EU548050.1 --
------ 0
EU548048.1 --
------ 0
EU548049.1 --
------ 0
AF207722.1 --
------ 0
EU548037.1 --
------ 0

```
   AF207723.1      ------------------------------------------------------
------ 0
   EU548038.1      ------------------------------------------------------
------ 0
   EU548036.1      ------------------------------------------------------
------ 0
   EU548035.1      ------------------------------------------------------
------ 0
   AF207720.1      ------------------------------------------------------
------ 0
   AB601576.1      ------------------------------------------------------
------ 0
   EU548040.1      ------------------------------------------------------
------ 0
   EU548039.1      ------------------------------------------------------
------ 0
   AF207721.1      ------------------------------------------------------
------ 0
   AF207724.1      ------------------------------------------------------
------ 0
   JX982499.1      ------------------------------------------------------
------ 0
   EU548041.1      ------------------------------------------------------
------ 0
   EU548045.1      ------------------------------------------------------
------ 0
   AF207725.1      ------------------------------------------------------
------ 0
   EU548046.1      ------------------------------------------------------
------ 0
   AF207714.1      ------------------------------------------------------
------ 0
   AF207713.1      ------------------------------------------------------
------ 0
   AF207712.1      ------------------------------------------------------
------ 0
   AY750628.1      ------------------------------------------------------
------ 0
   EF689084.1      ------------------------------------------------------
------ 0
   EF689085.1      ------------------------------------------------------
------ 0
   AB119070.1      ------------------------------------------------------
------ 956
   EF987742.1      ------------------------------------------------------
------ 0
   AB026105.1      ------------------------------------------------------
------ 0
   MW148603.1
TATTTCTGGGTTAGCAGTTTAGGTTGGGGTGACCTCGGAGAATAAAATAACCTCCGAGTG   2280
   AB051263.1      ------------------------------------------------------
------ 0
   AF068544.1      ------------------------------------------------------
------ 0


   JX982502.1      ------------------------------------------------------
------ 0
   JX982501.1      ------------------------------------------------------
------ 0
   JX982498.1      ------------------------------------------------------
------ 0
   JX982497.1      ------------------------------------------------------
------ 0
   JX982495.1      ------------------------------------------------------
------ 0
```

```
    JX982496.1          -------------------------------------------------------
------ 0
    JX982500.1          -------------------------------------------------------
------ 0
    EU548051.1          -------------------------------------------------------
------ 0
    EU548044.1          -------------------------------------------------------
------ 0
    EU548042.1          -------------------------------------------------------
------ 0
    EU548043.1          -------------------------------------------------------
------ 0
    EU548047.1          -------------------------------------------------------
------ 0
    EU548050.1          -------------------------------------------------------
------ 0
    EU548048.1          -------------------------------------------------------
------ 0
    EU548049.1          -------------------------------------------------------
------ 0
    AF207722.1          -------------------------------------------------------
------ 0
    EU548037.1          -------------------------------------------------------
------ 0
    AF207723.1          -------------------------------------------------------
------ 0
    EU548038.1          -------------------------------------------------------
------ 0
    EU548036.1          -------------------------------------------------------
------ 0
    EU548035.1          -------------------------------------------------------
------ 0
    AF207720.1          -------------------------------------------------------
------ 0
    AB601576.1          -------------------------------------------------------
------ 0
    EU548040.1          -------------------------------------------------------
------ 0
    EU548039.1          -------------------------------------------------------
------ 0
    AF207721.1          -------------------------------------------------------
------ 0
    AF207724.1          -------------------------------------------------------
------ 0
    JX982499.1          -------------------------------------------------------
------ 0
    EU548041.1          -------------------------------------------------------
------ 0
    EU548045.1          -------------------------------------------------------
------ 0
    AF207725.1          -------------------------------------------------------
------ 0
    EU548046.1          -------------------------------------------------------
------ 0
    AF207714.1          -------------------------------------------------------
------ 0
    AF207713.1          -------------------------------------------------------
------ 0
    AF207712.1          -------------------------------------------------------
------ 0
    AY750628.1          -------------------------------------------------------
------ 0
    EF689084.1          -------------------------------------------------------
------ 0
    EF689085.1          -------------------------------------------------------
------ 0
```

```
    AB119070.1      ------------------------------------------------------
------ 956
    EF987742.1      ------------------------------------------------------
------ 0
    AB026105.1      ------------------------------------------------------
------ 0
    MW148603.1
ATTTAATCTGAGACAAACCAGTCGAAGCGTCCTATCATTAATTGATCCAATAATTTGATC   2340
    AB051263.1      ------------------------------------------------------
------ 0
    AF068544.1      ------------------------------------------------------
------ 0


    JX982502.1      ------------------------------------------------------
------ 0
    JX982501.1      ------------------------------------------------------
------ 0
    JX982498.1      ------------------------------------------------------
------ 0
    JX982497.1      ------------------------------------------------------
------ 0
    JX982495.1      ------------------------------------------------------
------ 0
    JX982496.1      ------------------------------------------------------
------ 0
    JX982500.1      ------------------------------------------------------
------ 0
    EU548051.1      ------------------------------------------------------
------ 0
    EU548044.1      ------------------------------------------------------
------ 0
    EU548042.1      ------------------------------------------------------
------ 0
    EU548043.1      ------------------------------------------------------
------ 0
    EU548047.1      ------------------------------------------------------
------ 0
    EU548050.1      ------------------------------------------------------
------ 0
    EU548048.1      ------------------------------------------------------
------ 0
    EU548049.1      ------------------------------------------------------
------ 0
    AF207722.1      ------------------------------------------------------
------ 0
    EU548037.1      ------------------------------------------------------
------ 0
    AF207723.1      ------------------------------------------------------
------ 0
    EU548038.1      ------------------------------------------------------
------ 0
    EU548036.1      ------------------------------------------------------
------ 0
    EU548035.1      ------------------------------------------------------
------ 0
    AF207720.1      ------------------------------------------------------
------ 0
    AB601576.1      ------------------------------------------------------
------ 0
    EU548040.1      ------------------------------------------------------
------ 0
    EU548039.1      ------------------------------------------------------
------ 0
    AF207721.1      ------------------------------------------------------
------ 0
```

AF207724.1 --
------ 0
JX982499.1 --
------ 0
EU548041.1 --
------ 0
EU548045.1 --
------ 0
AF207725.1 --
------ 0
EU548046.1 --
------ 0
AF207714.1 --
------ 0
AF207713.1 --
------ 0
AF207712.1 --
------ 0
AY750628.1 --
------ 0
EF689084.1 --
------ 0
EF689085.1 --
------ 0
AB119070.1 --
------ 956
EF987742.1 --
------ 0
AB026105.1 --
------ 0
MW148603.1
AACGGAACAAGTTACCCTAGGGATAACAGCGCAATCCTATTTGAGAGTCCATATCAACAA 2400
AB051263.1 --
------ 0
AF068544.1 --
------ 0

JX982502.1 --
------ 0
JX982501.1 --
------ 0
JX982498.1 --
------ 0
JX982497.1 --
------ 0
JX982495.1 --
------ 0
JX982496.1 --
------ 0
JX982500.1 --
------ 0
EU548051.1 --
------ 0
EU548044.1 --
------ 0
EU548042.1 --
------ 0
EU548043.1 --
------ 0
EU548047.1 --
------ 0
EU548050.1 --
------ 0
EU548048.1 --
------ 0

```
   EU548049.1       ------------------------------------------------------
------ 0
   AF207722.1       ------------------------------------------------------
------ 0
   EU548037.1       ------------------------------------------------------
------ 0
   AF207723.1       ------------------------------------------------------
------ 0
   EU548038.1       ------------------------------------------------------
------ 0
   EU548036.1       ------------------------------------------------------
------ 0
   EU548035.1       ------------------------------------------------------
------ 0
   AF207720.1       ------------------------------------------------------
------ 0
   AB601576.1       ------------------------------------------------------
------ 0
   EU548040.1       ------------------------------------------------------
------ 0
   EU548039.1       ------------------------------------------------------
------ 0
   AF207721.1       ------------------------------------------------------
------ 0
   AF207724.1       ------------------------------------------------------
------ 0
   JX982499.1       ------------------------------------------------------
------ 0
   EU548041.1       ------------------------------------------------------
------ 0
   EU548045.1       ------------------------------------------------------
------ 0
   AF207725.1       ------------------------------------------------------
------ 0
   EU548046.1       ------------------------------------------------------
------ 0
   AF207714.1       ------------------------------------------------------
------ 0
   AF207713.1       ------------------------------------------------------
------ 0
   AF207712.1       ------------------------------------------------------
------ 0
   AY750628.1       ------------------------------------------------------
------ 0
   EF689084.1       ------------------------------------------------------
------ 0
   EF689085.1       ------------------------------------------------------
------ 0
   AB119070.1       ------------------------------------------------------
------ 956
   EF987742.1       ------------------------------------------------------
------ 0
   AB026105.1       ------------------------------------------------------
------ 0
   MW148603.1
TAGGGTTTACGACCTCGATGTTGGATCAGGACATCCTAATGGTGCAGCAGCTATTAATGG   2460
   AB051263.1       ------------------------------------------------------
------ 0
   AF068544.1       ------------------------------------------------------
------ 0


   JX982502.1       ------------------------------------------------------
------ 0
   JX982501.1       ------------------------------------------------------
------ 0
```

JX982498.1 --
------ 0
JX982497.1 --
------ 0
JX982495.1 --
------ 0
JX982496.1 --
------ 0
JX982500.1 --
------ 0
EU548051.1 --
------ 0
EU548044.1 --
------ 0
EU548042.1 --
------ 0
EU548043.1 --
------ 0
EU548047.1 --
------ 0
EU548050.1 --
------ 0
EU548048.1 --
------ 0
EU548049.1 --
------ 0
AF207722.1 --
------ 0
EU548037.1 --
------ 0
AF207723.1 --
------ 0
EU548038.1 --
------ 0
EU548036.1 --
------ 0
EU548035.1 --
------ 0
AF207720.1 --
------ 0
AB601576.1 --
------ 0
EU548040.1 --
------ 0
EU548039.1 --
------ 0
AF207721.1 --
------ 0
AF207724.1 --
------ 0
JX982499.1 --
------ 0
EU548041.1 --
------ 0
EU548045.1 --
------ 0
AF207725.1 --
------ 0
EU548046.1 --
------ 0
AF207714.1 --
------ 0
AF207713.1 --
------ 0
AF207712.1 --
------ 0

```
   AY750628.1      ------------------------------------------------------
------ 0
   EF689084.1      ------------------------------------------------------
------ 0
   EF689085.1      ------------------------------------------------------
------ 0
   AB119070.1      ------------------------------------------------------
------ 956
   EF987742.1      ------------------------------------------------------
------ 0
   AB026105.1      ------------------------------------------------------
------ 0
   MW148603.1
TTCGTTTGTTCAACGATTAAAGTCCTACGTGATCTGAGTTCAGACCGGAGCAATCCAGGT   2520
   AB051263.1      ------------------------------------------------------
------ 0
   AF068544.1      ------------------------------------------------------
------ 0


   JX982502.1      ------------------------------------------------------
------ 0
   JX982501.1      ------------------------------------------------------
------ 0
   JX982498.1      ------------------------------------------------------
------ 0
   JX982497.1      ------------------------------------------------------
------ 0
   JX982495.1      ------------------------------------------------------
------ 0
   JX982496.1      ------------------------------------------------------
------ 0
   JX982500.1      ------------------------------------------------------
------ 0
   EU548051.1      ------------------------------------------------------
------ 0
   EU548044.1      ------------------------------------------------------
------ 0
   EU548042.1      ------------------------------------------------------
------ 0
   EU548043.1      ------------------------------------------------------
------ 0
   EU548047.1      ------------------------------------------------------
------ 0
   EU548050.1      ------------------------------------------------------
------ 0
   EU548048.1      ------------------------------------------------------
------ 0
   EU548049.1      ------------------------------------------------------
------ 0
   AF207722.1      ------------------------------------------------------
------ 0
   EU548037.1      ------------------------------------------------------
------ 0
   AF207723.1      ------------------------------------------------------
------ 0
   EU548038.1      ------------------------------------------------------
------ 0
   EU548036.1      ------------------------------------------------------
------ 0
   EU548035.1      ------------------------------------------------------
------ 0
   AF207720.1      ------------------------------------------------------
------ 0
   AB601576.1      ------------------------------------------------------
------ 0
```

```
    EU548040.1       ------------------------------------------------------
------ 0
    EU548039.1       ------------------------------------------------------
------ 0
    AF207721.1       ------------------------------------------------------
------ 0
    AF207724.1       ------------------------------------------------------
------ 0
    JX982499.1       ------------------------------------------------------
------ 0
    EU548041.1       ------------------------------------------------------
------ 0
    EU548045.1       ------------------------------------------------------
------ 0
    AF207725.1       ------------------------------------------------------
------ 0
    EU548046.1       ------------------------------------------------------
------ 0
    AF207714.1       ------------------------------------------------------
------ 0
    AF207713.1       ------------------------------------------------------
------ 0
    AF207712.1       ------------------------------------------------------
------ 0
    AY750628.1       ------------------------------------------------------
------ 0
    EF689084.1       ------------------------------------------------------
------ 0
    EF689085.1       ------------------------------------------------------
------ 0
    AB119070.1       ------------------------------------------------------
------ 956
    EF987742.1       ------------------------------------------------------
------ 0
    AB026105.1       ------------------------------------------------------
------ 0
    MW148603.1
CGGTTTCTATCTATTATAATTACTTCTCCCAGTACGAAAGGACAAGAGAAGTAGGGCCTA   2580
    AB051263.1       ------------------------------------------------------
------ 0
    AF068544.1       ------------------------------------------------------
------ 0


    JX982502.1       ------------------------------------------------------
------ 0
    JX982501.1       ------------------------------------------------------
------ 0
    JX982498.1       ------------------------------------------------------
------ 0
    JX982497.1       ------------------------------------------------------
------ 0
    JX982495.1       ------------------------------------------------------
------ 0
    JX982496.1       ------------------------------------------------------
------ 0
    JX982500.1       ------------------------------------------------------
------ 0
    EU548051.1       ------------------------------------------------------
------ 0
    EU548044.1       ------------------------------------------------------
------ 0
    EU548042.1       ------------------------------------------------------
------ 0
    EU548043.1       ------------------------------------------------------
------ 0
```

EU548047.1 --
------ 0
EU548050.1 --
------ 0
EU548048.1 --
------ 0
EU548049.1 --
------ 0
AF207722.1 --
------ 0
EU548037.1 --
------ 0
AF207723.1 --
------ 0
EU548038.1 --
------ 0
EU548036.1 --
------ 0
EU548035.1 --
------ 0
AF207720.1 --
------ 0
AB601576.1 --
------ 0
EU548040.1 --
------ 0
EU548039.1 --
------ 0
AF207721.1 --
------ 0
AF207724.1 --
------ 0
JX982499.1 --
------ 0
EU548041.1 --
------ 0
EU548045.1 --
------ 0
AF207725.1 --
------ 0
EU548046.1 --
------ 0
AF207714.1 --
------ 0
AF207713.1 --
------ 0
AF207712.1 --
------ 0
AY750628.1 --
------ 0
EF689084.1 --
------ 0
EF689085.1 --
------ 0
AB119070.1 --
------ 956
EF987742.1 --
------ 0
AB026105.1 --
------ 0
MW148603.1
TTCTACAGGAAAGCCTTAGGACTAATAGATGATATAATCTCAATCTAACCAGTCCACTCC 2640
AB051263.1 --
------ 0
AF068544.1 --
------ 0

```
    JX982502.1      ------------------------------------------------------
------ 0
    JX982501.1      ------------------------------------------------------
------ 0
    JX982498.1      ------------------------------------------------------
------ 0
    JX982497.1      ------------------------------------------------------
------ 0
    JX982495.1      ------------------------------------------------------
------ 0
    JX982496.1      ------------------------------------------------------
------ 0
    JX982500.1      ------------------------------------------------------
------ 0
    EU548051.1      ------------------------------------------------------
------ 0
    EU548044.1      ------------------------------------------------------
------ 0
    EU548042.1      ------------------------------------------------------
------ 0
    EU548043.1      ------------------------------------------------------
------ 0
    EU548047.1      ------------------------------------------------------
------ 0
    EU548050.1      ------------------------------------------------------
------ 0
    EU548048.1      ------------------------------------------------------
------ 0
    EU548049.1      ------------------------------------------------------
------ 0
    AF207722.1      ------------------------------------------------------
------ 0
    EU548037.1      ------------------------------------------------------
------ 0
    AF207723.1      ------------------------------------------------------
------ 0
    EU548038.1      ------------------------------------------------------
------ 0
    EU548036.1      ------------------------------------------------------
------ 0
    EU548035.1      ------------------------------------------------------
------ 0
    AF207720.1      ------------------------------------------------------
------ 0
    AB601576.1      ------------------------------------------------------
------ 0
    EU548040.1      ------------------------------------------------------
------ 0
    EU548039.1      ------------------------------------------------------
------ 0
    AF207721.1      ------------------------------------------------------
------ 0
    AF207724.1      ------------------------------------------------------
------ 0
    JX982499.1      ------------------------------------------------------
------ 0
    EU548041.1      ------------------------------------------------------
------ 0
    EU548045.1      ------------------------------------------------------
------ 0
    AF207725.1      ------------------------------------------------------
------ 0
    EU548046.1      ------------------------------------------------------
------ 0
```

```
    AF207714.1       ------------------------------------------------------
------ 0
    AF207713.1       ------------------------------------------------------
------ 0
    AF207712.1       ------------------------------------------------------
------ 0
    AY750628.1       ------------------------------------------------------
------ 0
    EF689084.1       ------------------------------------------------------
------ 0
    EF689085.1       ------------------------------------------------------
------ 0
    AB119070.1       ------------------------------------------------------
------ 956
    EF987742.1       ------------------------------------------------------
------ 0
    AB026105.1       ------------------------------------------------------
------ 0
    MW148603.1
CCCCATAACCCTAGAAATAGGGTTTGTTAGGGTGGCAGAGCCCAGTAATTGCGTAAAACT   2700
    AB051263.1       ------------------------------------------------------
------ 0
    AF068544.1       ------------------------------------------------------
------ 0


    JX982502.1       ------------------------------------------------------
------ 0
    JX982501.1       ------------------------------------------------------
------ 0
    JX982498.1       ------------------------------------------------------
------ 0
    JX982497.1       ------------------------------------------------------
------ 0
    JX982495.1       ------------------------------------------------------
------ 0
    JX982496.1       ------------------------------------------------------
------ 0
    JX982500.1       ------------------------------------------------------
------ 0
    EU548051.1       ------------------------------------------------------
------ 0
    EU548044.1       ------------------------------------------------------
------ 0
    EU548042.1       ------------------------------------------------------
------ 0
    EU548043.1       ------------------------------------------------------
------ 0
    EU548047.1       ------------------------------------------------------
------ 0
    EU548050.1       ------------------------------------------------------
------ 0
    EU548048.1       ------------------------------------------------------
------ 0
    EU548049.1       ------------------------------------------------------
------ 0
    AF207722.1       ------------------------------------------------------
------ 0
    EU548037.1       ------------------------------------------------------
------ 0
    AF207723.1       ------------------------------------------------------
------ 0
    EU548038.1       ------------------------------------------------------
------ 0
    EU548036.1       ------------------------------------------------------
------ 0
```

```
   EU548035.1      ------------------------------------------------------
------ 0
   AF207720.1      ------------------------------------------------------
------ 0
   AB601576.1      ------------------------------------------------------
------ 0
   EU548040.1      ------------------------------------------------------
------ 0
   EU548039.1      ------------------------------------------------------
------ 0
   AF207721.1      ------------------------------------------------------
------ 0
   AF207724.1      ------------------------------------------------------
------ 0
   JX982499.1      ------------------------------------------------------
------ 0
   EU548041.1      ------------------------------------------------------
------ 0
   EU548045.1      ------------------------------------------------------
------ 0
   AF207725.1      ------------------------------------------------------
------ 0
   EU548046.1      ------------------------------------------------------
------ 0
   AF207714.1      ------------------------------------------------------
------ 0
   AF207713.1      ------------------------------------------------------
------ 0
   AF207712.1      ------------------------------------------------------
------ 0
   AY750628.1      ------------------------------------------------------
------ 0
   EF689084.1      ------------------------------------------------------
------ 0
   EF689085.1      ------------------------------------------------------
------ 0
   AB119070.1      ------------------------------------------------------
------ 956
   EF987742.1      ------------------------------------------------------
------ 0
   AB026105.1      ------------------------------------------------------
------ 0
   MW148603.1
TAAACCTTTATTACCAGAGGTTCAAATCCTCTCCCTAACATCATGTTTATAATTAACATT   2760
   AB051263.1      ------------------------------------------------------
------ 0
   AF068544.1      ------------------------------------------------------
------ 0


   JX982502.1      ------------------------------------------------------
------ 0
   JX982501.1      ------------------------------------------------------
------ 0
   JX982498.1      ------------------------------------------------------
------ 0
   JX982497.1      ------------------------------------------------------
------ 0
   JX982495.1      ------------------------------------------------------
------ 0
   JX982496.1      ------------------------------------------------------
------ 0
   JX982500.1      ------------------------------------------------------
------ 0
   EU548051.1      ------------------------------------------------------
------ 0
```

```
   EU548044.1       ------------------------------------------------------
------ 0
   EU548042.1       ------------------------------------------------------
------ 0
   EU548043.1       ------------------------------------------------------
------ 0
   EU548047.1       ------------------------------------------------------
------ 0
   EU548050.1       ------------------------------------------------------
------ 0
   EU548048.1       ------------------------------------------------------
------ 0
   EU548049.1       ------------------------------------------------------
------ 0
   AF207722.1       ------------------------------------------------------
------ 0
   EU548037.1       ------------------------------------------------------
------ 0
   AF207723.1       ------------------------------------------------------
------ 0
   EU548038.1       ------------------------------------------------------
------ 0
   EU548036.1       ------------------------------------------------------
------ 0
   EU548035.1       ------------------------------------------------------
------ 0
   AF207720.1       ------------------------------------------------------
------ 0
   AB601576.1       ------------------------------------------------------
------ 0
   EU548040.1       ------------------------------------------------------
------ 0
   EU548039.1       ------------------------------------------------------
------ 0
   AF207721.1       ------------------------------------------------------
------ 0
   AF207724.1       ------------------------------------------------------
------ 0
   JX982499.1       ------------------------------------------------------
------ 0
   EU548041.1       ------------------------------------------------------
------ 0
   EU548045.1       ------------------------------------------------------
------ 0
   AF207725.1       ------------------------------------------------------
------ 0
   EU548046.1       ------------------------------------------------------
------ 0
   AF207714.1       ------------------------------------------------------
------ 0
   AF207713.1       ------------------------------------------------------
------ 0
   AF207712.1       ------------------------------------------------------
------ 0
   AY750628.1       ------------------------------------------------------
------ 0
   EF689084.1       ------------------------------------------------------
------ 0
   EF689085.1       ------------------------------------------------------
------ 0
   AB119070.1       ------------------------------------------------------
------ 956
   EF987742.1       ------------------------------------------------------
------ 0
   AB026105.1       ------------------------------------------------------
------ 0
```

MW148603.1
ATTTCACTTATTGTACCAATCCTACTCGCCGTAGCTTTCCTGACATTAGTAGAACGAAAA 2820
AB051263.1 --
------ 0
AF068544.1 --
------ 0

JX982502.1 --
------ 0
JX982501.1 --
------ 0
JX982498.1 --
------ 0
JX982497.1 --
------ 0
JX982495.1 --
------ 0
JX982496.1 --
------ 0
JX982500.1 --
------ 0
EU548051.1 --
------ 0
EU548044.1 --
------ 0
EU548042.1 --
------ 0
EU548043.1 --
------ 0
EU548047.1 --
------ 0
EU548050.1 --
------ 0
EU548048.1 --
------ 0
EU548049.1 --
------ 0
AF207722.1 --
------ 0
EU548037.1 --
------ 0
AF207723.1 --
------ 0
EU548038.1 --
------ 0
EU548036.1 --
------ 0
EU548035.1 --
------ 0
AF207720.1 --
------ 0
AB601576.1 --
------ 0
EU548040.1 --
------ 0
EU548039.1 --
------ 0
AF207721.1 --
------ 0
AF207724.1 --
------ 0
JX982499.1 --
------ 0
EU548041.1 --
------ 0

```
   EU548045.1      ------------------------------------------------------
------ 0
   AF207725.1      ------------------------------------------------------
------ 0
   EU548046.1      ------------------------------------------------------
------ 0
   AF207714.1      ------------------------------------------------------
------ 0
   AF207713.1      ------------------------------------------------------
------ 0
   AF207712.1      ------------------------------------------------------
------ 0
   AY750628.1      ------------------------------------------------------
------ 0
   EF689084.1      ------------------------------------------------------
------ 0
   EF689085.1      ------------------------------------------------------
------ 0
   AB119070.1      ------------------------------------------------------
------ 956
   EF987742.1      ------------------------------------------------------
------ 0
   AB026105.1      ------------------------------------------------------
------ 0
   MW148603.1
GTCTTAGGATACATACAACTTCGCAAAGGCCCAAACATTGTAGGACCCTACGGCCTCCTA   2880
   AB051263.1      ------------------------------------------------------
------ 0
   AF068544.1      ------------------------------------------------------
------ 0


   JX982502.1      ------------------------------------------------------
------ 0
   JX982501.1      ------------------------------------------------------
------ 0
   JX982498.1      ------------------------------------------------------
------ 0
   JX982497.1      ------------------------------------------------------
------ 0
   JX982495.1      ------------------------------------------------------
------ 0
   JX982496.1      ------------------------------------------------------
------ 0
   JX982500.1      ------------------------------------------------------
------ 0
   EU548051.1      ------------------------------------------------------
------ 0
   EU548044.1      ------------------------------------------------------
------ 0
   EU548042.1      ------------------------------------------------------
------ 0
   EU548043.1      ------------------------------------------------------
------ 0
   EU548047.1      ------------------------------------------------------
------ 0
   EU548050.1      ------------------------------------------------------
------ 0
   EU548048.1      ------------------------------------------------------
------ 0
   EU548049.1      ------------------------------------------------------
------ 0
   AF207722.1      ------------------------------------------------------
------ 0
   EU548037.1      ------------------------------------------------------
------ 0
```

```
   AF207723.1      ------------------------------------------------------
------ 0
   EU548038.1      ------------------------------------------------------
------ 0
   EU548036.1      ------------------------------------------------------
------ 0
   EU548035.1      ------------------------------------------------------
------ 0
   AF207720.1      ------------------------------------------------------
------ 0
   AB601576.1      ------------------------------------------------------
------ 0
   EU548040.1      ------------------------------------------------------
------ 0
   EU548039.1      ------------------------------------------------------
------ 0
   AF207721.1      ------------------------------------------------------
------ 0
   AF207724.1      ------------------------------------------------------
------ 0
   JX982499.1      ------------------------------------------------------
------ 0
   EU548041.1      ------------------------------------------------------
------ 0
   EU548045.1      ------------------------------------------------------
------ 0
   AF207725.1      ------------------------------------------------------
------ 0
   EU548046.1      ------------------------------------------------------
------ 0
   AF207714.1      ------------------------------------------------------
------ 0
   AF207713.1      ------------------------------------------------------
------ 0
   AF207712.1      ------------------------------------------------------
------ 0
   AY750628.1      ------------------------------------------------------
------ 0
   EF689084.1      ------------------------------------------------------
------ 0
   EF689085.1      ------------------------------------------------------
------ 0
   AB119070.1      ------------------------------------------------------
------ 956
   EF987742.1      ------------------------------------------------------
------ 0
   AB026105.1      ------------------------------------------------------
------ 0
   MW148603.1
CAACCAATTGCAGATGCTGTAAAACTTTTCACAAAAGAGCCATTACGACCCCTAACATCA   2940
   AB051263.1      ------------------------------------------------------
------ 0
   AF068544.1      ------------------------------------------------------
------ 0


   JX982502.1      ------------------------------------------------------
------ 0
   JX982501.1      ------------------------------------------------------
------ 0
   JX982498.1      ------------------------------------------------------
------ 0
   JX982497.1      ------------------------------------------------------
------ 0
   JX982495.1      ------------------------------------------------------
------ 0
```

```
    JX982496.1      ------------------------------------------------------
------ 0
    JX982500.1      ------------------------------------------------------
------ 0
    EU548051.1      ------------------------------------------------------
------ 0
    EU548044.1      ------------------------------------------------------
------ 0
    EU548042.1      ------------------------------------------------------
------ 0
    EU548043.1      ------------------------------------------------------
------ 0
    EU548047.1      ------------------------------------------------------
------ 0
    EU548050.1      ------------------------------------------------------
------ 0
    EU548048.1      ------------------------------------------------------
------ 0
    EU548049.1      ------------------------------------------------------
------ 0
    AF207722.1      ------------------------------------------------------
------ 0
    EU548037.1      ------------------------------------------------------
------ 0
    AF207723.1      ------------------------------------------------------
------ 0
    EU548038.1      ------------------------------------------------------
------ 0
    EU548036.1      ------------------------------------------------------
------ 0
    EU548035.1      ------------------------------------------------------
------ 0
    AF207720.1      ------------------------------------------------------
------ 0
    AB601576.1      ------------------------------------------------------
------ 0
    EU548040.1      ------------------------------------------------------
------ 0
    EU548039.1      ------------------------------------------------------
------ 0
    AF207721.1      ------------------------------------------------------
------ 0
    AF207724.1      ------------------------------------------------------
------ 0
    JX982499.1      ------------------------------------------------------
------ 0
    EU548041.1      ------------------------------------------------------
------ 0
    EU548045.1      ------------------------------------------------------
------ 0
    AF207725.1      ------------------------------------------------------
------ 0
    EU548046.1      ------------------------------------------------------
------ 0
    AF207714.1      ------------------------------------------------------
------ 0
    AF207713.1      ------------------------------------------------------
------ 0
    AF207712.1      ------------------------------------------------------
------ 0
    AY750628.1      ------------------------------------------------------
------ 0
    EF689084.1      ------------------------------------------------------
------ 0
    EF689085.1      ------------------------------------------------------
------ 0
```

```
    AB119070.1      ------------------------------------------------------
------ 956
    EF987742.1      ------------------------------------------------------
------ 0
    AB026105.1      ------------------------------------------------------
------ 0
    MW148603.1
TCTATTACCATATTCGTCATAGCTCCTATCCTAGCCCTTACACTAGCCCTAACAATATGA   3000
    AB051263.1      ------------------------------------------------------
------ 0
    AF068544.1      ------------------------------------------------------
------ 0


    JX982502.1      ------------------------------------------------------
------ 0
    JX982501.1      ------------------------------------------------------
------ 0
    JX982498.1      ------------------------------------------------------
------ 0
    JX982497.1      ------------------------------------------------------
------ 0
    JX982495.1      ------------------------------------------------------
------ 0
    JX982496.1      ------------------------------------------------------
------ 0
    JX982500.1      ------------------------------------------------------
------ 0
    EU548051.1      ------------------------------------------------------
------ 0
    EU548044.1      ------------------------------------------------------
------ 0
    EU548042.1      ------------------------------------------------------
------ 0
    EU548043.1      ------------------------------------------------------
------ 0
    EU548047.1      ------------------------------------------------------
------ 0
    EU548050.1      ------------------------------------------------------
------ 0
    EU548048.1      ------------------------------------------------------
------ 0
    EU548049.1      ------------------------------------------------------
------ 0
    AF207722.1      ------------------------------------------------------
------ 0
    EU548037.1      ------------------------------------------------------
------ 0
    AF207723.1      ------------------------------------------------------
------ 0
    EU548038.1      ------------------------------------------------------
------ 0
    EU548036.1      ------------------------------------------------------
------ 0
    EU548035.1      ------------------------------------------------------
------ 0
    AF207720.1      ------------------------------------------------------
------ 0
    AB601576.1      ------------------------------------------------------
------ 0
    EU548040.1      ------------------------------------------------------
------ 0
    EU548039.1      ------------------------------------------------------
------ 0
    AF207721.1      ------------------------------------------------------
------ 0
```

```
    AF207724.1      ------------------------------------------------------
------ 0
    JX982499.1      ------------------------------------------------------
------ 0
    EU548041.1      ------------------------------------------------------
------ 0
    EU548045.1      ------------------------------------------------------
------ 0
    AF207725.1      ------------------------------------------------------
------ 0
    EU548046.1      ------------------------------------------------------
------ 0
    AF207714.1      ------------------------------------------------------
------ 0
    AF207713.1      ------------------------------------------------------
------ 0
    AF207712.1      ------------------------------------------------------
------ 0
    AY750628.1      ------------------------------------------------------
------ 0
    EF689084.1      ------------------------------------------------------
------ 0
    EF689085.1      ------------------------------------------------------
------ 0
    AB119070.1      ------------------------------------------------------
------ 956
    EF987742.1      ------------------------------------------------------
------ 0
    AB026105.1      ------------------------------------------------------
------ 0
    MW148603.1
ATCCCACTACCAATGCCCTATCCCCTTATCAATATGAACTTAGGGATCCTATTTATACTA   3060
    AB051263.1      ------------------------------------------------------
------ 0
    AF068544.1      ------------------------------------------------------
------ 0


    JX982502.1      ------------------------------------------------------
------ 0
    JX982501.1      ------------------------------------------------------
------ 0
    JX982498.1      ------------------------------------------------------
------ 0
    JX982497.1      ------------------------------------------------------
------ 0
    JX982495.1      ------------------------------------------------------
------ 0
    JX982496.1      ------------------------------------------------------
------ 0
    JX982500.1      ------------------------------------------------------
------ 0
    EU548051.1      ------------------------------------------------------
------ 0
    EU548044.1      ------------------------------------------------------
------ 0
    EU548042.1      ------------------------------------------------------
------ 0
    EU548043.1      ------------------------------------------------------
------ 0
    EU548047.1      ------------------------------------------------------
------ 0
    EU548050.1      ------------------------------------------------------
------ 0
    EU548048.1      ------------------------------------------------------
------ 0
```

```
    EU548049.1      ------------------------------------------------------
------ 0
    AF207722.1      ------------------------------------------------------
------ 0
    EU548037.1      ------------------------------------------------------
------ 0
    AF207723.1      ------------------------------------------------------
------ 0
    EU548038.1      ------------------------------------------------------
------ 0
    EU548036.1      ------------------------------------------------------
------ 0
    EU548035.1      ------------------------------------------------------
------ 0
    AF207720.1      ------------------------------------------------------
------ 0
    AB601576.1      ------------------------------------------------------
------ 0
    EU548040.1      ------------------------------------------------------
------ 0
    EU548039.1      ------------------------------------------------------
------ 0
    AF207721.1      ------------------------------------------------------
------ 0
    AF207724.1      ------------------------------------------------------
------ 0
    JX982499.1      ------------------------------------------------------
------ 0
    EU548041.1      ------------------------------------------------------
------ 0
    EU548045.1      ------------------------------------------------------
------ 0
    AF207725.1      ------------------------------------------------------
------ 0
    EU548046.1      ------------------------------------------------------
------ 0
    AF207714.1      ------------------------------------------------------
------ 0
    AF207713.1      ------------------------------------------------------
------ 0
    AF207712.1      ------------------------------------------------------
------ 0
    AY750628.1      ------------------------------------------------------
------ 0
    EF689084.1      ------------------------------------------------------
------ 0
    EF689085.1      ------------------------------------------------------
------ 0
    AB119070.1      ------------------------------------------------------
------ 956
    EF987742.1      ------------------------------------------------------
------ 0
    AB026105.1      ------------------------------------------------------
------ 0
    MW148603.1
GCAATATCAAGCCTAGCTGTTTACTCTATCCTATGATCCGGATGGGCCTCAAACTCAAAA   3120
    AB051263.1      ------------------------------------------------------
------ 0
    AF068544.1      ------------------------------------------------------
------ 0


    JX982502.1      ------------------------------------------------------
------ 0
    JX982501.1      ------------------------------------------------------
------ 0
```

```
    JX982498.1       ------------------------------------------------------
------ 0
    JX982497.1       ------------------------------------------------------
------ 0
    JX982495.1       ------------------------------------------------------
------ 0
    JX982496.1       ------------------------------------------------------
------ 0
    JX982500.1       ------------------------------------------------------
------ 0
    EU548051.1       ------------------------------------------------------
------ 0
    EU548044.1       ------------------------------------------------------
------ 0
    EU548042.1       ------------------------------------------------------
------ 0
    EU548043.1       ------------------------------------------------------
------ 0
    EU548047.1       ------------------------------------------------------
------ 0
    EU548050.1       ------------------------------------------------------
------ 0
    EU548048.1       ------------------------------------------------------
------ 0
    EU548049.1       ------------------------------------------------------
------ 0
    AF207722.1       ------------------------------------------------------
------ 0
    EU548037.1       ------------------------------------------------------
------ 0
    AF207723.1       ------------------------------------------------------
------ 0
    EU548038.1       ------------------------------------------------------
------ 0
    EU548036.1       ------------------------------------------------------
------ 0
    EU548035.1       ------------------------------------------------------
------ 0
    AF207720.1       ------------------------------------------------------
------ 0
    AB601576.1       ------------------------------------------------------
------ 0
    EU548040.1       ------------------------------------------------------
------ 0
    EU548039.1       ------------------------------------------------------
------ 0
    AF207721.1       ------------------------------------------------------
------ 0
    AF207724.1       ------------------------------------------------------
------ 0
    JX982499.1       ------------------------------------------------------
------ 0
    EU548041.1       ------------------------------------------------------
------ 0
    EU548045.1       ------------------------------------------------------
------ 0
    AF207725.1       ------------------------------------------------------
------ 0
    EU548046.1       ------------------------------------------------------
------ 0
    AF207714.1       ------------------------------------------------------
------ 0
    AF207713.1       ------------------------------------------------------
------ 0
    AF207712.1       ------------------------------------------------------
------ 0
```

```
    AY750628.1        ------------------------------------------------------
------ 0
    EF689084.1        ------------------------------------------------------
------ 0
    EF689085.1        ------------------------------------------------------
------ 0
    AB119070.1        ------------------------------------------------------
------ 956
    EF987742.1        ------------------------------------------------------
------ 0
    AB026105.1        ------------------------------------------------------
------ 0
    MW148603.1
TACGCCCTAATCGGAGCCCTACGGGCCGTAGCCCAAACAATCTCCTACGAAGTCACATTA   3180
    AB051263.1        ------------------------------------------------------
------ 0
    AF068544.1        ------------------------------------------------------
------ 0


    JX982502.1        ------------------------------------------------------
------ 0
    JX982501.1        ------------------------------------------------------
------ 0
    JX982498.1        ------------------------------------------------------
------ 0
    JX982497.1        ------------------------------------------------------
------ 0
    JX982495.1        ------------------------------------------------------
------ 0
    JX982496.1        ------------------------------------------------------
------ 0
    JX982500.1        ------------------------------------------------------
------ 0
    EU548051.1        ------------------------------------------------------
------ 0
    EU548044.1        ------------------------------------------------------
------ 0
    EU548042.1        ------------------------------------------------------
------ 0
    EU548043.1        ------------------------------------------------------
------ 0
    EU548047.1        ------------------------------------------------------
------ 0
    EU548050.1        ------------------------------------------------------
------ 0
    EU548048.1        ------------------------------------------------------
------ 0
    EU548049.1        ------------------------------------------------------
------ 0
    AF207722.1        ------------------------------------------------------
------ 0
    EU548037.1        ------------------------------------------------------
------ 0
    AF207723.1        ------------------------------------------------------
------ 0
    EU548038.1        ------------------------------------------------------
------ 0
    EU548036.1        ------------------------------------------------------
------ 0
    EU548035.1        ------------------------------------------------------
------ 0
    AF207720.1        ------------------------------------------------------
------ 0
    AB601576.1        ------------------------------------------------------
------ 0
```

```
   EU548040.1      ------------------------------------------------------
------ 0
   EU548039.1      ------------------------------------------------------
------ 0
   AF207721.1      ------------------------------------------------------
------ 0
   AF207724.1      ------------------------------------------------------
------ 0
   JX982499.1      ------------------------------------------------------
------ 0
   EU548041.1      ------------------------------------------------------
------ 0
   EU548045.1      ------------------------------------------------------
------ 0
   AF207725.1      ------------------------------------------------------
------ 0
   EU548046.1      ------------------------------------------------------
------ 0
   AF207714.1      ------------------------------------------------------
------ 0
   AF207713.1      ------------------------------------------------------
------ 0
   AF207712.1      ------------------------------------------------------
------ 0
   AY750628.1      ------------------------------------------------------
------ 0
   EF689084.1      ------------------------------------------------------
------ 0
   EF689085.1      ------------------------------------------------------
------ 0
   AB119070.1      ------------------------------------------------------
------ 956
   EF987742.1      ------------------------------------------------------
------ 0
   AB026105.1      ------------------------------------------------------
------ 0
   MW148603.1
GCCATCATCCTATTATCAGTCCTACTAATAAATGGCTCCTTTACCCTATCCACTCTAATC   3240
   AB051263.1      ------------------------------------------------------
------ 0
   AF068544.1      ------------------------------------------------------
------ 0


   JX982502.1      ------------------------------------------------------
------ 0
   JX982501.1      ------------------------------------------------------
------ 0
   JX982498.1      ------------------------------------------------------
------ 0
   JX982497.1      ------------------------------------------------------
------ 0
   JX982495.1      ------------------------------------------------------
------ 0
   JX982496.1      ------------------------------------------------------
------ 0
   JX982500.1      ------------------------------------------------------
------ 0
   EU548051.1      ------------------------------------------------------
------ 0
   EU548044.1      ------------------------------------------------------
------ 0
   EU548042.1      ------------------------------------------------------
------ 0
   EU548043.1      ------------------------------------------------------
------ 0
```

```
   EU548047.1      ------------------------------------------------------
------ 0
   EU548050.1      ------------------------------------------------------
------ 0
   EU548048.1      ------------------------------------------------------
------ 0
   EU548049.1      ------------------------------------------------------
------ 0
   AF207722.1      ------------------------------------------------------
------ 0
   EU548037.1      ------------------------------------------------------
------ 0
   AF207723.1      ------------------------------------------------------
------ 0
   EU548038.1      ------------------------------------------------------
------ 0
   EU548036.1      ------------------------------------------------------
------ 0
   EU548035.1      ------------------------------------------------------
------ 0
   AF207720.1      ------------------------------------------------------
------ 0
   AB601576.1      ------------------------------------------------------
------ 0
   EU548040.1      ------------------------------------------------------
------ 0
   EU548039.1      ------------------------------------------------------
------ 0
   AF207721.1      ------------------------------------------------------
------ 0
   AF207724.1      ------------------------------------------------------
------ 0
   JX982499.1      ------------------------------------------------------
------ 0
   EU548041.1      ------------------------------------------------------
------ 0
   EU548045.1      ------------------------------------------------------
------ 0
   AF207725.1      ------------------------------------------------------
------ 0
   EU548046.1      ------------------------------------------------------
------ 0
   AF207714.1      ------------------------------------------------------
------ 0
   AF207713.1      ------------------------------------------------------
------ 0
   AF207712.1      ------------------------------------------------------
------ 0
   AY750628.1      ------------------------------------------------------
------ 0
   EF689084.1      ------------------------------------------------------
------ 0
   EF689085.1      ------------------------------------------------------
------ 0
   AB119070.1      ------------------------------------------------------
------ 956
   EF987742.1      ------------------------------------------------------
------ 0
   AB026105.1      ------------------------------------------------------
------ 0
   MW148603.1
GTCACACAAGAACACCTATGACTAATTTTCCCTGCATGACCTTTAGCTATAATATGATTT   3300
   AB051263.1      ------------------------------------------------------
------ 0
   AF068544.1      ------------------------------------------------------
------ 0
```

```
   JX982502.1      ------------------------------------------------------
------ 0
   JX982501.1      ------------------------------------------------------
------ 0
   JX982498.1      ------------------------------------------------------
------ 0
   JX982497.1      ------------------------------------------------------
------ 0
   JX982495.1      ------------------------------------------------------
------ 0
   JX982496.1      ------------------------------------------------------
------ 0
   JX982500.1      ------------------------------------------------------
------ 0
   EU548051.1      ------------------------------------------------------
------ 0
   EU548044.1      ------------------------------------------------------
------ 0
   EU548042.1      ------------------------------------------------------
------ 0
   EU548043.1      ------------------------------------------------------
------ 0
   EU548047.1      ------------------------------------------------------
------ 0
   EU548050.1      ------------------------------------------------------
------ 0
   EU548048.1      ------------------------------------------------------
------ 0
   EU548049.1      ------------------------------------------------------
------ 0
   AF207722.1      ------------------------------------------------------
------ 0
   EU548037.1      ------------------------------------------------------
------ 0
   AF207723.1      ------------------------------------------------------
------ 0
   EU548038.1      ------------------------------------------------------
------ 0
   EU548036.1      ------------------------------------------------------
------ 0
   EU548035.1      ------------------------------------------------------
------ 0
   AF207720.1      ------------------------------------------------------
------ 0
   AB601576.1      ------------------------------------------------------
------ 0
   EU548040.1      ------------------------------------------------------
------ 0
   EU548039.1      ------------------------------------------------------
------ 0
   AF207721.1      ------------------------------------------------------
------ 0
   AF207724.1      ------------------------------------------------------
------ 0
   JX982499.1      ------------------------------------------------------
------ 0
   EU548041.1      ------------------------------------------------------
------ 0
   EU548045.1      ------------------------------------------------------
------ 0
   AF207725.1      ------------------------------------------------------
------ 0
   EU548046.1      ------------------------------------------------------
------ 0
```

AF207714.1 --
------ 0
AF207713.1 --
------ 0
AF207712.1 --
------ 0
AY750628.1 --
------ 0
EF689084.1 --
------ 0
EF689085.1 --
------ 0
AB119070.1 --
------ 956
EF987742.1 --
------ 0
AB026105.1 --
------ 0
MW148603.1
ATCTCAACCTTAGCAGAAACTAACCGCGCCCCGTTCGACCTAACTGAGGGAGAATCCGAA 3360
AB051263.1 --
------ 0
AF068544.1 --
------ 0

JX982502.1 --
------ 0
JX982501.1 --
------ 0
JX982498.1 --
------ 0
JX982497.1 --
------ 0
JX982495.1 --
------ 0
JX982496.1 --
------ 0
JX982500.1 --
------ 0
EU548051.1 --
------ 0
EU548044.1 --
------ 0
EU548042.1 --
------ 0
EU548043.1 --
------ 0
EU548047.1 --
------ 0
EU548050.1 --
------ 0
EU548048.1 --
------ 0
EU548049.1 --
------ 0
AF207722.1 --
------ 0
EU548037.1 --
------ 0
AF207723.1 --
------ 0
EU548038.1 --
------ 0
EU548036.1 --
------ 0

```
   EU548035.1       ------------------------------------------------------
------ 0
   AF207720.1       ------------------------------------------------------
------ 0
   AB601576.1       ------------------------------------------------------
------ 0
   EU548040.1       ------------------------------------------------------
------ 0
   EU548039.1       ------------------------------------------------------
------ 0
   AF207721.1       ------------------------------------------------------
------ 0
   AF207724.1       ------------------------------------------------------
------ 0
   JX982499.1       ------------------------------------------------------
------ 0
   EU548041.1       ------------------------------------------------------
------ 0
   EU548045.1       ------------------------------------------------------
------ 0
   AF207725.1       ------------------------------------------------------
------ 0
   EU548046.1       ------------------------------------------------------
------ 0
   AF207714.1       ------------------------------------------------------
------ 0
   AF207713.1       ------------------------------------------------------
------ 0
   AF207712.1       ------------------------------------------------------
------ 0
   AY750628.1       ------------------------------------------------------
------ 0
   EF689084.1       ------------------------------------------------------
------ 0
   EF689085.1       ------------------------------------------------------
------ 0
   AB119070.1       ------------------------------------------------------
------ 956
   EF987742.1       ------------------------------------------------------
------ 0
   AB026105.1       ------------------------------------------------------
------ 0
   MW148603.1
CTAGTTTCAGGGTTCAACGTCGAATATGCAGCAGGACCATTCGCCCTATTCTTCCTAGCC   3420
   AB051263.1       ------------------------------------------------------
------ 0
   AF068544.1       ------------------------------------------------------
------ 0


   JX982502.1       ------------------------------------------------------
------ 0
   JX982501.1       ------------------------------------------------------
------ 0
   JX982498.1       ------------------------------------------------------
------ 0
   JX982497.1       ------------------------------------------------------
------ 0
   JX982495.1       ------------------------------------------------------
------ 0
   JX982496.1       ------------------------------------------------------
------ 0
   JX982500.1       ------------------------------------------------------
------ 0
   EU548051.1       ------------------------------------------------------
------ 0
```

```
   EU548044.1       ------------------------------------------------------
------ 0
   EU548042.1       ------------------------------------------------------
------ 0
   EU548043.1       ------------------------------------------------------
------ 0
   EU548047.1       ------------------------------------------------------
------ 0
   EU548050.1       ------------------------------------------------------
------ 0
   EU548048.1       ------------------------------------------------------
------ 0
   EU548049.1       ------------------------------------------------------
------ 0
   AF207722.1       ------------------------------------------------------
------ 0
   EU548037.1       ------------------------------------------------------
------ 0
   AF207723.1       ------------------------------------------------------
------ 0
   EU548038.1       ------------------------------------------------------
------ 0
   EU548036.1       ------------------------------------------------------
------ 0
   EU548035.1       ------------------------------------------------------
------ 0
   AF207720.1       ------------------------------------------------------
------ 0
   AB601576.1       ------------------------------------------------------
------ 0
   EU548040.1       ------------------------------------------------------
------ 0
   EU548039.1       ------------------------------------------------------
------ 0
   AF207721.1       ------------------------------------------------------
------ 0
   AF207724.1       ------------------------------------------------------
------ 0
   JX982499.1       ------------------------------------------------------
------ 0
   EU548041.1       ------------------------------------------------------
------ 0
   EU548045.1       ------------------------------------------------------
------ 0
   AF207725.1       ------------------------------------------------------
------ 0
   EU548046.1       ------------------------------------------------------
------ 0
   AF207714.1       ------------------------------------------------------
------ 0
   AF207713.1       ------------------------------------------------------
------ 0
   AF207712.1       ------------------------------------------------------
------ 0
   AY750628.1       ------------------------------------------------------
------ 0
   EF689084.1       ------------------------------------------------------
------ 0
   EF689085.1       ------------------------------------------------------
------ 0
   AB119070.1       ------------------------------------------------------
------ 956
   EF987742.1       ------------------------------------------------------
------ 0
   AB026105.1       ------------------------------------------------------
------ 0
```

```
    MW148603.1
GAATACGCCAACATCATCATAATAAATATCCTCACAACTATCCTATTCTTCGGCGCATTT   3480
    AB051263.1      ------------------------------------------------------
------ 0
    AF068544.1      ------------------------------------------------------
------ 0


    JX982502.1      ------------------------------------------------------
------ 0
    JX982501.1      ------------------------------------------------------
------ 0
    JX982498.1      ------------------------------------------------------
------ 0
    JX982497.1      ------------------------------------------------------
------ 0
    JX982495.1      ------------------------------------------------------
------ 0
    JX982496.1      ------------------------------------------------------
------ 0
    JX982500.1      ------------------------------------------------------
------ 0
    EU548051.1      ------------------------------------------------------
------ 0
    EU548044.1      ------------------------------------------------------
------ 0
    EU548042.1      ------------------------------------------------------
------ 0
    EU548043.1      ------------------------------------------------------
------ 0
    EU548047.1      ------------------------------------------------------
------ 0
    EU548050.1      ------------------------------------------------------
------ 0
    EU548048.1      ------------------------------------------------------
------ 0
    EU548049.1      ------------------------------------------------------
------ 0
    AF207722.1      ------------------------------------------------------
------ 0
    EU548037.1      ------------------------------------------------------
------ 0
    AF207723.1      ------------------------------------------------------
------ 0
    EU548038.1      ------------------------------------------------------
------ 0
    EU548036.1      ------------------------------------------------------
------ 0
    EU548035.1      ------------------------------------------------------
------ 0
    AF207720.1      ------------------------------------------------------
------ 0
    AB601576.1      ------------------------------------------------------
------ 0
    EU548040.1      ------------------------------------------------------
------ 0
    EU548039.1      ------------------------------------------------------
------ 0
    AF207721.1      ------------------------------------------------------
------ 0
    AF207724.1      ------------------------------------------------------
------ 0
    JX982499.1      ------------------------------------------------------
------ 0
    EU548041.1      ------------------------------------------------------
------ 0
```

```
   EU548045.1      ------------------------------------------------------
------ 0
   AF207725.1      ------------------------------------------------------
------ 0
   EU548046.1      ------------------------------------------------------
------ 0
   AF207714.1      ------------------------------------------------------
------ 0
   AF207713.1      ------------------------------------------------------
------ 0
   AF207712.1      ------------------------------------------------------
------ 0
   AY750628.1      ------------------------------------------------------
------ 0
   EF689084.1      ------------------------------------------------------
------ 0
   EF689085.1      ------------------------------------------------------
------ 0
   AB119070.1      ------------------------------------------------------
------ 956
   EF987742.1      ------------------------------------------------------
------ 0
   AB026105.1      ------------------------------------------------------
------ 0
   MW148603.1
CACACCCCCTACCTTCCAGAATTATATTCCATTAATTTCACTATAAAAACCCTCTTATTA   3540
   AB051263.1      ------------------------------------------------------
------ 0
   AF068544.1      ------------------------------------------------------
------ 0


   JX982502.1      ------------------------------------------------------
------ 0
   JX982501.1      ------------------------------------------------------
------ 0
   JX982498.1      ------------------------------------------------------
------ 0
   JX982497.1      ------------------------------------------------------
------ 0
   JX982495.1      ------------------------------------------------------
------ 0
   JX982496.1      ------------------------------------------------------
------ 0
   JX982500.1      ------------------------------------------------------
------ 0
   EU548051.1      ------------------------------------------------------
------ 0
   EU548044.1      ------------------------------------------------------
------ 0
   EU548042.1      ------------------------------------------------------
------ 0
   EU548043.1      ------------------------------------------------------
------ 0
   EU548047.1      ------------------------------------------------------
------ 0
   EU548050.1      ------------------------------------------------------
------ 0
   EU548048.1      ------------------------------------------------------
------ 0
   EU548049.1      ------------------------------------------------------
------ 0
   AF207722.1      ------------------------------------------------------
------ 0
   EU548037.1      ------------------------------------------------------
------ 0
```

```
   AF207723.1      ------------------------------------------------------
------ 0
   EU548038.1      ------------------------------------------------------
------ 0
   EU548036.1      ------------------------------------------------------
------ 0
   EU548035.1      ------------------------------------------------------
------ 0
   AF207720.1      ------------------------------------------------------
------ 0
   AB601576.1      ------------------------------------------------------
------ 0
   EU548040.1      ------------------------------------------------------
------ 0
   EU548039.1      ------------------------------------------------------
------ 0
   AF207721.1      ------------------------------------------------------
------ 0
   AF207724.1      ------------------------------------------------------
------ 0
   JX982499.1      ------------------------------------------------------
------ 0
   EU548041.1      ------------------------------------------------------
------ 0
   EU548045.1      ------------------------------------------------------
------ 0
   AF207725.1      ------------------------------------------------------
------ 0
   EU548046.1      ------------------------------------------------------
------ 0
   AF207714.1      ------------------------------------------------------
------ 0
   AF207713.1      ------------------------------------------------------
------ 0
   AF207712.1      ------------------------------------------------------
------ 0
   AY750628.1      ------------------------------------------------------
------ 0
   EF689084.1      ------------------------------------------------------
------ 0
   EF689085.1      ------------------------------------------------------
------ 0
   AB119070.1      ------------------------------------------------------
------ 956
   EF987742.1      ------------------------------------------------------
------ 0
   AB026105.1      ------------------------------------------------------
------ 0
   MW148603.1
ACAACTTCTTTCCTATGAATTCGAGCATCATACCCCCGATTCCGCTATGACCAACTAATA   3600
   AB051263.1      ------------------------------------------------------
------ 0
   AF068544.1      ------------------------------------------------------
------ 0


   JX982502.1      ------------------------------------------------------
------ 0
   JX982501.1      ------------------------------------------------------
------ 0
   JX982498.1      ------------------------------------------------------
------ 0
   JX982497.1      ------------------------------------------------------
------ 0
   JX982495.1      ------------------------------------------------------
------ 0
```

```
    JX982496.1       ------------------------------------------------------
------ 0
    JX982500.1       ------------------------------------------------------
------ 0
    EU548051.1       ------------------------------------------------------
------ 0
    EU548044.1       ------------------------------------------------------
------ 0
    EU548042.1       ------------------------------------------------------
------ 0
    EU548043.1       ------------------------------------------------------
------ 0
    EU548047.1       ------------------------------------------------------
------ 0
    EU548050.1       ------------------------------------------------------
------ 0
    EU548048.1       ------------------------------------------------------
------ 0
    EU548049.1       ------------------------------------------------------
------ 0
    AF207722.1       ------------------------------------------------------
------ 0
    EU548037.1       ------------------------------------------------------
------ 0
    AF207723.1       ------------------------------------------------------
------ 0
    EU548038.1       ------------------------------------------------------
------ 0
    EU548036.1       ------------------------------------------------------
------ 0
    EU548035.1       ------------------------------------------------------
------ 0
    AF207720.1       ------------------------------------------------------
------ 0
    AB601576.1       ------------------------------------------------------
------ 0
    EU548040.1       ------------------------------------------------------
------ 0
    EU548039.1       ------------------------------------------------------
------ 0
    AF207721.1       ------------------------------------------------------
------ 0
    AF207724.1       ------------------------------------------------------
------ 0
    JX982499.1       ------------------------------------------------------
------ 0
    EU548041.1       ------------------------------------------------------
------ 0
    EU548045.1       ------------------------------------------------------
------ 0
    AF207725.1       ------------------------------------------------------
------ 0
    EU548046.1       ------------------------------------------------------
------ 0
    AF207714.1       ------------------------------------------------------
------ 0
    AF207713.1       ------------------------------------------------------
------ 0
    AF207712.1       ------------------------------------------------------
------ 0
    AY750628.1       ------------------------------------------------------
------ 0
    EF689084.1       ------------------------------------------------------
------ 0
    EF689085.1       ------------------------------------------------------
------ 0
```

```
    AB119070.1      ------------------------------------------------------
------ 956
    EF987742.1      ------------------------------------------------------
------ 0
    AB026105.1      ------------------------------------------------------
------ 0
    MW148603.1
CACTTGCTATGAAAAAATTTTCTTCCCTTGACACTAGCCTTATGTATATGACATATAGCC   3660
    AB051263.1      ------------------------------------------------------
------ 0
    AF068544.1      ------------------------------------------------------
------ 0


    JX982502.1      ------------------------------------------------------
------ 0
    JX982501.1      ------------------------------------------------------
------ 0
    JX982498.1      ------------------------------------------------------
------ 0
    JX982497.1      ------------------------------------------------------
------ 0
    JX982495.1      ------------------------------------------------------
------ 0
    JX982496.1      ------------------------------------------------------
------ 0
    JX982500.1      ------------------------------------------------------
------ 0
    EU548051.1      ------------------------------------------------------
------ 0
    EU548044.1      ------------------------------------------------------
------ 0
    EU548042.1      ------------------------------------------------------
------ 0
    EU548043.1      ------------------------------------------------------
------ 0
    EU548047.1      ------------------------------------------------------
------ 0
    EU548050.1      ------------------------------------------------------
------ 0
    EU548048.1      ------------------------------------------------------
------ 0
    EU548049.1      ------------------------------------------------------
------ 0
    AF207722.1      ------------------------------------------------------
------ 0
    EU548037.1      ------------------------------------------------------
------ 0
    AF207723.1      ------------------------------------------------------
------ 0
    EU548038.1      ------------------------------------------------------
------ 0
    EU548036.1      ------------------------------------------------------
------ 0
    EU548035.1      ------------------------------------------------------
------ 0
    AF207720.1      ------------------------------------------------------
------ 0
    AB601576.1      ------------------------------------------------------
------ 0
    EU548040.1      ------------------------------------------------------
------ 0
    EU548039.1      ------------------------------------------------------
------ 0
    AF207721.1      ------------------------------------------------------
------ 0
```

```
    AF207724.1       ------------------------------------------------------
------ 0
    JX982499.1       ------------------------------------------------------
------ 0
    EU548041.1       ------------------------------------------------------
------ 0
    EU548045.1       ------------------------------------------------------
------ 0
    AF207725.1       ------------------------------------------------------
------ 0
    EU548046.1       ------------------------------------------------------
------ 0
    AF207714.1       ------------------------------------------------------
------ 0
    AF207713.1       ------------------------------------------------------
------ 0
    AF207712.1       ------------------------------------------------------
------ 0
    AY750628.1       ------------------------------------------------------
------ 0
    EF689084.1       ------------------------------------------------------
------ 0
    EF689085.1       ------------------------------------------------------
------ 0
    AB119070.1       ------------------------------------------------------
------ 956
    EF987742.1       ------------------------------------------------------
------ 0
    AB026105.1       ------------------------------------------------------
------ 0
    MW148603.1
CTCCCCATTATAACTGCGAGCATCCCCCCACAAACATAAGAAATATGTCTGACAAAAGAG   3720
    AB051263.1       ------------------------------------------------------
------ 0
    AF068544.1       ------------------------------------------------------
------ 0


    JX982502.1       ------------------------------------------------------
------ 0
    JX982501.1       ------------------------------------------------------
------ 0
    JX982498.1       ------------------------------------------------------
------ 0
    JX982497.1       ------------------------------------------------------
------ 0
    JX982495.1       ------------------------------------------------------
------ 0
    JX982496.1       ------------------------------------------------------
------ 0
    JX982500.1       ------------------------------------------------------
------ 0
    EU548051.1       ------------------------------------------------------
------ 0
    EU548044.1       ------------------------------------------------------
------ 0
    EU548042.1       ------------------------------------------------------
------ 0
    EU548043.1       ------------------------------------------------------
------ 0
    EU548047.1       ------------------------------------------------------
------ 0
    EU548050.1       ------------------------------------------------------
------ 0
    EU548048.1       ------------------------------------------------------
------ 0
```

EU548049.1 --
------ 0
AF207722.1 --
------ 0
EU548037.1 --
------ 0
AF207723.1 --
------ 0
EU548038.1 --
------ 0
EU548036.1 --
------ 0
EU548035.1 --
------ 0
AF207720.1 --
------ 0
AB601576.1 --
------ 0
EU548040.1 --
------ 0
EU548039.1 --
------ 0
AF207721.1 --
------ 0
AF207724.1 --
------ 0
JX982499.1 --
------ 0
EU548041.1 --
------ 0
EU548045.1 --
------ 0
AF207725.1 --
------ 0
EU548046.1 --
------ 0
AF207714.1 --
------ 0
AF207713.1 --
------ 0
AF207712.1 --
------ 0
AY750628.1 --
------ 0
EF689084.1 --
------ 0
EF689085.1 --
------ 0
AB119070.1 --
------ 956
EF987742.1 --
------ 0
AB026105.1 --
------ 0
MW148603.1
TTACTTTGATAGAGTAAATCATAGAGGTTCAAACCCTCTTATTTCTAGAACTAAAGGAAT 3780
AB051263.1 --
------ 0
AF068544.1 --
------ 0

JX982502.1 --
------ 0
JX982501.1 --
------ 0

```
    JX982498.1          ------------------------------------------------------
------ 0
    JX982497.1          ------------------------------------------------------
------ 0
    JX982495.1          ------------------------------------------------------
------ 0
    JX982496.1          ------------------------------------------------------
------ 0
    JX982500.1          ------------------------------------------------------
------ 0
    EU548051.1          ------------------------------------------------------
------ 0
    EU548044.1          ------------------------------------------------------
------ 0
    EU548042.1          ------------------------------------------------------
------ 0
    EU548043.1          ------------------------------------------------------
------ 0
    EU548047.1          ------------------------------------------------------
------ 0
    EU548050.1          ------------------------------------------------------
------ 0
    EU548048.1          ------------------------------------------------------
------ 0
    EU548049.1          ------------------------------------------------------
------ 0
    AF207722.1          ------------------------------------------------------
------ 0
    EU548037.1          ------------------------------------------------------
------ 0
    AF207723.1          ------------------------------------------------------
------ 0
    EU548038.1          ------------------------------------------------------
------ 0
    EU548036.1          ------------------------------------------------------
------ 0
    EU548035.1          ------------------------------------------------------
------ 0
    AF207720.1          ------------------------------------------------------
------ 0
    AB601576.1          ------------------------------------------------------
------ 0
    EU548040.1          ------------------------------------------------------
------ 0
    EU548039.1          ------------------------------------------------------
------ 0
    AF207721.1          ------------------------------------------------------
------ 0
    AF207724.1          ------------------------------------------------------
------ 0
    JX982499.1          ------------------------------------------------------
------ 0
    EU548041.1          ------------------------------------------------------
------ 0
    EU548045.1          ------------------------------------------------------
------ 0
    AF207725.1          ------------------------------------------------------
------ 0
    EU548046.1          ------------------------------------------------------
------ 0
    AF207714.1          ------------------------------------------------------
------ 0
    AF207713.1          ------------------------------------------------------
------ 0
    AF207712.1          ------------------------------------------------------
------ 0
```

```
    AY750628.1      ------------------------------------------------------
------ 0
    EF689084.1      ------------------------------------------------------
------ 0
    EF689085.1      ------------------------------------------------------
------ 0
    AB119070.1      ------------------------------------------------------
------ 956
    EF987742.1      ------------------------------------------------------
------ 0
    AB026105.1      ------------------------------------------------------
------ 0
    MW148603.1
CGAACCTAATCCTAAGAACTCAAAAATCTTCGTGCTACCAAATTTACACCAAATTCTAAA   3840
    AB051263.1      ------------------------------------------------------
------ 0
    AF068544.1      ------------------------------------------------------
------ 0


    JX982502.1      ------------------------------------------------------
------ 0
    JX982501.1      ------------------------------------------------------
------ 0
    JX982498.1      ------------------------------------------------------
------ 0
    JX982497.1      ------------------------------------------------------
------ 0
    JX982495.1      ------------------------------------------------------
------ 0
    JX982496.1      ------------------------------------------------------
------ 0
    JX982500.1      ------------------------------------------------------
------ 0
    EU548051.1      ------------------------------------------------------
------ 0
    EU548044.1      ------------------------------------------------------
------ 0
    EU548042.1      ------------------------------------------------------
------ 0
    EU548043.1      ------------------------------------------------------
------ 0
    EU548047.1      ------------------------------------------------------
------ 0
    EU548050.1      ------------------------------------------------------
------ 0
    EU548048.1      ------------------------------------------------------
------ 0
    EU548049.1      ------------------------------------------------------
------ 0
    AF207722.1      ------------------------------------------------------
------ 0
    EU548037.1      ------------------------------------------------------
------ 0
    AF207723.1      ------------------------------------------------------
------ 0
    EU548038.1      ------------------------------------------------------
------ 0
    EU548036.1      ------------------------------------------------------
------ 0
    EU548035.1      ------------------------------------------------------
------ 0
    AF207720.1      ------------------------------------------------------
------ 0
    AB601576.1      ------------------------------------------------------
------ 0
```

```
   EU548040.1      ------------------------------------------------------
------ 0
   EU548039.1      ------------------------------------------------------
------ 0
   AF207721.1      ------------------------------------------------------
------ 0
   AF207724.1      ------------------------------------------------------
------ 0
   JX982499.1      ------------------------------------------------------
------ 0
   EU548041.1      ------------------------------------------------------
------ 0
   EU548045.1      ------------------------------------------------------
------ 0
   AF207725.1      ------------------------------------------------------
------ 0
   EU548046.1      ------------------------------------------------------
------ 0
   AF207714.1      ------------------------------------------------------
------ 0
   AF207713.1      ------------------------------------------------------
------ 0
   AF207712.1      ------------------------------------------------------
------ 0
   AY750628.1      ------------------------------------------------------
------ 0
   EF689084.1      ------------------------------------------------------
------ 0
   EF689085.1      ------------------------------------------------------
------ 0
   AB119070.1      ------------------------------------------------------
------ 956
   EF987742.1      ------------------------------------------------------
------ 0
   AB026105.1      ------------------------------------------------------
------ 0
   MW148603.1
GTAAGGTCAGCTAATTAAGCTATCGGGCCCATACCCCGAAAATGTTGGTTTATCCCCTTC   3900
   AB051263.1      ------------------------------------------------------
------ 0
   AF068544.1      ------------------------------------------------------
------ 0


   JX982502.1      ------------------------------------------------------
------ 0
   JX982501.1      ------------------------------------------------------
------ 0
   JX982498.1      ------------------------------------------------------
------ 0
   JX982497.1      ------------------------------------------------------
------ 0
   JX982495.1      ------------------------------------------------------
------ 0
   JX982496.1      ------------------------------------------------------
------ 0
   JX982500.1      ------------------------------------------------------
------ 0
   EU548051.1      ------------------------------------------------------
------ 0
   EU548044.1      ------------------------------------------------------
------ 0
   EU548042.1      ------------------------------------------------------
------ 0
   EU548043.1      ------------------------------------------------------
------ 0
```

EU548047.1 --
------ 0
EU548050.1 --
------ 0
EU548048.1 --
------ 0
EU548049.1 --
------ 0
AF207722.1 --
------ 0
EU548037.1 --
------ 0
AF207723.1 --
------ 0
EU548038.1 --
------ 0
EU548036.1 --
------ 0
EU548035.1 --
------ 0
AF207720.1 --
------ 0
AB601576.1 --
------ 0
EU548040.1 --
------ 0
EU548039.1 --
------ 0
AF207721.1 --
------ 0
AF207724.1 --
------ 0
JX982499.1 --
------ 0
EU548041.1 --
------ 0
EU548045.1 --
------ 0
AF207725.1 --
------ 0
EU548046.1 --
------ 0
AF207714.1 --
------ 0
AF207713.1 --
------ 0
AF207712.1 --
------ 0
AY750628.1 --------
ATTAAACCCCCTATTCTCACCATTATCATATTTACTATTATCTCAGGGACTA 52
EF689084.1 --
------ 0
EF689085.1 --
------ 0
AB119070.1 --
------ 956
EF987742.1 --
------ 0
AB026105.1 --
------ 0
MW148603.1
CCGTACTAATTAAACCCCCTATTCTCACCATTATCATATTTACTATTATCTCAGGGACTA 3960
AB051263.1 --
------ 0
AF068544.1 --
------ 0

```
   JX982502.1      ------------------------------------------------------
------ 0
   JX982501.1      ------------------------------------------------------
------ 0
   JX982498.1      ------------------------------------------------------
------ 0
   JX982497.1      ------------------------------------------------------
------ 0
   JX982495.1      ------------------------------------------------------
------ 0
   JX982496.1      ------------------------------------------------------
------ 0
   JX982500.1      ------------------------------------------------------
------ 0
   EU548051.1      ------------------------------------------------------
------ 0
   EU548044.1      ------------------------------------------------------
------ 0
   EU548042.1      ------------------------------------------------------
------ 0
   EU548043.1      ------------------------------------------------------
------ 0
   EU548047.1      ------------------------------------------------------
------ 0
   EU548050.1      ------------------------------------------------------
------ 0
   EU548048.1      ------------------------------------------------------
------ 0
   EU548049.1      ------------------------------------------------------
------ 0
   AF207722.1      ------------------------------------------------------
------ 0
   EU548037.1      ------------------------------------------------------
------ 0
   AF207723.1      ------------------------------------------------------
------ 0
   EU548038.1      ------------------------------------------------------
------ 0
   EU548036.1      ------------------------------------------------------
------ 0
   EU548035.1      ------------------------------------------------------
------ 0
   AF207720.1      ------------------------------------------------------
------ 0
   AB601576.1      ------------------------------------------------------
------ 0
   EU548040.1      ------------------------------------------------------
------ 0
   EU548039.1      ------------------------------------------------------
------ 0
   AF207721.1      ------------------------------------------------------
------ 0
   AF207724.1      ------------------------------------------------------
------ 0
   JX982499.1      ------------------------------------------------------
------ 0
   EU548041.1      ------------------------------------------------------
------ 0
   EU548045.1      ------------------------------------------------------
------ 0
   AF207725.1      ------------------------------------------------------
------ 0
   EU548046.1      ------------------------------------------------------
------ 0
```

```
   AF207714.1      ------------------------------------------------------
------ 0
   AF207713.1      ------------------------------------------------------
------ 0
   AF207712.1      ------------------------------------------------------
------ 0
   AY750628.1
TCATAGTACTAATAAGCTCCCACTGATTAACAATTTGAATCGGATTTGAAATAAACATAC   112
   EF689084.1      ------------------------------------------------------
------ 0
   EF689085.1      ------------------------------------------------------
------ 0
   AB119070.1      ------------------------------------------------------
------ 956
   EF987742.1      ------------------------------------------------------
------ 0
   AB026105.1      ------------------------------------------------------
------ 0
   MW148603.1
TCATAGTACTAATAAGCTCCCACTGATTAACAATTTGAATCGGATTTGAAATAAACATAC   4020
   AB051263.1      ------------------------------------------------------
------ 0
   AF068544.1      ------------------------------------------------------
------ 0


   JX982502.1      ------------------------------------------------------
------ 0
   JX982501.1      ------------------------------------------------------
------ 0
   JX982498.1      ------------------------------------------------------
------ 0
   JX982497.1      ------------------------------------------------------
------ 0
   JX982495.1      ------------------------------------------------------
------ 0
   JX982496.1      ------------------------------------------------------
------ 0
   JX982500.1      ------------------------------------------------------
------ 0
   EU548051.1      ------------------------------------------------------
------ 0
   EU548044.1      ------------------------------------------------------
------ 0
   EU548042.1      ------------------------------------------------------
------ 0
   EU548043.1      ------------------------------------------------------
------ 0
   EU548047.1      ------------------------------------------------------
------ 0
   EU548050.1      ------------------------------------------------------
------ 0
   EU548048.1      ------------------------------------------------------
------ 0
   EU548049.1      ------------------------------------------------------
------ 0
   AF207722.1      ------------------------------------------------------
------ 0
   EU548037.1      ------------------------------------------------------
------ 0
   AF207723.1      ------------------------------------------------------
------ 0
   EU548038.1      ------------------------------------------------------
------ 0
   EU548036.1      ------------------------------------------------------
------ 0
```

```
   EU548035.1      ------------------------------------------------------
------ 0
   AF207720.1      ------------------------------------------------------
------ 0
   AB601576.1      ------------------------------------------------------
------ 0
   EU548040.1      ------------------------------------------------------
------ 0
   EU548039.1      ------------------------------------------------------
------ 0
   AF207721.1      ------------------------------------------------------
------ 0
   AF207724.1      ------------------------------------------------------
------ 0
   JX982499.1      ------------------------------------------------------
------ 0
   EU548041.1      ------------------------------------------------------
------ 0
   EU548045.1      ------------------------------------------------------
------ 0
   AF207725.1      ------------------------------------------------------
------ 0
   EU548046.1      ------------------------------------------------------
------ 0
   AF207714.1      ------------------------------------------------------
------ 0
   AF207713.1      ------------------------------------------------------
------ 0
   AF207712.1      ------------------------------------------------------
------ 0
   AY750628.1
TAGCCATCATTCCCATCCTAATAAAAAAATTCAGCCCACGAGCAATTGAAGCAGCCACAA   172
   EF689084.1      ------------------------------------------------------
------ 0
   EF689085.1      ------------------------------------------------------
------ 0
   AB119070.1      ------------------------------------------------------
------ 956
   EF987742.1      ------------------------------------------------------
------ 0
   AB026105.1      ------------------------------------------------------
------ 0
   MW148603.1
TAGCCATCATTCCCATCCTAATAAAAAAATTCAGCCCACGAGCAATTGAAGCAGCCACAA   4080
   AB051263.1      ------------------------------------------------------
------ 0
   AF068544.1      ------------------------------------------------------
------ 0


   JX982502.1      ------------------------------------------------------
------ 0
   JX982501.1      ------------------------------------------------------
------ 0
   JX982498.1      ------------------------------------------------------
------ 0
   JX982497.1      ------------------------------------------------------
------ 0
   JX982495.1      ------------------------------------------------------
------ 0
   JX982496.1      ------------------------------------------------------
------ 0
   JX982500.1      ------------------------------------------------------
------ 0
   EU548051.1      ------------------------------------------------------
------ 0
```

```
    EU548044.1        ------------------------------------------------------
------ 0
    EU548042.1        ------------------------------------------------------
------ 0
    EU548043.1        ------------------------------------------------------
------ 0
    EU548047.1        ------------------------------------------------------
------ 0
    EU548050.1        ------------------------------------------------------
------ 0
    EU548048.1        ------------------------------------------------------
------ 0
    EU548049.1        ------------------------------------------------------
------ 0
    AF207722.1        ------------------------------------------------------
------ 0
    EU548037.1        ------------------------------------------------------
------ 0
    AF207723.1        ------------------------------------------------------
------ 0
    EU548038.1        ------------------------------------------------------
------ 0
    EU548036.1        ------------------------------------------------------
------ 0
    EU548035.1        ------------------------------------------------------
------ 0
    AF207720.1        ------------------------------------------------------
------ 0
    AB601576.1        ------------------------------------------------------
------ 0
    EU548040.1        ------------------------------------------------------
------ 0
    EU548039.1        ------------------------------------------------------
------ 0
    AF207721.1        ------------------------------------------------------
------ 0
    AF207724.1        ------------------------------------------------------
------ 0
    JX982499.1        ------------------------------------------------------
------ 0
    EU548041.1        ------------------------------------------------------
------ 0
    EU548045.1        ------------------------------------------------------
------ 0
    AF207725.1        ------------------------------------------------------
------ 0
    EU548046.1        ------------------------------------------------------
------ 0
    AF207714.1        ------------------------------------------------------
------ 0
    AF207713.1        ------------------------------------------------------
------ 0
    AF207712.1        ------------------------------------------------------
------ 0
    AY750628.1
AATATTTCCTCACCCAAGCCACTGCATCTATACTCCTAATACTAGGAATTATCATAAACC   232
    EF689084.1        ------------------------------------------------------
------ 0
    EF689085.1        ------------------------------------------------------
------ 0
    AB119070.1        ------------------------------------------------------
------ 956
    EF987742.1        ------------------------------------------------------
------ 0
    AB026105.1        ------------------------------------------------------
------ 0
```

MW148603.1
AATATTTCCTCACCCAAGCCACTGCATCTATACTCCTAATACTAGGAATTATCATAAACC 4140
AB051263.1 --
------ 0
AF068544.1 --
------ 0

JX982502.1 --
------ 0
JX982501.1 --
------ 0
JX982498.1 --
------ 0
JX982497.1 --
------ 0
JX982495.1 --
------ 0
JX982496.1 --
------ 0
JX982500.1 --
------ 0
EU548051.1 --
------ 0
EU548044.1 --
------ 0
EU548042.1 --
------ 0
EU548043.1 --
------ 0
EU548047.1 --
------ 0
EU548050.1 --
------ 0
EU548048.1 --
------ 0
EU548049.1 --
------ 0
AF207722.1 --
------ 0
EU548037.1 --
------ 0
AF207723.1 --
------ 0
EU548038.1 --
------ 0
EU548036.1 --
------ 0
EU548035.1 --
------ 0
AF207720.1 --
------ 0
AB601576.1 --
------ 0
EU548040.1 --
------ 0
EU548039.1 --
------ 0
AF207721.1 --
------ 0
AF207724.1 --
------ 0
JX982499.1 --
------ 0
EU548041.1 --
------ 0

```
   EU548045.1      ------------------------------------------------------
------ 0
   AF207725.1      ------------------------------------------------------
------ 0
   EU548046.1      ------------------------------------------------------
------ 0
   AF207714.1      ------------------------------------------------------
------ 0
   AF207713.1      ------------------------------------------------------
------ 0
   AF207712.1      ------------------------------------------------------
------ 0
   AY750628.1
TATTATTAACAGGACAATGAACAGCCCTAAACATCCTAAACCCAATCGTATCTAACATAA   292
   EF689084.1      ------------------------------------------------------
------ 0
   EF689085.1      ------------------------------------------------------
------ 0
   AB119070.1      ------------------------------------------------------
------ 956
   EF987742.1      ------------------------------------------------------
------ 0
   AB026105.1      ------------------------------------------------------
------ 0
   MW148603.1
TATTATTAACAGGACAATGAACAGCCCTAAACATCCTAAACCCAATCGTATCTAACATAA   4200
   AB051263.1      ------------------------------------------------------
------ 0
   AF068544.1      ------------------------------------------------------
------ 0


   JX982502.1      ------------------------------------------------------
------ 0
   JX982501.1      ------------------------------------------------------
------ 0
   JX982498.1      ------------------------------------------------------
------ 0
   JX982497.1      ------------------------------------------------------
------ 0
   JX982495.1      ------------------------------------------------------
------ 0
   JX982496.1      ------------------------------------------------------
------ 0
   JX982500.1      ------------------------------------------------------
------ 0
   EU548051.1      ------------------------------------------------------
------ 0
   EU548044.1      ------------------------------------------------------
------ 0
   EU548042.1      ------------------------------------------------------
------ 0
   EU548043.1      ------------------------------------------------------
------ 0
   EU548047.1      ------------------------------------------------------
------ 0
   EU548050.1      ------------------------------------------------------
------ 0
   EU548048.1      ------------------------------------------------------
------ 0
   EU548049.1      ------------------------------------------------------
------ 0
   AF207722.1      ------------------------------------------------------
------ 0
   EU548037.1      ------------------------------------------------------
------ 0
```

AF207723.1 --
------ 0
EU548038.1 --
------ 0
EU548036.1 --
------ 0
EU548035.1 --
------ 0
AF207720.1 --
------ 0
AB601576.1 --
------ 0
EU548040.1 --
------ 0
EU548039.1 --
------ 0
AF207721.1 --
------ 0
AF207724.1 --
------ 0
JX982499.1 --
------ 0
EU548041.1 --
------ 0
EU548045.1 --
------ 0
AF207725.1 --
------ 0
EU548046.1 --
------ 0
AF207714.1 --
------ 0
AF207713.1 --
------ 0
AF207712.1 --
------ 0
AY750628.1
TAACAGTAGCCTTATCAATAAAACTAGGATTATCACCTTTCCACTTCTGAGTACCCGAAG 352
EF689084.1 --
------ 0
EF689085.1 --
------ 0
AB119070.1 --
------ 956
EF987742.1 --
------ 0
AB026105.1 --
------ 0
MW148603.1
TAACAGTAGCCTTATCAATAAAACTAGGATTATCACCTTTCCACTTCTGAGTACCCGAAG 4260
AB051263.1 --
------ 0
AF068544.1 --
------ 0

JX982502.1 --
------ 0
JX982501.1 --
------ 0
JX982498.1 --
------ 0
JX982497.1 --
------ 0
JX982495.1 --
------ 0

```
    JX982496.1      ------------------------------------------------------
------ 0
    JX982500.1      ------------------------------------------------------
------ 0
    EU548051.1      ------------------------------------------------------
------ 0
    EU548044.1      ------------------------------------------------------
------ 0
    EU548042.1      ------------------------------------------------------
------ 0
    EU548043.1      ------------------------------------------------------
------ 0
    EU548047.1      ------------------------------------------------------
------ 0
    EU548050.1      ------------------------------------------------------
------ 0
    EU548048.1      ------------------------------------------------------
------ 0
    EU548049.1      ------------------------------------------------------
------ 0
    AF207722.1      ------------------------------------------------------
------ 0
    EU548037.1      ------------------------------------------------------
------ 0
    AF207723.1      ------------------------------------------------------
------ 0
    EU548038.1      ------------------------------------------------------
------ 0
    EU548036.1      ------------------------------------------------------
------ 0
    EU548035.1      ------------------------------------------------------
------ 0
    AF207720.1      ------------------------------------------------------
------ 0
    AB601576.1      ------------------------------------------------------
------ 0
    EU548040.1      ------------------------------------------------------
------ 0
    EU548039.1      ------------------------------------------------------
------ 0
    AF207721.1      ------------------------------------------------------
------ 0
    AF207724.1      ------------------------------------------------------
------ 0
    JX982499.1      ------------------------------------------------------
------ 0
    EU548041.1      ------------------------------------------------------
------ 0
    EU548045.1      ------------------------------------------------------
------ 0
    AF207725.1      ------------------------------------------------------
------ 0
    EU548046.1      ------------------------------------------------------
------ 0
    AF207714.1      ------------------------------------------------------
------ 0
    AF207713.1      ------------------------------------------------------
------ 0
    AF207712.1      ------------------------------------------------------
------ 0
    AY750628.1
TAACCCAAGGAGTCCCATTAATGTCAGGAATAATCCTACTAACTTGGCAAAAAATTGCCC    412
    EF689084.1      ------------------------------------------------------
------ 0
    EF689085.1      ------------------------------------------------------
------ 0
```

```
   AB119070.1      ------------------------------------------------------
------ 956
   EF987742.1      ------------------------------------------------------
------ 0
   AB026105.1      ------------------------------------------------------
------ 0
   MW148603.1
TAACCCAAGGAGTCCCATTAATGTCAGGAATAATCCTACTAACTTGGCAAAAAATTGCCC   4320
   AB051263.1      ------------------------------------------------------
------ 0
   AF068544.1      ------------------------------------------------------
------ 0


   JX982502.1      ------------------------------------------------------
------ 0
   JX982501.1      ------------------------------------------------------
------ 0
   JX982498.1      ------------------------------------------------------
------ 0
   JX982497.1      ------------------------------------------------------
------ 0
   JX982495.1      ------------------------------------------------------
------ 0
   JX982496.1      ------------------------------------------------------
------ 0
   JX982500.1      ------------------------------------------------------
------ 0
   EU548051.1      ------------------------------------------------------
------ 0
   EU548044.1      ------------------------------------------------------
------ 0
   EU548042.1      ------------------------------------------------------
------ 0
   EU548043.1      ------------------------------------------------------
------ 0
   EU548047.1      ------------------------------------------------------
------ 0
   EU548050.1      ------------------------------------------------------
------ 0
   EU548048.1      ------------------------------------------------------
------ 0
   EU548049.1      ------------------------------------------------------
------ 0
   AF207722.1      ------------------------------------------------------
------ 0
   EU548037.1      ------------------------------------------------------
------ 0
   AF207723.1      ------------------------------------------------------
------ 0
   EU548038.1      ------------------------------------------------------
------ 0
   EU548036.1      ------------------------------------------------------
------ 0
   EU548035.1      ------------------------------------------------------
------ 0
   AF207720.1      ------------------------------------------------------
------ 0
   AB601576.1      ------------------------------------------------------
------ 0
   EU548040.1      ------------------------------------------------------
------ 0
   EU548039.1      ------------------------------------------------------
------ 0
   AF207721.1      ------------------------------------------------------
------ 0
```

```
   AF207724.1      ------------------------------------------------------
------ 0
   JX982499.1      ------------------------------------------------------
------ 0
   EU548041.1      ------------------------------------------------------
------ 0
   EU548045.1      ------------------------------------------------------
------ 0
   AF207725.1      ------------------------------------------------------
------ 0
   EU548046.1      ------------------------------------------------------
------ 0
   AF207714.1      ------------------------------------------------------
------ 0
   AF207713.1      ------------------------------------------------------
------ 0
   AF207712.1      ------------------------------------------------------
------ 0
   AY750628.1
CCCTATCTGTCCTGTACCAAATAGCCCCCTCCATAAACACACACTTATTAATAACTATAG   472
   EF689084.1      ------------------------------------------------------
------ 0
   EF689085.1      ------------------------------------------------------
------ 0
   AB119070.1      ------------------------------------------------------
------ 956
   EF987742.1      ------------------------------------------------------
------ 0
   AB026105.1      ------------------------------------------------------
------ 0
   MW148603.1
CCCTATCTGTCCTGTACCAAATAGCCCCCTCCATAAACACACACTTATTAATAACTATAG   4380
   AB051263.1      ------------------------------------------------------
------ 0
   AF068544.1      ------------------------------------------------------
------ 0


   JX982502.1      ------------------------------------------------------
------ 0
   JX982501.1      ------------------------------------------------------
------ 0
   JX982498.1      ------------------------------------------------------
------ 0
   JX982497.1      ------------------------------------------------------
------ 0
   JX982495.1      ------------------------------------------------------
------ 0
   JX982496.1      ------------------------------------------------------
------ 0
   JX982500.1      ------------------------------------------------------
------ 0
   EU548051.1      ------------------------------------------------------
------ 0
   EU548044.1      ------------------------------------------------------
------ 0
   EU548042.1      ------------------------------------------------------
------ 0
   EU548043.1      ------------------------------------------------------
------ 0
   EU548047.1      ------------------------------------------------------
------ 0
   EU548050.1      ------------------------------------------------------
------ 0
   EU548048.1      ------------------------------------------------------
------ 0
```

EU548049.1 --
------ 0
AF207722.1 --
------ 0
EU548037.1 --
------ 0
AF207723.1 --
------ 0
EU548038.1 --
------ 0
EU548036.1 --
------ 0
EU548035.1 --
------ 0
AF207720.1 --
------ 0
AB601576.1 --
------ 0
EU548040.1 --
------ 0
EU548039.1 --
------ 0
AF207721.1 --
------ 0
AF207724.1 --
------ 0
JX982499.1 --
------ 0
EU548041.1 --
------ 0
EU548045.1 --
------ 0
AF207725.1 --
------ 0
EU548046.1 --
------ 0
AF207714.1 --
------ 0
AF207713.1 --
------ 0
AF207712.1 --
------ 0
AY750628.1
CATTCATATCTGTCTTAATTGGAGGATGGGGAGGCCTTAACCAAACACAACTACGAAAAA 532
EF689084.1 --
------ 0
EF689085.1 --
------ 0
AB119070.1 --
------ 956
EF987742.1 --
------ 0
AB026105.1 --
------ 0
MW148603.1
CATTCATATCTGTCTTAATTGGAGGATGGGGAGGCCTTAACCAAACACAACTACGAAAAA 4440
AB051263.1 --
------ 0
AF068544.1 --
------ 0

JX982502.1 --
------ 0
JX982501.1 --
------ 0

```
    JX982498.1        ------------------------------------------------------
------ 0
    JX982497.1        ------------------------------------------------------
------ 0
    JX982495.1        ------------------------------------------------------
------ 0
    JX982496.1        ------------------------------------------------------
------ 0
    JX982500.1        ------------------------------------------------------
------ 0
    EU548051.1        ------------------------------------------------------
------ 0
    EU548044.1        ------------------------------------------------------
------ 0
    EU548042.1        ------------------------------------------------------
------ 0
    EU548043.1        ------------------------------------------------------
------ 0
    EU548047.1        ------------------------------------------------------
------ 0
    EU548050.1        ------------------------------------------------------
------ 0
    EU548048.1        ------------------------------------------------------
------ 0
    EU548049.1        ------------------------------------------------------
------ 0
    AF207722.1        ------------------------------------------------------
------ 0
    EU548037.1        ------------------------------------------------------
------ 0
    AF207723.1        ------------------------------------------------------
------ 0
    EU548038.1        ------------------------------------------------------
------ 0
    EU548036.1        ------------------------------------------------------
------ 0
    EU548035.1        ------------------------------------------------------
------ 0
    AF207720.1        ------------------------------------------------------
------ 0
    AB601576.1        ------------------------------------------------------
------ 0
    EU548040.1        ------------------------------------------------------
------ 0
    EU548039.1        ------------------------------------------------------
------ 0
    AF207721.1        ------------------------------------------------------
------ 0
    AF207724.1        ------------------------------------------------------
------ 0
    JX982499.1        ------------------------------------------------------
------ 0
    EU548041.1        ------------------------------------------------------
------ 0
    EU548045.1        ------------------------------------------------------
------ 0
    AF207725.1        ------------------------------------------------------
------ 0
    EU548046.1        ------------------------------------------------------
------ 0
    AF207714.1        ------------------------------------------------------
------ 0
    AF207713.1        ------------------------------------------------------
------ 0
    AF207712.1        ------------------------------------------------------
------ 0
```

```
    AY750628.1
TTCTAGCCTACTCATCAATCGCACACATAGGATGAATGATCGCCGTAACAACATATAACC   592
    EF689084.1      ------------------------------------------------------
------ 0
    EF689085.1      ------------------------------------------------------
------ 0
    AB119070.1      ------------------------------------------------------
------ 956
    EF987742.1      ------------------------------------------------------
------ 0
    AB026105.1      ------------------------------------------------------
------ 0
    MW148603.1
TTCTAGCCTACTCATCAATCGCACACATAGGATGAATGATCGCCGTAACAACATATAACC   4500
    AB051263.1      ------------------------------------------------------
------ 0
    AF068544.1      ------------------------------------------------------
------ 0


    JX982502.1      ------------------------------------------------------
------ 0
    JX982501.1      ------------------------------------------------------
------ 0
    JX982498.1      ------------------------------------------------------
------ 0
    JX982497.1      ------------------------------------------------------
------ 0
    JX982495.1      ------------------------------------------------------
------ 0
    JX982496.1      ------------------------------------------------------
------ 0
    JX982500.1      ------------------------------------------------------
------ 0
    EU548051.1      ------------------------------------------------------
------ 0
    EU548044.1      ------------------------------------------------------
------ 0
    EU548042.1      ------------------------------------------------------
------ 0
    EU548043.1      ------------------------------------------------------
------ 0
    EU548047.1      ------------------------------------------------------
------ 0
    EU548050.1      ------------------------------------------------------
------ 0
    EU548048.1      ------------------------------------------------------
------ 0
    EU548049.1      ------------------------------------------------------
------ 0
    AF207722.1      ------------------------------------------------------
------ 0
    EU548037.1      ------------------------------------------------------
------ 0
    AF207723.1      ------------------------------------------------------
------ 0
    EU548038.1      ------------------------------------------------------
------ 0
    EU548036.1      ------------------------------------------------------
------ 0
    EU548035.1      ------------------------------------------------------
------ 0
    AF207720.1      ------------------------------------------------------
------ 0
    AB601576.1      ------------------------------------------------------
------ 0
```

```
    EU548040.1       ------------------------------------------------------
------ 0
    EU548039.1       ------------------------------------------------------
------ 0
    AF207721.1       ------------------------------------------------------
------ 0
    AF207724.1       ------------------------------------------------------
------ 0
    JX982499.1       ------------------------------------------------------
------ 0
    EU548041.1       ------------------------------------------------------
------ 0
    EU548045.1       ------------------------------------------------------
------ 0
    AF207725.1       ------------------------------------------------------
------ 0
    EU548046.1       ------------------------------------------------------
------ 0
    AF207714.1       ------------------------------------------------------
------ 0
    AF207713.1       ------------------------------------------------------
------ 0
    AF207712.1       ------------------------------------------------------
------ 0
    AY750628.1
CAACCCTGATATTACTAAACCTCACAATTTACATTATAATAACACTAGGAACATTCATAC   652
    EF689084.1       ------------------------------------------------------
------ 0
    EF689085.1       ------------------------------------------------------
------ 0
    AB119070.1       ------------------------------------------------------
------ 956
    EF987742.1       ------------------------------------------------------
------ 0
    AB026105.1       ------------------------------------------------------
------ 0
    MW148603.1
CAACCCTGATATTACTAAACCTCACAATTTACATTATAATAACACTAGGAACATTCATAC   4560
    AB051263.1       ------------------------------------------------------
------ 0
    AF068544.1       ------------------------------------------------------
------ 0


    JX982502.1       ------------------------------------------------------
------ 0
    JX982501.1       ------------------------------------------------------
------ 0
    JX982498.1       ------------------------------------------------------
------ 0
    JX982497.1       ------------------------------------------------------
------ 0
    JX982495.1       ------------------------------------------------------
------ 0
    JX982496.1       ------------------------------------------------------
------ 0
    JX982500.1       ------------------------------------------------------
------ 0
    EU548051.1       ------------------------------------------------------
------ 0
    EU548044.1       ------------------------------------------------------
------ 0
    EU548042.1       ------------------------------------------------------
------ 0
    EU548043.1       ------------------------------------------------------
------ 0
```

EU548047.1 --
------ 0
EU548050.1 --
------ 0
EU548048.1 --
------ 0
EU548049.1 --
------ 0
AF207722.1 --
------ 0
EU548037.1 --
------ 0
AF207723.1 --
------ 0
EU548038.1 --
------ 0
EU548036.1 --
------ 0
EU548035.1 --
------ 0
AF207720.1 --
------ 0
AB601576.1 --
------ 0
EU548040.1 --
------ 0
EU548039.1 --
------ 0
AF207721.1 --
------ 0
AF207724.1 --
------ 0
JX982499.1 --
------ 0
EU548041.1 --
------ 0
EU548045.1 --
------ 0
AF207725.1 --
------ 0
EU548046.1 --
------ 0
AF207714.1 --
------ 0
AF207713.1 --
------ 0
AF207712.1 --
------ 0
AY750628.1
TATTTTCACTCAGCTCATCTACAACTACACTATCACTATCCCTTACATGAAATAAACTCC 712
EF689084.1 --
------ 0
EF689085.1 --
------ 0
AB119070.1 --
------ 956
EF987742.1 --
------ 0
AB026105.1 --
------ 0
MW148603.1
TATTTTCATTCAGCTCATCTACAACTACACTATCACTATCCCTTACATGAAATAAACTCC 4620
AB051263.1 --
------ 0
AF068544.1 --
------ 0

```
    JX982502.1        ------------------------------------------------------
------ 0
    JX982501.1        ------------------------------------------------------
------ 0
    JX982498.1        ------------------------------------------------------
------ 0
    JX982497.1        ------------------------------------------------------
------ 0
    JX982495.1        ------------------------------------------------------
------ 0
    JX982496.1        ------------------------------------------------------
------ 0
    JX982500.1        ------------------------------------------------------
------ 0
    EU548051.1        ------------------------------------------------------
------ 0
    EU548044.1        ------------------------------------------------------
------ 0
    EU548042.1        ------------------------------------------------------
------ 0
    EU548043.1        ------------------------------------------------------
------ 0
    EU548047.1        ------------------------------------------------------
------ 0
    EU548050.1        ------------------------------------------------------
------ 0
    EU548048.1        ------------------------------------------------------
------ 0
    EU548049.1        ------------------------------------------------------
------ 0
    AF207722.1        ------------------------------------------------------
------ 0
    EU548037.1        ------------------------------------------------------
------ 0
    AF207723.1        ------------------------------------------------------
------ 0
    EU548038.1        ------------------------------------------------------
------ 0
    EU548036.1        ------------------------------------------------------
------ 0
    EU548035.1        ------------------------------------------------------
------ 0
    AF207720.1        ------------------------------------------------------
------ 0
    AB601576.1        ------------------------------------------------------
------ 0
    EU548040.1        ------------------------------------------------------
------ 0
    EU548039.1        ------------------------------------------------------
------ 0
    AF207721.1        ------------------------------------------------------
------ 0
    AF207724.1        ------------------------------------------------------
------ 0
    JX982499.1        ------------------------------------------------------
------ 0
    EU548041.1        ------------------------------------------------------
------ 0
    EU548045.1        ------------------------------------------------------
------ 0
    AF207725.1        ------------------------------------------------------
------ 0
    EU548046.1        ------------------------------------------------------
------ 0
```

```
   AF207714.1      ------------------------------------------------------
------ 0
   AF207713.1      ------------------------------------------------------
------ 0
   AF207712.1      ------------------------------------------------------
------ 0
   AY750628.1
CACTAATCACCTCACTAATCCTTACCATCATACTATCACTAGGAGGCTTACCACCACTTT   772
   EF689084.1      ------------------------------------------------------
------ 0
   EF689085.1      ------------------------------------------------------
------ 0
   AB119070.1      ------------------------------------------------------
------ 956
   EF987742.1      ------------------------------------------------------
------ 0
   AB026105.1      ------------------------------------------------------
------ 0
   MW148603.1
CACTAATCACCTCACTAATCCTTACCATCATACTATCACTAGGAGGCTTACCACCACTTT   4680
   AB051263.1      ------------------------------------------------------
------ 0
   AF068544.1      ------------------------------------------------------
------ 0


   JX982502.1      ------------------------------------------------------
------ 0
   JX982501.1      ------------------------------------------------------
------ 0
   JX982498.1      ------------------------------------------------------
------ 0
   JX982497.1      ------------------------------------------------------
------ 0
   JX982495.1      ------------------------------------------------------
------ 0
   JX982496.1      ------------------------------------------------------
------ 0
   JX982500.1      ------------------------------------------------------
------ 0
   EU548051.1      ------------------------------------------------------
------ 0
   EU548044.1      ------------------------------------------------------
------ 0
   EU548042.1      ------------------------------------------------------
------ 0
   EU548043.1      ------------------------------------------------------
------ 0
   EU548047.1      ------------------------------------------------------
------ 0
   EU548050.1      ------------------------------------------------------
------ 0
   EU548048.1      ------------------------------------------------------
------ 0
   EU548049.1      ------------------------------------------------------
------ 0
   AF207722.1      ------------------------------------------------------
------ 0
   EU548037.1      ------------------------------------------------------
------ 0
   AF207723.1      ------------------------------------------------------
------ 0
   EU548038.1      ------------------------------------------------------
------ 0
   EU548036.1      ------------------------------------------------------
------ 0
```

```
   EU548035.1      ------------------------------------------------------
------ 0
   AF207720.1      ------------------------------------------------------
------ 0
   AB601576.1      ------------------------------------------------------
------ 0
   EU548040.1      ------------------------------------------------------
------ 0
   EU548039.1      ------------------------------------------------------
------ 0
   AF207721.1      ------------------------------------------------------
------ 0
   AF207724.1      ------------------------------------------------------
------ 0
   JX982499.1      ------------------------------------------------------
------ 0
   EU548041.1      ------------------------------------------------------
------ 0
   EU548045.1      ------------------------------------------------------
------ 0
   AF207725.1      ------------------------------------------------------
------ 0
   EU548046.1      ------------------------------------------------------
------ 0
   AF207714.1      ------------------------------------------------------
------ 0
   AF207713.1      ------------------------------------------------------
------ 0
   AF207712.1      ------------------------------------------------------
------ 0
   AY750628.1
CAGGCTTCGTACCCAAATGAATAATCATCCACGAACTCACAAAAAACAACATAATTACTG   832
   EF689084.1      ------------------------------------------------------
------ 0
   EF689085.1      ------------------------------------------------------
------ 0
   AB119070.1      ------------------------------------------------------
------ 956
   EF987742.1      ------------------------------------------------------
------ 0
   AB026105.1      ------------------------------------------------------
------ 0
   MW148603.1
CAGGCTTCGTACCCAAATGAATAATCATCCACGAACTCACAAAAAACAACATAATTACTG   4740
   AB051263.1      ------------------------------------------------------
------ 0
   AF068544.1      ------------------------------------------------------
------ 0


   JX982502.1      ------------------------------------------------------
------ 0
   JX982501.1      ------------------------------------------------------
------ 0
   JX982498.1      ------------------------------------------------------
------ 0
   JX982497.1      ------------------------------------------------------
------ 0
   JX982495.1      ------------------------------------------------------
------ 0
   JX982496.1      ------------------------------------------------------
------ 0
   JX982500.1      ------------------------------------------------------
------ 0
   EU548051.1      ------------------------------------------------------
------ 0
```

```
    EU548044.1       ------------------------------------------------------
------ 0
    EU548042.1       ------------------------------------------------------
------ 0
    EU548043.1       ------------------------------------------------------
------ 0
    EU548047.1       ------------------------------------------------------
------ 0
    EU548050.1       ------------------------------------------------------
------ 0
    EU548048.1       ------------------------------------------------------
------ 0
    EU548049.1       ------------------------------------------------------
------ 0
    AF207722.1       ------------------------------------------------------
------ 0
    EU548037.1       ------------------------------------------------------
------ 0
    AF207723.1       ------------------------------------------------------
------ 0
    EU548038.1       ------------------------------------------------------
------ 0
    EU548036.1       ------------------------------------------------------
------ 0
    EU548035.1       ------------------------------------------------------
------ 0
    AF207720.1       ------------------------------------------------------
------ 0
    AB601576.1       ------------------------------------------------------
------ 0
    EU548040.1       ------------------------------------------------------
------ 0
    EU548039.1       ------------------------------------------------------
------ 0
    AF207721.1       ------------------------------------------------------
------ 0
    AF207724.1       ------------------------------------------------------
------ 0
    JX982499.1       ------------------------------------------------------
------ 0
    EU548041.1       ------------------------------------------------------
------ 0
    EU548045.1       ------------------------------------------------------
------ 0
    AF207725.1       ------------------------------------------------------
------ 0
    EU548046.1       ------------------------------------------------------
------ 0
    AF207714.1       ------------------------------------------------------
------ 0
    AF207713.1       ------------------------------------------------------
------ 0
    AF207712.1       ------------------------------------------------------
------ 0
    AY750628.1
CAGCAATATTCATAACAATCACAGCCCTACTAAACTTATACTTTTACATACGACTAACAT   892
    EF689084.1       ------------------------------------------------------
------ 0
    EF689085.1       ------------------------------------------------------
------ 0
    AB119070.1       ------------------------------------------------------
------ 956
    EF987742.1       ------------------------------------------------------
------ 0
    AB026105.1       ------------------------------------------------------
------ 0
```

```
    MW148603.1
CAGCAATATTCATAACAATCACAGCCCTACTAAACTTATACTTTTACATACGACTAACAT   4800
    AB051263.1      ------------------------------------------------------
------ 0
    AF068544.1      ------------------------------------------------------
------ 0


    JX982502.1      ------------------------------------------------------
------ 0
    JX982501.1      ------------------------------------------------------
------ 0
    JX982498.1      ------------------------------------------------------
------ 0
    JX982497.1      ------------------------------------------------------
------ 0
    JX982495.1      ------------------------------------------------------
------ 0
    JX982496.1      ------------------------------------------------------
------ 0
    JX982500.1      ------------------------------------------------------
------ 0
    EU548051.1      ------------------------------------------------------
------ 0
    EU548044.1      ------------------------------------------------------
------ 0
    EU548042.1      ------------------------------------------------------
------ 0
    EU548043.1      ------------------------------------------------------
------ 0
    EU548047.1      ------------------------------------------------------
------ 0
    EU548050.1      ------------------------------------------------------
------ 0
    EU548048.1      ------------------------------------------------------
------ 0
    EU548049.1      ------------------------------------------------------
------ 0
    AF207722.1      ------------------------------------------------------
------ 0
    EU548037.1      ------------------------------------------------------
------ 0
    AF207723.1      ------------------------------------------------------
------ 0
    EU548038.1      ------------------------------------------------------
------ 0
    EU548036.1      ------------------------------------------------------
------ 0
    EU548035.1      ------------------------------------------------------
------ 0
    AF207720.1      ------------------------------------------------------
------ 0
    AB601576.1      ------------------------------------------------------
------ 0
    EU548040.1      ------------------------------------------------------
------ 0
    EU548039.1      ------------------------------------------------------
------ 0
    AF207721.1      ------------------------------------------------------
------ 0
    AF207724.1      ------------------------------------------------------
------ 0
    JX982499.1      ------------------------------------------------------
------ 0
    EU548041.1      ------------------------------------------------------
------ 0
```

```
    EU548045.1      ------------------------------------------------------
------ 0
    AF207725.1      ------------------------------------------------------
------ 0
    EU548046.1      ------------------------------------------------------
------ 0
    AF207714.1      ------------------------------------------------------
------ 0
    AF207713.1      ------------------------------------------------------
------ 0
    AF207712.1      ------------------------------------------------------
------ 0
    AY750628.1
ACGCAACAGCACTAACCTTATTCCCCTCAACAAATAACATAAAAATAAAATGGCAATTTG   952
    EF689084.1      ------------------------------------------------------
------ 0
    EF689085.1      ------------------------------------------------------
------ 0
    AB119070.1      ------------------------------------------------------
------ 956
    EF987742.1      ------------------------------------------------------
------ 0
    AB026105.1      ------------------------------------------------------
------ 0
    MW148603.1
ACGCAACAGCACTAACCTTATTCCCCTCAACAAATAACATAAAAATAAAATGGCAATTTG   4860
    AB051263.1      ------------------------------------------------------
------ 0
    AF068544.1      ------------------------------------------------------
------ 0


    JX982502.1      ------------------------------------------------------
------ 0
    JX982501.1      ------------------------------------------------------
------ 0
    JX982498.1      ------------------------------------------------------
------ 0
    JX982497.1      ------------------------------------------------------
------ 0
    JX982495.1      ------------------------------------------------------
------ 0
    JX982496.1      ------------------------------------------------------
------ 0
    JX982500.1      ------------------------------------------------------
------ 0
    EU548051.1      ------------------------------------------------------
------ 0
    EU548044.1      ------------------------------------------------------
------ 0
    EU548042.1      ------------------------------------------------------
------ 0
    EU548043.1      ------------------------------------------------------
------ 0
    EU548047.1      ------------------------------------------------------
------ 0
    EU548050.1      ------------------------------------------------------
------ 0
    EU548048.1      ------------------------------------------------------
------ 0
    EU548049.1      ------------------------------------------------------
------ 0
    AF207722.1      ------------------------------------------------------
------ 0
    EU548037.1      ------------------------------------------------------
------ 0
```

```
    AF207723.1      ------------------------------------------------------
------ 0
    EU548038.1      ------------------------------------------------------
------ 0
    EU548036.1      ------------------------------------------------------
------ 0
    EU548035.1      ------------------------------------------------------
------ 0
    AF207720.1      ------------------------------------------------------
------ 0
    AB601576.1      ------------------------------------------------------
------ 0
    EU548040.1      ------------------------------------------------------
------ 0
    EU548039.1      ------------------------------------------------------
------ 0
    AF207721.1      ------------------------------------------------------
------ 0
    AF207724.1      ------------------------------------------------------
------ 0
    JX982499.1      ------------------------------------------------------
------ 0
    EU548041.1      ------------------------------------------------------
------ 0
    EU548045.1      ------------------------------------------------------
------ 0
    AF207725.1      ------------------------------------------------------
------ 0
    EU548046.1      ------------------------------------------------------
------ 0
    AF207714.1      ------------------------------------------------------
------ 0
    AF207713.1      ------------------------------------------------------
------ 0
    AF207712.1      ------------------------------------------------------
------ 0
    AY750628.1
AAAGCACAAAAAATACAACCCTATTACCCCCATTAATTGTGATATCAACTATACTCCTCC   1012
    EF689084.1      ------------------------------------------------------
------ 0
    EF689085.1      ------------------------------------------------------
------ 0
    AB119070.1      ------------------------------------------------------
------ 956
    EF987742.1      ------------------------------------------------------
------ 0
    AB026105.1      ------------------------------------------------------
------ 0
    MW148603.1
AAAGCACAAAAAATACAACCCTATTACCCCCATTAATTGTAATATCAACTATACTCCTCC   4920
    AB051263.1      ------------------------------------------------------
------ 0
    AF068544.1      ------------------------------------------------------
------ 0


    JX982502.1      ------------------------------------------------------
------ 0
    JX982501.1      ------------------------------------------------------
------ 0
    JX982498.1      ------------------------------------------------------
------ 0
    JX982497.1      ------------------------------------------------------
------ 0
    JX982495.1      ------------------------------------------------------
------ 0
```

```
    JX982496.1        ------------------------------------------------------
------ 0
    JX982500.1        ------------------------------------------------------
------ 0
    EU548051.1        ------------------------------------------------------
------ 0
    EU548044.1        ------------------------------------------------------
------ 0
    EU548042.1        ------------------------------------------------------
------ 0
    EU548043.1        ------------------------------------------------------
------ 0
    EU548047.1        ------------------------------------------------------
------ 0
    EU548050.1        ------------------------------------------------------
------ 0
    EU548048.1        ------------------------------------------------------
------ 0
    EU548049.1        ------------------------------------------------------
------ 0
    AF207722.1        ------------------------------------------------------
------ 0
    EU548037.1        ------------------------------------------------------
------ 0
    AF207723.1        ------------------------------------------------------
------ 0
    EU548038.1        ------------------------------------------------------
------ 0
    EU548036.1        ------------------------------------------------------
------ 0
    EU548035.1        ------------------------------------------------------
------ 0
    AF207720.1        ------------------------------------------------------
------ 0
    AB601576.1        ------------------------------------------------------
------ 0
    EU548040.1        ------------------------------------------------------
------ 0
    EU548039.1        ------------------------------------------------------
------ 0
    AF207721.1        ------------------------------------------------------
------ 0
    AF207724.1        ------------------------------------------------------
------ 0
    JX982499.1        ------------------------------------------------------
------ 0
    EU548041.1        ------------------------------------------------------
------ 0
    EU548045.1        ------------------------------------------------------
------ 0
    AF207725.1        ------------------------------------------------------
------ 0
    EU548046.1        ------------------------------------------------------
------ 0
    AF207714.1        ------------------------------------------------------
------ 0
    AF207713.1        ------------------------------------------------------
------ 0
    AF207712.1        ------------------------------------------------------
------ 0
    AY750628.1        CACTCACCCCAATAATACCAACACTATTCTAG----------------------
------ 1044
    EF689084.1        ------------------------------------------------------
------ 0
    EF689085.1        ------------------------------------------------------
------ 0
```

```
    AB119070.1       ------------------------------------------------------
------ 956
    EF987742.1       ------------------------------------------------------
------ 0
    AB026105.1       ------------------------------------------------------
------ 0
    MW148603.1
CACTCACCCCAATAATACCAACACTATTCTAGGAGTTTAGGTTAAAAAGACCAAGGACCT   4980
    AB051263.1       ------------------------------------------------------
------ 0
    AF068544.1       ------------------------------------------------------
------ 0


    JX982502.1       ------------------------------------------------------
------ 0
    JX982501.1       ------------------------------------------------------
------ 0
    JX982498.1       ------------------------------------------------------
------ 0
    JX982497.1       ------------------------------------------------------
------ 0
    JX982495.1       ------------------------------------------------------
------ 0
    JX982496.1       ------------------------------------------------------
------ 0
    JX982500.1       ------------------------------------------------------
------ 0
    EU548051.1       ------------------------------------------------------
------ 0
    EU548044.1       ------------------------------------------------------
------ 0
    EU548042.1       ------------------------------------------------------
------ 0
    EU548043.1       ------------------------------------------------------
------ 0
    EU548047.1       ------------------------------------------------------
------ 0
    EU548050.1       ------------------------------------------------------
------ 0
    EU548048.1       ------------------------------------------------------
------ 0
    EU548049.1       ------------------------------------------------------
------ 0
    AF207722.1       ------------------------------------------------------
------ 0
    EU548037.1       ------------------------------------------------------
------ 0
    AF207723.1       ------------------------------------------------------
------ 0
    EU548038.1       ------------------------------------------------------
------ 0
    EU548036.1       ------------------------------------------------------
------ 0
    EU548035.1       ------------------------------------------------------
------ 0
    AF207720.1       ------------------------------------------------------
------ 0
    AB601576.1       ------------------------------------------------------
------ 0
    EU548040.1       ------------------------------------------------------
------ 0
    EU548039.1       ------------------------------------------------------
------ 0
    AF207721.1       ------------------------------------------------------
------ 0
```

```
   AF207724.1      ------------------------------------------------------
------ 0
   JX982499.1      ------------------------------------------------------
------ 0
   EU548041.1      ------------------------------------------------------
------ 0
   EU548045.1      ------------------------------------------------------
------ 0
   AF207725.1      ------------------------------------------------------
------ 0
   EU548046.1      ------------------------------------------------------
------ 0
   AF207714.1      ------------------------------------------------------
------ 0
   AF207713.1      ------------------------------------------------------
------ 0
   AF207712.1      ------------------------------------------------------
------ 0
   AY750628.1      ------------------------------------------------------
------ 1044
   EF689084.1      ------------------------------------------------------
------ 0
   EF689085.1      ------------------------------------------------------
------ 0
   AB119070.1      ------------------------------------------------------
------ 956
   EF987742.1      ------------------------------------------------------
------ 0
   AB026105.1      ------------------------------------------------------
------ 0
   MW148603.1
TCAAAGCCCTAAGTAAGTGACACTCACTTAACTCCTGATTCCCATCATAAGGACTGCAAG   5040
   AB051263.1      ------------------------------------------------------
------ 0
   AF068544.1      ------------------------------------------------------
------ 0


   JX982502.1      ------------------------------------------------------
------ 0
   JX982501.1      ------------------------------------------------------
------ 0
   JX982498.1      ------------------------------------------------------
------ 0
   JX982497.1      ------------------------------------------------------
------ 0
   JX982495.1      ------------------------------------------------------
------ 0
   JX982496.1      ------------------------------------------------------
------ 0
   JX982500.1      ------------------------------------------------------
------ 0
   EU548051.1      ------------------------------------------------------
------ 0
   EU548044.1      ------------------------------------------------------
------ 0
   EU548042.1      ------------------------------------------------------
------ 0
   EU548043.1      ------------------------------------------------------
------ 0
   EU548047.1      ------------------------------------------------------
------ 0
   EU548050.1      ------------------------------------------------------
------ 0
   EU548048.1      ------------------------------------------------------
------ 0
```

EU548049.1 --
------ 0
AF207722.1 --
------ 0
EU548037.1 --
------ 0
AF207723.1 --
------ 0
EU548038.1 --
------ 0
EU548036.1 --
------ 0
EU548035.1 --
------ 0
AF207720.1 --
------ 0
AB601576.1 --
------ 0
EU548040.1 --
------ 0
EU548039.1 --
------ 0
AF207721.1 --
------ 0
AF207724.1 --
------ 0
JX982499.1 --
------ 0
EU548041.1 --
------ 0
EU548045.1 --
------ 0
AF207725.1 --
------ 0
EU548046.1 --
------ 0
AF207714.1 --
------ 0
AF207713.1 --
------ 0
AF207712.1 --
------ 0
AY750628.1 --
------ 1044
EF689084.1 --
------ 0
EF689085.1 --
------ 0
AB119070.1 --
------ 956
EF987742.1 --
------ 0
AB026105.1 --
------ 0
MW148603.1
GGTATATCTCACATCTATTGAACGCAAATCAATCACTTTAATTAAGCTAAGCCCTTCCTA 5100
AB051263.1 --
------ 0
AF068544.1 --
------ 0

JX982502.1 --
------ 0
JX982501.1 --
------ 0

```
    JX982498.1      ------------------------------------------------------
------ 0
    JX982497.1      ------------------------------------------------------
------ 0
    JX982495.1      ------------------------------------------------------
------ 0
    JX982496.1      ------------------------------------------------------
------ 0
    JX982500.1      ------------------------------------------------------
------ 0
    EU548051.1      ------------------------------------------------------
------ 0
    EU548044.1      ------------------------------------------------------
------ 0
    EU548042.1      ------------------------------------------------------
------ 0
    EU548043.1      ------------------------------------------------------
------ 0
    EU548047.1      ------------------------------------------------------
------ 0
    EU548050.1      ------------------------------------------------------
------ 0
    EU548048.1      ------------------------------------------------------
------ 0
    EU548049.1      ------------------------------------------------------
------ 0
    AF207722.1      ------------------------------------------------------
------ 0
    EU548037.1      ------------------------------------------------------
------ 0
    AF207723.1      ------------------------------------------------------
------ 0
    EU548038.1      ------------------------------------------------------
------ 0
    EU548036.1      ------------------------------------------------------
------ 0
    EU548035.1      ------------------------------------------------------
------ 0
    AF207720.1      ------------------------------------------------------
------ 0
    AB601576.1      ------------------------------------------------------
------ 0
    EU548040.1      ------------------------------------------------------
------ 0
    EU548039.1      ------------------------------------------------------
------ 0
    AF207721.1      ------------------------------------------------------
------ 0
    AF207724.1      ------------------------------------------------------
------ 0
    JX982499.1      ------------------------------------------------------
------ 0
    EU548041.1      ------------------------------------------------------
------ 0
    EU548045.1      ------------------------------------------------------
------ 0
    AF207725.1      ------------------------------------------------------
------ 0
    EU548046.1      ------------------------------------------------------
------ 0
    AF207714.1      ------------------------------------------------------
------ 0
    AF207713.1      ------------------------------------------------------
------ 0
    AF207712.1      ------------------------------------------------------
------ 0
```

```
    AY750628.1        ------------------------------------------------------
------ 1044
    EF689084.1        ------------------------------------------------------
------ 0
    EF689085.1        ------------------------------------------------------
------ 0
    AB119070.1        ------------------------------------------------------
------ 956
    EF987742.1        ------------------------------------------------------
------ 0
    AB026105.1        ------------------------------------------------------
------ 0
    MW148603.1
GATTGGTGGGCTACCATCCCACGAAACTTTAGTTAACAGCTAAACACCCTAATCAACTGG   5160
    AB051263.1        ------------------------------------------------------
------ 0
    AF068544.1        ------------------------------------------------------
------ 0


    JX982502.1        ------------------------------------------------------
------ 0
    JX982501.1        ------------------------------------------------------
------ 0
    JX982498.1        ------------------------------------------------------
------ 0
    JX982497.1        ------------------------------------------------------
------ 0
    JX982495.1        ------------------------------------------------------
------ 0
    JX982496.1        ------------------------------------------------------
------ 0
    JX982500.1        ------------------------------------------------------
------ 0
    EU548051.1        ------------------------------------------------------
------ 0
    EU548044.1        ------------------------------------------------------
------ 0
    EU548042.1        ------------------------------------------------------
------ 0
    EU548043.1        ------------------------------------------------------
------ 0
    EU548047.1        ------------------------------------------------------
------ 0
    EU548050.1        ------------------------------------------------------
------ 0
    EU548048.1        ------------------------------------------------------
------ 0
    EU548049.1        ------------------------------------------------------
------ 0
    AF207722.1        ------------------------------------------------------
------ 0
    EU548037.1        ------------------------------------------------------
------ 0
    AF207723.1        ------------------------------------------------------
------ 0
    EU548038.1        ------------------------------------------------------
------ 0
    EU548036.1        ------------------------------------------------------
------ 0
    EU548035.1        ------------------------------------------------------
------ 0
    AF207720.1        ------------------------------------------------------
------ 0
    AB601576.1        ------------------------------------------------------
------ 0
```

```
    EU548040.1      ------------------------------------------------------
------ 0
    EU548039.1      ------------------------------------------------------
------ 0
    AF207721.1      ------------------------------------------------------
------ 0
    AF207724.1      ------------------------------------------------------
------ 0
    JX982499.1      ------------------------------------------------------
------ 0
    EU548041.1      ------------------------------------------------------
------ 0
    EU548045.1      ------------------------------------------------------
------ 0
    AF207725.1      ------------------------------------------------------
------ 0
    EU548046.1      ------------------------------------------------------
------ 0
    AF207714.1      ------------------------------------------------------
------ 0
    AF207713.1      ------------------------------------------------------
------ 0
    AF207712.1      ------------------------------------------------------
------ 0
    AY750628.1      ------------------------------------------------------
------ 1044
    EF689084.1      ------------------------------------------------------
------ 0
    EF689085.1      ------------------------------------------------------
------ 0
    AB119070.1      ------------------------------------------------------
------ 956
    EF987742.1      ------------------------------------------------------
------ 0
    AB026105.1      ------------------------------------------------------
------ 0
    MW148603.1
CTTCAATCTACTTCTCCCGCCGCGAAGGAAAAAAGGCGGGAGAAGCCCCGGCAGGGTTGA   5220
    AB051263.1      ------------------------------------------------------
------ 0
    AF068544.1      ------------------------------------------------------
------ 0


    JX982502.1      ------------------------------------------------------
------ 0
    JX982501.1      ------------------------------------------------------
------ 0
    JX982498.1      ------------------------------------------------------
------ 0
    JX982497.1      ------------------------------------------------------
------ 0
    JX982495.1      ------------------------------------------------------
------ 0
    JX982496.1      ------------------------------------------------------
------ 0
    JX982500.1      ------------------------------------------------------
------ 0
    EU548051.1      ------------------------------------------------------
------ 0
    EU548044.1      ------------------------------------------------------
------ 0
    EU548042.1      ------------------------------------------------------
------ 0
    EU548043.1      ------------------------------------------------------
------ 0
```

EU548047.1 --
------ 0
EU548050.1 --
------ 0
EU548048.1 --
------ 0
EU548049.1 --
------ 0
AF207722.1 --
------ 0
EU548037.1 --
------ 0
AF207723.1 --
------ 0
EU548038.1 --
------ 0
EU548036.1 --
------ 0
EU548035.1 --
------ 0
AF207720.1 --
------ 0
AB601576.1 --
------ 0
EU548040.1 --
------ 0
EU548039.1 --
------ 0
AF207721.1 --
------ 0
AF207724.1 --
------ 0
JX982499.1 --
------ 0
EU548041.1 --
------ 0
EU548045.1 --
------ 0
AF207725.1 --
------ 0
EU548046.1 --
------ 0
AF207714.1 --
------ 0
AF207713.1 --
------ 0
AF207712.1 --
------ 0
AY750628.1 --
------ 1044
EF689084.1 --
------ 0
EF689085.1 --
------ 0
AB119070.1 --
------ 956
EF987742.1 --
------ 0
AB026105.1 --
------ 0
MW148603.1
AGCTGCTTCTTTGAATTTGCAATTCAACGTGATATTTCACCACAGAGCTTTGGCAAAAAG 5280
AB051263.1 --
------ 0
AF068544.1 --
------ 0

```
    JX982502.1      -------------------------------------------------------
------ 0
    JX982501.1      -------------------------------------------------------
------ 0
    JX982498.1      -------------------------------------------------------
------ 0
    JX982497.1      -------------------------------------------------------
------ 0
    JX982495.1      -------------------------------------------------------
------ 0
    JX982496.1      -------------------------------------------------------
------ 0
    JX982500.1      -------------------------------------------------------
------ 0
    EU548051.1      -------------------------------------------------------
------ 0
    EU548044.1      -------------------------------------------------------
------ 0
    EU548042.1      -------------------------------------------------------
------ 0
    EU548043.1      -------------------------------------------------------
------ 0
    EU548047.1      -------------------------------------------------------
------ 0
    EU548050.1      -------------------------------------------------------
------ 0
    EU548048.1      -------------------------------------------------------
------ 0
    EU548049.1      -------------------------------------------------------
------ 0
    AF207722.1      -------------------------------------------------------
------ 0
    EU548037.1      -------------------------------------------------------
------ 0
    AF207723.1      -------------------------------------------------------
------ 0
    EU548038.1      -------------------------------------------------------
------ 0
    EU548036.1      -------------------------------------------------------
------ 0
    EU548035.1      -------------------------------------------------------
------ 0
    AF207720.1      -------------------------------------------------------
------ 0
    AB601576.1      -------------------------------------------------------
------ 0
    EU548040.1      -------------------------------------------------------
------ 0
    EU548039.1      -------------------------------------------------------
------ 0
    AF207721.1      -------------------------------------------------------
------ 0
    AF207724.1      -------------------------------------------------------
------ 0
    JX982499.1      -------------------------------------------------------
------ 0
    EU548041.1      -------------------------------------------------------
------ 0
    EU548045.1      -------------------------------------------------------
------ 0
    AF207725.1      -------------------------------------------------------
------ 0
    EU548046.1      -------------------------------------------------------
------ 0
```

```
   AF207714.1      ------------------------------------------------------
------ 0
   AF207713.1      ------------------------------------------------------
------ 0
   AF207712.1      ------------------------------------------------------
------ 0
   AY750628.1      ------------------------------------------------------
------ 1044
   EF689084.1      ------------------------------------------------------
------ 0
   EF689085.1      ------------------------------------------------------
------ 0
   AB119070.1      ------------------------------------------------------
------ 956
   EF987742.1      ------------------------------------------------------
------ 0
   AB026105.1      ------------------------------------------------------
------ 0
   MW148603.1
GGGACTTAAACCCCTATTCTTAGATTTACAGTCTAATGCCTTTATCAGCCATTTTACCTA   5340
   AB051263.1      ------------------------------------------------------
------ 0
   AF068544.1      ------------------------------------------------------
------ 0


   JX982502.1      ------------------------------------------------------
------ 0
   JX982501.1      ------------------------------------------------------
------ 0
   JX982498.1      ------------------------------------------------------
------ 0
   JX982497.1      ------------------------------------------------------
------ 0
   JX982495.1      ------------------------------------------------------
------ 0
   JX982496.1      ------------------------------------------------------
------ 0
   JX982500.1      ------------------------------------------------------
------ 0
   EU548051.1      ------------------------------------------------------
------ 0
   EU548044.1      ------------------------------------------------------
------ 0
   EU548042.1      ------------------------------------------------------
------ 0
   EU548043.1      ------------------------------------------------------
------ 0
   EU548047.1      ------------------------------------------------------
------ 0
   EU548050.1      ------------------------------------------------------
------ 0
   EU548048.1      ------------------------------------------------------
------ 0
   EU548049.1      ------------------------------------------------------
------ 0
   AF207722.1      ------------------------------------------------------
------ 0
   EU548037.1      ------------------------------------------------------
------ 0
   AF207723.1      ------------------------------------------------------
------ 0
   EU548038.1      ------------------------------------------------------
------ 0
   EU548036.1      ------------------------------------------------------
------ 0
```

```
   EU548035.1      ------------------------------------------------------
------ 0
   AF207720.1      ------------------------------------------------------
------ 0
   AB601576.1      ------------------------------------------------------
------ 0
   EU548040.1      ------------------------------------------------------
------ 0
   EU548039.1      ------------------------------------------------------
------ 0
   AF207721.1      ------------------------------------------------------
------ 0
   AF207724.1      ------------------------------------------------------
------ 0
   JX982499.1      ------------------------------------------------------
------ 0
   EU548041.1      ------------------------------------------------------
------ 0
   EU548045.1      ------------------------------------------------------
------ 0
   AF207725.1      ------------------------------------------------------
------ 0
   EU548046.1      ------------------------------------------------------
------ 0
   AF207714.1      ------------------------------------------------------
------ 0
   AF207713.1      ------------------------------------------------------
------ 0
   AF207712.1      ------------------------------------------------------
------ 0
   AY750628.1      ------------------------------------------------------
------ 1044
   EF689084.1      ------------------------------------------------------
------ 0
   EF689085.1      ------------------------------------------------------
------ 0
   AB119070.1      ------------------------------------------------------
------ 956
   EF987742.1      ------------------------------------------------------
------ 0
   AB026105.1      ------------------------------------------------------
------ 0
   MW148603.1
TGTTCATTAATCGATGATTATTCTCCACTAATCACAAAGACATCGGCACCCTCTACTTCT   5400
   AB051263.1      ------------------------------------------------------
------ 0
   AF068544.1      ------------------------------------------------------
------ 0


   JX982502.1      ------------------------------------------------------
------ 0
   JX982501.1      ------------------------------------------------------
------ 0
   JX982498.1      ------------------------------------------------------
------ 0
   JX982497.1      ------------------------------------------------------
------ 0
   JX982495.1      ------------------------------------------------------
------ 0
   JX982496.1      ------------------------------------------------------
------ 0
   JX982500.1      ------------------------------------------------------
------ 0
   EU548051.1      ------------------------------------------------------
------ 0
```

```
    EU548044.1          ------------------------------------------------------
------ 0
    EU548042.1          ------------------------------------------------------
------ 0
    EU548043.1          ------------------------------------------------------
------ 0
    EU548047.1          ------------------------------------------------------
------ 0
    EU548050.1          ------------------------------------------------------
------ 0
    EU548048.1          ------------------------------------------------------
------ 0
    EU548049.1          ------------------------------------------------------
------ 0
    AF207722.1          ------------------------------------------------------
------ 0
    EU548037.1          ------------------------------------------------------
------ 0
    AF207723.1          ------------------------------------------------------
------ 0
    EU548038.1          ------------------------------------------------------
------ 0
    EU548036.1          ------------------------------------------------------
------ 0
    EU548035.1          ------------------------------------------------------
------ 0
    AF207720.1          ------------------------------------------------------
------ 0
    AB601576.1          ------------------------------------------------------
------ 0
    EU548040.1          ------------------------------------------------------
------ 0
    EU548039.1          ------------------------------------------------------
------ 0
    AF207721.1          ------------------------------------------------------
------ 0
    AF207724.1          ------------------------------------------------------
------ 0
    JX982499.1          ------------------------------------------------------
------ 0
    EU548041.1          ------------------------------------------------------
------ 0
    EU548045.1          ------------------------------------------------------
------ 0
    AF207725.1          ------------------------------------------------------
------ 0
    EU548046.1          ------------------------------------------------------
------ 0
    AF207714.1          ------------------------------------------------------
------ 0
    AF207713.1          ------------------------------------------------------
------ 0
    AF207712.1          ------------------------------------------------------
------ 0
    AY750628.1          ------------------------------------------------------
------ 1044
    EF689084.1          ------------------------------------------------------
------ 0
    EF689085.1          ------------------------------------------------------
------ 0
    AB119070.1          ------------------------------------------------------
------ 956
    EF987742.1          ------------------------------------------------------
------ 0
    AB026105.1          ------------------------------------------------------
------ 0
```

MW148603.1
TATTTGGTGCATGAGCCGGAATGGTAGGGACCGCTCTCAGTCTACTGATCCGTGCTGAAC 5460
AB051263.1 --
------ 0
AF068544.1 --
------ 0

JX982502.1 --
------ 0
JX982501.1 --
------ 0
JX982498.1 --
------ 0
JX982497.1 --
------ 0
JX982495.1 --
------ 0
JX982496.1 --
------ 0
JX982500.1 --
------ 0
EU548051.1 --
------ 0
EU548044.1 --
------ 0
EU548042.1 --
------ 0
EU548043.1 --
------ 0
EU548047.1 --
------ 0
EU548050.1 --
------ 0
EU548048.1 --
------ 0
EU548049.1 --
------ 0
AF207722.1 --
------ 0
EU548037.1 --
------ 0
AF207723.1 --
------ 0
EU548038.1 --
------ 0
EU548036.1 --
------ 0
EU548035.1 --
------ 0
AF207720.1 --
------ 0
AB601576.1 --
------ 0
EU548040.1 --
------ 0
EU548039.1 --
------ 0
AF207721.1 --
------ 0
AF207724.1 --
------ 0
JX982499.1 --
------ 0
EU548041.1 --
------ 0

```
    EU548045.1      ------------------------------------------------------
------ 0
    AF207725.1      ------------------------------------------------------
------ 0
    EU548046.1      ------------------------------------------------------
------ 0
    AF207714.1      ------------------------------------------------------
------ 0
    AF207713.1      ------------------------------------------------------
------ 0
    AF207712.1      ------------------------------------------------------
------ 0
    AY750628.1      ------------------------------------------------------
------ 1044
    EF689084.1      ------------------------------------------------------
------ 0
    EF689085.1      ------------------------------------------------------
------ 0
    AB119070.1      ------------------------------------------------------
------ 956
    EF987742.1      ------------------------------------------------------
------ 0
    AB026105.1      ------------------------------------------------------
------ 0
    MW148603.1
TAAGTCAACCTGGCGCTCTGCTAGGAGACGACCAGATTTATAATGTAATCGTAACTGCTC    5520
    AB051263.1      ------------------------------------------------------
------ 0
    AF068544.1      ------------------------------------------------------
------ 0


    JX982502.1      ------------------------------------------------------
------ 0
    JX982501.1      ------------------------------------------------------
------ 0
    JX982498.1      ------------------------------------------------------
------ 0
    JX982497.1      ------------------------------------------------------
------ 0
    JX982495.1      ------------------------------------------------------
------ 0
    JX982496.1      ------------------------------------------------------
------ 0
    JX982500.1      ------------------------------------------------------
------ 0
    EU548051.1      ------------------------------------------------------
------ 0
    EU548044.1      ------------------------------------------------------
------ 0
    EU548042.1      ------------------------------------------------------
------ 0
    EU548043.1      ------------------------------------------------------
------ 0
    EU548047.1      ------------------------------------------------------
------ 0
    EU548050.1      ------------------------------------------------------
------ 0
    EU548048.1      ------------------------------------------------------
------ 0
    EU548049.1      ------------------------------------------------------
------ 0
    AF207722.1      ------------------------------------------------------
------ 0
    EU548037.1      ------------------------------------------------------
------ 0
```

```
    AF207723.1      ------------------------------------------------------
------ 0
    EU548038.1      ------------------------------------------------------
------ 0
    EU548036.1      ------------------------------------------------------
------ 0
    EU548035.1      ------------------------------------------------------
------ 0
    AF207720.1      ------------------------------------------------------
------ 0
    AB601576.1      ------------------------------------------------------
------ 0
    EU548040.1      ------------------------------------------------------
------ 0
    EU548039.1      ------------------------------------------------------
------ 0
    AF207721.1      ------------------------------------------------------
------ 0
    AF207724.1      ------------------------------------------------------
------ 0
    JX982499.1      ------------------------------------------------------
------ 0
    EU548041.1      ------------------------------------------------------
------ 0
    EU548045.1      ------------------------------------------------------
------ 0
    AF207725.1      ------------------------------------------------------
------ 0
    EU548046.1      ------------------------------------------------------
------ 0
    AF207714.1      ------------------------------------------------------
------ 0
    AF207713.1      ------------------------------------------------------
------ 0
    AF207712.1      ------------------------------------------------------
------ 0
    AY750628.1      ------------------------------------------------------
------ 1044
    EF689084.1      ------------------------------------------------------
------ 0
    EF689085.1      ------------------------------------------------------
------ 0
    AB119070.1      ------------------------------------------------------
------ 956
    EF987742.1      ------------------------------------------------------
------ 0
    AB026105.1      ------------------------------------------------------
------ 0
    MW148603.1
ACGCATTTGTAATAATTTTCTTCATAGTAATACCCATCATGCTTGGGGGCTTTGGGAACT   5580
    AB051263.1      ------------------------------------------------------
------ 0
    AF068544.1      ------------------------------------------------------
------ 0


    JX982502.1      ------------------------------------------------------
------ 0
    JX982501.1      ------------------------------------------------------
------ 0
    JX982498.1      ------------------------------------------------------
------ 0
    JX982497.1      ------------------------------------------------------
------ 0
    JX982495.1      ------------------------------------------------------
------ 0
```

```
    JX982496.1         ------------------------------------------------------
------ 0
    JX982500.1         ------------------------------------------------------
------ 0
    EU548051.1         ------------------------------------------------------
------ 0
    EU548044.1         ------------------------------------------------------
------ 0
    EU548042.1         ------------------------------------------------------
------ 0
    EU548043.1         ------------------------------------------------------
------ 0
    EU548047.1         ------------------------------------------------------
------ 0
    EU548050.1         ------------------------------------------------------
------ 0
    EU548048.1         ------------------------------------------------------
------ 0
    EU548049.1         ------------------------------------------------------
------ 0
    AF207722.1         ------------------------------------------------------
------ 0
    EU548037.1         ------------------------------------------------------
------ 0
    AF207723.1         ------------------------------------------------------
------ 0
    EU548038.1         ------------------------------------------------------
------ 0
    EU548036.1         ------------------------------------------------------
------ 0
    EU548035.1         ------------------------------------------------------
------ 0
    AF207720.1         ------------------------------------------------------
------ 0
    AB601576.1         ------------------------------------------------------
------ 0
    EU548040.1         ------------------------------------------------------
------ 0
    EU548039.1         ------------------------------------------------------
------ 0
    AF207721.1         ------------------------------------------------------
------ 0
    AF207724.1         ------------------------------------------------------
------ 0
    JX982499.1         ------------------------------------------------------
------ 0
    EU548041.1         ------------------------------------------------------
------ 0
    EU548045.1         ------------------------------------------------------
------ 0
    AF207725.1         ------------------------------------------------------
------ 0
    EU548046.1         ------------------------------------------------------
------ 0
    AF207714.1         ------------------------------------------------------
------ 0
    AF207713.1         ------------------------------------------------------
------ 0
    AF207712.1         ------------------------------------------------------
------ 0
    AY750628.1         ------------------------------------------------------
------ 1044
    EF689084.1         ------------------------------------------------------
------ 0
    EF689085.1         ------------------------------------------------------
------ 0
```

AB119070.1 --
------ 956
EF987742.1 --
------ 0
AB026105.1 --
------ 0
MW148603.1
GACTTATTCCTCTAATAATCGGCGCACCTGACATAGCATTCCCACGGATAAACAACATAA 5640
AB051263.1 --
------ 0
AF068544.1 --
------ 0

JX982502.1 --
------ 0
JX982501.1 --
------ 0
JX982498.1 --
------ 0
JX982497.1 --
------ 0
JX982495.1 --
------ 0
JX982496.1 --
------ 0
JX982500.1 --
------ 0
EU548051.1 --
------ 0
EU548044.1 --
------ 0
EU548042.1 --
------ 0
EU548043.1 --
------ 0
EU548047.1 --
------ 0
EU548050.1 --
------ 0
EU548048.1 --
------ 0
EU548049.1 --
------ 0
AF207722.1 --
------ 0
EU548037.1 --
------ 0
AF207723.1 --
------ 0
EU548038.1 --
------ 0
EU548036.1 --
------ 0
EU548035.1 --
------ 0
AF207720.1 --
------ 0
AB601576.1 --
------ 0
EU548040.1 --
------ 0
EU548039.1 --
------ 0
AF207721.1 --
------ 0

```
    AF207724.1      ------------------------------------------------------
------ 0
    JX982499.1      ------------------------------------------------------
------ 0
    EU548041.1      ------------------------------------------------------
------ 0
    EU548045.1      ------------------------------------------------------
------ 0
    AF207725.1      ------------------------------------------------------
------ 0
    EU548046.1      ------------------------------------------------------
------ 0
    AF207714.1      ------------------------------------------------------
------ 0
    AF207713.1      ------------------------------------------------------
------ 0
    AF207712.1      ------------------------------------------------------
------ 0
    AY750628.1      ------------------------------------------------------
------ 1044
    EF689084.1      ------------------------------------------------------
------ 0
    EF689085.1      ------------------------------------------------------
------ 0
    AB119070.1      ------------------------------------------------------
------ 956
    EF987742.1      ------------------------------------------------------
------ 0
    AB026105.1      ------------------------------------------------------
------ 0
    MW148603.1
GCTTCTGGCTTCTTCCTCCCTCTTTTCTTCTCCTACTAGCTTCCTCTATGGTAGAAGCAG   5700
    AB051263.1      ------------------------------------------------------
------ 0
    AF068544.1      ------------------------------------------------------
------ 0


    JX982502.1      ------------------------------------------------------
------ 0
    JX982501.1      ------------------------------------------------------
------ 0
    JX982498.1      ------------------------------------------------------
------ 0
    JX982497.1      ------------------------------------------------------
------ 0
    JX982495.1      ------------------------------------------------------
------ 0
    JX982496.1      ------------------------------------------------------
------ 0
    JX982500.1      ------------------------------------------------------
------ 0
    EU548051.1      ------------------------------------------------------
------ 0
    EU548044.1      ------------------------------------------------------
------ 0
    EU548042.1      ------------------------------------------------------
------ 0
    EU548043.1      ------------------------------------------------------
------ 0
    EU548047.1      ------------------------------------------------------
------ 0
    EU548050.1      ------------------------------------------------------
------ 0
    EU548048.1      ------------------------------------------------------
------ 0
```

```
    EU548049.1        ------------------------------------------------------
------ 0
    AF207722.1        ------------------------------------------------------
------ 0
    EU548037.1        ------------------------------------------------------
------ 0
    AF207723.1        ------------------------------------------------------
------ 0
    EU548038.1        ------------------------------------------------------
------ 0
    EU548036.1        ------------------------------------------------------
------ 0
    EU548035.1        ------------------------------------------------------
------ 0
    AF207720.1        ------------------------------------------------------
------ 0
    AB601576.1        ------------------------------------------------------
------ 0
    EU548040.1        ------------------------------------------------------
------ 0
    EU548039.1        ------------------------------------------------------
------ 0
    AF207721.1        ------------------------------------------------------
------ 0
    AF207724.1        ------------------------------------------------------
------ 0
    JX982499.1        ------------------------------------------------------
------ 0
    EU548041.1        ------------------------------------------------------
------ 0
    EU548045.1        ------------------------------------------------------
------ 0
    AF207725.1        ------------------------------------------------------
------ 0
    EU548046.1        ------------------------------------------------------
------ 0
    AF207714.1        ------------------------------------------------------
------ 0
    AF207713.1        ------------------------------------------------------
------ 0
    AF207712.1        ------------------------------------------------------
------ 0
    AY750628.1        ------------------------------------------------------
------ 1044
    EF689084.1        ------------------------------------------------------
------ 0
    EF689085.1        ------------------------------------------------------
------ 0
    AB119070.1        ------------------------------------------------------
------ 956
    EF987742.1        ------------------------------------------------------
------ 0
    AB026105.1        ------------------------------------------------------
------ 0
    MW148603.1
GTGCAGGGACTGGATGAACTGTATACCCCCCTTTAGCAGGAAATCTAGCACATGCTGGAG   5760
    AB051263.1        ------------------------------------------------------
------ 0
    AF068544.1        ------------------------------------------------------
------ 0


    JX982502.1        ------------------------------------------------------
------ 0
    JX982501.1        ------------------------------------------------------
------ 0
```

```
   JX982498.1      ------------------------------------------------------
------ 0
   JX982497.1      ------------------------------------------------------
------ 0
   JX982495.1      ------------------------------------------------------
------ 0
   JX982496.1      ------------------------------------------------------
------ 0
   JX982500.1      ------------------------------------------------------
------ 0
   EU548051.1      ------------------------------------------------------
------ 0
   EU548044.1      ------------------------------------------------------
------ 0
   EU548042.1      ------------------------------------------------------
------ 0
   EU548043.1      ------------------------------------------------------
------ 0
   EU548047.1      ------------------------------------------------------
------ 0
   EU548050.1      ------------------------------------------------------
------ 0
   EU548048.1      ------------------------------------------------------
------ 0
   EU548049.1      ------------------------------------------------------
------ 0
   AF207722.1      ------------------------------------------------------
------ 0
   EU548037.1      ------------------------------------------------------
------ 0
   AF207723.1      ------------------------------------------------------
------ 0
   EU548038.1      ------------------------------------------------------
------ 0
   EU548036.1      ------------------------------------------------------
------ 0
   EU548035.1      ------------------------------------------------------
------ 0
   AF207720.1      ------------------------------------------------------
------ 0
   AB601576.1      ------------------------------------------------------
------ 0
   EU548040.1      ------------------------------------------------------
------ 0
   EU548039.1      ------------------------------------------------------
------ 0
   AF207721.1      ------------------------------------------------------
------ 0
   AF207724.1      ------------------------------------------------------
------ 0
   JX982499.1      ------------------------------------------------------
------ 0
   EU548041.1      ------------------------------------------------------
------ 0
   EU548045.1      ------------------------------------------------------
------ 0
   AF207725.1      ------------------------------------------------------
------ 0
   EU548046.1      ------------------------------------------------------
------ 0
   AF207714.1      ------------------------------------------------------
------ 0
   AF207713.1      ------------------------------------------------------
------ 0
   AF207712.1      ------------------------------------------------------
------ 0
```

```
    AY750628.1       ------------------------------------------------------
------ 1044
    EF689084.1       ------------------------------------------------------
------ 0
    EF689085.1       ------------------------------------------------------
------ 0
    AB119070.1       ------------------------------------------------------
------ 956
    EF987742.1       ------------------------------------------------------
------ 0
    AB026105.1       ------------------------------------------------------
------ 0
    MW148603.1
CATCCGTGGACCTGGCAATCTTTTCTCTACACTTAGCTGGTGTTTCATCTATCTTAGGGT   5820
    AB051263.1       ------------------------------------------------------
------ 0
    AF068544.1       ------------------------------------------------------
------ 0


    JX982502.1       ------------------------------------------------------
------ 0
    JX982501.1       ------------------------------------------------------
------ 0
    JX982498.1       ------------------------------------------------------
------ 0
    JX982497.1       ------------------------------------------------------
------ 0
    JX982495.1       ------------------------------------------------------
------ 0
    JX982496.1       ------------------------------------------------------
------ 0
    JX982500.1       ------------------------------------------------------
------ 0
    EU548051.1       ------------------------------------------------------
------ 0
    EU548044.1       ------------------------------------------------------
------ 0
    EU548042.1       ------------------------------------------------------
------ 0
    EU548043.1       ------------------------------------------------------
------ 0
    EU548047.1       ------------------------------------------------------
------ 0
    EU548050.1       ------------------------------------------------------
------ 0
    EU548048.1       ------------------------------------------------------
------ 0
    EU548049.1       ------------------------------------------------------
------ 0
    AF207722.1       ------------------------------------------------------
------ 0
    EU548037.1       ------------------------------------------------------
------ 0
    AF207723.1       ------------------------------------------------------
------ 0
    EU548038.1       ------------------------------------------------------
------ 0
    EU548036.1       ------------------------------------------------------
------ 0
    EU548035.1       ------------------------------------------------------
------ 0
    AF207720.1       ------------------------------------------------------
------ 0
    AB601576.1       ------------------------------------------------------
------ 0
```

EU548040.1 --
------ 0
EU548039.1 --
------ 0
AF207721.1 --
------ 0
AF207724.1 --
------ 0
JX982499.1 --
------ 0
EU548041.1 --
------ 0
EU548045.1 --
------ 0
AF207725.1 --
------ 0
EU548046.1 --
------ 0
AF207714.1 --
------ 0
AF207713.1 --
------ 0
AF207712.1 --
------ 0
AY750628.1 --
------ 1044
EF689084.1 --
------ 0
EF689085.1 --
------ 0
AB119070.1 --
------ 956
EF987742.1 --
------ 0
AB026105.1 --
------ 0
MW148603.1
CAATCAACTTTATTACTACTATTATCAACATAAAACCGCCTGCCATGTCACAATACCAAA 5880
AB051263.1 --
------ 0
AF068544.1 --
------ 0

JX982502.1 --
------ 0
JX982501.1 --
------ 0
JX982498.1 --
------ 0
JX982497.1 --
------ 0
JX982495.1 --
------ 0
JX982496.1 --
------ 0
JX982500.1 --
------ 0
EU548051.1 --
------ 0
EU548044.1 --
------ 0
EU548042.1 --
------ 0
EU548043.1 --
------ 0

```
    EU548047.1      ------------------------------------------------------
------ 0
    EU548050.1      ------------------------------------------------------
------ 0
    EU548048.1      ------------------------------------------------------
------ 0
    EU548049.1      ------------------------------------------------------
------ 0
    AF207722.1      ------------------------------------------------------
------ 0
    EU548037.1      ------------------------------------------------------
------ 0
    AF207723.1      ------------------------------------------------------
------ 0
    EU548038.1      ------------------------------------------------------
------ 0
    EU548036.1      ------------------------------------------------------
------ 0
    EU548035.1      ------------------------------------------------------
------ 0
    AF207720.1      ------------------------------------------------------
------ 0
    AB601576.1      ------------------------------------------------------
------ 0
    EU548040.1      ------------------------------------------------------
------ 0
    EU548039.1      ------------------------------------------------------
------ 0
    AF207721.1      ------------------------------------------------------
------ 0
    AF207724.1      ------------------------------------------------------
------ 0
    JX982499.1      ------------------------------------------------------
------ 0
    EU548041.1      ------------------------------------------------------
------ 0
    EU548045.1      ------------------------------------------------------
------ 0
    AF207725.1      ------------------------------------------------------
------ 0
    EU548046.1      ------------------------------------------------------
------ 0
    AF207714.1      ------------------------------------------------------
------ 0
    AF207713.1      ------------------------------------------------------
------ 0
    AF207712.1      ------------------------------------------------------
------ 0
    AY750628.1      ------------------------------------------------------
------ 1044
    EF689084.1      ------------------------------------------------------
------ 0
    EF689085.1      ------------------------------------------------------
------ 0
    AB119070.1      ------------------------------------------------------
------ 956
    EF987742.1      ------------------------------------------------------
------ 0
    AB026105.1      ------------------------------------------------------
------ 0
    MW148603.1
CTCCACTATTTGTTTGATCAGTTTTAATTACAGCCGTACTTCTTCTTCTGTCCCTGCCAG   5940
    AB051263.1      ------------------------------------------------------
------ 0
    AF068544.1      ------------------------------------------------------
------ 0
```

```
    JX982502.1       ------------------------------------------------------
------ 0
    JX982501.1       ------------------------------------------------------
------ 0
    JX982498.1       ------------------------------------------------------
------ 0
    JX982497.1       ------------------------------------------------------
------ 0
    JX982495.1       ------------------------------------------------------
------ 0
    JX982496.1       ------------------------------------------------------
------ 0
    JX982500.1       ------------------------------------------------------
------ 0
    EU548051.1       ------------------------------------------------------
------ 0
    EU548044.1       ------------------------------------------------------
------ 0
    EU548042.1       ------------------------------------------------------
------ 0
    EU548043.1       ------------------------------------------------------
------ 0
    EU548047.1       ------------------------------------------------------
------ 0
    EU548050.1       ------------------------------------------------------
------ 0
    EU548048.1       ------------------------------------------------------
------ 0
    EU548049.1       ------------------------------------------------------
------ 0
    AF207722.1       ------------------------------------------------------
------ 0
    EU548037.1       ------------------------------------------------------
------ 0
    AF207723.1       ------------------------------------------------------
------ 0
    EU548038.1       ------------------------------------------------------
------ 0
    EU548036.1       ------------------------------------------------------
------ 0
    EU548035.1       ------------------------------------------------------
------ 0
    AF207720.1       ------------------------------------------------------
------ 0
    AB601576.1       ------------------------------------------------------
------ 0
    EU548040.1       ------------------------------------------------------
------ 0
    EU548039.1       ------------------------------------------------------
------ 0
    AF207721.1       ------------------------------------------------------
------ 0
    AF207724.1       ------------------------------------------------------
------ 0
    JX982499.1       ------------------------------------------------------
------ 0
    EU548041.1       ------------------------------------------------------
------ 0
    EU548045.1       ------------------------------------------------------
------ 0
    AF207725.1       ------------------------------------------------------
------ 0
    EU548046.1       ------------------------------------------------------
------ 0
```

AF207714.1 --
------ 0
AF207713.1 --
------ 0
AF207712.1 --
------ 0
AY750628.1 --
------ 1044
EF689084.1 --
------ 0
EF689085.1 --
------ 0
AB119070.1 --
------ 956
EF987742.1 --
------ 0
AB026105.1 --
------ 0
MW148603.1
TTTTAGCAGCCGGCATTACTATATTACTTACAGACCGTAATCTAAATACTACTTTCTTTG 6000
AB051263.1 --
------ 0
AF068544.1 --
------ 0

JX982502.1 --
------ 0
JX982501.1 --
------ 0
JX982498.1 --
------ 0
JX982497.1 --
------ 0
JX982495.1 --
------ 0
JX982496.1 --
------ 0
JX982500.1 --
------ 0
EU548051.1 --
------ 0
EU548044.1 --
------ 0
EU548042.1 --
------ 0
EU548043.1 --
------ 0
EU548047.1 --
------ 0
EU548050.1 --
------ 0
EU548048.1 --
------ 0
EU548049.1 --
------ 0
AF207722.1 --
------ 0
EU548037.1 --
------ 0
AF207723.1 --
------ 0
EU548038.1 --
------ 0
EU548036.1 --
------ 0

```
   EU548035.1      ------------------------------------------------------
------ 0
   AF207720.1      ------------------------------------------------------
------ 0
   AB601576.1      ------------------------------------------------------
------ 0
   EU548040.1      ------------------------------------------------------
------ 0
   EU548039.1      ------------------------------------------------------
------ 0
   AF207721.1      ------------------------------------------------------
------ 0
   AF207724.1      ------------------------------------------------------
------ 0
   JX982499.1      ------------------------------------------------------
------ 0
   EU548041.1      ------------------------------------------------------
------ 0
   EU548045.1      ------------------------------------------------------
------ 0
   AF207725.1      ------------------------------------------------------
------ 0
   EU548046.1      ------------------------------------------------------
------ 0
   AF207714.1      ------------------------------------------------------
------ 0
   AF207713.1      ------------------------------------------------------
------ 0
   AF207712.1      ------------------------------------------------------
------ 0
   AY750628.1      ------------------------------------------------------
------ 1044
   EF689084.1      ------------------------------------------------------
------ 0
   EF689085.1      ------------------------------------------------------
------ 0
   AB119070.1      ------------------------------------------------------
------ 956
   EF987742.1      ------------------------------------------------------
------ 0
   AB026105.1      ------------------------------------------------------
------ 0
   MW148603.1
ACCCAGCCGGAGGGGGGGACCCTATCCTGTACCAACACTTATTTTGATTCTTTGGGCACC   6060
   AB051263.1      ------------------------------------------------------
------ 0
   AF068544.1      ------------------------------------------------------
------ 0


   JX982502.1      ------------------------------------------------------
------ 0
   JX982501.1      ------------------------------------------------------
------ 0
   JX982498.1      ------------------------------------------------------
------ 0
   JX982497.1      ------------------------------------------------------
------ 0
   JX982495.1      ------------------------------------------------------
------ 0
   JX982496.1      ------------------------------------------------------
------ 0
   JX982500.1      ------------------------------------------------------
------ 0
   EU548051.1      ------------------------------------------------------
------ 0
```

```
    EU548044.1          ------------------------------------------------------
------ 0
    EU548042.1          ------------------------------------------------------
------ 0
    EU548043.1          ------------------------------------------------------
------ 0
    EU548047.1          ------------------------------------------------------
------ 0
    EU548050.1          ------------------------------------------------------
------ 0
    EU548048.1          ------------------------------------------------------
------ 0
    EU548049.1          ------------------------------------------------------
------ 0
    AF207722.1          ------------------------------------------------------
------ 0
    EU548037.1          ------------------------------------------------------
------ 0
    AF207723.1          ------------------------------------------------------
------ 0
    EU548038.1          ------------------------------------------------------
------ 0
    EU548036.1          ------------------------------------------------------
------ 0
    EU548035.1          ------------------------------------------------------
------ 0
    AF207720.1          ------------------------------------------------------
------ 0
    AB601576.1          ------------------------------------------------------
------ 0
    EU548040.1          ------------------------------------------------------
------ 0
    EU548039.1          ------------------------------------------------------
------ 0
    AF207721.1          ------------------------------------------------------
------ 0
    AF207724.1          ------------------------------------------------------
------ 0
    JX982499.1          ------------------------------------------------------
------ 0
    EU548041.1          ------------------------------------------------------
------ 0
    EU548045.1          ------------------------------------------------------
------ 0
    AF207725.1          ------------------------------------------------------
------ 0
    EU548046.1          ------------------------------------------------------
------ 0
    AF207714.1          ------------------------------------------------------
------ 0
    AF207713.1          ------------------------------------------------------
------ 0
    AF207712.1          ------------------------------------------------------
------ 0
    AY750628.1          ------------------------------------------------------
------ 1044
    EF689084.1          ------------------------------------------------------
------ 0
    EF689085.1          ------------------------------------------------------
------ 0
    AB119070.1          ------------------------------------------------------
------ 956
    EF987742.1          ------------------------------------------------------
------ 0
    AB026105.1          ------------------------------------------------------
------ 0
```

```
    MW148603.1
CAGAAGTTTATATCCTGATTCTCCCAGGATTTGGTATCATTTCACACGTTGTAACTTACT   6120
    AB051263.1      ------------------------------------------------------
------ 0
    AF068544.1      ------------------------------------------------------
------ 0


    JX982502.1      ------------------------------------------------------
------ 0
    JX982501.1      ------------------------------------------------------
------ 0
    JX982498.1      ------------------------------------------------------
------ 0
    JX982497.1      ------------------------------------------------------
------ 0
    JX982495.1      ------------------------------------------------------
------ 0
    JX982496.1      ------------------------------------------------------
------ 0
    JX982500.1      ------------------------------------------------------
------ 0
    EU548051.1      ------------------------------------------------------
------ 0
    EU548044.1      ------------------------------------------------------
------ 0
    EU548042.1      ------------------------------------------------------
------ 0
    EU548043.1      ------------------------------------------------------
------ 0
    EU548047.1      ------------------------------------------------------
------ 0
    EU548050.1      ------------------------------------------------------
------ 0
    EU548048.1      ------------------------------------------------------
------ 0
    EU548049.1      ------------------------------------------------------
------ 0
    AF207722.1      ------------------------------------------------------
------ 0
    EU548037.1      ------------------------------------------------------
------ 0
    AF207723.1      ------------------------------------------------------
------ 0
    EU548038.1      ------------------------------------------------------
------ 0
    EU548036.1      ------------------------------------------------------
------ 0
    EU548035.1      ------------------------------------------------------
------ 0
    AF207720.1      ------------------------------------------------------
------ 0
    AB601576.1      ------------------------------------------------------
------ 0
    EU548040.1      ------------------------------------------------------
------ 0
    EU548039.1      ------------------------------------------------------
------ 0
    AF207721.1      ------------------------------------------------------
------ 0
    AF207724.1      ------------------------------------------------------
------ 0
    JX982499.1      ------------------------------------------------------
------ 0
    EU548041.1      ------------------------------------------------------
------ 0
```

```
    EU548045.1       ------------------------------------------------------
------ 0
    AF207725.1       ------------------------------------------------------
------ 0
    EU548046.1       ------------------------------------------------------
------ 0
    AF207714.1       ------------------------------------------------------
------ 0
    AF207713.1       ------------------------------------------------------
------ 0
    AF207712.1       ------------------------------------------------------
------ 0
    AY750628.1       ------------------------------------------------------
------ 1044
    EF689084.1       ------------------------------------------------------
------ 0
    EF689085.1       ------------------------------------------------------
------ 0
    AB119070.1       ------------------------------------------------------
------ 956
    EF987742.1       ------------------------------------------------------
------ 0
    AB026105.1       ------------------------------------------------------
------ 0
    MW148603.1
ATTCAGGAAAAAAAGAACCATTTGGTTATATGGGAATAGTATGGGCAATAATATCAATTG   6180
    AB051263.1       ------------------------------------------------------
------ 0
    AF068544.1       ------------------------------------------------------
------ 0


    JX982502.1       ------------------------------------------------------
------ 0
    JX982501.1       ------------------------------------------------------
------ 0
    JX982498.1       ------------------------------------------------------
------ 0
    JX982497.1       ------------------------------------------------------
------ 0
    JX982495.1       ------------------------------------------------------
------ 0
    JX982496.1       ------------------------------------------------------
------ 0
    JX982500.1       ------------------------------------------------------
------ 0
    EU548051.1       ------------------------------------------------------
------ 0
    EU548044.1       ------------------------------------------------------
------ 0
    EU548042.1       ------------------------------------------------------
------ 0
    EU548043.1       ------------------------------------------------------
------ 0
    EU548047.1       ------------------------------------------------------
------ 0
    EU548050.1       ------------------------------------------------------
------ 0
    EU548048.1       ------------------------------------------------------
------ 0
    EU548049.1       ------------------------------------------------------
------ 0
    AF207722.1       ------------------------------------------------------
------ 0
    EU548037.1       ------------------------------------------------------
------ 0
```

```
   AF207723.1      ------------------------------------------------------
------ 0
   EU548038.1      ------------------------------------------------------
------ 0
   EU548036.1      ------------------------------------------------------
------ 0
   EU548035.1      ------------------------------------------------------
------ 0
   AF207720.1      ------------------------------------------------------
------ 0
   AB601576.1      ------------------------------------------------------
------ 0
   EU548040.1      ------------------------------------------------------
------ 0
   EU548039.1      ------------------------------------------------------
------ 0
   AF207721.1      ------------------------------------------------------
------ 0
   AF207724.1      ------------------------------------------------------
------ 0
   JX982499.1      ------------------------------------------------------
------ 0
   EU548041.1      ------------------------------------------------------
------ 0
   EU548045.1      ------------------------------------------------------
------ 0
   AF207725.1      ------------------------------------------------------
------ 0
   EU548046.1      ------------------------------------------------------
------ 0
   AF207714.1      ------------------------------------------------------
------ 0
   AF207713.1      ------------------------------------------------------
------ 0
   AF207712.1      ------------------------------------------------------
------ 0
   AY750628.1      ------------------------------------------------------
------ 1044
   EF689084.1      ------------------------------------------------------
------ 0
   EF689085.1      ------------------------------------------------------
------ 0
   AB119070.1      ------------------------------------------------------
------ 956
   EF987742.1      ------------------------------------------------------
------ 0
   AB026105.1      ------------------------------------------------------
------ 0
   MW148603.1
GTTTCCTAGGATTTATCGTATGAGCCCACCATATATTTACTGTGGGCCTGGACGTCGACA   6240
   AB051263.1      ------------------------------------------------------
------ 0
   AF068544.1      ------------------------------------------------------
------ 0


   JX982502.1      ------------------------------------------------------
------ 0
   JX982501.1      ------------------------------------------------------
------ 0
   JX982498.1      ------------------------------------------------------
------ 0
   JX982497.1      ------------------------------------------------------
------ 0
   JX982495.1      ------------------------------------------------------
------ 0
```

JX982496.1 --
------ 0
JX982500.1 --
------ 0
EU548051.1 --
------ 0
EU548044.1 --
------ 0
EU548042.1 --
------ 0
EU548043.1 --
------ 0
EU548047.1 --
------ 0
EU548050.1 --
------ 0
EU548048.1 --
------ 0
EU548049.1 --
------ 0
AF207722.1 --
------ 0
EU548037.1 --
------ 0
AF207723.1 --
------ 0
EU548038.1 --
------ 0
EU548036.1 --
------ 0
EU548035.1 --
------ 0
AF207720.1 --
------ 0
AB601576.1 --
------ 0
EU548040.1 --
------ 0
EU548039.1 --
------ 0
AF207721.1 --
------ 0
AF207724.1 --
------ 0
JX982499.1 --
------ 0
EU548041.1 --
------ 0
EU548045.1 --
------ 0
AF207725.1 --
------ 0
EU548046.1 --
------ 0
AF207714.1 --
------ 0
AF207713.1 --
------ 0
AF207712.1 --
------ 0
AY750628.1 --
------ 1044
EF689084.1 --
------ 0
EF689085.1 --
------ 0

```
   AB119070.1      ------------------------------------------------------
------ 956
   EF987742.1      ------------------------------------------------------
------ 0
   AB026105.1      ------------------------------------------------------
------ 0
   MW148603.1
CACGAGCATATTTCACTTCAGCTACTATAATCATCGCTATCCCCACAGGAGTAAAAGTAT   6300
   AB051263.1      ------------------------------------------------------
------ 0
   AF068544.1      ------------------------------------------------------
------ 0


   JX982502.1      ------------------------------------------------------
------ 0
   JX982501.1      ------------------------------------------------------
------ 0
   JX982498.1      ------------------------------------------------------
------ 0
   JX982497.1      ------------------------------------------------------
------ 0
   JX982495.1      ------------------------------------------------------
------ 0
   JX982496.1      ------------------------------------------------------
------ 0
   JX982500.1      ------------------------------------------------------
------ 0
   EU548051.1      ------------------------------------------------------
------ 0
   EU548044.1      ------------------------------------------------------
------ 0
   EU548042.1      ------------------------------------------------------
------ 0
   EU548043.1      ------------------------------------------------------
------ 0
   EU548047.1      ------------------------------------------------------
------ 0
   EU548050.1      ------------------------------------------------------
------ 0
   EU548048.1      ------------------------------------------------------
------ 0
   EU548049.1      ------------------------------------------------------
------ 0
   AF207722.1      ------------------------------------------------------
------ 0
   EU548037.1      ------------------------------------------------------
------ 0
   AF207723.1      ------------------------------------------------------
------ 0
   EU548038.1      ------------------------------------------------------
------ 0
   EU548036.1      ------------------------------------------------------
------ 0
   EU548035.1      ------------------------------------------------------
------ 0
   AF207720.1      ------------------------------------------------------
------ 0
   AB601576.1      ------------------------------------------------------
------ 0
   EU548040.1      ------------------------------------------------------
------ 0
   EU548039.1      ------------------------------------------------------
------ 0
   AF207721.1      ------------------------------------------------------
------ 0
```

```
    AF207724.1       ------------------------------------------------------
------ 0
    JX982499.1       ------------------------------------------------------
------ 0
    EU548041.1       ------------------------------------------------------
------ 0
    EU548045.1       ------------------------------------------------------
------ 0
    AF207725.1       ------------------------------------------------------
------ 0
    EU548046.1       ------------------------------------------------------
------ 0
    AF207714.1       ------------------------------------------------------
------ 0
    AF207713.1       ------------------------------------------------------
------ 0
    AF207712.1       ------------------------------------------------------
------ 0
    AY750628.1       ------------------------------------------------------
------ 1044
    EF689084.1       ------------------------------------------------------
------ 0
    EF689085.1       ------------------------------------------------------
------ 0
    AB119070.1       ------------------------------------------------------
------ 956
    EF987742.1       ------------------------------------------------------
------ 0
    AB026105.1       ------------------------------------------------------
------ 0
    MW148603.1
TCAGCTGATTAGCCACTCTGCATGGAGGAAATATCAAATGAGCTCCTGCTATACTATGAG   6360
    AB051263.1       ------------------------------------------------------
------ 0
    AF068544.1       ------------------------------------------------------
------ 0


    JX982502.1       ------------------------------------------------------
------ 0
    JX982501.1       ------------------------------------------------------
------ 0
    JX982498.1       ------------------------------------------------------
------ 0
    JX982497.1       ------------------------------------------------------
------ 0
    JX982495.1       ------------------------------------------------------
------ 0
    JX982496.1       ------------------------------------------------------
------ 0
    JX982500.1       ------------------------------------------------------
------ 0
    EU548051.1       ------------------------------------------------------
------ 0
    EU548044.1       ------------------------------------------------------
------ 0
    EU548042.1       ------------------------------------------------------
------ 0
    EU548043.1       ------------------------------------------------------
------ 0
    EU548047.1       ------------------------------------------------------
------ 0
    EU548050.1       ------------------------------------------------------
------ 0
    EU548048.1       ------------------------------------------------------
------ 0
```

EU548049.1 --
------ 0
AF207722.1 --
------ 0
EU548037.1 --
------ 0
AF207723.1 --
------ 0
EU548038.1 --
------ 0
EU548036.1 --
------ 0
EU548035.1 --
------ 0
AF207720.1 --
------ 0
AB601576.1 --
------ 0
EU548040.1 --
------ 0
EU548039.1 --
------ 0
AF207721.1 --
------ 0
AF207724.1 --
------ 0
JX982499.1 --
------ 0
EU548041.1 --
------ 0
EU548045.1 --
------ 0
AF207725.1 --
------ 0
EU548046.1 --
------ 0
AF207714.1 --
------ 0
AF207713.1 --
------ 0
AF207712.1 --
------ 0
AY750628.1 --
------ 1044
EF689084.1 --
------ 0
EF689085.1 --
------ 0
AB119070.1 --
------ 956
EF987742.1 --
------ 0
AB026105.1 --
------ 0
MW148603.1
CCTTAGGGTTTATTTTTCTATTTACAGTGGGGGGTCTAACGGGCATTGTACTATCTAACT 6420
AB051263.1 --
------ 0
AF068544.1 --
------ 0

JX982502.1 --
------ 0
JX982501.1 --
------ 0

```
    JX982498.1      ------------------------------------------------------
------ 0
    JX982497.1      ------------------------------------------------------
------ 0
    JX982495.1      ------------------------------------------------------
------ 0
    JX982496.1      ------------------------------------------------------
------ 0
    JX982500.1      ------------------------------------------------------
------ 0
    EU548051.1      ------------------------------------------------------
------ 0
    EU548044.1      ------------------------------------------------------
------ 0
    EU548042.1      ------------------------------------------------------
------ 0
    EU548043.1      ------------------------------------------------------
------ 0
    EU548047.1      ------------------------------------------------------
------ 0
    EU548050.1      ------------------------------------------------------
------ 0
    EU548048.1      ------------------------------------------------------
------ 0
    EU548049.1      ------------------------------------------------------
------ 0
    AF207722.1      ------------------------------------------------------
------ 0
    EU548037.1      ------------------------------------------------------
------ 0
    AF207723.1      ------------------------------------------------------
------ 0
    EU548038.1      ------------------------------------------------------
------ 0
    EU548036.1      ------------------------------------------------------
------ 0
    EU548035.1      ------------------------------------------------------
------ 0
    AF207720.1      ------------------------------------------------------
------ 0
    AB601576.1      ------------------------------------------------------
------ 0
    EU548040.1      ------------------------------------------------------
------ 0
    EU548039.1      ------------------------------------------------------
------ 0
    AF207721.1      ------------------------------------------------------
------ 0
    AF207724.1      ------------------------------------------------------
------ 0
    JX982499.1      ------------------------------------------------------
------ 0
    EU548041.1      ------------------------------------------------------
------ 0
    EU548045.1      ------------------------------------------------------
------ 0
    AF207725.1      ------------------------------------------------------
------ 0
    EU548046.1      ------------------------------------------------------
------ 0
    AF207714.1      ------------------------------------------------------
------ 0
    AF207713.1      ------------------------------------------------------
------ 0
    AF207712.1      ------------------------------------------------------
------ 0
```

AY750628.1 --
------ 1044
EF689084.1 --
------ 0
EF689085.1 --
------ 0
AB119070.1 --
------ 956
EF987742.1 --
------ 0
AB026105.1 --
------ 0
MW148603.1
CATCACTAGACATTGTCCTTCACGATACGTATTATGTAGTAGCACATTTCCACTACGTCC 6480
AB051263.1 --
------ 0
AF068544.1 --
------ 0

JX982502.1 --
------ 0
JX982501.1 --
------ 0
JX982498.1 --
------ 0
JX982497.1 --
------ 0
JX982495.1 --
------ 0
JX982496.1 --
------ 0
JX982500.1 --
------ 0
EU548051.1 --
------ 0
EU548044.1 --
------ 0
EU548042.1 --
------ 0
EU548043.1 --
------ 0
EU548047.1 --
------ 0
EU548050.1 --
------ 0
EU548048.1 --
------ 0
EU548049.1 --
------ 0
AF207722.1 --
------ 0
EU548037.1 --
------ 0
AF207723.1 --
------ 0
EU548038.1 --
------ 0
EU548036.1 --
------ 0
EU548035.1 --
------ 0
AF207720.1 --
------ 0
AB601576.1 --
------ 0

EU548040.1 --
------ 0
EU548039.1 --
------ 0
AF207721.1 --
------ 0
AF207724.1 --
------ 0
JX982499.1 --
------ 0
EU548041.1 --
------ 0
EU548045.1 --
------ 0
AF207725.1 --
------ 0
EU548046.1 --
------ 0
AF207714.1 --
------ 0
AF207713.1 --
------ 0
AF207712.1 --
------ 0
AY750628.1 --
------ 1044
EF689084.1 --
------ 0
EF689085.1 --
------ 0
AB119070.1 --
------ 956
EF987742.1 --
------ 0
AB026105.1 --
------ 0
MW148603.1
TTTCAATAGGGGCAGTATTTGCAATTATAGGTGGATTCGTCCACTGATTCCCACTATTCA 6540
AB051263.1 --
------ 0
AF068544.1 --
------ 0

JX982502.1 --
------ 0
JX982501.1 --
------ 0
JX982498.1 --
------ 0
JX982497.1 --
------ 0
JX982495.1 --
------ 0
JX982496.1 --
------ 0
JX982500.1 --
------ 0
EU548051.1 --
------ 0
EU548044.1 --
------ 0
EU548042.1 --
------ 0
EU548043.1 --
------ 0

EU548047.1 --
------ 0
EU548050.1 --
------ 0
EU548048.1 --
------ 0
EU548049.1 --
------ 0
AF207722.1 --
------ 0
EU548037.1 --
------ 0
AF207723.1 --
------ 0
EU548038.1 --
------ 0
EU548036.1 --
------ 0
EU548035.1 --
------ 0
AF207720.1 --
------ 0
AB601576.1 --
------ 0
EU548040.1 --
------ 0
EU548039.1 --
------ 0
AF207721.1 --
------ 0
AF207724.1 --
------ 0
JX982499.1 --
------ 0
EU548041.1 --
------ 0
EU548045.1 --
------ 0
AF207725.1 --
------ 0
EU548046.1 --
------ 0
AF207714.1 --
------ 0
AF207713.1 --
------ 0
AF207712.1 --
------ 0
AY750628.1 --
------ 1044
EF689084.1 --
------ 0
EF689085.1 --
------ 0
AB119070.1 --
------ 956
EF987742.1 --
------ 0
AB026105.1 --
------ 0
MW148603.1
CAGGCTATACCCTAAATGATGTATGAGCAAAAATTCATTTCACGATCATATTTGTAGGAG 6600
AB051263.1 --
------ 0
AF068544.1 --
------ 0

```
JX982502.1      ------------------------------------------------------
------ 0
JX982501.1      ------------------------------------------------------
------ 0
JX982498.1      ------------------------------------------------------
------ 0
JX982497.1      ------------------------------------------------------
------ 0
JX982495.1      ------------------------------------------------------
------ 0
JX982496.1      ------------------------------------------------------
------ 0
JX982500.1      ------------------------------------------------------
------ 0
EU548051.1      ------------------------------------------------------
------ 0
EU548044.1      ------------------------------------------------------
------ 0
EU548042.1      ------------------------------------------------------
------ 0
EU548043.1      ------------------------------------------------------
------ 0
EU548047.1      ------------------------------------------------------
------ 0
EU548050.1      ------------------------------------------------------
------ 0
EU548048.1      ------------------------------------------------------
------ 0
EU548049.1      ------------------------------------------------------
------ 0
AF207722.1      ------------------------------------------------------
------ 0
EU548037.1      ------------------------------------------------------
------ 0
AF207723.1      ------------------------------------------------------
------ 0
EU548038.1      ------------------------------------------------------
------ 0
EU548036.1      ------------------------------------------------------
------ 0
EU548035.1      ------------------------------------------------------
------ 0
AF207720.1      ------------------------------------------------------
------ 0
AB601576.1      ------------------------------------------------------
------ 0
EU548040.1      ------------------------------------------------------
------ 0
EU548039.1      ------------------------------------------------------
------ 0
AF207721.1      ------------------------------------------------------
------ 0
AF207724.1      ------------------------------------------------------
------ 0
JX982499.1      ------------------------------------------------------
------ 0
EU548041.1      ------------------------------------------------------
------ 0
EU548045.1      ------------------------------------------------------
------ 0
AF207725.1      ------------------------------------------------------
------ 0
EU548046.1      ------------------------------------------------------
------ 0
```

```
   AF207714.1      ------------------------------------------------------
------ 0
   AF207713.1      ------------------------------------------------------
------ 0
   AF207712.1      ------------------------------------------------------
------ 0
   AY750628.1      ------------------------------------------------------
------ 1044
   EF689084.1      ------------------------------------------------------
------ 0
   EF689085.1      ------------------------------------------------------
------ 0
   AB119070.1      ------------------------------------------------------
------ 956
   EF987742.1      ------------------------------------------------------
------ 0
   AB026105.1      ------------------------------------------------------
------ 0
   MW148603.1
TAAACATAACATTCTTTCCTCAACATTTCCTGGGCCTATCAGGCATACCTCGACGCTACT   6660
   AB051263.1      ------------------------------------------------------
------ 0
   AF068544.1      ------------------------------------------------------
------ 0


   JX982502.1      ------------------------------------------------------
------ 0
   JX982501.1      ------------------------------------------------------
------ 0
   JX982498.1      ------------------------------------------------------
------ 0
   JX982497.1      ------------------------------------------------------
------ 0
   JX982495.1      ------------------------------------------------------
------ 0
   JX982496.1      ------------------------------------------------------
------ 0
   JX982500.1      ------------------------------------------------------
------ 0
   EU548051.1      ------------------------------------------------------
------ 0
   EU548044.1      ------------------------------------------------------
------ 0
   EU548042.1      ------------------------------------------------------
------ 0
   EU548043.1      ------------------------------------------------------
------ 0
   EU548047.1      ------------------------------------------------------
------ 0
   EU548050.1      ------------------------------------------------------
------ 0
   EU548048.1      ------------------------------------------------------
------ 0
   EU548049.1      ------------------------------------------------------
------ 0
   AF207722.1      ------------------------------------------------------
------ 0
   EU548037.1      ------------------------------------------------------
------ 0
   AF207723.1      ------------------------------------------------------
------ 0
   EU548038.1      ------------------------------------------------------
------ 0
   EU548036.1      ------------------------------------------------------
------ 0
```

```
   EU548035.1      ------------------------------------------------------
------ 0
   AF207720.1      ------------------------------------------------------
------ 0
   AB601576.1      ------------------------------------------------------
------ 0
   EU548040.1      ------------------------------------------------------
------ 0
   EU548039.1      ------------------------------------------------------
------ 0
   AF207721.1      ------------------------------------------------------
------ 0
   AF207724.1      ------------------------------------------------------
------ 0
   JX982499.1      ------------------------------------------------------
------ 0
   EU548041.1      ------------------------------------------------------
------ 0
   EU548045.1      ------------------------------------------------------
------ 0
   AF207725.1      ------------------------------------------------------
------ 0
   EU548046.1      ------------------------------------------------------
------ 0
   AF207714.1      ------------------------------------------------------
------ 0
   AF207713.1      ------------------------------------------------------
------ 0
   AF207712.1      ------------------------------------------------------
------ 0
   AY750628.1      ------------------------------------------------------
------ 1044
   EF689084.1      ------------------------------------------------------
------ 0
   EF689085.1      ------------------------------------------------------
------ 0
   AB119070.1      ------------------------------------------------------
------ 956
   EF987742.1      ------------------------------------------------------
------ 0
   AB026105.1      ------------------------------------------------------
------ 0
   MW148603.1
CTGATTATCCAGATGCTTATACAACATGAAATACAGTGTCCTCCATGGGCTCATTCATCT   6720
   AB051263.1      ------------------------------------------------------
------ 0
   AF068544.1      ------------------------------------------------------
------ 0


   JX982502.1      ------------------------------------------------------
------ 0
   JX982501.1      ------------------------------------------------------
------ 0
   JX982498.1      ------------------------------------------------------
------ 0
   JX982497.1      ------------------------------------------------------
------ 0
   JX982495.1      ------------------------------------------------------
------ 0
   JX982496.1      ------------------------------------------------------
------ 0
   JX982500.1      ------------------------------------------------------
------ 0
   EU548051.1      ------------------------------------------------------
------ 0
```

```
    EU548044.1        ------------------------------------------------------
------ 0
    EU548042.1        ------------------------------------------------------
------ 0
    EU548043.1        ------------------------------------------------------
------ 0
    EU548047.1        ------------------------------------------------------
------ 0
    EU548050.1        ------------------------------------------------------
------ 0
    EU548048.1        ------------------------------------------------------
------ 0
    EU548049.1        ------------------------------------------------------
------ 0
    AF207722.1        ------------------------------------------------------
------ 0
    EU548037.1        ------------------------------------------------------
------ 0
    AF207723.1        ------------------------------------------------------
------ 0
    EU548038.1        ------------------------------------------------------
------ 0
    EU548036.1        ------------------------------------------------------
------ 0
    EU548035.1        ------------------------------------------------------
------ 0
    AF207720.1        ------------------------------------------------------
------ 0
    AB601576.1        ------------------------------------------------------
------ 0
    EU548040.1        ------------------------------------------------------
------ 0
    EU548039.1        ------------------------------------------------------
------ 0
    AF207721.1        ------------------------------------------------------
------ 0
    AF207724.1        ------------------------------------------------------
------ 0
    JX982499.1        ------------------------------------------------------
------ 0
    EU548041.1        ------------------------------------------------------
------ 0
    EU548045.1        ------------------------------------------------------
------ 0
    AF207725.1        ------------------------------------------------------
------ 0
    EU548046.1        ------------------------------------------------------
------ 0
    AF207714.1        ------------------------------------------------------
------ 0
    AF207713.1        ------------------------------------------------------
------ 0
    AF207712.1        ------------------------------------------------------
------ 0
    AY750628.1        ------------------------------------------------------
------ 1044
    EF689084.1        ------------------------------------------------------
------ 0
    EF689085.1        ------------------------------------------------------
------ 0
    AB119070.1        ------------------------------------------------------
------ 956
    EF987742.1        ------------------------------------------------------
------ 0
    AB026105.1        ------------------------------------------------------
------ 0
```

```
    MW148603.1
CATTAACGGCAGTCATACTAATGATCTTCATGATTTGAGAAGCTTTCGCATCCAAACGAG    6780
    AB051263.1       ------------------------------------------------------
------ 0
    AF068544.1       ------------------------------------------------------
------ 0


    JX982502.1       ------------------------------------------------------
------ 0
    JX982501.1       ------------------------------------------------------
------ 0
    JX982498.1       ------------------------------------------------------
------ 0
    JX982497.1       ------------------------------------------------------
------ 0
    JX982495.1       ------------------------------------------------------
------ 0
    JX982496.1       ------------------------------------------------------
------ 0
    JX982500.1       ------------------------------------------------------
------ 0
    EU548051.1       ------------------------------------------------------
------ 0
    EU548044.1       ------------------------------------------------------
------ 0
    EU548042.1       ------------------------------------------------------
------ 0
    EU548043.1       ------------------------------------------------------
------ 0
    EU548047.1       ------------------------------------------------------
------ 0
    EU548050.1       ------------------------------------------------------
------ 0
    EU548048.1       ------------------------------------------------------
------ 0
    EU548049.1       ------------------------------------------------------
------ 0
    AF207722.1       ------------------------------------------------------
------ 0
    EU548037.1       ------------------------------------------------------
------ 0
    AF207723.1       ------------------------------------------------------
------ 0
    EU548038.1       ------------------------------------------------------
------ 0
    EU548036.1       ------------------------------------------------------
------ 0
    EU548035.1       ------------------------------------------------------
------ 0
    AF207720.1       ------------------------------------------------------
------ 0
    AB601576.1       ------------------------------------------------------
------ 0
    EU548040.1       ------------------------------------------------------
------ 0
    EU548039.1       ------------------------------------------------------
------ 0
    AF207721.1       ------------------------------------------------------
------ 0
    AF207724.1       ------------------------------------------------------
------ 0
    JX982499.1       ------------------------------------------------------
------ 0
    EU548041.1       ------------------------------------------------------
------ 0
```

```
   EU548045.1      ------------------------------------------------------
------ 0
   AF207725.1      ------------------------------------------------------
------ 0
   EU548046.1      ------------------------------------------------------
------ 0
   AF207714.1      ------------------------------------------------------
------ 0
   AF207713.1      ------------------------------------------------------
------ 0
   AF207712.1      ------------------------------------------------------
------ 0
   AY750628.1      ------------------------------------------------------
------ 1044
   EF689084.1      ------------------------------------------------------
------ 0
   EF689085.1      ------------------------------------------------------
------ 0
   AB119070.1      ------------------------------------------------------
------ 956
   EF987742.1      ------------------------------------------------------
------ 0
   AB026105.1      ------------------------------------------------------
------ 0
   MW148603.1
AAGTATTGACAGTTGAATTAACCTCAACTAACATTGAATGATTGCATGGGTGTCCCCCTC   6840
   AB051263.1      ------------------------------------------------------
------ 0
   AF068544.1      ------------------------------------------------------
------ 0


   JX982502.1      ------------------------------------------------------
------ 0
   JX982501.1      ------------------------------------------------------
------ 0
   JX982498.1      ------------------------------------------------------
------ 0
   JX982497.1      ------------------------------------------------------
------ 0
   JX982495.1      ------------------------------------------------------
------ 0
   JX982496.1      ------------------------------------------------------
------ 0
   JX982500.1      ------------------------------------------------------
------ 0
   EU548051.1      ------------------------------------------------------
------ 0
   EU548044.1      ------------------------------------------------------
------ 0
   EU548042.1      ------------------------------------------------------
------ 0
   EU548043.1      ------------------------------------------------------
------ 0
   EU548047.1      ------------------------------------------------------
------ 0
   EU548050.1      ------------------------------------------------------
------ 0
   EU548048.1      ------------------------------------------------------
------ 0
   EU548049.1      ------------------------------------------------------
------ 0
   AF207722.1      ------------------------------------------------------
------ 0
   EU548037.1      ------------------------------------------------------
------ 0
```

```
    AF207723.1        ------------------------------------------------------
------ 0
    EU548038.1        ------------------------------------------------------
------ 0
    EU548036.1        ------------------------------------------------------
------ 0
    EU548035.1        ------------------------------------------------------
------ 0
    AF207720.1        ------------------------------------------------------
------ 0
    AB601576.1        ------------------------------------------------------
------ 0
    EU548040.1        ------------------------------------------------------
------ 0
    EU548039.1        ------------------------------------------------------
------ 0
    AF207721.1        ------------------------------------------------------
------ 0
    AF207724.1        ------------------------------------------------------
------ 0
    JX982499.1        ------------------------------------------------------
------ 0
    EU548041.1        ------------------------------------------------------
------ 0
    EU548045.1        ------------------------------------------------------
------ 0
    AF207725.1        ------------------------------------------------------
------ 0
    EU548046.1        ------------------------------------------------------
------ 0
    AF207714.1        ------------------------------------------------------
------ 0
    AF207713.1        ------------------------------------------------------
------ 0
    AF207712.1        ------------------------------------------------------
------ 0
    AY750628.1        ------------------------------------------------------
------ 1044
    EF689084.1        ------------------------------------------------------
------ 0
    EF689085.1        ------------------------------------------------------
------ 0
    AB119070.1        ------------------------------------------------------
------ 956
    EF987742.1        ------------------------------------------------------
------ 0
    AB026105.1        ------------------------------------------------------
------ 0
    MW148603.1
CATACCACACATTCGAAGAACCAACCTACGTACTATCAAAATAAGAAAGGAAGGAATCGA   6900
    AB051263.1        ------------------------------------------------------
------ 0
    AF068544.1        ------------------------------------------------------
------ 0


    JX982502.1        ------------------------------------------------------
------ 0
    JX982501.1        ------------------------------------------------------
------ 0
    JX982498.1        ------------------------------------------------------
------ 0
    JX982497.1        ------------------------------------------------------
------ 0
    JX982495.1        ------------------------------------------------------
------ 0
```

```
    JX982496.1       ------------------------------------------------------
------ 0
    JX982500.1       ------------------------------------------------------
------ 0
    EU548051.1       ------------------------------------------------------
------ 0
    EU548044.1       ------------------------------------------------------
------ 0
    EU548042.1       ------------------------------------------------------
------ 0
    EU548043.1       ------------------------------------------------------
------ 0
    EU548047.1       ------------------------------------------------------
------ 0
    EU548050.1       ------------------------------------------------------
------ 0
    EU548048.1       ------------------------------------------------------
------ 0
    EU548049.1       ------------------------------------------------------
------ 0
    AF207722.1       ------------------------------------------------------
------ 0
    EU548037.1       ------------------------------------------------------
------ 0
    AF207723.1       ------------------------------------------------------
------ 0
    EU548038.1       ------------------------------------------------------
------ 0
    EU548036.1       ------------------------------------------------------
------ 0
    EU548035.1       ------------------------------------------------------
------ 0
    AF207720.1       ------------------------------------------------------
------ 0
    AB601576.1       ------------------------------------------------------
------ 0
    EU548040.1       ------------------------------------------------------
------ 0
    EU548039.1       ------------------------------------------------------
------ 0
    AF207721.1       ------------------------------------------------------
------ 0
    AF207724.1       ------------------------------------------------------
------ 0
    JX982499.1       ------------------------------------------------------
------ 0
    EU548041.1       ------------------------------------------------------
------ 0
    EU548045.1       ------------------------------------------------------
------ 0
    AF207725.1       ------------------------------------------------------
------ 0
    EU548046.1       ------------------------------------------------------
------ 0
    AF207714.1       ------------------------------------------------------
------ 0
    AF207713.1       ------------------------------------------------------
------ 0
    AF207712.1       ------------------------------------------------------
------ 0
    AY750628.1       ------------------------------------------------------
------ 1044
    EF689084.1       ------------------------------------------------------
------ 0
    EF689085.1       ------------------------------------------------------
------ 0
```

```
    AB119070.1      ------------------------------------------------------
------ 956
    EF987742.1      ------------------------------------------------------
------ 0
    AB026105.1      ------------------------------------------------------
------ 0
    MW148603.1      
ACCCCCTAAGACTGGTTTCAAGCCAATATCATAACCACTATGTCTTTCTCGATAAGAGGT   6960
    AB051263.1      ------------------------------------------------------
------ 0
    AF068544.1      ------------------------------------------------------
------ 0


    JX982502.1      ------------------------------------------------------
------ 0
    JX982501.1      ------------------------------------------------------
------ 0
    JX982498.1      ------------------------------------------------------
------ 0
    JX982497.1      ------------------------------------------------------
------ 0
    JX982495.1      ------------------------------------------------------
------ 0
    JX982496.1      ------------------------------------------------------
------ 0
    JX982500.1      ------------------------------------------------------
------ 0
    EU548051.1      ------------------------------------------------------
------ 0
    EU548044.1      ------------------------------------------------------
------ 0
    EU548042.1      ------------------------------------------------------
------ 0
    EU548043.1      ------------------------------------------------------
------ 0
    EU548047.1      ------------------------------------------------------
------ 0
    EU548050.1      ------------------------------------------------------
------ 0
    EU548048.1      ------------------------------------------------------
------ 0
    EU548049.1      ------------------------------------------------------
------ 0
    AF207722.1      ------------------------------------------------------
------ 0
    EU548037.1      ------------------------------------------------------
------ 0
    AF207723.1      ------------------------------------------------------
------ 0
    EU548038.1      ------------------------------------------------------
------ 0
    EU548036.1      ------------------------------------------------------
------ 0
    EU548035.1      ------------------------------------------------------
------ 0
    AF207720.1      ------------------------------------------------------
------ 0
    AB601576.1      ------------------------------------------------------
------ 0
    EU548040.1      ------------------------------------------------------
------ 0
    EU548039.1      ------------------------------------------------------
------ 0
    AF207721.1      ------------------------------------------------------
------ 0
```

```
    AF207724.1        ------------------------------------------------------
------ 0
    JX982499.1        ------------------------------------------------------
------ 0
    EU548041.1        ------------------------------------------------------
------ 0
    EU548045.1        ------------------------------------------------------
------ 0
    AF207725.1        ------------------------------------------------------
------ 0
    EU548046.1        ------------------------------------------------------
------ 0
    AF207714.1        ------------------------------------------------------
------ 0
    AF207713.1        ------------------------------------------------------
------ 0
    AF207712.1        ------------------------------------------------------
------ 0
    AY750628.1        ------------------------------------------------------
------ 1044
    EF689084.1        ------------------------------------------------------
------ 0
    EF689085.1        ------------------------------------------------------
------ 0
    AB119070.1        ------------------------------------------------------
------ 956
    EF987742.1        ------------------------------------------------------
------ 0
    AB026105.1        ------------------------------------------------------
------ 0
    MW148603.1
ATTAGTAAAAATTACATGACTTTGTCAAAGTCAAATTATAGGTGAAAGTCCTTTATATCT   7020
    AB051263.1        ------------------------------------------------------
------ 0
    AF068544.1        ------------------------------------------------------
------ 0


    JX982502.1        ------------------------------------------------------
------ 0
    JX982501.1        ------------------------------------------------------
------ 0
    JX982498.1        ------------------------------------------------------
------ 0
    JX982497.1        ------------------------------------------------------
------ 0
    JX982495.1        ------------------------------------------------------
------ 0
    JX982496.1        ------------------------------------------------------
------ 0
    JX982500.1        ------------------------------------------------------
------ 0
    EU548051.1        ------------------------------------------------------
------ 0
    EU548044.1        ------------------------------------------------------
------ 0
    EU548042.1        ------------------------------------------------------
------ 0
    EU548043.1        ------------------------------------------------------
------ 0
    EU548047.1        ------------------------------------------------------
------ 0
    EU548050.1        ------------------------------------------------------
------ 0
    EU548048.1        ------------------------------------------------------
------ 0
```

EU548049.1 --
------ 0
AF207722.1 --
------ 0
EU548037.1 --
------ 0
AF207723.1 --
------ 0
EU548038.1 --
------ 0
EU548036.1 --
------ 0
EU548035.1 --
------ 0
AF207720.1 --
------ 0
AB601576.1 --
------ 0
EU548040.1 --
------ 0
EU548039.1 --
------ 0
AF207721.1 --
------ 0
AF207724.1 --
------ 0
JX982499.1 --
------ 0
EU548041.1 --
------ 0
EU548045.1 --
------ 0
AF207725.1 --
------ 0
EU548046.1 --
------ 0
AF207714.1 --
------ 0
AF207713.1 --
------ 0
AF207712.1 --
------ 0
AY750628.1 --
------ 1044
EF689084.1 --
------ 0
EF689085.1 --
------ 0
AB119070.1 --
------ 956
EF987742.1 --
------ 0
AB026105.1 --
------ 0
MW148603.1
CTATGGCATACCCTTTCCAAATAGGCCTCCAAGATGCAGCCTCTCCTATCATAGAGGAAC 7080
AB051263.1 --
------ 0
AF068544.1 --
------ 0

JX982502.1 --
------ 0
JX982501.1 --
------ 0

```
    JX982498.1      ------------------------------------------------------
------ 0
    JX982497.1      ------------------------------------------------------
------ 0
    JX982495.1      ------------------------------------------------------
------ 0
    JX982496.1      ------------------------------------------------------
------ 0
    JX982500.1      ------------------------------------------------------
------ 0
    EU548051.1      ------------------------------------------------------
------ 0
    EU548044.1      ------------------------------------------------------
------ 0
    EU548042.1      ------------------------------------------------------
------ 0
    EU548043.1      ------------------------------------------------------
------ 0
    EU548047.1      ------------------------------------------------------
------ 0
    EU548050.1      ------------------------------------------------------
------ 0
    EU548048.1      ------------------------------------------------------
------ 0
    EU548049.1      ------------------------------------------------------
------ 0
    AF207722.1      ------------------------------------------------------
------ 0
    EU548037.1      ------------------------------------------------------
------ 0
    AF207723.1      ------------------------------------------------------
------ 0
    EU548038.1      ------------------------------------------------------
------ 0
    EU548036.1      ------------------------------------------------------
------ 0
    EU548035.1      ------------------------------------------------------
------ 0
    AF207720.1      ------------------------------------------------------
------ 0
    AB601576.1      ------------------------------------------------------
------ 0
    EU548040.1      ------------------------------------------------------
------ 0
    EU548039.1      ------------------------------------------------------
------ 0
    AF207721.1      ------------------------------------------------------
------ 0
    AF207724.1      ------------------------------------------------------
------ 0
    JX982499.1      ------------------------------------------------------
------ 0
    EU548041.1      ------------------------------------------------------
------ 0
    EU548045.1      ------------------------------------------------------
------ 0
    AF207725.1      ------------------------------------------------------
------ 0
    EU548046.1      ------------------------------------------------------
------ 0
    AF207714.1      ------------------------------------------------------
------ 0
    AF207713.1      ------------------------------------------------------
------ 0
    AF207712.1      ------------------------------------------------------
------ 0
```

```
    AY750628.1      ------------------------------------------------------
------ 1044
    EF689084.1      ------------------------------------------------------
------ 0
    EF689085.1      ------------------------------------------------------
------ 0
    AB119070.1      ------------------------------------------------------
------ 956
    EF987742.1      ------------------------------------------------------
------ 0
    AB026105.1      ------------------------------------------------------
------ 0
    MW148603.1
TTCTACACTTTCACGATCATACACTAATAATTGTATTTCTAATTAGTTCTCTTGTACTTT   7140
    AB051263.1      ------------------------------------------------------
------ 0
    AF068544.1      ------------------------------------------------------
------ 0


    JX982502.1      ------------------------------------------------------
------ 0
    JX982501.1      ------------------------------------------------------
------ 0
    JX982498.1      ------------------------------------------------------
------ 0
    JX982497.1      ------------------------------------------------------
------ 0
    JX982495.1      ------------------------------------------------------
------ 0
    JX982496.1      ------------------------------------------------------
------ 0
    JX982500.1      ------------------------------------------------------
------ 0
    EU548051.1      ------------------------------------------------------
------ 0
    EU548044.1      ------------------------------------------------------
------ 0
    EU548042.1      ------------------------------------------------------
------ 0
    EU548043.1      ------------------------------------------------------
------ 0
    EU548047.1      ------------------------------------------------------
------ 0
    EU548050.1      ------------------------------------------------------
------ 0
    EU548048.1      ------------------------------------------------------
------ 0
    EU548049.1      ------------------------------------------------------
------ 0
    AF207722.1      ------------------------------------------------------
------ 0
    EU548037.1      ------------------------------------------------------
------ 0
    AF207723.1      ------------------------------------------------------
------ 0
    EU548038.1      ------------------------------------------------------
------ 0
    EU548036.1      ------------------------------------------------------
------ 0
    EU548035.1      ------------------------------------------------------
------ 0
    AF207720.1      ------------------------------------------------------
------ 0
    AB601576.1      ------------------------------------------------------
------ 0
```

```
   EU548040.1      ------------------------------------------------------
------ 0
   EU548039.1      ------------------------------------------------------
------ 0
   AF207721.1      ------------------------------------------------------
------ 0
   AF207724.1      ------------------------------------------------------
------ 0
   JX982499.1      ------------------------------------------------------
------ 0
   EU548041.1      ------------------------------------------------------
------ 0
   EU548045.1      ------------------------------------------------------
------ 0
   AF207725.1      ------------------------------------------------------
------ 0
   EU548046.1      ------------------------------------------------------
------ 0
   AF207714.1      ------------------------------------------------------
------ 0
   AF207713.1      ------------------------------------------------------
------ 0
   AF207712.1      ------------------------------------------------------
------ 0
   AY750628.1      ------------------------------------------------------
------ 1044
   EF689084.1      ------------------------------------------------------
------ 0
   EF689085.1      ------------------------------------------------------
------ 0
   AB119070.1      ------------------------------------------------------
------ 956
   EF987742.1      ------------------------------------------------------
------ 0
   AB026105.1      ------------------------------------------------------
------ 0
   MW148603.1
ACATTATTTCAGTAATACTAACTACCAAGCTTACGCATACAAGTACTATAGACGCCCAAG   7200
   AB051263.1      ------------------------------------------------------
------ 0
   AF068544.1      ------------------------------------------------------
------ 0


   JX982502.1      ------------------------------------------------------
------ 0
   JX982501.1      ------------------------------------------------------
------ 0
   JX982498.1      ------------------------------------------------------
------ 0
   JX982497.1      ------------------------------------------------------
------ 0
   JX982495.1      ------------------------------------------------------
------ 0
   JX982496.1      ------------------------------------------------------
------ 0
   JX982500.1      ------------------------------------------------------
------ 0
   EU548051.1      ------------------------------------------------------
------ 0
   EU548044.1      ------------------------------------------------------
------ 0
   EU548042.1      ------------------------------------------------------
------ 0
   EU548043.1      ------------------------------------------------------
------ 0
```

```
   EU548047.1      ------------------------------------------------------
------ 0
   EU548050.1      ------------------------------------------------------
------ 0
   EU548048.1      ------------------------------------------------------
------ 0
   EU548049.1      ------------------------------------------------------
------ 0
   AF207722.1      ------------------------------------------------------
------ 0
   EU548037.1      ------------------------------------------------------
------ 0
   AF207723.1      ------------------------------------------------------
------ 0
   EU548038.1      ------------------------------------------------------
------ 0
   EU548036.1      ------------------------------------------------------
------ 0
   EU548035.1      ------------------------------------------------------
------ 0
   AF207720.1      ------------------------------------------------------
------ 0
   AB601576.1      ------------------------------------------------------
------ 0
   EU548040.1      ------------------------------------------------------
------ 0
   EU548039.1      ------------------------------------------------------
------ 0
   AF207721.1      ------------------------------------------------------
------ 0
   AF207724.1      ------------------------------------------------------
------ 0
   JX982499.1      ------------------------------------------------------
------ 0
   EU548041.1      ------------------------------------------------------
------ 0
   EU548045.1      ------------------------------------------------------
------ 0
   AF207725.1      ------------------------------------------------------
------ 0
   EU548046.1      ------------------------------------------------------
------ 0
   AF207714.1      ------------------------------------------------------
------ 0
   AF207713.1      ------------------------------------------------------
------ 0
   AF207712.1      ------------------------------------------------------
------ 0
   AY750628.1      ------------------------------------------------------
------ 1044
   EF689084.1      ------------------------------------------------------
------ 0
   EF689085.1      ------------------------------------------------------
------ 0
   AB119070.1      ------------------------------------------------------
------ 956
   EF987742.1      ------------------------------------------------------
------ 0
   AB026105.1      ------------------------------------------------------
------ 0
   MW148603.1
CAGTTGAAACAATCTGAACCATCCTACCAGCCATTATTTTGATCATAATCGCTCTACCCT   7260
   AB051263.1      ------------------------------------------------------
------ 0
   AF068544.1      ------------------------------------------------------
------ 0
```

```
    JX982502.1        ------------------------------------------------------
------ 0
    JX982501.1        ------------------------------------------------------
------ 0
    JX982498.1        ------------------------------------------------------
------ 0
    JX982497.1        ------------------------------------------------------
------ 0
    JX982495.1        ------------------------------------------------------
------ 0
    JX982496.1        ------------------------------------------------------
------ 0
    JX982500.1        ------------------------------------------------------
------ 0
    EU548051.1        ------------------------------------------------------
------ 0
    EU548044.1        ------------------------------------------------------
------ 0
    EU548042.1        ------------------------------------------------------
------ 0
    EU548043.1        ------------------------------------------------------
------ 0
    EU548047.1        ------------------------------------------------------
------ 0
    EU548050.1        ------------------------------------------------------
------ 0
    EU548048.1        ------------------------------------------------------
------ 0
    EU548049.1        ------------------------------------------------------
------ 0
    AF207722.1        ------------------------------------------------------
------ 0
    EU548037.1        ------------------------------------------------------
------ 0
    AF207723.1        ------------------------------------------------------
------ 0
    EU548038.1        ------------------------------------------------------
------ 0
    EU548036.1        ------------------------------------------------------
------ 0
    EU548035.1        ------------------------------------------------------
------ 0
    AF207720.1        ------------------------------------------------------
------ 0
    AB601576.1        ------------------------------------------------------
------ 0
    EU548040.1        ------------------------------------------------------
------ 0
    EU548039.1        ------------------------------------------------------
------ 0
    AF207721.1        ------------------------------------------------------
------ 0
    AF207724.1        ------------------------------------------------------
------ 0
    JX982499.1        ------------------------------------------------------
------ 0
    EU548041.1        ------------------------------------------------------
------ 0
    EU548045.1        ------------------------------------------------------
------ 0
    AF207725.1        ------------------------------------------------------
------ 0
    EU548046.1        ------------------------------------------------------
------ 0
```

AF207714.1 --
------ 0
AF207713.1 --
------ 0
AF207712.1 --
------ 0
AY750628.1 --
------ 1044
EF689084.1 --
------ 0
EF689085.1 --
------ 0
AB119070.1 --
------ 956
EF987742.1 --
------ 0
AB026105.1 --
------ 0
MW148603.1
CGCTACGAATTCTCTATATGATAGACGAGATCAATAACCCCTCTTTAACCGTAAAAACTA 7320
AB051263.1 --
------ 0
AF068544.1 --
------ 0

JX982502.1 --
------ 0
JX982501.1 --
------ 0
JX982498.1 --
------ 0
JX982497.1 --
------ 0
JX982495.1 --
------ 0
JX982496.1 --
------ 0
JX982500.1 --
------ 0
EU548051.1 --
------ 0
EU548044.1 --
------ 0
EU548042.1 --
------ 0
EU548043.1 --
------ 0
EU548047.1 --
------ 0
EU548050.1 --
------ 0
EU548048.1 --
------ 0
EU548049.1 --
------ 0
AF207722.1 --
------ 0
EU548037.1 --
------ 0
AF207723.1 --
------ 0
EU548038.1 --
------ 0
EU548036.1 --
------ 0

```
   EU548035.1      ------------------------------------------------------
------ 0
   AF207720.1      ------------------------------------------------------
------ 0
   AB601576.1      ------------------------------------------------------
------ 0
   EU548040.1      ------------------------------------------------------
------ 0
   EU548039.1      ------------------------------------------------------
------ 0
   AF207721.1      ------------------------------------------------------
------ 0
   AF207724.1      ------------------------------------------------------
------ 0
   JX982499.1      ------------------------------------------------------
------ 0
   EU548041.1      ------------------------------------------------------
------ 0
   EU548045.1      ------------------------------------------------------
------ 0
   AF207725.1      ------------------------------------------------------
------ 0
   EU548046.1      ------------------------------------------------------
------ 0
   AF207714.1      ------------------------------------------------------
------ 0
   AF207713.1      ------------------------------------------------------
------ 0
   AF207712.1      ------------------------------------------------------
------ 0
   AY750628.1      ------------------------------------------------------
------ 1044
   EF689084.1      ------------------------------------------------------
------ 0
   EF689085.1      ------------------------------------------------------
------ 0
   AB119070.1      ------------------------------------------------------
------ 956
   EF987742.1      ------------------------------------------------------
------ 0
   AB026105.1      ------------------------------------------------------
------ 0
   MW148603.1
TGGGTCACCAATGATACTGAAGTTATGAATATACAGACTACGAAGACTTAAACTTCGACT   7380
   AB051263.1      ------------------------------------------------------
------ 0
   AF068544.1      ------------------------------------------------------
------ 0


   JX982502.1      ------------------------------------------------------
------ 0
   JX982501.1      ------------------------------------------------------
------ 0
   JX982498.1      ------------------------------------------------------
------ 0
   JX982497.1      ------------------------------------------------------
------ 0
   JX982495.1      ------------------------------------------------------
------ 0
   JX982496.1      ------------------------------------------------------
------ 0
   JX982500.1      ------------------------------------------------------
------ 0
   EU548051.1      ------------------------------------------------------
------ 0
```

```
    EU548044.1      ------------------------------------------------------
------ 0
    EU548042.1      ------------------------------------------------------
------ 0
    EU548043.1      ------------------------------------------------------
------ 0
    EU548047.1      ------------------------------------------------------
------ 0
    EU548050.1      ------------------------------------------------------
------ 0
    EU548048.1      ------------------------------------------------------
------ 0
    EU548049.1      ------------------------------------------------------
------ 0
    AF207722.1      ------------------------------------------------------
------ 0
    EU548037.1      ------------------------------------------------------
------ 0
    AF207723.1      ------------------------------------------------------
------ 0
    EU548038.1      ------------------------------------------------------
------ 0
    EU548036.1      ------------------------------------------------------
------ 0
    EU548035.1      ------------------------------------------------------
------ 0
    AF207720.1      ------------------------------------------------------
------ 0
    AB601576.1      ------------------------------------------------------
------ 0
    EU548040.1      ------------------------------------------------------
------ 0
    EU548039.1      ------------------------------------------------------
------ 0
    AF207721.1      ------------------------------------------------------
------ 0
    AF207724.1      ------------------------------------------------------
------ 0
    JX982499.1      ------------------------------------------------------
------ 0
    EU548041.1      ------------------------------------------------------
------ 0
    EU548045.1      ------------------------------------------------------
------ 0
    AF207725.1      ------------------------------------------------------
------ 0
    EU548046.1      ------------------------------------------------------
------ 0
    AF207714.1      ------------------------------------------------------
------ 0
    AF207713.1      ------------------------------------------------------
------ 0
    AF207712.1      ------------------------------------------------------
------ 0
    AY750628.1      ------------------------------------------------------
------ 1044
    EF689084.1      ------------------------------------------------------
------ 0
    EF689085.1      ------------------------------------------------------
------ 0
    AB119070.1      ------------------------------------------------------
------ 956
    EF987742.1      ------------------------------------------------------
------ 0
    AB026105.1      ------------------------------------------------------
------ 0
```

MW148603.1
CCTACATAATCCCAACTCAAGAACTAAAACCAGGAGAACTACGACTTCTAGAAGTGGATA 7440
AB051263.1 --
------ 0
AF068544.1 --
------ 0

JX982502.1 --
------ 0
JX982501.1 --
------ 0
JX982498.1 --
------ 0
JX982497.1 --
------ 0
JX982495.1 --
------ 0
JX982496.1 --
------ 0
JX982500.1 --
------ 0
EU548051.1 --
------ 0
EU548044.1 --
------ 0
EU548042.1 --
------ 0
EU548043.1 --
------ 0
EU548047.1 --
------ 0
EU548050.1 --
------ 0
EU548048.1 --
------ 0
EU548049.1 --
------ 0
AF207722.1 --
------ 0
EU548037.1 --
------ 0
AF207723.1 --
------ 0
EU548038.1 --
------ 0
EU548036.1 --
------ 0
EU548035.1 --
------ 0
AF207720.1 --
------ 0
AB601576.1 --
------ 0
EU548040.1 --
------ 0
EU548039.1 --
------ 0
AF207721.1 --
------ 0
AF207724.1 --
------ 0
JX982499.1 --
------ 0
EU548041.1 --
------ 0

```
    EU548045.1       ------------------------------------------------------
------ 0
    AF207725.1       ------------------------------------------------------
------ 0
    EU548046.1       ------------------------------------------------------
------ 0
    AF207714.1       ------------------------------------------------------
------ 0
    AF207713.1       ------------------------------------------------------
------ 0
    AF207712.1       ------------------------------------------------------
------ 0
    AY750628.1       ------------------------------------------------------
------ 1044
    EF689084.1       ------------------------------------------------------
------ 0
    EF689085.1       ------------------------------------------------------
------ 0
    AB119070.1       ------------------------------------------------------
------ 956
    EF987742.1       ------------------------------------------------------
------ 0
    AB026105.1       ------------------------------------------------------
------ 0
    MW148603.1
ATCGAGTAGTGCTCCCAATAGAAATAACAATTCGTATACTAATTTCTTCCGAGGATGTAT   7500
    AB051263.1       ------------------------------------------------------
------ 0
    AF068544.1       ------------------------------------------------------
------ 0


    JX982502.1       ------------------------------------------------------
------ 0
    JX982501.1       ------------------------------------------------------
------ 0
    JX982498.1       ------------------------------------------------------
------ 0
    JX982497.1       ------------------------------------------------------
------ 0
    JX982495.1       ------------------------------------------------------
------ 0
    JX982496.1       ------------------------------------------------------
------ 0
    JX982500.1       ------------------------------------------------------
------ 0
    EU548051.1       ------------------------------------------------------
------ 0
    EU548044.1       ------------------------------------------------------
------ 0
    EU548042.1       ------------------------------------------------------
------ 0
    EU548043.1       ------------------------------------------------------
------ 0
    EU548047.1       ------------------------------------------------------
------ 0
    EU548050.1       ------------------------------------------------------
------ 0
    EU548048.1       ------------------------------------------------------
------ 0
    EU548049.1       ------------------------------------------------------
------ 0
    AF207722.1       ------------------------------------------------------
------ 0
    EU548037.1       ------------------------------------------------------
------ 0
```

```
    AF207723.1       ------------------------------------------------------
------ 0
    EU548038.1       ------------------------------------------------------
------ 0
    EU548036.1       ------------------------------------------------------
------ 0
    EU548035.1       ------------------------------------------------------
------ 0
    AF207720.1       ------------------------------------------------------
------ 0
    AB601576.1       ------------------------------------------------------
------ 0
    EU548040.1       ------------------------------------------------------
------ 0
    EU548039.1       ------------------------------------------------------
------ 0
    AF207721.1       ------------------------------------------------------
------ 0
    AF207724.1       ------------------------------------------------------
------ 0
    JX982499.1       ------------------------------------------------------
------ 0
    EU548041.1       ------------------------------------------------------
------ 0
    EU548045.1       ------------------------------------------------------
------ 0
    AF207725.1       ------------------------------------------------------
------ 0
    EU548046.1       ------------------------------------------------------
------ 0
    AF207714.1       ------------------------------------------------------
------ 0
    AF207713.1       ------------------------------------------------------
------ 0
    AF207712.1       ------------------------------------------------------
------ 0
    AY750628.1       ------------------------------------------------------
------ 1044
    EF689084.1       ------------------------------------------------------
------ 0
    EF689085.1       ------------------------------------------------------
------ 0
    AB119070.1       ------------------------------------------------------
------ 956
    EF987742.1       ------------------------------------------------------
------ 0
    AB026105.1       ------------------------------------------------------
------ 0
    MW148603.1
TACACTCATGAGCCGTCCCATCCCTAGGATTAAAAACTGATGCTATCCCAGGACGCCTTA   7560
    AB051263.1       ------------------------------------------------------
------ 0
    AF068544.1       ------------------------------------------------------
------ 0


    JX982502.1       ------------------------------------------------------
------ 0
    JX982501.1       ------------------------------------------------------
------ 0
    JX982498.1       ------------------------------------------------------
------ 0
    JX982497.1       ------------------------------------------------------
------ 0
    JX982495.1       ------------------------------------------------------
------ 0
```

```
   JX982496.1       ------------------------------------------------------
------ 0
   JX982500.1       ------------------------------------------------------
------ 0
   EU548051.1       ------------------------------------------------------
------ 0
   EU548044.1       ------------------------------------------------------
------ 0
   EU548042.1       ------------------------------------------------------
------ 0
   EU548043.1       ------------------------------------------------------
------ 0
   EU548047.1       ------------------------------------------------------
------ 0
   EU548050.1       ------------------------------------------------------
------ 0
   EU548048.1       ------------------------------------------------------
------ 0
   EU548049.1       ------------------------------------------------------
------ 0
   AF207722.1       ------------------------------------------------------
------ 0
   EU548037.1       ------------------------------------------------------
------ 0
   AF207723.1       ------------------------------------------------------
------ 0
   EU548038.1       ------------------------------------------------------
------ 0
   EU548036.1       ------------------------------------------------------
------ 0
   EU548035.1       ------------------------------------------------------
------ 0
   AF207720.1       ------------------------------------------------------
------ 0
   AB601576.1       ------------------------------------------------------
------ 0
   EU548040.1       ------------------------------------------------------
------ 0
   EU548039.1       ------------------------------------------------------
------ 0
   AF207721.1       ------------------------------------------------------
------ 0
   AF207724.1       ------------------------------------------------------
------ 0
   JX982499.1       ------------------------------------------------------
------ 0
   EU548041.1       ------------------------------------------------------
------ 0
   EU548045.1       ------------------------------------------------------
------ 0
   AF207725.1       ------------------------------------------------------
------ 0
   EU548046.1       ------------------------------------------------------
------ 0
   AF207714.1       ------------------------------------------------------
------ 0
   AF207713.1       ------------------------------------------------------
------ 0
   AF207712.1       ------------------------------------------------------
------ 0
   AY750628.1       ------------------------------------------------------
------ 1044
   EF689084.1       ------------------------------------------------------
------ 0
   EF689085.1       ------------------------------------------------------
------ 0
```

AB119070.1 --
------ 956
EF987742.1 --
------ 0
AB026105.1 --
------ 0
MW148603.1
ACCAAACTACTATTATAGCCATGCGACCGGGACTATACTACGGCCAATGCTCTGAAATCT 7620
AB051263.1 --
------ 0
AF068544.1 --
------ 0

JX982502.1 --
------ 0
JX982501.1 --
------ 0
JX982498.1 --
------ 0
JX982497.1 --
------ 0
JX982495.1 --
------ 0
JX982496.1 --
------ 0
JX982500.1 --
------ 0
EU548051.1 --
------ 0
EU548044.1 --
------ 0
EU548042.1 --
------ 0
EU548043.1 --
------ 0
EU548047.1 --
------ 0
EU548050.1 --
------ 0
EU548048.1 --
------ 0
EU548049.1 --
------ 0
AF207722.1 --
------ 0
EU548037.1 --
------ 0
AF207723.1 --
------ 0
EU548038.1 --
------ 0
EU548036.1 --
------ 0
EU548035.1 --
------ 0
AF207720.1 --
------ 0
AB601576.1 --
------ 0
EU548040.1 --
------ 0
EU548039.1 --
------ 0
AF207721.1 --
------ 0

```
    AF207724.1       ------------------------------------------------------
------ 0
    JX982499.1       ------------------------------------------------------
------ 0
    EU548041.1       ------------------------------------------------------
------ 0
    EU548045.1       ------------------------------------------------------
------ 0
    AF207725.1       ------------------------------------------------------
------ 0
    EU548046.1       ------------------------------------------------------
------ 0
    AF207714.1       ------------------------------------------------------
------ 0
    AF207713.1       ------------------------------------------------------
------ 0
    AF207712.1       ------------------------------------------------------
------ 0
    AY750628.1       ------------------------------------------------------
------ 1044
    EF689084.1       ------------------------------------------------------
------ 0
    EF689085.1       ------------------------------------------------------
------ 0
    AB119070.1       ------------------------------------------------------
------ 956
    EF987742.1       ------------------------------------------------------
------ 0
    AB026105.1       ------------------------------------------------------
------ 0
    MW148603.1
GCGGCTCTAATCACAGCTTCATACCTATTGTCCTTGAGCTAGTACCTTTATCATACTTCG   7680
    AB051263.1       ------------------------------------------------------
------ 0
    AF068544.1       ------------------------------------------------------
------ 0


    JX982502.1       ------------------------------------------------------
------ 0
    JX982501.1       ------------------------------------------------------
------ 0
    JX982498.1       ------------------------------------------------------
------ 0
    JX982497.1       ------------------------------------------------------
------ 0
    JX982495.1       ------------------------------------------------------
------ 0
    JX982496.1       ------------------------------------------------------
------ 0
    JX982500.1       ------------------------------------------------------
------ 0
    EU548051.1       ------------------------------------------------------
------ 0
    EU548044.1       ------------------------------------------------------
------ 0
    EU548042.1       ------------------------------------------------------
------ 0
    EU548043.1       ------------------------------------------------------
------ 0
    EU548047.1       ------------------------------------------------------
------ 0
    EU548050.1       ------------------------------------------------------
------ 0
    EU548048.1       ------------------------------------------------------
------ 0
```

```
   EU548049.1      ------------------------------------------------------
------ 0
   AF207722.1      ------------------------------------------------------
------ 0
   EU548037.1      ------------------------------------------------------
------ 0
   AF207723.1      ------------------------------------------------------
------ 0
   EU548038.1      ------------------------------------------------------
------ 0
   EU548036.1      ------------------------------------------------------
------ 0
   EU548035.1      ------------------------------------------------------
------ 0
   AF207720.1      ------------------------------------------------------
------ 0
   AB601576.1      ------------------------------------------------------
------ 0
   EU548040.1      ------------------------------------------------------
------ 0
   EU548039.1      ------------------------------------------------------
------ 0
   AF207721.1      ------------------------------------------------------
------ 0
   AF207724.1      ------------------------------------------------------
------ 0
   JX982499.1      ------------------------------------------------------
------ 0
   EU548041.1      ------------------------------------------------------
------ 0
   EU548045.1      ------------------------------------------------------
------ 0
   AF207725.1      ------------------------------------------------------
------ 0
   EU548046.1      ------------------------------------------------------
------ 0
   AF207714.1      ------------------------------------------------------
------ 0
   AF207713.1      ------------------------------------------------------
------ 0
   AF207712.1      ------------------------------------------------------
------ 0
   AY750628.1      ------------------------------------------------------
------ 1044
   EF689084.1      ------------------------------------------------------
------ 0
   EF689085.1      ------------------------------------------------------
------ 0
   AB119070.1      ------------------------------------------------------
------ 956
   EF987742.1      ------------------------------------------------------
------ 0
   AB026105.1      ------------------------------------------------------
------ 0
   MW148603.1
AAAAATGATCTGCCTCAATACTATAAATTCACCGAGAAGCTAAATAGCATTAACCTTTTA   7740
   AB051263.1      ------------------------------------------------------
------ 0
   AF068544.1      ------------------------------------------------------
------ 0


   JX982502.1      ------------------------------------------------------
------ 0
   JX982501.1      ------------------------------------------------------
------ 0
```

```
    JX982498.1          ------------------------------------------------------
------ 0
    JX982497.1          ------------------------------------------------------
------ 0
    JX982495.1          ------------------------------------------------------
------ 0
    JX982496.1          ------------------------------------------------------
------ 0
    JX982500.1          ------------------------------------------------------
------ 0
    EU548051.1          ------------------------------------------------------
------ 0
    EU548044.1          ------------------------------------------------------
------ 0
    EU548042.1          ------------------------------------------------------
------ 0
    EU548043.1          ------------------------------------------------------
------ 0
    EU548047.1          ------------------------------------------------------
------ 0
    EU548050.1          ------------------------------------------------------
------ 0
    EU548048.1          ------------------------------------------------------
------ 0
    EU548049.1          ------------------------------------------------------
------ 0
    AF207722.1          ------------------------------------------------------
------ 0
    EU548037.1          ------------------------------------------------------
------ 0
    AF207723.1          ------------------------------------------------------
------ 0
    EU548038.1          ------------------------------------------------------
------ 0
    EU548036.1          ------------------------------------------------------
------ 0
    EU548035.1          ------------------------------------------------------
------ 0
    AF207720.1          ------------------------------------------------------
------ 0
    AB601576.1          ------------------------------------------------------
------ 0
    EU548040.1          ------------------------------------------------------
------ 0
    EU548039.1          ------------------------------------------------------
------ 0
    AF207721.1          ------------------------------------------------------
------ 0
    AF207724.1          ------------------------------------------------------
------ 0
    JX982499.1          ------------------------------------------------------
------ 0
    EU548041.1          ------------------------------------------------------
------ 0
    EU548045.1          ------------------------------------------------------
------ 0
    AF207725.1          ------------------------------------------------------
------ 0
    EU548046.1          ------------------------------------------------------
------ 0
    AF207714.1          ------------------------------------------------------
------ 0
    AF207713.1          ------------------------------------------------------
------ 0
    AF207712.1          ------------------------------------------------------
------ 0
```

```
    AY750628.1       ------------------------------------------------------
------ 1044
    EF689084.1       ------------------------------------------------------
------ 0
    EF689085.1       ------------------------------------------------------
------ 0
    AB119070.1       ------------------------------------------------------
------ 956
    EF987742.1       ------------------------------------------------------
------ 0
    AB026105.1       ------------------------------------------------------
------ 0
    MW148603.1
AGTTAAAGATTGAGAGCATAAATCTCTCCTCAGTGATATGCCACAATTAGACACTTCAAC   7800
    AB051263.1       ------------------------------------------------------
------ 0
    AF068544.1       ------------------------------------------------------
------ 0


    JX982502.1       ------------------------------------------------------
------ 0
    JX982501.1       ------------------------------------------------------
------ 0
    JX982498.1       ------------------------------------------------------
------ 0
    JX982497.1       ------------------------------------------------------
------ 0
    JX982495.1       ------------------------------------------------------
------ 0
    JX982496.1       ------------------------------------------------------
------ 0
    JX982500.1       ------------------------------------------------------
------ 0
    EU548051.1       ------------------------------------------------------
------ 0
    EU548044.1       ------------------------------------------------------
------ 0
    EU548042.1       ------------------------------------------------------
------ 0
    EU548043.1       ------------------------------------------------------
------ 0
    EU548047.1       ------------------------------------------------------
------ 0
    EU548050.1       ------------------------------------------------------
------ 0
    EU548048.1       ------------------------------------------------------
------ 0
    EU548049.1       ------------------------------------------------------
------ 0
    AF207722.1       ------------------------------------------------------
------ 0
    EU548037.1       ------------------------------------------------------
------ 0
    AF207723.1       ------------------------------------------------------
------ 0
    EU548038.1       ------------------------------------------------------
------ 0
    EU548036.1       ------------------------------------------------------
------ 0
    EU548035.1       ------------------------------------------------------
------ 0
    AF207720.1       ------------------------------------------------------
------ 0
    AB601576.1       ------------------------------------------------------
------ 0
```

EU548040.1 --
------ 0
EU548039.1 --
------ 0
AF207721.1 --
------ 0
AF207724.1 --
------ 0
JX982499.1 --
------ 0
EU548041.1 --
------ 0
EU548045.1 --
------ 0
AF207725.1 --
------ 0
EU548046.1 --
------ 0
AF207714.1 --
------ 0
AF207713.1 --
------ 0
AF207712.1 --
------ 0
AY750628.1 --
------ 1044
EF689084.1 --
------ 0
EF689085.1 --
------ 0
AB119070.1 --
------ 956
EF987742.1 --
------ 0
AB026105.1 --
------ 0
MW148603.1
ATGATTAATCACTATTTTATCAATAATTGTAACCCTATTTTTTATATTTCAACTAAAACT 7860
AB051263.1 --
------ 0
AF068544.1 --
------ 0

JX982502.1 --
------ 0
JX982501.1 --
------ 0
JX982498.1 --
------ 0
JX982497.1 --
------ 0
JX982495.1 --
------ 0
JX982496.1 --
------ 0
JX982500.1 --
------ 0
EU548051.1 --
------ 0
EU548044.1 --
------ 0
EU548042.1 --
------ 0
EU548043.1 --
------ 0

```
   EU548047.1      ------------------------------------------------------
------ 0
   EU548050.1      ------------------------------------------------------
------ 0
   EU548048.1      ------------------------------------------------------
------ 0
   EU548049.1      ------------------------------------------------------
------ 0
   AF207722.1      ------------------------------------------------------
------ 0
   EU548037.1      ------------------------------------------------------
------ 0
   AF207723.1      ------------------------------------------------------
------ 0
   EU548038.1      ------------------------------------------------------
------ 0
   EU548036.1      ------------------------------------------------------
------ 0
   EU548035.1      ------------------------------------------------------
------ 0
   AF207720.1      ------------------------------------------------------
------ 0
   AB601576.1      ------------------------------------------------------
------ 0
   EU548040.1      ------------------------------------------------------
------ 0
   EU548039.1      ------------------------------------------------------
------ 0
   AF207721.1      ------------------------------------------------------
------ 0
   AF207724.1      ------------------------------------------------------
------ 0
   JX982499.1      ------------------------------------------------------
------ 0
   EU548041.1      ------------------------------------------------------
------ 0
   EU548045.1      ------------------------------------------------------
------ 0
   AF207725.1      ------------------------------------------------------
------ 0
   EU548046.1      ------------------------------------------------------
------ 0
   AF207714.1      ------------------------------------------------------
------ 0
   AF207713.1      ------------------------------------------------------
------ 0
   AF207712.1      ------------------------------------------------------
------ 0
   AY750628.1      ------------------------------------------------------
------ 1044
   EF689084.1      ------------------------------------------------------
------ 0
   EF689085.1      ------------------------------------------------------
------ 0
   AB119070.1      ------------------------------------------------------
------ 956
   EF987742.1      ------------------------------------------------------
------ 0
   AB026105.1      ------------------------------------------------------
------ 0
   MW148603.1
ATCAAAATACAACTTTCCAGAAAACCCTGAACCAAAATTAGTGGCTACATCAAAATCTAC   7920
   AB051263.1      ------------------------------------------------------
------ 0
   AF068544.1      ------------------------------------------------------
------ 0
```

```
    JX982502.1       ------------------------------------------------------
------ 0
    JX982501.1       ------------------------------------------------------
------ 0
    JX982498.1       ------------------------------------------------------
------ 0
    JX982497.1       ------------------------------------------------------
------ 0
    JX982495.1       ------------------------------------------------------
------ 0
    JX982496.1       ------------------------------------------------------
------ 0
    JX982500.1       ------------------------------------------------------
------ 0
    EU548051.1       ------------------------------------------------------
------ 0
    EU548044.1       ------------------------------------------------------
------ 0
    EU548042.1       ------------------------------------------------------
------ 0
    EU548043.1       ------------------------------------------------------
------ 0
    EU548047.1       ------------------------------------------------------
------ 0
    EU548050.1       ------------------------------------------------------
------ 0
    EU548048.1       ------------------------------------------------------
------ 0
    EU548049.1       ------------------------------------------------------
------ 0
    AF207722.1       ------------------------------------------------------
------ 0
    EU548037.1       ------------------------------------------------------
------ 0
    AF207723.1       ------------------------------------------------------
------ 0
    EU548038.1       ------------------------------------------------------
------ 0
    EU548036.1       ------------------------------------------------------
------ 0
    EU548035.1       ------------------------------------------------------
------ 0
    AF207720.1       ------------------------------------------------------
------ 0
    AB601576.1       ------------------------------------------------------
------ 0
    EU548040.1       ------------------------------------------------------
------ 0
    EU548039.1       ------------------------------------------------------
------ 0
    AF207721.1       ------------------------------------------------------
------ 0
    AF207724.1       ------------------------------------------------------
------ 0
    JX982499.1       ------------------------------------------------------
------ 0
    EU548041.1       ------------------------------------------------------
------ 0
    EU548045.1       ------------------------------------------------------
------ 0
    AF207725.1       ------------------------------------------------------
------ 0
    EU548046.1       ------------------------------------------------------
------ 0
```

```
    AF207714.1      ------------------------------------------------------
------ 0
    AF207713.1      ------------------------------------------------------
------ 0
    AF207712.1      ------------------------------------------------------
------ 0
    AY750628.1      ------------------------------------------------------
------ 1044
    EF689084.1      ------------------------------------------------------
------ 0
    EF689085.1      ------------------------------------------------------
------ 0
    AB119070.1      ------------------------------------------------------
------ 956
    EF987742.1      ------------------------------------------------------
------ 0
    AB026105.1      ------------------------------------------------------
------ 0
    MW148603.1
TACACCTTGAGAAAAGAAATGAACGAAAATCTATTTTCCTCATTCACTACCCCTACAATA    7980
    AB051263.1      ------------------------------------------------------
------ 0
    AF068544.1      ------------------------------------------------------
------ 0


    JX982502.1      ------------------------------------------------------
------ 0
    JX982501.1      ------------------------------------------------------
------ 0
    JX982498.1      ------------------------------------------------------
------ 0
    JX982497.1      ------------------------------------------------------
------ 0
    JX982495.1      ------------------------------------------------------
------ 0
    JX982496.1      ------------------------------------------------------
------ 0
    JX982500.1      ------------------------------------------------------
------ 0
    EU548051.1      ------------------------------------------------------
------ 0
    EU548044.1      ------------------------------------------------------
------ 0
    EU548042.1      ------------------------------------------------------
------ 0
    EU548043.1      ------------------------------------------------------
------ 0
    EU548047.1      ------------------------------------------------------
------ 0
    EU548050.1      ------------------------------------------------------
------ 0
    EU548048.1      ------------------------------------------------------
------ 0
    EU548049.1      ------------------------------------------------------
------ 0
    AF207722.1      ------------------------------------------------------
------ 0
    EU548037.1      ------------------------------------------------------
------ 0
    AF207723.1      ------------------------------------------------------
------ 0
    EU548038.1      ------------------------------------------------------
------ 0
    EU548036.1      ------------------------------------------------------
------ 0
```

EU548035.1 --
------ 0
AF207720.1 --
------ 0
AB601576.1 --
------ 0
EU548040.1 --
------ 0
EU548039.1 --
------ 0
AF207721.1 --
------ 0
AF207724.1 --
------ 0
JX982499.1 --
------ 0
EU548041.1 --
------ 0
EU548045.1 --
------ 0
AF207725.1 --
------ 0
EU548046.1 --
------ 0
AF207714.1 --
------ 0
AF207713.1 --
------ 0
AF207712.1 --
------ 0
AY750628.1 --
------ 1044
EF689084.1 --
------ 0
EF689085.1 --
------ 0
AB119070.1 --
------ 956
EF987742.1 --
------ 0
AB026105.1 --
------ 0
MW148603.1
ATAGGATTGCCTATCGTCATCCTCATCACCATATTCCCAGGTATTATATTCCCCTCACCC 8040
AB051263.1 --
------ 0
AF068544.1 --
------ 0

JX982502.1 --
------ 0
JX982501.1 --
------ 0
JX982498.1 --
------ 0
JX982497.1 --
------ 0
JX982495.1 --
------ 0
JX982496.1 --
------ 0
JX982500.1 --
------ 0
EU548051.1 --
------ 0

```
   EU548044.1        ------------------------------------------------------
------ 0
   EU548042.1        ------------------------------------------------------
------ 0
   EU548043.1        ------------------------------------------------------
------ 0
   EU548047.1        ------------------------------------------------------
------ 0
   EU548050.1        ------------------------------------------------------
------ 0
   EU548048.1        ------------------------------------------------------
------ 0
   EU548049.1        ------------------------------------------------------
------ 0
   AF207722.1        ------------------------------------------------------
------ 0
   EU548037.1        ------------------------------------------------------
------ 0
   AF207723.1        ------------------------------------------------------
------ 0
   EU548038.1        ------------------------------------------------------
------ 0
   EU548036.1        ------------------------------------------------------
------ 0
   EU548035.1        ------------------------------------------------------
------ 0
   AF207720.1        ------------------------------------------------------
------ 0
   AB601576.1        ------------------------------------------------------
------ 0
   EU548040.1        ------------------------------------------------------
------ 0
   EU548039.1        ------------------------------------------------------
------ 0
   AF207721.1        ------------------------------------------------------
------ 0
   AF207724.1        ------------------------------------------------------
------ 0
   JX982499.1        ------------------------------------------------------
------ 0
   EU548041.1        ------------------------------------------------------
------ 0
   EU548045.1        ------------------------------------------------------
------ 0
   AF207725.1        ------------------------------------------------------
------ 0
   EU548046.1        ------------------------------------------------------
------ 0
   AF207714.1        ------------------------------------------------------
------ 0
   AF207713.1        ------------------------------------------------------
------ 0
   AF207712.1        ------------------------------------------------------
------ 0
   AY750628.1        ------------------------------------------------------
------ 1044
   EF689084.1        ------------------------------------------------------
------ 0
   EF689085.1        ------------------------------------------------------
------ 0
   AB119070.1        ------------------------------------------------------
------ 956
   EF987742.1        ------------------------------------------------------
------ 0
   AB026105.1        ------------------------------------------------------
------ 0
```

MW148603.1
AACCGACTGATTAACAACCGACTCATTTCTATCCAACAATGATTGGTTCAATTAACATCA 8100
AB051263.1 --
------ 0
AF068544.1 --
------ 0

JX982502.1 --
------ 0
JX982501.1 --
------ 0
JX982498.1 --
------ 0
JX982497.1 --
------ 0
JX982495.1 --
------ 0
JX982496.1 --
------ 0
JX982500.1 --
------ 0
EU548051.1 --
------ 0
EU548044.1 --
------ 0
EU548042.1 --
------ 0
EU548043.1 --
------ 0
EU548047.1 --
------ 0
EU548050.1 --
------ 0
EU548048.1 --
------ 0
EU548049.1 --
------ 0
AF207722.1 --
------ 0
EU548037.1 --
------ 0
AF207723.1 --
------ 0
EU548038.1 --
------ 0
EU548036.1 --
------ 0
EU548035.1 --
------ 0
AF207720.1 --
------ 0
AB601576.1 --
------ 0
EU548040.1 --
------ 0
EU548039.1 --
------ 0
AF207721.1 --
------ 0
AF207724.1 --
------ 0
JX982499.1 --
------ 0
EU548041.1 --
------ 0

EU548045.1 --
------ 0
AF207725.1 --
------ 0
EU548046.1 --
------ 0
AF207714.1 --
------ 0
AF207713.1 --
------ 0
AF207712.1 --
------ 0
AY750628.1 --
------ 1044
EF689084.1 --
------ 0
EF689085.1 --
------ 0
AB119070.1 --
------ 956
EF987742.1 --
------ 0
AB026105.1 --
------ 0
MW148603.1
AAACAGATACTGTCCATTCACAACCAAAAAGGACAAACTTGGGCATTAATACTAATATCC 8160
AB051263.1 --
------ 0
AF068544.1 --
------ 0

JX982502.1 --
------ 0
JX982501.1 --
------ 0
JX982498.1 --
------ 0
JX982497.1 --
------ 0
JX982495.1 --
------ 0
JX982496.1 --
------ 0
JX982500.1 --
------ 0
EU548051.1 --
------ 0
EU548044.1 --
------ 0
EU548042.1 --
------ 0
EU548043.1 --
------ 0
EU548047.1 --
------ 0
EU548050.1 --
------ 0
EU548048.1 --
------ 0
EU548049.1 --
------ 0
AF207722.1 --
------ 0
EU548037.1 --
------ 0

```
   AF207723.1      ------------------------------------------------------
------ 0
   EU548038.1      ------------------------------------------------------
------ 0
   EU548036.1      ------------------------------------------------------
------ 0
   EU548035.1      ------------------------------------------------------
------ 0
   AF207720.1      ------------------------------------------------------
------ 0
   AB601576.1      ------------------------------------------------------
------ 0
   EU548040.1      ------------------------------------------------------
------ 0
   EU548039.1      ------------------------------------------------------
------ 0
   AF207721.1      ------------------------------------------------------
------ 0
   AF207724.1      ------------------------------------------------------
------ 0
   JX982499.1      ------------------------------------------------------
------ 0
   EU548041.1      ------------------------------------------------------
------ 0
   EU548045.1      ------------------------------------------------------
------ 0
   AF207725.1      ------------------------------------------------------
------ 0
   EU548046.1      ------------------------------------------------------
------ 0
   AF207714.1      ------------------------------------------------------
------ 0
   AF207713.1      ------------------------------------------------------
------ 0
   AF207712.1      ------------------------------------------------------
------ 0
   AY750628.1      ------------------------------------------------------
------ 1044
   EF689084.1      ------------------------------------------------------
------ 0
   EF689085.1      ------------------------------------------------------
------ 0
   AB119070.1      ------------------------------------------------------
------ 956
   EF987742.1      ------------------------------------------------------
------ 0
   AB026105.1      ------------------------------------------------------
------ 0
   MW148603.1
CTAATCCTATTTATTGGGTCTACTAACCTGCTAGGTCTCTTACCTCACTCATTTACCCCT   8220
   AB051263.1      ------------------------------------------------------
------ 0
   AF068544.1      ------------------------------------------------------
------ 0


   JX982502.1      ------------------------------------------------------
------ 0
   JX982501.1      ------------------------------------------------------
------ 0
   JX982498.1      ------------------------------------------------------
------ 0
   JX982497.1      ------------------------------------------------------
------ 0
   JX982495.1      ------------------------------------------------------
------ 0
```

```
   JX982496.1       ------------------------------------------------------
------ 0
   JX982500.1       ------------------------------------------------------
------ 0
   EU548051.1       ------------------------------------------------------
------ 0
   EU548044.1       ------------------------------------------------------
------ 0
   EU548042.1       ------------------------------------------------------
------ 0
   EU548043.1       ------------------------------------------------------
------ 0
   EU548047.1       ------------------------------------------------------
------ 0
   EU548050.1       ------------------------------------------------------
------ 0
   EU548048.1       ------------------------------------------------------
------ 0
   EU548049.1       ------------------------------------------------------
------ 0
   AF207722.1       ------------------------------------------------------
------ 0
   EU548037.1       ------------------------------------------------------
------ 0
   AF207723.1       ------------------------------------------------------
------ 0
   EU548038.1       ------------------------------------------------------
------ 0
   EU548036.1       ------------------------------------------------------
------ 0
   EU548035.1       ------------------------------------------------------
------ 0
   AF207720.1       ------------------------------------------------------
------ 0
   AB601576.1       ------------------------------------------------------
------ 0
   EU548040.1       ------------------------------------------------------
------ 0
   EU548039.1       ------------------------------------------------------
------ 0
   AF207721.1       ------------------------------------------------------
------ 0
   AF207724.1       ------------------------------------------------------
------ 0
   JX982499.1       ------------------------------------------------------
------ 0
   EU548041.1       ------------------------------------------------------
------ 0
   EU548045.1       ------------------------------------------------------
------ 0
   AF207725.1       ------------------------------------------------------
------ 0
   EU548046.1       ------------------------------------------------------
------ 0
   AF207714.1       ------------------------------------------------------
------ 0
   AF207713.1       ------------------------------------------------------
------ 0
   AF207712.1       ------------------------------------------------------
------ 0
   AY750628.1       ------------------------------------------------------
------ 1044
   EF689084.1       ------------------------------------------------------
------ 0
   EF689085.1       ------------------------------------------------------
------ 0
```

```
    AB119070.1      ------------------------------------------------------
------ 956
    EF987742.1      ------------------------------------------------------
------ 0
    AB026105.1      ------------------------------------------------------
------ 0
    MW148603.1
ACCACACAACTGTCCCTGAACCTAGGAATAGCTATCCCCCTATGAGCAGGCACAGTAATT   8280
    AB051263.1      ------------------------------------------------------
------ 0
    AF068544.1      ------------------------------------------------------
------ 0


    JX982502.1      ------------------------------------------------------
------ 0
    JX982501.1      ------------------------------------------------------
------ 0
    JX982498.1      ------------------------------------------------------
------ 0
    JX982497.1      ------------------------------------------------------
------ 0
    JX982495.1      ------------------------------------------------------
------ 0
    JX982496.1      ------------------------------------------------------
------ 0
    JX982500.1      ------------------------------------------------------
------ 0
    EU548051.1      ------------------------------------------------------
------ 0
    EU548044.1      ------------------------------------------------------
------ 0
    EU548042.1      ------------------------------------------------------
------ 0
    EU548043.1      ------------------------------------------------------
------ 0
    EU548047.1      ------------------------------------------------------
------ 0
    EU548050.1      ------------------------------------------------------
------ 0
    EU548048.1      ------------------------------------------------------
------ 0
    EU548049.1      ------------------------------------------------------
------ 0
    AF207722.1      ------------------------------------------------------
------ 0
    EU548037.1      ------------------------------------------------------
------ 0
    AF207723.1      ------------------------------------------------------
------ 0
    EU548038.1      ------------------------------------------------------
------ 0
    EU548036.1      ------------------------------------------------------
------ 0
    EU548035.1      ------------------------------------------------------
------ 0
    AF207720.1      ------------------------------------------------------
------ 0
    AB601576.1      ------------------------------------------------------
------ 0
    EU548040.1      ------------------------------------------------------
------ 0
    EU548039.1      ------------------------------------------------------
------ 0
    AF207721.1      ------------------------------------------------------
------ 0
```

```
    AF207724.1       ------------------------------------------------------
------ 0
    JX982499.1       ------------------------------------------------------
------ 0
    EU548041.1       ------------------------------------------------------
------ 0
    EU548045.1       ------------------------------------------------------
------ 0
    AF207725.1       ------------------------------------------------------
------ 0
    EU548046.1       ------------------------------------------------------
------ 0
    AF207714.1       ------------------------------------------------------
------ 0
    AF207713.1       ------------------------------------------------------
------ 0
    AF207712.1       ------------------------------------------------------
------ 0
    AY750628.1       ------------------------------------------------------
------ 1044
    EF689084.1       ------------------------------------------------------
------ 0
    EF689085.1       ------------------------------------------------------
------ 0
    AB119070.1       ------------------------------------------------------
------ 956
    EF987742.1       ------------------------------------------------------
------ 0
    AB026105.1       ------------------------------------------------------
------ 0
    MW148603.1
ACTGGATTTCGACACAAAACAAAAGCCTCTTTAGCCCACTTTCTACCACAAGGAACCCCA   8340
    AB051263.1       ------------------------------------------------------
------ 0
    AF068544.1       ------------------------------------------------------
------ 0


    JX982502.1       ------------------------------------------------------
------ 0
    JX982501.1       ------------------------------------------------------
------ 0
    JX982498.1       ------------------------------------------------------
------ 0
    JX982497.1       ------------------------------------------------------
------ 0
    JX982495.1       ------------------------------------------------------
------ 0
    JX982496.1       ------------------------------------------------------
------ 0
    JX982500.1       ------------------------------------------------------
------ 0
    EU548051.1       ------------------------------------------------------
------ 0
    EU548044.1       ------------------------------------------------------
------ 0
    EU548042.1       ------------------------------------------------------
------ 0
    EU548043.1       ------------------------------------------------------
------ 0
    EU548047.1       ------------------------------------------------------
------ 0
    EU548050.1       ------------------------------------------------------
------ 0
    EU548048.1       ------------------------------------------------------
------ 0
```

```
   EU548049.1       ------------------------------------------------------
------ 0
   AF207722.1       ------------------------------------------------------
------ 0
   EU548037.1       ------------------------------------------------------
------ 0
   AF207723.1       ------------------------------------------------------
------ 0
   EU548038.1       ------------------------------------------------------
------ 0
   EU548036.1       ------------------------------------------------------
------ 0
   EU548035.1       ------------------------------------------------------
------ 0
   AF207720.1       ------------------------------------------------------
------ 0
   AB601576.1       ------------------------------------------------------
------ 0
   EU548040.1       ------------------------------------------------------
------ 0
   EU548039.1       ------------------------------------------------------
------ 0
   AF207721.1       ------------------------------------------------------
------ 0
   AF207724.1       ------------------------------------------------------
------ 0
   JX982499.1       ------------------------------------------------------
------ 0
   EU548041.1       ------------------------------------------------------
------ 0
   EU548045.1       ------------------------------------------------------
------ 0
   AF207725.1       ------------------------------------------------------
------ 0
   EU548046.1       ------------------------------------------------------
------ 0
   AF207714.1       ------------------------------------------------------
------ 0
   AF207713.1       ------------------------------------------------------
------ 0
   AF207712.1       ------------------------------------------------------
------ 0
   AY750628.1       ------------------------------------------------------
------ 1044
   EF689084.1       ------------------------------------------------------
------ 0
   EF689085.1       ------------------------------------------------------
------ 0
   AB119070.1       ------------------------------------------------------
------ 956
   EF987742.1       ------------------------------------------------------
------ 0
   AB026105.1       ------------------------------------------------------
------ 0
   MW148603.1
CTACCCCTAATCCCCATGCTCATTATCATCGAATCTATCAGCCTATTTATTCAACCCATG   8400
   AB051263.1       ------------------------------------------------------
------ 0
   AF068544.1       ------------------------------------------------------
------ 0


   JX982502.1       ------------------------------------------------------
------ 0
   JX982501.1       ------------------------------------------------------
------ 0
```

```
    JX982498.1       ------------------------------------------------------
------ 0
    JX982497.1       ------------------------------------------------------
------ 0
    JX982495.1       ------------------------------------------------------
------ 0
    JX982496.1       ------------------------------------------------------
------ 0
    JX982500.1       ------------------------------------------------------
------ 0
    EU548051.1       ------------------------------------------------------
------ 0
    EU548044.1       ------------------------------------------------------
------ 0
    EU548042.1       ------------------------------------------------------
------ 0
    EU548043.1       ------------------------------------------------------
------ 0
    EU548047.1       ------------------------------------------------------
------ 0
    EU548050.1       ------------------------------------------------------
------ 0
    EU548048.1       ------------------------------------------------------
------ 0
    EU548049.1       ------------------------------------------------------
------ 0
    AF207722.1       ------------------------------------------------------
------ 0
    EU548037.1       ------------------------------------------------------
------ 0
    AF207723.1       ------------------------------------------------------
------ 0
    EU548038.1       ------------------------------------------------------
------ 0
    EU548036.1       ------------------------------------------------------
------ 0
    EU548035.1       ------------------------------------------------------
------ 0
    AF207720.1       ------------------------------------------------------
------ 0
    AB601576.1       ------------------------------------------------------
------ 0
    EU548040.1       ------------------------------------------------------
------ 0
    EU548039.1       ------------------------------------------------------
------ 0
    AF207721.1       ------------------------------------------------------
------ 0
    AF207724.1       ------------------------------------------------------
------ 0
    JX982499.1       ------------------------------------------------------
------ 0
    EU548041.1       ------------------------------------------------------
------ 0
    EU548045.1       ------------------------------------------------------
------ 0
    AF207725.1       ------------------------------------------------------
------ 0
    EU548046.1       ------------------------------------------------------
------ 0
    AF207714.1       ------------------------------------------------------
------ 0
    AF207713.1       ------------------------------------------------------
------ 0
    AF207712.1       ------------------------------------------------------
------ 0
```

```
    AY750628.1      ------------------------------------------------------
------ 1044
    EF689084.1      ------------------------------------------------------
------ 0
    EF689085.1      ------------------------------------------------------
------ 0
    AB119070.1      ------------------------------------------------------
------ 956
    EF987742.1      ------------------------------------------------------
------ 0
    AB026105.1      ------------------------------------------------------
------ 0
    MW148603.1
GCCCTGGCCGTGCGACTAACAGCTAACATCACAGCGGGCCACCTATTAATTCACTTAATT   8460
    AB051263.1      ------------------------------------------------------
------ 0
    AF068544.1      ------------------------------------------------------
------ 0


    JX982502.1      ------------------------------------------------------
------ 0
    JX982501.1      ------------------------------------------------------
------ 0
    JX982498.1      ------------------------------------------------------
------ 0
    JX982497.1      ------------------------------------------------------
------ 0
    JX982495.1      ------------------------------------------------------
------ 0
    JX982496.1      ------------------------------------------------------
------ 0
    JX982500.1      ------------------------------------------------------
------ 0
    EU548051.1      ------------------------------------------------------
------ 0
    EU548044.1      ------------------------------------------------------
------ 0
    EU548042.1      ------------------------------------------------------
------ 0
    EU548043.1      ------------------------------------------------------
------ 0
    EU548047.1      ------------------------------------------------------
------ 0
    EU548050.1      ------------------------------------------------------
------ 0
    EU548048.1      ------------------------------------------------------
------ 0
    EU548049.1      ------------------------------------------------------
------ 0
    AF207722.1      ------------------------------------------------------
------ 0
    EU548037.1      ------------------------------------------------------
------ 0
    AF207723.1      ------------------------------------------------------
------ 0
    EU548038.1      ------------------------------------------------------
------ 0
    EU548036.1      ------------------------------------------------------
------ 0
    EU548035.1      ------------------------------------------------------
------ 0
    AF207720.1      ------------------------------------------------------
------ 0
    AB601576.1      ------------------------------------------------------
------ 0
```

```
   EU548040.1      ------------------------------------------------------
------ 0
   EU548039.1      ------------------------------------------------------
------ 0
   AF207721.1      ------------------------------------------------------
------ 0
   AF207724.1      ------------------------------------------------------
------ 0
   JX982499.1      ------------------------------------------------------
------ 0
   EU548041.1      ------------------------------------------------------
------ 0
   EU548045.1      ------------------------------------------------------
------ 0
   AF207725.1      ------------------------------------------------------
------ 0
   EU548046.1      ------------------------------------------------------
------ 0
   AF207714.1      ------------------------------------------------------
------ 0
   AF207713.1      ------------------------------------------------------
------ 0
   AF207712.1      ------------------------------------------------------
------ 0
   AY750628.1      ------------------------------------------------------
------ 1044
   EF689084.1      ------------------------------------------------------
------ 0
   EF689085.1      ------------------------------------------------------
------ 0
   AB119070.1      ------------------------------------------------------
------ 956
   EF987742.1      ------------------------------------------------------
------ 0
   AB026105.1      ------------------------------------------------------
------ 0
   MW148603.1
GGAGGAGCCACCCTAGCCCTAATAAACATTAGTACTGTTACAGCAATAATTACCTTTTCC   8520
   AB051263.1      ------------------------------------------------------
------ 0
   AF068544.1      ------------------------------------------------------
------ 0


   JX982502.1      ------------------------------------------------------
------ 0
   JX982501.1      ------------------------------------------------------
------ 0
   JX982498.1      ------------------------------------------------------
------ 0
   JX982497.1      ------------------------------------------------------
------ 0
   JX982495.1      ------------------------------------------------------
------ 0
   JX982496.1      ------------------------------------------------------
------ 0
   JX982500.1      ------------------------------------------------------
------ 0
   EU548051.1      ------------------------------------------------------
------ 0
   EU548044.1      ------------------------------------------------------
------ 0
   EU548042.1      ------------------------------------------------------
------ 0
   EU548043.1      ------------------------------------------------------
------ 0
```

EU548047.1 --
------ 0
EU548050.1 --
------ 0
EU548048.1 --
------ 0
EU548049.1 --
------ 0
AF207722.1 --
------ 0
EU548037.1 --
------ 0
AF207723.1 --
------ 0
EU548038.1 --
------ 0
EU548036.1 --
------ 0
EU548035.1 --
------ 0
AF207720.1 --
------ 0
AB601576.1 --
------ 0
EU548040.1 --
------ 0
EU548039.1 --
------ 0
AF207721.1 --
------ 0
AF207724.1 --
------ 0
JX982499.1 --
------ 0
EU548041.1 --
------ 0
EU548045.1 --
------ 0
AF207725.1 --
------ 0
EU548046.1 --
------ 0
AF207714.1 --
------ 0
AF207713.1 --
------ 0
AF207712.1 --
------ 0
AY750628.1 --
------ 1044
EF689084.1 --
------ 0
EF689085.1 --
------ 0
AB119070.1 --
------ 956
EF987742.1 --
------ 0
AB026105.1 --
------ 0
MW148603.1
ATCCTTGTCTTATTAACTATCTTAGAATTTGCAGTAGCCCTTATTCAAGCTTACGTCTTT 8580
AB051263.1 --
------ 0
AF068544.1 --
------ 0

JX982502.1 --
------ 0
JX982501.1 --
------ 0
JX982498.1 --
------ 0
JX982497.1 --
------ 0
JX982495.1 --
------ 0
JX982496.1 --
------ 0
JX982500.1 --
------ 0
EU548051.1 --
------ 0
EU548044.1 --
------ 0
EU548042.1 --
------ 0
EU548043.1 --
------ 0
EU548047.1 --
------ 0
EU548050.1 --
------ 0
EU548048.1 --
------ 0
EU548049.1 --
------ 0
AF207722.1 --
------ 0
EU548037.1 --
------ 0
AF207723.1 --
------ 0
EU548038.1 --
------ 0
EU548036.1 --
------ 0
EU548035.1 --
------ 0
AF207720.1 --
------ 0
AB601576.1 --
------ 0
EU548040.1 --
------ 0
EU548039.1 --
------ 0
AF207721.1 --
------ 0
AF207724.1 --
------ 0
JX982499.1 --
------ 0
EU548041.1 --
------ 0
EU548045.1 --
------ 0
AF207725.1 --
------ 0
EU548046.1 --
------ 0

AF207714.1 --
------ 0
AF207713.1 --
------ 0
AF207712.1 --
------ 0
AY750628.1 --
------ 1044
EF689084.1 --
------ 0
EF689085.1 --
------ 0
AB119070.1 --
------ 956
EF987742.1 --
------ 0
AB026105.1 --
------ 0
MW148603.1
ACCCTACTAGTAAGCCTATATTTACATGACAACACCTAATGACCCACCAAACACACTCAT 8640
AB051263.1 --
------ 0
AF068544.1 --
------ 0

JX982502.1 --
------ 0
JX982501.1 --
------ 0
JX982498.1 --
------ 0
JX982497.1 --
------ 0
JX982495.1 --
------ 0
JX982496.1 --
------ 0
JX982500.1 --
------ 0
EU548051.1 --
------ 0
EU548044.1 --
------ 0
EU548042.1 --
------ 0
EU548043.1 --
------ 0
EU548047.1 --
------ 0
EU548050.1 --
------ 0
EU548048.1 --
------ 0
EU548049.1 --
------ 0
AF207722.1 --
------ 0
EU548037.1 --
------ 0
AF207723.1 --
------ 0
EU548038.1 --
------ 0
EU548036.1 --
------ 0

EU548035.1 --
------ 0
AF207720.1 --
------ 0
AB601576.1 --
------ 0
EU548040.1 --
------ 0
EU548039.1 --
------ 0
AF207721.1 --
------ 0
AF207724.1 --
------ 0
JX982499.1 --
------ 0
EU548041.1 --
------ 0
EU548045.1 --
------ 0
AF207725.1 --
------ 0
EU548046.1 --
------ 0
AF207714.1 --
------ 0
AF207713.1 --
------ 0
AF207712.1 --
------ 0
AY750628.1 --
------ 1044
EF689084.1 --
------ 0
EF689085.1 --
------ 0
AB119070.1 --
------ 956
EF987742.1 --
------ 0
AB026105.1 --
------ 0
MW148603.1
ATCACATAGTCAATCCAAGCCCATGACCCCTGACAGGAGCTCTTTCCGCCCTACTTACAA 8700
AB051263.1 --
------ 0
AF068544.1 --
------ 0

JX982502.1 --
------ 0
JX982501.1 --
------ 0
JX982498.1 --
------ 0
JX982497.1 --
------ 0
JX982495.1 --
------ 0
JX982496.1 --
------ 0
JX982500.1 --
------ 0
EU548051.1 --
------ 0

```
    EU548044.1        ------------------------------------------------------
------ 0
    EU548042.1        ------------------------------------------------------
------ 0
    EU548043.1        ------------------------------------------------------
------ 0
    EU548047.1        ------------------------------------------------------
------ 0
    EU548050.1        ------------------------------------------------------
------ 0
    EU548048.1        ------------------------------------------------------
------ 0
    EU548049.1        ------------------------------------------------------
------ 0
    AF207722.1        ------------------------------------------------------
------ 0
    EU548037.1        ------------------------------------------------------
------ 0
    AF207723.1        ------------------------------------------------------
------ 0
    EU548038.1        ------------------------------------------------------
------ 0
    EU548036.1        ------------------------------------------------------
------ 0
    EU548035.1        ------------------------------------------------------
------ 0
    AF207720.1        ------------------------------------------------------
------ 0
    AB601576.1        ------------------------------------------------------
------ 0
    EU548040.1        ------------------------------------------------------
------ 0
    EU548039.1        ------------------------------------------------------
------ 0
    AF207721.1        ------------------------------------------------------
------ 0
    AF207724.1        ------------------------------------------------------
------ 0
    JX982499.1        ------------------------------------------------------
------ 0
    EU548041.1        ------------------------------------------------------
------ 0
    EU548045.1        ------------------------------------------------------
------ 0
    AF207725.1        ------------------------------------------------------
------ 0
    EU548046.1        ------------------------------------------------------
------ 0
    AF207714.1        ------------------------------------------------------
------ 0
    AF207713.1        ------------------------------------------------------
------ 0
    AF207712.1        ------------------------------------------------------
------ 0
    AY750628.1        ------------------------------------------------------
------ 1044
    EF689084.1        ------------------------------------------------------
------ 0
    EF689085.1        ------------------------------------------------------
------ 0
    AB119070.1        ------------------------------------------------------
------ 956
    EF987742.1        ------------------------------------------------------
------ 0
    AB026105.1        ------------------------------------------------------
------ 0
```

```
    MW148603.1
CATCAGGACTAGCAATATGATTCCACTACAATTCATTGTCTCTTCTAACCCTAGGAACTA    8760
    AB051263.1        ------------------------------------------------------
------ 0
    AF068544.1        ------------------------------------------------------
------ 0


    JX982502.1        ------------------------------------------------------
------ 0
    JX982501.1        ------------------------------------------------------
------ 0
    JX982498.1        ------------------------------------------------------
------ 0
    JX982497.1        ------------------------------------------------------
------ 0
    JX982495.1        ------------------------------------------------------
------ 0
    JX982496.1        ------------------------------------------------------
------ 0
    JX982500.1        ------------------------------------------------------
------ 0
    EU548051.1        ------------------------------------------------------
------ 0
    EU548044.1        ------------------------------------------------------
------ 0
    EU548042.1        ------------------------------------------------------
------ 0
    EU548043.1        ------------------------------------------------------
------ 0
    EU548047.1        ------------------------------------------------------
------ 0
    EU548050.1        ------------------------------------------------------
------ 0
    EU548048.1        ------------------------------------------------------
------ 0
    EU548049.1        ------------------------------------------------------
------ 0
    AF207722.1        ------------------------------------------------------
------ 0
    EU548037.1        ------------------------------------------------------
------ 0
    AF207723.1        ------------------------------------------------------
------ 0
    EU548038.1        ------------------------------------------------------
------ 0
    EU548036.1        ------------------------------------------------------
------ 0
    EU548035.1        ------------------------------------------------------
------ 0
    AF207720.1        ------------------------------------------------------
------ 0
    AB601576.1        ------------------------------------------------------
------ 0
    EU548040.1        ------------------------------------------------------
------ 0
    EU548039.1        ------------------------------------------------------
------ 0
    AF207721.1        ------------------------------------------------------
------ 0
    AF207724.1        ------------------------------------------------------
------ 0
    JX982499.1        ------------------------------------------------------
------ 0
    EU548041.1        ------------------------------------------------------
------ 0
```

```
    EU548045.1        ------------------------------------------------------
------ 0
    AF207725.1        ------------------------------------------------------
------ 0
    EU548046.1        ------------------------------------------------------
------ 0
    AF207714.1        ------------------------------------------------------
------ 0
    AF207713.1        ------------------------------------------------------
------ 0
    AF207712.1        ------------------------------------------------------
------ 0
    AY750628.1        ------------------------------------------------------
------ 1044
    EF689084.1        ------------------------------------------------------
------ 0
    EF689085.1        ------------------------------------------------------
------ 0
    AB119070.1        ------------------------------------------------------
------ 956
    EF987742.1        ------------------------------------------------------
------ 0
    AB026105.1        ------------------------------------------------------
------ 0
    MW148603.1
CAGCTAATGTACTAACCATATATCAATGGTGACGAGATGTGGTCCGAGAAGGAACATTTC    8820
    AB051263.1        ------------------------------------------------------
------ 0
    AF068544.1        ------------------------------------------------------
------ 0


    JX982502.1        ------------------------------------------------------
------ 0
    JX982501.1        ------------------------------------------------------
------ 0
    JX982498.1        ------------------------------------------------------
------ 0
    JX982497.1        ------------------------------------------------------
------ 0
    JX982495.1        ------------------------------------------------------
------ 0
    JX982496.1        ------------------------------------------------------
------ 0
    JX982500.1        ------------------------------------------------------
------ 0
    EU548051.1        ------------------------------------------------------
------ 0
    EU548044.1        ------------------------------------------------------
------ 0
    EU548042.1        ------------------------------------------------------
------ 0
    EU548043.1        ------------------------------------------------------
------ 0
    EU548047.1        ------------------------------------------------------
------ 0
    EU548050.1        ------------------------------------------------------
------ 0
    EU548048.1        ------------------------------------------------------
------ 0
    EU548049.1        ------------------------------------------------------
------ 0
    AF207722.1        ------------------------------------------------------
------ 0
    EU548037.1        ------------------------------------------------------
------ 0
```

```
    AF207723.1        ------------------------------------------------------
------ 0
    EU548038.1        ------------------------------------------------------
------ 0
    EU548036.1        ------------------------------------------------------
------ 0
    EU548035.1        ------------------------------------------------------
------ 0
    AF207720.1        ------------------------------------------------------
------ 0
    AB601576.1        ------------------------------------------------------
------ 0
    EU548040.1        ------------------------------------------------------
------ 0
    EU548039.1        ------------------------------------------------------
------ 0
    AF207721.1        ------------------------------------------------------
------ 0
    AF207724.1        ------------------------------------------------------
------ 0
    JX982499.1        ------------------------------------------------------
------ 0
    EU548041.1        ------------------------------------------------------
------ 0
    EU548045.1        ------------------------------------------------------
------ 0
    AF207725.1        ------------------------------------------------------
------ 0
    EU548046.1        ------------------------------------------------------
------ 0
    AF207714.1        ------------------------------------------------------
------ 0
    AF207713.1        ------------------------------------------------------
------ 0
    AF207712.1        ------------------------------------------------------
------ 0
    AY750628.1        ------------------------------------------------------
------ 1044
    EF689084.1        ------------------------------------------------------
------ 0
    EF689085.1        ------------------------------------------------------
------ 0
    AB119070.1        ------------------------------------------------------
------ 956
    EF987742.1        ------------------------------------------------------
------ 0
    AB026105.1        ------------------------------------------------------
------ 0
    MW148603.1
AAGGCCACCATACCCCCACTGTTCAAAAAGGTTTACGATACGGAATAATCCTCTTCATCA   8880
    AB051263.1        ------------------------------------------------------
------ 0
    AF068544.1        ------------------------------------------------------
------ 0


    JX982502.1        ------------------------------------------------------
------ 0
    JX982501.1        ------------------------------------------------------
------ 0
    JX982498.1        ------------------------------------------------------
------ 0
    JX982497.1        ------------------------------------------------------
------ 0
    JX982495.1        ------------------------------------------------------
------ 0
```

```
    JX982496.1      ------------------------------------------------------
------ 0
    JX982500.1      ------------------------------------------------------
------ 0
    EU548051.1      ------------------------------------------------------
------ 0
    EU548044.1      ------------------------------------------------------
------ 0
    EU548042.1      ------------------------------------------------------
------ 0
    EU548043.1      ------------------------------------------------------
------ 0
    EU548047.1      ------------------------------------------------------
------ 0
    EU548050.1      ------------------------------------------------------
------ 0
    EU548048.1      ------------------------------------------------------
------ 0
    EU548049.1      ------------------------------------------------------
------ 0
    AF207722.1      ------------------------------------------------------
------ 0
    EU548037.1      ------------------------------------------------------
------ 0
    AF207723.1      ------------------------------------------------------
------ 0
    EU548038.1      ------------------------------------------------------
------ 0
    EU548036.1      ------------------------------------------------------
------ 0
    EU548035.1      ------------------------------------------------------
------ 0
    AF207720.1      ------------------------------------------------------
------ 0
    AB601576.1      ------------------------------------------------------
------ 0
    EU548040.1      ------------------------------------------------------
------ 0
    EU548039.1      ------------------------------------------------------
------ 0
    AF207721.1      ------------------------------------------------------
------ 0
    AF207724.1      ------------------------------------------------------
------ 0
    JX982499.1      ------------------------------------------------------
------ 0
    EU548041.1      ------------------------------------------------------
------ 0
    EU548045.1      ------------------------------------------------------
------ 0
    AF207725.1      ------------------------------------------------------
------ 0
    EU548046.1      ------------------------------------------------------
------ 0
    AF207714.1      ------------------------------------------------------
------ 0
    AF207713.1      ------------------------------------------------------
------ 0
    AF207712.1      ------------------------------------------------------
------ 0
    AY750628.1      ------------------------------------------------------
------ 1044
    EF689084.1      ------------------------------------------------------
------ 0
    EF689085.1      ------------------------------------------------------
------ 0
```

```
    AB119070.1       ------------------------------------------------------
------ 956
    EF987742.1       ------------------------------------------------------
------ 0
    AB026105.1       ------------------------------------------------------
------ 0
    MW148603.1
CATCCGAAGTCTTTTTCTTTGCGGGCTTCTTCTGGCTTTTTACCATTCAAGCCTAGCCC   8940
    AB051263.1       ------------------------------------------------------
------ 0
    AF068544.1       ------------------------------------------------------
------ 0


    JX982502.1       ------------------------------------------------------
------ 0
    JX982501.1       ------------------------------------------------------
------ 0
    JX982498.1       ------------------------------------------------------
------ 0
    JX982497.1       ------------------------------------------------------
------ 0
    JX982495.1       ------------------------------------------------------
------ 0
    JX982496.1       ------------------------------------------------------
------ 0
    JX982500.1       ------------------------------------------------------
------ 0
    EU548051.1       ------------------------------------------------------
------ 0
    EU548044.1       ------------------------------------------------------
------ 0
    EU548042.1       ------------------------------------------------------
------ 0
    EU548043.1       ------------------------------------------------------
------ 0
    EU548047.1       ------------------------------------------------------
------ 0
    EU548050.1       ------------------------------------------------------
------ 0
    EU548048.1       ------------------------------------------------------
------ 0
    EU548049.1       ------------------------------------------------------
------ 0
    AF207722.1       ------------------------------------------------------
------ 0
    EU548037.1       ------------------------------------------------------
------ 0
    AF207723.1       ------------------------------------------------------
------ 0
    EU548038.1       ------------------------------------------------------
------ 0
    EU548036.1       ------------------------------------------------------
------ 0
    EU548035.1       ------------------------------------------------------
------ 0
    AF207720.1       ------------------------------------------------------
------ 0
    AB601576.1       ------------------------------------------------------
------ 0
    EU548040.1       ------------------------------------------------------
------ 0
    EU548039.1       ------------------------------------------------------
------ 0
    AF207721.1       ------------------------------------------------------
------ 0
```

```
   AF207724.1      ------------------------------------------------------
------ 0
   JX982499.1      ------------------------------------------------------
------ 0
   EU548041.1      ------------------------------------------------------
------ 0
   EU548045.1      ------------------------------------------------------
------ 0
   AF207725.1      ------------------------------------------------------
------ 0
   EU548046.1      ------------------------------------------------------
------ 0
   AF207714.1      ------------------------------------------------------
------ 0
   AF207713.1      ------------------------------------------------------
------ 0
   AF207712.1      ------------------------------------------------------
------ 0
   AY750628.1      ------------------------------------------------------
------ 1044
   EF689084.1      ------------------------------------------------------
------ 0
   EF689085.1      ------------------------------------------------------
------ 0
   AB119070.1      ------------------------------------------------------
------ 956
   EF987742.1      ------------------------------------------------------
------ 0
   AB026105.1      ------------------------------------------------------
------ 0
   MW148603.1
CAACACCCGAACTTGGAGGGTGCTGACCACCTACAGGTATTACACCCCTAAACCCCTTAG   9000
   AB051263.1      ------------------------------------------------------
------ 0
   AF068544.1      ------------------------------------------------------
------ 0


   JX982502.1      ------------------------------------------------------
------ 0
   JX982501.1      ------------------------------------------------------
------ 0
   JX982498.1      ------------------------------------------------------
------ 0
   JX982497.1      ------------------------------------------------------
------ 0
   JX982495.1      ------------------------------------------------------
------ 0
   JX982496.1      ------------------------------------------------------
------ 0
   JX982500.1      ------------------------------------------------------
------ 0
   EU548051.1      ------------------------------------------------------
------ 0
   EU548044.1      ------------------------------------------------------
------ 0
   EU548042.1      ------------------------------------------------------
------ 0
   EU548043.1      ------------------------------------------------------
------ 0
   EU548047.1      ------------------------------------------------------
------ 0
   EU548050.1      ------------------------------------------------------
------ 0
   EU548048.1      ------------------------------------------------------
------ 0
```

EU548049.1 --
------ 0
AF207722.1 --
------ 0
EU548037.1 --
------ 0
AF207723.1 --
------ 0
EU548038.1 --
------ 0
EU548036.1 --
------ 0
EU548035.1 --
------ 0
AF207720.1 --
------ 0
AB601576.1 --
------ 0
EU548040.1 --
------ 0
EU548039.1 --
------ 0
AF207721.1 --
------ 0
AF207724.1 --
------ 0
JX982499.1 --
------ 0
EU548041.1 --
------ 0
EU548045.1 --
------ 0
AF207725.1 --
------ 0
EU548046.1 --
------ 0
AF207714.1 --
------ 0
AF207713.1 --
------ 0
AF207712.1 --
------ 0
AY750628.1 --
------ 1044
EF689084.1 --
------ 0
EF689085.1 --
------ 0
AB119070.1 --
------ 956
EF987742.1 --
------ 0
AB026105.1 --
------ 0
MW148603.1
AAGTACCATTACTAAATACCTCTGTCCTCCTAGCCTCCGGAGTCTCTATTACTTGGGCCC 9060
AB051263.1 --
------ 0
AF068544.1 --
------ 0

JX982502.1 --
------ 0
JX982501.1 --
------ 0

```
    JX982498.1          ------------------------------------------------------
------ 0
    JX982497.1          ------------------------------------------------------
------ 0
    JX982495.1          ------------------------------------------------------
------ 0
    JX982496.1          ------------------------------------------------------
------ 0
    JX982500.1          ------------------------------------------------------
------ 0
    EU548051.1          ------------------------------------------------------
------ 0
    EU548044.1          ------------------------------------------------------
------ 0
    EU548042.1          ------------------------------------------------------
------ 0
    EU548043.1          ------------------------------------------------------
------ 0
    EU548047.1          ------------------------------------------------------
------ 0
    EU548050.1          ------------------------------------------------------
------ 0
    EU548048.1          ------------------------------------------------------
------ 0
    EU548049.1          ------------------------------------------------------
------ 0
    AF207722.1          ------------------------------------------------------
------ 0
    EU548037.1          ------------------------------------------------------
------ 0
    AF207723.1          ------------------------------------------------------
------ 0
    EU548038.1          ------------------------------------------------------
------ 0
    EU548036.1          ------------------------------------------------------
------ 0
    EU548035.1          ------------------------------------------------------
------ 0
    AF207720.1          ------------------------------------------------------
------ 0
    AB601576.1          ------------------------------------------------------
------ 0
    EU548040.1          ------------------------------------------------------
------ 0
    EU548039.1          ------------------------------------------------------
------ 0
    AF207721.1          ------------------------------------------------------
------ 0
    AF207724.1          ------------------------------------------------------
------ 0
    JX982499.1          ------------------------------------------------------
------ 0
    EU548041.1          ------------------------------------------------------
------ 0
    EU548045.1          ------------------------------------------------------
------ 0
    AF207725.1          ------------------------------------------------------
------ 0
    EU548046.1          ------------------------------------------------------
------ 0
    AF207714.1          ------------------------------------------------------
------ 0
    AF207713.1          ------------------------------------------------------
------ 0
    AF207712.1          ------------------------------------------------------
------ 0
```

```
    AY750628.1       ------------------------------------------------------
------ 1044
    EF689084.1       ------------------------------------------------------
------ 0
    EF689085.1       ------------------------------------------------------
------ 0
    AB119070.1       ------------------------------------------------------
------ 956
    EF987742.1       ------------------------------------------------------
------ 0
    AB026105.1       ------------------------------------------------------
------ 0
    MW148603.1
ACCACAGCCTCATAGAAGGGGACCGCAAACACATACTCCAAGCCCTATTTATCACAATCT   9120
    AB051263.1       ------------------------------------------------------
------ 0
    AF068544.1       ------------------------------------------------------
------ 0


    JX982502.1       ------------------------------------------------------
------ 0
    JX982501.1       ------------------------------------------------------
------ 0
    JX982498.1       ------------------------------------------------------
------ 0
    JX982497.1       ------------------------------------------------------
------ 0
    JX982495.1       ------------------------------------------------------
------ 0
    JX982496.1       ------------------------------------------------------
------ 0
    JX982500.1       ------------------------------------------------------
------ 0
    EU548051.1       ------------------------------------------------------
------ 0
    EU548044.1       ------------------------------------------------------
------ 0
    EU548042.1       ------------------------------------------------------
------ 0
    EU548043.1       ------------------------------------------------------
------ 0
    EU548047.1       ------------------------------------------------------
------ 0
    EU548050.1       ------------------------------------------------------
------ 0
    EU548048.1       ------------------------------------------------------
------ 0
    EU548049.1       ------------------------------------------------------
------ 0
    AF207722.1       ------------------------------------------------------
------ 0
    EU548037.1       ------------------------------------------------------
------ 0
    AF207723.1       ------------------------------------------------------
------ 0
    EU548038.1       ------------------------------------------------------
------ 0
    EU548036.1       ------------------------------------------------------
------ 0
    EU548035.1       ------------------------------------------------------
------ 0
    AF207720.1       ------------------------------------------------------
------ 0
    AB601576.1       ------------------------------------------------------
------ 0
```

```
   EU548040.1      ------------------------------------------------------
------ 0
   EU548039.1      ------------------------------------------------------
------ 0
   AF207721.1      ------------------------------------------------------
------ 0
   AF207724.1      ------------------------------------------------------
------ 0
   JX982499.1      ------------------------------------------------------
------ 0
   EU548041.1      ------------------------------------------------------
------ 0
   EU548045.1      ------------------------------------------------------
------ 0
   AF207725.1      ------------------------------------------------------
------ 0
   EU548046.1      ------------------------------------------------------
------ 0
   AF207714.1      ------------------------------------------------------
------ 0
   AF207713.1      ------------------------------------------------------
------ 0
   AF207712.1      ------------------------------------------------------
------ 0
   AY750628.1      ------------------------------------------------------
------ 1044
   EF689084.1      ------------------------------------------------------
------ 0
   EF689085.1      ------------------------------------------------------
------ 0
   AB119070.1      ------------------------------------------------------
------ 956
   EF987742.1      ------------------------------------------------------
------ 0
   AB026105.1      ------------------------------------------------------
------ 0
   MW148603.1
CCCTAGGCCTGTATTTTACTGTCCTACAAGCCTCCGAATACTACGAAGCTCCATTCACAA   9180
   AB051263.1      ------------------------------------------------------
------ 0
   AF068544.1      ------------------------------------------------------
------ 0


   JX982502.1      ------------------------------------------------------
------ 0
   JX982501.1      ------------------------------------------------------
------ 0
   JX982498.1      ------------------------------------------------------
------ 0
   JX982497.1      ------------------------------------------------------
------ 0
   JX982495.1      ------------------------------------------------------
------ 0
   JX982496.1      ------------------------------------------------------
------ 0
   JX982500.1      ------------------------------------------------------
------ 0
   EU548051.1      ------------------------------------------------------
------ 0
   EU548044.1      ------------------------------------------------------
------ 0
   EU548042.1      ------------------------------------------------------
------ 0
   EU548043.1      ------------------------------------------------------
------ 0
```

EU548047.1 --
------ 0
EU548050.1 --
------ 0
EU548048.1 --
------ 0
EU548049.1 --
------ 0
AF207722.1 --
------ 0
EU548037.1 --
------ 0
AF207723.1 --
------ 0
EU548038.1 --
------ 0
EU548036.1 --
------ 0
EU548035.1 --
------ 0
AF207720.1 --
------ 0
AB601576.1 --
------ 0
EU548040.1 --
------ 0
EU548039.1 --
------ 0
AF207721.1 --
------ 0
AF207724.1 --
------ 0
JX982499.1 --
------ 0
EU548041.1 --
------ 0
EU548045.1 --
------ 0
AF207725.1 --
------ 0
EU548046.1 --
------ 0
AF207714.1 --
------ 0
AF207713.1 --
------ 0
AF207712.1 --
------ 0
AY750628.1 --
------ 1044
EF689084.1 --
------ 0
EF689085.1 --
------ 0
AB119070.1 --
------ 956
EF987742.1 --
------ 0
AB026105.1 --
------ 0
MW148603.1
TCTCGGACGGAGTCTACGGCTCTACATTTTTTATAGCCACAGGATTCCACGGCCTCCATG 9240
AB051263.1 --
------ 0
AF068544.1 --
------ 0

```
    JX982502.1        ------------------------------------------------------
------ 0
    JX982501.1        ------------------------------------------------------
------ 0
    JX982498.1        ------------------------------------------------------
------ 0
    JX982497.1        ------------------------------------------------------
------ 0
    JX982495.1        ------------------------------------------------------
------ 0
    JX982496.1        ------------------------------------------------------
------ 0
    JX982500.1        ------------------------------------------------------
------ 0
    EU548051.1        ------------------------------------------------------
------ 0
    EU548044.1        ------------------------------------------------------
------ 0
    EU548042.1        ------------------------------------------------------
------ 0
    EU548043.1        ------------------------------------------------------
------ 0
    EU548047.1        ------------------------------------------------------
------ 0
    EU548050.1        ------------------------------------------------------
------ 0
    EU548048.1        ------------------------------------------------------
------ 0
    EU548049.1        ------------------------------------------------------
------ 0
    AF207722.1        ------------------------------------------------------
------ 0
    EU548037.1        ------------------------------------------------------
------ 0
    AF207723.1        ------------------------------------------------------
------ 0
    EU548038.1        ------------------------------------------------------
------ 0
    EU548036.1        ------------------------------------------------------
------ 0
    EU548035.1        ------------------------------------------------------
------ 0
    AF207720.1        ------------------------------------------------------
------ 0
    AB601576.1        ------------------------------------------------------
------ 0
    EU548040.1        ------------------------------------------------------
------ 0
    EU548039.1        ------------------------------------------------------
------ 0
    AF207721.1        ------------------------------------------------------
------ 0
    AF207724.1        ------------------------------------------------------
------ 0
    JX982499.1        ------------------------------------------------------
------ 0
    EU548041.1        ------------------------------------------------------
------ 0
    EU548045.1        ------------------------------------------------------
------ 0
    AF207725.1        ------------------------------------------------------
------ 0
    EU548046.1        ------------------------------------------------------
------ 0
```

```
    AF207714.1       ------------------------------------------------------
------ 0
    AF207713.1       ------------------------------------------------------
------ 0
    AF207712.1       ------------------------------------------------------
------ 0
    AY750628.1       ------------------------------------------------------
------ 1044
    EF689084.1       ------------------------------------------------------
------ 0
    EF689085.1       ------------------------------------------------------
------ 0
    AB119070.1       ------------------------------------------------------
------ 956
    EF987742.1       ------------------------------------------------------
------ 0
    AB026105.1       ------------------------------------------------------
------ 0
    MW148603.1
TCATTATCGGATCTACATTTCTTATCGTATGTTTCCTACGACAACTAAGCTACCATTTTA   9300
    AB051263.1       ------------------------------------------------------
------ 0
    AF068544.1       ------------------------------------------------------
------ 0


    JX982502.1       ------------------------------------------------------
------ 0
    JX982501.1       ------------------------------------------------------
------ 0
    JX982498.1       ------------------------------------------------------
------ 0
    JX982497.1       ------------------------------------------------------
------ 0
    JX982495.1       ------------------------------------------------------
------ 0
    JX982496.1       ------------------------------------------------------
------ 0
    JX982500.1       ------------------------------------------------------
------ 0
    EU548051.1       ------------------------------------------------------
------ 0
    EU548044.1       ------------------------------------------------------
------ 0
    EU548042.1       ------------------------------------------------------
------ 0
    EU548043.1       ------------------------------------------------------
------ 0
    EU548047.1       ------------------------------------------------------
------ 0
    EU548050.1       ------------------------------------------------------
------ 0
    EU548048.1       ------------------------------------------------------
------ 0
    EU548049.1       ------------------------------------------------------
------ 0
    AF207722.1       ------------------------------------------------------
------ 0
    EU548037.1       ------------------------------------------------------
------ 0
    AF207723.1       ------------------------------------------------------
------ 0
    EU548038.1       ------------------------------------------------------
------ 0
    EU548036.1       ------------------------------------------------------
------ 0
```

```
   EU548035.1      ------------------------------------------------------
------ 0
   AF207720.1      ------------------------------------------------------
------ 0
   AB601576.1      ------------------------------------------------------
------ 0
   EU548040.1      ------------------------------------------------------
------ 0
   EU548039.1      ------------------------------------------------------
------ 0
   AF207721.1      ------------------------------------------------------
------ 0
   AF207724.1      ------------------------------------------------------
------ 0
   JX982499.1      ------------------------------------------------------
------ 0
   EU548041.1      ------------------------------------------------------
------ 0
   EU548045.1      ------------------------------------------------------
------ 0
   AF207725.1      ------------------------------------------------------
------ 0
   EU548046.1      ------------------------------------------------------
------ 0
   AF207714.1      ------------------------------------------------------
------ 0
   AF207713.1      ------------------------------------------------------
------ 0
   AF207712.1      ------------------------------------------------------
------ 0
   AY750628.1      ------------------------------------------------------
------ 1044
   EF689084.1      ------------------------------------------------------
------ 0
   EF689085.1      ------------------------------------------------------
------ 0
   AB119070.1      ------------------------------------------------------
------ 956
   EF987742.1      ------------------------------------------------------
------ 0
   AB026105.1      ------------------------------------------------------
------ 0
   MW148603.1
CATCTAATCACCATTTTGGATTCGAAGCAGCTGCCTGATATTGGCACTTTGTAGATGTCG   9360
   AB051263.1      ------------------------------------------------------
------ 0
   AF068544.1      ------------------------------------------------------
------ 0


   JX982502.1      ------------------------------------------------------
------ 0
   JX982501.1      ------------------------------------------------------
------ 0
   JX982498.1      ------------------------------------------------------
------ 0
   JX982497.1      ------------------------------------------------------
------ 0
   JX982495.1      ------------------------------------------------------
------ 0
   JX982496.1      ------------------------------------------------------
------ 0
   JX982500.1      ------------------------------------------------------
------ 0
   EU548051.1      ------------------------------------------------------
------ 0
```

```
    EU548044.1      ------------------------------------------------------------
------ 0
    EU548042.1      ------------------------------------------------------------
------ 0
    EU548043.1      ------------------------------------------------------------
------ 0
    EU548047.1      ------------------------------------------------------------
------ 0
    EU548050.1      ------------------------------------------------------------
------ 0
    EU548048.1      ------------------------------------------------------------
------ 0
    EU548049.1      ------------------------------------------------------------
------ 0
    AF207722.1      ------------------------------------------------------------
------ 0
    EU548037.1      ------------------------------------------------------------
------ 0
    AF207723.1      ------------------------------------------------------------
------ 0
    EU548038.1      ------------------------------------------------------------
------ 0
    EU548036.1      ------------------------------------------------------------
------ 0
    EU548035.1      ------------------------------------------------------------
------ 0
    AF207720.1      ------------------------------------------------------------
------ 0
    AB601576.1      ------------------------------------------------------------
------ 0
    EU548040.1      ------------------------------------------------------------
------ 0
    EU548039.1      ------------------------------------------------------------
------ 0
    AF207721.1      ------------------------------------------------------------
------ 0
    AF207724.1      ------------------------------------------------------------
------ 0
    JX982499.1      ------------------------------------------------------------
------ 0
    EU548041.1      ------------------------------------------------------------
------ 0
    EU548045.1      ------------------------------------------------------------
------ 0
    AF207725.1      ------------------------------------------------------------
------ 0
    EU548046.1      ------------------------------------------------------------
------ 0
    AF207714.1      ------------------------------------------------------------
------ 0
    AF207713.1      ------------------------------------------------------------
------ 0
    AF207712.1      ------------------------------------------------------------
------ 0
    AY750628.1      ------------------------------------------------------------
------ 1044
    EF689084.1      ------------------------------------------------------------
------ 0
    EF689085.1      ------------------------------------------------------------
------ 0
    AB119070.1      ------------------------------------------------------------
------ 956
    EF987742.1      ------------------------------------------------------------
------ 0
    AB026105.1      ------------------------------------------------------------
------ 0
```

MW148603.1
TATGACTATTCCTATATGTATCTATCTATTGATGAGGATCTTATTTCTCTAGTATCAACA 9420
AB051263.1 --
------ 0
AF068544.1 --
------ 0

JX982502.1 --
------ 0
JX982501.1 --
------ 0
JX982498.1 --
------ 0
JX982497.1 --
------ 0
JX982495.1 --
------ 0
JX982496.1 --
------ 0
JX982500.1 --
------ 0
EU548051.1 --
------ 0
EU548044.1 --
------ 0
EU548042.1 --
------ 0
EU548043.1 --
------ 0
EU548047.1 --
------ 0
EU548050.1 --
------ 0
EU548048.1 --
------ 0
EU548049.1 --
------ 0
AF207722.1 --
------ 0
EU548037.1 --
------ 0
AF207723.1 --
------ 0
EU548038.1 --
------ 0
EU548036.1 --
------ 0
EU548035.1 --
------ 0
AF207720.1 --
------ 0
AB601576.1 --
------ 0
EU548040.1 --
------ 0
EU548039.1 --
------ 0
AF207721.1 --
------ 0
AF207724.1 --
------ 0
JX982499.1 --
------ 0
EU548041.1 --
------ 0

EU548045.1 --
------ 0
AF207725.1 --
------ 0
EU548046.1 --
------ 0
AF207714.1 --
------ 0
AF207713.1 --
------ 0
AF207712.1 --
------ 0
AY750628.1 --
------ 1044
EF689084.1 --
------ 0
EF689085.1 --
------ 0
AB119070.1 --
------ 956
EF987742.1 --
------ 0
AB026105.1 --
------ 0
MW148603.1
AGTACAGTTGACTTCCAATTAACTAGTTCTGGTCTAACCCAGAGAGAAATAATAAATATA 9480
AB051263.1 --
------ 0
AF068544.1 --
------ 0

JX982502.1 --
------ 0
JX982501.1 --
------ 0
JX982498.1 --
------ 0
JX982497.1 --
------ 0
JX982495.1 --
------ 0
JX982496.1 --
------ 0
JX982500.1 --
------ 0
EU548051.1 --
------ 0
EU548044.1 --
------ 0
EU548042.1 --
------ 0
EU548043.1 --
------ 0
EU548047.1 --
------ 0
EU548050.1 --
------ 0
EU548048.1 --
------ 0
EU548049.1 --
------ 0
AF207722.1 --
------ 0
EU548037.1 --
------ 0

```
    AF207723.1      ------------------------------------------------------
------ 0
    EU548038.1      ------------------------------------------------------
------ 0
    EU548036.1      ------------------------------------------------------
------ 0
    EU548035.1      ------------------------------------------------------
------ 0
    AF207720.1      ------------------------------------------------------
------ 0
    AB601576.1      ------------------------------------------------------
------ 0
    EU548040.1      ------------------------------------------------------
------ 0
    EU548039.1      ------------------------------------------------------
------ 0
    AF207721.1      ------------------------------------------------------
------ 0
    AF207724.1      ------------------------------------------------------
------ 0
    JX982499.1      ------------------------------------------------------
------ 0
    EU548041.1      ------------------------------------------------------
------ 0
    EU548045.1      ------------------------------------------------------
------ 0
    AF207725.1      ------------------------------------------------------
------ 0
    EU548046.1      ------------------------------------------------------
------ 0
    AF207714.1      ------------------------------------------------------
------ 0
    AF207713.1      ------------------------------------------------------
------ 0
    AF207712.1      ------------------------------------------------------
------ 0
    AY750628.1      ------------------------------------------------------
------ 1044
    EF689084.1      ------------------------------------------------------
------ 0
    EF689085.1      ------------------------------------------------------
------ 0
    AB119070.1      ------------------------------------------------------
------ 956
    EF987742.1      ------------------------------------------------------
------ 0
    AB026105.1      ------------------------------------------------------
------ 0
    MW148603.1
ATATTAACCATACTTATCAATGTATCCCTAGCATCTCTACTTATTCTAATCGCATTCTGA   9540
    AB051263.1      ------------------------------------------------------
------ 0
    AF068544.1      ------------------------------------------------------
------ 0


    JX982502.1      ------------------------------------------------------
------ 0
    JX982501.1      ------------------------------------------------------
------ 0
    JX982498.1      ------------------------------------------------------
------ 0
    JX982497.1      ------------------------------------------------------
------ 0
    JX982495.1      ------------------------------------------------------
------ 0
```

```
   JX982496.1      ------------------------------------------------------
------ 0
   JX982500.1      ------------------------------------------------------
------ 0
   EU548051.1      ------------------------------------------------------
------ 0
   EU548044.1      ------------------------------------------------------
------ 0
   EU548042.1      ------------------------------------------------------
------ 0
   EU548043.1      ------------------------------------------------------
------ 0
   EU548047.1      ------------------------------------------------------
------ 0
   EU548050.1      ------------------------------------------------------
------ 0
   EU548048.1      ------------------------------------------------------
------ 0
   EU548049.1      ------------------------------------------------------
------ 0
   AF207722.1      ------------------------------------------------------
------ 0
   EU548037.1      ------------------------------------------------------
------ 0
   AF207723.1      ------------------------------------------------------
------ 0
   EU548038.1      ------------------------------------------------------
------ 0
   EU548036.1      ------------------------------------------------------
------ 0
   EU548035.1      ------------------------------------------------------
------ 0
   AF207720.1      ------------------------------------------------------
------ 0
   AB601576.1      ------------------------------------------------------
------ 0
   EU548040.1      ------------------------------------------------------
------ 0
   EU548039.1      ------------------------------------------------------
------ 0
   AF207721.1      ------------------------------------------------------
------ 0
   AF207724.1      ------------------------------------------------------
------ 0
   JX982499.1      ------------------------------------------------------
------ 0
   EU548041.1      ------------------------------------------------------
------ 0
   EU548045.1      ------------------------------------------------------
------ 0
   AF207725.1      ------------------------------------------------------
------ 0
   EU548046.1      ------------------------------------------------------
------ 0
   AF207714.1      ------------------------------------------------------
------ 0
   AF207713.1      ------------------------------------------------------
------ 0
   AF207712.1      ------------------------------------------------------
------ 0
   AY750628.1      ------------------------------------------------------
------ 1044
   EF689084.1      ------------------------------------------------------
------ 0
   EF689085.1      ------------------------------------------------------
------ 0
```

```
    AB119070.1       ------------------------------------------------------
------ 956
    EF987742.1       ------------------------------------------------------
------ 0
    AB026105.1       ------------------------------------------------------
------ 0
    MW148603.1
CTACCTCAACTAAATGTCTACACAGAAAAAGCAAGCCCATATGAATGTGGTTTTGATCCC   9600
    AB051263.1       ------------------------------------------------------
------ 0
    AF068544.1       ------------------------------------------------------
------ 0


    JX982502.1       ------------------------------------------------------
------ 0
    JX982501.1       ------------------------------------------------------
------ 0
    JX982498.1       ------------------------------------------------------
------ 0
    JX982497.1       ------------------------------------------------------
------ 0
    JX982495.1       ------------------------------------------------------
------ 0
    JX982496.1       ------------------------------------------------------
------ 0
    JX982500.1       ------------------------------------------------------
------ 0
    EU548051.1       ------------------------------------------------------
------ 0
    EU548044.1       ------------------------------------------------------
------ 0
    EU548042.1       ------------------------------------------------------
------ 0
    EU548043.1       ------------------------------------------------------
------ 0
    EU548047.1       ------------------------------------------------------
------ 0
    EU548050.1       ------------------------------------------------------
------ 0
    EU548048.1       ------------------------------------------------------
------ 0
    EU548049.1       ------------------------------------------------------
------ 0
    AF207722.1       ------------------------------------------------------
------ 0
    EU548037.1       ------------------------------------------------------
------ 0
    AF207723.1       ------------------------------------------------------
------ 0
    EU548038.1       ------------------------------------------------------
------ 0
    EU548036.1       ------------------------------------------------------
------ 0
    EU548035.1       ------------------------------------------------------
------ 0
    AF207720.1       ------------------------------------------------------
------ 0
    AB601576.1       ------------------------------------------------------
------ 0
    EU548040.1       ------------------------------------------------------
------ 0
    EU548039.1       ------------------------------------------------------
------ 0
    AF207721.1       ------------------------------------------------------
------ 0
```

```
    AF207724.1       ------------------------------------------------------
------ 0
    JX982499.1       ------------------------------------------------------
------ 0
    EU548041.1       ------------------------------------------------------
------ 0
    EU548045.1       ------------------------------------------------------
------ 0
    AF207725.1       ------------------------------------------------------
------ 0
    EU548046.1       ------------------------------------------------------
------ 0
    AF207714.1       ------------------------------------------------------
------ 0
    AF207713.1       ------------------------------------------------------
------ 0
    AF207712.1       ------------------------------------------------------
------ 0
    AY750628.1       ------------------------------------------------------
------ 1044
    EF689084.1       ------------------------------------------------------
------ 0
    EF689085.1       ------------------------------------------------------
------ 0
    AB119070.1       ------------------------------------------------------
------ 956
    EF987742.1       ------------------------------------------------------
------ 0
    AB026105.1       ------------------------------------------------------
------ 0
    MW148603.1
TTAGGATCAGCACGCTTACCATTCTCCATAAAATTTTTCCTGGTAGCTATTACATTCTTA   9660
    AB051263.1       ------------------------------------------------------
------ 0
    AF068544.1       ------------------------------------------------------
------ 0


    JX982502.1       ------------------------------------------------------
------ 0
    JX982501.1       ------------------------------------------------------
------ 0
    JX982498.1       ------------------------------------------------------
------ 0
    JX982497.1       ------------------------------------------------------
------ 0
    JX982495.1       ------------------------------------------------------
------ 0
    JX982496.1       ------------------------------------------------------
------ 0
    JX982500.1       ------------------------------------------------------
------ 0
    EU548051.1       ------------------------------------------------------
------ 0
    EU548044.1       ------------------------------------------------------
------ 0
    EU548042.1       ------------------------------------------------------
------ 0
    EU548043.1       ------------------------------------------------------
------ 0
    EU548047.1       ------------------------------------------------------
------ 0
    EU548050.1       ------------------------------------------------------
------ 0
    EU548048.1       ------------------------------------------------------
------ 0
```

EU548049.1 --
------ 0
AF207722.1 --
------ 0
EU548037.1 --
------ 0
AF207723.1 --
------ 0
EU548038.1 --
------ 0
EU548036.1 --
------ 0
EU548035.1 --
------ 0
AF207720.1 --
------ 0
AB601576.1 --
------ 0
EU548040.1 --
------ 0
EU548039.1 --
------ 0
AF207721.1 --
------ 0
AF207724.1 --
------ 0
JX982499.1 --
------ 0
EU548041.1 --
------ 0
EU548045.1 --
------ 0
AF207725.1 --
------ 0
EU548046.1 --
------ 0
AF207714.1 --
------ 0
AF207713.1 --
------ 0
AF207712.1 --
------ 0
AY750628.1 --
------ 1044
EF689084.1 --
------ 0
EF689085.1 --
------ 0
AB119070.1 --
------ 956
EF987742.1 --
------ 0
AB026105.1 --
------ 0
MW148603.1
TTATTCGACCTAGAAATTGCACTACTACTGCCTCTACCATGAGCCTCACAATCAATTAAC 9720
AB051263.1 --
------ 0
AF068544.1 --
------ 0

JX982502.1 --
------ 0
JX982501.1 --
------ 0

```
   JX982498.1          ------------------------------------------------------
------ 0
   JX982497.1          ------------------------------------------------------
------ 0
   JX982495.1          ------------------------------------------------------
------ 0
   JX982496.1          ------------------------------------------------------
------ 0
   JX982500.1          ------------------------------------------------------
------ 0
   EU548051.1          ------------------------------------------------------
------ 0
   EU548044.1          ------------------------------------------------------
------ 0
   EU548042.1          ------------------------------------------------------
------ 0
   EU548043.1          ------------------------------------------------------
------ 0
   EU548047.1          ------------------------------------------------------
------ 0
   EU548050.1          ------------------------------------------------------
------ 0
   EU548048.1          ------------------------------------------------------
------ 0
   EU548049.1          ------------------------------------------------------
------ 0
   AF207722.1          ------------------------------------------------------
------ 0
   EU548037.1          ------------------------------------------------------
------ 0
   AF207723.1          ------------------------------------------------------
------ 0
   EU548038.1          ------------------------------------------------------
------ 0
   EU548036.1          ------------------------------------------------------
------ 0
   EU548035.1          ------------------------------------------------------
------ 0
   AF207720.1          ------------------------------------------------------
------ 0
   AB601576.1          ------------------------------------------------------
------ 0
   EU548040.1          ------------------------------------------------------
------ 0
   EU548039.1          ------------------------------------------------------
------ 0
   AF207721.1          ------------------------------------------------------
------ 0
   AF207724.1          ------------------------------------------------------
------ 0
   JX982499.1          ------------------------------------------------------
------ 0
   EU548041.1          ------------------------------------------------------
------ 0
   EU548045.1          ------------------------------------------------------
------ 0
   AF207725.1          ------------------------------------------------------
------ 0
   EU548046.1          ------------------------------------------------------
------ 0
   AF207714.1          ------------------------------------------------------
------ 0
   AF207713.1          ------------------------------------------------------
------ 0
   AF207712.1          ------------------------------------------------------
------ 0
```

```
    AY750628.1       ------------------------------------------------------
------ 1044
    EF689084.1       ------------------------------------------------------
------ 0
    EF689085.1       ------------------------------------------------------
------ 0
    AB119070.1       ------------------------------------------------------
------ 956
    EF987742.1       ------------------------------------------------------
------ 0
    AB026105.1       ------------------------------------------------------
------ 0
    MW148603.1
CTAAAAACTACACTCACCATAGCACTAGCCCTAATCTCCCTACTAGCCGCAAGCCTAGCC   9780
    AB051263.1       ------------------------------------------------------
------ 0
    AF068544.1       ------------------------------------------------------
------ 0


    JX982502.1       ------------------------------------------------------
------ 0
    JX982501.1       ------------------------------------------------------
------ 0
    JX982498.1       ------------------------------------------------------
------ 0
    JX982497.1       ------------------------------------------------------
------ 0
    JX982495.1       ------------------------------------------------------
------ 0
    JX982496.1       ------------------------------------------------------
------ 0
    JX982500.1       ------------------------------------------------------
------ 0
    EU548051.1       ------------------------------------------------------
------ 0
    EU548044.1       ------------------------------------------------------
------ 0
    EU548042.1       ------------------------------------------------------
------ 0
    EU548043.1       ------------------------------------------------------
------ 0
    EU548047.1       ------------------------------------------------------
------ 0
    EU548050.1       ------------------------------------------------------
------ 0
    EU548048.1       ------------------------------------------------------
------ 0
    EU548049.1       ------------------------------------------------------
------ 0
    AF207722.1       ------------------------------------------------------
------ 0
    EU548037.1       ------------------------------------------------------
------ 0
    AF207723.1       ------------------------------------------------------
------ 0
    EU548038.1       ------------------------------------------------------
------ 0
    EU548036.1       ------------------------------------------------------
------ 0
    EU548035.1       ------------------------------------------------------
------ 0
    AF207720.1       ------------------------------------------------------
------ 0
    AB601576.1       ------------------------------------------------------
------ 0
```

EU548040.1 --
------ 0
EU548039.1 --
------ 0
AF207721.1 --
------ 0
AF207724.1 --
------ 0
JX982499.1 --
------ 0
EU548041.1 --
------ 0
EU548045.1 --
------ 0
AF207725.1 --
------ 0
EU548046.1 --
------ 0
AF207714.1 --
------ 0
AF207713.1 --
------ 0
AF207712.1 --
------ 0
AY750628.1 --
------ 1044
EF689084.1 --
------ 0
EF689085.1 --
------ 0
AB119070.1 --
------ 956
EF987742.1 --
------ 0
AB026105.1 --
------ 0
MW148603.1
TACGAATGAACTGAAGAGGGCCTAGAATGAAGCGAATATGATAATTAGTTTAACAAAAAC 9840
AB051263.1 --
------ 0
AF068544.1 --
------ 0

JX982502.1 --
------ 0
JX982501.1 --
------ 0
JX982498.1 --
------ 0
JX982497.1 --
------ 0
JX982495.1 --
------ 0
JX982496.1 --
------ 0
JX982500.1 --
------ 0
EU548051.1 --
------ 0
EU548044.1 --
------ 0
EU548042.1 --
------ 0
EU548043.1 --
------ 0

EU548047.1 --
------ 0
EU548050.1 --
------ 0
EU548048.1 --
------ 0
EU548049.1 --
------ 0
AF207722.1 --
------ 0
EU548037.1 --
------ 0
AF207723.1 --
------ 0
EU548038.1 --
------ 0
EU548036.1 --
------ 0
EU548035.1 --
------ 0
AF207720.1 --
------ 0
AB601576.1 --
------ 0
EU548040.1 --
------ 0
EU548039.1 --
------ 0
AF207721.1 --
------ 0
AF207724.1 --
------ 0
JX982499.1 --
------ 0
EU548041.1 --
------ 0
EU548045.1 --
------ 0
AF207725.1 --
------ 0
EU548046.1 --
------ 0
AF207714.1 --
------ 0
AF207713.1 --
------ 0
AF207712.1 --
------ 0
AY750628.1 --
------ 1044
EF689084.1 --
------ 0
EF689085.1 --
------ 0
AB119070.1 --
------ 956
EF987742.1 --
------ 0
AB026105.1 --
------ 0
MW148603.1
AAATGATTTCGACTCATTAGATTGTAACTCATATTACAATTATCAAATGTCCGTAGTATA 9900
AB051263.1 --
------ 0
AF068544.1 --
------ 0

JX982502.1   --
------ 0
JX982501.1   --
------ 0
JX982498.1   --
------ 0
JX982497.1   --
------ 0
JX982495.1   --
------ 0
JX982496.1   --
------ 0
JX982500.1   --
------ 0
EU548051.1   --
------ 0
EU548044.1   --
------ 0
EU548042.1   --
------ 0
EU548043.1   --
------ 0
EU548047.1   --
------ 0
EU548050.1   --
------ 0
EU548048.1   --
------ 0
EU548049.1   --
------ 0
AF207722.1   --
------ 0
EU548037.1   --
------ 0
AF207723.1   --
------ 0
EU548038.1   --
------ 0
EU548036.1   --
------ 0
EU548035.1   --
------ 0
AF207720.1   --
------ 0
AB601576.1   --
------ 0
EU548040.1   --
------ 0
EU548039.1   --
------ 0
AF207721.1   --
------ 0
AF207724.1   --
------ 0
JX982499.1   --
------ 0
EU548041.1   --
------ 0
EU548045.1   --
------ 0
AF207725.1   --
------ 0
EU548046.1   --
------ 0

```
    AF207714.1        ------------------------------------------------------
------ 0
    AF207713.1        ------------------------------------------------------
------ 0
    AF207712.1        ------------------------------------------------------
------ 0
    AY750628.1        ------------------------------------------------------
------ 1044
    EF689084.1        ------------------------------------------------------
------ 0
    EF689085.1        ------------------------------------------------------
------ 0
    AB119070.1        ------------------------------------------------------
------ 956
    EF987742.1        ------------------------------------------------------
------ 0
    AB026105.1        ------------------------------------------------------
------ 0
    MW148603.1
TATCAACATTTTTCTAGCCTTTACTCTATCCTTTATAGGGCTACTAATCTACCGATCCCA   9960
    AB051263.1        ------------------------------------------------------
------ 0
    AF068544.1        ------------------------------------------------------
------ 0


    JX982502.1        ------------------------------------------------------
------ 0
    JX982501.1        ------------------------------------------------------
------ 0
    JX982498.1        ------------------------------------------------------
------ 0
    JX982497.1        ------------------------------------------------------
------ 0
    JX982495.1        ------------------------------------------------------
------ 0
    JX982496.1        ------------------------------------------------------
------ 0
    JX982500.1        ------------------------------------------------------
------ 0
    EU548051.1        ------------------------------------------------------
------ 0
    EU548044.1        ------------------------------------------------------
------ 0
    EU548042.1        ------------------------------------------------------
------ 0
    EU548043.1        ------------------------------------------------------
------ 0
    EU548047.1        ------------------------------------------------------
------ 0
    EU548050.1        ------------------------------------------------------
------ 0
    EU548048.1        ------------------------------------------------------
------ 0
    EU548049.1        ------------------------------------------------------
------ 0
    AF207722.1        ------------------------------------------------------
------ 0
    EU548037.1        ------------------------------------------------------
------ 0
    AF207723.1        ------------------------------------------------------
------ 0
    EU548038.1        ------------------------------------------------------
------ 0
    EU548036.1        ------------------------------------------------------
------ 0
```

```
   EU548035.1      ------------------------------------------------------
------ 0
   AF207720.1      ------------------------------------------------------
------ 0
   AB601576.1      ------------------------------------------------------
------ 0
   EU548040.1      ------------------------------------------------------
------ 0
   EU548039.1      ------------------------------------------------------
------ 0
   AF207721.1      ------------------------------------------------------
------ 0
   AF207724.1      ------------------------------------------------------
------ 0
   JX982499.1      ------------------------------------------------------
------ 0
   EU548041.1      ------------------------------------------------------
------ 0
   EU548045.1      ------------------------------------------------------
------ 0
   AF207725.1      ------------------------------------------------------
------ 0
   EU548046.1      ------------------------------------------------------
------ 0
   AF207714.1      ------------------------------------------------------
------ 0
   AF207713.1      ------------------------------------------------------
------ 0
   AF207712.1      ------------------------------------------------------
------ 0
   AY750628.1      ------------------------------------------------------
------ 1044
   EF689084.1      ------------------------------------------------------
------ 0
   EF689085.1      ------------------------------------------------------
------ 0
   AB119070.1      ------------------------------------------------------
------ 956
   EF987742.1      ------------------------------------------------------
------ 0
   AB026105.1      ------------------------------------------------------
------ 0
   MW148603.1
CCTAATATCCTCTCTTCTCTGCCTAGAAGGTATGATGTTATCCCTCTTCGTCATAATAAC   10020
   AB051263.1      ------------------------------------------------------
------ 0
   AF068544.1      ------------------------------------------------------
------ 0


   JX982502.1      ------------------------------------------------------
------ 0
   JX982501.1      ------------------------------------------------------
------ 0
   JX982498.1      ------------------------------------------------------
------ 0
   JX982497.1      ------------------------------------------------------
------ 0
   JX982495.1      ------------------------------------------------------
------ 0
   JX982496.1      ------------------------------------------------------
------ 0
   JX982500.1      ------------------------------------------------------
------ 0
   EU548051.1      ------------------------------------------------------
------ 0
```

```
    EU548044.1          ------------------------------------------------------
------ 0
    EU548042.1          ------------------------------------------------------
------ 0
    EU548043.1          ------------------------------------------------------
------ 0
    EU548047.1          ------------------------------------------------------
------ 0
    EU548050.1          ------------------------------------------------------
------ 0
    EU548048.1          ------------------------------------------------------
------ 0
    EU548049.1          ------------------------------------------------------
------ 0
    AF207722.1          ------------------------------------------------------
------ 0
    EU548037.1          ------------------------------------------------------
------ 0
    AF207723.1          ------------------------------------------------------
------ 0
    EU548038.1          ------------------------------------------------------
------ 0
    EU548036.1          ------------------------------------------------------
------ 0
    EU548035.1          ------------------------------------------------------
------ 0
    AF207720.1          ------------------------------------------------------
------ 0
    AB601576.1          ------------------------------------------------------
------ 0
    EU548040.1          ------------------------------------------------------
------ 0
    EU548039.1          ------------------------------------------------------
------ 0
    AF207721.1          ------------------------------------------------------
------ 0
    AF207724.1          ------------------------------------------------------
------ 0
    JX982499.1          ------------------------------------------------------
------ 0
    EU548041.1          ------------------------------------------------------
------ 0
    EU548045.1          ------------------------------------------------------
------ 0
    AF207725.1          ------------------------------------------------------
------ 0
    EU548046.1          ------------------------------------------------------
------ 0
    AF207714.1          ------------------------------------------------------
------ 0
    AF207713.1          ------------------------------------------------------
------ 0
    AF207712.1          ------------------------------------------------------
------ 0
    AY750628.1          ------------------------------------------------------
------ 1044
    EF689084.1          ------------------------------------------------------
------ 0
    EF689085.1          ------------------------------------------------------
------ 0
    AB119070.1          ------------------------------------------------------
------ 956
    EF987742.1          ------------------------------------------------------
------ 0
    AB026105.1          ------------------------------------------------------
------ 0
```

MW148603.1
AGTCACCATCCTGGCAAATCACTTTACACTAGCCAGTATAACCCCTATTATCCTCCTTGT 10080
AB051263.1 --
------ 0
AF068544.1 --
------ 0

JX982502.1 --
------ 0
JX982501.1 --
------ 0
JX982498.1 --
------ 0
JX982497.1 --
------ 0
JX982495.1 --
------ 0
JX982496.1 --
------ 0
JX982500.1 --
------ 0
EU548051.1 --
------ 0
EU548044.1 --
------ 0
EU548042.1 --
------ 0
EU548043.1 --
------ 0
EU548047.1 --
------ 0
EU548050.1 --
------ 0
EU548048.1 --
------ 0
EU548049.1 --
------ 0
AF207722.1 --
------ 0
EU548037.1 --
------ 0
AF207723.1 --
------ 0
EU548038.1 --
------ 0
EU548036.1 --
------ 0
EU548035.1 --
------ 0
AF207720.1 --
------ 0
AB601576.1 --
------ 0
EU548040.1 --
------ 0
EU548039.1 --
------ 0
AF207721.1 --
------ 0
AF207724.1 --
------ 0
JX982499.1 --
------ 0
EU548041.1 --
------ 0

EU548045.1 --
------ 0
AF207725.1 --
------ 0
EU548046.1 --
------ 0
AF207714.1 --
------ 0
AF207713.1 --
------ 0
AF207712.1 --
------ 0
AY750628.1 --
------ 1044
EF689084.1 --
------ 0
EF689085.1 --
------ 0
AB119070.1 --
------ 956
EF987742.1 --
------ 0
AB026105.1 --
------ 0
MW148603.1
ATTTGCAGCCTGCGAAGCAGCATTAGGCTTATCCTTGCTAGTAATAATTTCCTCCACATA 10140
AB051263.1 --
------ 0
AF068544.1 --
------ 0

JX982502.1 --
------ 0
JX982501.1 --
------ 0
JX982498.1 --
------ 0
JX982497.1 --
------ 0
JX982495.1 --
------ 0
JX982496.1 --
------ 0
JX982500.1 --
------ 0
EU548051.1 --
------ 0
EU548044.1 --
------ 0
EU548042.1 --
------ 0
EU548043.1 --
------ 0
EU548047.1 --
------ 0
EU548050.1 --
------ 0
EU548048.1 --
------ 0
EU548049.1 --
------ 0
AF207722.1 --
------ 0
EU548037.1 --
------ 0

AF207723.1 --
------ 0
EU548038.1 --
------ 0
EU548036.1 --
------ 0
EU548035.1 --
------ 0
AF207720.1 --
------ 0
AB601576.1 --
------ 0
EU548040.1 --
------ 0
EU548039.1 --
------ 0
AF207721.1 --
------ 0
AF207724.1 --
------ 0
JX982499.1 --
------ 0
EU548041.1 --
------ 0
EU548045.1 --
------ 0
AF207725.1 --
------ 0
EU548046.1 --
------ 0
AF207714.1 --
------ 0
AF207713.1 --
------ 0
AF207712.1 --
------ 0
AY750628.1 --
------ 1044
EF689084.1 --
------ 0
EF689085.1 --
------ 0
AB119070.1 --
------ 956
EF987742.1 --
------ 0
AB026105.1 --
------ 0
MW148603.1
CGGGACAGATTATGTGCAAAACCTAAATCTATTACAATGCTAAAAATTATTATCCCAACC 10200
AB051263.1 --
------ 0
AF068544.1 --
------ 0

JX982502.1 --
------ 0
JX982501.1 --
------ 0
JX982498.1 --
------ 0
JX982497.1 --
------ 0
JX982495.1 --
------ 0

```
   JX982496.1      ------------------------------------------------------
------ 0
   JX982500.1      ------------------------------------------------------
------ 0
   EU548051.1      ------------------------------------------------------
------ 0
   EU548044.1      ------------------------------------------------------
------ 0
   EU548042.1      ------------------------------------------------------
------ 0
   EU548043.1      ------------------------------------------------------
------ 0
   EU548047.1      ------------------------------------------------------
------ 0
   EU548050.1      ------------------------------------------------------
------ 0
   EU548048.1      ------------------------------------------------------
------ 0
   EU548049.1      ------------------------------------------------------
------ 0
   AF207722.1      ------------------------------------------------------
------ 0
   EU548037.1      ------------------------------------------------------
------ 0
   AF207723.1      ------------------------------------------------------
------ 0
   EU548038.1      ------------------------------------------------------
------ 0
   EU548036.1      ------------------------------------------------------
------ 0
   EU548035.1      ------------------------------------------------------
------ 0
   AF207720.1      ------------------------------------------------------
------ 0
   AB601576.1      ------------------------------------------------------
------ 0
   EU548040.1      ------------------------------------------------------
------ 0
   EU548039.1      ------------------------------------------------------
------ 0
   AF207721.1      ------------------------------------------------------
------ 0
   AF207724.1      ------------------------------------------------------
------ 0
   JX982499.1      ------------------------------------------------------
------ 0
   EU548041.1      ------------------------------------------------------
------ 0
   EU548045.1      ------------------------------------------------------
------ 0
   AF207725.1      ------------------------------------------------------
------ 0
   EU548046.1      ------------------------------------------------------
------ 0
   AF207714.1      ------------------------------------------------------
------ 0
   AF207713.1      ------------------------------------------------------
------ 0
   AF207712.1      ------------------------------------------------------
------ 0
   AY750628.1      ------------------------------------------------------
------ 1044
   EF689084.1      ------------------------------------------------------
------ 0
   EF689085.1      ------------------------------------------------------
------ 0
```

AB119070.1 --
------ 956
EF987742.1 --
------ 0
AB026105.1 --
------ 0
MW148603.1
ATAATATTAATCCCCTTAACATGACTATCAAAACCCAATATAATTTGAATTAATACAACA 10260
AB051263.1 --
------ 0
AF068544.1 --
------ 0

JX982502.1 --
------ 0
JX982501.1 --
------ 0
JX982498.1 --
------ 0
JX982497.1 --
------ 0
JX982495.1 --
------ 0
JX982496.1 --
------ 0
JX982500.1 --
------ 0
EU548051.1 --
------ 0
EU548044.1 --
------ 0
EU548042.1 --
------ 0
EU548043.1 --
------ 0
EU548047.1 --
------ 0
EU548050.1 --
------ 0
EU548048.1 --
------ 0
EU548049.1 --
------ 0
AF207722.1 --
------ 0
EU548037.1 --
------ 0
AF207723.1 --
------ 0
EU548038.1 --
------ 0
EU548036.1 --
------ 0
EU548035.1 --
------ 0
AF207720.1 --
------ 0
AB601576.1 --
------ 0
EU548040.1 --
------ 0
EU548039.1 --
------ 0
AF207721.1 --
------ 0

```
    AF207724.1       ------------------------------------------------------
------ 0
    JX982499.1       ------------------------------------------------------
------ 0
    EU548041.1       ------------------------------------------------------
------ 0
    EU548045.1       ------------------------------------------------------
------ 0
    AF207725.1       ------------------------------------------------------
------ 0
    EU548046.1       ------------------------------------------------------
------ 0
    AF207714.1       ------------------------------------------------------
------ 0
    AF207713.1       ------------------------------------------------------
------ 0
    AF207712.1       ------------------------------------------------------
------ 0
    AY750628.1       ------------------------------------------------------
------ 1044
    EF689084.1       ------------------------------------------------------
------ 0
    EF689085.1       ------------------------------------------------------
------ 0
    AB119070.1       ------------------------------------------------------
------ 956
    EF987742.1       ------------------------------------------------------
------ 0
    AB026105.1       ------------------------------------------------------
------ 0
    MW148603.1
GCTCACAGCATATTAATTAGTCTAATCAGTCTAACGTACCTAAATCAACTCACAGACAAT   10320
    AB051263.1       ------------------------------------------------------
------ 0
    AF068544.1       ------------------------------------------------------
------ 0


    JX982502.1       ------------------------------------------------------
------ 0
    JX982501.1       ------------------------------------------------------
------ 0
    JX982498.1       ------------------------------------------------------
------ 0
    JX982497.1       ------------------------------------------------------
------ 0
    JX982495.1       ------------------------------------------------------
------ 0
    JX982496.1       ------------------------------------------------------
------ 0
    JX982500.1       ------------------------------------------------------
------ 0
    EU548051.1       ------------------------------------------------------
------ 0
    EU548044.1       ------------------------------------------------------
------ 0
    EU548042.1       ------------------------------------------------------
------ 0
    EU548043.1       ------------------------------------------------------
------ 0
    EU548047.1       ------------------------------------------------------
------ 0
    EU548050.1       ------------------------------------------------------
------ 0
    EU548048.1       ------------------------------------------------------
------ 0
```

```
    EU548049.1      ------------------------------------------------------
------ 0
    AF207722.1      ------------------------------------------------------
------ 0
    EU548037.1      ------------------------------------------------------
------ 0
    AF207723.1      ------------------------------------------------------
------ 0
    EU548038.1      ------------------------------------------------------
------ 0
    EU548036.1      ------------------------------------------------------
------ 0
    EU548035.1      ------------------------------------------------------
------ 0
    AF207720.1      ------------------------------------------------------
------ 0
    AB601576.1      ------------------------------------------------------
------ 0
    EU548040.1      ------------------------------------------------------
------ 0
    EU548039.1      ------------------------------------------------------
------ 0
    AF207721.1      ------------------------------------------------------
------ 0
    AF207724.1      ------------------------------------------------------
------ 0
    JX982499.1      ------------------------------------------------------
------ 0
    EU548041.1      ------------------------------------------------------
------ 0
    EU548045.1      ------------------------------------------------------
------ 0
    AF207725.1      ------------------------------------------------------
------ 0
    EU548046.1      ------------------------------------------------------
------ 0
    AF207714.1      ------------------------------------------------------
------ 0
    AF207713.1      ------------------------------------------------------
------ 0
    AF207712.1      ------------------------------------------------------
------ 0
    AY750628.1      ------------------------------------------------------
------ 1044
    EF689084.1      ------------------------------------------------------
------ 0
    EF689085.1      ------------------------------------------------------
------ 0
    AB119070.1      ------------------------------------------------------
------ 956
    EF987742.1      ------------------------------------------------------
------ 0
    AB026105.1      ------------------------------------------------------
------ 0
    MW148603.1
AGCCTAAACTTCTCATTACTATTCTTCGCAGACTCCTTATCAACGCCCTTACTAGTGCTC   10380
    AB051263.1      ------------------------------------------------------
------ 0
    AF068544.1      ------------------------------------------------------
------ 0


    JX982502.1      ------------------------------------------------------
------ 0
    JX982501.1      ------------------------------------------------------
------ 0
```

```
JX982498.1      ------------------------------------------------------
------ 0
JX982497.1      ------------------------------------------------------
------ 0
JX982495.1      ------------------------------------------------------
------ 0
JX982496.1      ------------------------------------------------------
------ 0
JX982500.1      ------------------------------------------------------
------ 0
EU548051.1      ------------------------------------------------------
------ 0
EU548044.1      ------------------------------------------------------
------ 0
EU548042.1      ------------------------------------------------------
------ 0
EU548043.1      ------------------------------------------------------
------ 0
EU548047.1      ------------------------------------------------------
------ 0
EU548050.1      ------------------------------------------------------
------ 0
EU548048.1      ------------------------------------------------------
------ 0
EU548049.1      ------------------------------------------------------
------ 0
AF207722.1      ------------------------------------------------------
------ 0
EU548037.1      ------------------------------------------------------
------ 0
AF207723.1      ------------------------------------------------------
------ 0
EU548038.1      ------------------------------------------------------
------ 0
EU548036.1      ------------------------------------------------------
------ 0
EU548035.1      ------------------------------------------------------
------ 0
AF207720.1      ------------------------------------------------------
------ 0
AB601576.1      ------------------------------------------------------
------ 0
EU548040.1      ------------------------------------------------------
------ 0
EU548039.1      ------------------------------------------------------
------ 0
AF207721.1      ------------------------------------------------------
------ 0
AF207724.1      ------------------------------------------------------
------ 0
JX982499.1      ------------------------------------------------------
------ 0
EU548041.1      ------------------------------------------------------
------ 0
EU548045.1      ------------------------------------------------------
------ 0
AF207725.1      ------------------------------------------------------
------ 0
EU548046.1      ------------------------------------------------------
------ 0
AF207714.1      ------------------------------------------------------
------ 0
AF207713.1      ------------------------------------------------------
------ 0
AF207712.1      ------------------------------------------------------
------ 0
```

```
    AY750628.1         ------------------------------------------------------
------ 1044
    EF689084.1         ------------------------------------------------------
------ 0
    EF689085.1         ------------------------------------------------------
------ 0
    AB119070.1         ------------------------------------------------------
------ 956
    EF987742.1         ------------------------------------------------------
------ 0
    AB026105.1         ------------------------------------------------------
------ 0
    MW148603.1
ACAACATGGCTTCTTCCCCTGATGCTCATAGCAAGCCAACACCACCTGTCAAAAGAAACT   10440
    AB051263.1         ------------------------------------------------------
------ 0
    AF068544.1         ------------------------------------------------------
------ 0


    JX982502.1         ------------------------------------------------------
------ 0
    JX982501.1         ------------------------------------------------------
------ 0
    JX982498.1         ------------------------------------------------------
------ 0
    JX982497.1         ------------------------------------------------------
------ 0
    JX982495.1         ------------------------------------------------------
------ 0
    JX982496.1         ------------------------------------------------------
------ 0
    JX982500.1         ------------------------------------------------------
------ 0
    EU548051.1         ------------------------------------------------------
------ 0
    EU548044.1         ------------------------------------------------------
------ 0
    EU548042.1         ------------------------------------------------------
------ 0
    EU548043.1         ------------------------------------------------------
------ 0
    EU548047.1         ------------------------------------------------------
------ 0
    EU548050.1         ------------------------------------------------------
------ 0
    EU548048.1         ------------------------------------------------------
------ 0
    EU548049.1         ------------------------------------------------------
------ 0
    AF207722.1         ------------------------------------------------------
------ 0
    EU548037.1         ------------------------------------------------------
------ 0
    AF207723.1         ------------------------------------------------------
------ 0
    EU548038.1         ------------------------------------------------------
------ 0
    EU548036.1         ------------------------------------------------------
------ 0
    EU548035.1         ------------------------------------------------------
------ 0
    AF207720.1         ------------------------------------------------------
------ 0
    AB601576.1         ------------------------------------------------------
------ 0
```

```
   EU548040.1       ------------------------------------------------------
------ 0
   EU548039.1       ------------------------------------------------------
------ 0
   AF207721.1       ------------------------------------------------------
------ 0
   AF207724.1       ------------------------------------------------------
------ 0
   JX982499.1       ------------------------------------------------------
------ 0
   EU548041.1       ------------------------------------------------------
------ 0
   EU548045.1       ------------------------------------------------------
------ 0
   AF207725.1       ------------------------------------------------------
------ 0
   EU548046.1       ------------------------------------------------------
------ 0
   AF207714.1       ------------------------------------------------------
------ 0
   AF207713.1       ------------------------------------------------------
------ 0
   AF207712.1       ------------------------------------------------------
------ 0
   AY750628.1       ------------------------------------------------------
------ 1044
   EF689084.1       ------------------------------------------------------
------ 0
   EF689085.1       ------------------------------------------------------
------ 0
   AB119070.1       ------------------------------------------------------
------ 956
   EF987742.1       ------------------------------------------------------
------ 0
   AB026105.1       ------------------------------------------------------
------ 0
   MW148603.1
CTAACCCGCAAAAAACTCTACATCACAATGCTAGTAATATTACAACTGTTCCTAATTATA   10500
   AB051263.1       ------------------------------------------------------
------ 0
   AF068544.1       ------------------------------------------------------
------ 0


   JX982502.1       ------------------------------------------------------
------ 0
   JX982501.1       ------------------------------------------------------
------ 0
   JX982498.1       ------------------------------------------------------
------ 0
   JX982497.1       ------------------------------------------------------
------ 0
   JX982495.1       ------------------------------------------------------
------ 0
   JX982496.1       ------------------------------------------------------
------ 0
   JX982500.1       ------------------------------------------------------
------ 0
   EU548051.1       ------------------------------------------------------
------ 0
   EU548044.1       ------------------------------------------------------
------ 0
   EU548042.1       ------------------------------------------------------
------ 0
   EU548043.1       ------------------------------------------------------
------ 0
```

EU548047.1 --
------ 0
EU548050.1 --
------ 0
EU548048.1 --
------ 0
EU548049.1 --
------ 0
AF207722.1 --
------ 0
EU548037.1 --
------ 0
AF207723.1 --
------ 0
EU548038.1 --
------ 0
EU548036.1 --
------ 0
EU548035.1 --
------ 0
AF207720.1 --
------ 0
AB601576.1 --
------ 0
EU548040.1 --
------ 0
EU548039.1 --
------ 0
AF207721.1 --
------ 0
AF207724.1 --
------ 0
JX982499.1 --
------ 0
EU548041.1 --
------ 0
EU548045.1 --
------ 0
AF207725.1 --
------ 0
EU548046.1 --
------ 0
AF207714.1 --
------ 0
AF207713.1 --
------ 0
AF207712.1 --
------ 0
AY750628.1 --
------ 1044
EF689084.1 --
------ 0
EF689085.1 --
------ 0
AB119070.1 --
------ 956
EF987742.1 --
------ 0
AB026105.1 --
------ 0
MW148603.1
ACATTTACAGCCACAGAACTAATCATATTCTACATTCTATTTGAAGCTACTCTCATGCCA 10560
AB051263.1 --
------ 0
AF068544.1 --
------ 0

```
    JX982502.1        ------------------------------------------------------
------ 0
    JX982501.1        ------------------------------------------------------
------ 0
    JX982498.1        ------------------------------------------------------
------ 0
    JX982497.1        ------------------------------------------------------
------ 0
    JX982495.1        ------------------------------------------------------
------ 0
    JX982496.1        ------------------------------------------------------
------ 0
    JX982500.1        ------------------------------------------------------
------ 0
    EU548051.1        ------------------------------------------------------
------ 0
    EU548044.1        ------------------------------------------------------
------ 0
    EU548042.1        ------------------------------------------------------
------ 0
    EU548043.1        ------------------------------------------------------
------ 0
    EU548047.1        ------------------------------------------------------
------ 0
    EU548050.1        ------------------------------------------------------
------ 0
    EU548048.1        ------------------------------------------------------
------ 0
    EU548049.1        ------------------------------------------------------
------ 0
    AF207722.1        ------------------------------------------------------
------ 0
    EU548037.1        ------------------------------------------------------
------ 0
    AF207723.1        ------------------------------------------------------
------ 0
    EU548038.1        ------------------------------------------------------
------ 0
    EU548036.1        ------------------------------------------------------
------ 0
    EU548035.1        ------------------------------------------------------
------ 0
    AF207720.1        ------------------------------------------------------
------ 0
    AB601576.1        ------------------------------------------------------
------ 0
    EU548040.1        ------------------------------------------------------
------ 0
    EU548039.1        ------------------------------------------------------
------ 0
    AF207721.1        ------------------------------------------------------
------ 0
    AF207724.1        ------------------------------------------------------
------ 0
    JX982499.1        ------------------------------------------------------
------ 0
    EU548041.1        ------------------------------------------------------
------ 0
    EU548045.1        ------------------------------------------------------
------ 0
    AF207725.1        ------------------------------------------------------
------ 0
    EU548046.1        ------------------------------------------------------
------ 0
```

AF207714.1 --
------ 0
AF207713.1 --
------ 0
AF207712.1 --
------ 0
AY750628.1 --
------ 1044
EF689084.1 --
------ 0
EF689085.1 --
------ 0
AB119070.1 --
------ 956
EF987742.1 --
------ 0
AB026105.1 --
------ 0
MW148603.1
ACACTAATTATCATCACTCGATGGGGCAACCAGACAGAACGACTAAATGCTGGCCTATAC 10620
AB051263.1 --
------ 0
AF068544.1 --
------ 0

JX982502.1 --
------ 0
JX982501.1 --
------ 0
JX982498.1 --
------ 0
JX982497.1 --
------ 0
JX982495.1 --
------ 0
JX982496.1 --
------ 0
JX982500.1 --
------ 0
EU548051.1 --
------ 0
EU548044.1 --
------ 0
EU548042.1 --
------ 0
EU548043.1 --
------ 0
EU548047.1 --
------ 0
EU548050.1 --
------ 0
EU548048.1 --
------ 0
EU548049.1 --
------ 0
AF207722.1 --
------ 0
EU548037.1 --
------ 0
AF207723.1 --
------ 0
EU548038.1 --
------ 0
EU548036.1 --
------ 0

```
   EU548035.1      ------------------------------------------------------
------ 0
   AF207720.1      ------------------------------------------------------
------ 0
   AB601576.1      ------------------------------------------------------
------ 0
   EU548040.1      ------------------------------------------------------
------ 0
   EU548039.1      ------------------------------------------------------
------ 0
   AF207721.1      ------------------------------------------------------
------ 0
   AF207724.1      ------------------------------------------------------
------ 0
   JX982499.1      ------------------------------------------------------
------ 0
   EU548041.1      ------------------------------------------------------
------ 0
   EU548045.1      ------------------------------------------------------
------ 0
   AF207725.1      ------------------------------------------------------
------ 0
   EU548046.1      ------------------------------------------------------
------ 0
   AF207714.1      ------------------------------------------------------
------ 0
   AF207713.1      ------------------------------------------------------
------ 0
   AF207712.1      ------------------------------------------------------
------ 0
   AY750628.1      ------------------------------------------------------
------ 1044
   EF689084.1      ------------------------------------------------------
------ 0
   EF689085.1      ------------------------------------------------------
------ 0
   AB119070.1      ------------------------------------------------------
------ 956
   EF987742.1      ------------------------------------------------------
------ 0
   AB026105.1      ------------------------------------------------------
------ 0
   MW148603.1
TTTTTATTTTACACTCTAGTGGGCTCCTTGCCCCTCCTAATCGCCCTACTATGACTCCAA   10680
   AB051263.1      ------------------------------------------------------
------ 0
   AF068544.1      ------------------------------------------------------
------ 0


   JX982502.1      ------------------------------------------------------
------ 0
   JX982501.1      ------------------------------------------------------
------ 0
   JX982498.1      ------------------------------------------------------
------ 0
   JX982497.1      ------------------------------------------------------
------ 0
   JX982495.1      ------------------------------------------------------
------ 0
   JX982496.1      ------------------------------------------------------
------ 0
   JX982500.1      ------------------------------------------------------
------ 0
   EU548051.1      ------------------------------------------------------
------ 0
```

```
   EU548044.1      ------------------------------------------------------
------ 0
   EU548042.1      ------------------------------------------------------
------ 0
   EU548043.1      ------------------------------------------------------
------ 0
   EU548047.1      ------------------------------------------------------
------ 0
   EU548050.1      ------------------------------------------------------
------ 0
   EU548048.1      ------------------------------------------------------
------ 0
   EU548049.1      ------------------------------------------------------
------ 0
   AF207722.1      ------------------------------------------------------
------ 0
   EU548037.1      ------------------------------------------------------
------ 0
   AF207723.1      ------------------------------------------------------
------ 0
   EU548038.1      ------------------------------------------------------
------ 0
   EU548036.1      ------------------------------------------------------
------ 0
   EU548035.1      ------------------------------------------------------
------ 0
   AF207720.1      ------------------------------------------------------
------ 0
   AB601576.1      ------------------------------------------------------
------ 0
   EU548040.1      ------------------------------------------------------
------ 0
   EU548039.1      ------------------------------------------------------
------ 0
   AF207721.1      ------------------------------------------------------
------ 0
   AF207724.1      ------------------------------------------------------
------ 0
   JX982499.1      ------------------------------------------------------
------ 0
   EU548041.1      ------------------------------------------------------
------ 0
   EU548045.1      ------------------------------------------------------
------ 0
   AF207725.1      ------------------------------------------------------
------ 0
   EU548046.1      ------------------------------------------------------
------ 0
   AF207714.1      ------------------------------------------------------
------ 0
   AF207713.1      ------------------------------------------------------
------ 0
   AF207712.1      ------------------------------------------------------
------ 0
   AY750628.1      ------------------------------------------------------
------ 1044
   EF689084.1      ------------------------------------------------------
------ 0
   EF689085.1      ------------------------------------------------------
------ 0
   AB119070.1      ------------------------------------------------------
------ 956
   EF987742.1      ------------------------------------------------------
------ 0
   AB026105.1      ------------------------------------------------------
------ 0
```

```
    MW148603.1
AACAACTTAGGTACCCTTAATCTATTAATCATACAATACTGAGCACAACCCTTACCAAAC    10740
    AB051263.1      ------------------------------------------------------
------ 0
    AF068544.1      ------------------------------------------------------
------ 0


    JX982502.1      ------------------------------------------------------
------ 0
    JX982501.1      ------------------------------------------------------
------ 0
    JX982498.1      ------------------------------------------------------
------ 0
    JX982497.1      ------------------------------------------------------
------ 0
    JX982495.1      ------------------------------------------------------
------ 0
    JX982496.1      ------------------------------------------------------
------ 0
    JX982500.1      ------------------------------------------------------
------ 0
    EU548051.1      ------------------------------------------------------
------ 0
    EU548044.1      ------------------------------------------------------
------ 0
    EU548042.1      ------------------------------------------------------
------ 0
    EU548043.1      ------------------------------------------------------
------ 0
    EU548047.1      ------------------------------------------------------
------ 0
    EU548050.1      ------------------------------------------------------
------ 0
    EU548048.1      ------------------------------------------------------
------ 0
    EU548049.1      ------------------------------------------------------
------ 0
    AF207722.1      ------------------------------------------------------
------ 0
    EU548037.1      ------------------------------------------------------
------ 0
    AF207723.1      ------------------------------------------------------
------ 0
    EU548038.1      ------------------------------------------------------
------ 0
    EU548036.1      ------------------------------------------------------
------ 0
    EU548035.1      ------------------------------------------------------
------ 0
    AF207720.1      ------------------------------------------------------
------ 0
    AB601576.1      ------------------------------------------------------
------ 0
    EU548040.1      ------------------------------------------------------
------ 0
    EU548039.1      ------------------------------------------------------
------ 0
    AF207721.1      ------------------------------------------------------
------ 0
    AF207724.1      ------------------------------------------------------
------ 0
    JX982499.1      ------------------------------------------------------
------ 0
    EU548041.1      ------------------------------------------------------
------ 0
```

EU548045.1 --
------ 0
AF207725.1 --
------ 0
EU548046.1 --
------ 0
AF207714.1 --
------ 0
AF207713.1 --
------ 0
AF207712.1 --
------ 0
AY750628.1 --
------ 1044
EF689084.1 --
------ 0
EF689085.1 --
------ 0
AB119070.1 --
------ 956
EF987742.1 --
------ 0
AB026105.1 --
------ 0
MW148603.1
TCTTGATCCAACACCCTACTATGACTAGCATGCATAATAGCATTCATAGTTAAAATACCC 10800
AB051263.1 --
------ 0
AF068544.1 --
------ 0

JX982502.1 --
------ 0
JX982501.1 --
------ 0
JX982498.1 --
------ 0
JX982497.1 --
------ 0
JX982495.1 --
------ 0
JX982496.1 --
------ 0
JX982500.1 --
------ 0
EU548051.1 --
------ 0
EU548044.1 --
------ 0
EU548042.1 --
------ 0
EU548043.1 --
------ 0
EU548047.1 --
------ 0
EU548050.1 --
------ 0
EU548048.1 --
------ 0
EU548049.1 --
------ 0
AF207722.1 --
------ 0
EU548037.1 --
------ 0

AF207723.1 -- 0
EU548038.1 -- 0
EU548036.1 -- 0
EU548035.1 -- 0
AF207720.1 -- 0
AB601576.1 -- 0
EU548040.1 -- 0
EU548039.1 -- 0
AF207721.1 -- 0
AF207724.1 -- 0
JX982499.1 -- 0
EU548041.1 -- 0
EU548045.1 -- 0
AF207725.1 -- 0
EU548046.1 -- 0
AF207714.1 -- 0
AF207713.1 -- 0
AF207712.1 -- 0
AY750628.1 -- 1044
EF689084.1 -- 0
EF689085.1 -- 0
AB119070.1 -- 956
EF987742.1 -- 0
AB026105.1 -- 0
MW148603.1 CTATATGGCCTCCACCTGTGACTACCAAAAGCCCATGTAGAAGCCCCTATCGCGGGGTCC 10860
AB051263.1 -- 0
AF068544.1 -- 0

JX982502.1 -- 0
JX982501.1 -- 0
JX982498.1 -- 0
JX982497.1 -- 0
JX982495.1 -- 0

```
   JX982496.1        ------------------------------------------------------
------ 0
   JX982500.1        ------------------------------------------------------
------ 0
   EU548051.1        ------------------------------------------------------
------ 0
   EU548044.1        ------------------------------------------------------
------ 0
   EU548042.1        ------------------------------------------------------
------ 0
   EU548043.1        ------------------------------------------------------
------ 0
   EU548047.1        ------------------------------------------------------
------ 0
   EU548050.1        ------------------------------------------------------
------ 0
   EU548048.1        ------------------------------------------------------
------ 0
   EU548049.1        ------------------------------------------------------
------ 0
   AF207722.1        ------------------------------------------------------
------ 0
   EU548037.1        ------------------------------------------------------
------ 0
   AF207723.1        ------------------------------------------------------
------ 0
   EU548038.1        ------------------------------------------------------
------ 0
   EU548036.1        ------------------------------------------------------
------ 0
   EU548035.1        ------------------------------------------------------
------ 0
   AF207720.1        ------------------------------------------------------
------ 0
   AB601576.1        ------------------------------------------------------
------ 0
   EU548040.1        ------------------------------------------------------
------ 0
   EU548039.1        ------------------------------------------------------
------ 0
   AF207721.1        ------------------------------------------------------
------ 0
   AF207724.1        ------------------------------------------------------
------ 0
   JX982499.1        ------------------------------------------------------
------ 0
   EU548041.1        ------------------------------------------------------
------ 0
   EU548045.1        ------------------------------------------------------
------ 0
   AF207725.1        ------------------------------------------------------
------ 0
   EU548046.1        ------------------------------------------------------
------ 0
   AF207714.1        ------------------------------------------------------
------ 0
   AF207713.1        ------------------------------------------------------
------ 0
   AF207712.1        ------------------------------------------------------
------ 0
   AY750628.1        ------------------------------------------------------
------ 1044
   EF689084.1        ------------------------------------------------------
------ 0
   EF689085.1        ------------------------------------------------------
------ 0
```

AB119070.1 --
------ 956
EF987742.1 --
------ 0
AB026105.1 --
------ 0
MW148603.1
ATAGTCCTTGCCGCCGTACTCCTTAAGCTAGGAGGATATGGAATGATGCGAATCACCATA 10920
AB051263.1 --
------ 0
AF068544.1 --
------ 0

JX982502.1 --
------ 0
JX982501.1 --
------ 0
JX982498.1 --
------ 0
JX982497.1 --
------ 0
JX982495.1 --
------ 0
JX982496.1 --
------ 0
JX982500.1 --
------ 0
EU548051.1 --
------ 0
EU548044.1 --
------ 0
EU548042.1 --
------ 0
EU548043.1 --
------ 0
EU548047.1 --
------ 0
EU548050.1 --
------ 0
EU548048.1 --
------ 0
EU548049.1 --
------ 0
AF207722.1 --
------ 0
EU548037.1 --
------ 0
AF207723.1 --
------ 0
EU548038.1 --
------ 0
EU548036.1 --
------ 0
EU548035.1 --
------ 0
AF207720.1 --
------ 0
AB601576.1 --
------ 0
EU548040.1 --
------ 0
EU548039.1 --
------ 0
AF207721.1 --
------ 0

```
    AF207724.1      ------------------------------------------------------
------ 0
    JX982499.1      ------------------------------------------------------
------ 0
    EU548041.1      ------------------------------------------------------
------ 0
    EU548045.1      ------------------------------------------------------
------ 0
    AF207725.1      ------------------------------------------------------
------ 0
    EU548046.1      ------------------------------------------------------
------ 0
    AF207714.1      ------------------------------------------------------
------ 0
    AF207713.1      ------------------------------------------------------
------ 0
    AF207712.1      ------------------------------------------------------
------ 0
    AY750628.1      ------------------------------------------------------
------ 1044
    EF689084.1      ------------------------------------------------------
------ 0
    EF689085.1      ------------------------------------------------------
------ 0
    AB119070.1      ------------------------------------------------------
------ 956
    EF987742.1      ------------------------------------------------------
------ 0
    AB026105.1      ------------------------------------------------------
------ 0
    MW148603.1
CTACTAAATCCACTAACAAGCTACATAGCATACCCCTTTATAATACTATCATTATGAGGG   10980
    AB051263.1      ------------------------------------------------------
------ 0
    AF068544.1      ------------------------------------------------------
------ 0


    JX982502.1      ------------------------------------------------------
------ 0
    JX982501.1      ------------------------------------------------------
------ 0
    JX982498.1      ------------------------------------------------------
------ 0
    JX982497.1      ------------------------------------------------------
------ 0
    JX982495.1      ------------------------------------------------------
------ 0
    JX982496.1      ------------------------------------------------------
------ 0
    JX982500.1      ------------------------------------------------------
------ 0
    EU548051.1      ------------------------------------------------------
------ 0
    EU548044.1      ------------------------------------------------------
------ 0
    EU548042.1      ------------------------------------------------------
------ 0
    EU548043.1      ------------------------------------------------------
------ 0
    EU548047.1      ------------------------------------------------------
------ 0
    EU548050.1      ------------------------------------------------------
------ 0
    EU548048.1      ------------------------------------------------------
------ 0
```

```
   EU548049.1      ------------------------------------------------------
------ 0
   AF207722.1      ------------------------------------------------------
------ 0
   EU548037.1      ------------------------------------------------------
------ 0
   AF207723.1      ------------------------------------------------------
------ 0
   EU548038.1      ------------------------------------------------------
------ 0
   EU548036.1      ------------------------------------------------------
------ 0
   EU548035.1      ------------------------------------------------------
------ 0
   AF207720.1      ------------------------------------------------------
------ 0
   AB601576.1      ------------------------------------------------------
------ 0
   EU548040.1      ------------------------------------------------------
------ 0
   EU548039.1      ------------------------------------------------------
------ 0
   AF207721.1      ------------------------------------------------------
------ 0
   AF207724.1      ------------------------------------------------------
------ 0
   JX982499.1      ------------------------------------------------------
------ 0
   EU548041.1      ------------------------------------------------------
------ 0
   EU548045.1      ------------------------------------------------------
------ 0
   AF207725.1      ------------------------------------------------------
------ 0
   EU548046.1      ------------------------------------------------------
------ 0
   AF207714.1      ------------------------------------------------------
------ 0
   AF207713.1      ------------------------------------------------------
------ 0
   AF207712.1      ------------------------------------------------------
------ 0
   AY750628.1      ------------------------------------------------------
------ 1044
   EF689084.1      ------------------------------------------------------
------ 0
   EF689085.1      ------------------------------------------------------
------ 0
   AB119070.1      ------------------------------------------------------
------ 956
   EF987742.1      ------------------------------------------------------
------ 0
   AB026105.1      ------------------------------------------------------
------ 0
   MW148603.1
ATAATCATAACCAGCTCCATCTGCTTACGCCAAACAGACCTAAAATCCTTAATTGCCTAC   11040
   AB051263.1      ------------------------------------------------------
------ 0
   AF068544.1      ------------------------------------------------------
------ 0


   JX982502.1      ------------------------------------------------------
------ 0
   JX982501.1      ------------------------------------------------------
------ 0
```

```
    JX982498.1          ------------------------------------------------------
------ 0
    JX982497.1          ------------------------------------------------------
------ 0
    JX982495.1          ------------------------------------------------------
------ 0
    JX982496.1          ------------------------------------------------------
------ 0
    JX982500.1          ------------------------------------------------------
------ 0
    EU548051.1          ------------------------------------------------------
------ 0
    EU548044.1          ------------------------------------------------------
------ 0
    EU548042.1          ------------------------------------------------------
------ 0
    EU548043.1          ------------------------------------------------------
------ 0
    EU548047.1          ------------------------------------------------------
------ 0
    EU548050.1          ------------------------------------------------------
------ 0
    EU548048.1          ------------------------------------------------------
------ 0
    EU548049.1          ------------------------------------------------------
------ 0
    AF207722.1          ------------------------------------------------------
------ 0
    EU548037.1          ------------------------------------------------------
------ 0
    AF207723.1          ------------------------------------------------------
------ 0
    EU548038.1          ------------------------------------------------------
------ 0
    EU548036.1          ------------------------------------------------------
------ 0
    EU548035.1          ------------------------------------------------------
------ 0
    AF207720.1          ------------------------------------------------------
------ 0
    AB601576.1          ------------------------------------------------------
------ 0
    EU548040.1          ------------------------------------------------------
------ 0
    EU548039.1          ------------------------------------------------------
------ 0
    AF207721.1          ------------------------------------------------------
------ 0
    AF207724.1          ------------------------------------------------------
------ 0
    JX982499.1          ------------------------------------------------------
------ 0
    EU548041.1          ------------------------------------------------------
------ 0
    EU548045.1          ------------------------------------------------------
------ 0
    AF207725.1          ------------------------------------------------------
------ 0
    EU548046.1          ------------------------------------------------------
------ 0
    AF207714.1          ------------------------------------------------------
------ 0
    AF207713.1          ------------------------------------------------------
------ 0
    AF207712.1          ------------------------------------------------------
------ 0
```

```
    AY750628.1       ------------------------------------------------------
------ 1044
    EF689084.1       ------------------------------------------------------
------ 0
    EF689085.1       ------------------------------------------------------
------ 0
    AB119070.1       ------------------------------------------------------
------ 956
    EF987742.1       ------------------------------------------------------
------ 0
    AB026105.1       ------------------------------------------------------
------ 0
    MW148603.1
TCCTCTGTAAGCCACATAGCCCTAGTAATCATAGCTGTACTAATCCAATCACCATGAAGC   11100
    AB051263.1       ------------------------------------------------------
------ 0
    AF068544.1       ------------------------------------------------------
------ 0


    JX982502.1       ------------------------------------------------------
------ 0
    JX982501.1       ------------------------------------------------------
------ 0
    JX982498.1       ------------------------------------------------------
------ 0
    JX982497.1       ------------------------------------------------------
------ 0
    JX982495.1       ------------------------------------------------------
------ 0
    JX982496.1       ------------------------------------------------------
------ 0
    JX982500.1       ------------------------------------------------------
------ 0
    EU548051.1       ------------------------------------------------------
------ 0
    EU548044.1       ------------------------------------------------------
------ 0
    EU548042.1       ------------------------------------------------------
------ 0
    EU548043.1       ------------------------------------------------------
------ 0
    EU548047.1       ------------------------------------------------------
------ 0
    EU548050.1       ------------------------------------------------------
------ 0
    EU548048.1       ------------------------------------------------------
------ 0
    EU548049.1       ------------------------------------------------------
------ 0
    AF207722.1       ------------------------------------------------------
------ 0
    EU548037.1       ------------------------------------------------------
------ 0
    AF207723.1       ------------------------------------------------------
------ 0
    EU548038.1       ------------------------------------------------------
------ 0
    EU548036.1       ------------------------------------------------------
------ 0
    EU548035.1       ------------------------------------------------------
------ 0
    AF207720.1       ------------------------------------------------------
------ 0
    AB601576.1       ------------------------------------------------------
------ 0
```

```
    EU548040.1       ------------------------------------------------------
------ 0
    EU548039.1       ------------------------------------------------------
------ 0
    AF207721.1       ------------------------------------------------------
------ 0
    AF207724.1       ------------------------------------------------------
------ 0
    JX982499.1       ------------------------------------------------------
------ 0
    EU548041.1       ------------------------------------------------------
------ 0
    EU548045.1       ------------------------------------------------------
------ 0
    AF207725.1       ------------------------------------------------------
------ 0
    EU548046.1       ------------------------------------------------------
------ 0
    AF207714.1       ------------------------------------------------------
------ 0
    AF207713.1       ------------------------------------------------------
------ 0
    AF207712.1       ------------------------------------------------------
------ 0
    AY750628.1       ------------------------------------------------------
------ 1044
    EF689084.1       ------------------------------------------------------
------ 0
    EF689085.1       ------------------------------------------------------
------ 0
    AB119070.1       ------------------------------------------------------
------ 956
    EF987742.1       ------------------------------------------------------
------ 0
    AB026105.1       ------------------------------------------------------
------ 0
    MW148603.1
TATATAGGAGCAACAGCCCTAATAATTGCTCACGGTTTAACATCGTCCATGTTGTTCTGC   11160
    AB051263.1       ------------------------------------------------------
------ 0
    AF068544.1       ------------------------------------------------------
------ 0


    JX982502.1       ------------------------------------------------------
------ 0
    JX982501.1       ------------------------------------------------------
------ 0
    JX982498.1       ------------------------------------------------------
------ 0
    JX982497.1       ------------------------------------------------------
------ 0
    JX982495.1       ------------------------------------------------------
------ 0
    JX982496.1       ------------------------------------------------------
------ 0
    JX982500.1       ------------------------------------------------------
------ 0
    EU548051.1       ------------------------------------------------------
------ 0
    EU548044.1       ------------------------------------------------------
------ 0
    EU548042.1       ------------------------------------------------------
------ 0
    EU548043.1       ------------------------------------------------------
------ 0
```

EU548047.1 --
------ 0
EU548050.1 --
------ 0
EU548048.1 --
------ 0
EU548049.1 --
------ 0
AF207722.1 --
------ 0
EU548037.1 --
------ 0
AF207723.1 --
------ 0
EU548038.1 --
------ 0
EU548036.1 --
------ 0
EU548035.1 --
------ 0
AF207720.1 --
------ 0
AB601576.1 --
------ 0
EU548040.1 --
------ 0
EU548039.1 --
------ 0
AF207721.1 --
------ 0
AF207724.1 --
------ 0
JX982499.1 --
------ 0
EU548041.1 --
------ 0
EU548045.1 --
------ 0
AF207725.1 --
------ 0
EU548046.1 --
------ 0
AF207714.1 --
------ 0
AF207713.1 --
------ 0
AF207712.1 --
------ 0
AY750628.1 --
------ 1044
EF689084.1 --
------ 0
EF689085.1 --
------ 0
AB119070.1 --
------ 956
EF987742.1 --
------ 0
AB026105.1 --
------ 0
MW148603.1
CTAGCCAATTCCAACTACGAACGTATTCACAGCCGCACTATAATCCTCGCACGAGGATTA 11220
AB051263.1 --
------ 0
AF068544.1 --
------ 0

JX982502.1 ---
------ 0
JX982501.1 ---
------ 0
JX982498.1 ---
------ 0
JX982497.1 ---
------ 0
JX982495.1 ---
------ 0
JX982496.1 ---
------ 0
JX982500.1 ---
------ 0
EU548051.1 ---
------ 0
EU548044.1 ---
------ 0
EU548042.1 ---
------ 0
EU548043.1 ---
------ 0
EU548047.1 ---
------ 0
EU548050.1 ---
------ 0
EU548048.1 ---
------ 0
EU548049.1 ---
------ 0
AF207722.1 ---
------ 0
EU548037.1 ---
------ 0
AF207723.1 ---
------ 0
EU548038.1 ---
------ 0
EU548036.1 ---
------ 0
EU548035.1 ---
------ 0
AF207720.1 ---
------ 0
AB601576.1 ---
------ 0
EU548040.1 ---
------ 0
EU548039.1 ---
------ 0
AF207721.1 ---
------ 0
AF207724.1 ---
------ 0
JX982499.1 ---
------ 0
EU548041.1 ---
------ 0
EU548045.1 ---
------ 0
AF207725.1 ---
------ 0
EU548046.1 ---
------ 0

```
   AF207714.1      ------------------------------------------------------
------ 0
   AF207713.1      ------------------------------------------------------
------ 0
   AF207712.1      ------------------------------------------------------
------ 0
   AY750628.1      ------------------------------------------------------
------ 1044
   EF689084.1      ------------------------------------------------------
------ 0
   EF689085.1      ------------------------------------------------------
------ 0
   AB119070.1      ------------------------------------------------------
------ 956
   EF987742.1      ------------------------------------------------------
------ 0
   AB026105.1      ------------------------------------------------------
------ 0
   MW148603.1
CAAACACTCCTACCACTAATAGCTGCATGATGACTACTTGCCAGCTTAACTAATTTAGCT   11280
   AB051263.1      ------------------------------------------------------
------ 0
   AF068544.1      ------------------------------------------------------
------ 0


   JX982502.1      ------------------------------------------------------
------ 0
   JX982501.1      ------------------------------------------------------
------ 0
   JX982498.1      ------------------------------------------------------
------ 0
   JX982497.1      ------------------------------------------------------
------ 0
   JX982495.1      ------------------------------------------------------
------ 0
   JX982496.1      ------------------------------------------------------
------ 0
   JX982500.1      ------------------------------------------------------
------ 0
   EU548051.1      ------------------------------------------------------
------ 0
   EU548044.1      ------------------------------------------------------
------ 0
   EU548042.1      ------------------------------------------------------
------ 0
   EU548043.1      ------------------------------------------------------
------ 0
   EU548047.1      ------------------------------------------------------
------ 0
   EU548050.1      ------------------------------------------------------
------ 0
   EU548048.1      ------------------------------------------------------
------ 0
   EU548049.1      ------------------------------------------------------
------ 0
   AF207722.1      ------------------------------------------------------
------ 0
   EU548037.1      ------------------------------------------------------
------ 0
   AF207723.1      ------------------------------------------------------
------ 0
   EU548038.1      ------------------------------------------------------
------ 0
   EU548036.1      ------------------------------------------------------
------ 0
```

```
   EU548035.1      ------------------------------------------------------
------ 0
   AF207720.1      ------------------------------------------------------
------ 0
   AB601576.1      ------------------------------------------------------
------ 0
   EU548040.1      ------------------------------------------------------
------ 0
   EU548039.1      ------------------------------------------------------
------ 0
   AF207721.1      ------------------------------------------------------
------ 0
   AF207724.1      ------------------------------------------------------
------ 0
   JX982499.1      ------------------------------------------------------
------ 0
   EU548041.1      ------------------------------------------------------
------ 0
   EU548045.1      ------------------------------------------------------
------ 0
   AF207725.1      ------------------------------------------------------
------ 0
   EU548046.1      ------------------------------------------------------
------ 0
   AF207714.1      ------------------------------------------------------
------ 0
   AF207713.1      ------------------------------------------------------
------ 0
   AF207712.1      ------------------------------------------------------
------ 0
   AY750628.1      ------------------------------------------------------
------ 1044
   EF689084.1      ------------------------------------------------------
------ 0
   EF689085.1      ------------------------------------------------------
------ 0
   AB119070.1      ------------------------------------------------------
------ 956
   EF987742.1      ------------------------------------------------------
------ 0
   AB026105.1      ------------------------------------------------------
------ 0
   MW148603.1
TTACCACCCACAATCAACCTAGTAGGAGAGTTATTCGTAGTAATAGCCTCATTCTCATGA   11340
   AB051263.1      ------------------------------------------------------
------ 0
   AF068544.1      ------------------------------------------------------
------ 0


   JX982502.1      ------------------------------------------------------
------ 0
   JX982501.1      ------------------------------------------------------
------ 0
   JX982498.1      ------------------------------------------------------
------ 0
   JX982497.1      ------------------------------------------------------
------ 0
   JX982495.1      ------------------------------------------------------
------ 0
   JX982496.1      ------------------------------------------------------
------ 0
   JX982500.1      ------------------------------------------------------
------ 0
   EU548051.1      ------------------------------------------------------
------ 0
```

```
    EU548044.1          ------------------------------------------------------
------ 0
    EU548042.1          ------------------------------------------------------
------ 0
    EU548043.1          ------------------------------------------------------
------ 0
    EU548047.1          ------------------------------------------------------
------ 0
    EU548050.1          ------------------------------------------------------
------ 0
    EU548048.1          ------------------------------------------------------
------ 0
    EU548049.1          ------------------------------------------------------
------ 0
    AF207722.1          ------------------------------------------------------
------ 0
    EU548037.1          ------------------------------------------------------
------ 0
    AF207723.1          ------------------------------------------------------
------ 0
    EU548038.1          ------------------------------------------------------
------ 0
    EU548036.1          ------------------------------------------------------
------ 0
    EU548035.1          ------------------------------------------------------
------ 0
    AF207720.1          ------------------------------------------------------
------ 0
    AB601576.1          ------------------------------------------------------
------ 0
    EU548040.1          ------------------------------------------------------
------ 0
    EU548039.1          ------------------------------------------------------
------ 0
    AF207721.1          ------------------------------------------------------
------ 0
    AF207724.1          ------------------------------------------------------
------ 0
    JX982499.1          ------------------------------------------------------
------ 0
    EU548041.1          ------------------------------------------------------
------ 0
    EU548045.1          ------------------------------------------------------
------ 0
    AF207725.1          ------------------------------------------------------
------ 0
    EU548046.1          ------------------------------------------------------
------ 0
    AF207714.1          ------------------------------------------------------
------ 0
    AF207713.1          ------------------------------------------------------
------ 0
    AF207712.1          ------------------------------------------------------
------ 0
    AY750628.1          ------------------------------------------------------
------ 1044
    EF689084.1          ------------------------------------------------------
------ 0
    EF689085.1          ------------------------------------------------------
------ 0
    AB119070.1          ------------------------------------------------------
------ 956
    EF987742.1          ------------------------------------------------------
------ 0
    AB026105.1          ------------------------------------------------------
------ 0
```

MW148603.1
TCCAACATTACCATTGCCCTAATAGGAGTAAACATTACCATCACCGCCTTATACTCCTTA 11400
AB051263.1 --
------ 0
AF068544.1 --
------ 0

JX982502.1 --
------ 0
JX982501.1 --
------ 0
JX982498.1 --
------ 0
JX982497.1 --
------ 0
JX982495.1 --
------ 0
JX982496.1 --
------ 0
JX982500.1 --
------ 0
EU548051.1 --
------ 0
EU548044.1 --
------ 0
EU548042.1 --
------ 0
EU548043.1 --
------ 0
EU548047.1 --
------ 0
EU548050.1 --
------ 0
EU548048.1 --
------ 0
EU548049.1 --
------ 0
AF207722.1 --
------ 0
EU548037.1 --
------ 0
AF207723.1 --
------ 0
EU548038.1 --
------ 0
EU548036.1 --
------ 0
EU548035.1 --
------ 0
AF207720.1 --
------ 0
AB601576.1 --
------ 0
EU548040.1 --
------ 0
EU548039.1 --
------ 0
AF207721.1 --
------ 0
AF207724.1 --
------ 0
JX982499.1 --
------ 0
EU548041.1 --
------ 0

```
    EU548045.1        ------------------------------------------------------
------ 0
    AF207725.1        ------------------------------------------------------
------ 0
    EU548046.1        ------------------------------------------------------
------ 0
    AF207714.1        ------------------------------------------------------
------ 0
    AF207713.1        ------------------------------------------------------
------ 0
    AF207712.1        ------------------------------------------------------
------ 0
    AY750628.1        ------------------------------------------------------
------ 1044
    EF689084.1        ------------------------------------------------------
------ 0
    EF689085.1        ------------------------------------------------------
------ 0
    AB119070.1        ------------------------------------------------------
------ 956
    EF987742.1        ------------------------------------------------------
------ 0
    AB026105.1        ------------------------------------------------------
------ 0
    MW148603.1
TACATACTAATCACTACACAACGCGGAAAGTGTACGCATCACATCAAAAATATCAAACCA    11460
    AB051263.1        ------------------------------------------------------
------ 0
    AF068544.1        ------------------------------------------------------
------ 0


    JX982502.1        ------------------------------------------------------
------ 0
    JX982501.1        ------------------------------------------------------
------ 0
    JX982498.1        ------------------------------------------------------
------ 0
    JX982497.1        ------------------------------------------------------
------ 0
    JX982495.1        ------------------------------------------------------
------ 0
    JX982496.1        ------------------------------------------------------
------ 0
    JX982500.1        ------------------------------------------------------
------ 0
    EU548051.1        ------------------------------------------------------
------ 0
    EU548044.1        ------------------------------------------------------
------ 0
    EU548042.1        ------------------------------------------------------
------ 0
    EU548043.1        ------------------------------------------------------
------ 0
    EU548047.1        ------------------------------------------------------
------ 0
    EU548050.1        ------------------------------------------------------
------ 0
    EU548048.1        ------------------------------------------------------
------ 0
    EU548049.1        ------------------------------------------------------
------ 0
    AF207722.1        ------------------------------------------------------
------ 0
    EU548037.1        ------------------------------------------------------
------ 0
```

```
   AF207723.1      ------------------------------------------------------
------ 0
   EU548038.1      ------------------------------------------------------
------ 0
   EU548036.1      ------------------------------------------------------
------ 0
   EU548035.1      ------------------------------------------------------
------ 0
   AF207720.1      ------------------------------------------------------
------ 0
   AB601576.1      ------------------------------------------------------
------ 0
   EU548040.1      ------------------------------------------------------
------ 0
   EU548039.1      ------------------------------------------------------
------ 0
   AF207721.1      ------------------------------------------------------
------ 0
   AF207724.1      ------------------------------------------------------
------ 0
   JX982499.1      ------------------------------------------------------
------ 0
   EU548041.1      ------------------------------------------------------
------ 0
   EU548045.1      ------------------------------------------------------
------ 0
   AF207725.1      ------------------------------------------------------
------ 0
   EU548046.1      ------------------------------------------------------
------ 0
   AF207714.1      ------------------------------------------------------
------ 0
   AF207713.1      ------------------------------------------------------
------ 0
   AF207712.1      ------------------------------------------------------
------ 0
   AY750628.1      ------------------------------------------------------
------ 1044
   EF689084.1      ------------------------------------------------------
------ 0
   EF689085.1      ------------------------------------------------------
------ 0
   AB119070.1      ------------------------------------------------------
------ 956
   EF987742.1      ------------------------------------------------------
------ 0
   AB026105.1      ------------------------------------------------------
------ 0
   MW148603.1
TCTTCTACACGGGAAAATTCCCTTATAGCCCTCCACCTCCTACCTCTACTCCTCCTGTCA   11520
   AB051263.1      ------------------------------------------------------
------ 0
   AF068544.1      ------------------------------------------------------
------ 0


   JX982502.1      ------------------------------------------------------
------ 0
   JX982501.1      ------------------------------------------------------
------ 0
   JX982498.1      ------------------------------------------------------
------ 0
   JX982497.1      ------------------------------------------------------
------ 0
   JX982495.1      ------------------------------------------------------
------ 0
```

```
    JX982496.1      ------------------------------------------------------
------ 0
    JX982500.1      ------------------------------------------------------
------ 0
    EU548051.1      ------------------------------------------------------
------ 0
    EU548044.1      ------------------------------------------------------
------ 0
    EU548042.1      ------------------------------------------------------
------ 0
    EU548043.1      ------------------------------------------------------
------ 0
    EU548047.1      ------------------------------------------------------
------ 0
    EU548050.1      ------------------------------------------------------
------ 0
    EU548048.1      ------------------------------------------------------
------ 0
    EU548049.1      ------------------------------------------------------
------ 0
    AF207722.1      ------------------------------------------------------
------ 0
    EU548037.1      ------------------------------------------------------
------ 0
    AF207723.1      ------------------------------------------------------
------ 0
    EU548038.1      ------------------------------------------------------
------ 0
    EU548036.1      ------------------------------------------------------
------ 0
    EU548035.1      ------------------------------------------------------
------ 0
    AF207720.1      ------------------------------------------------------
------ 0
    AB601576.1      ------------------------------------------------------
------ 0
    EU548040.1      ------------------------------------------------------
------ 0
    EU548039.1      ------------------------------------------------------
------ 0
    AF207721.1      ------------------------------------------------------
------ 0
    AF207724.1      ------------------------------------------------------
------ 0
    JX982499.1      ------------------------------------------------------
------ 0
    EU548041.1      ------------------------------------------------------
------ 0
    EU548045.1      ------------------------------------------------------
------ 0
    AF207725.1      ------------------------------------------------------
------ 0
    EU548046.1      ------------------------------------------------------
------ 0
    AF207714.1      ------------------------------------------------------
------ 0
    AF207713.1      ------------------------------------------------------
------ 0
    AF207712.1      ------------------------------------------------------
------ 0
    AY750628.1      ------------------------------------------------------
------ 1044
    EF689084.1      ------------------------------------------------------
------ 0
    EF689085.1      ------------------------------------------------------
------ 0
```

```
   AB119070.1      ------------------------------------------------------
------ 956
   EF987742.1      ------------------------------------------------------
------ 0
   AB026105.1      ------------------------------------------------------
------ 0
   MW148603.1
CTCAACCCTAAAATTATCTTAGGGTACATTTACTGTAAATATAGTTTAATAAAAACATTA   11580
   AB051263.1      ------------------------------------------------------
------ 0
   AF068544.1      ------------------------------------------------------
------ 0


   JX982502.1      ------------------------------------------------------
------ 0
   JX982501.1      ------------------------------------------------------
------ 0
   JX982498.1      ------------------------------------------------------
------ 0
   JX982497.1      ------------------------------------------------------
------ 0
   JX982495.1      ------------------------------------------------------
------ 0
   JX982496.1      ------------------------------------------------------
------ 0
   JX982500.1      ------------------------------------------------------
------ 0
   EU548051.1      ------------------------------------------------------
------ 0
   EU548044.1      ------------------------------------------------------
------ 0
   EU548042.1      ------------------------------------------------------
------ 0
   EU548043.1      ------------------------------------------------------
------ 0
   EU548047.1      ------------------------------------------------------
------ 0
   EU548050.1      ------------------------------------------------------
------ 0
   EU548048.1      ------------------------------------------------------
------ 0
   EU548049.1      ------------------------------------------------------
------ 0
   AF207722.1      ------------------------------------------------------
------ 0
   EU548037.1      ------------------------------------------------------
------ 0
   AF207723.1      ------------------------------------------------------
------ 0
   EU548038.1      ------------------------------------------------------
------ 0
   EU548036.1      ------------------------------------------------------
------ 0
   EU548035.1      ------------------------------------------------------
------ 0
   AF207720.1      ------------------------------------------------------
------ 0
   AB601576.1      ------------------------------------------------------
------ 0
   EU548040.1      ------------------------------------------------------
------ 0
   EU548039.1      ------------------------------------------------------
------ 0
   AF207721.1      ------------------------------------------------------
------ 0
```

AF207724.1 --
------ 0
JX982499.1 --
------ 0
EU548041.1 --
------ 0
EU548045.1 --
------ 0
AF207725.1 --
------ 0
EU548046.1 --
------ 0
AF207714.1 --
------ 0
AF207713.1 --
------ 0
AF207712.1 --
------ 0
AY750628.1 --
------ 1044
EF689084.1 --
------ 0
EF689085.1 --
------ 0
AB119070.1 --
------ 956
EF987742.1 --
------ 0
AB026105.1 --
------ 0
MW148603.1
GATTGTGAATCTAACAATAAAAGCTCAAACCTTTTTATTTACCGAAAAAGCACCACGCAT 11640
AB051263.1 --
------ 0
AF068544.1 --
------ 0

JX982502.1 --
------ 0
JX982501.1 --
------ 0
JX982498.1 --
------ 0
JX982497.1 --
------ 0
JX982495.1 --
------ 0
JX982496.1 --
------ 0
JX982500.1 --
------ 0
EU548051.1 --
------ 0
EU548044.1 --
------ 0
EU548042.1 --
------ 0
EU548043.1 --
------ 0
EU548047.1 --
------ 0
EU548050.1 --
------ 0
EU548048.1 --
------ 0

EU548049.1 --
------ 0
AF207722.1 --
------ 0
EU548037.1 --
------ 0
AF207723.1 --
------ 0
EU548038.1 --
------ 0
EU548036.1 --
------ 0
EU548035.1 --
------ 0
AF207720.1 --
------ 0
AB601576.1 --
------ 0
EU548040.1 --
------ 0
EU548039.1 --
------ 0
AF207721.1 --
------ 0
AF207724.1 --
------ 0
JX982499.1 --
------ 0
EU548041.1 --
------ 0
EU548045.1 --
------ 0
AF207725.1 --
------ 0
EU548046.1 --
------ 0
AF207714.1 --
------ 0
AF207713.1 --
------ 0
AF207712.1 --
------ 0
AY750628.1 --
------ 1044
EF689084.1 --
------ 0
EF689085.1 --
------ 0
AB119070.1 --
------ 956
EF987742.1 --
------ 0
AB026105.1 --
------ 0
MW148603.1
GAACTGCTAACTCATGCTCCCGTGTATAAAAACACGGCTTTTTCAACTTTTAAAGGATAG 11700
AB051263.1 --
------ 0
AF068544.1 --
------ 0

JX982502.1 --
------ 0
JX982501.1 --
------ 0

```
    JX982498.1       ------------------------------------------------------
------ 0
    JX982497.1       ------------------------------------------------------
------ 0
    JX982495.1       ------------------------------------------------------
------ 0
    JX982496.1       ------------------------------------------------------
------ 0
    JX982500.1       ------------------------------------------------------
------ 0
    EU548051.1       ------------------------------------------------------
------ 0
    EU548044.1       ------------------------------------------------------
------ 0
    EU548042.1       ------------------------------------------------------
------ 0
    EU548043.1       ------------------------------------------------------
------ 0
    EU548047.1       ------------------------------------------------------
------ 0
    EU548050.1       ------------------------------------------------------
------ 0
    EU548048.1       ------------------------------------------------------
------ 0
    EU548049.1       ------------------------------------------------------
------ 0
    AF207722.1       ------------------------------------------------------
------ 0
    EU548037.1       ------------------------------------------------------
------ 0
    AF207723.1       ------------------------------------------------------
------ 0
    EU548038.1       ------------------------------------------------------
------ 0
    EU548036.1       ------------------------------------------------------
------ 0
    EU548035.1       ------------------------------------------------------
------ 0
    AF207720.1       ------------------------------------------------------
------ 0
    AB601576.1       ------------------------------------------------------
------ 0
    EU548040.1       ------------------------------------------------------
------ 0
    EU548039.1       ------------------------------------------------------
------ 0
    AF207721.1       ------------------------------------------------------
------ 0
    AF207724.1       ------------------------------------------------------
------ 0
    JX982499.1       ------------------------------------------------------
------ 0
    EU548041.1       ------------------------------------------------------
------ 0
    EU548045.1       ------------------------------------------------------
------ 0
    AF207725.1       ------------------------------------------------------
------ 0
    EU548046.1       ------------------------------------------------------
------ 0
    AF207714.1       ------------------------------------------------------
------ 0
    AF207713.1       ------------------------------------------------------
------ 0
    AF207712.1       ------------------------------------------------------
------ 0
```

```
    AY750628.1       ------------------------------------------------------
------ 1044
    EF689084.1       ------------------------------------------------------
------ 0
    EF689085.1       ------------------------------------------------------
------ 0
    AB119070.1       ------------------------------------------------------
------ 956
    EF987742.1       ------------------------------------------------------
------ 0
    AB026105.1       ------------------------------------------------------
------ 0
    MW148603.1
TAGTAATCCATTGGTCTTAGGAACCAAAAAATTGGTGCAACTCCAAATAAAAGTAATTAA   11760
    AB051263.1       ------------------------------------------------------
------ 0
    AF068544.1       ------------------------------------------------------
------ 0


    JX982502.1       ------------------------------------------------------
------ 0
    JX982501.1       ------------------------------------------------------
------ 0
    JX982498.1       ------------------------------------------------------
------ 0
    JX982497.1       ------------------------------------------------------
------ 0
    JX982495.1       ------------------------------------------------------
------ 0
    JX982496.1       ------------------------------------------------------
------ 0
    JX982500.1       ------------------------------------------------------
------ 0
    EU548051.1       ------------------------------------------------------
------ 0
    EU548044.1       ------------------------------------------------------
------ 0
    EU548042.1       ------------------------------------------------------
------ 0
    EU548043.1       ------------------------------------------------------
------ 0
    EU548047.1       ------------------------------------------------------
------ 0
    EU548050.1       ------------------------------------------------------
------ 0
    EU548048.1       ------------------------------------------------------
------ 0
    EU548049.1       ------------------------------------------------------
------ 0
    AF207722.1       ------------------------------------------------------
------ 0
    EU548037.1       ------------------------------------------------------
------ 0
    AF207723.1       ------------------------------------------------------
------ 0
    EU548038.1       ------------------------------------------------------
------ 0
    EU548036.1       ------------------------------------------------------
------ 0
    EU548035.1       ------------------------------------------------------
------ 0
    AF207720.1       ------------------------------------------------------
------ 0
    AB601576.1       ------------------------------------------------------
------ 0
```

EU548040.1 --
------ 0
EU548039.1 --
------ 0
AF207721.1 --
------ 0
AF207724.1 --
------ 0
JX982499.1 --
------ 0
EU548041.1 --
------ 0
EU548045.1 --
------ 0
AF207725.1 --
------ 0
EU548046.1 --
------ 0
AF207714.1 --
------ 0
AF207713.1 --
------ 0
AF207712.1 --
------ 0
AY750628.1 --
------ 1044
EF689084.1 --
------ 0
EF689085.1 --
------ 0
AB119070.1 --
------ 956
EF987742.1 --
------ 0
AB026105.1 --
------ 0
MW148603.1
CTTACTCACTTCCTCTGTACTTGTGACACTACTAATACTTACTTTCCCCATCATAATATC 11820
AB051263.1 --
------ 0
AF068544.1 --
------ 0

JX982502.1 --
------ 0
JX982501.1 --
------ 0
JX982498.1 --
------ 0
JX982497.1 --
------ 0
JX982495.1 --
------ 0
JX982496.1 --
------ 0
JX982500.1 --
------ 0
EU548051.1 --
------ 0
EU548044.1 --
------ 0
EU548042.1 --
------ 0
EU548043.1 --
------ 0

EU548047.1 --
------ 0
EU548050.1 --
------ 0
EU548048.1 --
------ 0
EU548049.1 --
------ 0
AF207722.1 --
------ 0
EU548037.1 --
------ 0
AF207723.1 --
------ 0
EU548038.1 --
------ 0
EU548036.1 --
------ 0
EU548035.1 --
------ 0
AF207720.1 --
------ 0
AB601576.1 --
------ 0
EU548040.1 --
------ 0
EU548039.1 --
------ 0
AF207721.1 --
------ 0
AF207724.1 --
------ 0
JX982499.1 --
------ 0
EU548041.1 --
------ 0
EU548045.1 --
------ 0
AF207725.1 --
------ 0
EU548046.1 --
------ 0
AF207714.1 --
------ 0
AF207713.1 --
------ 0
AF207712.1 --
------ 0
AY750628.1 --
------ 1044
EF689084.1 --
------ 0
EF689085.1 --
------ 0
AB119070.1 --
------ 956
EF987742.1 --
------ 0
AB026105.1 --
------ 0
MW148603.1
TAGCACGACTATATACGCCAACAAATCATACCCTCAATACGTAAAAACCGCTACTTCATA 11880
AB051263.1 --
------ 0
AF068544.1 --
------ 0

```
    JX982502.1         ------------------------------------------------------
------ 0
    JX982501.1         ------------------------------------------------------
------ 0
    JX982498.1         ------------------------------------------------------
------ 0
    JX982497.1         ------------------------------------------------------
------ 0
    JX982495.1         ------------------------------------------------------
------ 0
    JX982496.1         ------------------------------------------------------
------ 0
    JX982500.1         ------------------------------------------------------
------ 0
    EU548051.1         ------------------------------------------------------
------ 0
    EU548044.1         ------------------------------------------------------
------ 0
    EU548042.1         ------------------------------------------------------
------ 0
    EU548043.1         ------------------------------------------------------
------ 0
    EU548047.1         ------------------------------------------------------
------ 0
    EU548050.1         ------------------------------------------------------
------ 0
    EU548048.1         ------------------------------------------------------
------ 0
    EU548049.1         ------------------------------------------------------
------ 0
    AF207722.1         ------------------------------------------------------
------ 0
    EU548037.1         ------------------------------------------------------
------ 0
    AF207723.1         ------------------------------------------------------
------ 0
    EU548038.1         ------------------------------------------------------
------ 0
    EU548036.1         ------------------------------------------------------
------ 0
    EU548035.1         ------------------------------------------------------
------ 0
    AF207720.1         ------------------------------------------------------
------ 0
    AB601576.1         ------------------------------------------------------
------ 0
    EU548040.1         ------------------------------------------------------
------ 0
    EU548039.1         ------------------------------------------------------
------ 0
    AF207721.1         ------------------------------------------------------
------ 0
    AF207724.1         ------------------------------------------------------
------ 0
    JX982499.1         ------------------------------------------------------
------ 0
    EU548041.1         ------------------------------------------------------
------ 0
    EU548045.1         ------------------------------------------------------
------ 0
    AF207725.1         ------------------------------------------------------
------ 0
    EU548046.1         ------------------------------------------------------
------ 0
```

```
    AF207714.1        ------------------------------------------------------
------ 0
    AF207713.1        ------------------------------------------------------
------ 0
    AF207712.1        ------------------------------------------------------
------ 0
    AY750628.1        ------------------------------------------------------
------ 1044
    EF689084.1        ------------------------------------------------------
------ 0
    EF689085.1        ------------------------------------------------------
------ 0
    AB119070.1        ------------------------------------------------------
------ 956
    EF987742.1        ------------------------------------------------------
------ 0
    AB026105.1        ------------------------------------------------------
------ 0
    MW148603.1
CGCCTTCATGATCAGCTTAATTCCCACAATGATATTTCTCCACCTTGGACAAGACACAAT   11940
    AB051263.1        ------------------------------------------------------
------ 0
    AF068544.1        ------------------------------------------------------
------ 0


    JX982502.1        ------------------------------------------------------
------ 0
    JX982501.1        ------------------------------------------------------
------ 0
    JX982498.1        ------------------------------------------------------
------ 0
    JX982497.1        ------------------------------------------------------
------ 0
    JX982495.1        ------------------------------------------------------
------ 0
    JX982496.1        ------------------------------------------------------
------ 0
    JX982500.1        ------------------------------------------------------
------ 0
    EU548051.1        ------------------------------------------------------
------ 0
    EU548044.1        ------------------------------------------------------
------ 0
    EU548042.1        ------------------------------------------------------
------ 0
    EU548043.1        ------------------------------------------------------
------ 0
    EU548047.1        ------------------------------------------------------
------ 0
    EU548050.1        ------------------------------------------------------
------ 0
    EU548048.1        ------------------------------------------------------
------ 0
    EU548049.1        ------------------------------------------------------
------ 0
    AF207722.1        ------------------------------------------------------
------ 0
    EU548037.1        ------------------------------------------------------
------ 0
    AF207723.1        ------------------------------------------------------
------ 0
    EU548038.1        ------------------------------------------------------
------ 0
    EU548036.1        ------------------------------------------------------
------ 0
```

```
   EU548035.1      ------------------------------------------------------
------ 0
   AF207720.1      ------------------------------------------------------
------ 0
   AB601576.1      ------------------------------------------------------
------ 0
   EU548040.1      ------------------------------------------------------
------ 0
   EU548039.1      ------------------------------------------------------
------ 0
   AF207721.1      ------------------------------------------------------
------ 0
   AF207724.1      ------------------------------------------------------
------ 0
   JX982499.1      ------------------------------------------------------
------ 0
   EU548041.1      ------------------------------------------------------
------ 0
   EU548045.1      ------------------------------------------------------
------ 0
   AF207725.1      ------------------------------------------------------
------ 0
   EU548046.1      ------------------------------------------------------
------ 0
   AF207714.1      ------------------------------------------------------
------ 0
   AF207713.1      ------------------------------------------------------
------ 0
   AF207712.1      ------------------------------------------------------
------ 0
   AY750628.1      ------------------------------------------------------
------ 1044
   EF689084.1      ------------------------------------------------------
------ 0
   EF689085.1      ------------------------------------------------------
------ 0
   AB119070.1      ------------------------------------------------------
------ 956
   EF987742.1      ------------------------------------------------------
------ 0
   AB026105.1      ------------------------------------------------------
------ 0
   MW148603.1
AATTTCAAACTGACACTGAATTACAATCCAAACAATAAAATTGTCACTCAGCTTTAAACT   12000
   AB051263.1      ------------------------------------------------------
------ 0
   AF068544.1      ------------------------------------------------------
------ 0


   JX982502.1      ------------------------------------------------------
------ 0
   JX982501.1      ------------------------------------------------------
------ 0
   JX982498.1      ------------------------------------------------------
------ 0
   JX982497.1      ------------------------------------------------------
------ 0
   JX982495.1      ------------------------------------------------------
------ 0
   JX982496.1      ------------------------------------------------------
------ 0
   JX982500.1      ------------------------------------------------------
------ 0
   EU548051.1      ------------------------------------------------------
------ 0
```

```
    EU548044.1        ------------------------------------------------------
------ 0
    EU548042.1        ------------------------------------------------------
------ 0
    EU548043.1        ------------------------------------------------------
------ 0
    EU548047.1        ------------------------------------------------------
------ 0
    EU548050.1        ------------------------------------------------------
------ 0
    EU548048.1        ------------------------------------------------------
------ 0
    EU548049.1        ------------------------------------------------------
------ 0
    AF207722.1        ------------------------------------------------------
------ 0
    EU548037.1        ------------------------------------------------------
------ 0
    AF207723.1        ------------------------------------------------------
------ 0
    EU548038.1        ------------------------------------------------------
------ 0
    EU548036.1        ------------------------------------------------------
------ 0
    EU548035.1        ------------------------------------------------------
------ 0
    AF207720.1        ------------------------------------------------------
------ 0
    AB601576.1        ------------------------------------------------------
------ 0
    EU548040.1        ------------------------------------------------------
------ 0
    EU548039.1        ------------------------------------------------------
------ 0
    AF207721.1        ------------------------------------------------------
------ 0
    AF207724.1        ------------------------------------------------------
------ 0
    JX982499.1        ------------------------------------------------------
------ 0
    EU548041.1        ------------------------------------------------------
------ 0
    EU548045.1        ------------------------------------------------------
------ 0
    AF207725.1        ------------------------------------------------------
------ 0
    EU548046.1        ------------------------------------------------------
------ 0
    AF207714.1        ------------------------------------------------------
------ 0
    AF207713.1        ------------------------------------------------------
------ 0
    AF207712.1        ------------------------------------------------------
------ 0
    AY750628.1        ------------------------------------------------------
------ 1044
    EF689084.1        ------------------------------------------------------
------ 0
    EF689085.1        ------------------------------------------------------
------ 0
    AB119070.1        ------------------------------------------------------
------ 956
    EF987742.1        ------------------------------------------------------
------ 0
    AB026105.1        ------------------------------------------------------
------ 0
```

MW148603.1
CGACTACTTCTCAATAATCTTCATACCAGTAGCACTACTCGTCACATGATCAATCATAGA 12060
AB051263.1 --
------ 0
AF068544.1 --
------ 0

JX982502.1 --
------ 0
JX982501.1 --
------ 0
JX982498.1 --
------ 0
JX982497.1 --
------ 0
JX982495.1 --
------ 0
JX982496.1 --
------ 0
JX982500.1 --
------ 0
EU548051.1 --
------ 0
EU548044.1 --
------ 0
EU548042.1 --
------ 0
EU548043.1 --
------ 0
EU548047.1 --
------ 0
EU548050.1 --
------ 0
EU548048.1 --
------ 0
EU548049.1 --
------ 0
AF207722.1 --
------ 0
EU548037.1 --
------ 0
AF207723.1 --
------ 0
EU548038.1 --
------ 0
EU548036.1 --
------ 0
EU548035.1 --
------ 0
AF207720.1 --
------ 0
AB601576.1 --
------ 0
EU548040.1 --
------ 0
EU548039.1 --
------ 0
AF207721.1 --
------ 0
AF207724.1 --
------ 0
JX982499.1 --
------ 0
EU548041.1 --
------ 0

EU548045.1 --
------ 0
AF207725.1 --
------ 0
EU548046.1 --
------ 0
AF207714.1 --
------ 0
AF207713.1 --
------ 0
AF207712.1 --
------ 0
AY750628.1 --
------ 1044
EF689084.1 --
------ 0
EF689085.1 --
------ 0
AB119070.1 --
------ 956
EF987742.1 --
------ 0
AB026105.1 --
------ 0
MW148603.1
ATTCTCTATATGGTACATACACTCAGACCCCAACATCAATCGATTTTTCAAGTACTTACT 12120
AB051263.1 --
------ 0
AF068544.1 --
------ 0

JX982502.1 --
------ 0
JX982501.1 --
------ 0
JX982498.1 --
------ 0
JX982497.1 --
------ 0
JX982495.1 --
------ 0
JX982496.1 --
------ 0
JX982500.1 --
------ 0
EU548051.1 --
------ 0
EU548044.1 --
------ 0
EU548042.1 --
------ 0
EU548043.1 --
------ 0
EU548047.1 --
------ 0
EU548050.1 --
------ 0
EU548048.1 --
------ 0
EU548049.1 --
------ 0
AF207722.1 --
------ 0
EU548037.1 --
------ 0

```
   AF207723.1      ------------------------------------------------------
------ 0
   EU548038.1      ------------------------------------------------------
------ 0
   EU548036.1      ------------------------------------------------------
------ 0
   EU548035.1      ------------------------------------------------------
------ 0
   AF207720.1      ------------------------------------------------------
------ 0
   AB601576.1      ------------------------------------------------------
------ 0
   EU548040.1      ------------------------------------------------------
------ 0
   EU548039.1      ------------------------------------------------------
------ 0
   AF207721.1      ------------------------------------------------------
------ 0
   AF207724.1      ------------------------------------------------------
------ 0
   JX982499.1      ------------------------------------------------------
------ 0
   EU548041.1      ------------------------------------------------------
------ 0
   EU548045.1      ------------------------------------------------------
------ 0
   AF207725.1      ------------------------------------------------------
------ 0
   EU548046.1      ------------------------------------------------------
------ 0
   AF207714.1      ------------------------------------------------------
------ 0
   AF207713.1      ------------------------------------------------------
------ 0
   AF207712.1      ------------------------------------------------------
------ 0
   AY750628.1      ------------------------------------------------------
------ 1044
   EF689084.1      ------------------------------------------------------
------ 0
   EF689085.1      ------------------------------------------------------
------ 0
   AB119070.1      ------------------------------------------------------
------ 956
   EF987742.1      ------------------------------------------------------
------ 0
   AB026105.1      ------------------------------------------------------
------ 0
   MW148603.1
TCTATTTCTCATTACTATAATAATTCTGGTCACTGCCAACAACATATTCCAACTATTTAT   12180
   AB051263.1      ------------------------------------------------------
------ 0
   AF068544.1      ------------------------------------------------------
------ 0


   JX982502.1      ------------------------------------------------------
------ 0
   JX982501.1      ------------------------------------------------------
------ 0
   JX982498.1      ------------------------------------------------------
------ 0
   JX982497.1      ------------------------------------------------------
------ 0
   JX982495.1      ------------------------------------------------------
------ 0
```

```
    JX982496.1        -------------------------------------------------------
------ 0
    JX982500.1        -------------------------------------------------------
------ 0
    EU548051.1        -------------------------------------------------------
------ 0
    EU548044.1        -------------------------------------------------------
------ 0
    EU548042.1        -------------------------------------------------------
------ 0
    EU548043.1        -------------------------------------------------------
------ 0
    EU548047.1        -------------------------------------------------------
------ 0
    EU548050.1        -------------------------------------------------------
------ 0
    EU548048.1        -------------------------------------------------------
------ 0
    EU548049.1        -------------------------------------------------------
------ 0
    AF207722.1        -------------------------------------------------------
------ 0
    EU548037.1        -------------------------------------------------------
------ 0
    AF207723.1        -------------------------------------------------------
------ 0
    EU548038.1        -------------------------------------------------------
------ 0
    EU548036.1        -------------------------------------------------------
------ 0
    EU548035.1        -------------------------------------------------------
------ 0
    AF207720.1        -------------------------------------------------------
------ 0
    AB601576.1        -------------------------------------------------------
------ 0
    EU548040.1        -------------------------------------------------------
------ 0
    EU548039.1        -------------------------------------------------------
------ 0
    AF207721.1        -------------------------------------------------------
------ 0
    AF207724.1        -------------------------------------------------------
------ 0
    JX982499.1        -------------------------------------------------------
------ 0
    EU548041.1        -------------------------------------------------------
------ 0
    EU548045.1        -------------------------------------------------------
------ 0
    AF207725.1        -------------------------------------------------------
------ 0
    EU548046.1        -------------------------------------------------------
------ 0
    AF207714.1        -------------------------------------------------------
------ 0
    AF207713.1        -------------------------------------------------------
------ 0
    AF207712.1        -------------------------------------------------------
------ 0
    AY750628.1        -------------------------------------------------------
------ 1044
    EF689084.1        -------------------------------------------------------
------ 0
    EF689085.1        -------------------------------------------------------
------ 0
```

AB119070.1 --
------ 956
EF987742.1 --
------ 0
AB026105.1 --
------ 0
MW148603.1
CGGCTGAGAAGGAGTAGGAATTATATCATTCCTACTTATCGGATGATGATACGGACGAAC 12240
AB051263.1 --
------ 0
AF068544.1 --
------ 0

JX982502.1 --
------ 0
JX982501.1 --
------ 0
JX982498.1 --
------ 0
JX982497.1 --
------ 0
JX982495.1 --
------ 0
JX982496.1 --
------ 0
JX982500.1 --
------ 0
EU548051.1 --
------ 0
EU548044.1 --
------ 0
EU548042.1 --
------ 0
EU548043.1 --
------ 0
EU548047.1 --
------ 0
EU548050.1 --
------ 0
EU548048.1 --
------ 0
EU548049.1 --
------ 0
AF207722.1 --
------ 0
EU548037.1 --
------ 0
AF207723.1 --
------ 0
EU548038.1 --
------ 0
EU548036.1 --
------ 0
EU548035.1 --
------ 0
AF207720.1 --
------ 0
AB601576.1 --
------ 0
EU548040.1 --
------ 0
EU548039.1 --
------ 0
AF207721.1 --
------ 0

```
    AF207724.1       ------------------------------------------------------
------ 0
    JX982499.1       ------------------------------------------------------
------ 0
    EU548041.1       ------------------------------------------------------
------ 0
    EU548045.1       ------------------------------------------------------
------ 0
    AF207725.1       ------------------------------------------------------
------ 0
    EU548046.1       ------------------------------------------------------
------ 0
    AF207714.1       ------------------------------------------------------
------ 0
    AF207713.1       ------------------------------------------------------
------ 0
    AF207712.1       ------------------------------------------------------
------ 0
    AY750628.1       ------------------------------------------------------
------ 1044
    EF689084.1       ------------------------------------------------------
------ 0
    EF689085.1       ------------------------------------------------------
------ 0
    AB119070.1       ------------------------------------------------------
------ 956
    EF987742.1       ------------------------------------------------------
------ 0
    AB026105.1       ------------------------------------------------------
------ 0
    MW148603.1
AGACGCCAACACAGCAGCACTGCAGGCCATCCTATATAACCGCATTGGAGACGTAGGACT   12300
    AB051263.1       ------------------------------------------------------
------ 0
    AF068544.1       ------------------------------------------------------
------ 0


    JX982502.1       ------------------------------------------------------
------ 0
    JX982501.1       ------------------------------------------------------
------ 0
    JX982498.1       ------------------------------------------------------
------ 0
    JX982497.1       ------------------------------------------------------
------ 0
    JX982495.1       ------------------------------------------------------
------ 0
    JX982496.1       ------------------------------------------------------
------ 0
    JX982500.1       ------------------------------------------------------
------ 0
    EU548051.1       ------------------------------------------------------
------ 0
    EU548044.1       ------------------------------------------------------
------ 0
    EU548042.1       ------------------------------------------------------
------ 0
    EU548043.1       ------------------------------------------------------
------ 0
    EU548047.1       ------------------------------------------------------
------ 0
    EU548050.1       ------------------------------------------------------
------ 0
    EU548048.1       ------------------------------------------------------
------ 0
```

```
    EU548049.1      ------------------------------------------------------
------ 0
    AF207722.1      ------------------------------------------------------
------ 0
    EU548037.1      ------------------------------------------------------
------ 0
    AF207723.1      ------------------------------------------------------
------ 0
    EU548038.1      ------------------------------------------------------
------ 0
    EU548036.1      ------------------------------------------------------
------ 0
    EU548035.1      ------------------------------------------------------
------ 0
    AF207720.1      ------------------------------------------------------
------ 0
    AB601576.1      ------------------------------------------------------
------ 0
    EU548040.1      ------------------------------------------------------
------ 0
    EU548039.1      ------------------------------------------------------
------ 0
    AF207721.1      ------------------------------------------------------
------ 0
    AF207724.1      ------------------------------------------------------
------ 0
    JX982499.1      ------------------------------------------------------
------ 0
    EU548041.1      ------------------------------------------------------
------ 0
    EU548045.1      ------------------------------------------------------
------ 0
    AF207725.1      ------------------------------------------------------
------ 0
    EU548046.1      ------------------------------------------------------
------ 0
    AF207714.1      ------------------------------------------------------
------ 0
    AF207713.1      ------------------------------------------------------
------ 0
    AF207712.1      ------------------------------------------------------
------ 0
    AY750628.1      ------------------------------------------------------
------ 1044
    EF689084.1      ------------------------------------------------------
------ 0
    EF689085.1      ------------------------------------------------------
------ 0
    AB119070.1      ------------------------------------------------------
------ 956
    EF987742.1      ------------------------------------------------------
------ 0
    AB026105.1      ------------------------------------------------------
------ 0
    MW148603.1
TATCCTAGCCATAGCCTGATTCTTGATAAATCTAAATACATGAGACCTTCAACAAATCTT   12360
    AB051263.1      ------------------------------------------------------
------ 0
    AF068544.1      ------------------------------------------------------
------ 0


    JX982502.1      ------------------------------------------------------
------ 0
    JX982501.1      ------------------------------------------------------
------ 0
```

```
    JX982498.1          ------------------------------------------------------
------ 0
    JX982497.1          ------------------------------------------------------
------ 0
    JX982495.1          ------------------------------------------------------
------ 0
    JX982496.1          ------------------------------------------------------
------ 0
    JX982500.1          ------------------------------------------------------
------ 0
    EU548051.1          ------------------------------------------------------
------ 0
    EU548044.1          ------------------------------------------------------
------ 0
    EU548042.1          ------------------------------------------------------
------ 0
    EU548043.1          ------------------------------------------------------
------ 0
    EU548047.1          ------------------------------------------------------
------ 0
    EU548050.1          ------------------------------------------------------
------ 0
    EU548048.1          ------------------------------------------------------
------ 0
    EU548049.1          ------------------------------------------------------
------ 0
    AF207722.1          ------------------------------------------------------
------ 0
    EU548037.1          ------------------------------------------------------
------ 0
    AF207723.1          ------------------------------------------------------
------ 0
    EU548038.1          ------------------------------------------------------
------ 0
    EU548036.1          ------------------------------------------------------
------ 0
    EU548035.1          ------------------------------------------------------
------ 0
    AF207720.1          ------------------------------------------------------
------ 0
    AB601576.1          ------------------------------------------------------
------ 0
    EU548040.1          ------------------------------------------------------
------ 0
    EU548039.1          ------------------------------------------------------
------ 0
    AF207721.1          ------------------------------------------------------
------ 0
    AF207724.1          ------------------------------------------------------
------ 0
    JX982499.1          ------------------------------------------------------
------ 0
    EU548041.1          ------------------------------------------------------
------ 0
    EU548045.1          ------------------------------------------------------
------ 0
    AF207725.1          ------------------------------------------------------
------ 0
    EU548046.1          ------------------------------------------------------
------ 0
    AF207714.1          ------------------------------------------------------
------ 0
    AF207713.1          ------------------------------------------------------
------ 0
    AF207712.1          ------------------------------------------------------
------ 0
```

```
   AY750628.1      ------------------------------------------------------
------ 1044
   EF689084.1      ------------------------------------------------------
------ 0
   EF689085.1      ------------------------------------------------------
------ 0
   AB119070.1      ------------------------------------------------------
------ 956
   EF987742.1      ------------------------------------------------------
------ 0
   AB026105.1      ------------------------------------------------------
------ 0
   MW148603.1
CATAACCAGCAACGAAAACCTAACTATCCCCCTCGCAGGCTTACTACTAGCAGCCACCGG   12420
   AB051263.1      ------------------------------------------------------
------ 0
   AF068544.1      ------------------------------------------------------
------ 0


   JX982502.1      ------------------------------------------------------
------ 0
   JX982501.1      ------------------------------------------------------
------ 0
   JX982498.1      ------------------------------------------------------
------ 0
   JX982497.1      ------------------------------------------------------
------ 0
   JX982495.1      ------------------------------------------------------
------ 0
   JX982496.1      ------------------------------------------------------
------ 0
   JX982500.1      ------------------------------------------------------
------ 0
   EU548051.1      ------------------------------------------------------
------ 0
   EU548044.1      ------------------------------------------------------
------ 0
   EU548042.1      ------------------------------------------------------
------ 0
   EU548043.1      ------------------------------------------------------
------ 0
   EU548047.1      ------------------------------------------------------
------ 0
   EU548050.1      ------------------------------------------------------
------ 0
   EU548048.1      ------------------------------------------------------
------ 0
   EU548049.1      ------------------------------------------------------
------ 0
   AF207722.1      ------------------------------------------------------
------ 0
   EU548037.1      ------------------------------------------------------
------ 0
   AF207723.1      ------------------------------------------------------
------ 0
   EU548038.1      ------------------------------------------------------
------ 0
   EU548036.1      ------------------------------------------------------
------ 0
   EU548035.1      ------------------------------------------------------
------ 0
   AF207720.1      ------------------------------------------------------
------ 0
   AB601576.1      ------------------------------------------------------
------ 0
```

EU548040.1 --
------ 0
EU548039.1 --
------ 0
AF207721.1 --
------ 0
AF207724.1 --
------ 0
JX982499.1 --
------ 0
EU548041.1 --
------ 0
EU548045.1 --
------ 0
AF207725.1 --
------ 0
EU548046.1 --
------ 0
AF207714.1 --
------ 0
AF207713.1 --
------ 0
AF207712.1 --
------ 0
AY750628.1 --
------ 1044
EF689084.1 --
------ 0
EF689085.1 --
------ 0
AB119070.1 --
------ 956
EF987742.1 --
------ 0
AB026105.1 --
------ 0
MW148603.1
AAAATCCGCACAATTCGGTCTTCACCCGTGACTGCCCTCAGCCATAGAAGGACCAACCCC 12480
AB051263.1 --
------ 0
AF068544.1 --
------ 0

JX982502.1 --
------ 0
JX982501.1 --
------ 0
JX982498.1 --
------ 0
JX982497.1 --
------ 0
JX982495.1 --
------ 0
JX982496.1 --
------ 0
JX982500.1 --
------ 0
EU548051.1 --
------ 0
EU548044.1 --
------ 0
EU548042.1 --
------ 0
EU548043.1 --
------ 0

```
    EU548047.1      ------------------------------------------------------
------ 0
    EU548050.1      ------------------------------------------------------
------ 0
    EU548048.1      ------------------------------------------------------
------ 0
    EU548049.1      ------------------------------------------------------
------ 0
    AF207722.1      ------------------------------------------------------
------ 0
    EU548037.1      ------------------------------------------------------
------ 0
    AF207723.1      ------------------------------------------------------
------ 0
    EU548038.1      ------------------------------------------------------
------ 0
    EU548036.1      ------------------------------------------------------
------ 0
    EU548035.1      ------------------------------------------------------
------ 0
    AF207720.1      ------------------------------------------------------
------ 0
    AB601576.1      ------------------------------------------------------
------ 0
    EU548040.1      ------------------------------------------------------
------ 0
    EU548039.1      ------------------------------------------------------
------ 0
    AF207721.1      ------------------------------------------------------
------ 0
    AF207724.1      ------------------------------------------------------
------ 0
    JX982499.1      ------------------------------------------------------
------ 0
    EU548041.1      ------------------------------------------------------
------ 0
    EU548045.1      ------------------------------------------------------
------ 0
    AF207725.1      ------------------------------------------------------
------ 0
    EU548046.1      ------------------------------------------------------
------ 0
    AF207714.1      ------------------------------------------------------
------ 0
    AF207713.1      ------------------------------------------------------
------ 0
    AF207712.1      ------------------------------------------------------
------ 0
    AY750628.1      ------------------------------------------------------
------ 1044
    EF689084.1      ------------------------------------------------------
------ 0
    EF689085.1      ------------------------------------------------------
------ 0
    AB119070.1      ------------------------------------------------------
------ 956
    EF987742.1      ------------------------------------------------------
------ 0
    AB026105.1      ------------------------------------------------------
------ 0
    MW148603.1
CGTATCAGCCCTACTTCACTCAAGTACAATAGTTGTAGCAGGGATTTTTCTACTAATCCG   12540
    AB051263.1      ------------------------------------------------------
------ 0
    AF068544.1      ------------------------------------------------------
------ 0
```

JX982502.1 --
------ 0
JX982501.1 --
------ 0
JX982498.1 --
------ 0
JX982497.1 --
------ 0
JX982495.1 --
------ 0
JX982496.1 --
------ 0
JX982500.1 --
------ 0
EU548051.1 --
------ 0
EU548044.1 --
------ 0
EU548042.1 --
------ 0
EU548043.1 --
------ 0
EU548047.1 --
------ 0
EU548050.1 --
------ 0
EU548048.1 --
------ 0
EU548049.1 --
------ 0
AF207722.1 --
------ 0
EU548037.1 --
------ 0
AF207723.1 --
------ 0
EU548038.1 --
------ 0
EU548036.1 --
------ 0
EU548035.1 --
------ 0
AF207720.1 --
------ 0
AB601576.1 --
------ 0
EU548040.1 --
------ 0
EU548039.1 --
------ 0
AF207721.1 --
------ 0
AF207724.1 --
------ 0
JX982499.1 --
------ 0
EU548041.1 --
------ 0
EU548045.1 --
------ 0
AF207725.1 --
------ 0
EU548046.1 --
------ 0

AF207714.1 --
------ 0
AF207713.1 --
------ 0
AF207712.1 --
------ 0
AY750628.1 --
------ 1044
EF689084.1 --
------ 0
EF689085.1 --
------ 0
AB119070.1 --
------ 956
EF987742.1 --
------ 0
AB026105.1 --
------ 0
MW148603.1
ATTCCACCCCCTCATAGAGTATAATAAAACGATCCAAACCATCACACTATGCCTAGGAGC 12600
AB051263.1 --
------ 0
AF068544.1 --
------ 0

JX982502.1 --
------ 0
JX982501.1 --
------ 0
JX982498.1 --
------ 0
JX982497.1 --
------ 0
JX982495.1 --
------ 0
JX982496.1 --
------ 0
JX982500.1 --
------ 0
EU548051.1 --
------ 0
EU548044.1 --
------ 0
EU548042.1 --
------ 0
EU548043.1 --
------ 0
EU548047.1 --
------ 0
EU548050.1 --
------ 0
EU548048.1 --
------ 0
EU548049.1 --
------ 0
AF207722.1 --
------ 0
EU548037.1 --
------ 0
AF207723.1 --
------ 0
EU548038.1 --
------ 0
EU548036.1 --
------ 0

```
   EU548035.1      ------------------------------------------------------
------ 0
   AF207720.1      ------------------------------------------------------
------ 0
   AB601576.1      ------------------------------------------------------
------ 0
   EU548040.1      ------------------------------------------------------
------ 0
   EU548039.1      ------------------------------------------------------
------ 0
   AF207721.1      ------------------------------------------------------
------ 0
   AF207724.1      ------------------------------------------------------
------ 0
   JX982499.1      ------------------------------------------------------
------ 0
   EU548041.1      ------------------------------------------------------
------ 0
   EU548045.1      ------------------------------------------------------
------ 0
   AF207725.1      ------------------------------------------------------
------ 0
   EU548046.1      ------------------------------------------------------
------ 0
   AF207714.1      ------------------------------------------------------
------ 0
   AF207713.1      ------------------------------------------------------
------ 0
   AF207712.1      ------------------------------------------------------
------ 0
   AY750628.1      ------------------------------------------------------
------ 1044
   EF689084.1      ------------------------------------------------------
------ 0
   EF689085.1      ------------------------------------------------------
------ 0
   AB119070.1      ------------------------------------------------------
------ 956
   EF987742.1      ------------------------------------------------------
------ 0
   AB026105.1      ------------------------------------------------------
------ 0
   MW148603.1
AATTACAACCCTATTTACAGCAATCTGTGCTCTAACACAAAATGACATCAAAAAAATCGT   12660
   AB051263.1      ------------------------------------------------------
------ 0
   AF068544.1      ------------------------------------------------------
------ 0


   JX982502.1      ------------------------------------------------------
------ 0
   JX982501.1      ------------------------------------------------------
------ 0
   JX982498.1      ------------------------------------------------------
------ 0
   JX982497.1      ------------------------------------------------------
------ 0
   JX982495.1      ------------------------------------------------------
------ 0
   JX982496.1      ------------------------------------------------------
------ 0
   JX982500.1      ------------------------------------------------------
------ 0
   EU548051.1      ------------------------------------------------------
------ 0
```

```
    EU548044.1      ------------------------------------------------------
------ 0
    EU548042.1      ------------------------------------------------------
------ 0
    EU548043.1      ------------------------------------------------------
------ 0
    EU548047.1      ------------------------------------------------------
------ 0
    EU548050.1      ------------------------------------------------------
------ 0
    EU548048.1      ------------------------------------------------------
------ 0
    EU548049.1      ------------------------------------------------------
------ 0
    AF207722.1      ------------------------------------------------------
------ 0
    EU548037.1      ------------------------------------------------------
------ 0
    AF207723.1      ------------------------------------------------------
------ 0
    EU548038.1      ------------------------------------------------------
------ 0
    EU548036.1      ------------------------------------------------------
------ 0
    EU548035.1      ------------------------------------------------------
------ 0
    AF207720.1      ------------------------------------------------------
------ 0
    AB601576.1      ------------------------------------------------------
------ 0
    EU548040.1      ------------------------------------------------------
------ 0
    EU548039.1      ------------------------------------------------------
------ 0
    AF207721.1      ------------------------------------------------------
------ 0
    AF207724.1      ------------------------------------------------------
------ 0
    JX982499.1      ------------------------------------------------------
------ 0
    EU548041.1      ------------------------------------------------------
------ 0
    EU548045.1      ------------------------------------------------------
------ 0
    AF207725.1      ------------------------------------------------------
------ 0
    EU548046.1      ------------------------------------------------------
------ 0
    AF207714.1      ------------------------------------------------------
------ 0
    AF207713.1      ------------------------------------------------------
------ 0
    AF207712.1      ------------------------------------------------------
------ 0
    AY750628.1      ------------------------------------------------------
------ 1044
    EF689084.1      ------------------------------------------------------
------ 0
    EF689085.1      ------------------------------------------------------
------ 0
    AB119070.1      ------------------------------------------------------
------ 956
    EF987742.1      ------------------------------------------------------
------ 0
    AB026105.1      ------------------------------------------------------
------ 0
```

```
    MW148603.1
TGCCTTTTCCACCTCTAGCCAACTCGGATTAATAATAGTAACCATCGGAATTAACCAACC   12720
    AB051263.1      ------------------------------------------------------
------ 0
    AF068544.1      ------------------------------------------------------
------ 0


    JX982502.1      ------------------------------------------------------
------ 0
    JX982501.1      ------------------------------------------------------
------ 0
    JX982498.1      ------------------------------------------------------
------ 0
    JX982497.1      ------------------------------------------------------
------ 0
    JX982495.1      ------------------------------------------------------
------ 0
    JX982496.1      ------------------------------------------------------
------ 0
    JX982500.1      ------------------------------------------------------
------ 0
    EU548051.1      ------------------------------------------------------
------ 0
    EU548044.1      ------------------------------------------------------
------ 0
    EU548042.1      ------------------------------------------------------
------ 0
    EU548043.1      ------------------------------------------------------
------ 0
    EU548047.1      ------------------------------------------------------
------ 0
    EU548050.1      ------------------------------------------------------
------ 0
    EU548048.1      ------------------------------------------------------
------ 0
    EU548049.1      ------------------------------------------------------
------ 0
    AF207722.1      ------------------------------------------------------
------ 0
    EU548037.1      ------------------------------------------------------
------ 0
    AF207723.1      ------------------------------------------------------
------ 0
    EU548038.1      ------------------------------------------------------
------ 0
    EU548036.1      ------------------------------------------------------
------ 0
    EU548035.1      ------------------------------------------------------
------ 0
    AF207720.1      ------------------------------------------------------
------ 0
    AB601576.1      ------------------------------------------------------
------ 0
    EU548040.1      ------------------------------------------------------
------ 0
    EU548039.1      ------------------------------------------------------
------ 0
    AF207721.1      ------------------------------------------------------
------ 0
    AF207724.1      ------------------------------------------------------
------ 0
    JX982499.1      ------------------------------------------------------
------ 0
    EU548041.1      ------------------------------------------------------
------ 0
```

```
    EU548045.1       ------------------------------------------------------
------ 0
    AF207725.1       ------------------------------------------------------
------ 0
    EU548046.1       ------------------------------------------------------
------ 0
    AF207714.1       ------------------------------------------------------
------ 0
    AF207713.1       ------------------------------------------------------
------ 0
    AF207712.1       ------------------------------------------------------
------ 0
    AY750628.1       ------------------------------------------------------
------ 1044
    EF689084.1       ------------------------------------------------------
------ 0
    EF689085.1       ------------------------------------------------------
------ 0
    AB119070.1       ------------------------------------------------------
------ 956
    EF987742.1       ------------------------------------------------------
------ 0
    AB026105.1       ------------------------------------------------------
------ 0
    MW148603.1
CTACCTAGCATTCCTCCATATCTGCACCCACGCATTCTTCAAGGCTATACTATTCATATG   12780
    AB051263.1       ------------------------------------------------------
------ 0
    AF068544.1       ------------------------------------------------------
------ 0


    JX982502.1       ------------------------------------------------------
------ 0
    JX982501.1       ------------------------------------------------------
------ 0
    JX982498.1       ------------------------------------------------------
------ 0
    JX982497.1       ------------------------------------------------------
------ 0
    JX982495.1       ------------------------------------------------------
------ 0
    JX982496.1       ------------------------------------------------------
------ 0
    JX982500.1       ------------------------------------------------------
------ 0
    EU548051.1       ------------------------------------------------------
------ 0
    EU548044.1       ------------------------------------------------------
------ 0
    EU548042.1       ------------------------------------------------------
------ 0
    EU548043.1       ------------------------------------------------------
------ 0
    EU548047.1       ------------------------------------------------------
------ 0
    EU548050.1       ------------------------------------------------------
------ 0
    EU548048.1       ------------------------------------------------------
------ 0
    EU548049.1       ------------------------------------------------------
------ 0
    AF207722.1       ------------------------------------------------------
------ 0
    EU548037.1       ------------------------------------------------------
------ 0
```

```
   AF207723.1      ------------------------------------------------------
------ 0
   EU548038.1      ------------------------------------------------------
------ 0
   EU548036.1      ------------------------------------------------------
------ 0
   EU548035.1      ------------------------------------------------------
------ 0
   AF207720.1      ------------------------------------------------------
------ 0
   AB601576.1      ------------------------------------------------------
------ 0
   EU548040.1      ------------------------------------------------------
------ 0
   EU548039.1      ------------------------------------------------------
------ 0
   AF207721.1      ------------------------------------------------------
------ 0
   AF207724.1      ------------------------------------------------------
------ 0
   JX982499.1      ------------------------------------------------------
------ 0
   EU548041.1      ------------------------------------------------------
------ 0
   EU548045.1      ------------------------------------------------------
------ 0
   AF207725.1      ------------------------------------------------------
------ 0
   EU548046.1      ------------------------------------------------------
------ 0
   AF207714.1      ------------------------------------------------------
------ 0
   AF207713.1      ------------------------------------------------------
------ 0
   AF207712.1      ------------------------------------------------------
------ 0
   AY750628.1      ------------------------------------------------------
------ 1044
   EF689084.1      ------------------------------------------------------
------ 0
   EF689085.1      ------------------------------------------------------
------ 0
   AB119070.1      ------------------------------------------------------
------ 956
   EF987742.1      ------------------------------------------------------
------ 0
   AB026105.1      ------------------------------------------------------
------ 0
   MW148603.1
CTCCGGATCAATCATCCACAGCCTAAACGATGAACAAGACATCCGAAAAATAGGAGGGTT   12840
   AB051263.1      ------------------------------------------------------
------ 0
   AF068544.1      ------------------------------------------------------
------ 0


   JX982502.1      ------------------------------------------------------
------ 0
   JX982501.1      ------------------------------------------------------
------ 0
   JX982498.1      ------------------------------------------------------
------ 0
   JX982497.1      ------------------------------------------------------
------ 0
   JX982495.1      ------------------------------------------------------
------ 0
```

```
    JX982496.1      ------------------------------------------------------
------ 0
    JX982500.1      ------------------------------------------------------
------ 0
    EU548051.1      ------------------------------------------------------
------ 0
    EU548044.1      ------------------------------------------------------
------ 0
    EU548042.1      ------------------------------------------------------
------ 0
    EU548043.1      ------------------------------------------------------
------ 0
    EU548047.1      ------------------------------------------------------
------ 0
    EU548050.1      ------------------------------------------------------
------ 0
    EU548048.1      ------------------------------------------------------
------ 0
    EU548049.1      ------------------------------------------------------
------ 0
    AF207722.1      ------------------------------------------------------
------ 0
    EU548037.1      ------------------------------------------------------
------ 0
    AF207723.1      ------------------------------------------------------
------ 0
    EU548038.1      ------------------------------------------------------
------ 0
    EU548036.1      ------------------------------------------------------
------ 0
    EU548035.1      ------------------------------------------------------
------ 0
    AF207720.1      ------------------------------------------------------
------ 0
    AB601576.1      ------------------------------------------------------
------ 0
    EU548040.1      ------------------------------------------------------
------ 0
    EU548039.1      ------------------------------------------------------
------ 0
    AF207721.1      ------------------------------------------------------
------ 0
    AF207724.1      ------------------------------------------------------
------ 0
    JX982499.1      ------------------------------------------------------
------ 0
    EU548041.1      ------------------------------------------------------
------ 0
    EU548045.1      ------------------------------------------------------
------ 0
    AF207725.1      ------------------------------------------------------
------ 0
    EU548046.1      ------------------------------------------------------
------ 0
    AF207714.1      ------------------------------------------------------
------ 0
    AF207713.1      ------------------------------------------------------
------ 0
    AF207712.1      ------------------------------------------------------
------ 0
    AY750628.1      ------------------------------------------------------
------ 1044
    EF689084.1      ------------------------------------------------------
------ 0
    EF689085.1      ------------------------------------------------------
------ 0
```

```
   AB119070.1      ------------------------------------------------------
------ 956
   EF987742.1      ------------------------------------------------------
------ 0
   AB026105.1      ------------------------------------------------------
------ 0
   MW148603.1
ATTTAAAGCATTACCATTCACCACAACCTCACTAATTGTCGGAAGCCTAGCACTTACAGG   12900
   AB051263.1      ------------------------------------------------------
------ 0
   AF068544.1      ------------------------------------------------------
------ 0


   JX982502.1      ------------------------------------------------------
------ 0
   JX982501.1      ------------------------------------------------------
------ 0
   JX982498.1      ------------------------------------------------------
------ 0
   JX982497.1      ------------------------------------------------------
------ 0
   JX982495.1      ------------------------------------------------------
------ 0
   JX982496.1      ------------------------------------------------------
------ 0
   JX982500.1      ------------------------------------------------------
------ 0
   EU548051.1      ------------------------------------------------------
------ 0
   EU548044.1      ------------------------------------------------------
------ 0
   EU548042.1      ------------------------------------------------------
------ 0
   EU548043.1      ------------------------------------------------------
------ 0
   EU548047.1      ------------------------------------------------------
------ 0
   EU548050.1      ------------------------------------------------------
------ 0
   EU548048.1      ------------------------------------------------------
------ 0
   EU548049.1      ------------------------------------------------------
------ 0
   AF207722.1      ------------------------------------------------------
------ 0
   EU548037.1      ------------------------------------------------------
------ 0
   AF207723.1      ------------------------------------------------------
------ 0
   EU548038.1      ------------------------------------------------------
------ 0
   EU548036.1      ------------------------------------------------------
------ 0
   EU548035.1      ------------------------------------------------------
------ 0
   AF207720.1      ------------------------------------------------------
------ 0
   AB601576.1      ------------------------------------------------------
------ 0
   EU548040.1      ------------------------------------------------------
------ 0
   EU548039.1      ------------------------------------------------------
------ 0
   AF207721.1      ------------------------------------------------------
------ 0
```

AF207724.1 --
------ 0
JX982499.1 --
------ 0
EU548041.1 --
------ 0
EU548045.1 --
------ 0
AF207725.1 --
------ 0
EU548046.1 --
------ 0
AF207714.1 --
------ 0
AF207713.1 --
------ 0
AF207712.1 --
------ 0
AY750628.1 --
------ 1044
EF689084.1 --
------ 0
EF689085.1 --
------ 0
AB119070.1 --
------ 956
EF987742.1 --
------ 0
AB026105.1 --
------ 0
MW148603.1
AATACCCTTCTTAACGGGATTTTATTCCAAAGACCTAATCATTGAGACCGCCAACACGTC 12960
AB051263.1 --
------ 0
AF068544.1 --
------ 0

JX982502.1 --
------ 0
JX982501.1 --
------ 0
JX982498.1 --
------ 0
JX982497.1 --
------ 0
JX982495.1 --
------ 0
JX982496.1 --
------ 0
JX982500.1 --
------ 0
EU548051.1 --
------ 0
EU548044.1 --
------ 0
EU548042.1 --
------ 0
EU548043.1 --
------ 0
EU548047.1 --
------ 0
EU548050.1 --
------ 0
EU548048.1 --
------ 0

```
    EU548049.1       ------------------------------------------------------
------ 0
    AF207722.1       ------------------------------------------------------
------ 0
    EU548037.1       ------------------------------------------------------
------ 0
    AF207723.1       ------------------------------------------------------
------ 0
    EU548038.1       ------------------------------------------------------
------ 0
    EU548036.1       ------------------------------------------------------
------ 0
    EU548035.1       ------------------------------------------------------
------ 0
    AF207720.1       ------------------------------------------------------
------ 0
    AB601576.1       ------------------------------------------------------
------ 0
    EU548040.1       ------------------------------------------------------
------ 0
    EU548039.1       ------------------------------------------------------
------ 0
    AF207721.1       ------------------------------------------------------
------ 0
    AF207724.1       ------------------------------------------------------
------ 0
    JX982499.1       ------------------------------------------------------
------ 0
    EU548041.1       ------------------------------------------------------
------ 0
    EU548045.1       ------------------------------------------------------
------ 0
    AF207725.1       ------------------------------------------------------
------ 0
    EU548046.1       ------------------------------------------------------
------ 0
    AF207714.1       ------------------------------------------------------
------ 0
    AF207713.1       ------------------------------------------------------
------ 0
    AF207712.1       ------------------------------------------------------
------ 0
    AY750628.1       ------------------------------------------------------
------ 1044
    EF689084.1       ------------------------------------------------------
------ 0
    EF689085.1       ------------------------------------------------------
------ 0
    AB119070.1       ------------------------------------------------------
------ 956
    EF987742.1       ------------------------------------------------------
------ 0
    AB026105.1       ------------------------------------------------------
------ 0
    MW148603.1
GTGTACCAACGCCTGAGCCCTTCTACTAACCCTCGTTGCCACTTCCATAACAGCAGCCTA   13020
    AB051263.1       ------------------------------------------------------
------ 0
    AF068544.1       ------------------------------------------------------
------ 0


    JX982502.1       ------------------------------------------------------
------ 0
    JX982501.1       ------------------------------------------------------
------ 0
```

```
    JX982498.1       ------------------------------------------------------------
------ 0
    JX982497.1       ------------------------------------------------------------
------ 0
    JX982495.1       ------------------------------------------------------------
------ 0
    JX982496.1       ------------------------------------------------------------
------ 0
    JX982500.1       ------------------------------------------------------------
------ 0
    EU548051.1       ------------------------------------------------------------
------ 0
    EU548044.1       ------------------------------------------------------------
------ 0
    EU548042.1       ------------------------------------------------------------
------ 0
    EU548043.1       ------------------------------------------------------------
------ 0
    EU548047.1       ------------------------------------------------------------
------ 0
    EU548050.1       ------------------------------------------------------------
------ 0
    EU548048.1       ------------------------------------------------------------
------ 0
    EU548049.1       ------------------------------------------------------------
------ 0
    AF207722.1       ------------------------------------------------------------
------ 0
    EU548037.1       ------------------------------------------------------------
------ 0
    AF207723.1       ------------------------------------------------------------
------ 0
    EU548038.1       ------------------------------------------------------------
------ 0
    EU548036.1       ------------------------------------------------------------
------ 0
    EU548035.1       ------------------------------------------------------------
------ 0
    AF207720.1       ------------------------------------------------------------
------ 0
    AB601576.1       ------------------------------------------------------------
------ 0
    EU548040.1       ------------------------------------------------------------
------ 0
    EU548039.1       ------------------------------------------------------------
------ 0
    AF207721.1       ------------------------------------------------------------
------ 0
    AF207724.1       ------------------------------------------------------------
------ 0
    JX982499.1       ------------------------------------------------------------
------ 0
    EU548041.1       ------------------------------------------------------------
------ 0
    EU548045.1       ------------------------------------------------------------
------ 0
    AF207725.1       ------------------------------------------------------------
------ 0
    EU548046.1       ------------------------------------------------------------
------ 0
    AF207714.1       ------------------------------------------------------------
------ 0
    AF207713.1       ------------------------------------------------------------
------ 0
    AF207712.1       ------------------------------------------------------------
------ 0
```

```
    AY750628.1      ------------------------------------------------------
------ 1044
    EF689084.1      ------------------------------------------------------
------ 0
    EF689085.1      ------------------------------------------------------
------ 0
    AB119070.1      ------------------------------------------------------
------ 956
    EF987742.1      ------------------------------------------------------
------ 0
    AB026105.1      ------------------------------------------------------
------ 0
    MW148603.1
CAGTACTCGAATCATATTCTTTGCACTACTAGGACAACCCCGCTTCAACCCTATTATTAC   13080
    AB051263.1      ------------------------------------------------------
------ 0
    AF068544.1      ------------------------------------------------------
------ 0


    JX982502.1      ------------------------------------------------------
------ 0
    JX982501.1      ------------------------------------------------------
------ 0
    JX982498.1      ------------------------------------------------------
------ 0
    JX982497.1      ------------------------------------------------------
------ 0
    JX982495.1      ------------------------------------------------------
------ 0
    JX982496.1      ------------------------------------------------------
------ 0
    JX982500.1      ------------------------------------------------------
------ 0
    EU548051.1      ------------------------------------------------------
------ 0
    EU548044.1      ------------------------------------------------------
------ 0
    EU548042.1      ------------------------------------------------------
------ 0
    EU548043.1      ------------------------------------------------------
------ 0
    EU548047.1      ------------------------------------------------------
------ 0
    EU548050.1      ------------------------------------------------------
------ 0
    EU548048.1      ------------------------------------------------------
------ 0
    EU548049.1      ------------------------------------------------------
------ 0
    AF207722.1      ------------------------------------------------------
------ 0
    EU548037.1      ------------------------------------------------------
------ 0
    AF207723.1      ------------------------------------------------------
------ 0
    EU548038.1      ------------------------------------------------------
------ 0
    EU548036.1      ------------------------------------------------------
------ 0
    EU548035.1      ------------------------------------------------------
------ 0
    AF207720.1      ------------------------------------------------------
------ 0
    AB601576.1      ------------------------------------------------------
------ 0
```

EU548040.1 --
------ 0
EU548039.1 --
------ 0
AF207721.1 --
------ 0
AF207724.1 --
------ 0
JX982499.1 --
------ 0
EU548041.1 --
------ 0
EU548045.1 --
------ 0
AF207725.1 --
------ 0
EU548046.1 --
------ 0
AF207714.1 --
------ 0
AF207713.1 --
------ 0
AF207712.1 --
------ 0
AY750628.1 --
------ 1044
EF689084.1 --
------ 0
EF689085.1 --
------ 0
AB119070.1 --
------ 956
EF987742.1 --
------ 0
AB026105.1 --
------ 0
MW148603.1
AATCAACGAGAATAATCCACTCCTAATCAACTCCATTAAACGCTTACTATTTGGGAGTAT 13140
AB051263.1 --
------ 0
AF068544.1 --
------ 0

JX982502.1 --
------ 0
JX982501.1 --
------ 0
JX982498.1 --
------ 0
JX982497.1 --
------ 0
JX982495.1 --
------ 0
JX982496.1 --
------ 0
JX982500.1 --
------ 0
EU548051.1 --
------ 0
EU548044.1 --
------ 0
EU548042.1 --
------ 0
EU548043.1 --
------ 0

```
    EU548047.1       ------------------------------------------------------
------ 0
    EU548050.1       ------------------------------------------------------
------ 0
    EU548048.1       ------------------------------------------------------
------ 0
    EU548049.1       ------------------------------------------------------
------ 0
    AF207722.1       ------------------------------------------------------
------ 0
    EU548037.1       ------------------------------------------------------
------ 0
    AF207723.1       ------------------------------------------------------
------ 0
    EU548038.1       ------------------------------------------------------
------ 0
    EU548036.1       ------------------------------------------------------
------ 0
    EU548035.1       ------------------------------------------------------
------ 0
    AF207720.1       ------------------------------------------------------
------ 0
    AB601576.1       ------------------------------------------------------
------ 0
    EU548040.1       ------------------------------------------------------
------ 0
    EU548039.1       ------------------------------------------------------
------ 0
    AF207721.1       ------------------------------------------------------
------ 0
    AF207724.1       ------------------------------------------------------
------ 0
    JX982499.1       ------------------------------------------------------
------ 0
    EU548041.1       ------------------------------------------------------
------ 0
    EU548045.1       ------------------------------------------------------
------ 0
    AF207725.1       ------------------------------------------------------
------ 0
    EU548046.1       ------------------------------------------------------
------ 0
    AF207714.1       ------------------------------------------------------
------ 0
    AF207713.1       ------------------------------------------------------
------ 0
    AF207712.1       ------------------------------------------------------
------ 0
    AY750628.1       ------------------------------------------------------
------ 1044
    EF689084.1       ------------------------------------------------------
------ 0
    EF689085.1       ------------------------------------------------------
------ 0
    AB119070.1       ------------------------------------------------------
------ 956
    EF987742.1       ------------------------------------------------------
------ 0
    AB026105.1       ------------------------------------------------------
------ 0
    MW148603.1
CTTCGCAGGATTCTTAATTTCCCACAACCTTACACCCACTACCACCCCACAGATAACTAT   13200
    AB051263.1       ------------------------------------------------------
------ 0
    AF068544.1       ------------------------------------------------------
------ 0
```

```
    JX982502.1        ------------------------------------------------------
------ 0
    JX982501.1        ------------------------------------------------------
------ 0
    JX982498.1        ------------------------------------------------------
------ 0
    JX982497.1        ------------------------------------------------------
------ 0
    JX982495.1        ------------------------------------------------------
------ 0
    JX982496.1        ------------------------------------------------------
------ 0
    JX982500.1        ------------------------------------------------------
------ 0
    EU548051.1        ------------------------------------------------------
------ 0
    EU548044.1        ------------------------------------------------------
------ 0
    EU548042.1        ------------------------------------------------------
------ 0
    EU548043.1        ------------------------------------------------------
------ 0
    EU548047.1        ------------------------------------------------------
------ 0
    EU548050.1        ------------------------------------------------------
------ 0
    EU548048.1        ------------------------------------------------------
------ 0
    EU548049.1        ------------------------------------------------------
------ 0
    AF207722.1        ------------------------------------------------------
------ 0
    EU548037.1        ------------------------------------------------------
------ 0
    AF207723.1        ------------------------------------------------------
------ 0
    EU548038.1        ------------------------------------------------------
------ 0
    EU548036.1        ------------------------------------------------------
------ 0
    EU548035.1        ------------------------------------------------------
------ 0
    AF207720.1        ------------------------------------------------------
------ 0
    AB601576.1        ------------------------------------------------------
------ 0
    EU548040.1        ------------------------------------------------------
------ 0
    EU548039.1        ------------------------------------------------------
------ 0
    AF207721.1        ------------------------------------------------------
------ 0
    AF207724.1        ------------------------------------------------------
------ 0
    JX982499.1        ------------------------------------------------------
------ 0
    EU548041.1        ------------------------------------------------------
------ 0
    EU548045.1        ------------------------------------------------------
------ 0
    AF207725.1        ------------------------------------------------------
------ 0
    EU548046.1        ------------------------------------------------------
------ 0
```

AF207714.1 --
------ 0
AF207713.1 --
------ 0
AF207712.1 --
------ 0
AY750628.1 --
------ 1044
EF689084.1 --
------ 0
EF689085.1 --
------ 0
AB119070.1 --
------ 956
EF987742.1 --
------ 0
AB026105.1 --
------ 0
MW148603.1
GCCTTATTATCTTAAAATAATGGCTCTTGCTGTAACTATTCTGGGTTTCATCCTGGCACT 13260
AB051263.1 --
------ 0
AF068544.1 --
------ 0

JX982502.1 --
------ 0
JX982501.1 --
------ 0
JX982498.1 --
------ 0
JX982497.1 --
------ 0
JX982495.1 --
------ 0
JX982496.1 --
------ 0
JX982500.1 --
------ 0
EU548051.1 --
------ 0
EU548044.1 --
------ 0
EU548042.1 --
------ 0
EU548043.1 --
------ 0
EU548047.1 --
------ 0
EU548050.1 --
------ 0
EU548048.1 --
------ 0
EU548049.1 --
------ 0
AF207722.1 --
------ 0
EU548037.1 --
------ 0
AF207723.1 --
------ 0
EU548038.1 --
------ 0
EU548036.1 --
------ 0

```
   EU548035.1      ------------------------------------------------------
------ 0
   AF207720.1      ------------------------------------------------------
------ 0
   AB601576.1      ------------------------------------------------------
------ 0
   EU548040.1      ------------------------------------------------------
------ 0
   EU548039.1      ------------------------------------------------------
------ 0
   AF207721.1      ------------------------------------------------------
------ 0
   AF207724.1      ------------------------------------------------------
------ 0
   JX982499.1      ------------------------------------------------------
------ 0
   EU548041.1      ------------------------------------------------------
------ 0
   EU548045.1      ------------------------------------------------------
------ 0
   AF207725.1      ------------------------------------------------------
------ 0
   EU548046.1      ------------------------------------------------------
------ 0
   AF207714.1      ------------------------------------------------------
------ 0
   AF207713.1      ------------------------------------------------------
------ 0
   AF207712.1      ------------------------------------------------------
------ 0
   AY750628.1      ------------------------------------------------------
------ 1044
   EF689084.1      ------------------------------------------------------
------ 0
   EF689085.1      ------------------------------------------------------
------ 0
   AB119070.1      ------------------------------------------------------
------ 956
   EF987742.1      ------------------------------------------------------
------ 0
   AB026105.1      ------------------------------------------------------
------ 0
   MW148603.1
AGAACTAAACCTTACGATGCAAAGCCTAAAATTCAAATATCCAACCAACCTATTCAAATT   13320
   AB051263.1      ------------------------------------------------------
------ 0
   AF068544.1      ------------------------------------------------------
------ 0


   JX982502.1      ------------------------------------------------------
------ 0
   JX982501.1      ------------------------------------------------------
------ 0
   JX982498.1      ------------------------------------------------------
------ 0
   JX982497.1      ------------------------------------------------------
------ 0
   JX982495.1      ------------------------------------------------------
------ 0
   JX982496.1      ------------------------------------------------------
------ 0
   JX982500.1      ------------------------------------------------------
------ 0
   EU548051.1      ------------------------------------------------------
------ 0
```

```
   EU548044.1        ------------------------------------------------------
------ 0
   EU548042.1        ------------------------------------------------------
------ 0
   EU548043.1        ------------------------------------------------------
------ 0
   EU548047.1        ------------------------------------------------------
------ 0
   EU548050.1        ------------------------------------------------------
------ 0
   EU548048.1        ------------------------------------------------------
------ 0
   EU548049.1        ------------------------------------------------------
------ 0
   AF207722.1        ------------------------------------------------------
------ 0
   EU548037.1        ------------------------------------------------------
------ 0
   AF207723.1        ------------------------------------------------------
------ 0
   EU548038.1        ------------------------------------------------------
------ 0
   EU548036.1        ------------------------------------------------------
------ 0
   EU548035.1        ------------------------------------------------------
------ 0
   AF207720.1        ------------------------------------------------------
------ 0
   AB601576.1        ------------------------------------------------------
------ 0
   EU548040.1        ------------------------------------------------------
------ 0
   EU548039.1        ------------------------------------------------------
------ 0
   AF207721.1        ------------------------------------------------------
------ 0
   AF207724.1        ------------------------------------------------------
------ 0
   JX982499.1        ------------------------------------------------------
------ 0
   EU548041.1        ------------------------------------------------------
------ 0
   EU548045.1        ------------------------------------------------------
------ 0
   AF207725.1        ------------------------------------------------------
------ 0
   EU548046.1        ------------------------------------------------------
------ 0
   AF207714.1        ------------------------------------------------------
------ 0
   AF207713.1        ------------------------------------------------------
------ 0
   AF207712.1        ------------------------------------------------------
------ 0
   AY750628.1        ------------------------------------------------------
------ 1044
   EF689084.1        ------------------------------------------------------
------ 0
   EF689085.1        ------------------------------------------------------
------ 0
   AB119070.1        ------------------------------------------------------
------ 956
   EF987742.1        ------------------------------------------------------
------ 0
   AB026105.1        ------------------------------------------------------
------ 0
```

MW148603.1
CTCAAACATGTTGGGTTATTTCCCTACCATTATTCACCGCCTAATGCCCAAAACAAATCT 13380
AB051263.1 --
------ 0
AF068544.1 --
------ 0

JX982502.1 --
------ 0
JX982501.1 --
------ 0
JX982498.1 --
------ 0
JX982497.1 --
------ 0
JX982495.1 --
------ 0
JX982496.1 --
------ 0
JX982500.1 --
------ 0
EU548051.1 --
------ 0
EU548044.1 --
------ 0
EU548042.1 --
------ 0
EU548043.1 --
------ 0
EU548047.1 --
------ 0
EU548050.1 --
------ 0
EU548048.1 --
------ 0
EU548049.1 --
------ 0
AF207722.1 --
------ 0
EU548037.1 --
------ 0
AF207723.1 --
------ 0
EU548038.1 --
------ 0
EU548036.1 --
------ 0
EU548035.1 --
------ 0
AF207720.1 --
------ 0
AB601576.1 --
------ 0
EU548040.1 --
------ 0
EU548039.1 --
------ 0
AF207721.1 --
------ 0
AF207724.1 --
------ 0
JX982499.1 --
------ 0
EU548041.1 --
------ 0

EU548045.1 --
------ 0
AF207725.1 --
------ 0
EU548046.1 --
------ 0
AF207714.1 --
------ 0
AF207713.1 --
------ 0
AF207712.1 --
------ 0
AY750628.1 --
------ 1044
EF689084.1 --
------ 0
EF689085.1 --
------ 0
AB119070.1 --
------ 956
EF987742.1 --
------ 0
AB026105.1 --
------ 0
MW148603.1
GCTAATAAGCCAAAAATCAGCATCAACACTTCTAGATATAATTTGACTAGAAAAAATTCT 13440
AB051263.1 --
------ 0
AF068544.1 --
------ 0

JX982502.1 --
------ 0
JX982501.1 --
------ 0
JX982498.1 --
------ 0
JX982497.1 --
------ 0
JX982495.1 --
------ 0
JX982496.1 --
------ 0
JX982500.1 --
------ 0
EU548051.1 --
------ 0
EU548044.1 --
------ 0
EU548042.1 --
------ 0
EU548043.1 --
------ 0
EU548047.1 --
------ 0
EU548050.1 --
------ 0
EU548048.1 --
------ 0
EU548049.1 --
------ 0
AF207722.1 --
------ 0
EU548037.1 --
------ 0

```
   AF207723.1      ------------------------------------------------------
------ 0
   EU548038.1      ------------------------------------------------------
------ 0
   EU548036.1      ------------------------------------------------------
------ 0
   EU548035.1      ------------------------------------------------------
------ 0
   AF207720.1      ------------------------------------------------------
------ 0
   AB601576.1      ------------------------------------------------------
------ 0
   EU548040.1      ------------------------------------------------------
------ 0
   EU548039.1      ------------------------------------------------------
------ 0
   AF207721.1      ------------------------------------------------------
------ 0
   AF207724.1      ------------------------------------------------------
------ 0
   JX982499.1      ------------------------------------------------------
------ 0
   EU548041.1      ------------------------------------------------------
------ 0
   EU548045.1      ------------------------------------------------------
------ 0
   AF207725.1      ------------------------------------------------------
------ 0
   EU548046.1      ------------------------------------------------------
------ 0
   AF207714.1      ------------------------------------------------------
------ 0
   AF207713.1      ------------------------------------------------------
------ 0
   AF207712.1      ------------------------------------------------------
------ 0
   AY750628.1      ------------------------------------------------------
------ 1044
   EF689084.1      ------------------------------------------------------
------ 0
   EF689085.1      ------------------------------------------------------
------ 0
   AB119070.1      ------------------------------------------------------
------ 956
   EF987742.1      ------------------------------------------------------
------ 0
   AB026105.1      ------------------------------------------------------
------ 0
   MW148603.1
ACCAAAATCCATCTCCCATTTCCAAATAAAATTATCAATAACCATCTCAAGCCAGAAAGG   13500
   AB051263.1      ------------------------------------------------------
------ 0
   AF068544.1      ------------------------------------------------------
------ 0


   JX982502.1      ------------------------------------------------------
------ 0
   JX982501.1      ------------------------------------------------------
------ 0
   JX982498.1      ------------------------------------------------------
------ 0
   JX982497.1      ------------------------------------------------------
------ 0
   JX982495.1      ------------------------------------------------------
------ 0
```

```
    JX982496.1      ------------------------------------------------------
------ 0
    JX982500.1      ------------------------------------------------------
------ 0
    EU548051.1      ------------------------------------------------------
------ 0
    EU548044.1      ------------------------------------------------------
------ 0
    EU548042.1      ------------------------------------------------------
------ 0
    EU548043.1      ------------------------------------------------------
------ 0
    EU548047.1      ------------------------------------------------------
------ 0
    EU548050.1      ------------------------------------------------------
------ 0
    EU548048.1      ------------------------------------------------------
------ 0
    EU548049.1      ------------------------------------------------------
------ 0
    AF207722.1      ------------------------------------------------------
------ 0
    EU548037.1      ------------------------------------------------------
------ 0
    AF207723.1      ------------------------------------------------------
------ 0
    EU548038.1      ------------------------------------------------------
------ 0
    EU548036.1      ------------------------------------------------------
------ 0
    EU548035.1      ------------------------------------------------------
------ 0
    AF207720.1      ------------------------------------------------------
------ 0
    AB601576.1      ------------------------------------------------------
------ 0
    EU548040.1      ------------------------------------------------------
------ 0
    EU548039.1      ------------------------------------------------------
------ 0
    AF207721.1      ------------------------------------------------------
------ 0
    AF207724.1      ------------------------------------------------------
------ 0
    JX982499.1      ------------------------------------------------------
------ 0
    EU548041.1      ------------------------------------------------------
------ 0
    EU548045.1      ------------------------------------------------------
------ 0
    AF207725.1      ------------------------------------------------------
------ 0
    EU548046.1      ------------------------------------------------------
------ 0
    AF207714.1      ------------------------------------------------------
------ 0
    AF207713.1      ------------------------------------------------------
------ 0
    AF207712.1      ------------------------------------------------------
------ 0
    AY750628.1      ------------------------------------------------------
------ 1044
    EF689084.1      ------------------------------------------------------
------ 0
    EF689085.1      ------------------------------------------------------
------ 0
```

```
   AB119070.1      ------------------------------------------------------
------ 956
   EF987742.1      ------------------------------------------------------
------ 0
   AB026105.1      ------------------------------------------------------
------ 0
   MW148603.1
CCTAATCAAACTATACTTTATATCGTTCATATTAACACTAACCCTCAGCCTGCTAACACT   13560
   AB051263.1      ------------------------------------------------------
------ 0
   AF068544.1      ------------------------------------------------------
------ 0


   JX982502.1      ------------------------------------------------------
------ 0
   JX982501.1      ------------------------------------------------------
------ 0
   JX982498.1      ------------------------------------------------------
------ 0
   JX982497.1      ------------------------------------------------------
------ 0
   JX982495.1      ------------------------------------------------------
------ 0
   JX982496.1      ------------------------------------------------------
------ 0
   JX982500.1      ------------------------------------------------------
------ 0
   EU548051.1      ------------------------------------------------------
------ 0
   EU548044.1      ------------------------------------------------------
------ 0
   EU548042.1      ------------------------------------------------------
------ 0
   EU548043.1      ------------------------------------------------------
------ 0
   EU548047.1      ------------------------------------------------------
------ 0
   EU548050.1      ------------------------------------------------------
------ 0
   EU548048.1      ------------------------------------------------------
------ 0
   EU548049.1      ------------------------------------------------------
------ 0
   AF207722.1      ------------------------------------------------------
------ 0
   EU548037.1      ------------------------------------------------------
------ 0
   AF207723.1      ------------------------------------------------------
------ 0
   EU548038.1      ------------------------------------------------------
------ 0
   EU548036.1      ------------------------------------------------------
------ 0
   EU548035.1      ------------------------------------------------------
------ 0
   AF207720.1      ------------------------------------------------------
------ 0
   AB601576.1      ------------------------------------------------------
------ 0
   EU548040.1      ------------------------------------------------------
------ 0
   EU548039.1      ------------------------------------------------------
------ 0
   AF207721.1      ------------------------------------------------------
------ 0
```

```
    AF207724.1       ------------------------------------------------------
------ 0
    JX982499.1       ------------------------------------------------------
------ 0
    EU548041.1       ------------------------------------------------------
------ 0
    EU548045.1       ------------------------------------------------------
------ 0
    AF207725.1       ------------------------------------------------------
------ 0
    EU548046.1       ------------------------------------------------------
------ 0
    AF207714.1       ------------------------------------------------------
------ 0
    AF207713.1       ------------------------------------------------------
------ 0
    AF207712.1       ------------------------------------------------------
------ 0
    AY750628.1       ------------------------------------------------------
------ 1044
    EF689084.1       ------------------------------------------------------
------ 0
    EF689085.1       ------------------------------------------------------
------ 0
    AB119070.1       ------------------------------------------------------
------ 956
    EF987742.1       ------------------------------------------------------
------ 0
    AB026105.1       ------------------------------------------------------
------ 0
    MW148603.1
TAATTTCCACGAGTAACCTCCATAATCACCAAGACTCCAATAAAAAGAGACCATCCCGTA   13620
    AB051263.1       ------------------------------------------------------
------ 0
    AF068544.1       ------------------------------------------------------
------ 0


    JX982502.1       ------------------------------------------------------
------ 0
    JX982501.1       ------------------------------------------------------
------ 0
    JX982498.1       ------------------------------------------------------
------ 0
    JX982497.1       ------------------------------------------------------
------ 0
    JX982495.1       ------------------------------------------------------
------ 0
    JX982496.1       ------------------------------------------------------
------ 0
    JX982500.1       ------------------------------------------------------
------ 0
    EU548051.1       ------------------------------------------------------
------ 0
    EU548044.1       ------------------------------------------------------
------ 0
    EU548042.1       ------------------------------------------------------
------ 0
    EU548043.1       ------------------------------------------------------
------ 0
    EU548047.1       ------------------------------------------------------
------ 0
    EU548050.1       ------------------------------------------------------
------ 0
    EU548048.1       ------------------------------------------------------
------ 0
```

EU548049.1 --
------ 0
AF207722.1 --
------ 0
EU548037.1 --
------ 0
AF207723.1 --
------ 0
EU548038.1 --
------ 0
EU548036.1 --
------ 0
EU548035.1 --
------ 0
AF207720.1 --
------ 0
AB601576.1 --
------ 0
EU548040.1 --
------ 0
EU548039.1 --
------ 0
AF207721.1 --
------ 0
AF207724.1 --
------ 0
JX982499.1 --
------ 0
EU548041.1 --
------ 0
EU548045.1 --
------ 0
AF207725.1 --
------ 0
EU548046.1 --
------ 0
AF207714.1 --
------ 0
AF207713.1 --
------ 0
AF207712.1 --
------ 0
AY750628.1 --
------ 1044
EF689084.1 --
------ 0
EF689085.1 --
------ 0
AB119070.1 --
------ 956
EF987742.1 --
------ 0
AB026105.1 --
------ 0
MW148603.1
ACAATTACAAGTCAAGTCCCATAACTATACAGAGCCGCAATTCCTATAGCTTCTTCACTG 13680
AB051263.1 --
------ 0
AF068544.1 --
------ 0

JX982502.1 --
------ 0
JX982501.1 --
------ 0

```
    JX982498.1          ------------------------------------------------------
------ 0
    JX982497.1          ------------------------------------------------------
------ 0
    JX982495.1          ------------------------------------------------------
------ 0
    JX982496.1          ------------------------------------------------------
------ 0
    JX982500.1          ------------------------------------------------------
------ 0
    EU548051.1          ------------------------------------------------------
------ 0
    EU548044.1          ------------------------------------------------------
------ 0
    EU548042.1          ------------------------------------------------------
------ 0
    EU548043.1          ------------------------------------------------------
------ 0
    EU548047.1          ------------------------------------------------------
------ 0
    EU548050.1          ------------------------------------------------------
------ 0
    EU548048.1          ------------------------------------------------------
------ 0
    EU548049.1          ------------------------------------------------------
------ 0
    AF207722.1          ------------------------------------------------------
------ 0
    EU548037.1          ------------------------------------------------------
------ 0
    AF207723.1          ------------------------------------------------------
------ 0
    EU548038.1          ------------------------------------------------------
------ 0
    EU548036.1          ------------------------------------------------------
------ 0
    EU548035.1          ------------------------------------------------------
------ 0
    AF207720.1          ------------------------------------------------------
------ 0
    AB601576.1          ------------------------------------------------------
------ 0
    EU548040.1          ------------------------------------------------------
------ 0
    EU548039.1          ------------------------------------------------------
------ 0
    AF207721.1          ------------------------------------------------------
------ 0
    AF207724.1          ------------------------------------------------------
------ 0
    JX982499.1          ------------------------------------------------------
------ 0
    EU548041.1          ------------------------------------------------------
------ 0
    EU548045.1          ------------------------------------------------------
------ 0
    AF207725.1          ------------------------------------------------------
------ 0
    EU548046.1          ------------------------------------------------------
------ 0
    AF207714.1          ------------------------------------------------------
------ 0
    AF207713.1          ------------------------------------------------------
------ 0
    AF207712.1          ------------------------------------------------------
------ 0
```

```
    AY750628.1       ------------------------------------------------------
------ 1044
    EF689084.1       ------------------------------------------------------
------ 0
    EF689085.1       ------------------------------------------------------
------ 0
    AB119070.1       ------------------------------------------------------
------ 956
    EF987742.1       ------------------------------------------------------
------ 0
    AB026105.1       ------------------------------------------------------
------ 0
    MW148603.1
AAAAAGCCTGAATCACCTGTATCATAAATAACTCAATCACCCGCCCCATTAAACTTCAAT   13740
    AB051263.1       ------------------------------------------------------
------ 0
    AF068544.1       ------------------------------------------------------
------ 0


    JX982502.1       ------------------------------------------------------
------ 0
    JX982501.1       ------------------------------------------------------
------ 0
    JX982498.1       ------------------------------------------------------
------ 0
    JX982497.1       ------------------------------------------------------
------ 0
    JX982495.1       ------------------------------------------------------
------ 0
    JX982496.1       ------------------------------------------------------
------ 0
    JX982500.1       ------------------------------------------------------
------ 0
    EU548051.1       ------------------------------------------------------
------ 0
    EU548044.1       ------------------------------------------------------
------ 0
    EU548042.1       ------------------------------------------------------
------ 0
    EU548043.1       ------------------------------------------------------
------ 0
    EU548047.1       ------------------------------------------------------
------ 0
    EU548050.1       ------------------------------------------------------
------ 0
    EU548048.1       ------------------------------------------------------
------ 0
    EU548049.1       ------------------------------------------------------
------ 0
    AF207722.1       ------------------------------------------------------
------ 0
    EU548037.1       ------------------------------------------------------
------ 0
    AF207723.1       ------------------------------------------------------
------ 0
    EU548038.1       ------------------------------------------------------
------ 0
    EU548036.1       ------------------------------------------------------
------ 0
    EU548035.1       ------------------------------------------------------
------ 0
    AF207720.1       ------------------------------------------------------
------ 0
    AB601576.1       ------------------------------------------------------
------ 0
```

EU548040.1 -- 0
EU548039.1 -- 0
AF207721.1 -- 0
AF207724.1 -- 0
JX982499.1 -- 0
EU548041.1 -- 0
EU548045.1 -- 0
AF207725.1 -- 0
EU548046.1 -- 0
AF207714.1 -- 0
AF207713.1 -- 0
AF207712.1 -- 0
AY750628.1 -- 1044
EF689084.1 -- 0
EF689085.1 -- 0
AB119070.1 -- 956
EF987742.1 -- 0
AB026105.1 -- 0
MW148603.1 ACAACTTCAACCTCAACATCATCACCTTTTAAAATATAACAAGCAGTCAATAACTCAGAT 13800
AB051263.1 -- 0
AF068544.1 -- 0

JX982502.1 -- 0
JX982501.1 -- 0
JX982498.1 -- 0
JX982497.1 -- 0
JX982495.1 -- 0
JX982496.1 -- 0
JX982500.1 -- 0
EU548051.1 -- 0
EU548044.1 -- 0
EU548042.1 -- 0
EU548043.1 -- 0

EU548047.1 --
------ 0
EU548050.1 --
------ 0
EU548048.1 --
------ 0
EU548049.1 --
------ 0
AF207722.1 --
------ 0
EU548037.1 --
------ 0
AF207723.1 --
------ 0
EU548038.1 --
------ 0
EU548036.1 --
------ 0
EU548035.1 --
------ 0
AF207720.1 --
------ 0
AB601576.1 --
------ 0
EU548040.1 --
------ 0
EU548039.1 --
------ 0
AF207721.1 --
------ 0
AF207724.1 --
------ 0
JX982499.1 --
------ 0
EU548041.1 --
------ 0
EU548045.1 --
------ 0
AF207725.1 --
------ 0
EU548046.1 --
------ 0
AF207714.1 --
------ 0
AF207713.1 --
------ 0
AF207712.1 --
------ 0
AY750628.1 --
------ 1044
EF689084.1 --
------ 0
EF689085.1 --
------ 0
AB119070.1 --
------ 956
EF987742.1 --
------ 0
AB026105.1 --
------ 0
MW148603.1
AACAAGCCAACAATAAAGGCACCTAAAACAGCCTTATTAGAGACCCAAACCTCAGGATAC 13860
AB051263.1 --
------ 0
AF068544.1 --
------ 0

```
    JX982502.1          ------------------------------------------------------
------ 0
    JX982501.1          ------------------------------------------------------
------ 0
    JX982498.1          ------------------------------------------------------
------ 0
    JX982497.1          ------------------------------------------------------
------ 0
    JX982495.1          ------------------------------------------------------
------ 0
    JX982496.1          ------------------------------------------------------
------ 0
    JX982500.1          ------------------------------------------------------
------ 0
    EU548051.1          ------------------------------------------------------
------ 0
    EU548044.1          ------------------------------------------------------
------ 0
    EU548042.1          ------------------------------------------------------
------ 0
    EU548043.1          ------------------------------------------------------
------ 0
    EU548047.1          ------------------------------------------------------
------ 0
    EU548050.1          ------------------------------------------------------
------ 0
    EU548048.1          ------------------------------------------------------
------ 0
    EU548049.1          ------------------------------------------------------
------ 0
    AF207722.1          ------------------------------------------------------
------ 0
    EU548037.1          ------------------------------------------------------
------ 0
    AF207723.1          ------------------------------------------------------
------ 0
    EU548038.1          ------------------------------------------------------
------ 0
    EU548036.1          ------------------------------------------------------
------ 0
    EU548035.1          ------------------------------------------------------
------ 0
    AF207720.1          ------------------------------------------------------
------ 0
    AB601576.1          ------------------------------------------------------
------ 0
    EU548040.1          ------------------------------------------------------
------ 0
    EU548039.1          ------------------------------------------------------
------ 0
    AF207721.1          ------------------------------------------------------
------ 0
    AF207724.1          ------------------------------------------------------
------ 0
    JX982499.1          ------------------------------------------------------
------ 0
    EU548041.1          ------------------------------------------------------
------ 0
    EU548045.1          ------------------------------------------------------
------ 0
    AF207725.1          ------------------------------------------------------
------ 0
    EU548046.1          ------------------------------------------------------
------ 0
```

```
   AF207714.1      ------------------------------------------------------
------ 0
   AF207713.1      ------------------------------------------------------
------ 0
   AF207712.1      ------------------------------------------------------
------ 0
   AY750628.1      ------------------------------------------------------
------ 1044
   EF689084.1      ------------------------------------------------------
------ 0
   EF689085.1      ------------------------------------------------------
------ 0
   AB119070.1      ------------------------------------------------------
------ 956
   EF987742.1      ------------------------------------------------------
------ 0
   AB026105.1      ------------------------------------------------------
------ 0
   MW148603.1
TGCTCCGTAGCTATAGCTGTAGTGTATCCAAAAACAACTAACATTCCCCCCAAATAAATT   13920
   AB051263.1      ------------------------------------------------------
------ 0
   AF068544.1      ------------------------------------------------------
------ 0


   JX982502.1      ------------------------------------------------------
------ 0
   JX982501.1      ------------------------------------------------------
------ 0
   JX982498.1      ------------------------------------------------------
------ 0
   JX982497.1      ------------------------------------------------------
------ 0
   JX982495.1      ------------------------------------------------------
------ 0
   JX982496.1      ------------------------------------------------------
------ 0
   JX982500.1      ------------------------------------------------------
------ 0
   EU548051.1      ------------------------------------------------------
------ 0
   EU548044.1      ------------------------------------------------------
------ 0
   EU548042.1      ------------------------------------------------------
------ 0
   EU548043.1      ------------------------------------------------------
------ 0
   EU548047.1      ------------------------------------------------------
------ 0
   EU548050.1      ------------------------------------------------------
------ 0
   EU548048.1      ------------------------------------------------------
------ 0
   EU548049.1      ------------------------------------------------------
------ 0
   AF207722.1      ------------------------------------------------------
------ 0
   EU548037.1      ------------------------------------------------------
------ 0
   AF207723.1      ------------------------------------------------------
------ 0
   EU548038.1      ------------------------------------------------------
------ 0
   EU548036.1      ------------------------------------------------------
------ 0
```

EU548035.1 --
------ 0
AF207720.1 --
------ 0
AB601576.1 --
------ 0
EU548040.1 --
------ 0
EU548039.1 --
------ 0
AF207721.1 --
------ 0
AF207724.1 --
------ 0
JX982499.1 --
------ 0
EU548041.1 --
------ 0
EU548045.1 --
------ 0
AF207725.1 --
------ 0
EU548046.1 --
------ 0
AF207714.1 --
------ 0
AF207713.1 --
------ 0
AF207712.1 --
------ 0
AY750628.1 --
------ 1044
EF689084.1 --
------ 0
EF689085.1 --
------ 0
AB119070.1 --
------ 956
EF987742.1 --
------ 0
AB026105.1 --
------ 0
MW148603.1
AAAAATACTATTAAACCTAAAAAAGACCCTCCAAAACACAACACAATTCCACAACCAATA 13980
AB051263.1 --
------ 0
AF068544.1 --
------ 0

JX982502.1 --
------ 0
JX982501.1 --
------ 0
JX982498.1 --
------ 0
JX982497.1 --
------ 0
JX982495.1 --
------ 0
JX982496.1 --
------ 0
JX982500.1 --
------ 0
EU548051.1 --
------ 0

```
   EU548044.1      ------------------------------------------------------
------ 0
   EU548042.1      ------------------------------------------------------
------ 0
   EU548043.1      ------------------------------------------------------
------ 0
   EU548047.1      ------------------------------------------------------
------ 0
   EU548050.1      ------------------------------------------------------
------ 0
   EU548048.1      ------------------------------------------------------
------ 0
   EU548049.1      ------------------------------------------------------
------ 0
   AF207722.1      ------------------------------------------------------
------ 0
   EU548037.1      ------------------------------------------------------
------ 0
   AF207723.1      ------------------------------------------------------
------ 0
   EU548038.1      ------------------------------------------------------
------ 0
   EU548036.1      ------------------------------------------------------
------ 0
   EU548035.1      ------------------------------------------------------
------ 0
   AF207720.1      ------------------------------------------------------
------ 0
   AB601576.1      ------------------------------------------------------
------ 0
   EU548040.1      ------------------------------------------------------
------ 0
   EU548039.1      ------------------------------------------------------
------ 0
   AF207721.1      ------------------------------------------------------
------ 0
   AF207724.1      ------------------------------------------------------
------ 0
   JX982499.1      ------------------------------------------------------
------ 0
   EU548041.1      ------------------------------------------------------
------ 0
   EU548045.1      ------------------------------------------------------
------ 0
   AF207725.1      ------------------------------------------------------
------ 0
   EU548046.1      ------------------------------------------------------
------ 0
   AF207714.1      ------------------------------------------------------
------ 0
   AF207713.1      ------------------------------------------------------
------ 0
   AF207712.1      ------------------------------------------------------
------ 0
   AY750628.1      ------------------------------------------------------
------ 1044
   EF689084.1      ------------------------------------------------------
------ 0
   EF689085.1      ------------------------------------------------------
------ 0
   AB119070.1      ------------------------------------------------------
------ 956
   EF987742.1      ------------------------------------------------------
------ 0
   AB026105.1      ------------------------------------------------------
------ 0
```

```
    MW148603.1
GCCCCACTAATAATTAAAACCAACCCGCCGTAGATAGGAGAGGGTTTTGAAGAAAACCCT   14040
    AB051263.1       ------------------------------------------------------
------ 0
    AF068544.1       ------------------------------------------------------
------ 0


    JX982502.1       ------------------------------------------------------
------ 0
    JX982501.1       ------------------------------------------------------
------ 0
    JX982498.1       ------------------------------------------------------
------ 0
    JX982497.1       ------------------------------------------------------
------ 0
    JX982495.1       ------------------------------------------------------
------ 0
    JX982496.1       ------------------------------------------------------
------ 0
    JX982500.1       ------------------------------------------------------
------ 0
    EU548051.1       ------------------------------------------------------
------ 0
    EU548044.1       ------------------------------------------------------
------ 0
    EU548042.1       ------------------------------------------------------
------ 0
    EU548043.1       ------------------------------------------------------
------ 0
    EU548047.1       ------------------------------------------------------
------ 0
    EU548050.1       ------------------------------------------------------
------ 0
    EU548048.1       ------------------------------------------------------
------ 0
    EU548049.1       ------------------------------------------------------
------ 0
    AF207722.1       ------------------------------------------------------
------ 0
    EU548037.1       ------------------------------------------------------
------ 0
    AF207723.1       ------------------------------------------------------
------ 0
    EU548038.1       ------------------------------------------------------
------ 0
    EU548036.1       ------------------------------------------------------
------ 0
    EU548035.1       ------------------------------------------------------
------ 0
    AF207720.1       ------------------------------------------------------
------ 0
    AB601576.1       ------------------------------------------------------
------ 0
    EU548040.1       ------------------------------------------------------
------ 0
    EU548039.1       ------------------------------------------------------
------ 0
    AF207721.1       ------------------------------------------------------
------ 0
    AF207724.1       ------------------------------------------------------
------ 0
    JX982499.1       ------------------------------------------------------
------ 0
    EU548041.1       ------------------------------------------------------
------ 0
```

EU548045.1 --
------ 0
AF207725.1 --
------ 0
EU548046.1 --
------ 0
AF207714.1 --
------ 0
AF207713.1 --
------ 0
AF207712.1 --
------ 0
AY750628.1 --
------ 1044
EF689084.1 --
------ 0
EF689085.1 --
------ 0
AB119070.1 --
------ 956
EF987742.1 --
------ 0
AB026105.1 --
------ 0
MW148603.1
ACAAAACTAACTACAAAAATAACACTTAGAATAAATACAATGTATGTCATCATTATTCCT 14100
AB051263.1 --
------ 0
AF068544.1 --
------ 0

JX982502.1 --
------ 0
JX982501.1 --
------ 0
JX982498.1 --
------ 0
JX982497.1 --
------ 0
JX982495.1 --
------ 0
JX982496.1 --
------ 0
JX982500.1 --
------ 0
EU548051.1 --
------ 0
EU548044.1 --
------ 0
EU548042.1 --
------ 0
EU548043.1 --
------ 0
EU548047.1 --
------ 0
EU548050.1 --
------ 0
EU548048.1 --
------ 0
EU548049.1 --
------ 0
AF207722.1 --
------ 0
EU548037.1 --
------ 0

```
    AF207723.1          ------------------------------------------------------
------ 0
    EU548038.1          ------------------------------------------------------
------ 0
    EU548036.1          ------------------------------------------------------
------ 0
    EU548035.1          ------------------------------------------------------
------ 0
    AF207720.1          ------------------------------------------------------
------ 0
    AB601576.1          ------------------------------------------------------
------ 0
    EU548040.1          ------------------------------------------------------
------ 0
    EU548039.1          ------------------------------------------------------
------ 0
    AF207721.1          ------------------------------------------------------
------ 0
    AF207724.1          ------------------------------------------------------
------ 0
    JX982499.1          ------------------------------------------------------
------ 0
    EU548041.1          ------------------------------------------------------
------ 0
    EU548045.1          ------------------------------------------------------
------ 0
    AF207725.1          ------------------------------------------------------
------ 0
    EU548046.1          ------------------------------------------------------
------ 0
    AF207714.1          ------------------------------------------------------
------ 0
    AF207713.1          ------------------------------------------------------
------ 0
    AF207712.1          ------------------------------------------------------
------ 0
    AY750628.1          ------------------------------------------------------
------ 1044
    EF689084.1          ------------------------------------------------------
------ 0
    EF689085.1          ------------------------------------------------------
------ 0
    AB119070.1          ------------------------------------------------------
------ 956
    EF987742.1          ------------------------------------------------------
------ 0
    AB026105.1          ------------------------------------------------------
------ 0
    MW148603.1
ACATGGAATCTAACCATGACTAGTGACATGAAAAATCACTGTTGTATTTCAACTATAAGA   14160
    AB051263.1          ------------------------------------------------------
------ 0
    AF068544.1          ------------------------------------------------------
------ 0


    JX982502.1          ------------------------------------------------------
------ 0
    JX982501.1          ------------------------------------------------------
------ 0
    JX982498.1          ------------------------------------------------------
------ 0
    JX982497.1          ------------------------------------------------------
------ 0
    JX982495.1          ------------------------------------------------------
------ 0
```

JX982496.1 --
------ 0
JX982500.1 --
------ 0
EU548051.1 --
------ 0
EU548044.1 --
------ 0
EU548042.1 --
------ 0
EU548043.1 --
------ 0
EU548047.1 --
------ 0
EU548050.1 --
------ 0
EU548048.1 --
------ 0
EU548049.1 --
------ 0
AF207722.1 --
------ 0
EU548037.1 --
------ 0
AF207723.1 --
------ 0
EU548038.1 --
------ 0
EU548036.1 --
------ 0
EU548035.1 --
------ 0
AF207720.1 --
------ 0
AB601576.1 --
------ 0
EU548040.1 --
------ 0
EU548039.1 --
------ 0
AF207721.1 --
------ 0
AF207724.1 --
------ 0
JX982499.1 --
------ 0
EU548041.1 --
------ 0
EU548045.1 --
------ 0
AF207725.1 --
------ 0
EU548046.1 --
------ 0
AF207714.1 --
CATCAACAACTCATTC 16
AF207713.1 --
CATCAACAACTCACTC 16
AF207712.1 --
CATCAACAACTCACTC 16
AY750628.1 --
------ 1044
EF689084.1 ------
ATGACCAACATTCGTAAAACCCACCCACTAACCAAAATCATCAACAACTCACTC 54
EF689085.1 ------
ATGACCAACATTCGTAAAACCCACCCACTAACCAAAATCATCAACAACTCACTC 54

```
    AB119070.1      ------------------------------------------------------
------ 956
    EF987742.1                                                      ------
ATGACCAACATTCGTAAAACCCACCCACTAACCAAAATCATCAACAACTCACTC  54
    AB026105.1                                                      ------
ATGACCAACATTCGTAAAACCCACCCACTAACCAAAATCATCAACAACTCACTC  54
    MW148603.1
ACATTAATGACCAACATTCGTAAAACCCACCCACTAACCAAAATCATCAACAACTCACTC   14220
    AB051263.1                                                      ------
ATGACCAACATTCGTAAAACCCACCCACTAACCAAAATCATCAACAACTCACTC  54
    AF068544.1                --------------------------------------------
CATCAACAACTCACTC  16


    JX982502.1      ------------------------------------------------------
------ 0
    JX982501.1      ------------------------------------------------------
------ 0
    JX982498.1      ------------------------------------------------------
------ 0
    JX982497.1      ------------------------------------------------------
------ 0
    JX982495.1      ------------------------------------------------------
------ 0
    JX982496.1      ------------------------------------------------------
------ 0
    JX982500.1      ------------------------------------------------------
------ 0
    EU548051.1      ------------------------------------------------------
------ 0
    EU548044.1      ------------------------------------------------------
------ 0
    EU548042.1      ------------------------------------------------------
------ 0
    EU548043.1      ------------------------------------------------------
------ 0
    EU548047.1      ------------------------------------------------------
------ 0
    EU548050.1      ------------------------------------------------------
------ 0
    EU548048.1      ------------------------------------------------------
------ 0
    EU548049.1      ------------------------------------------------------
------ 0
    AF207722.1      ------------------------------------------------------
------ 0
    EU548037.1      ------------------------------------------------------
------ 0
    AF207723.1      ------------------------------------------------------
------ 0
    EU548038.1      ------------------------------------------------------
------ 0
    EU548036.1      ------------------------------------------------------
------ 0
    EU548035.1      ------------------------------------------------------
------ 0
    AF207720.1      ------------------------------------------------------
------ 0
    AB601576.1      ------------------------------------------------------
------ 0
    EU548040.1      ------------------------------------------------------
------ 0
    EU548039.1      ------------------------------------------------------
------ 0
    AF207721.1      ------------------------------------------------------
------ 0
```

AF207724.1 --
------ 0
JX982499.1 --
------ 0
EU548041.1 --
------ 0
EU548045.1 --
------ 0
AF207725.1 --
------ 0
EU548046.1 --
------ 0
AF207714.1
ATTGACCTACCCGCCCCATCAAACATTTCAGCATGATGAAACTTCGGCTCCCTTCTCGGA 76
AF207713.1
ATTGACCTACCCACCCCATCAAACATTTCAGCATGATGAAACTTCGGCTCCCTTCTCGGA 76
AF207712.1
ATTGACCTACCCGCCCCATCAAACATTTCAGCATGATGAAACTTCGGCTCCCTTCTCGGA 76
AY750628.1 --
------ 1044
EF689084.1
ATTGACCTACCCGCCCCATCAAACATTTCAGCATGATGAAACTTCGGCTCCCTTCTCGGA 114
EF689085.1
ATTGACCTACCCGCCCCATCAAACATTTCAGCATGATGAAACTTCGGCTCCCTTCTCGGA 114
AB119070.1 --
------ 956
EF987742.1
ATTGACCTACCCGCCCCATCAAACATTTCAGCATGATGAAACTTCGGCTCCCTTCTCGGA 114
AB026105.1
ATTGACCTACCCGCCCCATCAAACATTTCAGCATGATGAAACTTCGGCTCCCTTCTCGGA 114
MW148603.1
ATTGACCTACCCGCCCCATCAAACATTTCAGCATGATGAAACTTCGGCTCCCTTCTCGGA 14280
AB051263.1
ATTGACCTACCCGCCCCATCAAACATTTCAGCATGATGAAACTTCGGCTCCCTTCTCGGA 114
AF068544.1
ATTGACCTACCCGCCCCATCAAACATTTCAGCATGATGAAACTTCGGCTCCCTTCTCGGA 76

JX982502.1 --
------ 0
JX982501.1 --
------ 0
JX982498.1 --
------ 0
JX982497.1 --
------ 0
JX982495.1 --
------ 0
JX982496.1 --
------ 0
JX982500.1 --
------ 0
EU548051.1 --
------ 0
EU548044.1 --
------ 0
EU548042.1 --
------ 0
EU548043.1 --
------ 0
EU548047.1 --
------ 0
EU548050.1 --
------ 0
EU548048.1 --
------ 0

EU548049.1 --
------ 0
AF207722.1 --
------ 0
EU548037.1 --
------ 0
AF207723.1 --
------ 0
EU548038.1 --
------ 0
EU548036.1 --
------ 0
EU548035.1 --
------ 0
AF207720.1 --
------ 0
AB601576.1 --
------ 0
EU548040.1 --
------ 0
EU548039.1 --
------ 0
AF207721.1 --
------ 0
AF207724.1 --
------ 0
JX982499.1 --
------ 0
EU548041.1 --
------ 0
EU548045.1 --
------ 0
AF207725.1 --
------ 0
EU548046.1 --
------ 0
AF207714.1
ATCTGCCTAATTATTCAGATTCTTACAGGTTTATTTTTAGCCATACACTATACATCAGAC 136
AF207713.1
ATCTGCCTAATTATTCAGATTCTTACAGGTTTATTTTTAGCCATACACTATACATCAGAC 136
AF207712.1
ATCTGCCTAATTATTCAGATTCTTACAGGTTTATTTTTAGCCATACACTATACATCAGAC 136
AY750628.1 --
------ 1044
EF689084.1
ATCTGCCTAATTATTCAGATTCTTACAGGTTTATTTTTAGCCATACACTATACATCAGAC 174
EF689085.1
ATCTGCCTAATTATTCAGATTCTTACAGGTTTATTTTTAGCCATACACTATACATCAGAC 174
AB119070.1 --
------ 956
EF987742.1
ATCTGCCTAATTATTCAGATTCTTACAGGTTTATTTTTAGCCATACACTATACATCAGAC 174
AB026105.1
ATCTGCCTAATTATTCAGATTCTTACAGGTTTATTTTTAGCCATACACTATACATCAGAC 174
MW148603.1
ATCTGCCTAATTATTCAGATTCTTACAGGTTTATTTTTAGCCATACACTATACATCAGAC 14340
AB051263.1
ATCTGCCTAATTATTCAGATTCTTACAGGTTTATTTTTAGCCATACACTATACATCAGAC 174
AF068544.1
ATCTGCCTAATTATTCAGATTCTTACAGGTTTATTTTTAGCCATACACTATACATCAGAC 136

JX982502.1 --
------ 0
JX982501.1 --
------ 0

```
    JX982498.1       ------------------------------------------------------
------ 0
    JX982497.1       ------------------------------------------------------
------ 0
    JX982495.1       ------------------------------------------------------
------ 0
    JX982496.1       ------------------------------------------------------
------ 0
    JX982500.1       ------------------------------------------------------
------ 0
    EU548051.1       ------------------------------------------------------
------ 0
    EU548044.1       ------------------------------------------------------
------ 0
    EU548042.1       ------------------------------------------------------
------ 0
    EU548043.1       ------------------------------------------------------
------ 0
    EU548047.1       ------------------------------------------------------
------ 0
    EU548050.1       ------------------------------------------------------
------ 0
    EU548048.1       ------------------------------------------------------
------ 0
    EU548049.1       ------------------------------------------------------
------ 0
    AF207722.1       ------------------------------------------------------
------ 0
    EU548037.1       ------------------------------------------------------
------ 0
    AF207723.1       ------------------------------------------------------
------ 0
    EU548038.1       ------------------------------------------------------
------ 0
    EU548036.1       ------------------------------------------------------
------ 0
    EU548035.1       ------------------------------------------------------
------ 0
    AF207720.1       ------------------------------------------------------
------ 0
    AB601576.1       ------------------------------------------------------
------ 0
    EU548040.1       ------------------------------------------------------
------ 0
    EU548039.1       ------------------------------------------------------
------ 0
    AF207721.1       ------------------------------------------------------
------ 0
    AF207724.1       ------------------------------------------------------
------ 0
    JX982499.1       ------------------------------------------------------
------ 0
    EU548041.1       ------------------------------------------------------
------ 0
    EU548045.1       ------------------------------------------------------
------ 0
    AF207725.1       ------------------------------------------------------
------ 0
    EU548046.1       ------------------------------------------------------
------ 0
    AF207714.1
ACAGCCACAGCCTTTTCATCAGTCACCCATATCTGTCGAGACGTCAACTATGGCTGAATT   196
    AF207713.1
ACAGCCACAGCCTTTTCATCAGTCACCCATATCTGTCGAGACGTCAACTATGGCTGAATT   196
    AF207712.1
ACAGCCACAGCCTTTTCATCAGTCACCCATATCTGTCGAGACGTCAACTATGGCTGAATT   196
```

AY750628.1 --
------ 1044
EF689084.1
ACAGCCACAGCCTTTTCATCAGTCACCCATATCTGTCGAGACGTCAACTATGGCTGAATT 234
EF689085.1
ACAGCCACAGCCTTTTCATCAGTCACCCATATCTGTCGAGACGTCAACTATGGCTGAATT 234
AB119070.1 --
------ 956
EF987742.1
ACAGCCACAGCCTTTTCATCAGTCACCCATATCTGTCGAGACGTCAACTATGGCTGAATT 234
AB026105.1
ACAGCCACAGCCTTTTCATCAGTCACCCATATCTGTCGAGACGTCAACTATGGCTGAATT 234
MW148603.1
ACAGCCACAGCCTTTTCATCAGTCACCCATATCTGTCGAGACGTCAACTATGGCTGAATT 14400
AB051263.1
ACAGCCACAGCCTTTTCATCAGTCACCCATATCTGTCGAGACGTCAACTATGGCTGAATT 234
AF068544.1
ACAGCCACAGCCTTTTCATCAGTCACCCATATCTGTCGAGACGTCAACTATGGCTGAATT 196

JX982502.1 --
------ 0
JX982501.1 --
------ 0
JX982498.1 --
------ 0
JX982497.1 --
------ 0
JX982495.1 --
------ 0
JX982496.1 --
------ 0
JX982500.1 --
------ 0
EU548051.1 --
------ 0
EU548044.1 --
------ 0
EU548042.1 --
------ 0
EU548043.1 --
------ 0
EU548047.1 --
------ 0
EU548050.1 --
------ 0
EU548048.1 --
------ 0
EU548049.1 --
------ 0
AF207722.1 --
------ 0
EU548037.1 --
------ 0
AF207723.1 --
------ 0
EU548038.1 --
------ 0
EU548036.1 --
------ 0
EU548035.1 --
------ 0
AF207720.1 --
------ 0
AB601576.1 --
------ 0

```
    EU548040.1      ------------------------------------------------------
------ 0
    EU548039.1      ------------------------------------------------------
------ 0
    AF207721.1      ------------------------------------------------------
------ 0
    AF207724.1      ------------------------------------------------------
------ 0
    JX982499.1      ------------------------------------------------------
------ 0
    EU548041.1      ------------------------------------------------------
------ 0
    EU548045.1      ------------------------------------------------------
------ 0
    AF207725.1      ------------------------------------------------------
------ 0
    EU548046.1      ------------------------------------------------------
------ 0
    AF207714.1
ATCCGATACATACACGCAAACGGAGCTTCCATATTCTTTATCTGCCTGTTCCTGCACGTA   256
    AF207713.1
ATCCGATACATACACGCAAACGGAGCTTCCATATTCTTTATCTGCCTGTTCCTGCACGTA   256
    AF207712.1
ATCCGGTACATACACGCAAACGGAGCTTCCATATTCTTTATCTGCCTGTTCCTGCACGTA   256
    AY750628.1      ------------------------------------------------------
------ 1044
    EF689084.1
ATCCGATACATACACGCAAACGGAGCTTCCATATTCTTTATCTGCCTGTTCCTGCACGTA   294
    EF689085.1
ATCCGATACATACACGCAAACGGAGCTTCCATATTCTTTATCTGCCTGTTCCTGCACGTA   294
    AB119070.1      ------------------------------------------------------
------ 956
    EF987742.1
ATCCGATACATACACGCAAACGGAGCTTCCATATTCTTTATCTGCCTGTTCCTGCACGTA   294
    AB026105.1
ATCCGATACATACACGCAAACGGAGCTTCCATATTCTTTATCTGCCTGTTCCTGCACGTA   294
    MW148603.1
ATCCGATACATACACGCAAACGGAGCTTCCATATTCTTTATCTGCCTGTTCCTGCACGTA   14460
    AB051263.1
ATCCGATACATACACGCAAACGGAGCTTCCATATTCTTTATCTGCCTGTTCCTGCACGTA   294
    AF068544.1
ATCCGATACATACACGCAAACGGAGCTTCCATATTCTTTATCTGCCTGTTCCTGCACGTA   256


    JX982502.1      ------------------------------------------------------
------ 0
    JX982501.1      ------------------------------------------------------
------ 0
    JX982498.1      ------------------------------------------------------
------ 0
    JX982497.1      ------------------------------------------------------
------ 0
    JX982495.1      ------------------------------------------------------
------ 0
    JX982496.1      ------------------------------------------------------
------ 0
    JX982500.1      ------------------------------------------------------
------ 0
    EU548051.1      ------------------------------------------------------
------ 0
    EU548044.1      ------------------------------------------------------
------ 0
    EU548042.1      ------------------------------------------------------
------ 0
    EU548043.1      ------------------------------------------------------
------ 0
```

```
   EU548047.1      ------------------------------------------------------
------ 0
   EU548050.1      ------------------------------------------------------
------ 0
   EU548048.1      ------------------------------------------------------
------ 0
   EU548049.1      ------------------------------------------------------
------ 0
   AF207722.1      ------------------------------------------------------
------ 0
   EU548037.1      ------------------------------------------------------
------ 0
   AF207723.1      ------------------------------------------------------
------ 0
   EU548038.1      ------------------------------------------------------
------ 0
   EU548036.1      ------------------------------------------------------
------ 0
   EU548035.1      ------------------------------------------------------
------ 0
   AF207720.1      ------------------------------------------------------
------ 0
   AB601576.1      ------------------------------------------------------
------ 0
   EU548040.1      ------------------------------------------------------
------ 0
   EU548039.1      ------------------------------------------------------
------ 0
   AF207721.1      ------------------------------------------------------
------ 0
   AF207724.1      ------------------------------------------------------
------ 0
   JX982499.1      ------------------------------------------------------
------ 0
   EU548041.1      ------------------------------------------------------
------ 0
   EU548045.1      ------------------------------------------------------
------ 0
   AF207725.1      ------------------------------------------------------
------ 0
   EU548046.1      ------------------------------------------------------
------ 0
   AF207714.1
GGGCGGGGTTTATATTATGGATCTTATATATTCACCGAAACATGAAACATCGGCATTATC   316
   AF207713.1
GGGCGGGGTTTATATTATGGATCTTATATATTCACCGAAACATGAAACATCGGCATTATC   316
   AF207712.1
GGGCGGGGTTTATATTATGGATCTTATATATTCACCGAAACATGAAACATCGGCATTATC   316
   AY750628.1      ------------------------------------------------------
------ 1044
   EF689084.1
GGGCGGGGTTTATATTATGGATCTTATATATTCACCGAAACATGAAACATCGGCATTATC   354
   EF689085.1
GGGCGGGGTTTATATTATGGATCTTATATATTCACCGAAACATGAAACATCGGCATTATC   354
   AB119070.1      ------------------------------------------------------
------ 956
   EF987742.1
GGGCGGGGTTTATATTATGGATCTTATATATTCACCGAAACATGAAACATCGGCATTATC   354
   AB026105.1
GGGCGGGGTTTATATTATGGATCTTATATATTCACCGAAACATGAAACATCGGCATTATC   354
   MW148603.1
GGGCGGGGTTTATATTATGGATCTTATATATTCACCGAAACATGAAACATCGGCATTATC   14520
   AB051263.1
GGGCGGGGTTTATATTATGGATCTTATATATTCACCGAAACATGAAACATCGGCATTATC   354
   AF068544.1
GGGCGGGGTTTATATTATGGATCTTATATATTCACCGAAACATGAAACATCGGCATTATC   316
```

```
    JX982502.1      ------------------------------------------------------
------ 0
    JX982501.1      ------------------------------------------------------
------ 0
    JX982498.1      ------------------------------------------------------
------ 0
    JX982497.1      ------------------------------------------------------
------ 0
    JX982495.1      ------------------------------------------------------
------ 0
    JX982496.1      ------------------------------------------------------
------ 0
    JX982500.1      ------------------------------------------------------
------ 0
    EU548051.1      ------------------------------------------------------
------ 0
    EU548044.1      ------------------------------------------------------
------ 0
    EU548042.1      ------------------------------------------------------
------ 0
    EU548043.1      ------------------------------------------------------
------ 0
    EU548047.1      ------------------------------------------------------
------ 0
    EU548050.1      ------------------------------------------------------
------ 0
    EU548048.1      ------------------------------------------------------
------ 0
    EU548049.1      ------------------------------------------------------
------ 0
    AF207722.1      ------------------------------------------------------
------ 0
    EU548037.1      ------------------------------------------------------
------ 0
    AF207723.1      ------------------------------------------------------
------ 0
    EU548038.1      ------------------------------------------------------
------ 0
    EU548036.1      ------------------------------------------------------
------ 0
    EU548035.1      ------------------------------------------------------
------ 0
    AF207720.1      ------------------------------------------------------
------ 0
    AB601576.1      ------------------------------------------------------
------ 0
    EU548040.1      ------------------------------------------------------
------ 0
    EU548039.1      ------------------------------------------------------
------ 0
    AF207721.1      ------------------------------------------------------
------ 0
    AF207724.1      ------------------------------------------------------
------ 0
    JX982499.1      ------------------------------------------------------
------ 0
    EU548041.1      ------------------------------------------------------
------ 0
    EU548045.1      ------------------------------------------------------
------ 0
    AF207725.1      ------------------------------------------------------
------ 0
    EU548046.1      ------------------------------------------------------
------ 0
```

```
   AF207714.1      TTATTGTTCGCAGTCATAGCA---------------------------------
------ 337
   AF207713.1      TTATTGTTCGCAGTCATAGCA---------------------------------
------ 337
   AF207712.1      TTATTGTTCGCAGTCATAGCA---------------------------------
------ 337
   AY750628.1      ------------------------------------------------------
------ 1044
   EF689084.1
TTATTGTTCGCAGTCATAGCAACTGCATTCATAGGTTACGTTTTACCATGAGGACAAATA   414
   EF689085.1
TTATTGTTCGCAGTCATAGCAACTGCATTCATAGGTTACGTTTTACCATGAGGACAAATA   414
   AB119070.1      ------------------------------------------------------
------ 956
   EF987742.1
TTATTGTTCGCAGTCATAGCAACTGCATTCATAGGTTACGTTTTACCATGGGGACAAATA   414
   AB026105.1
TTATTGTTCGCAGTCATAGCAACTGCATTCATAGGTTACGTTTTACCATGGGGACAAATA   414
   MW148603.1
TTATTGTTCGCAGTCATAGCAACTGCATTCATAGGTTACGTTTTACCATGGGGACAAATA   14580
   AB051263.1      TTATTGTTCGCAGTCATAGCAACTGCATTCATAGGTTACGTTTTACCA------
------ 402
   AF068544.1      TTATTGTTCGCAGTCATAGCA---------------------------------
------ 337


   JX982502.1      ------------------------------------------------------
------ 0
   JX982501.1      ------------------------------------------------------
------ 0
   JX982498.1      ------------------------------------------------------
------ 0
   JX982497.1      ------------------------------------------------------
------ 0
   JX982495.1      ------------------------------------------------------
------ 0
   JX982496.1      ------------------------------------------------------
------ 0
   JX982500.1      ------------------------------------------------------
------ 0
   EU548051.1      ------------------------------------------------------
------ 0
   EU548044.1      ------------------------------------------------------
------ 0
   EU548042.1      ------------------------------------------------------
------ 0
   EU548043.1      ------------------------------------------------------
------ 0
   EU548047.1      ------------------------------------------------------
------ 0
   EU548050.1      ------------------------------------------------------
------ 0
   EU548048.1      ------------------------------------------------------
------ 0
   EU548049.1      ------------------------------------------------------
------ 0
   AF207722.1      ------------------------------------------------------
------ 0
   EU548037.1      ------------------------------------------------------
------ 0
   AF207723.1      ------------------------------------------------------
------ 0
   EU548038.1      ------------------------------------------------------
------ 0
   EU548036.1      ------------------------------------------------------
------ 0
```

```
   EU548035.1      ------------------------------------------------------
------ 0
   AF207720.1      ------------------------------------------------------
------ 0
   AB601576.1      ------------------------------------------------------
------ 0
   EU548040.1      ------------------------------------------------------
------ 0
   EU548039.1      ------------------------------------------------------
------ 0
   AF207721.1      ------------------------------------------------------
------ 0
   AF207724.1      ------------------------------------------------------
------ 0
   JX982499.1      ------------------------------------------------------
------ 0
   EU548041.1      ------------------------------------------------------
------ 0
   EU548045.1      ------------------------------------------------------
------ 0
   AF207725.1      ------------------------------------------------------
------ 0
   EU548046.1      ------------------------------------------------------
------ 0
   AF207714.1      ------------------------------------------------------
------ 337
   AF207713.1      ------------------------------------------------------
------ 337
   AF207712.1      ------------------------------------------------------
------ 337
   AY750628.1      ------------------------------------------------------
------ 1044
   EF689084.1
TCATTTTGGGGTGCAACCGTAATTACCAACTTACTGTCTGCTATCCCATATATCGGAACT   474
   EF689085.1
TCATTTTGGGGTGCAACCGTAATTACCAACTTACTGTCTGCTATCCCATATATCGGAACT   474
   AB119070.1      ------------------------------------------------------
------ 956
   EF987742.1
TCATTTTGGGGTGCAACCGTAATTACCAACTTACTGTCTGCTATCCCATATATCGGAACT   474
   AB026105.1
TCATTTTGGGGTGCAACCGTAATTACCAACTTACTGTCTGCTATCCCATATATCGGAACT   474
   MW148603.1
TCATTTTGGGGTGCAACCGTAATTACCAACTTACTGTCTGCTATCCCATATATCGGAACT   14640
   AB051263.1      ------------------------------------------------------
------ 402
   AF068544.1      ------------------------------------------------------
------ 337


   JX982502.1      ------------------------------------------------------
------ 0
   JX982501.1      ------------------------------------------------------
------ 0
   JX982498.1      ------------------------------------------------------
------ 0
   JX982497.1      ------------------------------------------------------
------ 0
   JX982495.1      ------------------------------------------------------
------ 0
   JX982496.1      ------------------------------------------------------
------ 0
   JX982500.1      ------------------------------------------------------
------ 0
   EU548051.1      ------------------------------------------------------
------ 0
```

EU548044.1 --
------ 0
EU548042.1 --
------ 0
EU548043.1 --
------ 0
EU548047.1 --
------ 0
EU548050.1 --
------ 0
EU548048.1 --
------ 0
EU548049.1 --
------ 0
AF207722.1 --
------ 0
EU548037.1 --
------ 0
AF207723.1 --
------ 0
EU548038.1 --
------ 0
EU548036.1 --
------ 0
EU548035.1 --
------ 0
AF207720.1 --
------ 0
AB601576.1 --
------ 0
EU548040.1 --
------ 0
EU548039.1 --
------ 0
AF207721.1 --
------ 0
AF207724.1 --
------ 0
JX982499.1 --
------ 0
EU548041.1 --
------ 0
EU548045.1 --
------ 0
AF207725.1 --
------ 0
EU548046.1 --
------ 0
AF207714.1 --
------ 337
AF207713.1 --
------ 337
AF207712.1 --
------ 337
AY750628.1 --
------ 1044
EF689084.1
AATCTTGTAGAATGAATCTGAGGCGGATTCTCAGTAGATAAAGCTACCCTAACACGATTC 534
EF689085.1
AATCTTGTAGAATGAATCTGAGGCGGATTCTCAGTAGATAAAGCTACCCTAACACGATTC 534
AB119070.1 --
------ 956
EF987742.1
AATCTTGTAGAATGAATCTGAGGCGGATTCTCAGTAGATAAAGCTACCCTAACACGATTC 534
AB026105.1
AATCTTGTAGAATGAATCTGAGGCGGATTCTCAGTAGATAAAGCTACCCTAACACGATTC 534

MW148603.1
AATCTTGTAGAATGAATCTGAGGCGGATTCTCAGTAGATAAAGCTACCCTAACACGATTC 14700
AB051263.1 --
------ 402
AF068544.1 --
------ 337

JX982502.1 --
------ 0
JX982501.1 --
------ 0
JX982498.1 --
------ 0
JX982497.1 --
------ 0
JX982495.1 --
------ 0
JX982496.1 --
------ 0
JX982500.1 --
------ 0
EU548051.1 --
------ 0
EU548044.1 --
------ 0
EU548042.1 --
------ 0
EU548043.1 --
------ 0
EU548047.1 --
------ 0
EU548050.1 --
------ 0
EU548048.1 --
------ 0
EU548049.1 --
------ 0
AF207722.1 --
------ 0
EU548037.1 --
------ 0
AF207723.1 --
------ 0
EU548038.1 --
------ 0
EU548036.1 --
------ 0
EU548035.1 --
------ 0
AF207720.1 --
------ 0
AB601576.1 --
------ 0
EU548040.1 --
------ 0
EU548039.1 --
------ 0
AF207721.1 --
------ 0
AF207724.1 --
------ 0
JX982499.1 --
------ 0
EU548041.1 --
------ 0

```
   EU548045.1      ------------------------------------------------------
------ 0
   AF207725.1      ------------------------------------------------------
------ 0
   EU548046.1      ------------------------------------------------------
------ 0
   AF207714.1      ------------------------------------------------------
------ 337
   AF207713.1      ------------------------------------------------------
------ 337
   AF207712.1      ------------------------------------------------------
------ 337
   AY750628.1      ------------------------------------------------------
------ 1044
   EF689084.1
TTCGCTTTCCACTTCATCCTACCATTCATCATTTCAGCCCTAGCAGCAGTCCACCTACTA   594
   EF689085.1
TTCGCTTTCCACTTCATCCTACCATTCATCATTTCAGCCCTAGCAGCAGTCCACCTACTA   594
   AB119070.1      ------------------------------------------------------
------ 956
   EF987742.1
TTCGCTTTCCACTTCATCCTACCATTCATCATTTCAGCCCTAGCAGCAGTCCACCTACTA   594
   AB026105.1
TTCGCTTTCCACTTCATCCTACCATTCATCATTTCAGCCCTAGCAGCAGTCCACCTACTA   594
   MW148603.1
TTCGCTTTCCACTTCATCCTACCATTCATCATTTCAGCCCTAGCAGCAGTCCACCTACTA   14760
   AB051263.1      ------------------------------------------------------
------ 402
   AF068544.1      ------------------------------------------------------
------ 337


   JX982502.1      ------------------------------------------------------
------ 0
   JX982501.1      ------------------------------------------------------
------ 0
   JX982498.1      ------------------------------------------------------
------ 0
   JX982497.1      ------------------------------------------------------
------ 0
   JX982495.1      ------------------------------------------------------
------ 0
   JX982496.1      ------------------------------------------------------
------ 0
   JX982500.1      ------------------------------------------------------
------ 0
   EU548051.1      ------------------------------------------------------
------ 0
   EU548044.1      ------------------------------------------------------
------ 0
   EU548042.1      ------------------------------------------------------
------ 0
   EU548043.1      ------------------------------------------------------
------ 0
   EU548047.1      ------------------------------------------------------
------ 0
   EU548050.1      ------------------------------------------------------
------ 0
   EU548048.1      ------------------------------------------------------
------ 0
   EU548049.1      ------------------------------------------------------
------ 0
   AF207722.1      ------------------------------------------------------
------ 0
   EU548037.1      ------------------------------------------------------
------ 0
```

```
   AF207723.1      ------------------------------------------------------
------ 0
   EU548038.1      ------------------------------------------------------
------ 0
   EU548036.1      ------------------------------------------------------
------ 0
   EU548035.1      ------------------------------------------------------
------ 0
   AF207720.1      ------------------------------------------------------
------ 0
   AB601576.1      ------------------------------------------------------
------ 0
   EU548040.1      ------------------------------------------------------
------ 0
   EU548039.1      ------------------------------------------------------
------ 0
   AF207721.1      ------------------------------------------------------
------ 0
   AF207724.1      ------------------------------------------------------
------ 0
   JX982499.1      ------------------------------------------------------
------ 0
   EU548041.1      ------------------------------------------------------
------ 0
   EU548045.1      ------------------------------------------------------
------ 0
   AF207725.1      ------------------------------------------------------
------ 0
   EU548046.1      ------------------------------------------------------
------ 0
   AF207714.1      ------------------------------------------------------
------ 337
   AF207713.1      ------------------------------------------------------
------ 337
   AF207712.1      ------------------------------------------------------
------ 337
   AY750628.1      ------------------------------------------------------
------ 1044
   EF689084.1
TTCCTCCACGAAACAGGATCTAATAACCCCTCCGGAATTCCATCTGACTCCGACAAAATC   654
   EF689085.1
TTCCTCCACGAAACAGGATCTAATAACCCCTCCGGAATTCCATCTGACTCCGACAAAATC   654
   AB119070.1      ------------------------------------------------------
------ 956
   EF987742.1
TTCCTCCACGAAACAGGATCTAATAACCCCTCCGGAATTCCATCTGACTCCGACAAAATC   654
   AB026105.1
TTCCTCCACGAAACAGGATCTAACAACCCCTCCGGAATTCCATCTGACTCCGACAAAATC   654
   MW148603.1
TTCCTCCACGAAACAGGATCTAATAACCCCTCCGGAATTCCATCTGACTCCGACAAAATC   14820
   AB051263.1      ------------------------------------------------------
------ 402
   AF068544.1      ------------------------------------------------------
------ 337


   JX982502.1      ------------------------------------------------------
------ 0
   JX982501.1      ------------------------------------------------------
------ 0
   JX982498.1      ------------------------------------------------------
------ 0
   JX982497.1      ------------------------------------------------------
------ 0
   JX982495.1      ------------------------------------------------------
------ 0
```

JX982496.1 --
------ 0
JX982500.1 --
------ 0
EU548051.1 --
------ 0
EU548044.1 --
------ 0
EU548042.1 --
------ 0
EU548043.1 --
------ 0
EU548047.1 --
------ 0
EU548050.1 --
------ 0
EU548048.1 --
------ 0
EU548049.1 --
------ 0
AF207722.1 --
------ 0
EU548037.1 --
------ 0
AF207723.1 --
------ 0
EU548038.1 --
------ 0
EU548036.1 --
------ 0
EU548035.1 --
------ 0
AF207720.1 --
------ 0
AB601576.1 --
------ 0
EU548040.1 --
------ 0
EU548039.1 --
------ 0
AF207721.1 --
------ 0
AF207724.1 --
------ 0
JX982499.1 --
------ 0
EU548041.1 --
------ 0
EU548045.1 --
------ 0
AF207725.1 --
------ 0
EU548046.1 --
------ 0
AF207714.1 --
------ 337
AF207713.1 --
------ 337
AF207712.1 --
------ 337
AY750628.1 --
------ 1044
EF689084.1
CCATTTCACCCTTACTACACCATCAAAGACATCCTAGGCGCCCTACTCCTTATTCTAATA 714
EF689085.1
CCATTTCACCCTTACTACACCATCAAAGACATCCTAGGCGCCCTACTCCTTATTCTAATA 714

AB119070.1 --
------ 956
EF987742.1
CCATTTCACCCTTACTACACCATCAAAGACATCCTAGGCGCCCTACTCCTTATTCTAATA 714
AB026105.1
CCATTTCACCCTTACTACACCATCAAAGACATCCTAGGCGCCCTACTCCTTATTCTAATA 714
MW148603.1
CCATTTCACCCTTACTACACCATCAAAGACATCCTAGGCGCCCTACTCCTTATTCTAATA 14880
AB051263.1 --
------ 402
AF068544.1 --
------ 337

JX982502.1 --
------ 0
JX982501.1 --
------ 0
JX982498.1 --
------ 0
JX982497.1 --
------ 0
JX982495.1 --
------ 0
JX982496.1 --
------ 0
JX982500.1 --
------ 0
EU548051.1 --
------ 0
EU548044.1 --
------ 0
EU548042.1 --
------ 0
EU548043.1 --
------ 0
EU548047.1 --
------ 0
EU548050.1 --
------ 0
EU548048.1 --
------ 0
EU548049.1 --
------ 0
AF207722.1 --
------ 0
EU548037.1 --
------ 0
AF207723.1 --
------ 0
EU548038.1 --
------ 0
EU548036.1 --
------ 0
EU548035.1 --
------ 0
AF207720.1 --
------ 0
AB601576.1 --
------ 0
EU548040.1 --
------ 0
EU548039.1 --
------ 0
AF207721.1 --
------ 0

AF207724.1 --
------ 0
JX982499.1 --
------ 0
EU548041.1 --
------ 0
EU548045.1 --
------ 0
AF207725.1 --
------ 0
EU548046.1 --
------ 0
AF207714.1 --
------ 337
AF207713.1 --
------ 337
AF207712.1 --
------ 337
AY750628.1 --
------ 1044
EF689084.1
CTAACACTATTAGTACTATTCTCACCTGACCTATTAGGAGACCCAGACAACTACATCCCC 774
EF689085.1
CTAACACTATTAGTACTATTCTCACCTGACCTATTAGGAGACCCAGACAACTACATCCCC 774
AB119070.1 --
------ 956
EF987742.1
CTAACACTATTAGTACTATTCTCACCTGACCTATTAGGAGACCCAGACAACTACATCCCC 774
AB026105.1
CTAACACTATTAGTACTATTCTCACCTGACCTATTAGGAGACCCAGACAACTACATCCCC 774
MW148603.1
CTAACACTATTAGTACTATTCTCACCTGACCTATTAGGAGACCCAGACAACTACATCCCC 14940
AB051263.1 --
------ 402
AF068544.1 --
------ 337

JX982502.1 --
------ 0
JX982501.1 --
------ 0
JX982498.1 --
------ 0
JX982497.1 --
------ 0
JX982495.1 --
------ 0
JX982496.1 --
------ 0
JX982500.1 --
------ 0
EU548051.1 --
------ 0
EU548044.1 --
------ 0
EU548042.1 --
------ 0
EU548043.1 --
------ 0
EU548047.1 --
------ 0
EU548050.1 --
------ 0
EU548048.1 --
------ 0

EU548049.1 --
------ 0
AF207722.1 --
------ 0
EU548037.1 --
------ 0
AF207723.1 --
------ 0
EU548038.1 --
------ 0
EU548036.1 --
------ 0
EU548035.1 --
------ 0
AF207720.1 --
------ 0
AB601576.1 --
------ 0
EU548040.1 --
------ 0
EU548039.1 --
------ 0
AF207721.1 --
------ 0
AF207724.1 --
------ 0
JX982499.1 --
------ 0
EU548041.1 --
------ 0
EU548045.1 --
------ 0
AF207725.1 --
------ 0
EU548046.1 --
------ 0
AF207714.1 --
------ 337
AF207713.1 --
------ 337
AF207712.1 --
------ 337
AY750628.1 --
------ 1044
EF689084.1
GCTAATCCCCTCAACACACCTCCCCACATTAAACCCGAGTGATACTTCCTATTCGCGTAT 834
EF689085.1
GCTAATCCCCTCAACACACCTCCCCACATTAAACCCGAGTGATACTTCCTATTCGCGTAT 834
AB119070.1 --
------ 956
EF987742.1
GCTAATCCCCTCAACACACCTCCCCACATTAAACCCGAGTGATACTTCCTATTCGCGTAT 834
AB026105.1
GCTAATCCCCTCAACACACCTCCCCACATTAAACCCGAGTGATACTTCCTATTCGCGTAT 834
MW148603.1
GCTAATCCCCTCAACACACCTCCCCACATTAAACCCGAGTGATACTTCCTATTCGCGTAT 15000
AB051263.1 --
------ 402
AF068544.1 --
------ 337

JX982502.1 --
------ 0
JX982501.1 --
------ 0

JX982498.1 --
------ 0
JX982497.1 --
------ 0
JX982495.1 --
------ 0
JX982496.1 --
------ 0
JX982500.1 --
------ 0
EU548051.1 --
------ 0
EU548044.1 --
------ 0
EU548042.1 --
------ 0
EU548043.1 --
------ 0
EU548047.1 --
------ 0
EU548050.1 --
------ 0
EU548048.1 --
------ 0
EU548049.1 --
------ 0
AF207722.1 --
------ 0
EU548037.1 --
------ 0
AF207723.1 --
------ 0
EU548038.1 --
------ 0
EU548036.1 --
------ 0
EU548035.1 --
------ 0
AF207720.1 --
------ 0
AB601576.1 --
------ 0
EU548040.1 --
------ 0
EU548039.1 --
------ 0
AF207721.1 --
------ 0
AF207724.1 --
------ 0
JX982499.1 --
------ 0
EU548041.1 --
------ 0
EU548045.1 --
------ 0
AF207725.1 --
------ 0
EU548046.1 --
------ 0
AF207714.1 --
------ 337
AF207713.1 --
------ 337
AF207712.1 --
------ 337

AY750628.1 --
------ 1044
EF689084.1
GCTATTCTGCGATCCATCCCCAACAAATTAGGAGGGGTACTAGCCCTAATCCTCTCCATT 894
EF689085.1
GCTATTCTGCGATCCATCCCCAACAAATTAGGAGGGGTACTAGCCCTAATCCTCTCCATT 894
AB119070.1 --
------ 956
EF987742.1
GCTATTCTGCGATCCATCCCCAACAAATTAGGAGGGGTACTAGCCCTAATCCTCTCCATT 894
AB026105.1
GCTATTCTGCGATCCATCCCCAACAAATTAGGAGGGGTACTAGCCCTAATCCTCTCCATT 894
MW148603.1
GCTATTCTGCGATCCATCCCCAACAAATTAGGAGGGGTACTAGCCCTAATCCTCTCCATT 15060
AB051263.1 --
------ 402
AF068544.1 --
------ 337

JX982502.1 --
------ 0
JX982501.1 --
------ 0
JX982498.1 --
------ 0
JX982497.1 --
------ 0
JX982495.1 --
------ 0
JX982496.1 --
------ 0
JX982500.1 --
------ 0
EU548051.1 --
------ 0
EU548044.1 --
------ 0
EU548042.1 --
------ 0
EU548043.1 --
------ 0
EU548047.1 --
------ 0
EU548050.1 --
------ 0
EU548048.1 --
------ 0
EU548049.1 --
------ 0
AF207722.1 --
------ 0
EU548037.1 --
------ 0
AF207723.1 --
------ 0
EU548038.1 --
------ 0
EU548036.1 --
------ 0
EU548035.1 --
------ 0
AF207720.1 --
------ 0
AB601576.1 --
------ 0

EU548040.1 --
------ 0
EU548039.1 --
------ 0
AF207721.1 --
------ 0
AF207724.1 --
------ 0
JX982499.1 --
------ 0
EU548041.1 --
------ 0
EU548045.1 --
------ 0
AF207725.1 --
------ 0
EU548046.1 --
------ 0
AF207714.1 --
------ 337
AF207713.1 --
------ 337
AF207712.1 --
------ 337
AY750628.1 --
------ 1044
EF689084.1
CTAATCCTAGCCATCATCCCCCTACTCCATACCTCAAAACAACGAAGCATAATATTCCGC 954
EF689085.1
CTAATCCTAGCCATCATCCCCCTACTCCATACCTCAAAACAACGAAGCATAATATTCCGC 954
AB119070.1 --
------ 956
EF987742.1
CTAATCCTAGCCATCATCCCCCTACTCCATACCTCAAAACAACGAAGCATAATATTCCGC 954
AB026105.1
CTAATCCTAGCCATCATCCCCCTACTCCATACCTCAAAACAACGAAGCATAATATTCCGC 954
MW148603.1
CTAATCCTAGCCATCATCCCCCTACTCCATACCTCAAAACAACGAAGCATAATATTCCGC 15120
AB051263.1 --
------ 402
AF068544.1 --
------ 337

JX982502.1 --
------ 0
JX982501.1 --
------ 0
JX982498.1 --
------ 0
JX982497.1 --
------ 0
JX982495.1 --
------ 0
JX982496.1 --
------ 0
JX982500.1 --
------ 0
EU548051.1 --
------ 0
EU548044.1 --
------ 0
EU548042.1 --
------ 0
EU548043.1 --
------ 0

```
    EU548047.1       ------------------------------------------------------
------ 0
    EU548050.1       ------------------------------------------------------
------ 0
    EU548048.1       ------------------------------------------------------
------ 0
    EU548049.1       ------------------------------------------------------
------ 0
    AF207722.1       ------------------------------------------------------
------ 0
    EU548037.1       ------------------------------------------------------
------ 0
    AF207723.1       ------------------------------------------------------
------ 0
    EU548038.1       ------------------------------------------------------
------ 0
    EU548036.1       ------------------------------------------------------
------ 0
    EU548035.1       ------------------------------------------------------
------ 0
    AF207720.1       ------------------------------------------------------
------ 0
    AB601576.1       ------------------------------------------------------
------ 0
    EU548040.1       ------------------------------------------------------
------ 0
    EU548039.1       ------------------------------------------------------
------ 0
    AF207721.1       ------------------------------------------------------
------ 0
    AF207724.1       ------------------------------------------------------
------ 0
    JX982499.1       ------------------------------------------------------
------ 0
    EU548041.1       ------------------------------------------------------
------ 0
    EU548045.1       ------------------------------------------------------
------ 0
    AF207725.1       ------------------------------------------------------
------ 0
    EU548046.1       ------------------------------------------------------
------ 0
    AF207714.1       ------------------------------------------------------
------ 337
    AF207713.1       ------------------------------------------------------
------ 337
    AF207712.1       ------------------------------------------------------
------ 337
    AY750628.1       ------------------------------------------------------
------ 1044
    EF689084.1
CCACTAAGTCAATGCTTATTCTGACTACTAGTAGCTGACCTCCTTACCCTAACCTGAATC   1014
    EF689085.1
CCACTAAGTCAATGCTTATTCTGACTACTAGTAGCTGACCTCCTTACCCTAACCTGAATC   1014
    AB119070.1       ------------------------------------------------------
------ 956
    EF987742.1
CCACTAAGTCAATGCTTATTCTGACTGCTAGTAGCTGACCTCCTTACCCTAACCTGAATC   1014
    AB026105.1
CCACTAAGTCAATGCTTATTCTGACTGCTAGTAGCTGACCTCCTTACCCTAACCTGAATC   1014
    MW148603.1
CCACTAAGTCAATGCTTATTCTGACTGCTAGTAGCTGACCTCCTTACCCTAACCTGAATC   15180
    AB051263.1       ------------------------------------------------------
------ 402
    AF068544.1       ------------------------------------------------------
------ 337
```

```
    JX982502.1          ------------------------------------------------------
------ 0
    JX982501.1          ------------------------------------------------------
------ 0
    JX982498.1          ------------------------------------------------------
------ 0
    JX982497.1          ------------------------------------------------------
------ 0
    JX982495.1          ------------------------------------------------------
------ 0
    JX982496.1          ------------------------------------------------------
------ 0
    JX982500.1          ------------------------------------------------------
------ 0
    EU548051.1          ------------------------------------------------------
------ 0
    EU548044.1          ------------------------------------------------------
------ 0
    EU548042.1          ------------------------------------------------------
------ 0
    EU548043.1          ------------------------------------------------------
------ 0
    EU548047.1          ------------------------------------------------------
------ 0
    EU548050.1          ------------------------------------------------------
------ 0
    EU548048.1          ------------------------------------------------------
------ 0
    EU548049.1          ------------------------------------------------------
------ 0
    AF207722.1          ------------------------------------------------------
------ 0
    EU548037.1          ------------------------------------------------------
------ 0
    AF207723.1          ------------------------------------------------------
------ 0
    EU548038.1          ------------------------------------------------------
------ 0
    EU548036.1          ------------------------------------------------------
------ 0
    EU548035.1          ------------------------------------------------------
------ 0
    AF207720.1          ------------------------------------------------------
------ 0
    AB601576.1          ------------------------------------------------------
------ 0
    EU548040.1          ------------------------------------------------------
------ 0
    EU548039.1          ------------------------------------------------------
------ 0
    AF207721.1          ------------------------------------------------------
------ 0
    AF207724.1          ------------------------------------------------------
------ 0
    JX982499.1          ------------------------------------------------------
------ 0
    EU548041.1          ------------------------------------------------------
------ 0
    EU548045.1          ------------------------------------------------------
------ 0
    AF207725.1          ------------------------------------------------------
------ 0
    EU548046.1          ------------------------------------------------------
------ 0
```

AF207714.1 --
------ 337
AF207713.1 --
------ 337
AF207712.1 --
------ 337
AY750628.1 --
------ 1044
EF689084.1
GGCGGCCAACCAGTAGAACACCCATTCATCATTATCGGCCAACTAGCCTCAATCCTCTAC 1074
EF689085.1
GGCGGCCAACCAGTAGAACACCCATTCATCATTATCGGCCAACTAGCCTCAATCCTCTAC 1074
AB119070.1 --
------ 956
EF987742.1
GGCGGCCAACCAGTAGAACACCCATTCATCATTATCGGCCAACTAGCCTCAATCCTCTAC 1074
AB026105.1
GGCGGCCAACCAGTAGAACACCCATTCATCATTATCGGCCAACTAGCCTCAATCCTCTAC 1074
MW148603.1
GGCGGCCAACCAGTAGAACACCCGTTCATCATTATCGGCCAACTAGCCTCAATCCTCTAC 15240
AB051263.1 --
------ 402
AF068544.1 --
------ 337

JX982502.1 --
------ 0
JX982501.1 --
------ 0
JX982498.1 --
------ 0
JX982497.1 --
------ 0
JX982495.1 --
------ 0
JX982496.1 --
------ 0
JX982500.1 --
------ 0
EU548051.1 -------------------
TTATACCTATTATCAGCATTATCGAAAATAACATATTAAAA 41
EU548044.1 -------------------
TTATACCTATTACCAGCATTATCGAAAATAACATATTAAAA 41
EU548042.1 -------------------
TTATACCTATTACCAGCATTATCGAAAATAACATATTAAAA 41
EU548043.1 -------------------
TTATACCTATTACCAGCATTATCGAAAATAACATATTAAAA 41
EU548047.1 -------------------
TTATACCTATTATCAGCATTATCGAAAATAACATATTAAAA 41
EU548050.1 -------------------
TTATACCTATTATCAGCATTATCGAAAATAACATATTAAAA 41
EU548048.1 -------------------
TTATACCTATTATCAGCATTATCGAAAATAACATATTAAAA 41
EU548049.1 -------------------
TTATACCTATTATCAGCATTATCGAAAATAACATATTAAAA 41
AF207722.1 --
------ 0
EU548037.1 -------------------
TTATACCTATTATCAGCATTATCGAAAATAACATATTAAAA 41
AF207723.1 --
------ 0
EU548038.1 -------------------
TTATACCTATTATCAGCATTATCGAAAATAACATATTAAAA 41
EU548036.1 -------------------
TTATACCTATTATCAGCATTATCGAAAATAACATATTAAAA 41

EU548035.1 -------------------
TTATACCTATTATCAGCATTATCGAAAATAACATATTAAAA 41
AF207720.1 --
------ 0
AB601576.1 --
------ 0
EU548040.1 -------------------
TTATACCTATTATCAGCATTATCGAAAATAACATATTAAAA 41
EU548039.1 -------------------
TTATACCTATTATCAGCATTATCGAAAATAACATATTAAAA 41
AF207721.1 --
------ 0
AF207724.1 --
------ 0
JX982499.1 --
------ 0
EU548041.1 -------------------
TTATACCTATTATCAGCATTATCGAAAATAACATATTAAAA 41
EU548045.1 -------------------
TTATACCTATTATCAGCATTATCGAAAATAACATATTAAAA 41
AF207725.1 --
------ 0
EU548046.1 -------------------
TTATACCTATTATCAGCATTATCGAAAATAACATATTAAAA 41
AF207714.1 --
------ 337
AF207713.1 --
------ 337
AF207712.1 --
------ 337
AY750628.1 --
------ 1044
EF689084.1
TTCATGATTCTCCTAGTCCTTATACCTATTATCAGCATTATCGAAAATAACATATTAAAA 1134
EF689085.1
TTCATGATTCTCCTAGTCCTTATACCTATTATCAGCATTATCGAAAATAACATATTAAAA 1134
AB119070.1 --
------ 956
EF987742.1
TTCATGATTCTCCTAGTCCTTATACCTATTATCAGCATTATCGAAAATAACATATTAAAA 1134
AB026105.1
TTCATGATTCTCCTAGTCCTTATACCTATTATCAGCATTATCGAAAATAACATATTAAAA 1134
MW148603.1
TTCATGATTCTCCTAGTCCTTATACCTATTATCAGCATTATCGAAAATAACATATTAAAA 15300
AB051263.1 --
------ 402
AF068544.1 --
------ 337

JX982502.1 --
------ 0
JX982501.1 --
------ 0
JX982498.1 --
------ 0
JX982497.1 --
------ 0
JX982495.1 --
------ 0
JX982496.1 --
------ 0
JX982500.1 --
------ 0
EU548051.1
TGAAGAGTCTTTGTAGTATAATAATTACTTTGGTCTTGTAAACCAAAAATGGAGAATCCC 101

```
   EU548044.1
TGAAGAGTCTTTGTAGTATAATAATTACTTTGGTCTTGTAAACCAAAAATGGAGAATCCC   101
   EU548042.1
TGAAGAGTCTTTGTAGTATAATAATTACTTTGGTCTTGTAAACCAAAAATGGAGAATCCC   101
   EU548043.1
TGAAGAGTCTTTGTAGTATAATAATTACTTTGGTCTTGTAAACCAAAAATGGAGAATCCC   101
   EU548047.1
TGAAGAGTCTTTGTAGTATAATAATTACTTTGGTCTTGTAAACCAAAAATGGAGAATCCC   101
   EU548050.1
TGAAGAGTCTTTGTAGTATAATAATTACTTTGGTCTTGTAAACCAAAAATGGAGAATCCC   101
   EU548048.1
TGAAGAGTCTTTGTAGTATAATAATTACTTTGGTCTTGTAAACCAAAAATGGAGAATCCC   101
   EU548049.1
TGAAGAGTCTTTGTAGTATAATAATTACTTTGGTCTTGTAAACCAAAAATGGAGAATCCC   101
   AF207722.1      ------------------------------------------------------
-----C 1
   EU548037.1
TGAAGAGTCTTTGTAGTATAATAATTACTTTGGTCTTGTAAACCAAAAATGGAGAATCCC   101
   AF207723.1      ------------------------------------------------------
-----C 1
   EU548038.1
TGAAGAGTCTTTGTAGTATAATAATTACTTTGGTCTTGTAAACCAAAAATGGAGAATCCC   101
   EU548036.1
TGAAGAGTCTTTGTAGTATAATAATTACTTTGGTCTTGTAAACCAAAAATGGAGAATCCC   101
   EU548035.1
TGAAGAGTCTTTGTAGTATAATAATTACTTTGGTCTTGTAAACCAAAAATGGAGAATCCC   101
   AF207720.1      ------------------------------------------------------
-----C 1
   AB601576.1      ------------------------------------------------------
------ 0
   EU548040.1
TGAAGAGTCTTTGTAGTATAATAATTACTTTGGTCTTGTAAACCAAAAATGGAGAATCCC   101
   EU548039.1
TGAAGAGTCTTTGTAGTATAATAATTACTTTGGTCTTGTAAACCAAAAATGGAGAATCCC   101
   AF207721.1      ------------------------------------------------------
-----C 1
   AF207724.1      ------------------------------------------------------
-----C 1
   JX982499.1      ------------------------------------------------------
------ 0
   EU548041.1
TGAAGAGTCTTTGTAGTATAATAATTACTTTGGTCTTGTAAACCAAAAATGGAGAATCCC   101
   EU548045.1
TGAAGAGTCTTTGTAGTATAATAATTACTTTGGTCTTGTAAACCAAAAATGGAGAATCCC   101
   AF207725.1      ------------------------------------------------------
-----C 1
   EU548046.1
TGAAGAGTCTTTGTAGTATAATAATTACTTTGGTCTTGTAAACCAAAAATGGAGAATCCC   101
   AF207714.1      ------------------------------------------------------
------ 337
   AF207713.1      ------------------------------------------------------
------ 337
   AF207712.1      ------------------------------------------------------
------ 337
   AY750628.1      ------------------------------------------------------
------ 1044
   EF689084.1      TGAAGA------------------------------------------------
------ 1140
   EF689085.1      TGAAGA------------------------------------------------
------ 1140
   AB119070.1      ------------------------------------------------------
------ 956
   EF987742.1      TGAAGA------------------------------------------------
------ 1140
   AB026105.1      TGAAGA------------------------------------------------
------ 1140
```

MW148603.1
TGAAGAGTCTTTGTAGTATAATAATTACTTTGGTCTTGTAAACCAAAAATGGAGAATCCC 15360
AB051263.1 --
------ 402
AF068544.1 --
------ 337

JX982502.1 --
------ 0
JX982501.1 --
------ 0
JX982498.1 --
------ 0
JX982497.1 --
------ 0
JX982495.1 --
------ 0
JX982496.1 --
------ 0
JX982500.1 --
------ 0
EU548051.1
ATCTCCCTAAGACTCAAGGAAGAAGCAACAGCCCCTCCATCAGCACCCAAAGCTGATATT 161
EU548044.1
ATCTCCCTAAGACTCAAGGAAGAAGCAACAGCCCCGCCATCAGCACCCAAAGCTGATATT 161
EU548042.1
ATCTCCCTAAGACTCAAGGAAGAAGCAACAGCCCCGCCATCAGCACCCAAAGCTGATATT 161
EU548043.1
ATCTCCCTAAGACTCAAGGAAGAAGCAACAGCCCCGCCATCAGCACCCAAAGCTGATATT 161
EU548047.1
ATCTCCCTAAGACTCAAGGAAGAAGCAACAGCCCCGCCATCAGCACCCAAAGCTGATATT 161
EU548050.1
ATCTCCCTAAGACTCAAGGAAGAAGCAACAGCCCCGCCATCAGCACCCAAAGCTGATATT 161
EU548048.1
ATCTCCCTAAGACTCAAGGAAGAAGCAACAGCCCCGCCATCAGCACCCAAAGCTGATATT 161
EU548049.1
ATCTCCCTAAGACTCAAGGAAGAAGCAACAGCCCCGCCATCAGCACCCAAAGCTGATATT 161
AF207722.1
ATCTCCCTAAGACTCAAGGAAGAAGCAACAGCCCCGCCATCAGCACCCAAAGCTGATATT 61
EU548037.1
ATCTCCCTAAGACTCAAGGAAGAAGCAACAGCCCCGCCATCAGCACCCAAAGCTGATATT 161
AF207723.1
ATCTCCCTAAGACTCAAGGAAGAAGCAACAGCCCCGCCATCAGCACCCAAAGCTGATATT 61
EU548038.1
ATCTCCCTAAGACTCAAGGAAGAAGCAACAGCCCCGCCATCAGCACCCAAAGCTGATATT 161
EU548036.1
ATCTCCCTAAGACTCAAGGAAGAAGCAACAGCCCCGCCATCAGCACCCAAAGCTGATATT 161
EU548035.1
ATCTCCCTAAGACTCAAGGAAGAAGCAACAGCCCCGCCATCAGCACCCAAAGCTGATATT 161
AF207720.1
ATCTCCCTAAGACTCAAGGAAGAAGCAACAGCCCCGCCATCAGCACCCAAAGCTGATATT 61
AB601576.1 --
------ 0
EU548040.1
ATCTCCCTAAGACTCAAGGAAGAAGCAACAGCCCCGCCATCAGCACCCAAAGCTGATATT 161
EU548039.1
ATCTCCCTAAGACTCAAGGAAGAAGCAACAGCCCCGCCATCAGCACCCAAAGCTGATATT 161
AF207721.1
ATCTCCCTAAGACTCAAGGAAGAAGCAACAGCCCCGCCATCAGCACCCAAAGCTGATATT 61
AF207724.1
ATCTCCCTAAGACTCAAGGAAGAAGCAACAGCCCCGCCATCAGCACCCAAAGCTGATATT 61
JX982499.1 --
------ 0
EU548041.1
ATCTCCCTAAGACTCAAGGAAGAAGCAACAGCCCCGCCATCAGCACCCAAAGCTGATATT 161

EU548045.1
ATCTCCCTAAGACTCAAGGAAGAAGCAACAGCCCCGCCATCAGCACCCAAAGCTGATATT 161
AF207725.1
ATCTCCCTAAGACTCAAGGAAGAAGCAACAGCCCCGCCATCAGCACCCAAAGCTGATATT 61
EU548046.1
ATCTCCCTAAGACTCAAGGAAGAAGCAACAGCCCCGCCATCAGCACCCAAAGCTGATATT 161
AF207714.1 --
------ 337
AF207713.1 --
------ 337
AF207712.1 --
------ 337
AY750628.1 --
------ 1044
EF689084.1 --
------ 1140
EF689085.1 --
------ 1140
AB119070.1 --
------ 956
EF987742.1 --
------ 1140
AB026105.1 --
------ 1140
MW148603.1
ATCTCCCTAAGACTCAAGGAAGAAGCAACAGCCCCGCCATCAGCACCCAAAGCTGATATT 15420
AB051263.1 --
------ 402
AF068544.1 --
------ 337

JX982502.1 ---
TTTAACAATAT 11
JX982501.1 ---
GTTAACAATAT 11
JX982498.1 ---
TTTAACAATAT 11
JX982497.1 ---
TTTAACAATAT 11
JX982495.1 ---
TTTAACAATAT 11
JX982496.1 ---
TTTAACAATAT 11
JX982500.1 ---
TTTAACAATAT 11
EU548051.1
CTAACTAAACTATTCCCTGATTTTCTCACCATAACTCTCTATCCATATACTTAACAATAT 221
EU548044.1
CTAACTAAACTATTCCCTGATTTTCTCACCATAACTCTCTATTCATATATTTAACAATAT 221
EU548042.1
CTAACTAAACTATTCCCTGATTTTCTCACCATAACTCTCTATTCATATATTTAACAATAT 221
EU548043.1
CTAACTAAACTATTCCCTGATTTTCTCACCATAACTCTCTATTCATATATTTAACAATAT 221
EU548047.1
CTAACTAAACTATTCCCTGATTTTCTCACCATAACTCTCTATTCATATATTTAACAATAT 221
EU548050.1
CTAACTAAACTATTCCCTGATTTTCTCACCATAACTCTCTATTCATATATTTAACAATAT 221
EU548048.1
CTAACTAAACTATTCCCTGATTTTCTCACCATAACTCTCTATTCATATATTTAACAATAT 221
EU548049.1
CTAACTAAACTATTCCCTGATTTTCTCACCATAACTCTCTATTCATATATTTAACAATAT 221
AF207722.1
CTAACTAAACTATTCCCTGATTTTCTCACCATAACTCTCTATTCATATATTTAACAATAT 121
EU548037.1
CTAACTAAACTATTCCCTGATTTTCTCACCATAACTCTCTATTCATATATTTAACAATAT 221

AF207723.1
CTAACTAAACTATTCCCTGATTTTCTCACCATAACTCTCTATTCATATATTTAACAATAT   121
EU548038.1
CTAACTAAACTATTCCCTGATTTTCTCACCATAACTCTCTATTCATATATTTAACAATAT   221
EU548036.1
CTAACTAAACTATTCCCTGATTTTCTCACCATAACTCTCTATTCATATATTTAACAATAT   221
EU548035.1
CTAACTAAACTATTCCCTGATTTTCTCACCATAACTCTCTATTCATATATTTAACAATAT   221
AF207720.1
CTAACTAAACTATTCCCTGATTTTCTCACCATAACTCTCTATTCATATATTTAACAATAT   121
AB601576.1 -----------
ATTCCCTGATTTTCTCACCATAACTCTCTATTCATATATTTAACAATAT   49
EU548040.1
CTAACTAAACTATTCCCTGATTTTCTCACCATAACTCTCTATTCATATATTTAACAATAT   221
EU548039.1
CTAACTAAACTATTCCCTGATTTTCTCACCATAACTCTCTATTCATATATTTAGCAATAT   221
AF207721.1
CTAACTAAACTATTCCCTGATTTTCTCACCATAACTCTCTATTCATATATTTAGCAATAT   121
AF207724.1
CTAACTAAACTATTCCCTGATTTTCTCACCATAACTCTCTATCCATATACTTAACAATAT   121
JX982499.1 ---
TTTAGCAATAT   11
EU548041.1
CTAACTAAACTATTCCCTGATTTTCTCACCATAACTCTCTATTCATATATTTAACAATAT   221
EU548045.1
CTAACTAAACTATTCCCTGATTTTCTCACCATAACTCTCTATTCATATATTTAACAATAT   221
AF207725.1
CTAACTAAACTATTCCCTGATTTTCTCACCATAACTCTCTATTCATATATTTAACAATAT   121
EU548046.1
CTAACTAAACTATTCCCTGATTTTCTCACCATAACTCTCTATTCATATATTTAACAATAT   221
AF207714.1 --
------ 337
AF207713.1 --
------ 337
AF207712.1 --
------ 337
AY750628.1 --
------ 1044
EF689084.1 --
------ 1140
EF689085.1 --
------ 1140
AB119070.1 --
------ 956
EF987742.1 --
------ 1140
AB026105.1 --
------ 1140
MW148603.1
CTAACTAAACTATTCCCTGATTTTCTCACCATAACTCTCTATTCATATATTTAACAATAT   15480
AB051263.1 --
------ 402
AF068544.1 --
------ 337

JX982502.1 CTAATGTGCTTGCCCGGTATGTATTTCTTTTT--
TTTTCCCCCCATGTACTTCGTGCATT   69
JX982501.1 CTAATGTACTTGCCCGGTATGTATTTCTTTTT--
TCTCCCCCCCATGTACTTCGTGCATT   69
JX982498.1 CTAATGTGCTTGCCCGGTATGTATTTCTTTTT--
TTTCCCCCCCATGTACTTCGTGCATT   69
JX982497.1 CTAATGTACTTGCCCGGTATGTATTTCTTTTT--
TCTCCCCCCCATGTACTTCGTGCATT   69
JX982495.1 CTAATGTGGTTGCCCGGTATGTATTTCTTTTT--
TTTCCCCCCCATGTACTTCGTGCATT   69

JX982496.1 CTAATGTGCTTGCCCGGTATGTATTTCTTTTT--
TTTCCCCCCCATGTACTTCGTGCATT 69
JX982500.1 CTAATGTGCTTGCCCGGTATGTATTTCTTTTT--
TTTCCCCCCCATGTACTTCGTGCATT 69
EU548051.1 CTAATGTGCTTGCCCGGTATGTATTTCTTTT---
TTTCCCCCCTATGTACTTCGTGCATT 278
EU548044.1 CTAATGTGCTTGCCCGGTATGTATTTCTTTT---
TTTCCCCCCTATGTACTTCGTGCATT 278
EU548042.1 CTAATGTGCTTGCCCGGTATGTATTTCTTTT---
TTTCCCCCCTATGTACTTCGTGCATT 278
EU548043.1 CTAATGTGCTTGCCCGGTATGTATTTCTTTT---
TTTCCCCCCTATGTACTTCGTGCATT 278
EU548047.1 CTAATGTGCTTGCCCGGTATGTATTTCTTTTT--
TTTCCCCCCCATGTACTTCGTGCATT 279
EU548050.1 CTAATGTGCTTGCCCGGTATGTATTTCTTTTT--
TTTCCCCCCCATGTACTTCGTGCATT 279
EU548048.1 CTAATGTGCTTGCCCGGTATGTATTTCTTTTT--
TTTCCCCCCCATGTACTTCGTGCATT 279
EU548049.1 CTAATGTGCTTGCCCGGTATGTATTTCTTTTT--
TTTCCCCCCCATGTACTTCGTGCATT 279
AF207722.1 CTAATGTGCTTGCCCGGTATGTATTTCTTTTT--
TTTTCCCCCCATGTACTTCGTGCATT 179
EU548037.1 CTAATGTACTTGCCCGGTATGTATTTCTTTTT--
TCTCCCCCCCATGTACTTCGTGCATT 279
AF207723.1 CTAATGTACTTGCCCGGTATGTATTTCTTTTT--
TCTCCCCCCCATGTACTTCGTGCATT 179
EU548038.1 CTAATGTACTTGCCCGGTATGTATTTCTTTTT--
TTTCCCCCCCATGTACTTCGTGCATT 279
EU548036.1 CTAATGTGCTTGCCCGGTATGTATTTCTTTTT--
TTTCCCCCCCATGTACTTCGTGCATT 279
EU548035.1 CTAATGTGCTTGCCCGGTATGTATTTCTTTTT--
TTTCCCCCCCATGTACTTCGTGCATT 279
AF207720.1 CTAATGTGCTTGCCCGGTATGTATTTCTTTTT--
TTTCCCCCCCATGTACTTCGTGCATT 179
AB601576.1
CTAATGTACTTGCCCGGTATGTATTTCTTTTTTTTTCCCCCCCATGTACTTCGTGCATT 109
EU548040.1 CTAATGTACTTGCCCGGTATGTATTTCTTTTTT-
TTTCCCCCCCATGTACTTCGTGCATT 280
EU548039.1 CTAATGTACTTGCCCGGTATGTATTTCTTTTTT-
TTTCCCCCCCATGTACTTCGTGCATT 280
AF207721.1 CTAATGTACTTGCCCGGTATGTATTTCTTTTTT-
TTTCCCCCCCATGTACTTCGTGCATT 180
AF207724.1 CTAATGTGCTTGCCCGGTATGTATTTCTTTTTT-
TTTCCCCCCCATGTACTTCGTGCATT 180
JX982499.1 CTAATGTACTTGCCCGGTATGTATTTCTTTTTT-
TTTCCCCCCCATGTACTTCGTGCATT 70
EU548041.1 CTAATGTGCTTGCCCGGTATGTATTTCTTTTTT-
TTTCCCCCCCATGTACTTCGTGCATT 280
EU548045.1 CTAATGTGCTTGCCCGGTATGTATTTCTTTTTT-
TTTCCCCCCCATGTACTTCGTGCATT 280
AF207725.1 CTAATGTGCTTGCCCGGTATGTATTTCTTTTTT-
TTTCCCCCCCATGTACTTCGTGCATT 180
EU548046.1 CTAATGTGCTTGCCCGGTATGTATTTCTTTTTT-
TTTCCCCCCCATGTACTTCGTGCATT 280
AF207714.1 --
------ 337
AF207713.1 --
------ 337
AF207712.1 --
------ 337
AY750628.1 --
------ 1044
EF689084.1 --
------ 1140
EF689085.1 --
------ 1140

AB119070.1 -- 956
EF987742.1 -- 1140
AB026105.1 -- 1140
MW148603.1 CTAATGTGCTTGCCCGGTATGTATTTCTTTTTT-TTTCCCCCCCATGTACTTCGTGCATT 15539
AB051263.1 -- 402
AF068544.1 -- 337

JX982502.1 ACTGGTTTGCCCCATGCATATAAGCATGTATATATTATGGTTGATTTTACATGTATCCAC 129
JX982501.1 ACTGGTTTGCCCCATGCATATAAGCATGTATATATTATGGTTGATTTTACATGAATCCAC 129
JX982498.1 ACTGGTTTGCCCCATGCATATAAGCATGTATATATTATGATTGATTTTACATGTATCCAT 129
JX982497.1 ACTGGTTTGCCCCATGCATATAAGCATGTATATATTATGGTTGATTTTACATGTATCCAC 129
JX982495.1 ACTGGTTTGCCCCATGCATATAAGCATGTATATATTATGGTTGATTTTACATGTATCCAC 129
JX982496.1 ACTGGTTTGCCCCATGCATATAAGCATGTATATATTATGGTTGATTTTACATGTATCCAC 129
JX982500.1 ACTGGTTTGCCCCATGCATATAAGCATGTATATATTATGGTTGATTTTACATGTATCCAC 129
EU548051.1 ACTGGTTTGCCCCATGCATATAAGCATGTATATATTATGGTTGATTTTACATGTATCCAC 338
EU548044.1 ACTGGTTTGCCCCATGCATATAAGCATGTATATATTATGGTTGATTTTACATGTATCCAC 338
EU548042.1 ACTGGTTTGCCCCATGCATATAAGCATGTATATATTATGGTTGATTTTACATGTATCCAC 338
EU548043.1 ACTGGTTTGCCCCATGCATATAAGCATGTATATATTATGGTTGATTTTACATGTATCCAC 338
EU548047.1 ACTGGTTTGCCCCATGCATATAAGCATGTATATATTATGATTGATTTTACATGTATCCAC 339
EU548050.1 ACTGGTTTGCCCCATGCATATAAGCATGTATATATTATGGTTGATTTTACATGTATCCAC 339
EU548048.1 ACTGGTTTGCCCCATGCATATAAGCATGTATATATTATGGTTGATTTTACATGTATCCAC 339
EU548049.1 ACTGGTTTGCCCCATGCATATAAGCATGTATATATTATGGTTGATTTTACATGTATCCAC 339
AF207722.1 ACTGGTTTGCCCCATGCATATAAGCATGTATATATTATGGTTGATTTTACATGTATCCAC 239
EU548037.1 ACTGGTTTGCCCCATGCATATAAGCATGTATATATTATGGTTGATTTTACATGTATCCAC 339
AF207723.1 ACTGGTTTGCCCCATGCATATAAGCATGTATATATTATGGTTGATTTTACATGTATCCAC 239
EU548038.1 ACTGGTTTGCCCCATGCATATAAGCATGTATATATTATGGTTGATTTTACATGTATCCAC 339
EU548036.1 ACTGGTTTGCCCCATGCATATAAGCATGTATATATTATGGTTGATCTTACATGTATCCAC 339
EU548035.1 ACTGGTTTGCCCCATGCATATAAGCATGTATATATTATGGTTGATTTTACATGTATCCAC 339
AF207720.1 ACTGGTTTGCCCCATGCATATAAGCATGTATATATTATGGTTGATTTTACATGTATCCAC 239
AB601576.1 ACTGGTTTGCCCCATGCATATAAGCATGTATATATTATGGTTGATTTTACATGTATCCAC 169
EU548040.1 ACTGGTTTGCCCCATGCATATAAGCATGTATATATTATGGTTGATTTTACATGTATCCAC 340
EU548039.1 ACTGGTTTGCCCCATGCATATAAGCATGTATATATTATGGTTGATTTTACATGTATCCAC 340
AF207721.1 ACTGGTTTGCCCCATGCATATAAGCATGTATATATTATGGTTGATTTTACATGTATCCAC 240

```
    AF207724.1
ACTGGTTTGCCCCATGCATATAAGCATGTATATATTATGGTTGATTTTACATGTATCCAC   240
    JX982499.1
ACTGGTTTGCCCCATGCATATAAGCATGTATATATTATGGTTGATTTTACATGTATCCAC   130
    EU548041.1
ACTGGTTTGCCCCATGCATATAAGCATGTATATATTATGGTTGATTTTACATGTATCCAC   340
    EU548045.1
ACTGGTTTGCCCCATGCATATAAGCATGTATATATTATGGTTGATTTTACATGTATCCAC   340
    AF207725.1
ACTGGTTTGCCCCATGCATATAAGCATGTATATATTATGGTTGATTTTACATGTATCCAC   240
    EU548046.1
ACTGGTTTGCCCCATGCATATAAGCATGTATATATTATGATTGATTTTACATGTATCCAT   340
    AF207714.1      ------------------------------------------------------
------ 337
    AF207713.1      ------------------------------------------------------
------ 337
    AF207712.1      ------------------------------------------------------
------ 337
    AY750628.1      ------------------------------------------------------
------ 1044
    EF689084.1      ------------------------------------------------------
------ 1140
    EF689085.1      ------------------------------------------------------
------ 1140
    AB119070.1      ------------------------------------------------------
------ 956
    EF987742.1      ------------------------------------------------------
------ 1140
    AB026105.1      ------------------------------------------------------
------ 1140
    MW148603.1
ACTGGTTTGCCCCATGCATATAAGCATGTATATATTATGGTTGATTTTACATGTATCCAC   15599
    AB051263.1      ------------------------------------------------------
------ 402
    AF068544.1      ------------------------------------------------------
------ 337


    JX982502.1
TTCACTTAGATCACGAGCTTTATCACCATGCCTCGAGAAACCATCAACCCTTGCCTGAAC   189
    JX982501.1
TTCACTTAGACCACGAACTTTATCACCATGCCTCGAGAAACCATCAACCCTTGCCTGAAC   189
    JX982498.1
TCCACTTAGACCACGAGCTTTATCACCATGCCTCGAGAAACCATCAACCCTTGCCTGAAC   189
    JX982497.1
TTCACTTAGACCACGAGCTTTATCACCATGCCTCGAGAAACCATCAACCCTTGCCTGAAC   189
    JX982495.1
TCCACTTAGACCACGAGCTTTATCACCATGCCTCGAGAAACCATCAACCCTTGCCTGAAC   189
    JX982496.1
TCCACTTAGACCACGAGCTTTATCACCATGCCTCGAGAAACCATCAACCCTTGCCTGAAC   189
    JX982500.1
TTCACTTAGACCACGAGCTTTATCACCATGCCTCGAGAAACCATCAACCCTTGCCTGAAC   189
    EU548051.1
TCCACTTAGATCACGAGCTTTATCACCATGCCTCGAGAAACCATCAACCCTTGCCTGAAC   398
    EU548044.1
TTCACTTAGACCACGAGCTTTATCACCATGCCTCGAGAAACCATCAACCCTTGCCTGAAC   398
    EU548042.1
TCCACTTAGACCACGAGCTTTATCACCATGCCTCGAGAAACCATCAACCCTTGCCTGAAC   398
    EU548043.1
TCCAGTTAGACCACGAGCTTTATCACCATGCCTCGAGAAACCATCAACCCTTGCCTGAAC   398
    EU548047.1
TCCACTTAGACCACGAGCTTTATCACCATGCCTCGAGAAACCATCAACCCTTGCCTGAAC   399
    EU548050.1
TCCACTTAGACCACGAGCTTTATCACCATGCCTCGAGAAACCATCAACCCTTGCCTGAAC   399
    EU548048.1
TCCACTTAGATCACGAGCTTTATCACCATGCCTCGAGAAACCATCAACCCTTGCCTGAAC   399
```

EU548049.1
TCCACTTAGATCACGAGCTTTATCACCATGCCTCGAGAAACCATCAACCCTTGCCTGAAC 399
AF207722.1
TTCACTTAGATCACGAGCTTTATCACCATGCCTCGAGAAACCATCAACCCTTGCCTGAAC 299
EU548037.1
TTCACTTAGACCACGAGCTTTATCACCATGCCTCGAGAAACCATCAACCCTTGCCTGAAC 399
AF207723.1
TTCACTTAGACCACGAGCTTTATCACCATGCCTCGAGAAACCATCAACCCTTGCCTGAAC 299
EU548038.1
TTCACTTAGACCACGAGCTTTATCACCATGCCTCGAGAAACCATCAACCCTTGCCTGAAC 399
EU548036.1
TTCACTTAGACCACGAGCTTTATCACCATGCCTCGAGAAACCATCAACCCTTGCCTGAAC 399
EU548035.1
TTCACTTAGACCACGAGCTTTATCACCATGCCTCGAGAAACCATCAACCCTTGCCTGAAC 399
AF207720.1
TTCACTTAGACCACGAGCTTTATCACCATGCCTCGAGAAACCATCAACCCTTGCCTGAAC 299
AB601576.1
TTCACTTAGACCACGAGCTTTATCACCATGCCTCGAGAAACCATCAACCCTTGCCTGAAC 229
EU548040.1
TTCACTTAGATCACGAGCTTTATCACCATGCCTCGAGAAACCATCAACCCTTGCCTGAAC 400
EU548039.1
TTCACTTAGACCACGAGCTTTATCACCATGCCTCGAGAAACCATCAACCCTTGCCTGAAC 400
AF207721.1
TTCACTTAGACCACGAGCTTTATCACCATGCCTCGAGAAACCATCAACCCTTGCCTGAAC 300
AF207724.1
TCCACTTAGATCACGAGCTTTATCACCATGCCTCGAGAAACCATCAACCCTTGCCTGAAC 300
JX982499.1
TTCACTTAGACCACGAGCTTTATCACCATGCCTCGAGAAACCATCAACCCTTGCCTGAAC 190
EU548041.1
TCCACTTAGACCACGAGCTTTATCACCATGCCTCGAGAAACCATCAACCCTTGCCTGAAC 400
EU548045.1
TCCACTTAGACCACGAGCTTTATCACCATGCCTCGAGAAACCATCAACCCTTGCCTGAAC 400
AF207725.1
TCCACTTAGACCACGAGCTTTATCACCATGCCTCGAGAAACCATCAACCCTTGCCTGAAC 300
EU548046.1
TCCACTTAGACCACGAGCTTTATCACCATGCCTCGAGAAACCATCAACCCTTGCCTGAAC 400
AF207714.1 --
------ 337
AF207713.1 --
------ 337
AF207712.1 --
------ 337
AY750628.1 --
------ 1044
EF689084.1 --
------ 1140
EF689085.1 --
------ 1140
AB119070.1 --
------ 956
EF987742.1 --
------ 1140
AB026105.1 --
------ 1140
MW148603.1
TCCACTTAGACCACGAGCTTTATCACCATGCCTCGAGAAACCATCAACCCTTGCCTGAAC 15659
AB051263.1 --
------ 402
AF068544.1 --
------ 337

JX982502.1
GTACACCTCTTCTCGCTCCGGGCCCATCAAATGTGGGGGTTCCTATCGTGAAACTATACC 249
JX982501.1
GTACACCTCTTCTCGCTCCGGGCCCATCAAATGTGGGGGTTCCTATCGTGAAACTATACC 249

JX982498.1
GTACACCTCTTCTCGCTCCGGGCCCATCAAATGTGGGGGTTTCTATCGTGAAACTATACC 249
JX982497.1
GTACACCTCTTCTCGCTCCGGGCCCATCAAATGTGGGGGTTCCTATCGTGAAACTATACC 249
JX982495.1
GTACACCTCTTCTCGCTCCGGGCCCATCAAATGTGGGGGTTCCTATCGTGAAACTATACC 249
JX982496.1
GTACACCTCTTCTCGCTCCGGGCCCATCAAATGTGGGGGTTCCTATCGTGAAACTATACC 249
JX982500.1
GTACACCTCTTCTCGCTCCGGGCCCATCAAATGTGGGGGTTCCTATCGTGAAACTATACC 249
EU548051.1
GTACACCTCTTCTCGCTCCGGGCCCATCAAATGTGGGGGTTCCTATCGTGAAACTATACC 458
EU548044.1
GTACACCTCTTCTCGCTCCGGGCCCATCAAATGTGGGGGTTTCTATCGTGAAACTATACC 458
EU548042.1
GTACACCTCTTCTCGCTCCGGGCCCATCAAATGTGGGGGTTTCTATCGTGAAACTATACC 458
EU548043.1
GTACACCTCTTCTCGCTCCGGGCCCATCAAATGTGGGGGTTTCTATCGTGAAACTATACC 458
EU548047.1
GTACACCTCTTCTCGCTCCGGGCCCATCAAATGTGGGGGTTCCTATCGTGAAACTATACC 459
EU548050.1
GTACACCTCTTCTCGCTCCGGGCCCATCAAATGTGGGGGTTCCTATCGTGAAACTATACC 459
EU548048.1
GTACACCTCTTCTCGCTCCGGGCCCATCAAATGTGGGGGTTCCTATCGTGAAACTATACC 459
EU548049.1
GTACACCTCTTCTCGCTCCGGGCCCATCGAATGTGGGGGTTCCTATCGTGAAACTATACC 459
AF207722.1
GTACACCTCTTCTCGCTCCGGGCCCATCAAATGTGGGGGTTCCTATCGTGAAACTATACC 359
EU548037.1
GTACACCTCTTCTCGCTCCGGGCCCATCAAATGTGGGGGTTCCTATCGTGAAACTATACC 459
AF207723.1
GTACACCTCTTCTCGCTCCGGGCCCATCAAATGTGGGGGTTCCTATCGTGAAACTATACC 359
EU548038.1
GTACACCTCTTCTCGCTCCGGGCCCATCAAATGTGGGGGTTCCTATCGTGAAACTATACC 459
EU548036.1
GTACACCTCTTCTCGCTCCGGGCCCATCAAATGTGGGGGTTCCTATCGTGAAACTATACC 459
EU548035.1
GTACACCTCTTCTCGCTCCGGGCCCATCAAATGTGGGGGTTCCTATCGTGAAACTATACC 459
AF207720.1
GTACACCTCTTCTCGCTCCGGGCCCATCAAATGTGGGGGTTCCTATCGTGAAACTATACC 359
AB601576.1
GTACACCTCTTCTCGCTCCGGGCCCATCAAATGTGGGGGTTCCTATCGTGAAACTATACC 289
EU548040.1
GTACACCTCTTCTCGCTCCGGGCCCATCAAATGTGGGGGTTCCTATCGTGAAACTATACC 460
EU548039.1
GTACACCTCTTCTCGCTCCGGGCCCATCAAATGTGGGGGTTCCTATCGTGAAACTATACC 460
AF207721.1
GTACACCTCTTCTCGCTCCGGGCCCATCAAATGTGGGGGTTCCTATCGTGAAACTATACC 360
AF207724.1
GTACACCTCTTCTCGCTCCGGGCCCATCAAATGTGGGGGTTCCTATCGTGAAACTATACC 360
JX982499.1
GTACACCTCTTCTCGCTCCGGGCCCATCAAATGTGGGGGTTCCTATCGTGAAACTATACC 250
EU548041.1
GTACACCTCTTCTCGCTCCGGGCCCATCAAATGTGGGGGTTTCTATCGTGAAACTATACC 460
EU548045.1
GTACACCTCTTCTCGCTCCGGGCCCATCGAATGTGGGGGTTTCTATCGTGAAACTATACC 460
AF207725.1
GTACACCTCTTCTCGCTCCGGGCCCATCGAATGTGGGGGTTTCTATCGTGAAACTATACC 360
EU548046.1
GTACACCTCTTCTCGCTCCGGGCCCATCAAATGTGGGGGTTTCTATCGTGAAACTATACC 460
AF207714.1 --
------ 337
AF207713.1 --
------ 337
AF207712.1 --
------ 337

```
   AY750628.1      ------------------------------------------------------
------ 1044
   EF689084.1      ------------------------------------------------------
------ 1140
   EF689085.1      ------------------------------------------------------
------ 1140
   AB119070.1      ------------------------------------------------------
------ 956
   EF987742.1      ------------------------------------------------------
------ 1140
   AB026105.1      ------------------------------------------------------
------ 1140
   MW148603.1
GTACACCTCTTCTCGCTCCGGGCCCATCAAATGTGGGGGTTCCTATCGTGAAACTATACC   15719
   AB051263.1      ------------------------------------------------------
------ 402
   AF068544.1      ------------------------------------------------------
------ 337


   JX982502.1
TGGCATCTGGTTCTTACTTCAGGGCCATACCAATCCTCAACCCAATCCTACTAACCTCTC   309
   JX982501.1
TGGCATCTGGTTCTTACTTCAGGGCCATATCAATCCTCAACCCAATCCTACTAACCTCTC   309
   JX982498.1
TGGCATCTGGTTCTTACTTCAGGGCCATATCAATCCTCAACCCAATCCTACTAACCTCTC   309
   JX982497.1
TGGCATCTGGTTCTTACTTCAGGGCCATATCAATCCTCAACCCAATCCTACTAACCTCTC   309
   JX982495.1
GGGCATCTGGTTCTTACTTCAGGGCCATATCAATCCTCAACCCAATCCTACTAACCTCTC   309
   JX982496.1
TGGCATCTGGTTCTTACTTCAGGGCCATATCAATCCTCAACCCAATCCTACTAACCTCTC   309
   JX982500.1
TGGCATCTGGTTCTTACTTCAGGGCCATATCAATCCTCAACCCAATCCTACTAACCTCTC   309
   EU548051.1      TGGCATCTGGTTCTTACTTCAGGGCCATATCAATCCTCAACCC-----------
------ 501
   EU548044.1      TGGCATCTGGTTCTTACTTCAGGGCCATATCAATCCTCAACCC-----------
------ 501
   EU548042.1      TGGCATCTGGTTCTTACTTCAGGGCCATATCAATCCTCAACCC-----------
------ 501
   EU548043.1      TGGCATCTGGTTCTTACTTCAGGGCCATATCAATCCTCAACCC-----------
------ 501
   EU548047.1      TGGCATCTGGTTCTTACTTCAGGGCCATATCAATCCTCAACCC-----------
------ 502
   EU548050.1      TGGCATCTGGTTCTTACTTCAGGGCCATATCAATCCTCAACCC-----------
------ 502
   EU548048.1      TGGCATCTGGTTCTTACTTCAGGGCCATATCAATCCTCAACCC-----------
------ 502
   EU548049.1      TGGCATCTGGTTCTTACTTCAGGGCCATATCAATCCTCAACCC-----------
------ 502
   AF207722.1      TGGCAT------------------------------------------------
------ 365
   EU548037.1      TGGCATCTGGTTCTTACTTCAGGGCCATATCAATCCTCAACCC-----------
------ 502
   AF207723.1      TGGCAT------------------------------------------------
------ 365
   EU548038.1      TGGCATCTGGTTCTTACTTCAGGGCCATATCAATCCTCAACCC-----------
------ 502
   EU548036.1      TGGCATCTGGTTCTTACTTCAGGGCCATATCAATCCTCAACCC-----------
------ 502
   EU548035.1      TGGCATCTGGTTCTTACTTCAGGGCCATATCAATCCTCAACCC-----------
------ 502
   AF207720.1      TGGCAT------------------------------------------------
------ 365
   AB601576.1
TGGCATCTGGTTCTTACTTCAGGGCCATATCAATCCTCAACCCAATCCTACTAACCTCTC   349
```

EU548040.1   TGGCATCTGGTTCTTACTTCAGGGCCATATCAATCCTCAACCC-----------
------ 503
EU548039.1   TGGCATCTGGTTCTTACTTCAGGGCCATATCAATCCTCAACCC-----------
------ 503
AF207721.1   TGGCAT--
------ 366
AF207724.1   TGGCAT--
------ 366
JX982499.1
TGGCATCTGGTTCTTACTTCAGGGCCATATCAATCCTCAACCCAATCCTACTAACCTCTC   310
EU548041.1   TGGCATCTGGTTCTTACTTCAGGGCCATATCAACCCTCAACCC-----------
------ 503
EU548045.1   TGGCATCTGGTTCTTACTTCAGGGCCATATCAATCCTCAACCC-----------
------ 503
AF207725.1   TGGCAT--
------ 366
EU548046.1   TGGCATCTGGTTCTTACTTCAGGGCCATATCAATCCTCAACCC-----------
------ 503
AF207714.1   --
------ 337
AF207713.1   --
------ 337
AF207712.1   --
------ 337
AY750628.1   --
------ 1044
EF689084.1   --
------ 1140
EF689085.1   --
------ 1140
AB119070.1   --
------ 956
EF987742.1   --
------ 1140
AB026105.1   --
------ 1140
MW148603.1
TGGCATCTGGTTCTTACTTCAGGGCCATATCAATCCTCAACCCAATCCTACTAACCTCTC   15779
AB051263.1   --
------ 402
AF068544.1   --
------ 337

JX982502.1
AAATGGGACATCTCGATGGACTAATGACTAATCAGCCCATGATCACACATAACTGTGGTG   369
JX982501.1
AAATGGGACATCTCGATGGACTAATGACTAATCAGCCCATGATCACACATAACTGTGGTG   369
JX982498.1
AAATGGGACATCTCGATGGACTAATGACTAATCAGCCCATGATCACACATAACTGTGGTG   369
JX982497.1
AAATGGGACATCTCGATGGACTAATGACTAATCAGCCCATGATCACACATAACTGTGGTG   369
JX982495.1
AAATGGGACATCTCGATGGACTAATGACTAATCAGCCCATGATCACACATAACTGTGGTG   369
JX982496.1
AAATGGGACATCTCGATGGACTAATGACTAATCAGCCCATGATCACACATAACTGTGGTG   369
JX982500.1
AAATGGGACATCTCGATGGACTAATGACTAATCAGCCCATGATCACACATAACTGTGGTG   369
EU548051.1   --
------ 501
EU548044.1   --
------ 501
EU548042.1   --
------ 501
EU548043.1   --
------ 501

```
    EU548047.1      ------------------------------------------------------
------ 502
    EU548050.1      ------------------------------------------------------
------ 502
    EU548048.1      ------------------------------------------------------
------ 502
    EU548049.1      ------------------------------------------------------
------ 502
    AF207722.1      ------------------------------------------------------
------ 365
    EU548037.1      ------------------------------------------------------
------ 502
    AF207723.1      ------------------------------------------------------
------ 365
    EU548038.1      ------------------------------------------------------
------ 502
    EU548036.1      ------------------------------------------------------
------ 502
    EU548035.1      ------------------------------------------------------
------ 502
    AF207720.1      ------------------------------------------------------
------ 365
    AB601576.1
AAATGGGACATCTCGATGGACTAATGACTAATCAGCCCATGATCACACATAACTGTGGTG    409
    EU548040.1      ------------------------------------------------------
------ 503
    EU548039.1      ------------------------------------------------------
------ 503
    AF207721.1      ------------------------------------------------------
------ 366
    AF207724.1      ------------------------------------------------------
------ 366
    JX982499.1
AAATGGGACATCTCGATGGACTAATGACTAATCAGCCCATGATCACACATAACTGTGGTG    370
    EU548041.1      ------------------------------------------------------
------ 503
    EU548045.1      ------------------------------------------------------
------ 503
    AF207725.1      ------------------------------------------------------
------ 366
    EU548046.1      ------------------------------------------------------
------ 503
    AF207714.1      ------------------------------------------------------
------ 337
    AF207713.1      ------------------------------------------------------
------ 337
    AF207712.1      ------------------------------------------------------
------ 337
    AY750628.1      ------------------------------------------------------
------ 1044
    EF689084.1      ------------------------------------------------------
------ 1140
    EF689085.1      ------------------------------------------------------
------ 1140
    AB119070.1      ------------------------------------------------------
------ 956
    EF987742.1      ------------------------------------------------------
------ 1140
    AB026105.1      ------------------------------------------------------
------ 1140
    MW148603.1
AAATGGGACATCTCGATGGACTAATGACTAATCAGCCCATGATCACACATAACTGTGGTG    15839
    AB051263.1      ------------------------------------------------------
------ 402
    AF068544.1      ------------------------------------------------------
------ 337
```

JX982502.1
TCATACATTTGGTATTTTTATTTTTTGGGGGGGGAGAACTTGGTATCACTCAGCTATGGC 429
JX982501.1
TCATACATTTGGTATTTTTATTTTTTGGGGGGGGAGAACTTGGTATCACTCAGCTATGGC 429
JX982498.1
TCATACATTTGGTATTTTTATTTTTTGGGGGGGGAGAACTTGGTATCACTCAGCTATGGC 429
JX982497.1
TCATACATTTGGTATTTTTATTTTTTGGGGGGGGAGAACTTGGTATCACTCAGCTATGGC 429
JX982495.1
TCATACATTTGGTATTTTTATTTTTTGGGGGGGGAGAACTTGGTATCACTCAGCTATGGC 429
JX982496.1
TCATACATTTGGTATTTTTATTTTTTGGGGGGGGAGAACTTGGTATCACTCAGCTATGGC 429
JX982500.1
TCATACATTTGGTATTTTTATTTTTTGGGGGGGGAGAACTTGGTATCACTCAGCTATGGC 429
EU548051.1 --
------ 501
EU548044.1 --
------ 501
EU548042.1 --
------ 501
EU548043.1 --
------ 501
EU548047.1 --
------ 502
EU548050.1 --
------ 502
EU548048.1 --
------ 502
EU548049.1 --
------ 502
AF207722.1 --
------ 365
EU548037.1 --
------ 502
AF207723.1 --
------ 365
EU548038.1 --
------ 502
EU548036.1 --
------ 502
EU548035.1 --
------ 502
AF207720.1 --
------ 365
AB601576.1
TCATACATTTGGTATTTTTATTTTTTGGGGGGGGAGAACTTGGTATCACTCAGCTATGGC 469
EU548040.1 --
------ 503
EU548039.1 --
------ 503
AF207721.1 --
------ 366
AF207724.1 --
------ 366
JX982499.1
TCATACATTTGGTATTTTTATTTTTTGGGGGGGGAGAACTTGGTATCACTCAGCTATGGC 430
EU548041.1 --
------ 503
EU548045.1 --
------ 503
AF207725.1 --
------ 366
EU548046.1 --
------ 503

```
    AF207714.1      ------------------------------------------------------
------ 337
    AF207713.1      ------------------------------------------------------
------ 337
    AF207712.1      ------------------------------------------------------
------ 337
    AY750628.1      ------------------------------------------------------
------ 1044
    EF689084.1      ------------------------------------------------------
------ 1140
    EF689085.1      ------------------------------------------------------
------ 1140
    AB119070.1      ------------------------------------------------------
------ 956
    EF987742.1      ------------------------------------------------------
------ 1140
    AB026105.1      ------------------------------------------------------
------ 1140
    MW148603.1
TCATACATTTGGTATTTTTATTTTTTGGGGGGGAGAACTTGGTATCACTCAGCTATGGC   15899
    AB051263.1      ------------------------------------------------------
------ 402
    AF068544.1      ------------------------------------------------------
------ 337


    JX982502.1
CAAGTATGGCCTCGTAGCAGTCAAATAATTTGTAGCTGGGCTTATCCTTCATCATTTATC   489
    JX982501.1
CAAGTATGGCCTCGTAGCAGTCAAATAATTTGTAGCTGGGCTTATCCTTCATCATTTATC   489
    JX982498.1
CAAGTATGGCCTCGTAGCAGTCAAATAATTTGTAGCTGGGCTTATCCTTCATCATTTATC   489
    JX982497.1
CAAGTATGGCCTCGTAGCAGTCAAATAATTTGTAGCTGGGCTTATCCTTCATCATTTATC   489
    JX982495.1
CTAGTATGGCCTCGTAGCAGTCAAATAATTTGTAGCTGGGCTTATCCTTCATCATTTATC   489
    JX982496.1
CAAGTATGGCCTCGTAGCAGTCAAATAATTTGTAGCTGGGCTTATCCTTCATCATTTATC   489
    JX982500.1
CAAGTATGGCCTCGTAGCAGTCAAATAATTTGTAGCTGGGCTTATCCTTCATCATTTATC   489
    EU548051.1      ------------------------------------------------------
------ 501
    EU548044.1      ------------------------------------------------------
------ 501
    EU548042.1      ------------------------------------------------------
------ 501
    EU548043.1      ------------------------------------------------------
------ 501
    EU548047.1      ------------------------------------------------------
------ 502
    EU548050.1      ------------------------------------------------------
------ 502
    EU548048.1      ------------------------------------------------------
------ 502
    EU548049.1      ------------------------------------------------------
------ 502
    AF207722.1      ------------------------------------------------------
------ 365
    EU548037.1      ------------------------------------------------------
------ 502
    AF207723.1      ------------------------------------------------------
------ 365
    EU548038.1      ------------------------------------------------------
------ 502
    EU548036.1      ------------------------------------------------------
------ 502
```

EU548035.1 --
------ 502
AF207720.1 --
------ 365
AB601576.1
CAAGTATGGCCTCGTAGCAGTCAAATAATTTGTAGCTGGGCTTATCCTTCATCATTTATC 529
EU548040.1 --
------ 503
EU548039.1 --
------ 503
AF207721.1 --
------ 366
AF207724.1 --
------ 366
JX982499.1
CAAGTATGGCCTCGTAGCAGTCAAATAACTTGTAGCTGGGCTTATCCTTCATCATTTATC 490
EU548041.1 --
------ 503
EU548045.1 --
------ 503
AF207725.1 --
------ 366
EU548046.1 --
------ 503
AF207714.1 --
------ 337
AF207713.1 --
------ 337
AF207712.1 --
------ 337
AY750628.1 --
------ 1044
EF689084.1 --
------ 1140
EF689085.1 --
------ 1140
AB119070.1 --
------ 956
EF987742.1 --
------ 1140
AB026105.1 --
------ 1140
MW148603.1
CAAGTATGGCCTCGTAGCAGTCAAATAATTTGTAGCTGGGCTTATCCTTCATCATTTATC 15959
AB051263.1 --
------ 402
AF068544.1 --
------ 337

JX982502.1 CGCATCGCATAGTCACAGGGTGTTATTCAGTCAATG------------------
------ 525
JX982501.1 CGCATCGCATAGTCACAGGGTGTTATTCAGTCAATG------------------
------ 525
JX982498.1 CGCATCGCATAGTCACAGGGTGTTATTCAGTCAATG------------------
------ 525
JX982497.1 CGCATCGCATAGTCACAGGGTGTTATTCAGTCAATG------------------
------ 525
JX982495.1 CGCATCGCATAGTCACAGGGTGTTATTCAGTCAATG------------------
------ 525
JX982496.1 CGCATCGCATAGTCACAGGGTGTTATTCAGTCAATG------------------
------ 525
JX982500.1 CGCATCGCATAGTTACAGGGTGTTATTCAGTCAATG------------------
------ 525
EU548051.1 --
------ 501

```
    EU548044.1          ------------------------------------------------------
------ 501
    EU548042.1          ------------------------------------------------------
------ 501
    EU548043.1          ------------------------------------------------------
------ 501
    EU548047.1          ------------------------------------------------------
------ 502
    EU548050.1          ------------------------------------------------------
------ 502
    EU548048.1          ------------------------------------------------------
------ 502
    EU548049.1          ------------------------------------------------------
------ 502
    AF207722.1          ------------------------------------------------------
------ 365
    EU548037.1          ------------------------------------------------------
------ 502
    AF207723.1          ------------------------------------------------------
------ 365
    EU548038.1          ------------------------------------------------------
------ 502
    EU548036.1          ------------------------------------------------------
------ 502
    EU548035.1          ------------------------------------------------------
------ 502
    AF207720.1          ------------------------------------------------------
------ 365
    AB601576.1          CGCATCGCATAGTCACAGGGTGTTATT---------------------------
------ 556
    EU548040.1          ------------------------------------------------------
------ 503
    EU548039.1          ------------------------------------------------------
------ 503
    AF207721.1          ------------------------------------------------------
------ 366
    AF207724.1          ------------------------------------------------------
------ 366
    JX982499.1          CGCATCGCATAGTCACAGGGTGTTATTCAGTCAATG------------------
------ 526
    EU548041.1          ------------------------------------------------------
------ 503
    EU548045.1          ------------------------------------------------------
------ 503
    AF207725.1          ------------------------------------------------------
------ 366
    EU548046.1          ------------------------------------------------------
------ 503
    AF207714.1          ------------------------------------------------------
------ 337
    AF207713.1          ------------------------------------------------------
------ 337
    AF207712.1          ------------------------------------------------------
------ 337
    AY750628.1          ------------------------------------------------------
------ 1044
    EF689084.1          ------------------------------------------------------
------ 1140
    EF689085.1          ------------------------------------------------------
------ 1140
    AB119070.1          ------------------------------------------------------
------ 956
    EF987742.1          ------------------------------------------------------
------ 1140
    AB026105.1          ------------------------------------------------------
------ 1140
```

```
    MW148603.1
CGCATCGCATAGTCACAGGGTGTTATTCAGTCAATGGTCACAGGACATACACACATATAT   16019
    AB051263.1      ------------------------------------------------------
------ 402
    AF068544.1      ------------------------------------------------------
------ 337


    JX982502.1      ------------------------------------------------------
------ 525
    JX982501.1      ------------------------------------------------------
------ 525
    JX982498.1      ------------------------------------------------------
------ 525
    JX982497.1      ------------------------------------------------------
------ 525
    JX982495.1      ------------------------------------------------------
------ 525
    JX982496.1      ------------------------------------------------------
------ 525
    JX982500.1      ------------------------------------------------------
------ 525
    EU548051.1      ------------------------------------------------------
------ 501
    EU548044.1      ------------------------------------------------------
------ 501
    EU548042.1      ------------------------------------------------------
------ 501
    EU548043.1      ------------------------------------------------------
------ 501
    EU548047.1      ------------------------------------------------------
------ 502
    EU548050.1      ------------------------------------------------------
------ 502
    EU548048.1      ------------------------------------------------------
------ 502
    EU548049.1      ------------------------------------------------------
------ 502
    AF207722.1      ------------------------------------------------------
------ 365
    EU548037.1      ------------------------------------------------------
------ 502
    AF207723.1      ------------------------------------------------------
------ 365
    EU548038.1      ------------------------------------------------------
------ 502
    EU548036.1      ------------------------------------------------------
------ 502
    EU548035.1      ------------------------------------------------------
------ 502
    AF207720.1      ------------------------------------------------------
------ 365
    AB601576.1      ------------------------------------------------------
------ 556
    EU548040.1      ------------------------------------------------------
------ 503
    EU548039.1      ------------------------------------------------------
------ 503
    AF207721.1      ------------------------------------------------------
------ 366
    AF207724.1      ------------------------------------------------------
------ 366
    JX982499.1      ------------------------------------------------------
------ 526
    EU548041.1      ------------------------------------------------------
------ 503
```

```
    EU548045.1      ------------------------------------------------------
------ 503
    AF207725.1      ------------------------------------------------------
------ 366
    EU548046.1      ------------------------------------------------------
------ 503
    AF207714.1      ------------------------------------------------------
------ 337
    AF207713.1      ------------------------------------------------------
------ 337
    AF207712.1      ------------------------------------------------------
------ 337
    AY750628.1      ------------------------------------------------------
------ 1044
    EF689084.1      ------------------------------------------------------
------ 1140
    EF689085.1      ------------------------------------------------------
------ 1140
    AB119070.1      ------------------------------------------------------
------ 956
    EF987742.1      ------------------------------------------------------
------ 1140
    AB026105.1      ------------------------------------------------------
------ 1140
    MW148603.1
CCACCCCCGTGCACACGTACGCACACGTACGCACACGTACGCACACGTACGCACACGTAC   16079
    AB051263.1      ------------------------------------------------------
------ 402
    AF068544.1      ------------------------------------------------------
------ 337


    JX982502.1      ------------------------------------------------------
------ 525
    JX982501.1      ------------------------------------------------------
------ 525
    JX982498.1      ------------------------------------------------------
------ 525
    JX982497.1      ------------------------------------------------------
------ 525
    JX982495.1      ------------------------------------------------------
------ 525
    JX982496.1      ------------------------------------------------------
------ 525
    JX982500.1      ------------------------------------------------------
------ 525
    EU548051.1      ------------------------------------------------------
------ 501
    EU548044.1      ------------------------------------------------------
------ 501
    EU548042.1      ------------------------------------------------------
------ 501
    EU548043.1      ------------------------------------------------------
------ 501
    EU548047.1      ------------------------------------------------------
------ 502
    EU548050.1      ------------------------------------------------------
------ 502
    EU548048.1      ------------------------------------------------------
------ 502
    EU548049.1      ------------------------------------------------------
------ 502
    AF207722.1      ------------------------------------------------------
------ 365
    EU548037.1      ------------------------------------------------------
------ 502
```

```
   AF207723.1      ------------------------------------------------------
------ 365
   EU548038.1      ------------------------------------------------------
------ 502
   EU548036.1      ------------------------------------------------------
------ 502
   EU548035.1      ------------------------------------------------------
------ 502
   AF207720.1      ------------------------------------------------------
------ 365
   AB601576.1      ------------------------------------------------------
------ 556
   EU548040.1      ------------------------------------------------------
------ 503
   EU548039.1      ------------------------------------------------------
------ 503
   AF207721.1      ------------------------------------------------------
------ 366
   AF207724.1      ------------------------------------------------------
------ 366
   JX982499.1      ------------------------------------------------------
------ 526
   EU548041.1      ------------------------------------------------------
------ 503
   EU548045.1      ------------------------------------------------------
------ 503
   AF207725.1      ------------------------------------------------------
------ 366
   EU548046.1      ------------------------------------------------------
------ 503
   AF207714.1      ------------------------------------------------------
------ 337
   AF207713.1      ------------------------------------------------------
------ 337
   AF207712.1      ------------------------------------------------------
------ 337
   AY750628.1      ------------------------------------------------------
------ 1044
   EF689084.1      ------------------------------------------------------
------ 1140
   EF689085.1      ------------------------------------------------------
------ 1140
   AB119070.1      ------------------------------------------------------
------ 956
   EF987742.1      ------------------------------------------------------
------ 1140
   AB026105.1      ------------------------------------------------------
------ 1140
   MW148603.1
GCACACGTACGCACACGTACGCACACGTACGCACACGTACGCACACGTACGCACACGTAC   16139
   AB051263.1      ------------------------------------------------------
------ 402
   AF068544.1      ------------------------------------------------------
------ 337


   JX982502.1      ------------------------------------------------------
------ 525
   JX982501.1      ------------------------------------------------------
------ 525
   JX982498.1      ------------------------------------------------------
------ 525
   JX982497.1      ------------------------------------------------------
------ 525
   JX982495.1      ------------------------------------------------------
------ 525
```

```
    JX982496.1          ------------------------------------------------------
------ 525
    JX982500.1          ------------------------------------------------------
------ 525
    EU548051.1          ------------------------------------------------------
------ 501
    EU548044.1          ------------------------------------------------------
------ 501
    EU548042.1          ------------------------------------------------------
------ 501
    EU548043.1          ------------------------------------------------------
------ 501
    EU548047.1          ------------------------------------------------------
------ 502
    EU548050.1          ------------------------------------------------------
------ 502
    EU548048.1          ------------------------------------------------------
------ 502
    EU548049.1          ------------------------------------------------------
------ 502
    AF207722.1          ------------------------------------------------------
------ 365
    EU548037.1          ------------------------------------------------------
------ 502
    AF207723.1          ------------------------------------------------------
------ 365
    EU548038.1          ------------------------------------------------------
------ 502
    EU548036.1          ------------------------------------------------------
------ 502
    EU548035.1          ------------------------------------------------------
------ 502
    AF207720.1          ------------------------------------------------------
------ 365
    AB601576.1          ------------------------------------------------------
------ 556
    EU548040.1          ------------------------------------------------------
------ 503
    EU548039.1          ------------------------------------------------------
------ 503
    AF207721.1          ------------------------------------------------------
------ 366
    AF207724.1          ------------------------------------------------------
------ 366
    JX982499.1          ------------------------------------------------------
------ 526
    EU548041.1          ------------------------------------------------------
------ 503
    EU548045.1          ------------------------------------------------------
------ 503
    AF207725.1          ------------------------------------------------------
------ 366
    EU548046.1          ------------------------------------------------------
------ 503
    AF207714.1          ------------------------------------------------------
------ 337
    AF207713.1          ------------------------------------------------------
------ 337
    AF207712.1          ------------------------------------------------------
------ 337
    AY750628.1          ------------------------------------------------------
------ 1044
    EF689084.1          ------------------------------------------------------
------ 1140
    EF689085.1          ------------------------------------------------------
------ 1140
```

```
   AB119070.1      ------------------------------------------------------
------ 956
   EF987742.1      ------------------------------------------------------
------ 1140
   AB026105.1      ------------------------------------------------------
------ 1140
   MW148603.1
GCACACGTACGCACACGTACGCACACGTACGCACACGTACGCACACGTACACACGTACGC   16199
   AB051263.1      ------------------------------------------------------
------ 402
   AF068544.1      ------------------------------------------------------
------ 337


   JX982502.1      ------------------------------------------------------
------ 525
   JX982501.1      ------------------------------------------------------
------ 525
   JX982498.1      ------------------------------------------------------
------ 525
   JX982497.1      ------------------------------------------------------
------ 525
   JX982495.1      ------------------------------------------------------
------ 525
   JX982496.1      ------------------------------------------------------
------ 525
   JX982500.1      ------------------------------------------------------
------ 525
   EU548051.1      ------------------------------------------------------
------ 501
   EU548044.1      ------------------------------------------------------
------ 501
   EU548042.1      ------------------------------------------------------
------ 501
   EU548043.1      ------------------------------------------------------
------ 501
   EU548047.1      ------------------------------------------------------
------ 502
   EU548050.1      ------------------------------------------------------
------ 502
   EU548048.1      ------------------------------------------------------
------ 502
   EU548049.1      ------------------------------------------------------
------ 502
   AF207722.1      ------------------------------------------------------
------ 365
   EU548037.1      ------------------------------------------------------
------ 502
   AF207723.1      ------------------------------------------------------
------ 365
   EU548038.1      ------------------------------------------------------
------ 502
   EU548036.1      ------------------------------------------------------
------ 502
   EU548035.1      ------------------------------------------------------
------ 502
   AF207720.1      ------------------------------------------------------
------ 365
   AB601576.1      ------------------------------------------------------
------ 556
   EU548040.1      ------------------------------------------------------
------ 503
   EU548039.1      ------------------------------------------------------
------ 503
   AF207721.1      ------------------------------------------------------
------ 366
```

```
    AF207724.1      ------------------------------------------------------
------ 366
    JX982499.1      ------------------------------------------------------
------ 526
    EU548041.1      ------------------------------------------------------
------ 503
    EU548045.1      ------------------------------------------------------
------ 503
    AF207725.1      ------------------------------------------------------
------ 366
    EU548046.1      ------------------------------------------------------
------ 503
    AF207714.1      ------------------------------------------------------
------ 337
    AF207713.1      ------------------------------------------------------
------ 337
    AF207712.1      ------------------------------------------------------
------ 337
    AY750628.1      ------------------------------------------------------
------ 1044
    EF689084.1      ------------------------------------------------------
------ 1140
    EF689085.1      ------------------------------------------------------
------ 1140
    AB119070.1      ------------------------------------------------------
------ 956
    EF987742.1      ------------------------------------------------------
------ 1140
    AB026105.1      ------------------------------------------------------
------ 1140
    MW148603.1
ACACGTACACACGTACACACGTACACACGTACGCACACGGTACGTATACACGTATCCAAC   16259
    AB051263.1      ------------------------------------------------------
------ 402
    AF068544.1      ------------------------------------------------------
------ 337


    JX982502.1      ------------------------------------------------------
------ 525
    JX982501.1      ------------------------------------------------------
------ 525
    JX982498.1      ------------------------------------------------------
------ 525
    JX982497.1      ------------------------------------------------------
------ 525
    JX982495.1      ------------------------------------------------------
------ 525
    JX982496.1      ------------------------------------------------------
------ 525
    JX982500.1      ------------------------------------------------------
------ 525
    EU548051.1      ------------------------------------------------------
------ 501
    EU548044.1      ------------------------------------------------------
------ 501
    EU548042.1      ------------------------------------------------------
------ 501
    EU548043.1      ------------------------------------------------------
------ 501
    EU548047.1      ------------------------------------------------------
------ 502
    EU548050.1      ------------------------------------------------------
------ 502
    EU548048.1      ------------------------------------------------------
------ 502
```

```
   EU548049.1      ------------------------------------------------------
------ 502
   AF207722.1      ------------------------------------------------------
------ 365
   EU548037.1      ------------------------------------------------------
------ 502
   AF207723.1      ------------------------------------------------------
------ 365
   EU548038.1      ------------------------------------------------------
------ 502
   EU548036.1      ------------------------------------------------------
------ 502
   EU548035.1      ------------------------------------------------------
------ 502
   AF207720.1      ------------------------------------------------------
------ 365
   AB601576.1      ------------------------------------------------------
------ 556
   EU548040.1      ------------------------------------------------------
------ 503
   EU548039.1      ------------------------------------------------------
------ 503
   AF207721.1      ------------------------------------------------------
------ 366
   AF207724.1      ------------------------------------------------------
------ 366
   JX982499.1      ------------------------------------------------------
------ 526
   EU548041.1      ------------------------------------------------------
------ 503
   EU548045.1      ------------------------------------------------------
------ 503
   AF207725.1      ------------------------------------------------------
------ 366
   EU548046.1      ------------------------------------------------------
------ 503
   AF207714.1      ------------------------------------------------------
------ 337
   AF207713.1      ------------------------------------------------------
------ 337
   AF207712.1      ------------------------------------------------------
------ 337
   AY750628.1      ------------------------------------------------------
------ 1044
   EF689084.1      ------------------------------------------------------
------ 1140
   EF689085.1      ------------------------------------------------------
------ 1140
   AB119070.1      ------------------------------------------------------
------ 956
   EF987742.1      ------------------------------------------------------
------ 1140
   AB026105.1      ------------------------------------------------------
------ 1140
   MW148603.1
AGATAAGAATTAACCTTTGTCAAACCCCCCTTACCCCCCGTAACTTCAAAAGTATACAAA   16319
   AB051263.1      ------------------------------------------------------
------ 402
   AF068544.1      ------------------------------------------------------
------ 337


   JX982502.1      ------------------------------------------------------
------ 525
   JX982501.1      ------------------------------------------------------
------ 525
```

```
    JX982498.1      ------------------------------------------------------
------ 525
    JX982497.1      ------------------------------------------------------
------ 525
    JX982495.1      ------------------------------------------------------
------ 525
    JX982496.1      ------------------------------------------------------
------ 525
    JX982500.1      ------------------------------------------------------
------ 525
    EU548051.1      ------------------------------------------------------
------ 501
    EU548044.1      ------------------------------------------------------
------ 501
    EU548042.1      ------------------------------------------------------
------ 501
    EU548043.1      ------------------------------------------------------
------ 501
    EU548047.1      ------------------------------------------------------
------ 502
    EU548050.1      ------------------------------------------------------
------ 502
    EU548048.1      ------------------------------------------------------
------ 502
    EU548049.1      ------------------------------------------------------
------ 502
    AF207722.1      ------------------------------------------------------
------ 365
    EU548037.1      ------------------------------------------------------
------ 502
    AF207723.1      ------------------------------------------------------
------ 365
    EU548038.1      ------------------------------------------------------
------ 502
    EU548036.1      ------------------------------------------------------
------ 502
    EU548035.1      ------------------------------------------------------
------ 502
    AF207720.1      ------------------------------------------------------
------ 365
    AB601576.1      ------------------------------------------------------
------ 556
    EU548040.1      ------------------------------------------------------
------ 503
    EU548039.1      ------------------------------------------------------
------ 503
    AF207721.1      ------------------------------------------------------
------ 366
    AF207724.1      ------------------------------------------------------
------ 366
    JX982499.1      ------------------------------------------------------
------ 526
    EU548041.1      ------------------------------------------------------
------ 503
    EU548045.1      ------------------------------------------------------
------ 503
    AF207725.1      ------------------------------------------------------
------ 366
    EU548046.1      ------------------------------------------------------
------ 503
    AF207714.1      ------------------------------------------------------
------ 337
    AF207713.1      ------------------------------------------------------
------ 337
    AF207712.1      ------------------------------------------------------
------ 337
```

```
    AY750628.1       ------------------------------------------------------
------ 1044
    EF689084.1       ------------------------------------------------------
------ 1140
    EF689085.1       ------------------------------------------------------
------ 1140
    AB119070.1       ------------------------------------------------------
------ 956
    EF987742.1       ------------------------------------------------------
------ 1140
    AB026105.1       ------------------------------------------------------
------ 1140
    MW148603.1
TACTTATAATCGCTCTGCCAAACCCCAAAAACAGAACTAAGCACATGCAACATATATTAG   16379
    AB051263.1       ------------------------------------------------------
------ 402
    AF068544.1       ------------------------------------------------------
------ 337


    JX982502.1       ------------------------------------------------------
------ 525
    JX982501.1       ------------------------------------------------------
------ 525
    JX982498.1       ------------------------------------------------------
------ 525
    JX982497.1       ------------------------------------------------------
------ 525
    JX982495.1       ------------------------------------------------------
------ 525
    JX982496.1       ------------------------------------------------------
------ 525
    JX982500.1       ------------------------------------------------------
------ 525
    EU548051.1       ------------------------------------------------------
------ 501
    EU548044.1       ------------------------------------------------------
------ 501
    EU548042.1       ------------------------------------------------------
------ 501
    EU548043.1       ------------------------------------------------------
------ 501
    EU548047.1       ------------------------------------------------------
------ 502
    EU548050.1       ------------------------------------------------------
------ 502
    EU548048.1       ------------------------------------------------------
------ 502
    EU548049.1       ------------------------------------------------------
------ 502
    AF207722.1       ------------------------------------------------------
------ 365
    EU548037.1       ------------------------------------------------------
------ 502
    AF207723.1       ------------------------------------------------------
------ 365
    EU548038.1       ------------------------------------------------------
------ 502
    EU548036.1       ------------------------------------------------------
------ 502
    EU548035.1       ------------------------------------------------------
------ 502
    AF207720.1       ------------------------------------------------------
------ 365
    AB601576.1       ------------------------------------------------------
------ 556
```

EU548040.1 --
------ 503
EU548039.1 --
------ 503
AF207721.1 --
------ 366
AF207724.1 --
------ 366
JX982499.1 --
------ 526
EU548041.1 --
------ 503
EU548045.1 --
------ 503
AF207725.1 --
------ 366
EU548046.1 --
------ 503
AF207714.1 --
------ 337
AF207713.1 --
------ 337
AF207712.1 --
------ 337
AY750628.1 --
------ 1044
EF689084.1 --
------ 1140
EF689085.1 --
------ 1140
AB119070.1 --
------ 956
EF987742.1 --
------ 1140
AB026105.1 --
------ 1140
MW148603.1
AAGTCACTAATACTGACACCAATTGACCCATTGAATAATTCCTATCTAGAAGACTATCTA 16439
AB051263.1 --
------ 402
AF068544.1 --
------ 337

JX982502.1 --
------ 525
JX982501.1 --
------ 525
JX982498.1 --
------ 525
JX982497.1 --
------ 525
JX982495.1 --
------ 525
JX982496.1 --
------ 525
JX982500.1 --
------ 525
EU548051.1 --
------ 501
EU548044.1 --
------ 501
EU548042.1 --
------ 501
EU548043.1 --
------ 501

```
   EU548047.1      ------------------------------------------------------
------ 502
   EU548050.1      ------------------------------------------------------
------ 502
   EU548048.1      ------------------------------------------------------
------ 502
   EU548049.1      ------------------------------------------------------
------ 502
   AF207722.1      ------------------------------------------------------
------ 365
   EU548037.1      ------------------------------------------------------
------ 502
   AF207723.1      ------------------------------------------------------
------ 365
   EU548038.1      ------------------------------------------------------
------ 502
   EU548036.1      ------------------------------------------------------
------ 502
   EU548035.1      ------------------------------------------------------
------ 502
   AF207720.1      ------------------------------------------------------
------ 365
   AB601576.1      ------------------------------------------------------
------ 556
   EU548040.1      ------------------------------------------------------
------ 503
   EU548039.1      ------------------------------------------------------
------ 503
   AF207721.1      ------------------------------------------------------
------ 366
   AF207724.1      ------------------------------------------------------
------ 366
   JX982499.1      ------------------------------------------------------
------ 526
   EU548041.1      ------------------------------------------------------
------ 503
   EU548045.1      ------------------------------------------------------
------ 503
   AF207725.1      ------------------------------------------------------
------ 366
   EU548046.1      ------------------------------------------------------
------ 503
   AF207714.1      ------------------------------------------------------
------ 337
   AF207713.1      ------------------------------------------------------
------ 337
   AF207712.1      ------------------------------------------------------
------ 337
   AY750628.1      ------------------------------------------------------
------ 1044
   EF689084.1      ------------------------------------------------------
------ 1140
   EF689085.1      ------------------------------------------------------
------ 1140
   AB119070.1      ------------------------------------------------------
------ 956
   EF987742.1      ------------------------------------------------------
------ 1140
   AB026105.1      ------------------------------------------------------
------ 1140
   MW148603.1
TAGATGTATTTATAACCCTTAACACTTATTACAAAACTTAAAATACCCTTTCTCACCCCC   16499
   AB051263.1      ------------------------------------------------------
------ 402
   AF068544.1      ------------------------------------------------------
------ 337
```

JX982502.1	-----	525
JX982501.1	-----	525
JX982498.1	-----	525
JX982497.1	-----	525
JX982495.1	-----	525
JX982496.1	-----	525
JX982500.1	-----	525
EU548051.1	-----	501
EU548044.1	-----	501
EU548042.1	-----	501
EU548043.1	-----	501
EU548047.1	-----	502
EU548050.1	-----	502
EU548048.1	-----	502
EU548049.1	-----	502
AF207722.1	-----	365
EU548037.1	-----	502
AF207723.1	-----	365
EU548038.1	-----	502
EU548036.1	-----	502
EU548035.1	-----	502
AF207720.1	-----	365
AB601576.1	-----	556
EU548040.1	-----	503
EU548039.1	-----	503
AF207721.1	-----	366
AF207724.1	-----	366
JX982499.1	-----	526
EU548041.1	-----	503
EU548045.1	-----	503
AF207725.1	-----	366
EU548046.1	-----	503
AF207714.1	-----	337
AF207713.1	-----	337
AF207712.1	-----	337
AY750628.1	-----	1044
EF689084.1	-----	1140
EF689085.1	-----	1140
AB119070.1	-----	956
EF987742.1	-----	1140
AB026105.1	-----	1140
MW148603.1	TCTCC	16504
AB051263.1	-----	402
AF068544.1	-----	337

Figure S6. Multiple alignment for the 43 mtDNA sequences of *Mustela lutreola* deposited in the GenBank [23] aligned to the MW148603 reference sequence, reported in this paper.
